# Effects of physical exercise and body weight on disease-specific outcomes of people with rheumatic and musculoskeletal diseases (RMDs): systematic reviews and meta-analyses informing the 2021 EULAR recommendations for lifestyle improvements in people with RMDs

**DOI:** 10.1136/rmdopen-2021-002168

**Published:** 2022-03-30

**Authors:** James M Gwinnutt, Maud Wieczorek, Giulio Cavalli, Andra Balanescu, Heike A Bischoff-Ferrari, Annelies Boonen, Savia de Souza, Annette de Thurah, Thomas E Dorner, Rikke Helene Moe, Polina Putrik, Javier Rodríguez-Carrio, Lucía Silva-Fernández, Tanja Stamm, Karen Walker-Bone, Joep Welling, Mirjana I Zlatković-Švenda, Francis Guillemin, Suzanne M M Verstappen

**Affiliations:** 1 Centre for Epidemiology Versus Arthritis, Faculty of Biology, Medicine and Health, The University of Manchester, Manchester, UK; 2 EA 4360 Apemac, Université de Lorraine, Nancy, France; 3 Center on Aging and Mobility, University of Zurich, Zurich, Switzerland; 4 Unit of Immunology, Rheumatology, Allergy and Rare Diseases, IRCCS San Raffaele Hospital and Vita-Salute San Raffaele University, Milan, Italy; 5 Department of Internal Medicine and Rheumatology, “Sf. Maria” Hospital, “Carol Davila” University of Medicine and Pharmacy, Bucharest, Romania; 6 Department of Aging Medicine and Aging Research, University Hospital Zurich and University of Zurich, Zurich, Switzerland; 7 University Clinic for Aging Medicine, City Hospital Zurich - Waid, Zurich, Switzerland; 8 Department of Internal Medicine, Division of Rheumatology, Maastricht University Medical Center, Maastricht, The Netherlands; 9 Care and Public Health Research Institute (CAPHRI), Maastricht University, Maastricht, The Netherlands; 10 Centre for Rheumatic Diseases, King's College London, London, UK; 11 Department of Clinical Medicine, Aarhus University, Aarhus, Denmark; 12 Department of Rheumatology, Aarhus University Hospital, Aarhus, Denmark; 13 Centre for Public Health, Department of Social and Preventive Medicine, Medical University of Vienna, Vienna, Austria; 14 Social Insurance Fund for Public Service, Railway and Mining Industries, Sitzenberg-Reidling, Austria; 15 Karl-Landsteiner Institute for Health Promotion Research, Sitzenberg-Reidling, Austria; 16 National Advisory Unit for Rehabilitation in Rheumatology, Division of Rheumatology and Research, Diakonhjemmet Hospital, Oslo, Norway; 17 Area of Immunology, Department of Functional Biology, Universidad de Oviedo, Oviedo, Spain; 18 Department of Metabolism, Instituto de Investigación Sanitaria del Principado de Asturias (ISPA), Oviedo, Spain; 19 Rheumatology Department, Hospital Universitari Son Espases, Palma de Mallorca, Spain; 20 Section for Outcomes Research, Center for Medical Statistics, Informatics, and Intelligent Systems, Medical University of Vienna, Vienna, Austria; 21 Ludwig Boltzmann Institute for Arthritis and Rehabilitation, Vienna, Austria; 22 MRC Versus Arthritis Centre for Musculoskeletal Health and Work, University of Southampton, Southampton, UK; 23 NVLE Dutch Patient Organization for Systemic Autoimmune Diseases, Utrecht, The Netherlands; 24 Institute of Rheumatology, University of Belgrade School of Medicine, Belgrade, Serbia; 25 Department of Internal Medicine, University of East Sarajevo Faculty of Medicine Foča, Republika Srpska, Bosnia and Herzegovina; 26 Inserm, CHRU Nancy, CIC-1433 Epidémiologie Clinique, Université de Lorraine, Nancy, France; 27 NIHR Manchester Biomedical Research Centre, Manchester University NHS Foundation Trust, Manchester Academic Health Science Centre, Manchester, UK

**Keywords:** epidemiology, arthritis, patient reported outcome measures, physical therapy modalities

## Abstract

**Background:**

A European League Against Rheumatism (EULAR) taskforce was convened to develop recommendations for lifestyle behaviours in rheumatic and musculoskeletal diseases (RMDs). This paper reviews the literature on the effects of physical exercise and body weight on disease-specific outcomes of people with RMDs.

**Methods:**

Three systematic reviews were conducted to summarise evidence related to exercise and weight in seven RMDs: osteoarthritis, rheumatoid arthritis, systemic lupus erythematosus, axial spondyloarthritis (axSpA), psoriatic arthritis, systemic sclerosis and gout. Systematic reviews and original studies were included if they assessed exercise or weight in one of the above RMDs, and reported results regarding disease-specific outcomes (eg, pain, function, joint damage). Systematic reviews were only included if published between 2013–2018. Search strategies were implemented in the Medline, Embase, Cochrane Library of systematic reviews and CENTRAL databases.

**Results:**

236 articles on exercise and 181 articles on weight were included. Exercise interventions resulted in improvements in outcomes such as pain and function across all the RMDs, although the size of the effect varied by RMD and intervention. Disease activity was not influenced by exercise, other than in axSpA. Increased body weight was associated with worse outcomes for the majority of RMDs and outcomes assessed. In general, study quality was moderate for the literature on exercise and body weight in RMDs, although there was large heterogeneity between studies.

**Conclusion:**

The current literature supports recommending exercise and the maintenance of a healthy body weight for people with RMDs.

**Key messages**

**What is already known about this subject?**

- Body weight and physical exercise are important for health.
- It is unclear whether body weight or change in body weight and exercise influence outcomes in rheumatic and musculoskeletal diseases (RMDs).

**What does this study add?**

- This study summarises the literature on the association between exercise and weight with disease outcomes of seven RMDs, concluding that performing exercise and a healthy weight are associated with better outcomes in people with RMDs.

**How might this impact on clinical practice or further developments?**

- People with RMDs should be encouraged to perform exercise if they do not currently perform exercise, or maintain exercise if performing sufficient quantities.
- People with RMDs should be encouraged and supported to attain and maintain a healthy body weight.

## Introduction

Rheumatic and musculoskeletal diseases (RMDs) comprise a wide range of conditions characterised by pain, disability and poorer quality of life (QoL).^1–3^ Globally, these conditions comprise a significant burden which is continuing to increase. For instance, the Global Burden of Disease study reported that the percentage increase of disability adjusted life years driven by RMDs (other than lower back pain) was 128.9% (95% CI 122.0% to 136.3%) between 1990 and 2019 across all age groups.^4^ While some RMDs have many effective pharmacological treatments (eg, rheumatoid arthritis (RA)^5^), for some the treatment options are limited (eg, systemic lupus erythematosus (SLE)^6^) and for others there are no effective disease modifying treatments (eg, osteoarthritis (OA)^7^). However, there is room for additional improvements in outcomes in all RMDs, potentially through modification of lifestyle behaviour.

Physical activity (including exercise)^8^ is clearly beneficial for health, regardless of the presence of chronic diseases such as RMDs. The WHO and American institutions recommend that all adults aged 18–65 years should participate in at least 150 min per week of moderate-intensity aerobic physical activity, or do at least 75 min of vigorous-intensity aerobic physical activity, or an equivalent combination of moderate- and vigorous-intensity activity (moderate intensity=3.0 to <6.0 METs (metabolic equivalent of task), vigorous intensity =≥6 METs, where 1 MET is the rate of energy expenditure at rest). Additionally, all adults aged 18–65 years should perform muscle-strengthening activities (eg, resistance training and weight lifting) involving major muscle groups on two or more days a week.^9 10^ In 2018, the European League Against Rheumatism (EULAR) recommended physical activity for people with inflammatory arthritis and OA,^11^ after a systematic review illustrating the benefits of exercise on strength, flexibility and cardiovascular fitness.^12^ Furthermore, exercise is closely linked to body weight. The prevalence of global obesity is increasing and obesity, as well as physical inactivity, is associated with poor health, comorbidity (eg, cardiovascular disease,^13^ diabetes,^14^) and increased risk of mortality.^13^ Therefore, there is an urgent need for strategies to ameliorate the obesity epidemic for the benefit of global health.

However, it is unclear whether exercise is effective at improving RMD-specific outcomes (eg, pain, disability), or whether excess weight is associated with worse RMD outcomes. Therefore, a EULAR taskforce was convened in 2018 to develop recommendations for lifestyle improvements in RMDs, with the focus on lifestyles that affect disease progression. The taskforce decided to focus on six lifestyle factors: diet, exercise, body weight, alcohol, smoking and (paid) work-participation, in seven diseases: RA, OA, SLE, axial spondyloarthritis (axSpA), psoriatic arthritis (PsA), systemic sclerosis (SSc) and gout (henceforth referred to collectively as RMDs). For each of these lifestyle factors, systematic reviews were performed, aiming to collate all relevant literature on each factor in order to formulate evidence-based recommendations. This article presents the results of the systematic reviews assessing the impact of exercise and body weight on the disease-specific outcomes of people with RMDs.

## Methods

### Design

These reviews were conducted following EULAR’s standard operating procedure for EULAR endorsed recommendations^15^ and are reported according to the Preferred Reporting Items for Systematic Reviews and Meta-Analyses guidelines.^16^

### Search strategy

A two-step process was used to identify studies to include in each review. Initially, a review of systematic reviews was conducted using the MEDLINE, EMBASE and Cochrane Library databases, aiming to identify existing systematic reviews on the included exposures and RMD progression (online supplemental tables 1 and 2, defined a priori by the study team) that were published from 1 January 2013 to 18 September 2018. Two reviewers screened the titles and abstracts (JMG and MW) and then a team of four reviewers screened the eligible full texts (JMG, MW, JR-C and GC; each full text screened by two reviewers). Only existing systematic reviews relating to exercise and weight are presented here.

10.1136/rmdopen-2021-002168.supp1 Supplementary data

Following this, separate systematic reviews of original studies of exercise and weight in RMDs were conducted (from inception to search date). It was decided that there were sufficient numbers of published systematic reviews regarding exercise and OA and therefore OA was not included in the systematic review of original studies of exercise. Search strategies for each review were developed based on a predefined PICO strategy (participants, intervention/exposure, comparison, outcome) (online supplemental tables 3 and 4 for search strategies) and implemented in the MEDLINE, EMBASE and CENTRAL databases (dates when strategies were implemented: exercise: 18 March 2019; weight: 14 March 2019). Titles and abstracts, followed by full texts, were screened by two reviewers (exercise: JMG and GC; weight: JMG and SMMV).

### Inclusion and exclusion criteria

Systematic reviews were included if they:

- Included adults with an RMD (OA, RA, SLE, axSpA, PsA, SSc, gout).
- Studied the relationship between exercise or weight and disease-specific outcomes (online supplemental table 5 for list of included outcomes).
- Published in English, French, Spanish or Italian.

No restrictions were implemented regarding the included study designs of studies in systematic reviews.

Original studies were included if they:

- Used a longitudinal study design (randomised controlled trials (RCTs), non-randomised trials, single-arm intervention studies, longitudinal observational studies)
- Included adults with an RMD (RA, SLE, axSpA, PsA, SSc, gout (and OA for the weight review)).
- Studied the relationship between exercise or weight and outcomes (online supplemental table 5 for list of included outcomes).
- Published in English, French, Spanish or Italian.

Conference abstracts were excluded.

### Risk of bias assessment

To assess the risk of bias of included systematic reviews and meta-analyses, the AMSTAR-2 tool was used.^17^ Each included review or meta-analysis was rated as critically low, low, moderate or high quality. For included RCTs, an abbreviated version of the Cochrane Risk of Bias tool was used,^18^ assessing four criteria: randomisation procedure, allocation concealment procedure, blinding of participants and blinding of assessors. Each aspect was rated as either low or high/unclear risk of bias. A machine learning algorithm was used to assist the process,^19^ which identifies passages of text from included manuscripts and assigns grades for each criteria. A reviewer (JMG) checked each of the algorithm’s grades and if there was disagreement, changed the grade. The QUIPS tool was used to assess the quality of observational studies across six domains: study population, attrition, exposure measurement, outcome measurement, confounding and statistical analysis.^20^

### Synthesis of data

Data from articles were extracted into prespecified tables, including study design, demographics, and results of outcomes and follow-up. The data from the individual articles are presented in the form of a narrative summary. Where possible, the mean and SD were extracted. SDs were estimated from 95% CIs or SEs when not reported. Means and SDs were estimated from medians and range/IQR when only these summary statistics were presented using published formulas.^21^ Furthermore, standardised mean differences (SMD) were calculated for individual studies as this allows results measured on different instruments to be compared and combined (SMDs provided in online supplemental materials). The SMD was estimated as the difference between the scores of the intervention and control group at follow-up divided by the pooled SD. Meta-analysis was used to combine the results of RCTs where possible. An SMD ≥0.2 was considered a small effect, ≥0.5 as a medium-sized effect and ≥0.8 as a large effect.^22^ Heterogeneity was quantified using the I^2^ statistic. All statistical analyses were performed using Stata V.14 (StataCorp).

Grades of Recommendations, Assessment, Development and Evaluation (GRADE) defines high-quality evidence as evidence where further research is very unlikely to change our confidence in the estimate of effect.^23^ Therefore, evidence was rated as high quality if supported by meta-analyses of ≥5 RCTs at low-moderate risk of bias reporting consistent results without important limitations.^24^ GRADE defines moderate quality evidence as evidence where further research is likely to have an important impact on the confidence of the estimate of effect, or may change the estimate.^23^ Evidence was rated as moderate if supported by meta-analyses of ≥3 RCTs or supported by a single RCT with a sample size ≥100 and at low-moderate risk of bias or multiple large observational studies. GRADE defines low quality evidence as evidence where further research is very likely to have an important influence on our confidence in the estimates, or likely to change the estimate.^23^ Evidence was rated as low if supported by multiple RCTs of small sample size or high risk of bias or by single observational studies only. GRADE defines very low quality of evidence as evidence where the estimate of the effect is very uncertain.^23^ Evidence was rated as very low if supported by single small RCTs, or non-randomised trials or single arm intervention studies. Evidence could be downgraded in the event of other potential biases (such as study limitations, inconsistency of results, imprecision, publication bias^24^ or conflicts of interest).

## Results

### Search strategy and study characteristics

**Figure 1:**
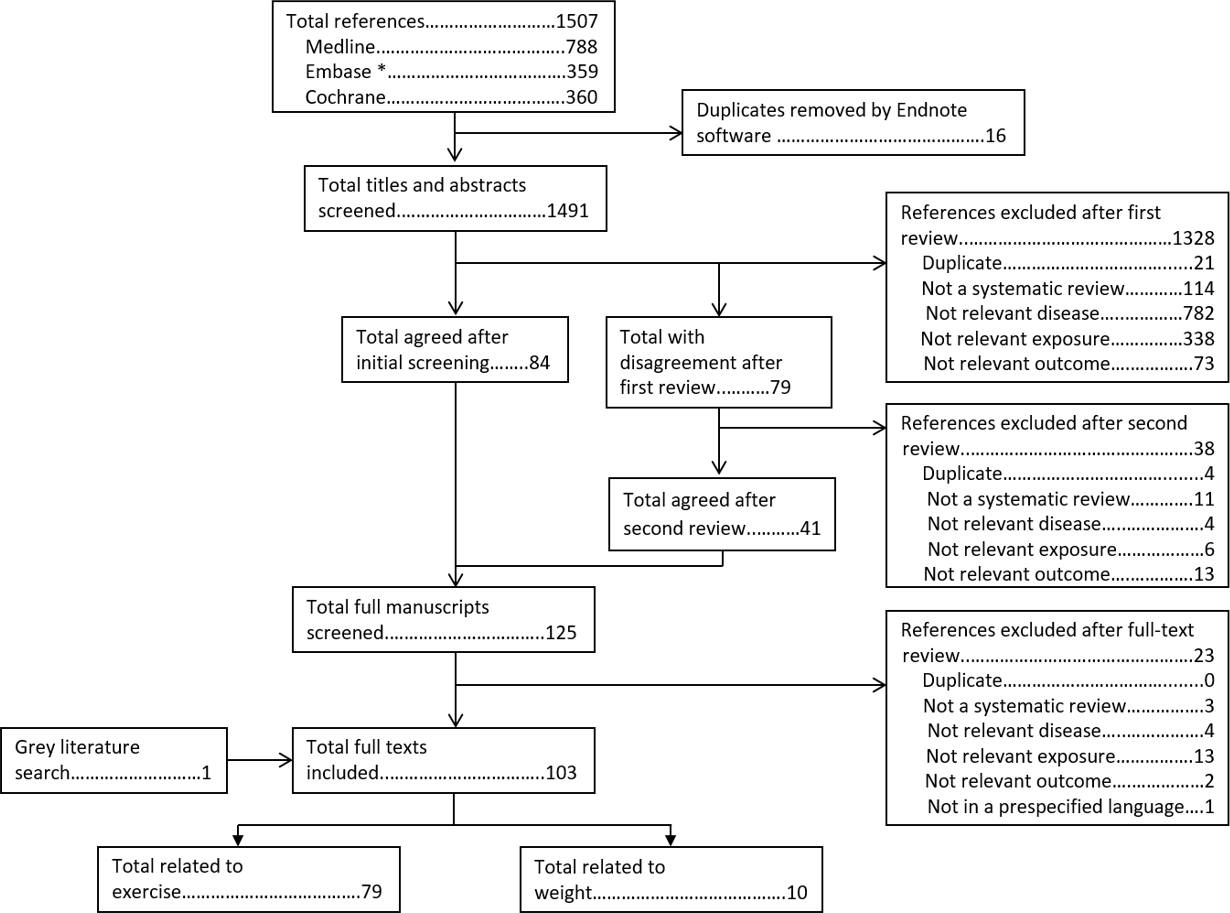
Flow chart of search strategy to identify published systematic reviews and meta-analyses. *EMBASE search excluded journals included in MEDLINE.

The search strategy to identify published systematic reviews identified 1507 abstracts, of which 16 were duplicates and were removed by reference managing software. After title and abstract screening, 1366 abstracts were excluded and the full manuscripts of the remaining 125 were screened. Seventy-nine of these assessed exercise and 10 assessed weight (figure 1).

**Figure 2:**
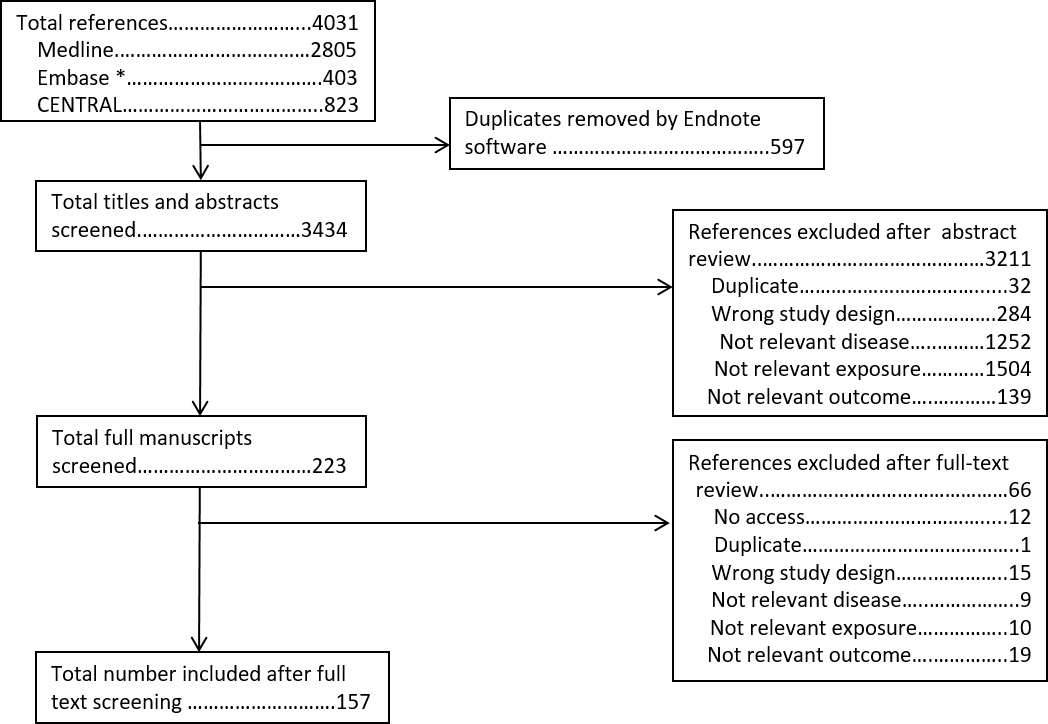
PRISMA flow chart for review of individual studies of exercise. *EMBASE search excluded journals included in MEDLINE. PRISMA, Preferred Reporting Items for Systematic Reviews and Meta-Analyses.

**Figure 3:**
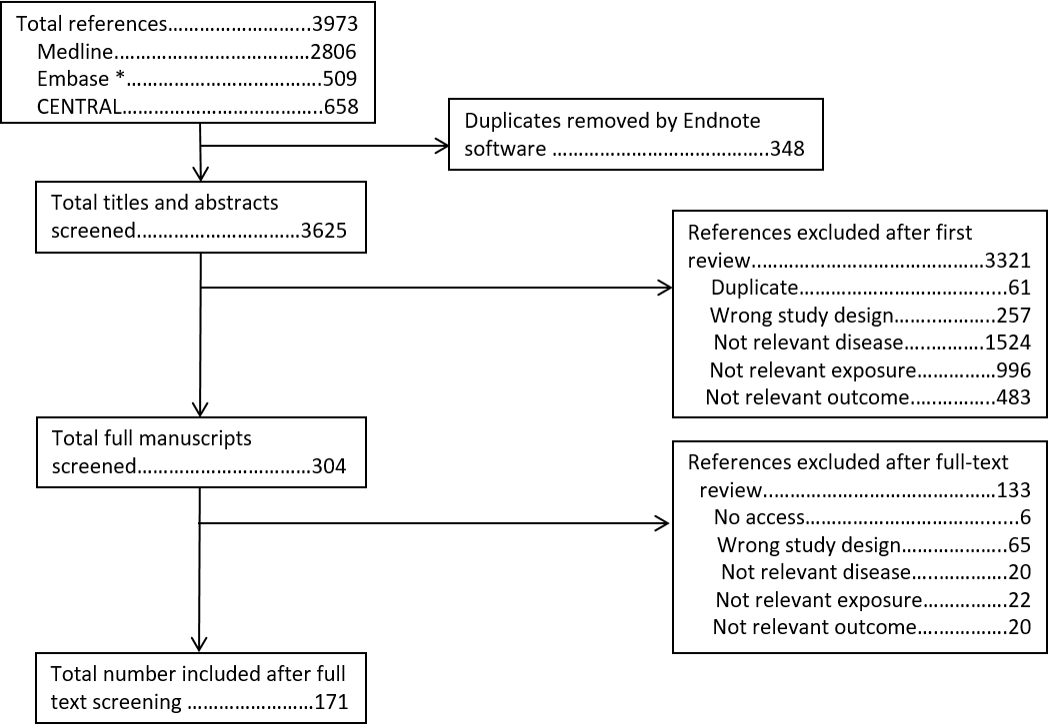
PRISMA flow chart for review of individual studies of weight. *EMBASE search excluded journals included in MEDLINE. PRISMA, Preferred Reporting Items for Systematic Reviews and Meta-Analyses.

The search strategy to identify original articles assessing exercise in RMDs identified 4031 abstracts. After removal of 597 duplicates, 3434 titles and abstracts were screened. Of these 223 full manuscripts were screened, of which 157 are included in this review (figure 2). The search strategy to identify original articles assessing weight identified 3973 abstracts. Once duplicates were removed, 3625 abstracts were screened, followed by screening of 304 full manuscripts. In total, 171 studies on weight were included (figure 3). Results are summarised in tables 1 and 2, with additional information on demographics, specific interventions and control groups (typically usual care or wait-list control), and results of meta-analyses provided in online supplemental material.

**Table 1:** Summary table of results regarding exercise

Level of evidence	Disease	Effect size			
		None	Small	Medium	Large
Very low	OA				
	RA				
	axSpA				
	Other	SLE Muscle strengthening (disease activity)		PsA Muscle strengthening (function, disease activity) SSc Muscle strengthening (function)	
Low	OA				Yoga (function)
	RA	Tai chi (pain, function, disease activity); Yoga (disease activity)		Yoga (function)	
	axSpA		Aquatic (pain, function, disease activity)	Aerobic (pain, function)	
	Other	SLE Aerobic (disease activity) SSc Aerobic + muscle strengthening (pain)	SSc Aerobic + muscle strengthening (pain)		
Moderate	OA				
	RA	Aerobic (disease activity); Aerobic + muscle strengthening (disease activity) Muscle strengthening (disease activity)	Aerobic + muscle strengthening (pain) Aquatic (pain, function) Muscle strengthening (pain, function)		
	axSpA			Muscle strengthening (pain, function, disease activity)	
	Other			SLE Aerobic (fatigue)	
High	OA		Aquatic (pain, function, HR-QoL); Exercise therapy (pain, function); Muscle strengthening (pain, function) Tai chi (stiffness, HR-QoL)	Aerobic (pain, function); Land-based (pain, function) Tai chi (pain, function)	
	RA		Aerobic (pain, function); Aerobic + muscle strengthening (function)		
	axSpA			Aerobic + muscle strengthening (pain, function, disease activity)	
	Other				

axSpA, axial spondyloarthritis; HR-QoL, health-related quality of life; OA, osteoarthritis; PsA, psoriatic arthritis; RA, rheumatoid arthritis; SLE, systemic lupus erythematosus; SSc, systemic sclerosis.

**Table 2:** Summary table of results from observational studies within the weight systematic review

Outcome	RMD						
	OA	RA	SLE	axSpA	PsA	SSc	Gout
Pain	✔✔✔	✔✔	–	✔	✔	–	–
Function	❌	✔✔✔	✔	–	✔	–	❌
Radiographic progression	❌	✔✔†	–	✔	–	–	–
Disease activity	–	✔✔✔	❌	✔✔	✔✔	–	✔ (serum uric acid, gout attacks)
Fatigue	–	✔	❌	✔	–	–	–
Comorbidities	–	✔✔	✔✔	✔	–	–	–
Other	–	–	–	–	✔(Enthesitis) ✔(Psoriasis score)	–	–
Mortality	–	✔✔✔*	–	–	–	✔*	–

Level of evidence of an association between weight and outcome: ✔=very low quality, ✔✔=low quality, ✔✔✔=moderate quality, ✔✔✔✔=high quality, ❌=No evidence of association between weight and outcome from included observational studies, –=No information from included observational studies

This table refers to results where increasing weight is associated with worse scores on outcome measures or higher risk of poor outcomes, other than where noted as follows

*Higher weight associated with lower risk of mortality.

†Higher weight associated with lower radiographic progression in RA.

axSpA, axial spondyloarthritis; OA, osteoarthritis; PsA, psoriatic arthritis; RA, rheumatoid arthritis; RMD, rheumatic and musculoskeletal diseases; SLE, systemic lupus erythematosus; SSc, systemic sclerosis.

### Exercise

#### Osteoarthritis

##### Aerobic exercise

Four meta-analyses of RCTs,^25–28^ four systematic reviews of RCTs^29–32^ and three systematic reviews of observational studies^33–35^ assessed aerobic exercise for OA. Aerobic exercise improved pain^25–27 30–33^ and function^25 29–32 34^ with small-medium effect sizes (pain SMD ranges −0.24 (95% CI −0.50 to 0.02)^30^ to -0.61 (95% CI −0.75 to –0.48)^25^; function −0.30 (95% CI −1.53 to 0.92)^28^ to −0.58 (95% CI −0.75 to –0.40)^25^) as well as one study reporting an association with health-related QoL (HR-QoL).^31^ Another review reported no association with radiographic damage^35^ (online supplemental tables 6 and 7).

##### Aquatic exercise

Five meta-analyses of RCTs^28 36–39^ and three systematic reviews of RCTs^7 32 40^ studied aquatic exercise for OA. Small effects on pain^7 28 32 36 37 39^ (SMDs ranging −0.26 (95% CI −0.41 to –0.11)^39^ to −1.16 (95% CI -3.03 to 0.71)^38^) function^7 32 37–39^ (SMDs ranging −0.22 (95% CI −0.38 to –0.07)^39^ to −0.55 (95% CI −0.94 to –0.16)^38^) and HR-QoL^37–39^ (SMDs ranging −0.21 (95% CI −0.59 to 0.18)^38^ to −0.25 (95% CI −0.49 to –0.01)^37^)were reported (online supplemental tables 8 and 9).

##### Guidelines / recommendations

Two systematic reviews^41 42^ summarised recent guidelines and evidence for OA and exercise, both concluding that exercise is strongly recommended for OA. A third paper^43^ contained a literature review supporting EULAR recommendations for the non-pharmacological management of OA, also recommending that patients with OA have regular, individualised exercise regimens (online supplemental tables 10 and 11).

##### High vs low intensity exercise

One Cochrane review^44^ reported that high intensity exercise was more beneficial than low intensity exercise for pain (meta-MD in the Western Ontario and McMaster Universities Osteoarthritis Index (WOMAC) pain scale −0.84 (95% CI −1.63 to –0.04)) and function (meta-MD WOMAC function −2.65 (95% CI −5.29 to –0.01)) in OA. A systematic review^45^ identified one prospective study^46^ that reported no exercise or low intensity exercise was associated with deterioration in physical function (online supplemental tables 12 and 13).

##### Home exercise

One meta-analysis^47^ reported that home exercise interventions were superior to no exercise in terms of pain (SMD −0.46 (95% CI −0.68 to –0.24)) and function (SMD −0.35 (95% CI −0.56 to –0.15)) in OA, but home exercise programmes were inferior to supervised exercise (online supplemental tables 14 and 15).

##### Land-based exercise

Several articles assessed the efficacy of any type of land-based exercise (as opposed to aquatic) for OA: five meta-analyses of RCTs,^36 48–51^ one systematic review of meta-analyses, reviews and RCTs^7^ and one systematic review of RCTs.^40^ The meta-analyses all reported small effects in favour of land based exercise in terms of pain (SMDs ranging from −0.24 (95% CI −0.42 to –0.06)^48^ to −0.49 (95% CI −0.59 to –0.39)^51^) and function (SMDs ranging from −0.34 (95% CI −0.50 to –0.18)^48^ to -0.52 (95% CI −0.64 to –0.39)^51^), with the systematic reviews in agreement. One meta-analysis also reported an improvement in HR-QoL following exercise (SMD 0.28 (95% CI 0.15 to 0.40))^51^ (online supplemental tables 16 and 17).

##### Multidisciplinary interventions

One meta-analysis^52^ and one systematic review^53^ reported multimodal interventions were superior to exercise only for pain, function and HR-QoL (online supplemental tables 18 and 19).

##### Muscle strengthening exercise

Nine meta-analyses of RCTs,^25–28 54–58^ four systematic reviews of RCTs,^29 59 60^ and one review of meta-analyses, reviews and RCTs^7^ assessed muscle strengthening exercise for OA. Small^26 55 56 58^ to medium^25 27 28^ sized effects on pain^7 59 60^ (SMDs ranging from −0.23 (95% CI −0.42 to –0.04)^55^ to −1.19 (95% CI −1.67 to –0.70)^57^), as well as small^28 56^ to medium^25 57 58^ effects on function were reported^7 59 60^ (SMDs ranging from −0.10 (95% CI −0.33 to 0.13)^55^ to −0.60 (95% CI −0.83 to –0.37)^25^). One meta-analysis reported no effect of muscle strengthening exercise on HR-QoL for hand OA,^56^ whereas a systematic review reported improvements in HR-QoL in knee OA^59^ (online supplemental tables 20 and 21).

##### Physical exercise therapy

In total, five meta-analyses of RCTs,^61–65^ two systematic reviews of RCTs,^60 66^ and one systematic review of reviews and RCTs^67^ assessed exercise therapy for OA. Small effects for pain^60 62–64 66^ (SMDs ranging from −0.20 (95% CI −0.28 to –0.11)^62^ to -0.71 (95% CI −1.60 to 0.19)^64^) and function were reported^60 62 63 65 66^ (SMDs ranging from −0.07 (95% CI −0.28, 0.15)^64^ to −0.33 (95% CI −0.50 to –0.15)^63^), and no effect on anxiety (SMD −0.11 (95% CI −0.26 to 0.05)) and depression (SMD −0.16 (95% CI −0.29 to –0.02)).^62^ One meta-analysis reported a medium-sized effect on HR-QoL in knee OA (SMD 0.70 (95% CI 0.20 to 1.20)),^61^ but another reported no effect in hip OA (SMD −0.06 (95% CI −0.27 to 0.16))^63^ (online supplemental tables 22 and 23).

##### Tai chi

Four meta-analyses of RCTs^26 68–70^ and one systematic review of RCTs^71^ assessed tai chi for OA. Medium-sized effects were reported in terms of pain^26 68–70^ (SMDs ranged from −0.45 (95% CI −0.70 to –0.20)^70^ to −0.77 (95% CI −1.13 to –0.41)^68^) and function^68–70^ (SMDs ranged from −0.61 (95% CI −0.85 to –0.37)^70^ to −0.75 (95% CI −0.98 to –0.52)^68^), and small effects on stiffness^68 70^ and HR-QoL^68 69^ (online supplemental tables 24 and 25).

##### Yoga

One meta-analysis of RCTs,^72^ two systematic reviews of RCTs^71 73^ and one systematic review of RCTs and single arm interventions,^74^ assessed yoga in OA. Three systematic reviews reported benefits of yoga on pain and function.^71 73 74^ The meta-analysis reported a large effect of yoga on function (SMD −1.83 (95% CI −2.09 to –1.57)).^72^ One systematic review reported inconsistent findings in terms of the effect of yoga on HR-QoL^74^ (online supplemental tables 26 and 27).

##### OA summary

The majority of systematic reviews and meta-analyses reported medium-sized effects of physical activity on pain and function in OA, including both aerobic and muscle strengthening physical activity. The quality of the evidence was generally moderate to high (table 1). While the majority of studies on OA included people with knee or hip OA, several studies included people with hand OA.^42 52 55 56 64 73^

#### Rheumatoid arthritis

##### Aerobic exercise

One meta-analysis,^75^ three systematic reviews^76–78^ (which highlighted a 2010 meta-analysis,^79^) eight RCTs,^80–87^ four non-randomised studies,^88–91^ one case–control study^92^ and two prospective cohort studies^93 94^ assessing aerobic exercise in RA were included. Aerobic exercise improved pain^76 77 79 80 82 84 87^ (meta-analysis^79^: SMD 0.31 (95% CI 0.06 to 0.55)); and function^76 79 80 84^ (meta-analysis^79^: SMD 0.24 (95% CI 0.10 to 0.38)) in RA with small effect sizes, although some studies reported statistically non-significant results. One meta-analysis and four included RCTs reported no effect of aerobic exercise on disease activity^79 80 82 83 85^ (meta-analysis^79^: SMD 0.08 (95% CI −0.08 to 0.25)), while another reported a weak correlation.^78^ Furthermore, studies reported a small effect on HR-QoL.^79 81 82 84^ Small effects were also reported on fatigue (meta-analysis^75^: SMD −0.31 (95% CI –0.55 to –0.06)), anxiety, depression and self-efficacy^75 81 82 85^ (online supplemental tables 28–31).

##### Aerobic + muscle strengthening exercise

Twenty-one reports of RCTs,^95–115^ one non-randomised trial,^116^ and seven single arm studies^117–123^ reported on interventions containing both aerobic and muscle strengthening elements in RA. Small effects were reported for pain^96 99 101 106 110–112^ and function,^95–100 102 106 108–111 115^ with wide heterogeneity between studies potentially due to differences in the interventions and follow-up lengths. No effect was observed for disease activity^102 106 108 110^ and depression.^97 101^ Two RCTs reported a small effect on HR-QoL, which was not statistically significant.^98 102^ One recent RCT reported a medium-sized effect on fatigue,^96^ with two older RCTs reporting no effect^101 115^ (online supplemental tables 32 and 33).

##### Aquatic exercise

Two systematic reviews,^77 124^ four RCTs^87 125–127^ and one non-randomised trial^89^ studied aquatic exercises for RA. Small-medium effects on pain^87 124 126^ and function^77 124–127^ were reported (online supplemental tables 34–36).

##### High vs low intensity

Three RCTs^128–130^ and one long-term extension^131^ were included, with none reporting significant differences between high and low intensity exercise on pain or function (online supplemental tables 37 and 38).

##### Home exercise

Two systematic reviews,^77 132^ and five RCTs^101 133–136^ studied home exercise programmes. The reviews concluded that home exercise was beneficial for reducing pain and function.^77 132^ In studies comparing home-based unsupervised physical activity to supervised physical activity, inconsistent results were reported in terms of pain^101 134 135^ and function^134–136^ potentially due to differences in the interventions between studies (online supplemental tables 39–41).

##### Muscle strengthening exercise

One meta-analysis of hand muscle strengthening exercises,^137^ three systematic reviews (one including a 2012 meta-analysis^138^)^77 139 140^ 17 reports of RCTs and long-term extensions,^128 131 141–155^ 1 non-randomised trial^156^ and 1 single-arm study^157^ assessed muscle strengthening exercise in RA. Small effects from muscle strengthening exercise were reported on pain^77 137 138 140 141 143 144 153^ (meta-analysis^138^: MD pain Visual Analogue Scale −4.13 (95% CI −10.97 to 2.71)). Inconsistent results were reported in terms of function, with several studies reporting improvements in function^77 137 138 143 148 149 151^ (meta-analysis^138^: MD Health Assessment Questionnaire −0.22 (95% CI −0.35 to –0.10)), whereas other RCTs reported no benefit,^128 144 147 152 153^ potentially due to differences in the comparison group where some were given low-intensity exercise or exercise advice. No effect was reported on disease activity^128 140 143 145 147^ and HR-QoL^142 144 145 147^ (online supplemental tables 42–44).

##### Tai chi

One systematic review (which identified a 2004 review^158^)^77^ one RCT,^159^ one non-randomised trial^160^ and two single-arm studies^161 162^ assessed tai chi for RA. The reviews and RCT reported no benefit in terms of pain,^159^ function,^77 158 159^ disease activity^77 158^ or depression^159^ (online supplemental tables 45–47).

##### Yoga

One meta-analysis,^72^ one systematic review,^73^ three RCTs^163–165^ and two non-randomised trials^166 167^ studied yoga for RA. The meta-analysis reported a large effect of yoga on pain (but included some patients with OA)^72^ as did one RCT,^165^ whereas two other RCTs reported no effect, and the systematic review graded the evidence as very low.^73 163 164^ The meta-analysis (only including studies of RA)^72^ and one RCT reported medium-sized effects on function,^163^ whereas another RCT reported no effect.^164^ No effect on disease activity was reported by two RCTs.^163 164^ Studies were generally small therefore potentially some findings are due to chance (online supplemental tables 48–50).

##### RA summary

There was moderate-high quality evidence of a small effect of physical activity on pain and function in RA. Physical activity did not affect disease activity (table 1).

#### Systemic lupus erythematosus

##### Aerobic exercise

Two meta-analyses,^168 169^ three systematic reviews,^170–172^ four RCTs^173–176^ and three non-randomised trials^177–179^ assessed aerobic exercise in SLE. One RCT reported lower pain,^173^ whereas another did not^174^ although both studies had low sample sizes. No effect of disease activity was reported.^168 173 174^ A medium-sized effect on fatigue was also reported (MD −0.61 [95% CI −1.19 to –0.02]^168^; MD −0.52 [95% CI −0.92 to –0.13]^169^)^168 169 171 172 175 176^ (online supplemental tables 51–53).

##### Aerobic & muscle strengthening exercise

One meta-analysis,^168^ one systematic review^171^ and three RCTs^173 180 181^ compared aerobic and muscle strengthening exercise, whereas one RCT^182^ and one non-randomised trial^183^ assessed interventions combining both aerobic and muscle strengthening exercises. Aerobic exercise was reported to lead to less fatigue compared with muscle strengthening in two studies,^168 171^ but two other RCTs reported no difference,^180 181^ although again sample sizes were small. No difference was reported in terms of disease activity^168 173 181^ or depression^168 173 180^ (online supplemental tables 54–56).

##### Muscle strengthening exercise

One RCT compared muscle strengthening exercise to control in SLE,^173^ reporting no effect on disease activity, pain and fatigue, but a medium-sized effect on depression in favour of muscle strengthening exercise (online supplemental tables 57 and 58).

##### SLE summary

There is moderate quality evidence of a medium-sized effect of aerobic exercise on fatigue in SLE. There is low-quality evidence that physical activity does not affect disease activity in SLE (table 1).

#### Axial spondyloarthritis

##### Aerobic exercise

Two systematic reviews,^184 185^ four RCTs,^186–189^ one single arm study^190^ and three prospective cohort studies^191–193^ assessed aerobic exercise in axSpA. One RCT investigating exercising with the aid of a video game console reported a medium-sized effect on pain, function and disease activity,^186^ whereas other studies of traditional exercise interventions reported no effect^184 187–189^ (online supplemental tables 59–62).

##### Aerobic + muscle strengthening exercise

Six meta-analyses,^194–199^ 3 systematic reviews,^184 185 200^ 10 RCTs,^201–210^ 3 non-randomised trials,^211–213^ 5 single arm studies^214–218^ and 1 prospective cohort study^219^ assessed aerobic + muscle strengthening exercise in axSpA. Small effects were reported for pain,^185 196 197 204 209 210^ but with some inconsistency^200 207 208^ (SMDs ranging from −0.22 (95% CI −0.49 to 0.06)^197^ to −0.42 (95% CI −0.74 to –0.09),^196^ whereas consistent medium-sized improvements in function^184 194–199 201 203 205 207 210^ (SMDs ranging from −0.44 (95% CI −0.79 to –0.09)^199^ to −0.72 (−1.03 to –0.40),^194^ and disease activity^184 194–199 201 205 206^ (SMDs ranging from −0.47 (95% CI −0.84 to –0.09)^196^ to -0.90 (−1.52 to –0.27)^194^ were reported. Medium-sized effects on fatigue^201 202^ and spinal mobility^195 198 199 205^ were also reported (online supplemental tables 63–66).

##### Aquatic exercise

Three systematic reviews^184 200 220^ and two RCTs^221 222^ assessing aquatic exercise for axSpA were included. Small, inconsistent effects were reported in terms of pain,^184 220–222^ function^184 200 220–222^ and disease activity^184 220–222^ and no effect on spinal mobility^184 221 222^ (online supplemental tables 67–69).

##### Home-based exercise

One meta-analysis,^197^ one systematic review,^185^ eight RCTs,^186 189 203 207 210 223–225^ five non-randomised trials^212 226–229^ and one single arm study^217^ assessed home-based exercise. Compared with control, home exercise had a medium-sized effect on pain,^186 197 210 224^ function,^186 189 197 210 223–225^ disease activity^186 197 223 224^ and HR-QoL.^186 224^ However, several studies found that group-based exercise was more effective than home-based exercise in terms of function^203 207^ and disease activity^203^ (online supplemental tables 70–72).

##### Muscle strengthening exercise

Eight RCTs (and two long-term follow-ups),^223 225 230–237^ three non-randomised trials^226–228^ and three single arm studies^238–240^ assessed muscle strengthening exercise. Medium-sized effects of muscle strengthening exercise were reported in terms of pain,^225 231–233 235^ function^223 225 230 232–235^ and disease activity^223 230 232–235^ (online supplemental tables 73 and 74).

##### AxSpA summary

There is high-quality evidence of a medium-sized effect of aerobic + muscle strengthening exercise in axSpA. Muscle strengthening exercise results in larger and more consistent effects on pain, function and disease activity compared with aerobic exercise (table 1).

#### Psoriatic arthritis

##### Muscle strengthening exercise

One RCT^241^ and one single arm study^242^ assessed muscle strengthening exercise in PsA. The RCT reported better function and disease activity at 12 weeks compared with the control arm^241^ (online supplemental tables 75 and 76).

##### PsA Summary

There is little evidence of the effect of physical activity in PsA.

#### Systemic sclerosis

##### Aerobic exercise

One single arm study^243^ reported improvements in function between baseline and 4 months after the intervention (online supplemental tables 77 and 78).

##### Aerobic + muscle strengthening exercise

One systematic review,^244^ two RCTs^245 246^ and one single arm study^247^ assessed aerobic plus muscle strengthening interventions for SSc, reporting a small effect on function^245 246^ but no effect on pain^246^ (online supplemental tables 79–81).

##### Aquatic exercise

One RCT,^248^ which only reported data on the intervention group, showed improvements in function from baseline to 18 weeks follow-up (online supplemental tables 82 and 83).

##### Muscle strengthening exercise

One RCT,^249^ one non-randomised trial^250^ and three single arm studies^251–253^ assessed muscle strengthening exercise in SSc. The RCT reported a medium-sized effect on function^249^ and the non-randomised study reported better outcomes in terms of pain and function in the exercise arm^250^ (online supplemental tables 84 and 85).

##### SSc summary

There is very-low-quality evidence of a medium effect of physical activity on function in SSc (table 1).

#### Gout

##### Aerobic exercise

One case–control study^254^ reported that performing regular exercise ≥150 min per week was associated with reduced odds of tophus in gout (online supplemental tables 86 and 87).

##### Yoga

One RCT compared yoga to blood-letting in gout,^255^ reporting benefits of yoga in terms of pain and serum uric acid (online supplemental tables 88 and 89).

##### Gout summary

There is little research on the effect of physical activity in gout.

### Body weight and weight reduction

#### Osteoarthritis

Two meta-analyses,^26 33^ 5 systematic reviews,^32 34 35 43 45^ 21 reports describing RCTs and long-term follow-up studies,^256–276^ 2 non-randomised trials^277 278^ and 6 single-arm intervention studies assessing weight-loss interventions,^279–284^ as well as 44 observational studies^285–328^ assessing the association between body weight and outcomes in OA were identified. From studies assessing weight-loss interventions, small effects on pain,^26 32 43 265–267 270 275^ function,^259 265–268 270 272 273^ stiffness^256 266 273^ and walking tests,^260 265 272 276^ but no effect on HR-QoL^260 269^ or patient global assessment^260 269^ were reported. In observational studies, higher body weight was associated with higher pain^33 291 292 299 300 302 307^ but not with worse function^45 287 297 303 319^ or radiographic progression^35 294 301 306^ (online supplemental tables 90–115).

#### Rheumatoid arthritis

Three meta-analyses^329–331^ and 61 observational studies^92 332–391^ assessed the relationship between body weight and outcomes in RA. Higher weight was associated with worse pain,^329 354 361 362 380^ function,^329 336 344 362^ disease activity,^329 330 336 338 339 342 344 347 357 361–363 366 384 389^ and fatigue,^351^ as well as more comorbidities.^331 355 360 365 374 390^ Higher baseline weight was associated with lower risk of death.^335 367 378 379^ However, high rates of weight loss were associated with increased mortality risk.^335 340 356 381^ Higher weight was associated with lower radiographic progression^337 341 346 347 368 371 375 376^ (online supplemental tables 116–132).

#### Systemic lupus erythematosus

Eight prospective studies were identified assessing the relationship between body weight and outcomes in SLE.^392–399^ Higher weight was associated with worse function,^394^ comorbidity,^396 398 399^ employment status^394^ and mental health,^392^ but no associations between weight and disease activity^395^ or fatigue^395^ were reported (online supplemental tables 133–139).

#### Axial spondyloarthritis

Thirteen observational studies were identified assessing the relationship between body weight and outcomes in axSpA.^192 359 400–410^ Higher weight was associated with worse pain,^410^ disease activity,^405 410^ fatigue,^405^ radiographic progression,^401 403^ comorbidity^359 404^ and lower odds of meeting response criteria.^405 409^ One unadjusted analysis reported people with higher weight were more likely to discontinue anti-tumour necrosis factor treatment,^406^ but another study adjusting for confounders reported no association^405^ (online supplemental tables 140–149).

#### Psoriatic arthritis

One meta-analysis,^330^ one RCT,^411^ one single-arm study^412^ and seven observational studies^413–419^ were identified assessing the relationship between weight and outcomes in PsA. Higher weight was associated with worse pain,^415^ function,^415^ disease activity,^330 414–417^ joint counts,^415^ enthesitis occurrence^413^ and psoriasis score.^415^ Interventional studies reported improvements in pain,^411 412^ function,^411 412^ patient global assessment,^411 412^ and reductions in disease activity^411 412^ and C-reactive protein^411 412^ following weight loss (online supplemental tables 150–170).

#### Systemic sclerosis

Two observational studies reported that higher weight was associated with a lower risk of mortality in SSc^420 421^ (online supplemental tables 171 and 172).

#### Gout

One systematic review,^422^ one single-arm study^423^ and five observational studies^424–428^ were identified assessing the relationship between weight and outcomes in gout. One observational study reported no association between weight and function.^428^ Weight loss was associated with improvements in serum uric acid level,^422 423 425^ and less frequent gout attacks.^422–425^ Higher weight was associated with renal failure in one study^426^ but not another^428^ (online supplemental tables 173–180).

## Discussion

These three systematic reviews summarise the current evidence regarding the effects of physical activity and body weight on disease outcomes of people with seven RMDs. The data from over 400 published reviews and original articles suggests there are benefits of exercising for a range of outcomes important to people with RMDs, and also indicate an association between heavier body weight and worse outcomes, although differences were noted across RMDs. There was moderate-to-high-quality evidence that exercise interventions resulted in less pain and better function in RMDs, and exercise was also associated with reductions in disease activity in axSpA. The size of the effect of exercise varied between RMDs. For instance, there were medium-sized effects demonstrated in people with axSpA and only small effects in those with RA. There was moderate-quality evidence that heavier body weight was associated with poorer outcomes, including pain, function and disease activity, with relatively consistent results across the seven RMDs. However, the amount of available evidence varied considerably between RMDs, with the majority of studies focusing on OA, RA, axSpA and to a lesser extent SLE, with only a few studies available focusing on PsA, SSc and gout.

The majority of studies of exercise across the RMDs focused on aerobic and muscle strengthening exercise. Both appeared to improve outcomes in the seven RMDs included in this review, and when combined they seemed to produce stronger effects, although relatively few studies compared aerobic plus muscle strengthening exercise to either form of exercise individually.^173^ A few studies indicated that supervised exercise was marginally more effective than unsupervised home-based exercise,^47 134 203^ although this should not deter individuals who do not wish to attend exercise classes from performing home-based exercise. Overall, the current evidence base indicates that exercising results in better outcomes in people with RMDs, when compared with groups assigned to control groups with no exercise intervention. People with RMDs should be encouraged by their health professionals to start exercising if they currently perform no exercise, or maintain their exercise if they already perform a sufficient amount of exercise. Health professionals should also recognise that there are potentially large barriers to initiating and continuing to exercise for people with RMDs, including time, resources and lack of motivation, as well as disease specific barriers.^429–431^

Heavier body weight was consistently associated with poorer outcomes, including pain, function, disease activity, fatigue and comorbidities. The included studies evaluating weight-loss interventions typically reported improved outcomes in the weight-loss groups compared with controls.^260 265 411^ Additionally, multiple observational studies have reported an association between heavier baseline weight and worse disease outcomes. While these observational studies typically have longer follow-up compared with RCTs, causality between heavier body weight and worse outcomes is difficult to establish, given the complex interrelated associations between heavy weight and other potential sociodemographic and biological factors that could be influencing outcomes.^432 433^ Furthermore, the majority of research focused on OA and RA, with few research studies published on the other RMDs. Despite this, given the deleterious effect of heavier weight on other outcomes (eg, cardiovascular disease^13^ and type 2 diabetes^14^) and the association between weight and poor outcomes in RMDs, people with RMDs should be encouraged to attain and maintain a healthy weight (what consists a healthy weight will be person-specific and should be decided in consultation between the person with an RMD and their health professional).

This review has several limitations. While the large scope and high number of studies included means that a large proportion of the literature has been surveyed, due to the breadth of the research questions, some studies may have been missed. However, the extensive search strategies which were implemented in several databases should mean that these missing studies comprise a low proportion of the total evidence base. Due to the large number of studies, data extraction and risk of bias assessment were not performed in duplicate. Furthermore, due to the nature of the method of identifying studies, some selection bias could be influencing the results, particularly in the body weight systematic review. Many studies will assess multiple factors influencing progression of outcomes, and will often only report the significant associations in their abstracts. As our search strategy identified studies reporting that they studied the association between weight and outcomes in their abstracts, this potentially means we missed studies that assessed the association, found no relationship, and therefore did not report they had assessed weight in their abstract. The majority of studies on exercise were RCTs, with strict inclusion criteria, meaning the samples in these studies were often highly selected, thus reducing generalisability. The majority of studies on weight were longitudinal observational studies. While these studies typically had longer follow-up periods than the few interventional studies included, there is the possibility of differential attrition leading to biased results. For instance, those with higher weight may be more likely to have worse outcomes, which could lead them to leave the study before these outcomes are assessed. Lastly, as the evidence within this review was used to formulate the taskforce’s recommendations, an update was not deemed appropriate. However, this means that some relevant papers have been published in the interim between implementing the search strategy and publishing this paper which are not included.^434 435^

In conclusion, these reviews inform the 2021 EULAR recommendations which advise people with RMDs to perform sufficient amounts of exercise, given the beneficial effects of exercise on numerous outcomes. Furthermore, the 2021 EULAR recommendations also advise people with RMDs to maintain a healthy weight, given the association between heavier weight and poor outcomes, as detailed in this review.

## Data Availability

Data sharing not applicable as no datasets generated and/or analysed for this study.

## References

[1] Smolen JS, Aletaha D, Barton A, et al. Rheumatoid arthritis. Nat Rev Dis Primers 2018;4:18001. 10.1038/nrdp.2018.1 29417936

[2] Martel-Pelletier J, Barr AJ, Cicuttini FM, et al. Osteoarthritis. Nat Rev Dis Primers 2016;2:16072. 10.1038/nrdp.2016.72 27734845

[3] Kaul A, Gordon C, Crow MK, et al. Systemic lupus erythematosus. Nat Rev Dis Primers 2016;2:16039. 10.1038/nrdp.2016.39 27306639

[4] GBD 2019 Diseases and Injuries Collaborators. Global burden of 369 diseases and injuries in 204 countries and territories, 1990-2019: a systematic analysis for the global burden of disease study 2019. Lancet 2020;396:1204–22. 10.1016/S0140-6736(20)30925-9 33069326 PMC7567026

[5] Singh JA, Christensen R, Wells GA, et al. Biologics for rheumatoid arthritis: an overview of Cochrane reviews. Cochrane Database Syst Rev 2009;4:CD007848. 10.1002/14651858.CD007848.pub2 PMC10636593 19821440

[6] Fanouriakis A, Kostopoulou M, Alunno A, et al. 2019 update of the EULAR recommendations for the management of systemic lupus erythematosus. Ann Rheum Dis 2019;78:736–45. 10.1136/annrheumdis-2019-215089 30926722

[7] McAlindon TE, Bannuru RR, Sullivan MC, et al. OARSI guidelines for the non-surgical management of knee osteoarthritis. Osteoarthritis Cartilage 2014;22:363. 10.1016/j.joca.2014.01.003 24462672

[8] Caspersen CJ, Powell KE, Christenson GM. Physical activity, exercise, and physical fitness: definitions and distinctions for health-related research. Public Health Rep 1985;100:126–31. 3920711 PMC1424733

[9] U.S. Department of Health and Human Services. Physical activity guidelines for Americans. 2nd edn. Washington, DC, 2018.

[10] World Health Organisation. Global recommendations on physical activity for health, 2010. 26180873

[11] Rausch Osthoff A-K, Niedermann K, Braun J, et al. 2018 EULAR recommendations for physical activity in people with inflammatory arthritis and osteoarthritis. Ann Rheum Dis 2018;77:1251–60. 10.1136/annrheumdis-2018-213585 29997112

[12] Rausch Osthoff A-K, Juhl CB, Knittle K, et al. Effects of exercise and physical activity promotion: meta-analysis Informing the 2018 EULAR recommendations for physical activity in people with rheumatoid arthritis, spondyloarthritis and hip/knee osteoarthritis. RMD Open 2018;4:e000713. 10.1136/rmdopen-2018-000713 30622734 PMC6307596

[13] , Afshin A, Forouzanfar MH, GBD 2015 Obesity Collaborators, et al. Health effects of overweight and obesity in 195 countries over 25 years. N Engl J Med 2017;377:13–27. 10.1056/NEJMoa1614362 28604169 PMC5477817

[14] Abdullah A, Peeters A, de Courten M, et al. The magnitude of association between overweight and obesity and the risk of diabetes: a meta-analysis of prospective cohort studies. Diabetes Res Clin Pract 2010;89:309–19. 10.1016/j.diabres.2010.04.012 20493574

[15] van der Heijde D, Aletaha D, Carmona L, et al. 2014 update of the EULAR standardised operating procedures for EULAR-endorsed recommendations. Ann Rheum Dis 2015;74:8-13. 10.1136/annrheumdis-2014-206350 25261577 PMC4283681

[16] Moher D, Liberati A, Tetzlaff J, et al. Preferred reporting items for systematic reviews and meta-analyses: the PRISMA statement. PLoS Med 2009;6:e1000097. 10.1371/journal.pmed.1000097 19621072 PMC2707599

[17] Shea BJ, Reeves BC, Wells G, et al. AMSTAR 2: a critical appraisal tool for systematic reviews that include randomised or non-randomised studies of healthcare interventions, or both. BMJ 2017;358:j4008. 10.1136/bmj.j4008 28935701 PMC5833365

[18] Higgins JPT, Altman DG, Gøtzsche PC, et al. The Cochrane collaboration's tool for assessing risk of bias in randomised trials. BMJ 2011;343:d5928. 10.1136/bmj.d5928 22008217 PMC3196245

[19] Soboczenski F, Trikalinos TA, Kuiper J, et al. Machine learning to help researchers evaluate biases in clinical trials: a prospective, randomized user study. BMC Med Inform Decis Mak 2019;19:96. 10.1186/s12911-019-0814-z 31068178 PMC6505190

[20] Hayden JA, van der Windt DA, Cartwright JL, et al. Assessing bias in studies of prognostic factors. Ann Intern Med 2013;158:280-6. 10.7326/0003-4819-158-4-201302190-00009 23420236

[21] Wan X, Wang W, Liu J, et al. Estimating the sample mean and standard deviation from the sample size, median, range and/or interquartile range. BMC Med Res Methodol 2014;14:135. 10.1186/1471-2288-14-135 25524443 PMC4383202

[22] Cohen J. Statistical power analysis for the behavioral sciences. 2nd ed. Hillsdale, NJ: Erlbaum, 1988.

[23] Guyatt GH, Oxman AD, Vist GE, et al. GRADE: an emerging consensus on rating quality of evidence and strength of recommendations. BMJ 2008;336:924–6. 10.1136/bmj.39489.470347.AD 18436948 PMC2335261

[24] Guyatt GH, Oxman AD, Kunz R, et al. What is "quality of evidence" and why is it important to clinicians? BMJ 2008;336:995–8. 10.1136/bmj.39490.551019.BE 18456631 PMC2364804

[25] Juhl C, Christensen R, Roos EM, et al. Impact of exercise type and dose on pain and disability in knee osteoarthritis: a systematic review and meta-regression analysis of randomized controlled trials. Arthritis Rheumatol 2014;66:622–36. 10.1002/art.38290 24574223

[26] Corbett MS, Rice SJC, Madurasinghe V, et al. Acupuncture and other physical treatments for the relief of pain due to osteoarthritis of the knee: network meta-analysis. Osteoarthritis Cartilage 2013;21:1290–8. 10.1016/j.joca.2013.05.007 23973143 PMC3769860

[27] Tanaka R, Ozawa J, Kito N, et al. Efficacy of strengthening or aerobic exercise on pain relief in people with knee osteoarthritis: a systematic review and meta-analysis of randomized controlled trials. Clin Rehabil 2013;27:1059–71. 10.1177/0269215513488898 23828186

[28] Uthman OA, van der Windt DA, Jordan JL, et al. Exercise for lower limb osteoarthritis: systematic review incorporating trial sequential analysis and network meta-analysis. BMJ 2013;347:f5555. 10.1136/bmj.f5555 24055922 PMC3779121

[29] Wijnen A, Bouma SE, Seeber GH, et al. The therapeutic validity and effectiveness of physiotherapeutic exercise following total hip arthroplasty for osteoarthritis: a systematic review. PLoS One 2018;13:e0194517. 10.1371/journal.pone.0194517 29547670 PMC5856403

[30] Alrushud AS, Rushton AB, Kanavaki AM, et al. Effect of physical activity and dietary restriction interventions on weight loss and the musculoskeletal function of overweight and obese older adults with knee osteoarthritis: a systematic review and mixed method data synthesis. BMJ Open 2017;7:e014537. 10.1136/bmjopen-2016-014537 PMC5541637 28600365

[31] Brosseau L, Taki J, Desjardins B, et al. The Ottawa panel clinical practice guidelines for the management of knee osteoarthritis. Part three: aerobic exercise programs. Clin Rehabil 2017;31:612–24. 10.1177/0269215517691085 28183194

[32] Quintrec J-LL, Verlhac B, Cadet C, et al. Physical exercise and weight loss for hip and knee osteoarthritis in very old patients: a systematic review of the literature. Open Rheumatol J 2014;8:89–95. 10.2174/1874312901408010089 25489352 PMC4258698

[33] Pozzobon D, Ferreira PH, Blyth FM, et al. Can obesity and physical activity predict outcomes of elective knee or hip surgery due to osteoarthritis? A meta-analysis of cohort studies. BMJ Open 2018;8:e017689. 10.1136/bmjopen-2017-017689 PMC5855486 29487072

[34] de Rooij M, van der Leeden M, Heymans MW, et al. Prognosis of pain and physical functioning in patients with knee osteoarthritis: a systematic review and meta-analysis. Arthritis Care Res 2016;68:481–92. 10.1002/acr.22693 26316234

[35] Bastick AN, Belo JN, Runhaar J, et al. What are the prognostic factors for radiographic progression of knee osteoarthritis? A meta-analysis. Clin Orthop Relat Res 2015;473:2969–89. 10.1007/s11999-015-4349-z 25995176 PMC4523522

[36] Beumer L, Wong J, Warden SJ, et al. Effects of exercise and manual therapy on pain associated with hip osteoarthritis: a systematic review and meta-analysis. Br J Sports Med 2016;50:458–63. 10.1136/bjsports-2015-095255 26612846

[37] Bartels EM, Juhl CB, Christensen R, et al. Aquatic exercise for the treatment of knee and hip osteoarthritis. Cochrane Database Syst Rev 2016;3:CD005523. 10.1002/14651858.CD005523.pub3 27007113 PMC9942938

[38] Lu M, Su Y, Zhang Y, et al. Effectiveness of aquatic exercise for treatment of knee osteoarthritis: systematic review and meta-analysis. Z Rheumatol 2015;74:543–52. 10.1007/s00393-014-1559-9 25691109

[39] Waller B, Ogonowska-Slodownik A, Vitor M, et al. Effect of therapeutic aquatic exercise on symptoms and function associated with lower limb osteoarthritis: systematic review with meta-analysis. Phys Ther 2014;94:1383–95. 10.2522/ptj.20130417 24903110

[40] Romeo A, Parazza S, Boschi M, et al. Manual therapy and therapeutic exercise in the treatment of osteoarthritis of the hip: a systematic review. Reumatismo 2013;65:63–74. 10.4081/reumatismo.2013.63 23877410

[41] Gay C, Chabaud A, Guilley E, et al. Educating patients about the benefits of physical activity and exercise for their hip and knee osteoarthritis. Systematic literature review. Ann Phys Rehabil Med 2016;59:174–83. 10.1016/j.rehab.2016.02.005 27053003

[42] Nelson AE, Allen KD, Golightly YM, et al. A systematic review of recommendations and guidelines for the management of osteoarthritis: the chronic osteoarthritis management initiative of the U.S. bone and joint initiative. Semin Arthritis Rheum 2014;43:701–12. 10.1016/j.semarthrit.2013.11.012 24387819

[43] Fernandes L, Hagen KB, Bijlsma JWJ, et al. EULAR recommendations for the non-pharmacological core management of hip and knee osteoarthritis. Ann Rheum Dis 2013;72:1125–35. 10.1136/annrheumdis-2012-202745 23595142

[44] Regnaux J-P, Lefevre-Colau M-M, Trinquart L, et al. High-intensity versus low-intensity physical activity or exercise in people with hip or knee osteoarthritis. Cochrane Database Syst Rev 2015:CD010203. 10.1002/14651858.CD010203.pub2 26513223 PMC9270723

[45] de Rooij M, van der Leeden M, Heymans MW, et al. Course and predictors of pain and physical functioning in patients with hip osteoarthritis: systematic review and meta-analysis. J Rehabil Med 2016;48:245–52. 10.2340/16501977-2057 26871564

[46] Juhakoski R, Malmivaara A, Lakka TA, et al. Determinants of pain and functioning in hip osteoarthritis - a two-year prospective study. Clin Rehabil 2013;27:281–7. 10.1177/0269215512453060 22843354

[47] Anwer S, Alghadir A, Brismée J-M. Effect of home exercise program in patients with knee osteoarthritis: a systematic review and meta-analysis. J Geriatr Phys Ther 2016;39:38–48. 10.1519/JPT.0000000000000045 25695471

[48] Moseng T, Dagfinrud H, Smedslund G, et al. The importance of dose in land-based supervised exercise for people with hip osteoarthritis. A systematic review and meta-analysis. Osteoarthritis Cartilage 2017;25:1563–76. 10.1016/j.joca.2017.06.004 28648741

[49] Fernandopulle S, Perry M, Manlapaz D, et al. Effect of Land-Based generic physical activity interventions on pain, physical function, and physical performance in hip and knee osteoarthritis: a systematic review and meta-analysis. Am J Phys Med Rehabil 2017;96:773–92. 10.1097/PHM.0000000000000736 28323761

[50] Henriksen M, Hansen JB, Klokker L, et al. Comparable effects of exercise and analgesics for pain secondary to knee osteoarthritis: a meta-analysis of trials included in Cochrane systematic reviews. J Comp Eff Res 2016;5:417–31. 10.2217/cer-2016-0007 27346368

[51] Fransen M, McConnell S, Harmer AR, et al. Exercise for osteoarthritis of the knee. Cochrane Database Syst Rev 2015;1:CD004376. 10.1002/14651858.CD004376.pub3 25569281 PMC10094004

[52] Aebischer B, Elsig S, Taeymans J. Effectiveness of physical and occupational therapy on pain, function and quality of life in patients with trapeziometacarpal osteoarthritis - A systematic review and meta-analysis. Hand Ther 2016;21:5–15. 10.1177/1758998315614037 27110291 PMC4778382

[53] Finney A, Healey E, Jordan JL, et al. Multidisciplinary approaches to managing osteoarthritis in multiple joint sites: a systematic review. BMC Musculoskelet Disord 2016;17:266. 10.1186/s12891-016-1125-5 27391036 PMC4938970

[54] Bartholdy C, Juhl C, Christensen R, et al. The role of muscle strengthening in exercise therapy for knee osteoarthritis: a systematic review and meta-regression analysis of randomized trials. Semin Arthritis Rheum 2017;47:9–21. 10.1016/j.semarthrit.2017.03.007 28438380

[55] Magni NE, McNair PJ, Rice DA. The effects of resistance training on muscle strength, joint pain, and hand function in individuals with hand osteoarthritis: a systematic review and meta-analysis. Arthritis Res Ther 2017;19:131. 10.1186/s13075-017-1348-3 28610637 PMC5470180

[56] Østerås N, Kjeken I, Smedslund G, et al. Exercise for hand osteoarthritis. Cochrane Database Syst Rev 2017;1:CD010388. 10.1002/14651858.CD010388.pub2 28141914 PMC6464796

[57] Coudeyre E, Jegu AG, Giustanini M, et al. Isokinetic muscle strengthening for knee osteoarthritis: a systematic review of randomized controlled trials with meta-analysis. Ann Phys Rehabil Med 2016;59:207–15. 10.1016/j.rehab.2016.01.013 27079585

[58] Li Y, Su Y, Chen S, et al. The effects of resistance exercise in patients with knee osteoarthritis: a systematic review and meta-analysis. Clin Rehabil 2016;30:947–59. 10.1177/0269215515610039 26471972

[59] Brosseau L, Taki J, Desjardins B, et al. The Ottawa panel clinical practice guidelines for the management of knee osteoarthritis. Part two: strengthening exercise programs. Clin Rehabil 2017;31:596–611. 10.1177/0269215517691084 28183213

[60] Brosseau L, Wells GA, Pugh AG, et al. Ottawa panel evidence-based clinical practice guidelines for therapeutic exercise in the management of hip osteoarthritis. Clin Rehabil 2016;30:935–46. 10.1177/0269215515606198 26400851

[61] Briani RV, Ferreira AS, Pazzinatto MF, et al. What interventions can improve quality of life or psychosocial factors of individuals with knee osteoarthritis? A systematic review with meta-analysis of primary outcomes from randomised controlled trials. Br J Sports Med 2018;52:1031–8. 10.1136/bjsports-2017-098099 29549150

[62] Hurley M, Dickson K, Hallett R, et al. Exercise interventions and patient beliefs for people with hip, knee or hip and knee osteoarthritis: a mixed methods review. Cochrane Database Syst Rev 2018;4:CD010842. 10.1002/14651858.CD010842.pub2 29664187 PMC6494515

[63] Sampath KK, Mani R, Miyamori T, et al. The effects of manual therapy or exercise therapy or both in people with hip osteoarthritis: a systematic review and meta-analysis. Clin Rehabil 2016;30:1141–55. 10.1177/0269215515622670 26701903

[64] Bertozzi L, Valdes K, Vanti C, et al. Investigation of the effect of conservative interventions in thumb carpometacarpal osteoarthritis: systematic review and meta-analysis. Disabil Rehabil 2015;37:2025–43. 10.3109/09638288.2014.996299 25559974

[65] Desveaux L, Beauchamp M, Goldstein R, et al. Community-based exercise programs as a strategy to optimize function in chronic disease: a systematic review. Med Care 2014;52:216–26. 10.1097/MLR.0000000000000065 24374411

[66] Ferreira GE, Robinson CC, Wiebusch M, et al. The effect of exercise therapy on knee adduction moment in individuals with knee osteoarthritis: a systematic review. Clin Biomech 2015;30:521–7. 10.1016/j.clinbiomech.2015.03.028 25896448

[67] Fehring TK, Fehring K, Odum SM, et al. Physical therapy mandates by Medicare administrative contractors: effective or wasteful? J Arthroplasty 2013;28:1459–62. 10.1016/j.arth.2013.05.027 23796555

[68] Zhang Y, Huang L, Su Y, et al. The effects of traditional Chinese exercise in treating knee osteoarthritis: a systematic review and meta-analysis. PLoS One 2017;12:e0170237. 10.1371/journal.pone.0170237 28121996 PMC5266306

[69] Chen Y-W, Hunt MA, Campbell KL, et al. The effect of Tai Chi on four chronic conditions-cancer, osteoarthritis, heart failure and chronic obstructive pulmonary disease: a systematic review and meta-analyses. Br J Sports Med 2016;50:397–407. 10.1136/bjsports-2014-094388 26383108

[70] Yan J-H, Gu W-J, Sun J, et al. Efficacy of Tai Chi on pain, stiffness and function in patients with osteoarthritis: a meta-analysis. PLoS One 2013;8:e61672. 10.1371/journal.pone.0061672 23620778 PMC3631149

[71] Brosseau L, Taki J, Desjardins B, et al. The Ottawa panel clinical practice guidelines for the management of knee osteoarthritis. Part one: introduction, and mind-body exercise programs. Clin Rehabil 2017;31:582–95. 10.1177/0269215517691083 28183188

[72] Wang Y, Lu S, Wang R, et al. Integrative effect of yoga practice in patients with knee arthritis: a PRISMA-compliant meta-analysis. Medicine 2018;97:e11742. 10.1097/MD.0000000000011742 30075589 PMC6081169

[73] Cramer H, Lauche R, Langhorst J, et al. Yoga for rheumatic diseases: a systematic review. Rheumatology 2013;52:2025–30. 10.1093/rheumatology/ket264 23934220

[74] Kan L, Zhang J, Yang Y, et al. The effects of yoga on pain, mobility, and quality of life in patients with knee osteoarthritis: a systematic review. Evid Based Complement Alternat Med 2016;2016:6016532. 10.1155/2016/6016532 27777597 PMC5061981

[75] Rongen-van Dartel SAA, Repping-Wuts H, Flendrie M, et al. Effect of aerobic exercise training on fatigue in rheumatoid arthritis: a meta-analysis. Arthritis Care Res 2015;67:1054–62. 10.1002/acr.22561 25624016

[76] Hernández-Hernández MV, Díaz-González F. Role of physical activity in the management and assessment of rheumatoid arthritis patients. Reumatol Clin 2017;13:214–20. 10.1016/j.reuma.2016.04.003 27263964

[77] Siegel P, Tencza M, Apodaca B, et al. Effectiveness of occupational therapy interventions for adults with rheumatoid arthritis: a systematic review. Am J Occup Ther 2017;71:7101180050. 10.5014/ajot.2017.023176 28027042

[78] Larkin L, Kennedy N. Correlates of physical activity in adults with rheumatoid arthritis: a systematic review. J Phys Act Health 2014;11:1248–61. 10.1123/jpah.2012-0194 23963816

[79] Baillet A, Zeboulon N, Gossec L, et al. Efficacy of cardiorespiratory aerobic exercise in rheumatoid arthritis: meta-analysis of randomized controlled trials. Arthritis Care Res 2010;62:984–92. 10.1002/acr.20146 20589690

[80] Katz P, Margaretten M, Gregorich S, et al. Physical activity to reduce fatigue in rheumatoid arthritis: a randomized controlled trial. Arthritis Care Res 2018;70:1–10. 10.1002/acr.23230 28378441

[81] Baxter SV, Hale LA, Stebbings S, et al. Walking is a feasible physical activity for people with rheumatoid arthritis: a feasibility randomized controlled trial. Musculoskeletal Care 2016;14:47–56. 10.1002/msc.1112 26228264

[82] Feldthusen C, Dean E, Forsblad-d'Elia H, et al. Effects of Person-Centered physical therapy on Fatigue-Related variables in persons with rheumatoid arthritis: a randomized controlled trial. Arch Phys Med Rehabil 2016;97:26–36. 10.1016/j.apmr.2015.09.022 26482574

[83] Sjöquist ES, Brodin N, Lampa J, et al. Physical activity coaching of patients with rheumatoid arthritis in everyday practice: a long-term follow-up. Musculoskeletal Care 2011;9:75–85. 10.1002/msc.199 21618399

[84] Brodin N, Eurenius E, Jensen I, et al. Coaching patients with early rheumatoid arthritis to healthy physical activity: a multicenter, randomized, controlled study. Arthritis Rheum 2008;59:325–31. 10.1002/art.23327 18311770

[85] Li LC, Davis AM, Lineker SC, et al. Effectiveness of the primary therapist model for rheumatoid arthritis rehabilitation: a randomized controlled trial. Arthritis Rheum 2006;55:42–52. 10.1002/art.21692 16463410

[86] Melikoglu MA, Karatay S, Senel K, et al. Association between dynamic exercise therapy and IGF-1 and IGFBP-3 concentrations in the patients with rheumatoid arthritis. Rheumatol Int 2006;26:309–13. 10.1007/s00296-005-0605-y 15933856

[87] Hansen TM, Hansen G, Langgaard AM, et al. Longterm physical training in rheumatoid arthritis. A randomized trial with different training programs and blinded observers. Scand J Rheumatol 1993;22:107–12. 10.3109/03009749309099253 8316770

[88] Nordström DC, Konttinen YT, Solovieva S, et al. In- and out-patient rehabilitation in rheumatoid arthritis. A controlled, open, longitudinal, cost-effectiveness study. Scand J Rheumatol 1996;25:200–6. 10.3109/03009749609069988 8792796

[89] Minor MA, Hewett JE. Physical fitness and work capacity in women with rheumatoid arthritis. Arthritis Care Res 1995;8:146–54. 10.1002/art.1790080306 7654798

[90] Noreau L, Martineau H, Roy L, et al. Effects of a modified dance-based exercise on cardiorespiratory fitness, psychological state and health status of persons with rheumatoid arthritis. Am J Phys Med Rehabil 1995;74:19–27. 10.1097/00002060-199501000-00004 7873109

[91] Ekblom B, Lövgren O, Alderin M, et al. Effect of short-term physical training on patients with rheumatoid arthritis. a six-month follow-up study. Scand J Rheumatol 1975;4:87–91. 10.3109/03009747509095620 1135613

[92] Nadareishvili Z, Michaud K, Hallenbeck JM, et al. Cardiovascular, rheumatologic, and pharmacologic predictors of stroke in patients with rheumatoid arthritis: a nested, case-control study. Arthritis Rheum 2008;59:1090–6. 10.1002/art.23935 18668583 PMC2778069

[93] Wolfe F, Michaud K. The risk of myocardial infarction and pharmacologic and nonpharmacologic myocardial infarction predictors in rheumatoid arthritis: a cohort and nested case-control analysis. Arthritis Rheum 2008;58:2612–21. 10.1002/art.23811 18759273

[94] Stenström CH. Radiologically observed progression of joint destruction and its relationship with demographic factors, disease severity, and exercise frequency in patients with rheumatoid arthritis. Phys Ther 1994;74:32–9. 10.1093/ptj/74.1.32 8265726

[95] Lange E, Kucharski D, Svedlund S, et al. Effects of aerobic and resistance exercise in older adults with rheumatoid arthritis: a randomized controlled trial. Arthritis Care Res 2019;71:61–70. 10.1002/acr.23589 PMC6590333 29696812

[96] Durcan L, Wilson F, Cunnane G. The effect of exercise on sleep and fatigue in rheumatoid arthritis: a randomized controlled study. J Rheumatol 2014;41:1966–73. 10.3899/jrheum.131282 25128510

[97] Breedland I, van Scheppingen C, Leijsma M, et al. Effects of a group-based exercise and educational program on physical performance and disease self-management in rheumatoid arthritis: a randomized controlled study. Phys Ther 2011;91:879–93. 10.2522/ptj.20090010 21474637

[98] Hurkmans EJ, van den Berg MH, Ronday KH, et al. Maintenance of physical activity after Internet-based physical activity interventions in patients with rheumatoid arthritis. Rheumatology 2010;49:167–72. 10.1093/rheumatology/kep285 19995857

[99] Flint-Wagner HG, Lisse J, Lohman TG, et al. Assessment of a sixteen-week training program on strength, pain, and function in rheumatoid arthritis patients. J Clin Rheumatol 2009;15:165–71. 10.1097/RHU.0b013e318190f95f 19279507

[100] Bulthuis Y, Drossaers-Bakker KW, Taal E, et al. Arthritis patients show long-term benefits from 3 weeks intensive exercise training directly following hospital discharge. Rheumatology 2007;46:1712–7. 10.1093/rheumatology/kem236 17956917

[101] Neuberger GB, Aaronson LS, Gajewski B, et al. Predictors of exercise and effects of exercise on symptoms, function, aerobic fitness, and disease outcomes of rheumatoid arthritis. Arthritis Rheum 2007;57:943–52. 10.1002/art.22903 17665488

[102] van den Berg MH, Ronday HK, Peeters AJ, et al. Using internet technology to deliver a home-based physical activity intervention for patients with rheumatoid arthritis: a randomized controlled trial. Arthritis Rheum 2006;55:935–45. 10.1002/art.22339 17139640

[103] Munneke M, de Jong Z, Zwinderman AH, et al. Effect of a high-intensity weight-bearing exercise program on radiologic damage progression of the large joints in subgroups of patients with rheumatoid arthritis. Arthritis Rheum 2005;53:410–7. 10.1002/art.21165 15934121

[104] de Jong Z, Munneke M, Lems WF, et al. Slowing of bone loss in patients with rheumatoid arthritis by long-term high-intensity exercise: results of a randomized, controlled trial. Arthritis Rheum 2004;50:1066–76. 10.1002/art.20117 15077288

[105] de Jong Z, Munneke M, Zwinderman AH, et al. Long term high intensity exercise and damage of small joints in rheumatoid arthritis. Ann Rheum Dis 2004;63:1399–405. 10.1136/ard.2003.015826 15479889 PMC1754798

[106] Häkkinen A, Sokka T, Hannonen P. A home-based two-year strength training period in early rheumatoid arthritis led to good long-term compliance: a five-year followup. Arthritis Rheum 2004;51:56–62. 10.1002/art.20088 14872456

[107] Häkkinen A, Sokka T, Kautiainen H, et al. Sustained maintenance of exercise induced muscle strength gains and normal bone mineral density in patients with early rheumatoid arthritis: a 5 year follow up. Ann Rheum Dis 2004;63:910–6. 10.1136/ard.2003.013003 15249317 PMC1755099

[108] de Jong Z, Munneke M, Zwinderman AH, et al. Is a long-term high-intensity exercise program effective and safe in patients with rheumatoid arthritis? Results of a randomized controlled trial. Arthritis Rheum 2003;48:2415–24. 10.1002/art.11216 13130460

[109] Westby MD, Wade JP, Rangno KK, et al. A randomized controlled trial to evaluate the effectiveness of an exercise program in women with rheumatoid arthritis taking low dose prednisone. J Rheumatol 2000;27:1674–80. 10914850

[110] Häkkinen A, Sokka T, Kotaniemi A, et al. Dynamic strength training in patients with early rheumatoid arthritis increases muscle strength but not bone mineral density. J Rheumatol 1999;26:1257–63. 10381039

[111] Lyngberg KK, Harreby M, Bentzen H, et al. Elderly rheumatoid arthritis patients on steroid treatment tolerate physical training without an increase in disease activity. Arch Phys Med Rehabil 1994;75:1189–95. 10.1016/0003-9993(94)90003-5 7979927

[112] Ekdahl C, Andersson SI, Moritz U, et al. Dynamic versus static training in patients with rheumatoid arthritis. Scand J Rheumatol 1990;19:17–26. 10.3109/03009749009092618 2309102

[113] Lyngberg K, Danneskiold-Samsøe B, Halskov O. The effect of physical training on patients with rheumatoid arthritis: changes in disease activity, muscle strength and aerobic capacity. A clinically controlled minimized cross-over study. Clin Exp Rheumatol 1988;6:253–60. 3052971

[114] Nordemar R, Ekblom B, Zachrisson L, et al. Physical training in rheumatoid arthritis: a controlled long-term study. I. Scand J Rheumatol 1981;10:17–23. 7013057

[115] Nordemar R. Physical training in rheumatoid arthritis: a controlled long-term study. II. Functional capacity and general attitudes. Scand J Rheumatol 1981;10:25–30. 10.1080/03009748109095266 7013058

[116] Stavropoulos-Kalinoglou A, Metsios GS, Veldhuijzen van Zanten JJJCS, et al. Individualised aerobic and resistance exercise training improves cardiorespiratory fitness and reduces cardiovascular risk in patients with rheumatoid arthritis. Ann Rheum Dis 2013;72:1819–25. 10.1136/annrheumdis-2012-202075 23155222

[117] Löfgren M, Opava CH, Demmelmaier I, et al. Long-term, health-enhancing physical activity is associated with reduction of pain but not pain sensitivity or improved exercise-induced hypoalgesia in persons with rheumatoid arthritis. Arthritis Res Ther 2018;20:262. 10.1186/s13075-018-1758-x 30477552 PMC6260682

[118] Nordgren B, Fridén C, Demmelmaier I, et al. An outsourced health-enhancing physical activity programme for people with rheumatoid arthritis: exploration of adherence and response. Rheumatology 2015;54:1065–73. 10.1093/rheumatology/keu444 25433043 PMC4481374

[119] Di Gioia L, Zincarelli C, Di Minno MND, et al. Effectiveness of a rehabilitative programme in improving fatigue and function in rheumatoid arthritis patients treated with biologics: a pilot study. Clin Exp Rheumatol 2013;31:285–8. 23406843

[120] Strasser B, Leeb G, Strehblow C, et al. The effects of strength and endurance training in patients with rheumatoid arthritis. Clin Rheumatol 2011;30:623–32. 10.1007/s10067-010-1584-2 20931346

[121] van der Giesen FJ, van Lankveld W, Hopman-Rock M, et al. Exploring the public health impact of an intensive exercise program for patients with rheumatoid arthritis: a dissemination and implementation study. Arthritis Care Res 2010;62:865–72. 10.1002/acr.20138 20535798

[122] de Jong Z, Munneke M, Kroon HM, et al. Long-term follow-up of a high-intensity exercise program in patients with rheumatoid arthritis. Clin Rheumatol 2009;28:663–71. 10.1007/s10067-009-1125-z 19247575

[123] Neuberger GB, Press AN, Lindsley HB, et al. Effects of exercise on fatigue, aerobic fitness, and disease activity measures in persons with rheumatoid arthritis. Res Nurs Health 1997;20:195–204. 10.1002/(SICI)1098-240X(199706)20:3<195::AID-NUR3>3.0.CO;2-D 9179174

[124] Al-Qubaeissy KY, Fatoye FA, Goodwin PC, et al. The effectiveness of hydrotherapy in the management of rheumatoid arthritis: a systematic review. Musculoskeletal Care 2013;11:3–18. 10.1002/msc.1028 22806987

[125] Siqueira US, Orsini Valente LG, de Mello MT, et al. Effectiveness of aquatic exercises in women with rheumatoid arthritis: a randomized, controlled, 16-Week Intervention-The HydRA trial. Am J Phys Med Rehabil 2017;96:167–75. 10.1097/PHM.0000000000000564 27386811

[126] Eversden L, Maggs F, Nightingale P, et al. A pragmatic randomised controlled trial of hydrotherapy and land exercises on overall well being and quality of life in rheumatoid arthritis. BMC Musculoskelet Disord 2007;8:23. 10.1186/1471-2474-8-23 17331241 PMC1821024

[127] Bilberg A, Ahlmén M, Mannerkorpi K. Moderately intensive exercise in a temperate pool for patients with rheumatoid arthritis: a randomized controlled study. Rheumatology 2005;44:502–8. 10.1093/rheumatology/keh528 15728422

[128] Lemmey AB, Marcora SM, Chester K, et al. Effects of high-intensity resistance training in patients with rheumatoid arthritis: a randomized controlled trial. Arthritis Rheum 2009;61:1726–34. 10.1002/art.24891 19950325

[129] van den Ende CH, Breedveld FC, le Cessie S, et al. Effect of intensive exercise on patients with active rheumatoid arthritis: a randomised clinical trial. Ann Rheum Dis 2000;59:615–21. 10.1136/ard.59.8.615 10913058 PMC1753212

[130] van den Ende CH, Hazes JM, le Cessie S, et al. Comparison of high and low intensity training in well controlled rheumatoid arthritis. Results of a randomised clinical trial. Ann Rheum Dis 1996;55:798–805. 10.1136/ard.55.11.798 8976635 PMC1010314

[131] Lemmey AB, Williams SL, Marcora SM, et al. Are the benefits of a high-intensity progressive resistance training program sustained in rheumatoid arthritis patients? A 3-year followup study. Arthritis Care Res 2012;64:71–5. 10.1002/acr.20523 21671413

[132] Hammond A, Prior Y. The effectiveness of home hand exercise programmes in rheumatoid arthritis: a systematic review. Br Med Bull 2016;119:49–62. 10.1093/bmb/ldw024 27365455

[133] Zernicke J, Kedor C, Müller A, et al. A prospective pilot study to evaluate an animated home-based physical exercise program as a treatment option for patients with rheumatoid arthritis. BMC Musculoskelet Disord 2016;17:351. 10.1186/s12891-016-1208-3 27538847 PMC4990861

[134] Seneca T, Hauge EM, Maribo T. Comparable effect of partly supervised and self-administered exercise programme in early rheumatoid arthritis--a randomised, controlled trial. Dan Med J 2015;62:A5127. 26239594

[135] Hsieh L-F, Chen S-C, Chuang C-C, et al. Supervised aerobic exercise is more effective than home aerobic exercise in female Chinese patients with rheumatoid arthritis. J Rehabil Med 2009;41:332–7. 10.2340/16501977-0330 19363565

[136] Stenström CH. Home exercise in rheumatoid arthritis functional class II: goal setting versus pain attention. J Rheumatol 1994;21:627–34. 8035384

[137] Williams MA, Srikesavan C, Heine PJ, et al. Exercise for rheumatoid arthritis of the hand. Cochrane Database Syst Rev 2018;7:CD003832. 10.1002/14651858.CD003832.pub3 30063798 PMC6513509

[138] Baillet A, Vaillant M, Guinot M, et al. Efficacy of resistance exercises in rheumatoid arthritis: meta-analysis of randomized controlled trials. Rheumatology 2012;51:519–27. 10.1093/rheumatology/ker330 22120463

[139] Daien CI, Hua C, Combe B, et al. Non-pharmacological and pharmacological interventions in patients with early arthritis: a systematic literature review informing the 2016 update of EULAR recommendations for the management of early arthritis. RMD Open 2017;3:e000404. 10.1136/rmdopen-2016-000404 28151539 PMC5237765

[140] Bergstra SA, Murgia A, Te Velde AF, et al. A systematic review into the effectiveness of hand exercise therapy in the treatment of rheumatoid arthritis. Clin Rheumatol 2014;33:1539–48. 10.1007/s10067-014-2691-2 24952308

[141] Lo C-N, Xia G, Leung BP. The effect of nerve mobilization exercise in patients with rheumatoid arthritis: a pilot study. Reumatismo 2017;69:111–8. 10.4081/reumatismo.2017.918 28933133

[142] Williamson E, McConkey C, Heine P, et al. Hand exercises for patients with rheumatoid arthritis: an extended follow-up of the SARAH randomised controlled trial. BMJ Open 2017;7:e013121. 10.1136/bmjopen-2016-013121 PMC5775458 28404610

[143] Lourenzi FM, Jones A, Pereira DF, et al. Effectiveness of an overall progressive resistance strength program for improving the functional capacity of patients with rheumatoid arthritis: a randomized controlled trial. Clin Rehabil 2017;31:1482–91. 10.1177/0269215517698732 29050507

[144] Lamb SE, Williamson EM, Heine PJ, et al. Exercises to improve function of the rheumatoid hand (SARAH): a randomised controlled trial. Lancet 2015;385:421–9. 10.1016/S0140-6736(14)60998-3 25308290

[145] Manning VL, Hurley MV, Scott DL, et al. Education, self-management, and upper extremity exercise training in people with rheumatoid arthritis: a randomized controlled trial. Arthritis Care Res 2014;66:217–27. 10.1002/acr.22102 23925924

[146] Dogu B, Sirzai H, Yilmaz F, et al. Effects of isotonic and isometric hand exercises on pain, hand functions, dexterity and quality of life in women with rheumatoid arthritis. Rheumatol Int 2013;33:2625–30. 10.1007/s00296-013-2787-z 23739921

[147] Baillet A, Payraud E, Niderprim V-A, et al. A dynamic exercise programme to improve patients' disability in rheumatoid arthritis: a prospective randomized controlled trial. Rheumatology 2009;48:410–5. 10.1093/rheumatology/ken511 19211654

[148] Masiero S, Boniolo A, Wassermann L, et al. Effects of an educational-behavioral joint protection program on people with moderate to severe rheumatoid arthritis: a randomized controlled trial. Clin Rheumatol 2007;26:2043–50. 10.1007/s10067-007-0615-0 17404783

[149] O'Brien AV, Jones P, Mullis R, et al. Conservative hand therapy treatments in rheumatoid arthritis--a randomized controlled trial. Rheumatology 2006;45:577–83. 10.1093/rheumatology/kei215 16319099

[150] Buljina AI, Taljanovic MS, Avdic DM, et al. Physical and exercise therapy for treatment of the rheumatoid hand. Arthritis Rheum 2001;45:392–7. 10.1002/1529-0131(200108)45:4<392::AID-ART353>3.0.CO;2-2 11501728

[151] Scholten C, Brodowicz T, Graninger W, et al. Persistent functional and social benefit 5 years after a multidisciplinary arthritis training program. Arch Phys Med Rehabil 1999;80:1282–7. 10.1016/S0003-9993(99)90030-8 10527088

[152] Boström C, Harms-Ringdahl K, Karreskog H, et al. Effects of static and dynamic shoulder rotator exercises in women with rheumatoid arthritis: a randomised comparison of impairment, disability, handicap, and health. Scand J Rheumatol 1998;27:281–90. 10.1080/030097498442398 9751469

[153] Komatireddy GR, Leitch RW, Cella K, et al. Efficacy of low load resistive muscle training in patients with rheumatoid arthritis functional class II and III. J Rheumatol 1997;24:1531–9. 9263147

[154] Hoenig H, Groff G, Pratt K, et al. A randomized controlled trial of home exercise on the rheumatoid hand. J Rheumatol 1993;20:785–9. 8336303

[155] Dellhag B, Wollersjö I, Bjelle A. Effect of active hand exercise and wax bath treatment in rheumatoid arthritis patients. Arthritis Care Res 1992;5:87–92. 10.1002/art.1790050207 1390969

[156] Marcora SM, Lemmey AB, Maddison PJ. Can progressive resistance training reverse cachexia in patients with rheumatoid arthritis? Results of a pilot study. J Rheumatol 2005;32:1031–9. 15940763

[157] Göksel Karatepe A, Günaydin R, Türkmen G, et al. Effects of home-based exercise program on the functional status and the quality of life in patients with rheumatoid arthritis: 1-year follow-up study. Rheumatol Int 2011;31:171–6. 10.1007/s00296-009-1242-7 19890634

[158] Han A, Robinson V, Judd M, et al. Tai chi for treating rheumatoid arthritis. Cochrane Database Syst Rev 2004;3:CD004849. 10.1002/14651858.CD004849 15266544

[159] Wang C. Tai Chi improves pain and functional status in adults with rheumatoid arthritis: results of a pilot single-blinded randomized controlled trial. Med Sport Sci 2008;52:218–29. 10.1159/000134302 18487901

[160] Shin J-H, Lee Y, Kim SG, et al. The beneficial effects of Tai Chi exercise on endothelial function and arterial stiffness in elderly women with rheumatoid arthritis. Arthritis Res Ther 2015;17:380. 10.1186/s13075-015-0893-x 26702640 PMC4718020

[161] Lee H-Y, Hale CA, Hemingway B, et al. Tai Chi exercise and auricular acupressure for people with rheumatoid arthritis: an evaluation study. J Clin Nurs 2012;21:2812–22. 10.1111/j.1365-2702.2011.04058.x 22830622

[162] Uhlig T, Fongen C, Steen E, et al. Exploring Tai Chi in rheumatoid arthritis: a quantitative and qualitative study. BMC Musculoskelet Disord 2010;11:43. 10.1186/1471-2474-11-43 20205741 PMC2845097

[163] Ward L, Stebbings S, Athens J, et al. Yoga for the management of pain and sleep in rheumatoid arthritis: a pilot randomized controlled trial. Musculoskeletal Care 2018;16:39–47. 10.1002/msc.1201 28621011

[164] Evans S, Moieni M, Lung K, et al. Impact of iyengar yoga on quality of life in young women with rheumatoid arthritis. Clin J Pain 2013;29:988–97. 10.1097/AJP.0b013e31827da381 23370082 PMC3644391

[165] Singh VK, Bhandari RB, Rana BB. Effect of yogic package on rheumatoid arthritis. Indian J Physiol Pharmacol 2011;55:329–35. 23362725

[166] Badsha H, Chhabra V, Leibman C, et al. The benefits of yoga for rheumatoid arthritis: results of a preliminary, structured 8-week program. Rheumatol Int 2009;29:1417–21. 10.1007/s00296-009-0871-1 19184028

[167] Bosch PR, Traustadóttir T, Howard P, et al. Functional and physiological effects of yoga in women with rheumatoid arthritis: a pilot study. Altern Ther Health Med 2009;15:24–31. 19623830

[168] O'Dwyer T, Durcan L, Wilson F. Exercise and physical activity in systemic lupus erythematosus: a systematic review with meta-analyses. Semin Arthritis Rheum 2017;47:204–15. 10.1016/j.semarthrit.2017.04.003 28477898

[169] Wu M-L, Yu K-H, Tsai J-C. The effectiveness of exercise in adults with systemic lupus erythematosus: a systematic review and meta-analysis to guide evidence-based practice. Worldviews Evid Based Nurs 2017;14:306–15. 10.1111/wvn.12221 28432856

[170] Andrades C, Fuego C, Manrique-Arija S, et al. Management of cardiovascular risk in systemic lupus erythematosus: a systematic review. Lupus 2017;26:1407–19. 10.1177/0961203317704710 28457197

[171] del Pino-Sedeño T, Trujillo-Martín MM, Ruiz-Irastorza G, et al. Effectiveness of nonpharmacologic interventions for decreasing fatigue in adults with systemic lupus erythematosus: a systematic review. Arthritis Care Res 2016;68:141–8. 10.1002/acr.22675 26238554

[172] Yuen HK, Cunningham MA. Optimal management of fatigue in patients with systemic lupus erythematosus: a systematic review. Ther Clin Risk Manag 2014;10:775–86. 10.2147/TCRM.S56063 25328393 PMC4199565

[173] Abrahão MI, Gomiero AB, Peccin MS, et al. Cardiovascular training vs. resistance training for improving quality of life and physical function in patients with systemic lupus erythematosus: a randomized controlled trial. Scand J Rheumatol 2016;45:197–201. 10.3109/03009742.2015.1094126 26525835

[174] Boström C, Elfving B, Dupré B, et al. Effects of a one-year physical activity programme for women with systemic lupus erythematosus - a randomized controlled study. Lupus 2016;25:602–16. 10.1177/0961203315622817 26768748

[175] Tench CM, McCarthy J, McCurdie I, et al. Fatigue in systemic lupus erythematosus: a randomized controlled trial of exercise. Rheumatology 2003;42:1050–4. 10.1093/rheumatology/keg289 12730519

[176] Robb-Nicholson LC, Daltroy L, Eaton H, et al. Effects of aerobic conditioning in lupus fatigue: a pilot study. Br J Rheumatol 1989;28:500–5. 10.1093/rheumatology/28.6.500 2590802

[177] Soriano-Maldonado A, Morillas-de-Laguno P, Sabio JM, et al. Effects of 12-week aerobic exercise on arterial stiffness, inflammation, and cardiorespiratory fitness in women with systemic lupus erythematosus: Non-Randomized controlled trial. J Clin Med 2018;7. 10.3390/jcm7120477. [Epub ahead of print: 24 11 2018]. PMC6306776 30477218

[178] dos Reis-Neto ET, da Silva AE, Monteiro CMdeC, et al. Supervised physical exercise improves endothelial function in patients with systemic lupus erythematosus. Rheumatology 2013;52:2187–95. 10.1093/rheumatology/ket283 23970541

[179] Carvalho MRPde, Sato EI, Tebexreni AS, et al. Effects of supervised cardiovascular training program on exercise tolerance, aerobic capacity, and quality of life in patients with systemic lupus erythematosus. Arthritis Rheum 2005;53:838–44. 10.1002/art.21605 16342102

[180] Bogdanovic G, Stojanovich L, Djokovic A, et al. Physical activity program is helpful for improving quality of life in patients with systemic lupus erythematosus. Tohoku J Exp Med 2015;237:193–9. 10.1620/tjem.237.193 26490344

[181] Ramsey-Goldman R, Schilling EM, Dunlop D, et al. A pilot study on the effects of exercise in patients with systemic lupus erythematosus. Arthritis Care Res 2000;13:262–9. 10.1002/1529-0131(200010)13:5&lt;262::aid-anr4&gt;3.0.co;2-8 14635294

[182] Miossi R, Benatti FB, Lúciade de Sá Pinto A, et al. Using exercise training to counterbalance chronotropic incompetence and delayed heart rate recovery in systemic lupus erythematosus: a randomized trial. Arthritis Care Res 2012;64:1159–66. 10.1002/acr.21678 22438298

[183] Timóteo RP, Silva AF, Micheli DC, et al. Increased flexibility, pain reduction and unaltered levels of IL-10 and CD11b + lymphocytes in patients with systemic lupus erythematosus were associated with kinesiotherapy. Lupus 2018;27:1159–68. 10.1177/0961203318768880 29635996

[184] Regel A, Sepriano A, Baraliakos X, et al. Efficacy and safety of non-pharmacological and non-biological pharmacological treatment: a systematic literature review Informing the 2016 update of the ASAS/EULAR recommendations for the management of axial spondyloarthritis. RMD Open 2017;3:e000397. 10.1136/rmdopen-2016-000397 28176966 PMC5278330

[185] O'Dwyer T, O'Shea F, Wilson F. Exercise therapy for spondyloarthritis: a systematic review. Rheumatol Int 2014;34:887–902. 10.1007/s00296-014-2965-7 24549404

[186] Karahan AY, Tok F, Yildirim P, et al. The effectiveness of Exergames in patients with ankylosing spondylitis: a randomized controlled trial. Adv Clin Exp Med 2016;25:931–6. 10.17219/acem/32590 28028958

[187] Jennings F, Oliveira HA, de Souza MC, et al. Effects of aerobic training in patients with ankylosing spondylitis. J Rheumatol 2015;42:2347–53. 10.3899/jrheum.150518 26523029

[188] Niedermann K, Sidelnikov E, Muggli C, et al. Effect of cardiovascular training on fitness and perceived disease activity in people with ankylosing spondylitis. Arthritis Care Res 2013;65:1844–52. 10.1002/acr.22062 23836515

[189] Sweeney S, Taylor G, Calin A. The effect of a home based exercise intervention package on outcome in ankylosing spondylitis: a randomized controlled trial. J Rheumatol 2002;29:763–6. 11950019

[190] Ajeganova S, Wörnert M, Hafström I. A four-week team-rehabilitation programme in a warm climate decreases disability and improves health and body function for up to one year: a prospective study in Swedish patients with inflammatory joint diseases. J Rehabil Med 2016;48:711–8. 10.2340/16501977-2122 27494270

[191] Brophy S, Cooksey R, Davies H, et al. The effect of physical activity and motivation on function in ankylosing spondylitis: a cohort study. Semin Arthritis Rheum 2013;42:619–26. 10.1016/j.semarthrit.2012.09.007 23351615 PMC3685805

[192] Ward MM. Predictors of the progression of functional disability in patients with ankylosing spondylitis. J Rheumatol 2002;29:1420–5. 12136900

[193] Uhrin Z, Kuzis S, Ward MM. Exercise and changes in health status in patients with ankylosing spondylitis. Arch Intern Med 2000;160:2969–75. 10.1001/archinte.160.19.2969 11041905

[194] Pécourneau V, Degboé Y, Barnetche T, et al. Effectiveness of exercise programs in ankylosing spondylitis: a meta-analysis of randomized controlled trials. Arch Phys Med Rehabil 2018;99:383–9. 10.1016/j.apmr.2017.07.015 28860095

[195] Chang W, Tsou Y, Lee C. Comparison between specific exercises and physical therapy for managing patients with ankylosing spondylitis: a meta-analysis of randomized controlled trials. Int J Clin Experiment Med 2016;9:17028–39.

[196] Millner JR, Barron JS, Beinke KM, et al. Exercise for ankylosing spondylitis: an evidence-based consensus statement. Semin Arthritis Rheum 2016;45:411–27. 10.1016/j.semarthrit.2015.08.003 26493464

[197] Liang H, Zhang H, Ji H, et al. Effects of home-based exercise intervention on health-related quality of life for patients with ankylosing spondylitis: a meta-analysis. Clin Rheumatol 2015;34:1737–44. 10.1007/s10067-015-2913-2 25771852

[198] Liang H, Li W-R, Zhang H, et al. Concurrent intervention with exercises and stabilized tumor necrosis factor inhibitor therapy reduced the disease activity in patients with ankylosing spondylitis: a meta-analysis. Medicine 2015;94:e2254. 10.1097/MD.0000000000002254 26683943 PMC5058915

[199] Martins NA, Furtado GE, Campos MJ, et al. Exercise and ankylosing spondylitis with New York modified criteria: a systematic review of controlled trials with meta-analysis. Acta Reumatol Port 2014;39:298–308. 25351868

[200] Sharan D, Rajkumar JS. Physiotherapy for ankylosing spondylitis: systematic review and a proposed rehabilitation protocol. Curr Rheumatol Rev 2017;13:121–5. 10.2174/1573397112666161025112750 27784233

[201] Sveaas SH, Bilberg A, Berg IJ, et al. High intensity exercise for 3 months reduces disease activity in axial spondyloarthritis (axSpA): a multicentre randomised trial of 100 patients. Br J Sports Med 2020;54:292–7. 10.1136/bjsports-2018-099943 30745314

[202] Sveaas SH, Berg IJ, Fongen C, et al. High-intensity cardiorespiratory and strength exercises reduced emotional distress and fatigue in patients with axial spondyloarthritis: a randomized controlled pilot study. Scand J Rheumatol 2018;47:117–21. 10.1080/03009742.2017.1347276 28891743

[203] Aydın T, Taşpınar Özgür, Sarıyıldız MA, et al. Evaluation of the effectiveness of home based or hospital based calisthenic exercises in patients with ankylosing spondylitis. J Back Musculoskelet Rehabil 2016;29:723–30. 10.3233/BMR-160677 26966823

[204] Roşu MO, Ţopa I, Chirieac R, et al. Effects of Pilates, McKenzie and Heckscher training on disease activity, spinal motility and pulmonary function in patients with ankylosing spondylitis: a randomized controlled trial. Rheumatol Int 2014;34:367–72. 10.1007/s00296-013-2869-y 24071935

[205] Sveaas SH, Berg IJ, Provan SA, et al. Efficacy of high intensity exercise on disease activity and cardiovascular risk in active axial spondyloarthritis: a randomized controlled pilot study. PLoS One 2014;9:e108688. 10.1371/journal.pone.0108688 25268365 PMC4182541

[206] Kjeken I, Bo I, Rønningen A, et al. A three-week multidisciplinary in-patient rehabilitation programme had positive long-term effects in patients with ankylosing spondylitis: randomized controlled trial. J Rehabil Med 2013;45:260–7. 10.2340/16501977-1078 23138412

[207] Analay Y, Ozcan E, Karan A, et al. The effectiveness of intensive group exercise on patients with ankylosing spondylitis. Clin Rehabil 2003;17:631–6. 10.1191/0269215503cr658oa 12971708

[208] Hidding A, van der Linden S, Gielen X, et al. Continuation of group physical therapy is necessary in ankylosing spondylitis: results of a randomized controlled trial. Arthritis Care Res 1994;7:90–6. 10.1002/art.1790070208 7857999

[209] Hidding A, van der Linden S, Boers M, et al. Is group physical therapy superior to individualized therapy in ankylosing spondylitis? A randomized controlled trial. Arthritis Care Res 1993;6:117–25. 10.1002/art.1790060303 8130287

[210] Kraag G, Stokes B, Groh J, et al. The effects of comprehensive home physiotherapy and supervision on patients with ankylosing spondylitis--a randomized controlled trial. J Rheumatol 1990;17:228–33. 2181127

[211] Levitova A, Hulejova H, Spiritovic M, et al. Clinical improvement and reduction in serum calprotectin levels after an intensive exercise programme for patients with ankylosing spondylitis and non-radiographic axial spondyloarthritis. Arthritis Res Ther 2016;18:275. 10.1186/s13075-016-1180-1 27887637 PMC5124318

[212] Aytekin E, Caglar NS, Ozgonenel L, et al. Home-based exercise therapy in patients with ankylosing spondylitis: effects on pain, mobility, disease activity, quality of life, and respiratory functions. Clin Rheumatol 2012;31:91–7. 10.1007/s10067-011-1791-5 21656347

[213] Viitanen JV, Heikkilä S. Functional changes in patients with spondylarthropathy. A controlled trial of the effects of short-term rehabilitation and 3-year follow-up. Rheumatol Int 2001;20:211–4. 10.1007/s002960100101 11518042

[214] Lubrano E, D'Angelo S, Parsons WJ, et al. Effectiveness of rehabilitation in active ankylosing spondylitis assessed by the ASAS response criteria. Rheumatology 2007;46:1672–5. 10.1093/rheumatology/kem247 17893100

[215] Band DA, Jones SD, Kennedy LG, et al. Which patients with ankylosing spondylitis derive most benefit from an inpatient management program? J Rheumatol 1997;24:2381–4. 9415646

[216] Viitanen JV, Lehtinen K, Suni J, et al. Fifteen months' follow-up of intensive inpatient physiotherapy and exercise in ankylosing spondylitis. Clin Rheumatol 1995;14:413–9. 10.1007/BF02207674 7586977

[217] Kraag G, Stokes B, Groh J, et al. The effects of comprehensive home physiotherapy and supervision on patients with ankylosing spondylitis--an 8-month followup. J Rheumatol 1994;21:261–3. 8182634

[218] Viitanen JV, Suni J, Kautiainen H, et al. Effect of physiotherapy on spinal mobility in ankylosing spondylitis. Scand J Rheumatol 1992;21:38–41. 10.3109/03009749209095061 1570486

[219] Escalas C, Dalichampt M, Dougados M, et al. Evaluation of physiotherapy in a prospective cohort of early axial spondyloarthritis. Data from the DESIR cohort. Joint Bone Spine 2016;83:185–90. 10.1016/j.jbspin.2015.05.008 26677991

[220] Zão A, Cantista P. The role of land and aquatic exercise in ankylosing spondylitis: a systematic review. Rheumatol Int 2017;37:1979–90. 10.1007/s00296-017-3829-8 28983663

[221] Dundar U, Solak O, Toktas H, et al. Effect of aquatic exercise on ankylosing spondylitis: a randomized controlled trial. Rheumatol Int 2014;34:1505–11. 10.1007/s00296-014-2980-8 24626605

[222] Karapolat H, Eyigor S, Zoghi M, et al. Are swimming or aerobic exercise better than conventional exercise in ankylosing spondylitis patients? A randomized controlled study. Eur J Phys Rehabil Med 2009;45:449–57. 20032902

[223] Hsieh L-F, Chuang C-C, Tseng C-S, et al. Combined home exercise is more effective than range-of-motion home exercise in patients with ankylosing spondylitis: a randomized controlled trial. Biomed Res Int 2014;2014:398190. 10.1155/2014/398190 25276785 PMC4170701

[224] Rodríguez-Lozano C, Juanola X, Cruz-Martínez J, et al. Outcome of an education and home-based exercise programme for patients with ankylosing spondylitis: a nationwide randomized study. Clin Exp Rheumatol 2013;31:739–48. 23899791

[225] Lim H-J, Moon Y-I, Lee MS. Effects of home-based daily exercise therapy on joint mobility, daily activity, pain, and depression in patients with ankylosing spondylitis. Rheumatol Int 2005;25:225–9. 10.1007/s00296-004-0536-z 15650833

[226] Yigit S, Sahin Z, Demir SE, et al. Home-based exercise therapy in ankylosing spondylitis: short-term prospective study in patients receiving tumor necrosis factor alpha inhibitors. Rheumatol Int 2013;33:71–7. 10.1007/s00296-011-2344-6 22218641

[227] Durmus D, Alayli G, Cil E, et al. Effects of a home-based exercise program on quality of life, fatigue, and depression in patients with ankylosing spondylitis. Rheumatol Int 2009;29:673–7. 10.1007/s00296-008-0756-8 18985351

[228] Durmuş D, Alaylı G, Uzun O, et al. Effects of two exercise interventions on pulmonary functions in the patients with ankylosing spondylitis. Joint Bone Spine 2009;76:150–5. 10.1016/j.jbspin.2008.06.013 19084457

[229] Karapolat H, Akkoc Y, Sari I, et al. Comparison of group-based exercise versus home-based exercise in patients with ankylosing spondylitis: effects on Bath ankylosing spondylitis indices, quality of life and depression. Clin Rheumatol 2008;27:695–700. 10.1007/s10067-007-0765-0 17985194

[230] Souza MCde, Jennings F, Morimoto H, et al. Swiss ball exercises improve muscle strength and walking performance in ankylosing spondylitis: a randomized controlled trial. Rev Bras Reumatol Engl Ed 2017;57:45–55. 10.1016/j.rbre.2016.09.009 28137402

[231] Kasapoglu Aksoy M, Birtane M, Taştekin N, et al. The effectiveness of structured group education on ankylosing spondylitis patients. J Clin Rheumatol 2017;23:138–43. 10.1097/RHU.0000000000000499 28248799

[232] Rosu OM, Ancuta C. McKenzie training in patients with early stages of ankylosing spondylitis: results of a 24-week controlled study. Eur J Phys Rehabil Med 2015;51:261–8. 25358635

[233] Masiero S, Poli P, Bonaldo L, et al. Supervised training and home-based rehabilitation in patients with stabilized ankylosing spondylitis on TNF inhibitor treatment: a controlled clinical trial with a 12-month follow-up. Clin Rehabil 2014;28:562–72. 10.1177/0269215513512214 24285801

[234] Altan L, Korkmaz N, Dizdar M, et al. Effect of Pilates training on people with ankylosing spondylitis. Rheumatol Int 2012;32:2093–9. 10.1007/s00296-011-1932-9 21499876

[235] Masiero S, Bonaldo L, Pigatto M, et al. Rehabilitation treatment in patients with ankylosing spondylitis stabilized with tumor necrosis factor inhibitor therapy: a randomized controlled trial. J Rheumatol 2011;38:1335–42. 10.3899/jrheum.100987 21459942

[236] Fernández-de-Las-Peñas C, Alonso-Blanco C, Alguacil-Diego IM, et al. One-year follow-up of two exercise interventions for the management of patients with ankylosing spondylitis: a randomized controlled trial. Am J Phys Med Rehabil 2006;85:559–67. 10.1097/01.phm.0000223358.25983.df 16788386

[237] Fernández-de-Las-Peñas C, Alonso-Blanco C, Morales-Cabezas M, et al. Two exercise interventions for the management of patients with ankylosing spondylitis: a randomized controlled trial. Am J Phys Med Rehabil 2005;84:407–19. 10.1097/01.phm.0000163862.89217.fe 15905654

[238] Gyurcsik ZN, András A, Bodnár N, et al. Improvement in pain intensity, spine stiffness, and mobility during a controlled individualized physiotherapy program in ankylosing spondylitis. Rheumatol Int 2012;32:3931–6. 10.1007/s00296-011-2325-9 22198694

[239] Hulejová H, Levitová A, Kuklová M, et al. No effect of physiotherapy on the serum levels of adipocytokines in patients with ankylosing spondylitis. Clin Rheumatol 2012;31:67–71. 10.1007/s10067-011-1773-7 21618078

[240] Ortancil O, Sarikaya S, Sapmaz P, et al. The effect(s) of a six-week home-based exercise program on the respiratory muscle and functional status in ankylosing spondylitis. J Clin Rheumatol 2009;15:68–70. 10.1097/RHU.0b013e31819b5ed0 19265348

[241] Roger-Silva D, Natour J, Moreira E, et al. A resistance exercise program improves functional capacity of patients with psoriatic arthritis: a randomized controlled trial. Clin Rheumatol 2018;37:389–95. 10.1007/s10067-017-3917-x 29185133

[242] Chimenti MS, Triggianese P, Conigliaro P, et al. Self-reported adherence to a home-based exercise program among patients affected by psoriatic arthritis with minimal disease activity. Drug Dev Res 2014;75(Suppl 1):S57–9. 10.1002/ddr.21197 25381979

[243] Antonioli CM, Bua G, Frigè A, et al. An individualized rehabilitation program in patients with systemic sclerosis may improve quality of life and hand mobility. Clin Rheumatol 2009;28:159–65. 10.1007/s10067-008-1006-x 18795394

[244] Moran ME. Scleroderma and evidence based non-pharmaceutical treatment modalities for digital ulcers: a systematic review. J Wound Care 2014;23:510–6. 10.12968/jowc.2014.23.10.510 25296352

[245] Rannou F, Boutron I, Mouthon L, et al. Personalized physical therapy versus usual care for patients with systemic sclerosis: a randomized controlled trial. Arthritis Care Res 2017;69:1050–9. 10.1002/acr.23098 27696703

[246] Schouffoer AA, Ninaber MK, Beaart-van de Voorde LJJ, et al. Randomized comparison of a multidisciplinary team care program with usual care in patients with systemic sclerosis. Arthritis Care Res 2011;63:909–17. 10.1002/acr.20448 21312348

[247] Pinto ALS, Oliveira NC, Gualano B, et al. Efficacy and safety of concurrent training in systemic sclerosis. J Strength Cond Res 2011;25:1423–8. 10.1519/JSC.0b013e3181d6858b 21116202

[248] Maddali Bongi S, Del Rosso A, Galluccio F, et al. Efficacy of a tailored rehabilitation program for systemic sclerosis. Clin Exp Rheumatol 2009;27:44–50. 19796561

[249] Stefanantoni K, Sciarra I, Iannace N, et al. Occupational therapy integrated with a self-administered stretching program on systemic sclerosis patients with hand involvement. Clin Exp Rheumatol 2016;34(Suppl 100):157–61. 27087678

[250] Horváth J, Bálint Z, Szép E, et al. Efficacy of intensive hand physical therapy in patients with systemic sclerosis. Clin Exp Rheumatol 2017;35(Suppl 106):159–66. 28869417

[251] Mugii N, Matsushita T, Oohata S, et al. Long-term follow-up of finger passive range of motion in Japanese systemic sclerosis patients treated with self-administered stretching. Mod Rheumatol 2019;29:484–90. 10.1080/14397595.2018.1466635 29667474

[252] Landim SF, Bertolo MB, Marcatto de Abreu MF, et al. The evaluation of a home-based program for hands in patients with systemic sclerosis. J Hand Ther 2019;32:313–21. 10.1016/j.jht.2017.10.013 29198478

[253] Mugii N, Hasegawa M, Matsushita T, et al. The efficacy of self-administered stretching for finger joint motion in Japanese patients with systemic sclerosis. J Rheumatol 2006;33:1586–92. 16881115

[254] Ma L, Sun R, Jia Z, et al. Clinical characteristics associated with subcutaneous tophi formation in Chinese gout patients: a retrospective study. Clin Rheumatol 2018;37:1359–65. 10.1007/s10067-017-3969-y 29354873

[255] Adithya Acharya K, Sharma A. Evaluation of the efficacy of siravyadha and guduchi siddha yoga basti in the management of vatarakta with special reference to gout. Int J Res Ayuveda Pharm 2019;4:402–9.

[256] Sadeghi A, Rad ZA, Sajedi B, et al. Effect of weight losing on the clinical status improvement of patients with knee osteoarthritis. Reumatol Clin 2019;15:73–6. 10.1016/j.reuma.2017.06.016 29102588

[257] O'Brien KM, Wiggers J, Williams A, et al. Telephone-based weight loss support for patients with knee osteoarthritis: a pragmatic randomised controlled trial. Osteoarthritis Cartilage 2018;26:485–94. 10.1016/j.joca.2018.01.003 29330101

[258] Allen KD, Oddone EZ, Coffman CJ, et al. Patient, provider, and combined interventions for managing osteoarthritis in primary care: a cluster randomized trial. Ann Intern Med 2017;166:401–11. 10.7326/M16-1245 28114648 PMC6862719

[259] Allen KD, Yancy WS, Bosworth HB, et al. A combined patient and provider intervention for management of osteoarthritis in veterans. Ann Intern Med 2016;164:73–83. 10.7326/M15-0378 26720751 PMC4732728

[260] Christensen R, Henriksen M, Leeds AR, et al. Effect of weight maintenance on symptoms of knee osteoarthritis in obese patients: a twelve-month randomized controlled trial. Arthritis Care Res 2015;67:640–50. 10.1002/acr.22504 PMC4657487 25370359

[261] Hunter DJ, Beavers DP, Eckstein F, et al. The intensive diet and exercise for arthritis (IDEA) trial: 18-month radiographic and MRI outcomes. Osteoarthritis Cartilage 2015;23:1090–8. 10.1016/j.joca.2015.03.034 25887362 PMC9178604

[262] Saraboon Y, Aree-Ue S, Maruo SJ. The effect of multifactorial intervention programs on health behavior and symptom control among community-dwelling overweight older adults with knee osteoarthritis. Orthop Nurs 2015;34:296–308. 10.1097/NOR.0000000000000180 26375841

[263] Beavers DP, Beavers KM, Loeser RF, et al. The independent and combined effects of intensive weight loss and exercise training on bone mineral density in overweight and obese older adults with osteoarthritis. Osteoarthritis Cartilage 2014;22:726–33. 10.1016/j.joca.2014.04.002 24742955 PMC4051847

[264] Henriksen M, Christensen R, Hunter DJ, et al. Structural changes in the knee during weight loss maintenance after a significant weight loss in obese patients with osteoarthritis: a report of secondary outcome analyses from a randomized controlled trial. Osteoarthritis Cartilage 2014;22:639–46. 10.1016/j.joca.2014.03.003 24636948

[265] Messier SP, Mihalko SL, Legault C, et al. Effects of intensive diet and exercise on knee joint loads, inflammation, and clinical outcomes among overweight and obese adults with knee osteoarthritis: the IDEA randomized clinical trial. JAMA 2013;310:1263–73. 10.1001/jama.2013.277669 24065013 PMC4450354

[266] Somers TJ, Blumenthal JA, Guilak F, et al. Pain coping skills training and lifestyle behavioral weight management in patients with knee osteoarthritis: a randomized controlled study. Pain 2012;153:1199–209. 10.1016/j.pain.2012.02.023 22503223 PMC3358356

[267] Bliddal H, Leeds AR, Stigsgaard L, et al. Weight loss as treatment for knee osteoarthritis symptoms in obese patients: 1-year results from a randomised controlled trial. Ann Rheum Dis 2011;70:1798–803. 10.1136/ard.2010.142018 21821622

[268] Gudbergsen H, Boesen M, Christensen R, et al. Radiographs and low field MRI (0.2T) as predictors of efficacy in a weight loss trial in obese women with knee osteoarthritis. BMC Musculoskelet Disord 2011;12. 10.1186/1471-2474-12-56 PMC3065448 21356061

[269] Riecke BF, Christensen R, Christensen P, et al. Comparing two low-energy diets for the treatment of knee osteoarthritis symptoms in obese patients: a pragmatic randomized clinical trial. Osteoarthritis Cartilage 2010;18:746–54. 10.1016/j.joca.2010.02.012 20206314

[270] Ravaud P, Flipo RM, Boutron I. ARTIST (osteoarthritis intervention standardized) study of standardised consultation versus usual care for patients with osteoarthritis of the knee in primary care in France: pragmatic randomised controlled trial. BMJ 2009;338:b421. 19237406 10.1136/bmj.b421 PMC2651104

[271] Miller GD, Nicklas BJ, Loeser RF. Inflammatory biomarkers and physical function in older, obese adults with knee pain and self-reported osteoarthritis after intensive weight-loss therapy. J Am Geriatr Soc 2008;56:644–51. 10.1111/j.1532-5415.2007.01636.x 18312558 PMC9899049

[272] Miller GD, Nicklas BJ, Davis C, et al. Intensive weight loss program improves physical function in older obese adults with knee osteoarthritis. Obesity 2006;14:1219–30. 10.1038/oby.2006.139 16899803

[273] Christensen R, Astrup A, Bliddal H. Weight loss: the treatment of choice for knee osteoarthritis? A randomized trial. Osteoarthritis Cartilage 2005;13:20–7. 10.1016/j.joca.2004.10.008 15639633

[274] Messier SP, Loeser RF, Miller GD, et al. Exercise and dietary weight loss in overweight and obese older adults with knee osteoarthritis: the arthritis, diet, and activity promotion trial. Arthritis Rheum 2004;50:1501–10. 10.1002/art.20256 15146420

[275] Rejeski WJ, Focht BC, Messier SP, et al. Obese, older adults with knee osteoarthritis: weight loss, exercise, and quality of life. Health Psychol 2002;21:419–26. 10.1037/0278-6133.21.5.419 12211508

[276] Messier SP, Loeser RF, Mitchell MN, et al. Exercise and weight loss in obese older adults with knee osteoarthritis: a preliminary study. J Am Geriatr Soc 2000;48:1062–72. 10.1111/j.1532-5415.2000.tb04781.x 10983905

[277] Toda Y. The effect of energy restriction, walking, and exercise on lower extremity lean body mass in obese women with osteoarthritis of the knee. J Orthop Sci 2001;6:148–54. 10.1007/s007760100063 11484101

[278] Huang MH, Chen CH, Chen TW, et al. The effects of weight reduction on the rehabilitation of patients with knee osteoarthritis and obesity. Arthritis Care Res 2000;13:398–405. 10.1002/1529-0131(200012)13:6<398::AID-ART10>3.0.CO;2-E 14635316

[279] Bartholdy C, Christensen R, Kristensen LE, et al. Association between weight loss and spontaneous changes in physical inactivity in overweight/obese individuals with knee osteoarthritis: an 8-week prospective cohort study. Arthritis Care Res 2020;72:397–404. 10.1002/acr.23868 30821925

[280] Aree-Ue S, Saraboon Y, Belza B. Long-Term adherence and effectiveness of a multicomponent intervention for community-dwelling overweight Thai older adults with knee osteoarthritis: 1-year follow up. J Gerontol Nurs 2017;43:40–8. 10.3928/00989134-20170111-09 28095581

[281] Atukorala I, Makovey J, Lawler L, et al. Is there a dose-response relationship between weight loss and symptom improvement in persons with knee osteoarthritis? Arthritis Care Res 2016;68:1106–14. 10.1002/acr.22805 26784732

[282] Bartels EM, Christensen R, Christensen P, et al. Effect of a 16 weeks weight loss program on osteoarthritis biomarkers in obese patients with knee osteoarthritis: a prospective cohort study. Osteoarthritis Cartilage 2014;22:1817-25. 10.1016/j.joca.2014.07.027 25106676

[283] Paans N, van den Akker-Scheek I, Dilling RG, et al. Effect of exercise and weight loss in people who have hip osteoarthritis and are overweight or obese: a prospective cohort study. Phys Ther 2013;93:137–46. 10.2522/ptj.20110418 23023813

[284] Gudbergsen H, Boesen M, Lohmander LS, et al. Weight loss is effective for symptomatic relief in obese subjects with knee osteoarthritis independently of joint damage severity assessed by high-field MRI and radiography. Osteoarthritis Cartilage 2012;20:495–502. 10.1016/j.joca.2012.02.639 22401872

[285] Bihlet AR, Byrjalsen I, Bay-Jensen A-C, et al. Identification of pain categories associated with change in pain in patients receiving placebo: data from two phase 3 randomized clinical trials in symptomatic knee osteoarthritis. BMC Musculoskelet Disord 2018;19:17. 10.1186/s12891-018-1938-5 29343266 PMC5773024

[286] Han A, Gellhorn AC. Trajectories of quality of life and associated risk factors in patients with knee osteoarthritis: findings from the osteoarthritis initiative. Am J Phys Med Rehabil 2018;97:620–7. 10.1097/PHM.0000000000000926 29547449

[287] Jacobs CA, Vranceanu A-M, Thompson KL, et al. Rapid progression of knee pain and osteoarthritis biomarkers greatest for patients with combined obesity and depression: data from the osteoarthritis initiative. Cartilage 2020;11:38–46. 10.1177/1947603518777577 29855190 PMC6921961

[288] Pelletier J-P, Raynauld J-P, Abram F, et al. Exploring determinants predicting response to intra-articular hyaluronic acid treatment in symptomatic knee osteoarthritis: 9-year follow-up data from the osteoarthritis initiative. Arthritis Res Ther 2018;20:40. 10.1186/s13075-018-1538-7 29490683 PMC5831607

[289] Eymard F, Chevalier X, Conrozier T. Obesity and radiological severity are associated with viscosupplementation failure in patients with knee osteoarthritis. J Orthop Res 2017;35:2269–74. 10.1002/jor.23529 28128473

[290] Moyer R, Wirth W, Eckstein F. Longitudinal changes in magnetic resonance imaging-based measures of femorotibial cartilage thickness as a function of alignment and obesity: data from the osteoarthritis initiative. Arthritis Care Res 2017;69:959–65. 10.1002/acr.23096 PMC5585678 27696763

[291] Bastick AN, Verkleij SPJ, Damen J, et al. Defining hip pain trajectories in early symptomatic hip osteoarthritis--5 year results from a nationwide prospective cohort study (CHECK). Osteoarthritis Cartilage 2016;24:768-75. 10.1016/j.joca.2015.11.023 26854794

[292] de Rezende MU, Hissadomi MI, de Campos GC, et al. One-Year results of an educational program on osteoarthritis: a prospective randomized controlled trial in Brazil. Geriatr Orthop Surg Rehabil 2016;7:86–94. 10.1177/2151458516645634 27239382 PMC4872185

[293] Beavers KM, Beavers DP, Newman JJ, et al. Effects of total and regional fat loss on plasma CRP and IL-6 in overweight and obese, older adults with knee osteoarthritis. Osteoarthritis Cartilage 2015;23:249–56. 10.1016/j.joca.2014.11.005 25450847 PMC4304884

[294] Chatterjee D, McGee A, Strauss E, et al. Subchondral calcium phosphate is ineffective for bone marrow edema lesions in adults with advanced osteoarthritis. Clin Orthop Relat Res 2015;473:2334–42. 10.1007/s11999-015-4311-0 25917421 PMC4457753

[295] Karsdal MA, Bihlet A, Byrjalsen I, et al. OA phenotypes, rather than disease stage, drive structural progression--identification of structural progressors from 2 phase III randomized clinical studies with symptomatic knee OA. Osteoarthritis Cartilage 2015;23:550–8. 10.1016/j.joca.2014.12.024 25576879

[296] Kobayashi N, Inaba Y, Yukizawa Y, et al. Use of 18F-fluoride positron emission tomography as a predictor of the hip osteoarthritis progression. Mod Rheumatol 2015;25:925–30. 10.3109/14397595.2015.1045257 25967130

[297] Magnusson K, Slatkowsky-Christensen B, van der Heijde D, et al. Body mass index and progressive hand osteoarthritis: data from the Oslo hand osteoarthritis cohort. Scand J Rheumatol 2015;44:331–6. 10.3109/03009742.2014.994560 25742965

[298] Gudbergsen H, Boesen M, Christensen R, et al. Changes in bone marrow lesions in response to weight-loss in obese knee osteoarthritis patients: a prospective cohort study. BMC Musculoskelet Disord 2013;14:106. 10.1186/1471-2474-14-106 23522337 PMC3618315

[299] Perrot S, Bertin P. "Feeling better" or "feeling well" in usual care of hip and knee osteoarthritis pain: determination of cutoff points for patient acceptable symptom state (PASS) and minimal clinically important improvement (MCII) at rest and on movement in a national multicenter cohort study of 2414 patients with painful osteoarthritis. Pain 2013;154:248–56. 10.1016/j.pain.2012.10.017 23265687

[300] Coffman CJ, Allen KD, Woolson RF. Mixed-effects regression modeling of real-time momentary pain assessments in osteoarthritis (OA) patients. Health Serv Outcomes Res Method 2012;12:200–18. 10.1007/s10742-012-0085-y

[301] Miyazaki T, Uchida K, Sato M, et al. Knee laxity after staircase exercise predicts radiographic disease progression in medial compartment knee osteoarthritis. Arthritis Rheum 2012;64:3908–16. 10.1002/art.34662 22886496

[302] Rabago D, Zgierska A, Fortney L, et al. Hypertonic dextrose injections (prolotherapy) for knee osteoarthritis: results of a single-arm uncontrolled study with 1-year follow-up. J Altern Complement Med 2012;18:408–14. 10.1089/acm.2011.0030 22515800 PMC3326267

[303] Sands GH, Brown PB, Essex MN. The Efficacy of Continuous Versus Intermittent Celecoxib Treatment in Osteoarthritis Patients with Body Mass Index ≥30 and <30 kg/m(2.). Open Rheumatol J 2013;7:32–7. 10.2174/1874312901307010032 23919092 PMC3731795

[304] Bartlett SJ, Ling SM, Mayo NE, et al. Identifying common trajectories of joint space narrowing over two years in knee osteoarthritis. Arthritis Care Res 2011;63:1722–8. 10.1002/acr.20614 21905250

[305] Bingham CO, Smugar SS, Wang H, et al. Predictors of response to cyclo-oxygenase-2 inhibitors in osteoarthritis: pooled results from two identical trials comparing etoricoxib, celecoxib, and placebo. Pain Med 2011;12:352–61. 10.1111/j.1526-4637.2011.01060.x 21332932

[306] Nishimura A, Hasegawa M, Kato K, et al. Risk factors for the incidence and progression of radiographic osteoarthritis of the knee among Japanese. Int Orthop 2011;35:839–43. 10.1007/s00264-010-1073-x 20559829 PMC3103966

[307] Richette P, Poitou C, Garnero P, et al. Benefits of massive weight loss on symptoms, systemic inflammation and cartilage turnover in obese patients with knee osteoarthritis. Ann Rheum Dis 2011;70:139–44. 10.1136/ard.2010.134015 20980288

[308] Woollard JD, Gil AB, Sparto P, et al. Change in knee cartilage volume in individuals completing a therapeutic exercise program for knee osteoarthritis. J Orthop Sports Phys Ther 2011;41:708–22. 10.2519/jospt.2011.3633 21891881 PMC3383656

[309] Yusuf E, Bijsterbosch J, Slagboom PE, et al. Body mass index and alignment and their interaction as risk factors for progression of knees with radiographic signs of osteoarthritis. Osteoarthritis Cartilage 2011;19:1117–22. 10.1016/j.joca.2011.06.001 21722745

[310] Shea MK, Houston DK, Nicklas BJ, et al. The effect of randomization to weight loss on total mortality in older overweight and obese adults: the ADAPT study. J Gerontol A Biol Sci Med Sci 2010;65:519–25. 10.1093/gerona/glp217 20080875 PMC3107029

[311] Eckstein F, Maschek S, Wirth W, et al. One year change of knee cartilage morphology in the first release of participants from the osteoarthritis initiative progression subcohort: association with sex, body mass index, symptoms and radiographic osteoarthritis status. Ann Rheum Dis 2009;68:674–9. 10.1136/ard.2008.089904 18519425 PMC2976866

[312] Le Graverand M-PH, Brandt K, Mazzuca SA, et al. Progressive increase in body mass index is not associated with a progressive increase in joint space narrowing in obese women with osteoarthritis of the knee. Ann Rheum Dis 2009;68:1734–8. 10.1136/ard.2007.085530 19060003

[313] Botha-Scheepers S, Dougados M, Ravaud P, et al. Effect of medial tibial plateau alignment on serial radiographs on the capacity to predict progression of knee osteoarthritis. Osteoarthritis Cartilage 2008;16:272–6. 10.1016/j.joca.2007.10.020 18262805

[314] Davies-Tuck ML, Wluka AE, Wang Y, et al. The natural history of cartilage defects in people with knee osteoarthritis. Osteoarthritis Cartilage 2008;16:337–42. 10.1016/j.joca.2007.07.005 17698376

[315] Pelletier J-P, Raynauld J-P, Berthiaume M-J, et al. Risk factors associated with the loss of cartilage volume on weight-bearing areas in knee osteoarthritis patients assessed by quantitative magnetic resonance imaging: a longitudinal study. Arthritis Res Ther 2007;9:R74. 10.1186/ar2272 17672891 PMC2206376

[316] Reijman M, Pols HAP, Bergink AP, et al. Body mass index associated with onset and progression of osteoarthritis of the knee but not of the hip: the Rotterdam study. Ann Rheum Dis 2007;66:158–62. 10.1136/ard.2006.053538 16837490 PMC1798486

[317] Raynauld J-P, Martel-Pelletier J, Berthiaume M-J, et al. Long term evaluation of disease progression through the quantitative magnetic resonance imaging of symptomatic knee osteoarthritis patients: correlation with clinical symptoms and radiographic changes. Arthritis Res Ther 2006;8:R21. 10.1186/ar1875 16507119 PMC1526551

[318] Wluka AE, Forbes A, Wang Y, et al. Knee cartilage loss in symptomatic knee osteoarthritis over 4.5 years. Arthritis Res Ther 2006;8:R90. 10.1186/ar1962 16704746 PMC1779368

[319] Sharma L, Cahue S, Song J, et al. Physical functioning over three years in knee osteoarthritis: role of psychosocial, local mechanical, and neuromuscular factors. Arthritis Rheum 2003;48:3359–70. 10.1002/art.11420 14673987

[320] Cicuttini F, Wluka A, Wang Y, et al. The determinants of change in patella cartilage volume in osteoarthritic knees. J Rheumatol 2002;29:2615–9. 12465162

[321] Wolfe F, Lane NE. The longterm outcome of osteoarthritis: rates and predictors of joint space narrowing in symptomatic patients with knee osteoarthritis. J Rheumatol 2002;29:139–46. 11824950

[322] Detora LM, Krupa D, Bolognese J, et al. Rofecoxib shows consistent efficacy in osteoarthritis clinical trials, regardless of specific patient demographic and disease factors. J Rheumatol 2001;28:2494–503. 11708424

[323] Cooper C, Snow S, McAlindon TE, et al. Risk factors for the incidence and progression of radiographic knee osteoarthritis. Arthritis Rheum 2000;43:995. 10.1002/1529-0131(200005)43:5<995::AID-ANR6>3.0.CO;2-1 10817551

[324] Harris PA, Hart DJ, Dacre JE, et al. The progression of radiological hand osteoarthritis over ten years: a clinical follow-up study. Osteoarthritis Cartilage 1994;2:247–52. 10.1016/S1063-4584(05)80076-7 11550709

[325] Ledingham J, Dawson S, Preston B, et al. Radiographic progression of hospital referred osteoarthritis of the hip. Ann Rheum Dis 1993;52:263–7. 10.1136/ard.52.4.263 8484691 PMC1005623

[326] Schouten JS, van den Ouweland FA, Valkenburg HA. A 12 year follow up study in the general population on prognostic factors of cartilage loss in osteoarthritis of the knee. Ann Rheum Dis 1992;51:932–7. 10.1136/ard.51.8.932 1417116 PMC1004797

[327] Berkhout B, Macfarlane JD, Cats A. Symptomatic osteoarthrosis of the knee: a follow-up study. Br J Rheumatol 1985;24:40–5. 10.1093/rheumatology/24.1.40 3978365

[328] Ahn JH, Kang HW, Yang TY, et al. Risk factors for radiographic progression of osteoarthritis after meniscus allograft transplantation. Arthroscopy 2016;32:2539–46. 10.1016/j.arthro.2016.04.023 27296871

[329] Liu Y, Hazlewood GS, Kaplan GG, et al. Impact of obesity on remission and disease activity in rheumatoid arthritis: a systematic review and meta-analysis. Arthritis Care Res 2017;69:157–65. 10.1002/acr.22932 27159376

[330] Lupoli R, Pizzicato P, Scalera A, et al. Impact of body weight on the achievement of minimal disease activity in patients with rheumatic diseases: a systematic review and meta-analysis. Arthritis Res Ther 2016;18:297. 10.1186/s13075-016-1194-8 27964760 PMC5155390

[331] Baghdadi LR, Woodman RJ, Shanahan EM, et al. The impact of traditional cardiovascular risk factors on cardiovascular outcomes in patients with rheumatoid arthritis: a systematic review and meta-analysis. PLoS One 2015;10:e0117952. 10.1371/journal.pone.0117952 25689371 PMC4331556

[332] Baker JF, Stokes A, Mikuls TR, et al. Current and early life weight and associations with mortality in rheumatoid arthritis. Clin Exp Rheumatol 2019;37:768–73. 30719967

[333] Hirose W, Harigai M, Uchiyama T, et al. Low body mass index and lymphocytopenia associate with Mycobacterium avium complex pulmonary disease in patients with rheumatoid arthritis. Mod Rheumatol 2019;29:105–12. 10.1080/14397595.2018.1452334 29532704

[334] Lechtenboehmer CA, Jaeger VK, Kyburz D, et al. Brief report: influence of disease activity in rheumatoid arthritis on radiographic progression of concomitant interphalangeal joint osteoarthritis. Arthritis Rheumatol 2019;71:43–9. 10.1002/art.40684 30073800

[335] England BR, Baker JF, Sayles H, et al. Body mass index, weight loss, and cause-specific mortality in rheumatoid arthritis. Arthritis Care Res 2018;70:11–18. 10.1002/acr.23258 PMC5650561 28426913

[336] Nikiphorou E, Norton S, Young A, et al. The association of obesity with disease activity, functional ability and quality of life in early rheumatoid arthritis: data from the early rheumatoid arthritis Study/Early rheumatoid arthritis network UK prospective cohorts. Rheumatology 2018;57:1194–202. 10.1093/rheumatology/key066 29590474

[337] Rydell E, Forslind K, Nilsson Jan-Åke, et al. Smoking, body mass index, disease activity, and the risk of rapid radiographic progression in patients with early rheumatoid arthritis. Arthritis Res Ther 2018;20:82. 10.1186/s13075-018-1575-2 29720260 PMC5932864

[338] Schulman E, Bartlett SJ, Schieir O, et al. Overweight, obesity, and the likelihood of achieving sustained remission in early rheumatoid arthritis: results from a multicenter prospective cohort study. Arthritis Care Res 2018;70:1185–91. 10.1002/acr.23457 29193840

[339] Smolen JS, Szumski A, Koenig AS, et al. Predictors of remission with etanercept-methotrexate induction therapy and loss of remission with etanercept maintenance, reduction, or withdrawal in moderately active rheumatoid arthritis: results of the PRESERVE trial. Arthritis Res Ther 2018;20:8. 10.1186/s13075-017-1484-9 29338762 PMC5771183

[340] Sparks JA, Chang S-C, Nguyen U-S, et al. Weight change during the early rheumatoid arthritis period and risk of subsequent mortality in women with rheumatoid arthritis and matched comparators. Arthritis Rheumatol 2018;70:18–29. 10.1002/art.40346 29193837 PMC5745282

[341] van der Heijde D, Durez P, Schett G, et al. Structural damage progression in patients with early rheumatoid arthritis treated with methotrexate, baricitinib, or baricitinib plus methotrexate based on clinical response in the phase 3 RA-BEGIN study. Clin Rheumatol 2018;37:2381–90. 10.1007/s10067-018-4221-0 30078086 PMC6097080

[342] Bird P, Nicholls D, Barrett R, et al. Longitudinal study of clinical prognostic factors in patients with early rheumatoid arthritis: the PREDICT study. Int J Rheum Dis 2017;20:460–8. 10.1111/1756-185X.13036 28205333

[343] D'Agostino M-A, Alten R, Mysler E, et al. Body mass index and clinical response to intravenous or subcutaneous abatacept in patients with rheumatoid arthritis. Clin Rheumatol 2017;36:2655–65. 10.1007/s10067-017-3788-1 28822046 PMC5681604

[344] George MD, Østergaard M, Conaghan PG, et al. Obesity and rates of clinical remission and low MRI inflammation in rheumatoid arthritis. Ann Rheum Dis 2017;76:1743–6. 10.1136/annrheumdis-2017-211569 28606966

[345] Iannone F, Courvoisier DS, Gottenberg JE, et al. Body mass does not impact the clinical response to intravenous abatacept in patients with rheumatoid arthritis. Analysis from the "pan-European registry collaboration for abatacept (PANABA). Clin Rheumatol 2017;36:773–9. 10.1007/s10067-016-3505-5 27966068

[346] Joo YB, Bang S-Y, Ryu JA, et al. Predictors of severe radiographic progression in patients with early rheumatoid arthritis: a prospective observational cohort study. Int J Rheum Dis 2017;20:1437–46. 10.1111/1756-185X.13054 28261973

[347] Levitsky A, Brismar K, Hafström I, et al. Obesity is a strong predictor of worse clinical outcomes and treatment responses in early rheumatoid arthritis: results from the SWEFOT trial. RMD Open 2017;3:e000458. 10.1136/rmdopen-2017-000458 28879052 PMC5574420

[348] Mariette X, Alten R, Nüßlein HG, et al. The effect of body mass index on clinical response to abatacept as a first-line biologic for rheumatoid arthritis: 6-month results from the 2-year, observational, prospective ACTION study. Joint Bone Spine 2017;84:571–6. 10.1016/j.jbspin.2016.10.011 28043761

[349] Miwa Y, Saito M, Furuya H, et al. Clinical characteristics of rheumatoid arthritis patients achieving functional remission after six months of non-tumor necrosis factor biological disease-modifying antirheumatic drugs (DMARDs) treatment. Intern Med 2017;56:2271–5. 10.2169/internalmedicine.8723-16 28794381 PMC5635297

[350] Ramírez J, Narváez JA, Ruiz-Esquide V, et al. Clinical and sonographic biomarkers of structural damage progression in RA patients in clinical remission: a prospective study with 12 months follow-up. Semin Arthritis Rheum 2017;47:303–9. 10.1016/j.semarthrit.2017.04.007 28549731

[351] Feldthusen C, Grimby-Ekman A, Forsblad-d'Elia H, et al. Explanatory factors and predictors of fatigue in persons with rheumatoid arthritis: a longitudinal study. J Rehabil Med 2016;48:469–76. 10.2340/16501977-2090 27097684

[352] Gardette A, Ottaviani S, Sellam J, et al. Body mass index and response to abatacept in rheumatoid arthritis. Eur J Clin Invest 2016;46:1048–52. 10.1111/eci.12691 27736006

[353] Gardette A, Ottaviani S, Sellam J, et al. Body mass index and response to tocilizumab in rheumatoid arthritis: a real life study. Clin Rheumatol 2016;35:857–61. 10.1007/s10067-016-3183-3 26801332

[354] McWilliams DF, Walsh DA. Factors predicting pain and early discontinuation of tumour necrosis factor-α-inhibitors in people with rheumatoid arthritis: results from the British Society for rheumatology biologics register. BMC Musculoskelet Disord 2016;17:337. 10.1186/s12891-016-1192-7 27515300 PMC4982340

[355] Tantayakom P, Koolvisoot A, Arromdee E, et al. Metabolic syndrome is associated with disease activity in patients with rheumatoid arthritis. Joint Bone Spine 2016;83:563–7. 10.1016/j.jbspin.2015.10.016 27238198

[356] Baker JF, Billig E, Michaud K, et al. Weight loss, the obesity paradox, and the risk of death in rheumatoid arthritis. Arthritis Rheumatol 2015;67:1711–7. 10.1002/art.39136 25940140 PMC4826750

[357] Iannone F, Fanizzi R, Notarnicola A, et al. Obesity reduces the drug survival of second line biological drugs following a first TNF-α inhibitor in rheumatoid arthritis patients. Joint Bone Spine 2015;82:187–91. 10.1016/j.jbspin.2014.12.006 25619156

[358] Pers Y-M, Godfrin-Valnet M, Lambert J, et al. Response to tocilizumab in rheumatoid arthritis is not influenced by the body mass index of the patient. J Rheumatol 2015;42:580–4. 10.3899/jrheum.140673 25641885

[359] Kim HW, Park JK, Yang J-A, et al. Comparison of tuberculosis incidence in ankylosing spondylitis and rheumatoid arthritis during tumor necrosis factor inhibitor treatment in an intermediate burden area. Clin Rheumatol 2014;33:1307–12. 10.1007/s10067-013-2387-z 24057090

[360] Ochi K, Go Y, Furuya T, et al. Risk factors associated with the occurrence of distal radius fractures in Japanese patients with rheumatoid arthritis: a prospective observational cohort study. Clin Rheumatol 2014;33:477–83. 10.1007/s10067-013-2415-z 24196989

[361] Sandberg MEC, Bengtsson C, Källberg H, et al. Overweight decreases the chance of achieving good response and low disease activity in early rheumatoid arthritis. Ann Rheum Dis 2014;73:2029–33. 10.1136/annrheumdis-2013-205094 24818635

[362] Ajeganova S, Andersson ML, Hafström I, et al. Association of obesity with worse disease severity in rheumatoid arthritis as well as with comorbidities: a long-term followup from disease onset. Arthritis Care Res 2013;65:78–87. 10.1002/acr.21710 22514159

[363] Gremese E, Carletto A, Padovan M, et al. Obesity and reduction of the response rate to anti-tumor necrosis factor α in rheumatoid arthritis: an approach to a personalized medicine. Arthritis Care Res 2013;65:94–100. 10.1002/acr.21768 22730143

[364] Kanecki K, Tyszko P, Wisłowska M, et al. Preliminary report on a study of health-related quality of life in patients with rheumatoid arthritis. Rheumatol Int 2013;33:429–34. 10.1007/s00296-012-2421-5 22453529 PMC3557393

[365] Dirven L, Huizinga TWJ, Allaart CF. Risk factors for reported influenza and influenza-like symptoms in patients with rheumatoid arthritis. Scand J Rheumatol 2012;41:359–65. 10.3109/03009742.2012.670729 22813350

[366] Wevers-de Boer K, Visser K, Heimans L, et al. Remission induction therapy with methotrexate and prednisone in patients with early rheumatoid and undifferentiated arthritis (the IMPROVED study). Ann Rheum Dis 2012;71:1472–7. 10.1136/annrheumdis-2011-200736 22402145

[367] Wolfe F, Michaud K. Effect of body mass index on mortality and clinical status in rheumatoid arthritis. Arthritis Care Res 2012;64:1471–9. 10.1002/acr.21627 22514152

[368] de Rooy DPC, van der Linden MPM, Knevel R, et al. Predicting arthritis outcomes--what can be learned from the Leiden Early Arthritis Clinic? Rheumatology 2011;50:93–100. 10.1093/rheumatology/keq230 20639266

[369] Klaasen R, Wijbrandts CA, Gerlag DM, et al. Body mass index and clinical response to infliximab in rheumatoid arthritis. Arthritis Rheum 2011;63:359–64. 10.1002/art.30136 21279992

[370] Liao KP, Weinblatt ME, Cui J, et al. Clinical predictors of erosion-free status in rheumatoid arthritis: a prospective cohort study. Rheumatology 2011;50:1473–9. 10.1093/rheumatology/ker129 21447567 PMC3133482

[371] Tekaya R, Sahli H, Zribi S. [Obesity has a protective effect on radiographic joint damage in rheumatoid arthritis]. Tunis Med 2011;89. 21557184

[372] Pye SR, Marshall T, Gaffney K, et al. Influence of arthritis and non-arthritis related factors on areal bone mineral density (BMDa) in women with longstanding inflammatory polyarthritis: a primary care based inception cohort. BMC Musculoskelet Disord 2010;11:106. 10.1186/1471-2474-11-106 20509941 PMC2889849

[373] Verstappen SMM, Bakker MF, Heurkens AHM, et al. Adverse events and factors associated with toxicity in patients with early rheumatoid arthritis treated with methotrexate tight control therapy: the CAMERA study. Ann Rheum Dis 2010;69:1044–8. 10.1136/ard.2008.106617 19581281

[374] Furuya T, Yamagiwa K, Ikai T, et al. Associated factors for falls and fear of falling in Japanese patients with rheumatoid arthritis. Clin Rheumatol 2009;28:1325–30. 10.1007/s10067-009-1229-5 19618097

[375] Hashimoto J, Garnero P, van der Heijde D, et al. A combination of biochemical markers of cartilage and bone turnover, radiographic damage and body mass index to predict the progression of joint destruction in patients with rheumatoid arthritis treated with disease-modifying anti-rheumatic drugs. Mod Rheumatol 2009;19:273–82. 10.1007/s10165-009-0170-4 19452245

[376] van der Helm-van Mil AHM, van der Kooij SM, Allaart CF, et al. A high body mass index has a protective effect on the amount of joint destruction in small joints in early rheumatoid arthritis. Ann Rheum Dis 2008;67:769–74. 10.1136/ard.2007.078832 17965124

[377] Cohen J-D, Dougados M, Goupille P, et al. Health assessment questionnaire score is the best predictor of 5-year quality of life in early rheumatoid arthritis. J Rheumatol 2006;33:1936–41. 16924692

[378] Escalante A, Haas RW, del Rincón I. Paradoxical effect of body mass index on survival in rheumatoid arthritis: role of comorbidity and systemic inflammation. Arch Intern Med 2005;165:1624–9. 10.1001/archinte.165.14.1624 16043681

[379] Maradit-Kremers H, Nicola PJ, Crowson CS, et al. Cardiovascular death in rheumatoid arthritis: a population-based study. Arthritis Rheum 2005;52:722–32. 10.1002/art.20878 15751097

[380] Sköldstam L, Brudin L, Hagfors L, et al. Weight reduction is not a major reason for improvement in rheumatoid arthritis from lacto-vegetarian, vegan or Mediterranean diets. Nutr J 2005;4:15. 10.1186/1475-2891-4-15 15871736 PMC1156940

[381] Kremers HM, Nicola PJ, Crowson CS, et al. Prognostic importance of low body mass index in relation to cardiovascular mortality in rheumatoid arthritis. Arthritis Rheum 2004;50:3450–7. 10.1002/art.20612 15529378

[382] Hoekstra M, van Ede AE, Haagsma CJ, et al. Factors associated with toxicity, final dose, and efficacy of methotrexate in patients with rheumatoid arthritis. Ann Rheum Dis 2003;62:423–6. 10.1136/ard.62.5.423 12695153 PMC1754533

[383] Krishnan E, Lingala B, Bruce B, et al. Disability in rheumatoid arthritis in the era of biological treatments. Ann Rheum Dis 2012;71:213–8. 10.1136/annrheumdis-2011-200354 21953343

[384] Kreps DJ, Halperin F, Desai SP, et al. Association of weight loss with improved disease activity in patients with rheumatoid arthritis: a retrospective analysis using electronic medical record data. Int J Clin Rheumtol 2018;13:1–10. 10.4172/1758-4272.1000154 29606976 PMC5875117

[385] Mori S, Yoshitama T, Hidaka T, et al. Comparative risk of hospitalized infection between biological agents in rheumatoid arthritis patients: a multicenter retrospective cohort study in Japan. PLoS One 2017;12:e0179179. 10.1371/journal.pone.0179179 28594905 PMC5464634

[386] Rashid N, Lin AT, Aranda G, et al. Rates, factors, reasons, and economic impact associated with switching in rheumatoid arthritis patients newly initiated on biologic disease modifying anti-rheumatic drugs in an integrated healthcare system. J Med Econ 2016;19:568–75. 10.3111/13696998.2016.1142448 26766553

[387] Ottaviani S, Gardette A, Roy C, et al. Body mass index and response to rituximab in rheumatoid arthritis. Joint Bone Spine 2015;82:432–6. 10.1016/j.jbspin.2015.02.011 26184536

[388] Ottaviani S, Gardette A, Tubach F, et al. Body mass index and response to infliximab in rheumatoid arthritis. Clin Exp Rheumatol 2015;33:478–83. 25962513

[389] Sparks JA, Halperin F, Karlson JC, et al. Impact of bariatric surgery on patients with rheumatoid arthritis. Arthritis Care Res 2015;67:1619–26. 10.1002/acr.22629 PMC4662646 26018243

[390] Gonzalez A, Maradit Kremers H, Crowson CS, et al. Do cardiovascular risk factors confer the same risk for cardiovascular outcomes in rheumatoid arthritis patients as in non-rheumatoid arthritis patients? Ann Rheum Dis 2008;67:64–9. 10.1136/ard.2006.059980 17517756

[391] Kent PD, Luthra HS, Michet C. Risk factors for methotrexate-induced abnormal laboratory monitoring results in patients with rheumatoid arthritis. J Rheumatol 2004;31:1727–31. 15338491

[392] Figueiredo-Braga M, Cornaby C, Bernardes M, et al. Correlation between physical markers and psychiatric health in a Portuguese systemic lupus erythematosus cohort: the role of suffering in chronic autoimmune disease. PLoS One 2018;13:e0195579. 10.1371/journal.pone.0195579 29659589 PMC5901990

[393] Jacobs J, Korswagen L-A, Schilder AM, et al. Six-year follow-up study of bone mineral density in patients with systemic lupus erythematosus. Osteoporos Int 2013;24:1827–33. 10.1007/s00198-012-2157-9 23052940

[394] Katz P, Yazdany J, Julian L, et al. Impact of obesity on functioning among women with systemic lupus erythematosus. Arthritis Care Res 2011;63:1357–64. 10.1002/acr.20526 PMC3183275 21702085

[395] Chaiamnuay S, Bertoli AM, Fernández M, et al. The impact of increased body mass index on systemic lupus erythematosus: data from LUMINA, a multiethnic cohort (LUMINA XLVI) [corrected]. J Clin Rheumatol 2007;13:128–33. 10.1097/RHU.0b013e3180645865 17551377

[396] Chaiamnuay S, Bertoli AM, Roseman JM, et al. African-American and Hispanic ethnicities, renal involvement and obesity predispose to hypertension in systemic lupus erythematosus: results from LUMINA, a multiethnic cohort (LUMINAXLV). Ann Rheum Dis 2007;66:618–22. 10.1136/ard.2006.059311 17107981 PMC1954629

[397] Uaratanawong S, Deesomchok U, Hiransuttikul N, et al. Four years follow-up of bone mineral density change in premenopausal women with systemic lupus erythematosus. J Med Assoc Thai 2004;87:1374–9. 15825716

[398] Bruce IN, Gladman DD, Urowitz MB. Detection and modification of risk factors for coronary artery disease in patients with systemic lupus erythematosus: a quality improvement study. Clin Exp Rheumatol 1998;16:435–40. 9706424

[399] Petri M, Perez-Gutthann S, Spence D, et al. Risk factors for coronary artery disease in patients with systemic lupus erythematosus. Am J Med 1992;93:513–9. 10.1016/0002-9343(92)90578-Y 1442853

[400] Hernández-Breijo B, Plasencia-Rodríguez C, Navarro-Compán V, et al. Association between concomitant csDMARDs and clinical response to TNF inhibitors in overweight patients with axial spondyloarthritis. Arthritis Res Ther 2019;21:66. 10.1186/s13075-019-1849-3 30786913 PMC6383284

[401] Jeong H, Eun YH, Kim IY, et al. Effect of tumor necrosis factor α inhibitors on spinal radiographic progression in patients with ankylosing spondylitis. Int J Rheum Dis 2018;21:1098–105. 10.1111/1756-185X.13270 29611287

[402] Pedersen SJ, Weber U, Said-Nahal R. Structural progression rate decreases over time on serial radiography and magnetic resonance imaging of sacroiliac joints and spine in a five-year follow-up study of patients with ankylosing spondylitis treated with tumour necrosis factor inhibitor. Scand J Rheumatol 2018:1–13. 10.1080/03009742.2018.1506822 30422733

[403] Maas F, Arends S, Wink FR, et al. Ankylosing spondylitis patients at risk of poor radiographic outcome show diminishing spinal radiographic progression during long-term treatment with TNF-α inhibitors. PLoS One 2017;12:e0177231. 10.1371/journal.pone.0177231 28640818 PMC5480831

[404] Maas F, Spoorenberg A, van der Slik BPG, et al. Clinical risk factors for the presence and development of vertebral fractures in patients with ankylosing spondylitis. Arthritis Care Res 2017;69:694–702. 10.1002/acr.22980 27389998

[405] Micheroli R, Hebeisen M, Wildi LM, et al. Impact of obesity on the response to tumor necrosis factor inhibitors in axial spondyloarthritis. Arthritis Res Ther 2017;19:164. 10.1186/s13075-017-1372-3 28724442 PMC5518107

[406] Hwang J, Kim H-M, Jeong H, et al. Higher body mass index and anti-drug antibodies predict the discontinuation of anti-TNF agents in Korean patients with axial spondyloarthritis. Rev Bras Reumatol Engl Ed 2017;57:311–9. 10.1016/j.rbre.2016.11.009 28743358

[407] van Weely SFE, Kneepkens EL, Nurmohamed MT, et al. Continuous improvement of physical functioning in ankylosing spondylitis patients by tumor necrosis factor inhibitors: three-year followup and predictors. Arthritis Care Res 2016;68:1522–9. 10.1002/acr.22869 26881893

[408] Maas F, Spoorenberg A, Brouwer E, et al. Spinal radiographic progression in patients with ankylosing spondylitis treated with TNF-α blocking therapy: a prospective longitudinal observational cohort study. PLoS One 2015;10:e0122693. 10.1371/journal.pone.0122693 25879956 PMC4400173

[409] Gremese E, Bernardi S, Bonazza S, et al. Body weight, gender and response to TNF-α blockers in axial spondyloarthritis. Rheumatology 2014;53:875–81. 10.1093/rheumatology/ket433 24407233

[410] Ottaviani S, Allanore Y, Tubach F, et al. Body mass index influences the response to infliximab in ankylosing spondylitis. Arthritis Res Ther 2012;14:R115. 10.1186/ar3841 22584116 PMC3446492

[411] Di Minno MND, Peluso R, Iervolino S, et al. Weight loss and achievement of minimal disease activity in patients with psoriatic arthritis starting treatment with tumour necrosis factor α blockers. Ann Rheum Dis 2014;73:1157–62. 10.1136/annrheumdis-2012-202812 23771989 PMC4033114

[412] Klingberg E, Bilberg A, BjÃ¶rkman S, et al. Weight loss improves disease activity in patients with psoriatic arthritis and obesity: an interventional study. Arthritis Res Ther 2019;21. 10.1186/s13075-019-1810-5 PMC6330463 30635024

[413] Polachek A, Li S, Chandran V, et al. Clinical Enthesitis in a prospective longitudinal psoriatic arthritis cohort: incidence, prevalence, characteristics, and outcome. Arthritis Care Res 2017;69:1685–91. 10.1002/acr.23174 27998023

[414] Højgaard P, Glintborg B, Kristensen LE, et al. The influence of obesity on response to tumour necrosis factor-α inhibitors in psoriatic arthritis: results from the DANBIO and ICEBIO registries. Rheumatology 2016;55:2191–9. 10.1093/rheumatology/kew326 27651526

[415] Eder L, Thavaneswaran A, Chandran V, et al. Obesity is associated with a lower probability of achieving sustained minimal disease activity state among patients with psoriatic arthritis. Ann Rheum Dis 2015;74:813–7. 10.1136/annrheumdis-2013-204448 24431392

[416] Mease PJ, Collier DH, Saunders KC, et al. Comparative effectiveness of biologic monotherapy versus combination therapy for patients with psoriatic arthritis: results from the Corrona registry. RMD Open 2015;1:e000181. 10.1136/rmdopen-2015-000181 26819748 PMC4716450

[417] di Minno MND, Peluso R, Iervolino S, et al. Obesity and the prediction of minimal disease activity: a prospective study in psoriatic arthritis. Arthritis Care Res 2013;65:141–7. 10.1002/acr.21711 22514189

[418] Iannone F, Fanizzi R, Scioscia C, et al. Body mass does not affect the remission of psoriatic arthritis patients on anti-TNF-α therapy. Scand J Rheumatol 2013;42:41–4. 10.3109/03009742.2012.715186 22991950

[419] Haddad A, Thavaneswaran A, Toloza S, et al. Diffuse idiopathic skeletal hyperostosis in psoriatic arthritis. J Rheumatol 2013;40:1367–73. 10.3899/jrheum.121433 23772085

[420] Marini C, Formichi B, Bauleo C, et al. Survival protection by bodyweight in isolated scleroderma-related pulmonary artery hypertension. Intern Emerg Med 2016;11:941–52. 10.1007/s11739-016-1446-2 27052360

[421] Assassi S, Del Junco D, Sutter K, et al. Clinical and genetic factors predictive of mortality in early systemic sclerosis. Arthritis Rheum 2009;61:1403–11. 10.1002/art.24734 19790132 PMC2883167

[422] Nielsen SM, Bartels EM, Henriksen M, et al. Weight loss for overweight and obese individuals with gout: a systematic review of longitudinal studies. Ann Rheum Dis 2017;76:1870–82. 10.1136/annrheumdis-2017-211472 28866649 PMC5705854

[423] Dessein PH, Shipton EA, Stanwix AE, et al. Beneficial effects of weight loss associated with moderate calorie/carbohydrate restriction, and increased proportional intake of protein and unsaturated fat on serum urate and lipoprotein levels in gout: a pilot study. Ann Rheum Dis 2000;59:539–43. 10.1136/ard.59.7.539 10873964 PMC1753185

[424] Nguyen U-SDT, Zhang Y, Louie-Gao Q, et al. Obesity paradox in recurrent attacks of gout in observational studies: clarification and remedy. Arthritis Care Res 2017;69:561–6. 10.1002/acr.22954 PMC5179319 27331767

[425] Romero-Talamás H, Daigle CR, Aminian A, et al. The effect of bariatric surgery on gout: a comparative study. Surg Obes Relat Dis 2014;10:1161–5. 10.1016/j.soard.2014.02.025 24935177

[426] Su BY-J, Lai H-M, Chen C-J, et al. Ischemia heart disease and greater waist circumference are risk factors of renal function deterioration in male gout patients. Clin Rheumatol 2008;27:581–6. 10.1007/s10067-007-0750-7 18030516

[427] Abhishek A, Valdes AM, Zhang W, et al. Association of serum uric acid and disease duration with frequent gout attacks: a case-control study. Arthritis Care Res 2016;68:1573–7. 10.1002/acr.22855 26866719

[428] Alvarez-Nemegyei J, Cen-Pisté JC, Medina-Escobedo M, et al. Factors associated with musculoskeletal disability and chronic renal failure in clinically diagnosed primary gout. J Rheumatol 2005;32:1923–7. 16206348

[429] Gwinnutt JM, Verstappen SM, Humphreys JH. The impact of lifestyle behaviours, physical activity and smoking on morbidity and mortality in patients with rheumatoid arthritis. Best Pract Res Clin Rheumatol 2020;34:101562. 10.1016/j.berh.2020.101562 32646673

[430] Veldhuijzen van Zanten JJCS, Rouse PC, Hale ED, et al. Perceived barriers, facilitators and benefits for regular physical activity and exercise in patients with rheumatoid arthritis: a review of the literature. Sports Med 2015;45:1401–12. 10.1007/s40279-015-0363-2 26219268 PMC4579262

[431] Gwinnutt JM, Alsafar H, Hyrich KL, et al. Do people with rheumatoid arthritis maintain their physical activity level at treatment onset over the first year of methotrexate therapy? Rheumatology 2021;60:4633–42. 10.1093/rheumatology/keab060 33605404 PMC8487269

[432] Klein-Wieringa IR, van der Linden MPM, Knevel R, et al. Baseline serum adipokine levels predict radiographic progression in early rheumatoid arthritis. Arthritis Rheum 2011;63:2567–74. 10.1002/art.30449 21567382

[433] Shin A, Shin S, Kim JH, et al. Association between socioeconomic status and comorbidities among patients with rheumatoid arthritis: results of a nationwide cross-sectional survey. Rheumatology 2019;58:1617–22. 10.1093/rheumatology/kez081 30892622

[434] Björk M, Dragioti E, Alexandersson H, et al. Inflammatory arthritis and the effect of physical activity on quality of life and self-reported function: a systematic review and meta-analysis. Arthritis Care Res 2022;74:31–43. 10.1002/acr.24805 34632707

[435] Ortolan A, Lorenzin M, Felicetti M, et al. Do obesity and overweight influence disease activity measures in axial spondyloarthritis? A systematic review and meta-analysis. Arthritis Care Res 2021;73:1815–25. 10.1002/acr.24416 32799405

