# Supplementary material for: Effects of physical exercise and body weight on disease-specific outcomes of people with rheumatic and musculoskeletal diseases (RMDs): systematic reviews and meta-analyses informing the 2021 EULAR recommendations for lifestyle improvements in people with RMDs
Source: RMD Open. 2022 Mar 30;8(1):e002168. doi: 10.1136/rmdopen-2021-002168 (PMC8971792; doi:10.1136/rmdopen-2021-002168)
Supplement: Supplementary data [file rmdopen-2021-002168supp001.pdf]

Supplementary tables – exercise and weight review

Supplementary table 1 – Search strategy for systematic review of published reviews and meta-analyses

Category	Term
Diseases	1. Arthritis, Rheumatoid (mesh) (exp) (include all subheadings)<br>2. Inflammatory $arthritis<br>3. Undifferentiated arthritis<br>4. RA<br>5. Atrophic arthritis<br>6. Proliferative arthritis<br>7. Osteoarth$<br>8. Arthrosis<br>9. Degenerative joint disease<br>10. Hypertrophic arthritis<br>11. Arthropathy<br>12. Polyarthritis<br>13. OA<br>14. Arthritis psoriatica<br>15. Arthropathic psoriasis<br>16. Psoriatic arthropathy<br>17. Arthritis, Psoriatic (mesh) (exp) (include all subheadings)<br>18. Psoria$ arthriti$ [have to uncheck “map team to subject heading”]<br>19. Psoria$ arthropath$ [have to uncheck “map team to subject heading”]<br>20. Undifferentiated oligoarthritis<br>21. Arthritic psoriasis<br>22. PsA<br>23. Ankylosing spondylitis (mesh) (exp) (include all subheadings)<br>24. Ankylosi$<br>25. Spondyloarthr$ [have to uncheck “map team to subject heading”]<br>26. Spondylarthr$ [have to uncheck “map team to subject heading”]<br>27. Spondylitis (mesh) (exp) (include all subheadings)<br>28. Bechtere$ [have to uncheck “map team to subject heading”]<br>29. Marie-Strumpell<br>30. Spinal arthritis<br>31. Lupus erythematosus, systemic (mesh) (exp) (include all subheadings)<br>32. systemic lupus erythematosus<br>33. SLE<br>34. Libman-Sacks disease<br>35. Libman Sacks disease<br>36. Lupus erythematosus disseminatus<br>37. Disseminated lupus erythematosus<br>38. Lupus syndrome<br>39. Scleroderma, Systemic (mesh) (exp) (include all subheadings)<br>40. SSc<br>~~41. Scleros$~~ (removed because of ALS, multiple sclerosis etc.)<br>42. Thibierge-Weissenbach syndrome<br>43. Morphea<br>44. Gout (mesh) (exp) (include all subheadings)

	45. Gout$<br>46. Podagra<br>47. Tophus<br>48. Tophi<br>49. Tophaceous<br>50. Urate<br>51. Uric acid<br>52. Hyperurecemi$ [have to uncheck “map team to subject heading”]<br>53. Hyperurecaemi$ [have to uncheck “map team to subject heading”]<br>54. Hyperuricemia$<br>55. Hyperuricaemi$ [have to uncheck “map team to subject heading”]<br>56. arthritis urica<br>57. Gout acute
Life-style exposures	58. Diet (mesh) (exp) (include all subheadings)<br>59. Nutrition<br>60. Food (mesh) (exp) (include all subheadings)<br>61. Food habit$<br>62. Nutritional status (mesh) (exp) (include all subheadings)<br>63. Vitamin$ (mesh) (exp) (include all subheadings)<br>64. Antioxidant$ (mesh) (exp) (include all subheadings)<br>65. Fatty acid$ (mesh) (exp) (include all subheadings)<br>66. Carbohydrate$ (mesh) (exp) (include all subheadings)<br>67. Diet$ protein<br>68. Calcium<br>69. Fish oil$ (mesh) (exp) (include all subheadings)<br>70. Fruit (mesh) (exp) (include all subheadings)<br>71. Vegetable$ (mesh) (exp) (include all subheadings)<br>72. Micronutrient$ (mesh) (exp) (include all subheadings)<br>73. Nutriment$<br>74. Neutraceutical$<br>75. Exercis$<br>76. Strength$<br>77. Endurance<br>78. Cardiorespiratory<br>79. Aerobic<br>80. Aerobic training<br>81. Exercise program$<br>82. Exercise therap$ [have to uncheck “map team to subject heading”]<br>83. Physical education<br>84. Physical training<br>85. Physical therapy<br>86. Physiotherapy<br>87. Muscle stretching<br>88. Sport (mesh) (exp) (include all subheadings)<br>89. Bod$y Weight (mesh) (exp) (include all subheadings)<br>90. Weight change<br>91. Weight loss (mesh) (exp) (include all subheadings)<br>92. Weight reduction<br>93. Weight gain<br>94. Anti obesity<br>95. Anti-obesity

	96. Antiobesity<br>97. Slimming<br>98. Smok$<br>99. Smoking (mesh) (exp) (include all subheadings)<br>100. Tobacco (mesh) (exp) (include all subheadings)<br>101. Cigarette$<br>102. Pipe$<br>103. Cigar$<br>104. Nicotine (mesh) (exp) (include all subheadings)<br>105. Water pipe<br>106. Hookah<br>107. Shisha<br>108. Paid work<br>109. Employment (mesh) (exp) (include all subheadings)<br>110. Work$ disability<br>111. Productivity<br>112. Employability<br>113. Work$ ability<br>114. Absenteeism (mesh) (exp) (include all subheadings)<br>115. Sick leave (mesh) (exp) (include all subheadings)<br>116. Presenteeism (mesh) (exp) (include all subheadings)<br>117. Sick$ absence<br>118. Work instability<br>119. Return to work (mesh) (exp) (include all subheadings)<br>120. Economic consequences<br>121. Occupational health<br>122. Labo$r
Systematic review terms	123. Systematic adj5 review<br>124. Narrative review<br>125. Meta-analysis (mesh) (exp)<br>126. Meta analysis<br>127. Meta adj5 analysis<br>128. Meta-synthesis<br>129. Meta synthesis<br>130. Meta adj5 synthesis<br>131. Literature review<br>132. Literature search<br>133. Meta-narrative review<br>134. Meta narrative review
Combining terms	135. RA – 1 OR 2 OR 3 OR 4 OR 5 OR 6<br>136. OA – 7 OR 8 OR 9 OR 10 OR 11 OR 12 OR 13<br>137. PSA – 14 OR 15 OR 16 OR 17 OR 18 OR 19 OR 20 OR 21 OR 22<br>138. AS – 23 OR 24 OR 25 OR 26 OR 27 OR 28 OR 29 OR 30<br>139. SLE – 31 OR 32 OR 33 OR 34 OR 35 OR 36 OR 37 OR 38<br>140. SSc – 39 OR 40 OR 41 OR 42 OR 43<br>141. Gout – 44 OR 45 OR 46 OR 47 OR 48 OR 49 OR 50 OR 51 OR 52 OR 53 OR 54 OR 55 OR 56 OR 57<br>142. Diseases – 136 OR 137 OR 138 OR 139 OR 140 OR 141 OR 142<br>143. Diet – 58 OR 59 OR 60 OR 61 OR 62 OR 63 OR 64 OR 65 OR 66 OR 67 OR 68 OR 69 OR 70 OR 71 OR 72 OR 73 OR 74

	144. Exercise – 75 OR 76 OR 77 OR 78 OR 79 OR 80 OR 81 OR 82 OR 83 OR 84 OR 85 OR 86 OR 87 OR 88<br>145. Weight – 89 OR 90 OR 91 OR 92 OR 93 OR 94 OR 95 OR 96 OR 97<br>146. Smoking - 98 OR 99 OR 100 OR 101 OR 102 OR 103 OR 104 OR 105 OR 106 OR 107<br>147. Work – 108 OR 109 OR 110 OR 111 OR 112 OR 113 OR 114 OR 115 OR 116 OR 117 OR 118 OR 119 OR 120 OR 121 OR 122<br>148. Exposures – 144 OR 145 OR 146 OR 147 OR 148<br>149. Systematic review terms - 123 OR 124 OR 125 OR 126 OR 127 OR 128 OR 129 OR 130 OR 131 OR 132 OR 133 OR 134 OR 135<br>150. 143 AND 149 AND 150

Supplementary table 2 – Search strategy to identify published systematic reviews and meta-analyses on alcohol

The results from the first review of published systematic reviews and meta-analyses (supplementary table 1) were presented at a teleconference in January 2019. At this teleconference, it was decided to add alcohol as an exposure of interest for this taskforce. This led to a second systematic review of published reviews and meta-analyses. For completeness, the search strategy for this review is below. The results from this review are not reported in this systematic review on exercise and weight; they are published in a separate review on smoking and alcohol. However, these studies are included in the flow chart of figure 1, hence the inclusion of the search strategy here.

Category	Term
Disease	1. Arthritis, Rheumatoid (mesh) (exp) (include all subheadings)<br>2. Inflammatory $arthritis<br>3. Undifferentiated arthritis<br>4. RA<br>5. Atrophic arthritis<br>6. Proliferative arthritis<br>7. Osteoarth$<br>8. Arthrosis<br>9. Degenerative joint disease<br>10. Hypertrophic arthritis<br>11. Arthropathy<br>12. Polyarthritis<br>13. OA<br>14. Arthritis psoriatica<br>15. Arthropathic psoriasis<br>16. Psoriatic arthropathy<br>17. Arthritis, Psoriatic (mesh) (exp) (include all subheadings)<br>18. Psoria$ arthriti$ [have to uncheck “map team to subject heading”]<br>19. Psoria$ arthropath$ [have to uncheck “map team to subject heading”]<br>20. Undifferentiated oligoarthritis<br>21. Arthritic psoriasis<br>22. PsA<br>23. Ankylosing spondylitis (mesh) (exp) (include all subheadings)<br>24. Ankylosi$<br>25. Spondyloarthr$ [have to uncheck “map team to subject heading”]<br>26. Spondylarthr$ [have to uncheck “map team to subject heading”]<br>27. Spondylitis (mesh) (exp) (include all subheadings)<br>28. Bechtere$ [have to uncheck “map team to subject heading”]<br>29. Marie-Strumpell<br>30. Spinal arthritis<br>31. Lupus erythematosus, systemic (mesh) (exp) (include all subheadings)<br>32. systemic lupus erythematosus<br>33. SLE<br>34. Libman-Sacks disease<br>35. Libman Sacks disease<br>36. Lupus erythematosus disseminatus<br>37. Disseminated lupus erythematosus<br>38. Lupus syndrome

	39. Scleroderma, Systemic (mesh) (exp) (include all subheadings)<br>40. SSc<br>41. Thibierge-Weissenbach syndrome<br>42. Morphea<br>43. Gout (mesh) (exp) (include all subheadings)<br>44. Gout$<br>45. Podagra<br>46. Tophus<br>47. Tophi<br>48. Tophaceous<br>49. Urate<br>50. Uric acid<br>51. Hyperurecemi$ [have to uncheck “map team to subject heading”]<br>52. Hyperurecaemi$ [have to uncheck “map team to subject heading”]<br>53. Hyperuricemia$<br>54. Hyperuricaemi$ [have to uncheck “map team to subject heading”]<br>55. arthritis urica<br>56. Gout acute
Exposure	57. Alcohol<br>58. Ethanol<br>59. Beer<br>60. Wine<br>61. Spirit$<br>62. liquor
Systematic review terms	63. Systematic adj5 review<br>64. Narrative review<br>65. Meta-analysis (mesh) (exp)<br>66. Meta analysis<br>67. Meta adj5 analysis<br>68. Meta-synthesis<br>69. Meta synthesis<br>70. Meta adj5 synthesis<br>71. Literature review<br>72. Literature search<br>73. Meta-narrative review<br>74. Meta narrative review
Combining terms	75. RA – 1 OR 2 OR 3 OR 4 OR 5 OR 6<br>76. OA – 7 OR 8 OR 9 OR 10 OR 11 OR 12 OR 13<br>77. PSA – 14 OR 15 OR 16 OR 17 OR 18 OR 19 OR 20 OR 21 OR 22<br>78. AS – 23 OR 24 OR 25 OR 26 OR 27 OR 28 OR 29 OR 30<br>79. SLE – 31 OR 32 OR 33 OR 34 OR 35 OR 36 OR 37 OR 38<br>80. SSc – 39 OR 40 OR 41 OR 42<br>81. Gout – 43 OR 44 OR 45 OR 46 OR 47 OR 48 OR 49 OR 50 OR 51 OR 52 OR 53 OR 54 OR 55 OR 56<br>82. Alcohol – 57 OR 58 OR 59 OR 60 OR 61 OR 62<br>83. Systematic review terms - 63 OR 64 OR 65 OR 66 OR 67 OR 68 OR 69 OR 70 OR 71 OR 72 OR 73 OR 74<br>84. Disease – 75 OR 76 OR 77 OR 78 OR 79 OR 80 OR 81<br>85. 82 AND 83 AND 84

Supplementary table 3 – Search strategy for systematic review of original articles focusing on exercise in RMDs

Category	Term
Diseases	1. Arthritis, Rheumatoid (mesh) (exp) (include all subheadings)<br>2. Inflammatory $arthritis<br>3. Undifferentiated arthritis<br>4. RA<br>5. Atrophic arthritis<br>6. Proliferative arthritis<br>7. Arthritis psoriatica<br>8. Arthropathic psoriasis<br>9. Psoriatic arthropathy<br>10. Arthritis, Psoriatic (mesh) (exp) (include all subheadings)<br>11. Psoria$ arthriti$ [have to uncheck "map team to subject heading"]<br>12. Psoria$ arthropath$ [have to uncheck "map team to subject heading"]<br>13. Undifferentiated oligoarthritis<br>14. Arthritic psoriasis<br>15. PsA<br>16. Ankylosing spondylitis (mesh) (exp) (include all subheadings)<br>17. Ankylosi$<br>18. Spondyloarthr$ [have to uncheck "map team to subject heading"]<br>19. Spondylarthr$ [have to uncheck "map team to subject heading"]<br>20. Spondylitis (mesh) (exp) (include all subheadings)<br>21. Bechtere$ [have to uncheck "map team to subject heading"]<br>22. Marie-Strumpell<br>23. Spinal arthritis<br>24. Lupus erythematosus, systemic (mesh) (exp) (include all subheadings)<br>25. systemic lupus erythematosus<br>26. SLE<br>27. Libman-Sacks disease<br>28. Libman Sacks disease<br>29. Lupus erythematosus disseminatus<br>30. Disseminated lupus erythematosus<br>31. Lupus syndrome<br>32. Scleroderma, Systemic (mesh) (exp) (include all subheadings)<br>33. SSc<br>34. Thibierge-Weissenbach syndrome<br>35. Morphea<br>36. Gout (mesh) (exp) (include all subheadings)<br>37. Gout$<br>38. Podagra<br>39. Tophus<br>40. Tophi

	41. Tophaceous<br>42. Urate<br>43. Uric acid<br>44. Hyperurecemi$ [have to uncheck “map team to subject heading”]<br>45. Hyperurecaemi$ [have to uncheck “map team to subject heading”]<br>46. Hyperuricemia$<br>47. Hyperuricaemi$ [have to uncheck “map team to subject heading”]<br>48. arthritis urica<br>49. Gout acute
Exercise	50. Exercis$<br>51. Strength$<br>52. Endurance<br>53. Cardiorespiratory<br>54. Aerobic<br>55. Aerobic training<br>56. Exercise program$<br>57. Exercise therap$ [have to uncheck “map team to subject heading”]<br>58. Physical education<br>59. Physical training<br>60. Physical therapy<br>61. Physiotherapy<br>62. Muscle stretching<br>63. Sport (mesh) (exp) (include all subheadings)<br>64. Resistance<br>65. Aquatic<br>66. Yoga<br>67. Tai-chi<br>68. Tai chi<br>69. Exercise therapy<br>70. Fitness<br>71. Running<br>72. Cycling<br>73. Sprinting<br>74. Jogging<br>75. Stretching
Exclusions	76. Cross-sectional<br>77. Cross sectional<br>78. Children<br>79. Child<br>80. Juvenile<br>81. Animal<br>82. Rat<br>83. rats

	84. Mouse<br>85. Case study<br>86. Case series<br>87. Systematic adj5 review<br>88. Narrative review<br>89. Meta-analysis (mesh) (exp)<br>90. Meta analysis<br>91. Meta adj5 analysis<br>92. Meta-synthesis<br>93. Meta synthesis<br>94. Meta adj5 synthesis<br>95. Literature review<br>96. Literature search<br>97. Meta-narrative review<br>98. Meta narrative review<br>99. Vascular resistance<br>100. Vascular resistance [mesh]
Study design terms	101. Randomised controlled trial<br>102. Randomised control trial<br>103. Randomized controlled trial (mesh) (exp)<br>104. Randomized control trial<br>105. RCT<br>106. Clinical trial (mesh) (exp)<br>107. Blind$<br>108. Cohort studies (mesh) (exp)<br>109. Observational stud$<br>110. Case-control studies (mesh) (exp)<br>111. Intervention stud$<br>112. Interventional stud$<br>113. Open label<br>114. Longitudinal studies (mesh) (exp)<br>115. Follow-up<br>116. Follow up<br>117. Prospectiv$<br>118. Retrospectiv$<br>119. Cohort$
Combining terms	120. RA – 1 OR 2 OR 3 OR 4 OR 5 OR 6<br>121. PSA – 7 OR 8 OR 9 OR 10 OR 11 OR 12 OR 13 OR 14 OR 15<br>122. AS – 16 OR 17 OR 18 OR 19 OR 20 OR 21 OR 22 OR 23<br>123. SLE – 24 OR 25 OR 26 OR 27 OR 28 OR 29 OR 30 OR 31<br>124. SSc – 32 OR 33 OR 34 OR 35<br>125. Gout – 36 OR 37 OR 38 OR 39 OR 40 OR 41 OR 42 OR 43 OR 44 OR 45 OR 46 OR 47 OR 48 OR 49

	126. Diseases – 120 OR 121 OR 122 OR 123 OR 124 OR 125<br>127. Exercise – 50 OR 51 OR 52 OR 53 OR 54 OR 55 OR 56 OR 57 OR 58 OR 59 OR 60 OR 61 OR 62 OR 63 OR 64 OR 65 OR 66 OR 67 OR 68 OR 69 OR 70 OR 71 OR 72 OR 73 OR 74 OR 75<br>128. Exclusions – 76 OR 77 OR 78 OR 79 OR 80 OR 81 OR 82 OR 83 OR 84 OR 85 OR 86 OR 87 OR 88 OR 89 OR 90 OR 91 OR 92 OR 93 OR 94 OR 95 OR 96 OR 97 OR 98 OR 99 O2 100<br>129. Study design terms – 101 OR 102 OR 103 OR 104 OR 105 OR 106 OR 107 OR 108 OR 109 OR 110 OR 111 OR 112 OR 113 OR 114 OR 115 OR 116 OR 117 OR 118 OR 119<br>130. 126 AND 127 AND 129<br>131. 130 NOT 128

Supplementary table 4 – Search strategy for systematic review of original articles focusing on weight in RMDs

Category	Term
Diseases	1. Arthritis, Rheumatoid (mesh) (exp) (include all subheadings)<br>2. Inflammatory $arthritis<br>3. Undifferentiated arthritis<br>4. RA<br>5. Atrophic arthritis<br>6. Proliferative arthritis<br>7. Osteoarth$<br>8. Arthrosis<br>9. Degenerative joint disease<br>10. Hypertrophic arthritis<br>11. Arthropathy<br>12. Polyarthritis<br>13. OA<br>14. Arthritis psoriatica<br>15. Arthropathic psoriasis<br>16. Psoriatic arthropathy<br>17. Arthritis, Psoriatic (mesh) (exp) (include all subheadings)<br>18. Psoria$ arthriti$ [have to uncheck “map team to subject heading”]<br>19. Psoria$ arthropath$ [have to uncheck “map team to subject heading”]<br>20. Undifferentiated oligoarthritis<br>21. Arthritic psoriasis<br>22. PsA<br>23. Ankylosing spondylitis (mesh) (exp) (include all subheadings)<br>24. Ankylosi$<br>25. Spondyloarthr$ [have to uncheck “map team to subject heading”]<br>26. Spondylarthr$ [have to uncheck “map team to subject heading”]<br>27. Spondylitis (mesh) (exp) (include all subheadings)<br>28. Bechtere$ [have to uncheck “map team to subject heading”]<br>29. Marie-Strumpell<br>30. Spinal arthritis<br>31. Lupus erythematosus, systemic (mesh) (exp) (include all subheadings)<br>32. systemic lupus erythematosus<br>33. SLE<br>34. Libman-Sacks disease<br>35. Libman Sacks disease<br>36. Lupus erythematosus disseminatus<br>37. Disseminated lupus erythematosus<br>38. Lupus syndrome<br>39. Scleroderma, Systemic (mesh) (exp) (include all subheadings)

	40. SSc<br>41. Thibierge-Weissenbach syndrome<br>42. Morphea<br>43. Gout (mesh) (exp) (include all subheadings)<br>44. Gout$<br>45. Podagra<br>46. Tophus<br>47. Tophi<br>48. Tophaceous<br>49. Urate<br>50. Uric acid<br>51. Hyperurecemi$ [have to uncheck "map team to subject heading"]<br>52. Hyperurecaemi$ [have to uncheck "map team to subject heading"]<br>53. Hyperuricemia$<br>54. Hyperuricaemi$ [have to uncheck "map team to subject heading"]<br>55. arthritis urica<br>56. Gout acute
Weight	57. Bod$y Weight (mesh) (exp) (include all subheadings)<br>58. Weight change<br>59. Weight loss (mesh) (exp) (include all subheadings)<br>60. Weight reduction<br>61. Weight gain<br>62. obesity<br>63. Anti obesity<br>64. Anti-obesity<br>65. Antiobesity<br>66. Slimming<br>67. BMI<br>68. Body mass index<br>69. Adiposity<br>70. Body adiposity index<br>71. Weight control<br>72. Total body mass<br>73. Bariatric
Exclusions	74. Cross-sectional<br>75. Cross sectional<br>76. Children<br>77. Child<br>78. Juvenile<br>79. Adolescent<br>80. Teenager<br>81. Animal<br>82. Rat

	83. rats<br>84. Mouse<br>85. mice<br>86. Case study<br>87. Case series<br>88. Systematic adj5 review<br>89. Narrative review<br>90. Meta-analysis (mesh) (exp)<br>91. Meta analysis<br>92. Meta adj5 analysis<br>93. Meta-synthesis<br>94. Meta synthesis<br>95. Meta adj5 synthesis<br>96. Literature review<br>97. Literature search<br>98. Meta-narrative review<br>99. Meta narrative review<br>100. Prostate cancer<br>101. Prostatic neoplasms (mesh) (exp)<br>102. Infectious arthritis<br>103. Arthroplasty<br>104. Total hip replacement<br>105. Total knee replacement
Study design terms	106. Randomised controlled trial<br>107. Randomised control trial<br>108. Randomized controlled trial (mesh) (exp)<br>109. Randomized control trial<br>110. RCT<br>111. Clinical trial (mesh) (exp)<br>112. Blind$<br>113. Cohort studies (mesh) (exp)<br>114. Observational stud$<br>115. Case-control studies (mesh) (exp)<br>116. Intervention stud$<br>117. Interventional stud$<br>118. Open label<br>119. Longitudinal studies (mesh) (exp)<br>120. Follow-up<br>121. Follow up<br>122. Prospectiv$<br>123. Retrospectiv$<br>124. Cohort$

Combining terms	125. RA – 1 OR 2 OR 3 OR 4 OR 5 OR 6<br>126. OA – 7 OR 8 OR 9 OR 10 OR 11 OR 12 OR 13<br>127. PSA – 14 OR 15 OR 16 OR 17 OR 18 OR 19 OR 20 OR 21 OR 22<br>128. AS – 23 OR 24 OR 25 OR 26 OR 27 OR 28 OR 29 OR 30<br>129. SLE – 31 OR 32 OR 33 OR 34 OR 35 OR 36 OR 37 OR 38<br>130. SSc – 39 OR 40 OR 41 OR 42<br>131. Gout – 43 OR 44 OR 45 OR 46 OR 47 OR 48 OR 49 OR 50 OR 51 OR 52 OR 53 OR 54 OR 55 OR 56<br>132. Diseases –125 OR 126 OR 127 OR 128 OR 129 OR 130 OR 131<br>133. Weight – 57 OR 59 OR 60 OR 61 OR 62 OR 63 OR 64 OR 65 OR 66 OR 67 OR 68 OR 69 OR 70 OR 71 OR 72 OR 73<br>134. Exclusions – 74 OR 75 OR 76 OR 77 OR 78 OR 79 OR 80 OR 81 OR 82 OR 83 OR 84 OR 85 OR 86 OR 87 OR 88 OR 89 OR 90 OR 91 OR 92 OR 93 OR 94 OR 95 OR 96 OR 97 OR 98 OR 99 OR 100 OR 101 OR 102 OR 103 OR 104 OR 105<br>135. Study design terms – 106 OR 107 OR 108 OR 109 OR 110 OR 111 OR 112 OR 113 OR 114 OR 115 OR 116 OR 117 OR 118 OR 119 OR 120 OR 121 OR 122 OR 123 OR 124<br>136. 132 AND 133 AND 135<br>137. 136 NOT 134

Supplementary table 5 – Included outcomes and examples of measures used to assess these outcomes

- Disease activity
  - OA
    - Western Ontario and McMaster Universities Arthritis Index [WOMAC]
  - RA
    - Acute phase reactants (i.e. C-reactive protein and erythrocyte sedimentation rate)
    - Swollen joint count
    - Tender joint count
    - Physician global assessment of disease activity (VAS)
    - Patient global health (VAS)
    - Disease activity composite measures (eg. Disease Activity Score [DAS28, DAS44], Rheumatoid arthritis Impact of Disease Score [RAID])
  - PsA [1]
    - Acute phase reactants (i.e. C-reactive protein and erythrocyte sedimentation rate)
    - Swollen joint count
    - Tender joint count
    - Physician global assessment of disease activity (VAS)
    - Patient global assessment of disease activity (VAS)
    - Dactylitis (e.g. Leeds dactylitis index)
    - Enthesitis (e.g. Mander/Newcastle Enthesitis Index, Leeds Enthesitis index)
    - Extent of psoriasis (e.g. Psoriasis Area and Severity Index [PASI])
    - Nail involvement (e.g. Nail Psoriasis Severity Index)
    - Disease activity composite measures (e.g. Composite Psoriatic Disease Activity Index [CPDAI], Disease Activity in Psoriatic Arthritis [DAPSA], clinical Disease Activity in Psoriatic Arthritis [cDAPSA], PsA Impact of Disease Score [PsAID] Psoriatic Arthritis Disease Activity Score [PASDAS])
  - AS [2]
    - Acute phase reactants (i.e. C-reactive protein and erythrocyte sedimentation rate)
    - Swollen joint count
    - Tender joint count
    - Disease activity composite measures (e.g. Ankylosing Spondylitis Disease Activity Score [ASDAS], Bath Ankylosing Spondylitis Disease Activity Index [BASDAI], Disease Activity Score [DAS44])
    - Enthesitis
    - Spinal mobility (e.g. Bath Ankylosing Spondylitis Metrology Index [BASMI])
    - Stiffness
  - SLE [3]
    - Disease activity composite measures (e.g. British Isles Lupus Assessment Group measure [BILAG], Systemic Lupus Erythematosus Disease Activity Index [SLEDAI])
    - Organ damage measures (e.g. Systemic Lupus International Collaborating Clinics (SLICC)/American College of Rheumatology Damage Index [SDI])
  - SSc [4]
    - Skin (e.g. Modified Rodnan skin score, visual analogue scale [VAS]/likert scale, Durometer reading)

        - Musculoskeletal (e.g. tender joint count, tender friction rubs assessed by doctor, serum creatinine)
        - Cardiac / pulmonary / renal / gastrointestinal involvement
        - Raynaud's phenomenon (e.g. Raynaud condition score, VAS raynauds)
        - Digital ulcers (e.g. activity digital tip ulcer count on volar surface, VAS digital ulcer)
        - Acute phase reactants (i.e. C-reactive protein and erythrocyte sedimentation rate)
    - Gout [5]
        - Serum urate
        - Gout flare recurrence
        - Tophus regression [6] / tophi number
        - Joint inflammation / tenderness score
- Physical functioning
    - OA
        - Physical function (e.g. the Knee Injury and Osteoarthritis Outcome Score [KOOS], Veterans Short Form 12 Health Survey [VR-12], Hip disability and Osteoarthritis Outcome Score [HOOS], WOMAC).
        - Objective measures (e.g. gait speed, grip strength)
        - Range of motion of effected joint
    - RA
        - Physical function (e.g. the Health Assessment Questionnaire [HAQ], Arthritis Impact Measurement Scale [AIMS], SF36-physical function)
        - Objective measures (e.g. gait speed, grip strength)
    - PsA
        - Physical function (e.g. the HAQ, Arthritis Impact Measurement Scale [AIMS], SF36-physical function)
        - Objective measures (e.g. gait speed, grip strength)
    - AS
        - Physical function (e.g. Health Assessment Questionnaire for the Spondylarthropathies [HAQ-S], Dougados Functional Index [DFI], Bath Ankylosing Spondyltitis Functional Index [BASFI])
        - Objective measures (e.g. gait speed, grip strength)
    - SLE [7]
        - Physical function (e.g. the HAQ, SF-36 physical function, Valued Life Activities Disability Scale)
        - Objective measures (e.g. gait speed, grip strength)
    - SSc
        - Physical function (e.g. the HAQ, SF-36).
        - Objective measures (e.g. gait speed, grip strength)
    - Gout
        - Physical function (e.g. HAQ [5;8], SF-36)
        - Objective measures (e.g. gait speed, grip strength)
- Pain
    - OA [9]
        - OARSI-OMERACT Initiative: New OA Pain Measure
        - Dallas Pain Questionnaire
        - Neck Pain and Disability Scale [NPAD]
        - WOMAC
        - Australian/Canadian Hand OA Index (AUSCAN)
    - RA

        - Patient pain rating (e.g. visual analogue scale)
    - PSA
        - Patient pain rating (e.g. visual analogue scale)
    - AS
        - Patient pain rating (e.g. visual analogue scale)
    - SLE
        - Patient pain rating (e.g. visual analogue scale)
    - SSc
        - Patient pain rating (e.g. visual analogue scale)
    - Gout
        - Patient pain rating (e.g. visual analogue scale / likert scale) [10]
- Fatigue
    - OA
        - Patient fatigue rating (e.g. visual analogue scale, other disease specific measure)
        - Generic fatigue questionnaire (e.g. Chalder Fatigue Scale)
    - RA
        - Patient fatigue rating (e.g. visual analogue scale, other disease specific measure)
        - Generic fatigue questionnaire (e.g. Chalder Fatigue Scale)
        - Bristol Rheumatoid Arthritis Fatigue – multidimensional questionnaire (BRAF-MDQ)
    - PSA
        - Patient fatigue rating (e.g. visual analogue scale, other disease specific measure)
        - Generic fatigue questionnaire (e.g. Chalder Fatigue Scale)
    - AS
        - Patient fatigue rating (e.g. visual analogue scale, other disease specific measure)
        - Generic fatigue questionnaire (e.g. Chalder Fatigue Scale)
    - SLE
        - Patient fatigue rating (e.g. visual analogue scale, other disease specific measure)
        - Generic fatigue questionnaire (e.g. Chalder Fatigue Scale)
    - SSc
        - Patient fatigue rating (e.g. visual analogue scale, other disease specific measure)
        - Generic fatigue questionnaire (e.g. Chalder Fatigue Scale)
    - Gout
        - Patient fatigue rating (e.g. visual analogue scale, other disease specific measure)
        - Generic fatigue questionnaire (e.g. Chalder Fatigue Scale)
- Erosions
    - Joint damage by X-ray (e.g. Sharp method, Larsen method, Lane Index, Wilke Index , Kellgren-Lawrence hand OA radiological index [9])
- Physical comorbidity
    - Major comorbidity
        - MACE (major adverse cardiac event)
        - Lung disease
        - Peptic ulcer disease
        - Liver disease

        - Renal disease
        - Tuberculosis / other serious infections
        - Diabetes
        - Hyperthyroidism
        - Depression
        - Cancer
        - Fractures
        - High cholesterol / dyslipidaemia
- Mental health
    - Mental health assessment questionnaires (e.g. Hospital Anxiety and Depression Scale (HADS), the AIMS, Mini-mental state examination)
- Quality of life (e.g. EQ-5D, SF-36)
    - Disease specific quality of life measures (e.g. RaQOL [11], ASQOL [12], PsAQoL [13])
- Work status
    - Categorical rating of work status (e.g. at work, retired, sick leave)
    - Number of days absent from work in a given time window

Supplementary table 6 – Description of reviews of aerobic exercise in OA

Table – Aerobic exercise (OA), description of reviews

Authors (date)	Review type	Study type included	Type of OA	Exposure detail	Number of studies included	Funders
Juhl (2014)[14]	MA	RCTs	Knee	Aerobic exercise	9	**Charity** (Health Insurance Foundation), **Professional body** (Danish Physiotherapy Association)
Corbett (2013)[15]	MA	RCTs	Knee	Aerobic exercise	114 §	**Government** (National Institute for Health Research [NIHR])
Tanaka (2013)[16]	MA	RCTs	Knee	Aerobic exercise	3	**No funding**
Uthman (2013)[17]	MA	RCTs	Knee, hip	Aerobic exercise	60 §	**Government** (National Institute for Health Research [NIHR])
Pozzobon (2018)[18]	SR	Observational	Knee, hip	Aerobic exercise prior to surgery	4	**No funding**
Wijnen (2018)[19]	SR	RCTs	Hip (post-surgery)	Aerobic exercise	2	**No funding**
Alrushud (2017)[20]	SR	RCTs	Knee	Aerobic exercise + dietary intervention	3	**Government** (Saudi Arabian Cultural Bureau), **University** (King Saud University)
Brosseau et al (2017)[21]	SR	RCTs	Knee	Aerobic exercise	5	**University** (University of Ottawa Research Chair)
de Rooij (2016)[22]	SR	Observational	Knee	Aerobic exercise	58	**Professional body** (Royal Dutch Society for Physical Therapy)
Bastick (2015)[23]	SR	Observational	Knee	Aerobic exercise	6	**Charity** (Dutch Arthritis Foundation)
Le Quintrec (2014)[24]	SR	RCTs	Knee, hip	Aerobic exercise in patients ≥70 years old	8	**Not reported** – authors declare no conflict of interest

§ Network meta-analysis looking at a range of exposures

MA = meta-analysis, OA = osteoarthritis, RCT = randomised controlled trial, SR = systematic review

Supplementary table 7 – Results from reviews of aerobic exercise studies in OA

Table – Aerobic exercise (OA), results and quality assessment

Outcome (outcome measure)	Study (date) [study type]	Standardised result, SMD (95% CI) unless otherwise stated	Natural result	AMSTAR2 quality	Rand. Seq.	Alloc. Conc.	Blind. Part.	Blind. Asses.
Pain	Juhl (2014) [MA][14]	Aerobic exercise vs no exercise<br>SMD -0.61 (-0.75, -0.48)		Moderate				
	Corbett (2013) [MA][15]	Aerobic exercise vs no exercise<br>SMD -0.55 (-0.89, -0.21)		Moderate				
	Tanaka (2013) [MA][16]	Aerobic exercise vs no exercise<br>SMD -0.45 (-0.77, -0.13)		Moderate				
	Uthman (2013) [MA][17]	Aerobic exercise vs no exercise<br>SMD -0.41 (-1.13, 0.30)		Moderate				
	Pozzobon (2018) [SR][18]		Patients who did more physical activity prior to surgery had less pain after surgery	Low				
	Alrushad (2017) [SR][20]	Aerobic exercise vs no exercise<br>SMD -0.24 (-0.50, 0.02) §		Moderate				
	Brosseau (2017) [SR][21]		One study reported improvements in pain	Moderate				
	de Rooij (2016) [SR][22]		Amount of general physical activity, practicing different sports and amount of sport did not predict pain levels. Physical activity at baseline weakly predicted pain	Moderate				
	Le Quintrec (2014) [SR][24]		4/5 trials reported improvements in pain after aerobic exercise in older adults over controls	Critically low				
Function	Juhl (2014) [MA][14]	Aerobic exercise vs no exercise<br>SMD -0.58 (-0.75, -0.40)		Moderate				
	Uthman (2013) [MA][17]	Aerobic exercise vs no exercise<br>SMD -0.30 (-1.53, 0.92)		Moderate				
	Pozzobon (2018) [SR][18]		The evidence of an association between aerobic activity and improved function following surgery was less clear	Low				
	Wijnen (2018) [SR][19]		1/2 studies reported improvements in function	Moderate				
	Alrushad (2017) [SR][20]	Aerobic exercise vs no exercise<br>SMD -0.34 (-0.59, -0.08) §		Moderate				
	Brosseau (2017) [SR][21]		One study reported improvements in function	Moderate				
	Le Quintrec (2014) [SR][24]		4/5 trials reported improvements in function after aerobic exercise in older adults over controls	Critically low				
	de Rooij (2016) [SR][22]		Physical activity was weakly associated with predicted function	Moderate				
QoL	Brosseau (2017) [SR][21]		One study reported improvements in QoL	Moderate				
Radiographic progression	Bastick (2015) [SR][23]		0/2 studies of running and 1/6 studies of regular sports reported an associated with radiographic progression	Moderate				

§ Calculated from one study included in the review that reported on function – pain[25], function[26]; Alloc. Conc. = allocation concealment, AMSTAR2 = Assessing the Methodological Quality of Systematic Reviews, BL = baseline, Blind. Asses. = Blinded assessors, Blind. Part. = blinded participants, CI = confidence interval, H/UC = high / unclear risk of bias, L = low risk of bias, MA = meta-analysis, OA= osteoarthritis, QoL = Quality of Life, Rand. Seq. = random sequence generation, RCT = randomised controlled trial, SD = standard deviation, SMD = standardised mean difference, SR = systematic review

Supplementary table 8 – Description of reviews of aquatic exercise in OA

Table – Aquatic exercise (OA), description of reviews

Authors (date)	Review type	Study type included	Type of OA	Exposure detail	Number of studies included	Funders
Beumer (2016)[27]	MA	RCTs	Hip	Aquatic exercise	4	**Not reported** – authors declare no conflicts of interest
Bartels (2016)[28]	MA	RCTs	Knee, hip	Aquatic exercise	13	**Charity** (Oak Foundation), **University** (Copenhagen University Library)
Lu (2015)[29]	MA	RCTs	Knee	Aquatic exercise	6	**Government** (State Administration of Traditional Chinese Medicine)
Waller (2014)[30]	MA	RCTs	Knee, hip	Aquatic exercise	11	**Government** (Academy of Finland, Social Insurance Institution of Finland)
Uthman (2013)[17]	MA	RCTs	Knee, hip	Aquatic exercise	60 §	**Government** (National Institute for Health Research [NIHR])
Le Quintrec (2014)[24]	SR	RCTs	Knee, hip	Aquatic exercise in patients ≥70 years old	5	**Not reported** – authors declare no conflict of interest
McAlindon (2014)[31]	SR	MA, SR, RCTs	Knee	Aquatic exercise	1	**Professional body** (OARSI)
Romeo (2013)[32]	SR	RCTs	Hip	Aquatic exercise [studies published 2007-12]	2	**Not reported**

§ Network meta-analysis looking at a range of exposures

MA = meta-analysis, OA = osteoarthritis, OARSI = Osteoarthritis Research Society International, RCT = randomised controlled trial, SR = systematic review

Supplementary table 9 – Results from reviews of aquatic exercise studies in OA

Table – Aquatic exercise (OA), results and quality assessment

Outcome (outcome measure)	Study (date) [study type]	Standardised result, SMD (95% CI) unless otherwise stated	Natural result	AMSTAR2 quality	Rand. Seq.	Alloc. Conc.	Blind. Part.	Blind. Asses.
Pain	Beumer (2016) [MA][27]	Aquatic exercise vs no exercise<br>SMD -0.53 (-0.96, -0.10)		Moderate				
	Bartels (2016) [MA][28]	Aquatic exercise vs no exercise<br>SMD -0.31 (-0.47, -0.15)		High				
	Lu (2015) [MA][29]	Aquatic exercise vs no exercise<br>SMD -1.16 (-3.03, 0.71)		Moderate				
	Waller (2014) [MA][30]	Aquatic exercise vs no exercise<br>SMD -0.26 (-0.41, -0.11)		Moderate				
	Uthman (2013) [MA][17]	Aquatic exercise vs no exercise<br>Aquatic strengthening SMD -0.75 (-1.42, -0.07);<br>Aquatic flexibility + strengthening SMD -0.96 (-1.64, -0.27);<br>Aquatic flexibility + aerobic SMD -0.07 (-0.98, 0.83);<br>Aquatic strengthening + aerobic SMD -0.92 (-2.08, 0.25);<br>Aquatic combined SMD -0.45 (-1.02, 0.11)		Moderate				
	Le Quintrec (2014) [SR][24]		2/2 studies reported aquatic exercise was better than no exercise for pain	Critically low				
	McAlindon (2014) [SR][31]		Identified one systematic review[33] that reported minor short-term benefits of aquatic exercise on pain	Moderate				
Function	Bartels (2016) [MA][28]	Aquatic exercise vs no exercise<br>SMD -0.32 (-0.47, -0.17)		High				
	Lu (2015) [MA][29]	Aquatic exercise vs no exercise<br>SMD -0.55 (-0.94, -0.16)		Moderate				
	Waller (2014) [MA][30]	Aquatic exercise vs no exercise<br>Self-report: SMD -0.30 (-0.43, -0.18)<br>Objective measure: SMD -0.22 (-0.38, -0.07)		Moderate				
	Uthman (2013) [MA][17]	Aquatic exercise vs no exercise<br>Aquatic strengthening SMD -0.43 (-1.42, 0.56);<br>Aquatic flexibility + strengthening SMD -0.61 (-1.75, -0.52);<br>Aquatic flexibility + aerobic SMD 0.07 (-1.23, 1.36);<br>Aquatic strengthening + aerobic SMD -0.86 (-2.52, 0.79),<br>Aquatic combined SMD -0.49 (-1.32, 0.33)		Moderate				
	Le Quintrec (2014) [SR][24]		2/2 studies reported aquatic exercise was better than no exercise for function	Critically low				
	McAlindon (2014) [SR][31]		Identified one systematic review[33] that reported moderate short-term benefits of aquatic exercise on function	Moderate				
	Romeo (2013) [SR][32]		1 study reporting no improvement in function	Moderate				

Alloc. Conc. = allocation concealment, AMSTAR2 = Assessing the Methodological Quality of Systematic Reviews, Blind. Asses. = Blinded assessors, Blind. Part. = blinded participants, CI = confidence interval, H/UC = high / unclear risk of bias, L = low risk of bias, MA = meta-analysis, OA= osteoarthritis, Rand. Seq. = random sequence generation, RCT = randomised controlled trial, SD = standard deviation, SMD = standardised mean difference, SR = systematic review

Table –Aquatic exercise (OA) cont., results and quality assessment

Outcome (outcome measure)	Study (date) [study type]	Standardised result, SMD (95% CI) unless otherwise stated	Natural result	AMSTAR2 quality	Rand. Seq.	Alloc. Conc.	Blind. Part.	Blind. Asses.
QoL	Bartels (2016) [MA][28]	Aquatic exercise vs no exercise<br>SMD -0.25 (-0.47, -0.01)		High				
	Waller (2014) [MA][30]	Aquatic exercise vs no exercise<br>Self-report: SMD -0.24 (-0.45, -0.04)		Moderate				
	Lu (2015) [MA][29]	Aquatic exercise vs no exercise<br>SMD -0.21 (-0.59, 0.18)		Moderate				
	McAlindon (2014) [SR][31]		Identified one systematic review[33] that reported moderate short-term benefits of aquatic exercise on QoL	Moderate				
Stiffness	Waller (2014) [MA][30]	Aquatic exercise vs no exercise<br>Self-report: SMD -0.20 (-0.36, -0.03)		Moderate				

Alloc. Conc. = allocation concealment, AMSTAR2 = Assessing the Methodological Quality of Systematic Reviews, Blind. Asses. = Blinded assessors, Blind. Part. = blinded participants, CI = confidence interval, H/UC = high / unclear risk of bias, L = low risk of bias, MA = meta-analysis, OA= osteoarthritis, QoL = Quality of life, Rand. Seq. = random sequence generation, RCT = randomised controlled trial, SD = standard deviation, SMD = standardised mean difference

Supplementary table 10 – Description of reviews of guidelines regarding exercise in OA

Table –Guidelines (OA), description of reviews

Authors (date)	Review type	Study type included	Type of OA	Exposure detail	Number of studies included	Funders
Gay (2016)[34]	SR	Guidelines	Knee, hip	Guidelines on exercise	8	**Industry** (Innovatherm)
Nelson (2014)[35]	SR	Guidelines	Knee, hip, hand	Guidelines on exercise	15	**Professional body** (U.S. Bone and Joint Initiative)
Fernandes (2013)[36]	SR	RCTs, Observational studies	Knee, hip	Exercise studies	95	**Professional body** (EULAR)

EULAR = European League Against Rheumatism, MA = meta-analysis, OA = osteoarthritis, RCT = randomised controlled trial, SR = systematic review, US = United States of America

Supplementary table 11 – Results from guidelines regarding exercise in OA

Table – Guidelines (OA), results and quality assessment

Outcome (outcome measure)	Study (date) [study type]	Standardised result, SMD (95% CI) unless otherwise stated	Natural result	AMSTAR2 quality	Rand. Seq.	Alloc. Conc.	Blind. Part.	Blind. Asses.
Pain	Gay (2016) [SR][34]		Exercise is a pillar of non-pharmacological OA treatment and leads to better pain and QoL – evidence graded A (i.e. "strong")	Moderate				
	Fernandes (2013) [SR][36]		Recommended patients with OA should have a regular, individualised exercise regime to improve pain	Critically low§				
Function	Fernandes (2013) [SR][36]		Recommended patients with OA should have a regular, individualised exercise regime to improve function	Critically low§				
QoL	Gay (2016) [SR][34]		Exercise is a pillar of non-pharmacological OA treatment and leads to better pain and QoL – evidence graded A (i.e. "strong")	Moderate				
Overall health	Nelson (2014) [SR][35]		12/15 guidelines strongly recommended exercise for knee and hip OA, less agreement regarding hand OA	Moderate				

§ Recommendations paper and so there is little information on the systematic review that was carried out to support the recommendations.

Alloc. Conc. = allocation concealment, AMSTAR2 = Assessing the Methodological Quality of Systematic Reviews, Blind. Asses. = Blinded assessors, Blind. Part. = blinded participants, CI = confidence interval, H/UC = high / unclear risk of bias, L = low risk of bias, OA= osteoarthritis, QoL = Quality of life, Rand. Seq. = random sequence generation, RCT = randomised controlled trial, SD = standard deviation, SMD = standardised mean difference, SR = systematic review

Supplementary table 12 – Description of reviews of studies comparing high and low intensity exercise in OA

Table –High vs low intensity (OA), description of reviews

Authors (date)	Review type	Study type included	Type of OA	Exposure detail	Number of studies included	Funders
Regnaux (2015)[37]	MA	RCTs	Knee, hip	Comparisons between high and low intensity exercise	6	**University** (EHESP – French School of Public Health, Centre de recherche Epidémiologies et Biostatistique, INSERM U1153), **Hospital** (Hôpital Hôtel-Dieu)
de Rooij (2016)[38]	SR	Cohort studies	Hip	Levels of exercise intensity	1	**Professional body** (Royal Dutch Society for Physical Therapy)

MA = meta-analysis, OA = osteoarthritis, RCT = randomised controlled trial, SR = systematic review

Supplementary table 13 – Results from reviews of studies comparing high and low intensity exercise in OA

Table –High vs low intensity (OA), results and quality assessment

Outcome (outcome measure)	Study (date) [study type]	Standardised result, SMD (95% CI) unless otherwise stated	Natural result	AMSTAR2 quality	Rand. Seq.	Alloc. Conc.	Blind. Part.	Blind. Asses.
Pain	Regnaux (2015) [MA][37]		WOMAC pain at study completion, high vs low intensity<br>Meta-MD: -0.84 (-1.63, -0.04)	High				
Function	Regnaux (2015) [MA][37]		WOMAC pain at study completion, high vs low intensity<br>Meta-MD: -2.65 (-5.29, -0.01)	High				
	de Rooij (2016) [SR][38]		One observational study reported that no supervised exercise and lower levels of physical exercise were associated with a deterioration of physical functioning	Moderate				

Alloc. Conc. = allocation concealment, AMSTAR2 = Assessing the Methodological Quality of Systematic Reviews, Blind. Asses. = Blinded assessors, Blind. Part. = blinded participants, CI = confidence interval, H/UC = high / unclear risk of bias, L = low risk of bias, MA = meta-analysis, MD = mean difference, OA= osteoarthritis, Rand. Seq. = random sequence generation, RCT = randomised controlled trial, SD = standard deviation, SMD = standardised mean difference, SR = systematic review, WOMAC = Western Ontario and McMaster Universities Osteoarthritis Index

Supplementary table 14 – Description of reviews of studies of home exercise in OA

Table – Home exercise (OA), description of reviews

Authors (date)	Review type	Study type included	Type of OA	Exposure detail	Number of studies included	Funders
Anwer (2016)[39]	MA	RCTs, CC	Knee	Home exercise vs no exercise / supervised exercise	16	**University** (King Saud University)

CC = case-control studies, MA = meta-analysis, OA = osteoarthritis, RCT = randomised controlled trial, SR = systematic review

Supplementary table 15 – Results from reviews of studies of home exercise in OA

Table – Home exercise (OA), results and quality assessment

Outcome (outcome measure)	Study (date) [study type]	Standardised result, SMD (95% CI) unless otherwise stated	Natural result	AMSTAR2 quality	Rand. Seq.	Alloc. Conc.	Blind. Part.	Blind. Asses.
Pain	Anwer (2016) [MA][39]	Home exercise vs no exercise<br>SMD -0.46 (-0.68, -0.24)<br>Home exercise vs other exercise intervention<br>SMD 0.23 (-0.02, 0.43)		Low				
Function	Anwer (2016) [MA][39]	Home exercise vs no exercise<br>SMD -0.35 (-0.56, -0.15)<br>Home exercise vs other exercise intervention<br>SMD 0.37 (0.17, 0.57)		Low				

Alloc. Conc. = allocation concealment, AMSTAR2 = Assessing the Methodological Quality of Systematic Reviews, Blind. Asses. = Blinded assessors, Blind. Part. = blinded participants, CI = confidence interval, H/UC = high / unclear risk of bias, L = low risk of bias, MA = meta-analysis, OA= osteoarthritis, Rand. Seq. = random sequence generation, SMD = standardised mean difference

Supplementary table 16 – Description of reviews of studies of land-based exercise in OA

Table – Land-based exercise (OA), description of reviews

Authors (date)	Review type	Study type included	Type of OA	Exposure detail	Number of studies included	Funders
Moseng (2017)[40]	MA	RCTs	Hip	Compliance to ACSM recommendations	12	**Professional body** (Norwegian Fund for Postgraduate Training in Physiotherapy)
Fernandopulle (2017)[41]	MA	RCTs	Knee, hip	Walking, recreational activities	14	**University** (School of Physiotherapy Research Support Fund, University of Otago)
Beumer (2016)[27]	MA	RCTs	Hip	Land based exercise	6	**Not reported** – authors declare no conflicts of interest
Henriksen (2016)[42]	MA	RCTs	Knee	Land based exercise – studies included in Cochrane reviews	34	**Charity** (Oak Foundation)
Fransen (2015)[43]	MA	RCTs	Knee	Land based exercise	44	**Government** (National Health and Medical Research Council, Australia)
McAlindon (2014)[31]	SR	MA, SRs, RCTs	Knee	Land based exercises	4	**Professional body** (OARSI)
Romeo (2013)[32]	SR	RCTs	Hip	Land-based exercise studies published 2007-12	3	**Not reported**

ACSM = American College of Sports Medicine, MA = meta-analysis, OA = osteoarthritis, OARSI = Osteoarthritis Reserch Society International, RCT = randomised controlled trial, SR = systematic review

Supplementary table 17 – Results from reviews of studies of land-based exercise in OA

Table – Land-based exercise (OA), results and quality assessment

Outcome (outcome measure)	Study (date) [study type]	Standardised result, SMD (95% CI) unless otherwise stated	Natural result	AMSTAR2 quality	Rand. Seq.	Alloc. Conc.	Blind. Part.	Blind. Asses.
Pain	Moseng (2017) [MA][40]	Exercise vs no exercise<br>SMD -0.24 (-0.42, -0.06)<br>Exercise with high compliance to ACSM vs no exercise<br>SMD -0.42 (-0.58, -0.26)<br>Exercise with low compliance to ACSM vs no exercise<br>SMD 0.04 (-0.24, 0.31)		Moderate				
	Fernandopulle (2017) [MA][41]	Walking vs control<br>3 months: 0.19 (-0.31, 0.68)<br>6 months: -1.55 (-3.62, 0.52)		Low				
	Beumer (2016) [MA][27]	Exercise vs minimal control<br>≤3 months: -0.40 (-1.06, 0.25)<br>4-12 months: -0.23 (-0.48, 0.03)<br>>12 months: -0.22 (-0.51, 0.06)		Moderate				
	Henriksen (2016) [MA][42]	Exercise vs sham, placebo or no intervention<br>SMD -0.46 (-0.59, -0.34)		Low				
	Fransen (2015) [MA][43]	Exercise vs no exercise controls<br>SMD -0.49 (-0.59, -0.39)		Moderate				
	McAlindon (2014) [SR][31]		4 MAs found small but clinically meaningful benefits of land-based exercise for pain – SMDs ranged from -0.34 (-0.19, -0.49) to -0.63 (-0.87, -0.39)	Moderate				
	Romeo (2013) [SR][32]		3 studies reported no evidence for effectiveness for pain. Controls were a mixture of usual care and other exercise types.	Moderate				

ACSM = American College of Sports Medicine, Alloc. Conc. = allocation concealment, AMSTAR2 = Assessing the Methodological Quality of Systematic Reviews, Blind. Asses. = Blinded assessors, Blind. Part. = blinded participants, CI = confidence interval, H/UC = high / unclear risk of bias, L = low risk of bias, MA = meta-analysis, OA= osteoarthritis, Rand. Seq. = random sequence generation, RCT = randomised controlled trial, SMD = standardised mean difference, SR =systematic review

Table – Land-based exercise (OA) cont., results and quality assessment

Outcome (outcome measure)	Study (date) [study type]	Standardised result, SMD (95% CI) unless otherwise stated	Natural result	AMSTAR2 quality	Rand. Seq.	Alloc. Conc.	Blind. Part.	Blind. Asses.
Function	Moseng (2017) [MA][40]	Exercise vs no exercise<br>SMD -0.34 (-0.50, -0.18)<br>Exercise with high compliance to ACSM vs no exercise<br>SMD -0.41 (-0.58, -0.24)<br>Exercise with low compliance to ACSM vs no exercise<br>SMD -0.23 (-0.52, 0.06)		Moderate				
	Fernandopulle (2017) [MA][41]		Walking vs control, mean difference (95% CI)<br>6 months: -10.38 (-12.27, -8.48)<br>Recreational activities vs control, WOMAC mean difference (95% CI)<br>-9.56 (-13.95, -5.17)<br>Conditioning exercises vs control, WOMAC mean difference (95% CI)<br>-3.74 (-5.70, -1.78)	Low				
	Fransen (2015) [MA][43]	Exercise vs no exercise controls<br>SMD -0.52 (-0.64, -0.39)		Moderate				
	McAlindon (2014) [SR][31]		4 MAs found small but clinically meaningful benefits of land-based exercise for function – SMD 0.25 (0.03, 0.48)	Moderate				
	Romeo (2013) [SR][32]		3 studies reported positive results for physical function. Controls were a mixture of usual care and other exercise types.	Moderate				
QoL	Fransen (2015) [MA][43]	Exercise vs no exercise controls<br>SMD 0.28 (0.15, 0.40)		Moderate				

ACSM = American College of Sports Medicine, Alloc. Conc. = allocation concealment, AMSTAR2 = Assessing the Methodological Quality of Systematic Reviews, Blind. Asses. = Blinded assessors, Blind. Part. = blinded participants, CI = confidence interval, H/UC = high / unclear risk of bias, L = low risk of bias, MA = meta-analysis, OA= osteoarthritis, QoL = Quality of Life, Rand. Seq. = random sequence generation, RCT = randomised controlled trial, SMD = standardised mean difference, SR =systematic review, WOMAC = Western Ontario and McMaster Universities Osteoarthritis Index

Supplementary table 18 – Description of reviews of studies of multidisciplinary exercise interventions in OA

Table – Multidisciplinary interventions (OA), description of reviews

Authors (date)	Review type	Study type included	Type of OA	Exposure detail	Number of studies included	Funders
Aebischer (2016)[44]	MA	RCTs, observational	Hand	Exercise therapy	10	**Professional body** (Swiss Society for Hand Rehabilitation, Swiss Physiotherapy Association)
Finney (2016)[45]	SR	RCTs	≥2 joints	Multidisciplinary interventions	4	**Government** (National Institute for Health Research)

BMI = body mass index, MA = meta-analysis, OA = osteoarthritis, RCT = randomised controlled trial, SR = systematic review

Supplementary table 19 – Results from reviews of studies of multidisciplinary exercise interventions in OA

Table – Multidisciplinary interventions (OA), results and quality assessment

Outcome (outcome measure)	Study (date) [study type]	Standardised result, SMD (95% CI) unless otherwise stated	Natural result	AMSTAR2 quality	Rand. Seq.	Alloc. Conc.	Blind. Part.	Blind. Asses.
Pain	Aebischer (2016) [MA][44]	Exercise therapy vs control<br>SMD -3.16 (-5.56, -0.75)		Moderate				
	Finney (2016) [SR][45]		Education + exercise interventions vs single discipline interventions, no interventions or usual care<br>3/4 studies reported improvements in pain	Moderate				
Function	Aebischer (2016) [MA][44]	Exercise therapy vs control<br>SMD -0.66 (-1.55, 0.23)		Moderate				
	Finney (2016) [SR][45]		Education + exercise interventions vs single discipline interventions, no interventions or usual care<br>0/3 studies reported improvements in function	Moderate				
QoL	Finney (2016) [SR][45]		Education + exercise interventions vs single discipline interventions, no interventions or usual care<br>3/4 studies reported improvements in QoL	Moderate				

Alloc. Conc. = allocation concealment, AMSTAR2 = Assessing the Methodological Quality of Systematic Reviews, Blind. Asses. = Blinded assessors, Blind. Part. = blinded participants, CI = confidence interval, H/UC = high / unclear risk of bias, L = low risk of bias, OA= osteoarthritis, QoL = Quality of life, Rand. Seq. = random sequence generation, RCT = randomised controlled trial, SMD = standardised mean difference, SR = systematic review

Supplementary table 20 – Description of reviews of studies of muscle strengthening exercise in OA

Table – Muscle strenghtening exercise (OA), description of reviews

Authors (date)	Review type	Study type included	Type of OA	Exposure detail	Number of studies included	Funders
Bartholdy (2017)[46]	MA	RCTs	Knee	ACSM muscle strengthening exercise interventions	45	**Charity** (Oak Foundation)
Magni (2017)[47]	MA	RCTs	Hand	Muscle strengthening training	5	**No funding**
Osteras (2017)[48]	MA	RCTs	Hand	Muscle strengthening exercises / stretching	7	**Professional body** (Norwegian Fund for Postgraduate Training in Physiotherapy)
Coudeyre (2016)[49]	MA	RCTs	Knee	Isokinetic muscle strengthening	9	**No funding**
Li (2016)[50]	MA	RCTs	Knee	Muscle strengthening exercises	17	**Government** (State Administration of Traditional Chinese Medicine)
Juhl (2014)[14]	MA	RCTs	Knee	Muscle strengthening exercise	32	**Charity** (Health Insurance Foundation), **Professional body** (Danish Physiotherapy Association)
Corbett (2013)[15]	MA	RCTs	Knee	Muscle strengthening exercise	114 §	**Government** (National Institute for Health Research [NIHR])
Tanaka (2013)[16]	MA	RCTs	Knee	Muscle strengthening exercise	7	**No funding**
Uthman (2013)[17]	MA	RCTs	Knee, hip	Muscle strengthening exercise	60 §	**Government** (National Institute for Health Research [NIHR])
Wijnen (2018)[19]	SR	RCTs	Hip	Muscle strengthening exercises following surgery	7	**No funding**
Brosseau (2017)[51]	SR	RCTs	Knee	Muscle strengthening exercises	26	**University** (University of Ottawa Research Chair)
Brosseau (2016)[52]	SR	RCTs	Hip	Muscle strengthening exercises	2	**University** (University of Ottawa Research Chair)
McAlindon (2014)[31]	SR	MA, SR, RCTs	Knee	Muscle strengthening training	2	**Professional body** (OARSI)

§ Network meta-analysis looking at a range of exposures

ACSM = American College of Sports Medicine, MA = meta-analysis, OA = osteoarthritis, OARSI = Osteoarthritis Research Society International, RCT = randomised controlled trial, SR = systematic review

Supplementary table 21 – Results from reviews of studies of muscle strengthening exercise in OA

Table – Muscle strengthening *exercise (OA) cont., results and quality assessment*

Outcome (outcome measure)	Study (date) [study type]	Standardised result, SMD (95% CI) unless otherwise stated	Natural result	AMSTAR2 quality	Rand. Seq.	Alloc. Conc.	Blind. Part.	Blind. Asses.
Pain	Bartholdy (2017) [MA][46]	ACSM interventions vs other exercise interventions<br>SMD -0.11 (-0.45, 0.24)		Moderate				
	Magni (2017) [MA][47]	Exercise vs no exercise<br>SMD -0.23 (-0.42, -0.04)		Moderate				
	Osteras (2017) [MA][48]	Exercise vs no exercise<br>Post-treatment: SMD -0.27 (-0.47, -0.07)<br>Medium/long term: SMD 0.09 (-0.18, 0.35)		Moderate				
	Coudeyre (2016) [MA][49]	Isokinetic muscle strengthening vs no exercise<br>SMD -1.19 (-1.67, -0.70)<br>Isokinetic muscle strengthening vs other exercise<br>SMD -1.24 (-1.67, -0.81)		Low				
	Li (2016) [MA][50]	Exercise vs no exercise<br>SMD -0.43 (-0.57, -0.29)		Moderate				
	Juhl (2014) [MA][14]	Exercise vs no exercise<br>SMD -0.62 (-0.79, -0.45)		Moderate				
	Corbett (2013) [MA][15]	Exercise vs no exercise<br>SMD -0.40 (-0.61, -0.19)		Moderate				
	Tanaka (2013) [MA][16]	Exercise vs no exercise<br>Non-weight bearing: SMD -1.42 (-2.09, -0.75)<br>Weight bearing: SMD -0.70 (-1.05, -0.35)		Moderate				
	Uthman (2013) [MA][17]	Exercise vs no exercise<br>SMD -0.81 (-1.13, -0.50)		Moderate				
	Brosseau (2017) [SR][51]		14/16 studies of strengthening programs reported clinical and significant improvements in pain.	Moderate				
	Brosseau (2016) [SR][52]		2 studies included, both showed clinical improvement, 1 showed statistically significant improvement [control = non-exercise activity / waiting list]	Moderate				
	McAlindon (2014) [SR][31]		2011 MA and SR demonstrated moderate effect size for reducing pain. Pain SMD -0.38 (-0.54, -0.23)	Moderate				

ACSM = American College of Sports Medicine, Alloc. Conc. = allocation concealment, AMSTAR2 = Assessing the Methodological Quality of Systematic Reviews, Blind. Asses. = Blinded assessors, Blind. Part. = blinded participants, CI = confidence interval, H/UC = high / unclear risk of bias, L = low risk of bias, MA = meta-analysis, OA= osteoarthritis, Rand. Seq. = random sequence generation, SMD = standardised mean difference, SR = systematic review

Table – Muscle strengthening *exercise (OA) cont., results and quality assessment*

Outcome (outcome measure)	Study (date) [study type]	Standardised result, SMD (95% CI) unless otherwise stated	Natural result	AMSTAR2 quality	Rand. Seq.	Alloc. Conc.	Blind. Part.	Blind. Asses.
Function	Bartholdy (2017) [MA][46]	ACSM interventions vs other exercise interventions<br>SMD -0.15 (-0.55, 0.24)		Moderate				
	Magni (2017) [MA][47]	Exercise vs no exercise<br>SMD -0.10 (-0.33, 0.13)		Moderate				
	Osteras (2017) [MA][48]	Exercise vs no exercise<br>Post-treatment: SMD -0.28 (-0.58, 0.02)<br>Medium/long term: SMD -0.05 (-0.31, 0.21)		Moderate				
	Coudeyre (2016) [MA][49]	Isokinetic muscle strengthening vs other exercise<br>SMD -0.58 (-1.11, -0.04)		Low				
	Li (2016) [MA][50]	Exercise vs no exercise<br>SMD -0.53 (-0.70, -0.37)		Moderate				
	Juhl (2014) [MA][14]	Exercise vs no exercise<br>SMD -0.60 (-0.83, -0.37)		Moderate				
	Uthman (2013) [MA][17]	Exercise vs no exercise<br>SMD -0.37 (-0.84, 0.09)		Moderate				
	Wijnen (2018) [SR][19]		4/7 studies reported significant improvements in joint function. 3/5 reported significant improvements in function performance [predominantly usual care as control; some studies had another type of exercise as control]	Moderate				
	Brosseau (2017) [SR][51]		12/12 studies of strengthening programs reported clinical and significant improvements in function	Moderate				
	McAlindon (2014) [SR][31]		2011 MA and SR demonstrated moderate effect size for improving function. Function SMD -0.41 (-0.66, -0.17)	Moderate				
QoL	Osteras (2017) [MA][48]		SF12 at 12 months, mean difference (95% CI)<br>MD 0.30 (-3.72, 4.32)	Moderate				
	Brosseau (2017) [SR][51]		3/3 studies of strengthening programs reported clinical and significant improvements in function	Moderate				
Grip strength	Magni (2017) [MA][47]		Grip strength, MD (95% CI)<br>MD 1.35 (-0.84, 3.54)	Moderate				

ACSM = American College of Sports Medicine, Alloc. Conc. = allocation concealment, AMSTAR2 = Assessing the Methodological Quality of Systematic Reviews, Blind. Asses. = Blinded assessors, Blind. Part. = blinded participants, CI = confidence interval, H/UC = high / unclear risk of bias, L = low risk of bias, MA = meta-analysis, MD = mean difference, OA= osteoarthritis, QoL= quality of life, Rand. Seq. = random sequence generation, SMD = standardised mean difference, SR = systematic review

Supplementary table 22 – Description of reviews of studies of exercise therapy interventions in OA

Table – Physiotherapy / exercise therapy (OA), description of reviews

Authors (date)	Review type	Study type included	Type of OA	Exposure detail	Number of studies included	Funders
Briani (2018)[53]	MA	RCTs	Knee	Exercise therapy	23	**No funding**
Hurley (2018)[54]	MA	RCTs	Knee, hip	Exercise therapy	21	**Charity** (Arthritis Research UK)
Aebischer (2016)[44]	MA	RCTs, observational	Hand	Exercise therapy	10	**Professional body** (Swiss Society for Hand Rehabilitation, Swiss Physiotherapy Association)
Sampath (2016)[55]	MA	RCTs	Hip	Exercise therapy	7	**No funding**
Bertozzi (2015)[56]	MA	RCTs	Hand	Exercise therapy	13	**No funding**
Desveaux (2014)[57]	MA	RCTs	Unreported	Community based exercise interventions	4	**Not reported –** authors declare no conflicts of interest
Brosseau (2016)[52]	SR	RCTs	Hip	Therapeutic exercise	4	**University** (University of Ottawa research chair)
Ferreira (2015)[58]	SR	RCT	Knee	Exercise therapy	3	**Not reported**
Fehring (2013)[59]	SR	Reviews, RCTs	Knee	Physical therapy [advanced stage OA]	3	**Not reported –** authors declare no conflicts of interest

MA = meta-analysis, OA = osteoarthritis, RCT = randomised controlled trial, SR = systematic review

Supplementary table 23 – Results from reviews of studies of exercise therapy interventions in OA

Table – Physiotherapy / exercise therapy (OA), results and quality assessment

Outcome (outcome measure)	Study (date) [study type]	Standardised result, SMD (95% CI) unless otherwise stated	Natural result	AMSTAR2 quality	Rand. Seq.	Alloc. Conc.	Blind. Part.	Blind. Asses.
Pain	Hurley (2018) [MA][54]	Exercise vs control<br>SMD -0.20 (-0.28, -0.11)		High				
	Sampath (2016) [MA][55]	Exercise therapy vs control<br>Post-treatment: SMD -0.27 (-0.50, -0.04)<br>Follow-up: SMD -0.24 (-0.41, -0.06)		Moderate				
	Bertozzi (2015) [MA][56]	Exercise vs control<br>Short term: SMD -0.71 (-1.60, 0.19)<br>Long term: SMD -0.03 (-0.24, 0.18)		Moderate				
	Brosseau (2016) [SR][52]		2/2 studies showed clinically important improvement in pain, 1 was statistically significant	Moderate				
	Ferreira (2015) [SR][58]		Those allocated to strengthening therapy group had benefits for pain	Moderate				
	Fehring (2013) [SR][59]		1/3 studies of advanced disease reported improvements in pain following exercise	Critically low				
Function	Hurley (2018) [MA][54]	Exercise vs control<br>SMD -0.27 (-0.37, -0.17)		High				
	Sampath (2016) [MA][55]	Exercise therapy vs control<br>Post-treatment: SMD -0.29 (-0.47, -0.11)<br>Follow-up: SMD -0.33 (-0.50, -0.15)		Moderate				
	Bertozzi (2015) [MA][56]	Exercise vs control<br>Long term: SMD -0.07 (-0.28, 0.15)		Moderate				
	Desveaux (2014) [MA][57]		6MWT, intervention vs control<br>MD 41.65 (20.51, 62.79)<br>Standard function capacity, intervention, vs control<br>SMD 0.18 (0.05, 0.31)	Low				
	Brosseau (2016) [SR][52]		2/2 studies showed clinically important and statistically significant improvement in function	Moderate				
	Ferreira (2015) [SR][58]		Those allocated to strengthening therapy group had benefits for function	Moderate				
Depression	Hurley (2018) [MA][54]	Exercise vs control<br>SMD -0.16 (-0.29, -0.02)		High				
Anxiety	Hurley (2018) [MA][54]	Exercise vs control<br>SMD -0.11 (-0.26, 0.05)		High				
QoL	Briani (2018) [MA][53]	Exercise therapy vs control<br>SMD 0.70 (0.20, 1.20)		Moderate				
	Sampath (2016) [MA][55]	Exercise therapy vs control<br>SMD -0.06 (-0.27. 0.16)		Moderate				
Self-efficacy	Hurley (2018) [MA][54]	Exercise vs control<br>SMD 0.46 (0.34, 0.58)		High				

Alloc. Conc. = allocation concealment, AMSTAR2 = Assessing the Methodological Quality of Systematic Reviews, Blind. Asses. = Blinded assessors, Blind. Part. = blinded participants, CI = confidence interval, H/UC = high / unclear risk of bias, L = low risk of bias, MA = meta-analysis, MD = mean difference, OA= osteoarthritis, QoL= quality of life, Rand. Seq. = random sequence generation, RCT = randomised controlled trial, SD = standard deviation, SMD = standardised mean difference, SR = systematic review

Supplementary table 24 – Description of reviews of studies of Tai Chi in OA

Table – Tai-Chi (OA), description of reviews

Authors (date)	Review type	Study type included	Type of OA	Exposure detail	Number of studies included	Funders
Zhang (2017)[60]	MA	RCTs	Knee	Tai-Chi	8	**Government** (State Administration of Traditional Chinese Medicine)
Chen (2016)[61]	MA	RCT	Knee, hip, spine	Tai-Chi	9	**University** (University of British Columbia), **Charity** (British Columbia Lung Association)
Corbett (2013)[15]	MA	RCTs	Knee	Aerobic exercise	114 §	**Government** (National Institute for Health Research [NIHR])
Yan (2013)[62]	MA	RCT	Any joint	Tai-Chi	7	**No funding**
Brosseau (2017)[63]	SR	RCTs	Knee	Tai-Chi	4	**University** (University of Ottawa Research Chair)

§ Network meta-analysis looking at a range of exposures
MA = meta-analysis, OA = osteoarthritis, RCT = randomised controlled trial, SR = systematic review

Supplementary table 25 – Results from reviews of studies of Tai Chi in OA

Table – Tai-Chi (OA), results and quality assessment

Outcome (outcome measure)	Study (date) [study type]	Standardised result, SMD (95% CI) unless otherwise stated	Natural result	AMSTAR2 quality	Rand. Seq.	Alloc. Conc.	Blind. Part.	Blind. Asses.
Pain	Zhang (2017) [MA][60]	Tai-Chi vs no exercise<br>SMD -0.77 (-1.13, -0.41)		Moderate				
	Chen (2016) [MA][61]	Tai-Chi vs no exercise<br>SMD -0.53 (-0.75, -0.32)		Moderate				
	Corbett (2013) [MA][15]	Tai-Chi vs no exercise<br>SMD -0.51 (-1.03, 0.01)		Moderate				
	Yan (2013) [MA][62]	Tai-Chi vs control<br>SMD -0.45 (-0.70, -0.20)		Low				
	Brosseau (2017) [SR][63]		Qigong style = Clinical but not significant benefit;<br>Sun style = Clinical but not significant benefit	Moderate				
Function	Zhang (2017) [MA][60]	Tai-Chi vs no exercise<br>SMD -0.75 (-0.98, -0.52)		Moderate				
	Chen (2016) [MA][61]	Tai-Chi vs no exercise<br>SMD -0.70 (-0.93, -0.47)		Moderate				
	Yan (2013) [MA][62]	Tai-Chi vs control<br>SMD -0.61 (-0.85, -0.37)		Low				
	Brosseau (2017) [SR][63]		Qigong style = Clinical but not significant benefit;<br>Sun style = Clinical and significant benefit	Moderate				
Stiffness	Zhang (2017) [MA][60]	Tai-Chi vs no exercise<br>SMD -0.56 (-0.96, -0.16)		Moderate				
	Yan (2013) [MA][62]	Tai-Chi vs control<br>SMD -0.31 (-0.60, -0.02)		Low				
QoL	Zhang (2017) [MA][60]	Tai-Chi vs no exercise<br>SMD 0.57 (0.17, 0.97)		Moderate				
	Chen (2016) [MA][61]	Tai-Chi vs no exercise<br>SMD 0.38 (0.75, 0.01)		Moderate				
	Brosseau (2017) [SR][63]		Qigong style = Clinical and significant benefit;<br>Sun style = no benefit	Moderate				

Alloc. Conc. = allocation concealment, AMSTAR2 = Assessing the Methodological Quality of Systematic Reviews, Blind. Asses. = Blinded assessors, Blind. Part. = blinded participants, CI = confidence interval, H/UC = high / unclear risk of bias, L = low risk of bias, MA = meta-analysis, OA= osteoarthritis, QoL = quality of life, Rand. Seq. = random sequence generation, RCT = randomised controlled trial, SMD = standardised mean difference, SR = systematic review

Supplementary table 26 – Description of reviews of studies of yoga in OA

Table – Yoga (OA), description of reviews

Authors (date)	Review type	Study type included	Type of OA	Exposure detail	Number of studies included	Funders
Wang (2018)[64]	MA	RCTs	Knee	Yoga	5	**Government** (National Natural Science Foundation of China)
Brosseau (2017)[63]	SR	RCTs	Knee	Yoga	1	**University** (University of Ottawa Research Chair)
Kan (2016)[65]	SR	RCTs, single arm int.	Knee	Yoga	9	**Not reported** – Authors declare no conflict of interest
Cramer (2013)[66]	SR	RCTs	Knee, hip, hand	Yoga	3	**Charity** (the Rut- and Klaus-Bahlsen-Foundation)

int. = intervention, MA = meta-analysis, OA = osteoarthritis, RCT = randomised controlled trial, SR = systematic review

Supplementary table 27 – Results from reviews of studies of yoga in OA

Table –Yoga (OA), results and quality assessment

Outcome (outcome measure)	Study (date) [study type]	Standardised result, SMD (95% CI) unless otherwise stated	Natural result	AMSTAR2 quality	Rand. Seq.	Alloc. Conc.	Blind. Part.	Blind. Asses.
Pain	Brosseau (2017) [SR][63]		Clinical and significant benefit	Moderate				
	Kan (2016) [SR][65]		3/4 RCTs reported reductions in pain vs. exercise control, 2/2 before/after studies reported pain reductions	Moderate				
	Cramer (2013) [SR][66]		3 studies – very low evidence for the effect of yoga on pain	Moderate				
Function	Wang (2018) [MA][64]	Yoga vs no exercise<br>SMD -1.83 (-2.09, -1.57)		Moderate				
	Brosseau (2017) [SR][63]		Clinical but not significant benefit	Moderate				
	Kan (2016) [SR][65]		1/2 studies reported improved function vs. exercise control	Moderate				
	Cramer (2013) [SR][66]		3 studies – very low evidence for the effect of yoga on function	Moderate				
QoL	Kan (2016) [SR][65]		1/3 RCTs reported improvements in QoL vs. exercise control, 1 before/after study reported QoL improvement	Moderate				

Alloc. Conc. = allocation concealment, AMSTAR2 = Assessing the Methodological Quality of Systematic Reviews, Blind. Asses. = Blinded assessors, Blind. Part. = blinded participants, CI = confidence interval, H/UC = high / unclear risk of bias, L = low risk of bias, OA= osteoarthritis, QoL = quality of life, Rand. Seq. = random sequence generation, RCT = randomised controlled trial, SMD = standardised mean difference, SR = systematic review

Supplementary table 28 – Description of reviews of studies of aerobic exercise in RA

Table – Aerobic exercise (RA), description of reviews

Authors (date)	Review type	Study type included	Exposure detail	Number of studies included	Funders
Rongen-van Dartel (2015)[67]	MA	RCTs	Aerobic exercise	5	**Not reported**
Hernandez-Hernandez (2017)[68]	SR	MA, RCTs, observational	Aerobic exercise	15	**Government** (Spanish Ministry of Health, European Regional Development Fund), **Professional body** (Asociación para la Ayuda a la Investigación en Reumatología del Hospital Universitario de Canarias)
Siegel (2017)[69]	SR	Reviews, RCTs, observational	Aerobic exercise	2	**Not reported**
Larkin (2014)[70]	SR	RCTs, observational	Physical activity levels	10	"Funded by lead author as part of her postgraduate studies"

MA = meta-analysis, RA = rheumatoid arthritis, RCT = randomised controlled trial, SR = systematic review

Supplementary table 29 – Description of original studies of aerobic exercise in RA

Table – Aerobic exercise (RA), description of included studies

Author (date) [country]	Study design	Inclusion criteria	Exposure detail	N	Age years, mean (SD)	N (%) female	Funders
Katz (2018) [USA][71]	RCT	Physician diagnosed RA, English/Spanish speaker, able to attend 3 research visits, Fatigue Short Form ≥18<br>Exclusions: BMI<20, engaged in regular exercise, nonambulatory, condition that would prevent walking, lower extremity joint surgery upcoming or in past 6 months, myocardial infarction in last 6 months, stroke, congestive heart failure, severe chronic obstructive pulmonary disorder	1) Received pedometer + diary to keep track of step count<br>2) Pedometer + sleep target<br>p) education only	1) 34<br>2) 34<br>p) 28	1) 55.9 (12.4)<br>2) 50.2 (14.1)<br>p) 59.1 (12.5)	1) 30 (88.2)<br>2) 30 (88.2)<br>p) 24 (85.7)	**Charity** (Rheumatology Research Foundation)
Baxter (2016) [New Zealand][72]	RCT	2010 ACR RA criteria, symptom duration >2 years, aged >20 years, fluent English<br>Exclusions: medical condition preventing completion of the intervention, cognitive impairment	1) Walking pre-defined route, 3-4 times per week for 6 weeks<br>p) Nutritional advice + usual care	1) 11<br>p) 22	1) 66.6 (10.1)<br>p) 59.4 (12.9)	Not reported	**Charity** (Maurice and Phyllis Paykel Trust)
Feldthusen (2016) [Sweden][73]	RCT	RA (ICD10), aged 20-65 years, DAS28<3.8, Fatigue VAS >50, symptom duration >3 years, stable medication for >6 months<br>Exclusions: other illnesses precluding study participation, inability to communicate in Swedish	1) 12 weeks moderate and vigorous physical exercise therapy + guidelines for balancing stress and developing a self-care plan<br>p) No exercise	1) 36<br>p) 34	1) 54.2 (8.5)<br>p) 52.7 (10.9)	1) 32 (88.9)<br>p) 30 (88.2)	**University** (University of Gothenburg Centre for Person-Centred Care, Medical Faculty – University of Gothenburg Sahlgrenska), **Government** (Swedish Research Council
Sjoquist (2011) [Sweden][74]	RCT	Aged >18 years, communicate in Swedish, Perform body function test	1) One year program aim at implementing healthy physical activity (moderate intensity, 30 mins ≥4x per week). Coached on how to perform exercises<br>p) Did not received intervention<br>[Long-term follow-up of Brodin et al (2008)[75]	1) 94<br>p) 134			
Brodin (2008) [Sweden][75]	RCT	Aged >18 years, communicate in Swedish, Perform body function test	1) One year program aim at implementing healthy physical activity (moderate intensity, 30 mins ≥4x per week). Coached on how to perform exercises<br>p) Did not received intervention	1) 94<br>p) 134	1) 54 (14.0)<br>p) 56 (13.9)	1) 68 (72.3)<br>p) 101 (75.4)	**Government**, **Charity, and Professional body §**

§Swedish Research Council, the Vårdal Foundation, the Swedish Rheumatism Association, the Vasterbotten County Council Research Fund, the Stockholm County Council (EXPO), the Signe and Reinhold Sund Foundation, the Dalarna Research Council, the Rune and Ulla Almlov Foundation, the Swedish Social Insurance Agency (Dagmar 1999), and the Health Care Science Postgraduate School at Karolinska Institutet.
ACR = American College of Rheumatology, BMI = body mass index, DAS28 = Disease Activity Score 28, DMARDs = Disease Modifying Anti-Rheumatic Drugs, HAQ = Health Assessment Questionnaire, N = number, RA = rheumatoid arthritis, RCT = randomised controlled trial, SD = standard deviation, USA = United States of America, VAS = Visual Analogue Scale

Table – Aerobic exercise (RA) cont., description of included studies

Author (date) [country]	Study design	Inclusion criteria	Exposure detail	N	Age years, mean (SD)	N (%) female	Funders
Li (2006) [Canada][76]	RCT	Required physical therapy and/or occupational therapy, not received rehabilitation in last 2 years<br>Exclusions: joint replacement surgery in last 3 months, scheduled to received surgery in next 3 months	1) Physical therapist model – education on diagnosis, pain management, energy conservation and joint protection, advice on exercises and assistive devices<br>p) Traditional treatment model including info on pain management	1) 63<br>p) 48	1) 54.2 (14.4)<br>p) 56.8 (13.2)	1) 55 (87.3)<br>p) 38 (79.2)	**Government** (Canadian Institutes of Health Research)
Melikoglu (2006) [Turkey][77]	RCT	1987 ACR RA criteria, Not clinically active disease (clinically active = morning stiffness >30 mins, ≥6 tender joints, ≥3 swollen joints, ESR >22mm/h), stable DMARDs, Functional Class I-II	1) Treadmill exercises<br>2) Range of motion exercises	1) 20<br>2) 20	1) 46.4 (8.3)<br>2) 50.3 (9.7)	1) 20 (100)<br>2) 20 (100)	**Not reported**
Hansen (1993) [Denmark][78]	RCT	Definite or classical RA<br>Exclusions: Functional status III-IV, aged <20 or >60 years, disease other than RA with contraindicate / make physical training impossible, already training ≥3x per week	1) Self-training after instruction<br>2) As 1, plus training with physio once per week<br>3) As 1 plus weekly group training<br>4) As 3 plus training in hot pool<br>p) No training	1) 15<br>2) 15<br>3) 15<br>4) 15<br>p) 15	Median (IQR)<br>1) 55 (44, 58)<br>2) 52 (46, 58)<br>3) 51 (42, 56)<br>4) 54 (44, 56)<br>p) 51 (46, 57)	1) 12 (80.0)<br>2) 7 (46.7)<br>3) 9 (60.0)<br>4) 11 (73.3)<br>p) 10 (66.7)	**Charity** (Danish Arthritis Foundation, Danish Physiotherapists' Research Fund), **Government** (Danish Research Council, Fund for Medical Research in South Jutland)
Nordstrom (1996) [Finland][79]	NRT	1987 ACR RA criteria	1) 3 week program of aerobic exercises plus pain relief, steroid injections and range of motion physio<br>p) outpatient physiotherapy	1) 20<br>p) 6	Not reported	Not reported	**University** (Helsinki University Central Hospital, Centre for International Mobility), **Charity** (Invalid Foundation, Finska Lakaresallskapet, Perklen Foundation)
Minor (1995) [USA][80]	NRT	Aged 21-64 years, women, expressed intent to exercise, no pre-existing medical condition, function class I-II	1) 12 weeks – low impact aerobic dance, walking, aquatics<br>p) Usual care	1) 20<br>p) 22	1) 46.0 (13.1)<br>p) 54.8 (8.4)	1) 20 (100)<br>p) 22 (100)	**Charity** (Arthritis Foundation), **Government (**NIDRR)
Noreau (1995) [Canada][81]	NRT	Confirmed diagnosis of RA, Functional class I-II, Free from unstable cardiopulmonary disease, no acute joint pain, ability to perform graded exercise on bike	1) 12 weeks, 2 sessions per week, 15-30 minutes aerobic exercise or aerobic dance without jumps. Also counselling with psychologist<br>p) No exercise control	1) 19<br>p) 10	1) 49.3 (13.0)<br>p) 49.4 (11.9)	1) 12 (63.2)<br>p) 8 (80.0)	**Hospital** (Centre Francois-Charon)

ACR = American College of Rheumatology, DAS28 = Disease Activity Score 28, DMARDs = Disease Modifying Anti-Rheumatic Drugs, ESR = erythrocyte sedimentation rate, IQR = interquartile range, N = number, NIDRR = National Institute on Disability and Rehabilitation Research, NRT = non-randomised trial, RA = rheumatoid arthritis, RCT = randomised controlled trial, SD = standard deviation, USA = United States of America, VAS = Visual Analogue Scale

Table – Aerobic exercise (RA) cont., description of included studies

Author (date) [country]	Study design	Inclusion criteria	Exposure detail	N	Age years, mean (SD)	N (%) female	Funders
Ekblom (1975) [Sweden][82]	NRT	Second or third degree RA, in a "non-acute stage"	1) Intensive exercise group + continued training ≥4 times per week<br>2) Intensive exercise group + continued training about 2 times per week<br>3) Intensive exercise group + stopped training<br>p1) Control group + continued training about 2 times per week<br>p2) Control group + no change to physical activity	1) 6<br>2) 12<br>3) 5<br>p1) 3<br>p2) 4	Not reported	Not reported	**Not reported**
Nadareishvili (2008) [USA][83]	Case control	National Database for Rheumatic Diseases, aged 25-100 years	Amount of weekly exercise	1230	At index event cases: 70.0 (9.6)<br>Controls: 69.5 (10.6)	Cases: 74.6%<br>Controls: 74.7%	**Industry** (Abbott, Amgen, Wyeth-Australia, Merck, Pfizer)
Wolfe (2008) [USA][84]	Pros. Obser.	National Database for Rheumatic Diseases	Self-reported amount of physical exercise	17738	18-103	77.8%	**Industry** (Abbott, Amgen, Wyeth-Australia, Merck, Pfizer)
Stenstrom (1994) [Sweden][85]	Pros. Obser.	Classical or definite RA, functional class II, aged <70 years, absence of other chronic disease, no arthroplasty on weight baring joints, no serious psychosocial complications, expressed interest in exercise	Patients self-reported average aerobic exercise frequency during 6 month period. Group divided into low and high frequency exercise at median value	69	Low: 52 (12.2)<br>High: 56 (9.4)	Low: 25 (73)<br>high: 31 (89)	**Government** (Gavleborg County Council, Sormland County Council), **University** (Karolinska)

N = number, NRT = non-randomised trial, Pros. Obser. = Prospective observational, RA = rheumatoid arthritis, SD = standard deviation, USA = United States of America

Supplementary table 30 – Results from reviews and interventional studies of aerobic exercise in RA

Table – Aerobic exercise (RA), results and quality assessment

Outcome (outcome measure)	Study (date) [study type]	Standardised result, SMD (95% CI) unless otherwise stated	Natural result	AMSTAR2 quality	Rand. Seq.	Alloc. Conc.	Blind. Part.	Blind. Asses.
Pain	Hernandez-Hernandez (2017) [SR][68]		1 MA[86] concluded that aerobic exercise improves pain (SMD 0.31 [95% CI 0.06, 0.55])	Critically low				
	Siegel (2017) [SR][69]		1 MA[86] concluded that aerobic exercise improves pain (SMD 0.31 [95% CI 0.06, 0.55])	Moderate				
	Larkin (2014) [SR][70]		No correlation between pain and physical activity	Low				
	Katz (2018) [RCT][71]	Exercise vs control at week 21<br>Pedometer vs control: SMD 0.19 (-0.31, 0.70)<br>Pedometer + sleep target vs control: SMD -0.20 (-0.70, 0.30)	Pain interference, BL / week 21, mean (SD)<br>Pedometer: 61.7 (6.4) / 59.2 (7.6)<br>Pedometer + sleep target: 61.1 (8.1) / 55.9 (8.3)<br>Control: 59.8 (7.3) / 57.6 (9.0)		H/UC	L	H/UC	H/UC
	Feldthusen (2016) [RCT][73]	Exercise vs control at 12 weeks<br>SMD -0.24 (-0.71, 0.23)	Pain VAS, BL / 12 weeks, mean (SD)<br>Exercise: 38.7 (22.0) / 33.8 (21.2)<br>Control: 40.9 (20.9) / 39.1 (23.7)		L	L	H/UC	L
	Sjoquist (2011) [RCT][74]	Exercise vs control at 2 years<br>SMD 0.16 (-0.11, 0.42)	Pain VAS, BL / 2 years, mean (SD §)<br>Exercise: 34.0 (18.2) / 34.3 (18.2)<br>Control: 35.5 (18.8) / 31.5 (17.7)		L	H/UC	H/UC	L
	Brodin (2008) [RCT][75]	Exercise vs control at 1 year<br>SMD -0.13 (-0.40, 0.13)	Pain VAS, BL / 1 year, mean (SD §)<br>Exercise: 34.8 (18.8) / 34.8 (18.8)<br>Control: 35.5 (18.8) / 37.3 (18.8)		L	H/UC	H/UC	L
	Li (2006) [RCT][76]	Exercise education vs control at 6 months<br>SMD 0.06 (-0.31, 0.44)	Pain VAS (0-10), BL / 6 months, mean (SD)<br>Exercise education: 6.86 (2.43) / 5.73 (2.72)<br>Control: 6.79 (2.34) / 5.57 (2.40)		L	H/UC	H/UC	H/UC
	Melikoglu (2006) [RCT][77]	Aerobic exercise vs range of motion at 15 days<br>SMD -0.22 (-0.84, 0.40)	Pain VAS, BL / 15 days, mean (SD)<br>Aerobic exercise: 5.2 (1.7) / 4.3 (1.2)<br>Range of motion: 4.5 (2.3) / 4.6 (2.3)		H/UC	H/UC	H/UC	L
	Hansen (1993) [RCT][78]	Exercise vs control at 2 years<br>Individual vs control: SMD -1.33 (-2.13, -0.54)<br>Physio vs control: SMD 0.33 (-0.39, 1.05)<br>Group vs control: SMD 1.00 (0.24, 1.76)<br>Group + pool vs control: SMD -0.49 (-1.21, 0.24)	Pain VAS, BL / 2 years, mean (SD †)<br>Individual exercise: 1.6 (0.3) / 1.4 (0.3)<br>Physio: 1.8 (0.4) / 1.9 (0.3)<br>Group training: 1.9 (0.4) / 2.1 (0.3)<br>Group + pool: 1.9 (0.2) / 1.6 (0.5)<br>Control: 1.9 (0.2) / 1.8 (0.3)		H/UC	H/UC	H/UC	L
	Bespoke meta-analysis including[71;73;75;78]	Exercise vs control<br>SMD -0.28 (-0.71, 0.16), I^2 71.1%						

§ Mean (SD) estimated from median (range) using published formula[87]
† Mean (SD) estimated from median (interquartile range using published formula[87]
Alloc. Conc. = allocation concealment, AMSTAR2 = Assessing the Methodological Quality of Systematic Reviews, BL = baseline, Blind. Asses. = Blinded assessors, Blind. Part. = blinded participants, CI = confidence interval, H/UC = high / unclear risk of bias, L = low risk of bias, RA rheumatoid arthritis, Rand. Seq. = random sequence generation, RCT = randomised controlled trial, SD = standard deviation, SMD = standardised mean difference, SR = systematic review, VAS = visual analogue scale

Table – Aerobic exercise (RA) cont., results and quality assessment

Outcome (outcome measure)	Study (date) [study type]	Standardised result, SMD (95% CI) unless otherwise stated	Natural result	AMSTAR2 quality	Rand. Seq.	Alloc. Conc.	Blind. Part.	Blind. Asses.
Pain	Nordstrom (1996) [NRT][79]	Exercise vs physio at 6 months<br>SMD 0.26 (-0.66, 1.17)	Pain VAS, BL / 6 months, mean (SD)<br>Exercise intervention: 42.5 (24.5) / 30.7 (23.7)<br>Physio: 46.7 (21.0) / 24.8 (18.8)					
	Noreau (1995) [NRT][81]	Exercise vs control at 12 weeks<br>SMD -0.12 (-0.89, 0.65)	Pain (AIMS), BL / 12 weeks, mean (SD)<br>Exercise: 4.37 (2.15) / 3.47 (1.85)<br>Control: 4.00 (2.15) / 3.70 (2.06)					

AIM = Arthritis Impact Measurement Scale, Alloc. Conc. = allocation concealment, AMSTAR2 = Assessing the Methodological Quality of Systematic Reviews, BL = baseline, Blind. Asses. = Blinded assessors, Blind. Part. = blinded participants, CI = confidence interval, H/UC = high / unclear risk of bias, L = low risk of bias, NRT = non-randomised trial, RA rheumatoid arthritis, Rand. Seq. = random sequence generation, SD = standard deviation, SMD = standardised mean difference, VAS = visual analogue scale

Table – Aerobic exercise (RA), results and quality assessment

Outcome (outcome measure)	Study (date) [study type]	Standardised result, SMD (95% CI) unless otherwise stated	Natural result	AMSTAR2 quality	Rand. Seq.	Alloc. Conc.	Blind. Part.	Blind. Asses.
Function	Hernandez-Hernandez (2017) [SR][68]		1 MA[86] concluded that aerobic exercise improves function (SMD 0.24 [95% CI 0.10, 0.38])	Critically low				
	Larkin (2014) [SR][70]		No correlation between function and physical activity	Low				
	Katz (2018) [RCT][71]	<u>Exercise vs control at week 21</u><br>Pedometer vs control: SMD -0.09 (-0.60, 0.41)<br>Pedometer + sleep target vs control: SMD -0.35 (-0.86, 0.15)	<u>HAQ, BL / week 21, mean (SD)</u><br>Pedometer: 1.39 (0.62) / 1.26 (0.66)<br>Pedometer + sleep target: 1.39 (0.68) / 1.08 (0.74)<br>Control: 1.28 (0.63) / 1.32 (0.61)		H/UC	L	H/UC	H/UC
	Sjoquist (2011) [RCT][74]	<u>Exercise vs control at 2 years</u><br>SMD 0.24 (-0.03, 0.50)	<u>HAQ, BL / 2 years, mean (SD §)</u><br>Exercise: 0.81 (0.45) / 0.79 (0.48)<br>Control: 0.88 (0.48) / 0.69 (0.38)		L	H/UC	H/UC	L
	Brodin (2008) [RCT][75]	<u>Exercise vs control at 1 year</u><br>SMD -0.29 (-0.56, -0.03)	<u>HAQ, BL / 1 year, mean (SD §)</u><br>Exercise: 0.81 (0.45) / 0.75 (0.40)<br>Control: 0.88 (0.48) / 0.88 (0.48)		L	H/UC	H/UC	L
	Li (2006) [RCT][76]	<u>Exercise education vs control at 6 months</u><br>SMD 0.16 (-0.22, 0.54)	<u>HAQ, BL / 6 months, mean (SD)</u><br>Exercise education: 0.94 (0.66) / 0.92 (0.75)<br>Control: 0.82 (0.65) / 0.81 (0.60)		L	H/UC	H/UC	H/UC
	Melikoglu (2006) [RCT][77]	<u>Aerobic exercise vs range of motion at 15 days</u><br>SMD -0.06 (-0.68, 0.56)	<u>HAQ, BL / 15 days, mean (SD)</u><br>Aerobic exercise: 5.6 (5.3) / 4.7 (4.7)<br>Range of motion: 5.1 (5.5) / 5.0 (5.5)		H/UC	H/UC	H/UC	L
	Bespoke meta-analysis including[71;75]	<u>Exercise vs control</u><br>SMD -0.25 (-0.48, -0.01), I^2 0.0%						
	Nordstrom (1996) [NRT][79]	<u>Exercise vs physio at 6 months</u><br>SMD -0.16 (-1.07, 0.75)	<u>HAQ, BL / 6 months, mean (SD)</u><br>Exercise intervention: 5.5 (4.4) / 4.5 (3.1)<br>Physio: 5.5 (4.4) / 5.0 (3.3)					
	Noreau (1995) [NRT][81]	<u>Exercise vs control at 12 weeks</u><br>SMD 0.11 (-0.66, 0.88)	<u>Physical activity (AIMS), BL / 12 weeks, mean (SD)</u><br>Exercise: 3.79 (2.39) / 3.26 (2.51)<br>Control: 3.00 (2.36) / 3.00 (2.16)					

AIM = Arthritis Impact Measurement Scale, Alloc. Conc. = allocation concealment, AMSTAR2 = Assessing the Methodological Quality of Systematic Reviews, BL = baseline, Blind. Asses. = Blinded assessors, Blind. Part. = blinded participants, CI = confidence interval, H/UC = high / unclear risk of bias, HAQ = Health Assessment Questionnaire, L = low risk of bias, MA = meta-analysis, NRT = non-randomised trial, RA rheumatoid arthritis, Rand. Seq. = random sequence generation, RCT = randomised controlled trial, SD = standard deviation, SMD = standardised mean difference, SR = systematic review

Table – Aerobic exercise (RA), results and quality assessment

Outcome (outcome measure)	Study (date) [study type]	Standardised result, SMD (95% CI) unless otherwise stated	Natural result	AMSTAR2 quality	Rand. Seq.	Alloc. Conc.	Blind. Part.	Blind. Asses.
Disease activity	Hernandez-Hernandez (2017) [SR][68]		1 MA[86] concluded that aerobic exercise does not improve disease activity	Critically low				
	Larkin (2014) [SR][70]		Weak positive correlations between disease activity and physical activity	Low				
	Katz (2018) [RCT][71]	<u>Exercise vs control at week 21</u><br>Pedometer vs control: SMD -0.15 (-0.65, 0.36)<br>Pedometer + sleep target vs control: SMD -0.26 (-0.76, 0.25)	<u>RADAI, BL / week 21, mean (SD)</u><br>Pedometer: 4.3 (2.0) / 3.8 (2.1)<br>Pedometer + sleep target: 4.4 (1.9) / 3.6 (1.9)<br>Control: 3.7 (2.0) / 4.1 (2.0)		H/UC	L	H/UC	H/UC
	Feldthusen (2016) [RCT][73]	<u>Exercise vs control at 12 weeks</u><br>SMD -0.16 (-0.63, 0.31)	<u>DAS28, BL / 12 weeks, mean (SD)</u><br>Exercise: 3.5 (1.1) / 3.0 (1.1)<br>Control: 3.2 (1.1) / 3.2 (1.4)		L	L	H/UC	L
	Sjoquist (2011) [RCT][74]	<u>Exercise vs control at 2 years</u><br>SMD 0.04 (-0.22, 0.30)	<u>DAS28, BL / 2 years, mean (SD)</u><br>Exercise: 3.29 (1.54) / 2.93 (1.71)<br>Control: 3.22 (1.45) / 2.87 (1.38)		L	H/UC	H/UC	L
	Li (2006) [RCT][76]	<u>Exercise education vs control at 6 months</u><br>SMD 0.13 (-0.25, 0.50)	<u>RADAI, BL / 6 months, mean (SD)</u><br>Exercise education: 5.08 (2.03) / 4.24 (2.26)<br>Control: 4.98 (1.99) / 3.97 (1.89)		L	H/UC	H/UC	H/UC
	Bespoke meta-analysis including[71;73;74]	<u>Exercise vs control</u><br>SMD 0.02 (-0.19, 0.23), I^2 0.0%						
Tender joints	Melikoglu (2006) [RCT][77]	<u>Aerobic exercise vs range of motion at 15 days</u><br>SMD -0.04 (-0.66, 0.58)	<u>Ritchie Index, BL / 15 days, mean (SD)</u><br>Aerobic exercise: 15.3 (10.1) / 11.1 (6.9)<br>Range of motion: 12.5 (11.6) / 11.4 (8.4)		H/UC	H/UC	H/UC	L
	Nordstrom (1996) [NRT][79]	<u>Exercise vs physio at 6 months</u><br>SMD 0.11 (-0.80, 1.03)	<u>Joint score index, BL / 6 months, mean (SD)</u><br>Exercise intervention: 8.7 (4.6) / 6.2 (4.7)<br>Physio: 5.7 (3.0) / 5.7 (3.5)					
	Noreau (1995) [NRT][81]	<u>Exercise vs control at 12 weeks</u><br>SMD 0.23 (-0.54, 1.00)	<u>Tender joint count, BL / 12 weeks, mean (SD)</u><br>Exercise: 3.11 (3.45) / 2.05 (1.84)<br>Control: 2.30 (1.49) / 1.60 (2.12)					

Alloc. Conc. = allocation concealment, AMSTAR2 = Assessing the Methodological Quality of Systematic Reviews, BL = baseline, Blind. Asses. = Blinded assessors, Blind. Part. = blinded participants, CI = confidence interval, DAS28 = Disease Activity Score 28, H/UC = high / unclear risk of bias, L = low risk of bias, MA = meta-analysis, NRT = non-randomised trial, RA rheumatoid arthritis, RADAI = rheumatoid arthritis diseae activity index, Rand. Seq. = random sequence generation, RCT = randomised controlled trial, SD = standard deviation, SMD = standardised mean difference, SR = systematic review

Table – Aerobic exercise (RA), results and quality assessment

Outcome (outcome measure)	Study (date) [study type]	Standardised result, SMD (95% CI) unless otherwise stated	Natural result	AMSTAR2 quality	Rand. Seq.	Alloc. Conc.	Blind. Part.	Blind. Asses.
Swollen joints	Hansen (1993) [RCT][78]	Exercise vs control at 2 years<br>Individual vs control: SMD -0.90 (-1.65, -0.15)<br>Physio vs control: SMD -0.96 (-1.71, -0.20)<br>Group vs control: SMD -0.48 (-1.21, 0.25)<br>Group + pool vs control: SMD -1.16 (-1.94, -0.39)	Swollen joint count, BL / 2 years, mean (SD †)<br>Individual exercise: 3.5 (1.3) / 3.4 (1.8)<br>Physio: 3.9 (1.0) / 3.3 (1.6)<br>Group training: 2.8 (1.3) / 4.5 (2.2)<br>Group + pool: 3.3 (0.9) / 2.8 (1.4)<br>Control: 3.7 (1.7) / 5.9 (3.5)		H/UC	H/UC	H/UC	L
	Noreau (1995) [NRT][81]	Exercise vs control at 12 weeks<br>SMD 0.04 (-0.73, 0.80)	Swollen joint count, BL / 12 weeks, mean (SD)<br>Exercise: 4.79 (4.40) / 4.53 (4.07)<br>Control: 4.80 (3.26) / 4.40 (2.76)					
Morning stiffness	Melikoglu (2006) [RCT][77]	Aerobic exercise vs range of motion at 15 days<br>SMD -0.35 (-0.97, 0.28)	Morning stiffness (mins), BL / 15 days, mean (SD)<br>Aerobic exercise: 38.7 (32.7) / 30.5 (19.2)<br>Range of motion: 44.7 (41.1) / 41.2 (39.5)		H/UC	H/UC	H/UC	L
	Hansen (1993) [RCT][78]	Exercise vs control at 2 years<br>Individual vs control: SMD -1.55 (-2.37, -0.73)<br>Physio vs control: SMD -0.36 (-1.08, 0.36)<br>Group vs control: SMD -1.98 (-2.87, -1.10)<br>Group + pool vs control: SMD -1.57 (-2.40, -0.75)	Morning stiffness, BL / 2 years, mean (SD †)<br>Individual exercise: 39.3 (14.1) / 24.5 (10.9)<br>Physio: 33.5 (16.1) / 46.0 (18.4)<br>Group training: 32.5 (14.4) / 17.5 (8.6)<br>Group + pool: 58.8 (18.7) / 25.0 (8.6)<br>Control: 53.3 (19.3) / 53.8 (24.4)		H/UC	H/UC	H/UC	L
QoL	Siegel (2017) [SR][69]		1 MA[86] concluded that aerobic exercise improves QoL	Moderate				
	Baxter (2016) [RCT][72]	Exercise vs control, change BL-6 weeks<br>SMD 0.95 (0.19, 1.71)	EuroQOL, change bl-6 weeks, mean (SD)<br>Exercise: 5.0 (4.8)<br>Control: -0.1 (5.6) p=0.71		L	L	H/UC	L
	Feldthusen (2016) [RCT][73]	Exercise vs control at 12 weeks<br>SMD 0.41 (-0.07, 0.88)	EQ5D, BL / 12 weeks, mean (SD)<br>Exercise: 52.3 (21.6) / 64.1 (18.1)<br>Control: 55.2 (20.1) / 57.0 (16.9)		L	L	H/UC	L
	Brodin (2008) [RCT][75]	Exercise vs control at 1 year<br>SMD 0.32 (0.05, 0.58)	EQ5D, BL / 1 year, mean (SD §)<br>Exercise: 60.8 (18.8) / 67.0 (15.7)<br>Control: 61.0 (18.5) / 61.5 (18.3)		L	H/UC	H/UC	L
	Bespoke meta-analysis including[72;73;75]	Exercise vs control<br>SMD 0.41 (0.15, 0.68), I^2 15.9%						

§ Mean (SD) estimated from median (range) using published formula[87]

† Mean (SD) estimated from median (interquartile range using published formula[87]

Alloc. Conc. = allocation concealment, AMSTAR2 = Assessing the Methodological Quality of Systematic Reviews, BL = baseline, Blind. Asses. = Blinded assessors, Blind. Part. = blinded participants, CI = confidence interval, H/UC = high / unclear risk of bias, L = low risk of bias, MA = meta-analysis, NRT = non-randomised trial, QoL = Quality of life, RA rheumatoid arthritis, Rand. Seq. = random sequence generation, RCT = randomised controlled trial, SD = standard deviation, SMD = standardised mean difference

Table – Aerobic exercise (RA), results and quality assessment

Outcome (outcome measure)	Study (date) [study type]	Standardised result, SMD (95% CI) unless otherwise stated	Natural result	AMSTAR2 quality	Rand. Seq.	Alloc. Conc.	Blind. Part.	Blind. Asses.
Patient global	Sjoquist (2011) [RCT][74]	Exercise vs control at 2 years<br>SMD 0.01 (-0.25, 0.28)	Patient global VAS, BL / 2 years, mean (SD §)<br>Exercise: 38.3 (17.6) / 32.0 (15.9)<br>Control: 34.5 (16.9) / 31.8 (17.9)		L	H/UC	H/UC	L
Anxiety	Feldthusen (2016) [RCT][73]	Exercise vs control at 12 weeks<br>SMD -0.71 (-1.19, -0.23)	HADS anxiety, BL / 12 weeks, mean (SD)<br>Exercise: 5.9 (3.4) / 4.5 (3.4)<br>Control: 7.0 (4.4) / 7.2 (4.2)		L	L	H/UC	L
Depression	Larkin (2014) [SR][70]		No correlation between depression and physical activity	Low				
	Katz (2018) [RCT][71]	Exercise vs control at week 21<br>Pedometer vs control: SMD 0.24 (-0.26, 0.75)<br>Pedometer + sleep target vs control:<br>SMD 0.43 (-0.07, 0.94)	PHQ-8, BL / week 21, mean (SD)<br>Pedometer: 9.2 (6.0) / 6.7 (4.9)<br>Pedometer + sleep target: 9.4 (5.3) / 7.3 (4.3)<br>Control: 7.4 (3.3) / 5.7 (2.8)		H/UC	L	H/UC	H/UC
	Feldthusen (2016) [RCT][73]	Exercise vs control at 12 weeks<br>SMD -0.54 (-1.02, -0.06)	HADS depression, BL / 12 weeks, mean (SD)<br>Exercise: 5.7 (3.2) / 4.3 (2.8)<br>Control: 6.3 (3.7) / 6.0 (3.5)		L	L	H/UC	L
	Bespoke meta-analysis including[71;73]	Exercise vs control<br>SMD -0.15 (-0.92, 0.62), I^2 79.6%						

§ Mean (SD) estimated from median (range) using published formula[87]

Alloc. Conc. = allocation concealment, AMSTAR2 = Assessing the Methodological Quality of Systematic Reviews, BL = baseline, Blind. Asses. = Blinded assessors, Blind. Part. = blinded participants, CI = confidence interval, H/UC = high / unclear risk of bias, HADS = Hospital Anxiety and Depression Scale, L = low risk of bias, MA = meta-analysis, PHQ-8 = Patient Health Questionnaire – 8, RA rheumatoid arthritis, Rand. Seq. = random sequence generation, RCT = randomised controlled trial, SD = standard deviation, SMD = standardised mean difference, SR = systematic review

Table – Aerobic exercise (RA), results and quality assessment

Outcome (outcome measure)	Study (date) [study type]	Standardised result, SMD (95% CI) unless otherwise stated	Natural result	AMSTAR2 quality	Rand. Seq.	Alloc. Conc.	Blind. Part.	Blind. Asses.
Fatigue	Rongen-van Dartel (2015) [MA][67]	Exercise vs no exercise<br>All studies: SMD -0.31 (-0.55, -0.06)<br>Low risk of bias: SMD -0.15 (-0.57, 0.27)<br>Unclear risk of bias: SMD -0.39 (-0.70, -0.09)		Moderate				
	Larkin (2014) [SR][70]		Negative correlation between fatigue and physical activity	Low				
	Katz (2018) [RCT][71]	Exercise vs control at week 21<br>Pedometer vs control: SMD 0.07 (-0.44, 0.57)<br>Pedometer + sleep target vs control: SMD -0.12 (-0.62, 0.38)	Fatigue Short form, BL / week 21, mean (SD)<br>Pedometer: 59.7 (6.4) / 56.6 (7.7)<br>Pedometer + sleep target: 60.3 (6.3) / 55.2 (7.1)<br>Control: 57.5 (7.5) / 56.1 (7.5)		H/UC	L	H/UC	H/UC
	Feldthusen (2016) [RCT][73]	Exercise vs control at 12 weeks<br>VAS: SMD -0.27 (-0.74, 0.20)<br>BRAF-MDQ: -0.66 (-1.14, -0.18)	VAS fatigue, BL / 12 weeks, mean (SD)<br>Exercise: 69.5 (17.1) / 45.5 (21.6)<br>Control: 66.9 (14.3) / 51.5 (23.0)<br>BRAF-MDQ, BL / 12 weeks, mean (SD)<br>Exercise: 37.7 (11.4) / 25.9 (11.5)<br>Control: 39.8 (10.6) / 33.1 (10.2)		L	L	H/UC	L
	Bespoke meta-analysis including[71;73]	Exercise vs control<br>SMD -0.30 (-1.01, 0.41), I^2 76.2%						

Alloc. Conc. = allocation concealment, AMSTAR2 = Assessing the Methodological Quality of Systematic Reviews, BL = baseline, Blind. Asses. = Blinded assessors, Blind. Part. = blinded participants, BRAF-MDQ = Bristol Rheumatoid Arthritis Fatigue – Multidimensional Questionnaire, CI = confidence interval, H/UC = high / unclear risk of bias, L = low risk of bias, MA = meta-analysis, NRT = non-randomised trial, RA rheumatoid arthritis, Rand. Seq. = random sequence generation, RCT = randomised controlled trial, SD = standard deviation, SMD = standardised mean difference, SR = systematic review, VAS = visual analogue scale

Table – Aerobic exercise (RA), results and quality assessment

Outcome (outcome measure)	Study (date) [study type]	Standardised result, SMD (95% CI) unless otherwise stated	Natural result	AMSTAR2 quality	Rand. Seq.	Alloc. Conc.	Blind. Part.	Blind. Asses.
Self-efficacy	Baxter (2016) [RCT][72]	Exercise vs control, change BL-6 weeks<br>SMD 1.06 (0.29, 1.83)	ASES, change bl-6 weeks, mean (SD)<br>Exercise: 22.6 (24.0)<br>Control: -3.5 (25.0) p=0.82		L	L	H/UC	L
	Feldthusen (2016) [RCT][73]	Exercise vs control at 12 weeks<br>SMD 0.53 (0.06, 1.01)	ASES, BL / 12 weeks, mean (SD)<br>Exercise: 61.4 (13.1) / 68.7 (15.8)<br>Control: 58.2 (15.9) / 60.0 (16.9)		L	L	H/UC	L
	Li (2006) [RCT][76]	Exercise education vs control at 6 months<br>Self-management: SMD 0.12 (-0.26, 0.50)<br>Disease management: SMD 0.04 (-0.34, 0.42)<br>Achieve outcomes: SMD -0.21 (-0.59, 0.16)	Self-efficacy, BL / 6 months, mean (SD)<br>*Self-management*<br>Exercise education: 6.41 (1.56) / 6.71 (1.72)<br>Control: 6.44 (1.62) / 6.50 (1.81)<br>*Disease management*<br>Exercise education: 6.69 (2.06) / 6.97 (1.82)<br>Control: 7.27 (1.56) / 6.90 (1.76)<br>*Achieve outcome*<br>Exercise education: 5.64 (1.95) / 5.74 (2.28)<br>Control: 6.08 (2.03) / 6.22 (2.21)		L	H/UC	H/UC	H/UC
	Bespoke meta-analysis including[72;73]	Exercise vs control<br>SMD 0.70 (0.22, 1.19), I^2 22.5%						
CRP	Melikoglu (2006) [RCT][77]	Aerobic exercise vs range of motion at 15 days<br>SMD 0.00 (-0.62, 0.62)	CRP, BL / 15 days, mean (SD)<br>Aerobic exercise: 0.4 (0.1) / 0.4 (0.1)<br>Range of motion: 0.4 (0.1) / 0.4 (0.1)		H/UC	H/UC	H/UC	L
	Nordstrom (1996) [NRT][79]	Exercise vs physio at 6 months<br>SMD -0.01 (-0.92, 0.90)	CRP, BL / 6 months, mean (SD)<br>Exercise intervention: 16.8 (15.2) / 15.8 (20.0)<br>Physio: 26.3 (14.3) / 16.0 (12.9)					
ESR	Melikoglu (2006) [RCT][77]	Aerobic exercise vs range of motion at 15 days<br>SMD 0.29 (-0.34, 0.91)	ESR, BL / 15 days, mean (SD)<br>Aerobic exercise: 23.2 (5.1) / 24.3 (6.1)<br>Range of motion: 21.2 (5.4) / 22.4 (7.2)		H/UC	H/UC	H/UC	L
	Hansen (1993) [RCT][78]	Exercise vs control at 2 years<br>Individual vs control: SMD -0.41 (-1.14, 0.31)<br>Physio vs control: SMD -1.06 (-1.83, -0.29)<br>Group vs control: SMD -0.41 (-1.14, 0.31)<br>Group + pool vs control: SMD -1.40 (-2.21, -0.60)	ESR, BL / 2 years, mean (SD †)<br>Individual exercise: 32.3 (9.5) / 25.8 (11.2)<br>Physio: 25.5 (5.2) / 20.8 (6.0)<br>Group training: 22.0 (10.3) / 25.0 (14.9)<br>Group + pool: 21.3 (3.7) / 17.5 (6.3)<br>Control: 22.5 (5.7) / 30.5 (11.5)		H/UC	H/UC	H/UC	L

† Mean (SD) estimated from median (interquartile range using published formula[87]

Alloc. Conc. = allocation concealment, AMSTAR2 = Assessing the Methodological Quality of Systematic Reviews, ASES = Arthritis Self-efficacy Scale, BL = baseline, Blind. Asses. = Blinded assessors, Blind. Part. = blinded participantsCI = confidence interval, CRP = C-Reactive Protein, ESR = erythrocyte sedimentation rate, H/UC = high / unclear risk of bias, L = low risk of bias, MA = meta-analysis, RA rheumatoid arthritis, Rand. Seq. = random sequence generation, RCT = randomised controlled trial, SD = standard deviation, SMD = standardised mean difference

Table – Aerobic exercise (RA), results and quality assessment

Outcome (outcome measure)	Study (date) [study type]	Standardised result, SMD (95% CI) unless otherwise stated	Natural result	AMSTAR2 quality	Rand. Seq.	Alloc. Conc.	Blind. Part.	Blind. Asses.
Grip strength	Larkin (2014) [SR][70]		No correlation between grip strength and physical activity	Low				
	Brodin (2008) [RCT][75]	<u>Exercise vs control at 1 year</u><br>SMD 0.44 (0.17, 0.71)	<u>Grip strength, BL / 1 year, mean (SD §)</u><br>Exercise: 466.3 (210.2) / 536.8 (243.1)<br>Control: 435.0 (206.9) / 440.8 (200.7)		L	H/UC	H/UC	L
	Minor (1995) [NRT][80]	<u>Exercise vs control at 12 months</u><br>0.28 (-0.33, 0.88)	<u>Grip strength at 12 months, mean (SD)</u><br>Exercise: 110 (60)<br>Control: 95 (49)					
Walk test	Ekblom (1975) [NRT][82]	<u>Exercise vs control (p2) at 6 months</u><br>1 vs p2: SMD -1.79 (-3.32, -0.25)<br>2 vs p2: SMD -1.78 (-3.09, -0.47)<br>3 vs p2: SMD -0.84 (-2.23, 0.55)	<u>850m walk test (mins), BL / 6 months, mean (SD)</u><br>1) 9.38 (2.87) / 8.11 (1.41)<br>2) 8.76 (1.57) / 7.99 (1.64)<br>3) 11.09 (1.15) / 9.11 (2.50)<br>p1) 8.43 (0.80) / 7.30 (0.13)<br>p2) 10.43 (1.58) / 11.03 (1.95)					
	Noreau (1995) [NRT][81]	<u>Exercise vs control at 12 weeks</u><br>SMD -0.06 (-0.82, 0.71)	<u>50ft walk, BL / 12 weeks, mean (SD)</u><br>Exercise: 6.37 (1.46) / 5.81 (1.40)<br>Control: 5.9 (0.8) / 5.9 (1.8)					
Radiological damage	Hansen (1993) [RCT][78]	<u>Exercise vs control at 2 years</u><br>Individual vs control: SMD -1.76 (-2.61, -0.91)<br>Physio vs control: SMD -1.66 (-2.49, -0.82)<br>Group vs control: SMD -1.82 (-2.68, -0.96)<br>Group + pool vs control: SMD -1.74 (-2.59, -0.89)	<u>Larsen score, BL / 2 years, mean (SD †)</u><br>Individual exercise: 43.0 (19.0) / 50.0 (20.1)<br>Physio: 47.5 (9.2) / 57.8 (14.1)<br>Group training: 41.8 (17.0) / 53.3 (16.4)<br>Group + pool: 42.0 (19.5) / 51.5 (19.0)<br>Control: 70.8 (8.9) / 77.5 (9.2)		H/UC	H/UC	H/UC	L
Work	Minor (1995) [NRT][80]	<u>Exercise vs control at 12 months</u><br>Hands: SMD 0.08 (-0.52, 0.69)<br>Lift: SMD 0.60 (-0.02, 1.22)<br>Legs: SMD 0.67 (0.05, 1.30)	<u>Work Capacity Evaluation at 12 months, mean (SD)</u><br>*<u>Hands</u>*<br>Exercise: 1.8 (1.1)<br>Control: 1.7 (1.3)<br>*<u>Lift</u>*<br>Exercise: 2.6 (0.5)<br>Control: 2.3 (0.5)<br>*<u>Legs</u>*<br>Exercise: 2.9 (0.2)<br>Control: 2.5 (0.8)					

† Mean (SD) estimated from median (interquartile range using published formula[87]

§ Mean (SD) estimated from median (range) using published formula[87]

Alloc. Conc. = allocation concealment, AMSTAR2 = Assessing the Methodological Quality of Systematic Reviews, BL = baseline, Blind. Asses. = Blinded assessors, Blind. Part. = blinded participants, CI = confidence interval, H/UC = high / unclear risk of bias, L = low risk of bias, NRT = non-randomised trial, RA rheumatoid arthritis, Rand. Seq. = random sequence generation, RCT = randomised controlled trial, SD = standard deviation, SMD = standardised mean difference

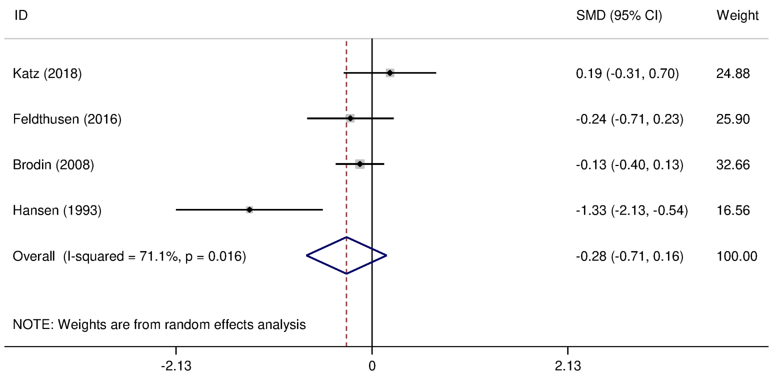

Figure – The effect of aerobic exercise on pain (RA)

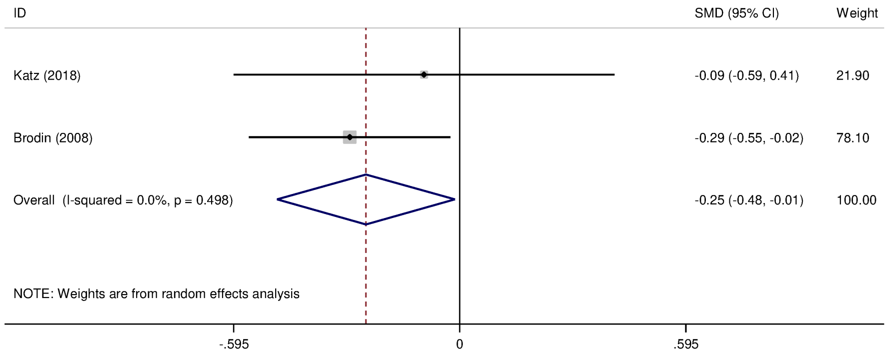

Figure – The effect of aerobic exercise on function (RA)

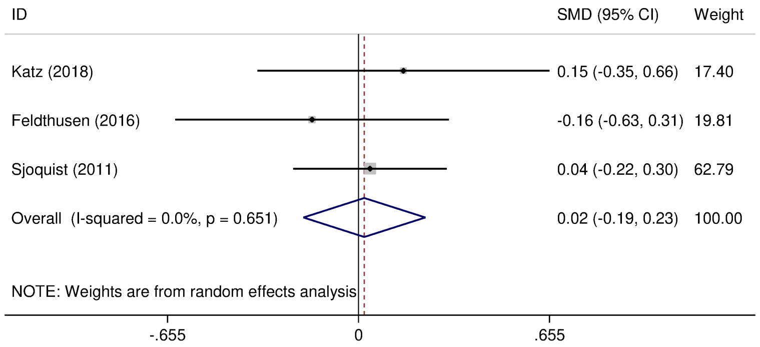

Figure – The effect of aerobic exercise on disease activity (RA)

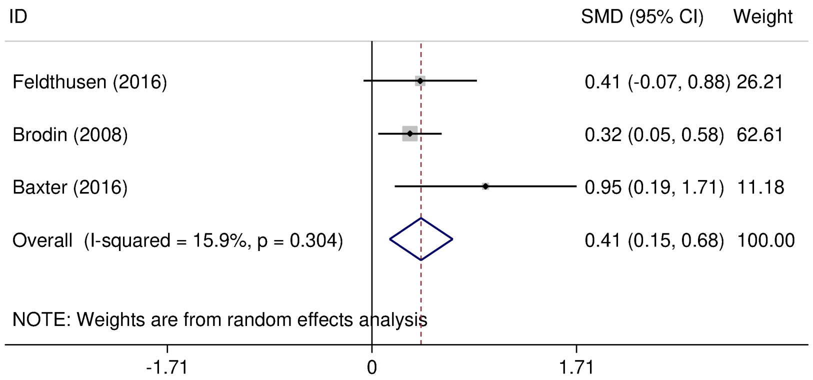

Figure – The effect of aerobic exercise on quality of life (RA)

Supplementary table 31 – Results from observational studies of aerobic exercise in RA

Table – Aerobic exercise (RA), results and quality assessment – observational studies

Outcome (outcome measure)	Study (date) [study type]	Standardised result, SMD (95% CI) unless otherwise stated	Natural result	Study Pop.	Attr.	Prog. Meas.	Outc. Meas.	Conf.	Stats.
Tender joints	Stenstrom (1994) [Prospective observational][85]		Ritchie Index at 4 years, mean (SD)<br>Low frequency exercise: 12 (8.9)<br>High frequency exercise: 12 (9.0)	M	M	M	L	M	M
ESR	Stenstrom (1994) [Prospective observational][85]		Ritchie Index at 4 years, mean (SD)<br>Low frequency exercise: 39 (21.7)<br>High frequency exercise: 31 (19.3)	M	M	M	L	M	M
Radiological damage	Stenstrom (1994) [Prospective observational][85]		Larsen score at 4 years, mean (SD)<br>Low frequency exercise: 83 (31.6)<br>High frequency exercise: 82 (48.4)	M	M	M	L	M	M
Comorbidity	Nadareishvili (2008) [Case-control][83]		Stroke, odds ratio (95% CI) [unadjusted]<br>Moderate or great vs low exercise: 1.29 (0.57, 2.91)	L	M	L	L	L	M
	Wolfe (2008) [Prospective observational][84]		Myocardial infarction, hazard ratio (95% CI) [adjusted for age & sex]<br>Aerobic exercise, yes vs no: 0.8 (0.6, 1.1)<br>First myocardial infarction, hazard ratio (95% CI) [adjusted for age & sex]<br>Aerobic exercise, yes vs no: 0.7 (0.5, 1.0)	L	M	M	L	H	L

Attr. = attrition, CI = confidence interval, Conf. = confounding, ESR = erythrocyte sedimentation rate, L = low risk of bias, M = moderate risk of bias, Outc. Meas = outcome measurement, Prog. Meas. = prognostic factor measurement, Pros. Obs. = prospective observational, RA = rheumatoid arthritis, Stats. = statistical analysis, Study Pop. = study population

Supplementary table 32 – Description of original studies of aerobic + muscle strengthening exercise in RA

Table – Aerobic + muscle strengthening exercise (RA), description of included studies

Author (date) [country]	Study design	Inclusion criteria	Exposure detail	N	Age years, mean (SD)	N (%) female	Funders
Lange (2019) [Sweden][88]	RCT	1987 ACR RA criteria, aged ≥65 years, symptom duration >2 years, DAS28 <5.1<br>Exclusions: unstable ischemic heart disease or arrhythmia that might preclude moderate intensity exercise, ongoing exercise of moderate-high intensity ≥2x per week, inability to speak/understand Swedish, inability to participate in physical testing involving walking/bicycling	1) Supervised exercise – aerobic and muscle strengthening, 2-3 sessions per week. 27 mins aerobic exercise at 70-89% max heart rate and 5 muscle strengthening exercises at 70-80% 1 repetition max<br>p) Individual meeting with physiotherapist where they were encouraged to follow the same exercise regime at home, but no gym-based exercise	1) 36<br>p) 38	1) 69.1 (2.6)<br>p) 70.1 (2.3)	1) 27 (75.0)<br>p) 29 (76.3)	**University** (University of Gothenburg), **Government** (Health and Medical Care Committee of the Regional Executive Board), **Hospital** (Sahlgrenska University Hospital), **Charity** (Swedish Rheumatism Association)
Durcan (2014) [Ireland][89]	RCT	1987 ACR RA criteria<br>Exclusions: Not independently mobile, live >1 hours from assessment centre, were deemed a falls risk or had severe medical conditions that were more limiting than arthritis: congestive heart failure with functional limitation, angina, active malignancy, uncontrolled thyroid disease, severe COPD, neurological conditions.	1) 12 week home program including aerobic, muscle strengthening and range of motion exercises<br>p) Standard care	1) 40<br>p) 38	1) 61 (8.0)<br>p) 59 (12)	1) 30 (75.0)<br>p) 20 (52.6)	**Not reported**
Breedland (2011) [The Netherlands][90]	RCT	1987 ACR RA criteria, aged 18-66 years<br>Exclusions: DAS28 > 5.1, cardiac or pulmonary diseases resulting in restrictions in ability to follow exercise program, functional class III-IV, no stable medication	1) Group exercise consisting of muscle exercise circuit and bicycle training for 60 mins and aqua jogging for 30 mins 2x per week<br>p) Waitlist control	1) 19<br>p) 15	1) 45 (11.9)<br>p) 51.8 (9.4)	1) 12 (63.2)<br>p) 12 (80.0)	**Charity** (Stichting Beatrixoord Noord-Nederland)
Hurkmans (2010) [The Netherlands][91]	RCT	1987 ACR RA, not physically active at moderate intensity, having a computer with internet, able to cycle on bicycle ergometer	Long term follow-up of van den Berg[92] (see below)	1) 56<br>p) 54	1) 50.6 (13.1)<br>p) 51.0 (10.9)	1) 43 (76.8)<br>p) 40 (74.1)	**Not reported** – authors declare no conflict of interest
Flint-Wagner (2009) [USA][93]	RCT	1987 ACR RA criteria, aged >18 years, functional class I-II, stable infliximab dose<br>Exclusions: participation in strength training exercise or an aerobic exercise regimen of 150 mins per week within past 3 months	1) 3x per week – walking warm up, strength training, aerobic exercise, abdominal exercises, cool down period<br>p) Usual care	1) 16<br>p) 8	1) 52.2 (13)<br>p) 49.0 (12.6)	19 (79.2)	**Industry** (Centocor, Inc.)

ACR = American College of Rheumatology, DAS28 = Disease Activity Score 28, int.= intervention, N = number, RA = rheumatoid arthritis, RCT = randomised controlled trial, SD = standard deviation, USA = United States of America

Table – Aerobic + muscle strengthening exercise (RA), description of included studies

Author (date) [country]	Study design	Inclusion criteria	Exposure detail	N	Age years, mean (SD)	N (%) female	Funders
Bulthuis (2007) [The Netherlands][94]	RCT	Admitted to hospital due to flare, or elective knee/hip replacement, 1987 ACR RA criteria, aged >18 years<br>Exclusions: serious cardiac disease, incapacitating pulmonary disease, serious hypertension (diastolic blood pressure >110 mmHg, pregnancy, insufficient understanding of Dutch, functional class IV	1) 3 weeks intensive exercise program at resort – training with physio, aerobic and muscle strengthening exercises. Also hydrotherapy<br>p) Usual care	1) 58<br>p) 40	1) 69 (12)<br>p) 67 (11)	1) 46 (79.3)<br>p) 31 (77.5)	**Charity** (The Dutch Arthritis Foundation), RVVZ (not defined in paper)
Neuberger (2007) [USA][95]	RCT	1987 ACR RA criteria, aged 40-70 years, communicate in English, ambulatory, no fibromyalgia or COPD, no beta-blockers or digitalis, not performing aerobic exercise 3x per week, having rheumatologist/physician approval to participate, meet criteria for aerobic fitness testing (no arrhythmias, recent myocardial infarction, acute infection, uncontrolled metabolic disease, known electrolyte abnormalities, or systolic BP 200 mm Hg or diastolic BP 115 mm Hg)	1) 12 weeks – low impact aerobic/ muscle strengthening exercise for 1hr 3x per week in a supervised group setting<br>2) Same as above but at home via videotape<br>p) No exercise control	1) 68<br>2) 79<br>p) 73	Median (range)<br>55.5 (40-70)	82.7% female	**Government** (National Institute of Nursing Research of the NIH)
van den Berg (2006) [The Netherlands][92]	RCT	1987 ACR RA criteria, not physically active for 30 mins in succession at moderate intensity on at least 5 days a week, availability of a computer with internet facilities, able to cycle of exercise bike, interested in study of physical activity, no cardiopulmonary problems	12 months of internet guided physical activity<br>1) individualised training – detailed physical activity program consisting of strengthening, range of motion, cycling on bike<br>2) general training intervention – had access to info about exercise, advised to complete recommended activity at least 5 days per week	1) 82<br>p) 78	1) 49.5 (12.9)<br>p) 49.8 (13.9)	1) 62 (75.6)<br>p) 60 (76.9)	**Charity** (ZONMw [Netherlands Organization for Health Research and Development], Dutch Arthritis Foundation)
Munneke (2005) [The Netherlands][96]	RCT	1987 ACR RA criteria, aged 20-70 years, 3 months stable DMARDs, functional class I-III, ability to cycle on home trainer, willingness to exercise bi-weekly, living in predefined area<br>Exclusions: inability to tolerate cardiorespiratory fitness training due to serious cardiac or lung disease, presence of one or more prostheses of weight bearing joints	1) Twice weekly session, 75 mins each, aimed to maintain cardiorespiratory and muscular fitness and flexibility<br>p) Usual care	1) 137<br>p) 140	Median (range)<br>1) 54 (46-61)<br>p) 54 (44-62)	1) 109 (79.6)<br>p) 112 (80.0)	**Government** (Dutch Health Car e Insurance Board)

ACR = American College of Rheumatology, BP = blood pressure, COPD = chronic obstructive pulmonary disorder, DMARDs = disease modifying anti-rheumatic drugs, int.= intervention, N = number, NIH = National Institutes for Health Research, RA = rheumatoid arthritis, RCT = randomised controlled trial, SD = standard deviation, USA = United States of America

Table – Aerobic + muscle strengthening exercise (RA), description of included studies

Author (date) [country]	Study design	Inclusion criteria	Exposure detail	N	Age years, mean (SD)	N (%) female	Funders
de Jong (2004) [The Netherlands][97]	RCT	1987 ACR RA criteria, aged 20-70 years, functional class I-III, stable DMARDs for 3 months, ability to ride exercise bike<br>Exclusions: cardiac/pulmonary disease precluding high-intensity exercise, prostheses of weight baring limbs, comorbidity strongly reducing life expectancy	1) High intensity exercise – groups, 2x per week, aiming to increase and maintain cardiovascular and muscle fitness – 3 parts: bike training, exercise circuit, sport of game<br>p) Usual care	1) 136<br>p) 145	Median (IQR)<br>1) 54 (46, 61)<br>p) 54 (45, 62)	1) 110 (80.9)<br>p) 115 (79.3)	**Government** (Dutch Health Care Insurance Board)
de Jong (2004) [The Netherlands][98]	RCT	See de Jong (2004)[97]	See de Jong (2004)[97]	1) 136<br>p) 145	1) 54 (16)<br>p) 54 (16)	1) 110 (81%)<br>p) 115 (79%)	**Not reported**
Hakkinen et al (2004) [Finland][99]	RCT	1987 ACR RA criteria, <2 years symptom duration, no treatment of DMARDs / prednisolone before inclusion	1) Strength training group – 2 years, home based, twice a week with moderate loads, encouraged to perform recreational physical activity (e.g. walking, skiing, cycling) 2x per week<br>p) Range of motion and stretching	1) 31<br>p) 31	1) 49 (10)<br>p) 49 (11)	1) 18 (58.1)<br>p) 20 (64.5)	**Government** (Central Finland Health Care District)
Hakkinen et al (2004) [Finland][100]	RCT	See above	See above	1) 31<br>p) 31	1) 49 (10)<br>p) 49 (11)	1) 18 (58.1)<br>p) 20 (64.5)	**Government** (Central Finland Health Care District)
de Jong (2003) [The Netherland][101]	RCT	See de Jong (2004)[97]	See de Jong (2004)[97]	1) 150<br>p) 150	1) 54.0 (16)<br>p) 53.5 (18)	1) 119 (79.3)<br>p) 118 (78.7)	**Government** (Dutch Health Care Insurance Board)
Westby (2000) [Canada][102]	RCT	1987 ACR RA criteria, symptom duration ≥1 year, functional class I-II, continuous low dose prednisone<br>Exclusions: fractures, significant cardiovascular disease, planned surgery, recent joint replacement (within 6 months, high dose prednisone (equivalent of 40mg/day ≥1 month	1) Attended education and given instructions for aerobic dance and strengthening program 3x per week for 45-60 mins<br>p) Asked to maintain previous level of exercise	1) 14<br>p) 16	1) 56.4 (10.1)<br>p) 56 (10.8)	1) 14 (100)<br>p) 16 (100)	**Charity** (British Columbia Health Research Foundation, The Arthritis Soceity)
Hakkinen (1999) [Finland][103]	RCT	See Hakkinen et al[99]	See Hakkinen et al[99]	1) 32<br>p) 33	1) 49.4 (10.1)<br>p) 49.0 (10.7)	1) 19 (59.4)<br>p) 21 (63.6)	**Government** (Central Finland Health Care District), **Charity** (Yrjo Jahnsson Foundation)

ACR = American College of Rheumatology, DMARDs = disease modifying anti-rheumatic drugs, int.= intervention, N = number, RA = rheumatoid arthritis, RCT = randomised controlled trial, SD = standard deviation

Table – Aerobic + muscle strengthening exercise (RA), description of included studies

Author (date) [country]	Study design	Inclusion criteria	Exposure detail	N	Age years, mean (SD)	N (%) female	Funders
Lyngberg (1994) [Denmark][104]	RCT	1987 ACR RA criteria, slight or moderate RA, 6 months steroid treatment (stable for 3 months, DMARDs stable for 4 months<br>Exclusions: heart disease, inability to perform exercises, patients with disease that may cause sudden death	1) Progressive interval training (exercise bicycle) then strengthening exercises (heel lifts, step climbing) and stretching<br>p) Non-exercise control	1) 12<br>p) 12	1) 65 (10.8)<br>p) 68 (7.2)	22 (91.7)	**Charity** (Danish Rheumatoid Arthritis Foundation, P. Carl Petersen Foundation, Grosserer A. V. Lykfeldt Foundation)
Ekdahl (1990) [Sweden][105]	RCT	Classical / definite RA involving joints of lower extremities, absence of other disease states that might influence results, functional class II, aged 20-65 years	1) Dynamic training aiming to improve strength, endurance, balance and aerobic capacity<br>2) Static training aiming to prevent limitations of joint mobility and promote muscle strength	67	53.0 (10.2)	43 (64.2)	**Charity** (Swedish Association Against Rheumatism, Signe and Reinhold Sund Foundation, Greta and Johan Kock Foundation), **Government** (Malmo County Council)
Lyngberg (1988) [Denmark][106]	RCT †	Definite or classical RA<br>Exclusions: Acute RA activity defined by the ARA, functional class III-IV and not able to use ergometerbicycle, not stable DMARDs/steroids for 6 months, not stable NSAIDs for 3 months, received intra-articular steroid injection less than 2 months ago, heart disease or non-acceptable exercise electrocardiogram	1) Tailored exercise program according to patients capacity – exercise consisted of aerobic exercise (exercise bike) and dynamic strength training<br>p) Control period	9	N 30-49 >50<br>4 / 5	7 (77.8)	**Charity** (Danish Rheumatoid Arthritis Foundation)
Nordemar (1981) [Sweden][107]	RCT	Definite or classical RA, moderate disease activity, functional stage I-III<br>Exclusions: severe disease	1) Exercise bike, also swimming, skiing, cycling, dancing, gymnastics, fast walking, jogging, and various organised spirts. Also participated in hospital exercise program led by physiotherapist – bike and strengthening exercises	1) 23<br>p) 23	1) 56 (9)<br>p) 58 (10)	1) 19 (82.6)<br>p) 19 (82.6)	**Charity** (King Gustaf V 80 years fund, Swedish National Association against Rheumatism), **Government** (Swedish Medical Research Council)

† Cross-over design

ACR = American College of Rheumatology, DMARDs = disease modifying anti-rheumatic drugs, HIV = human immunodeficiency virus, int.= intervention, N = number, RA = rheumatoid arthritis, RCT = randomised controlled trial, SD = standard deviation

Table – Aerobic + muscle strengthening exercise (RA), description of included studies

Author (date) [country]	Study design	Inclusion criteria	Exposure detail	N	Age years, mean (SD)	N (%) female	Funders
Nordemar (1981) [Sweden][108]	RCT	See above	See above	1) 23<br>p) 23	1) 56 (9)<br>p) 58 (10)	1) 19 (82.6)<br>p) 19 (82.6)	**Charity** (King Gustaf V 80 years fund, Swedish National Association against Rheumatism), **Government** (Swedish Medical Research Council)
Stavropoulos-Kalinoglou (2013) [UK][109]	NRT	1987 ACR RA criteria, sedentary lifestyle, stable medication for ≥3 months<br>Exclusions: joint surgery in preceding 6 months, amputation, co-morbidity incompatible with exercise	1) 6-month individualised exercise intervention – exercised in semi-supervised manner. Exercise included treadmills, cycle, rowing and some muscle strengthening training<br>p) Received verbal advice about cardiovascular and arthritis related benefits of exercise	1) 18<br>p) 18	1) 55.0 (9.8)<br>p) 52.8 (10.1)	1) 14 (77.8)<br>p) 14 (77.8)	**Hospital** (Dudley Group of Hospitals R&D Directorate cardiovascular programme grant), **University** (Wolverhampton University), **Charity** (Arthritis Research Campaign)
Lofgren (2018) [Sweden][110]	Single arm int.	Aged 18-75 years, HAQ<2, interested in participation, communicate in Swedish, not currently doing health enhancing physical activity	Health enhancing physical activity – 3 main components: ≥2 weekly strength training session, physical activity of at least moderate intensity for 30 mins, group support sessions	30	61 (10)	27 (90.0)	**Government** (Swedish Research Council, Strategic Research Program in Health Care Science)**, Charity** (§)**, Industry** (Combine Sweden), **University** (National Postgraduate School of Health Care Sciences)
Nordgren (2015) [Sweden][111]	Single arm int.	Aged 18-75 years, HAQ≤2, expressed interest in organised activity, communicate in Swedish	Three main components of program: (1) moderate intensity physical activity for ≥30 mins on most days of the week, (2) ≥2 weekly 45 min circuit training session including muscle strength training and aerobic exercises, (3) biweekly support groups	220	59 (8.8)	81%	**Government** (Swedish Research Council, Strategic Research Program in Health Care Sciences), **Charity** (Swedish Rheumatism Foundation, **University** (National Postgraduate School of Health Care Sciences)

ACR = American College of Rheumatology, Health Assessment Questionnaire, int.= intervention, N = number, NRT = non-randomised trial, RA = rheumatoid arthritis, RCT = randomised controlled trial, SD = standard deviation, UK = United Kingdom

Table – Aerobic + muscle strengthening exercise (RA), description of included studies

Author (date) [country]	Study design	Inclusion criteria	Exposure detail	N	Age years, mean (SD)	N (%) female	Funders
Di Gioia (2013) [Italy][112]	Single arm int.	1987 ACR RA criteria, non-response to DMARDs and treated with biologics for ≥12 months, DAS28>5.1<br>Exclusions: aged <18 years, RA functional class <II, intolerance to exercise, cognitive impairment, unstable anti-inflammatory/anti-rheumatic therapy, cachexia related to other diseases (cancer or HIV), instable medical conditions, pregnancy	Comprehensive rehabilitation provided by physicians, bio-engineers, occupational therapist, psychologist, exercise physiologist – included physical therapy with exercise aiming to improve aerobic fitness, muscle strength, mobility and balance	32	62.6 (13.4)	32 (100)	**Not reported** – authors declare no conflicts of interest
Strasser (2011) [Austria][113]	Single arm int.‡	1987 ACR RA criteria, symptom duration >2 years, stable medication for 3 months<br>Exclusions: Participation in another study, cardiac arrhythmia, recent myocardial infarction, stroke, cancer, hypertension	6 month supervised combined strength and endurance training program	20	59.3 (7.9)	19 (95.0)	**Charity** (Jubiläumsfonds of the Austrian National Bank)
van der Giesen (2010) [The Netherlands][114]	Single arm int.	RA patients<br>Exclusions: weight bearing prostheses or comorbidity	Rheumatoid Arthritis Patients in Training (RAPIT) program – supervised aerobic and strengthening exercises, 2x per week	150	51 (12)	121 (80.7)	**Charity** (Dutch Arthritis Association)
de Jong (2009) [The Netherlands][115]	Single arm int.	1987 ACR RA criteria, aged 20-70 years, stable DMARDs for 3 months, functional class I-III, ability to cycle on a home trainer, willingness to do bi-weekly exercise, living within 20km of assessment centre<br>Exclusions: inability to tolerate cardiorespiratory fitness training due to cardiac or lung disease, presence of one or more prostheses of weight-bearing joints	1.25 hour duration sessions consisting of bike training, exercises circuits aiming to improve muscle strength and aerobic capacity, and a sports and games session	71	56 (15)	61 (85.9)	**Charity** (Vrienden van Sole Mio foundation)
Neuberger (1997) [USA][116]	Single arm int.	Diagnosis of RA, mentally competent, able to read/speak English, able to ambulate, no history of fibromyalgia or severe COPD, no involved in regular exercise program, rheumatologist's approval to joint study	Low impact exercises for 1 hr, 3x per week. Class consisted of 4 phases: warm-up, strengthening, low-impact aerobic exercises, cool down.<br>High participation: 31-36 sessions<br>Moderate participation: 25-30 sessions<br>Low participation: ≤24 sessions	25	55 (range: 30-71)	14 (56.0)	**Government** (National Institute of Nursing Research of the NIH)

‡ Strasser et al had a control group, but none of the outcomes relevant to this review were reported for this group

ACR = American College of Rheumatology, COPD = chronic obstructive pulmonary disorder, DAS28 = Disease Activity Score 28, HIV = human immunodeficiency virus, int.= intervention, N = number, NIH = National Institutes for Health Research, RA = rheumatoid arthritis, RCT = randomised controlled trial, SD = standard deviation, USA = United States of America

Supplementary table 33 – Results from interventional studies of aerobic + muscle strengthening exercise in RA

Table – Aerobic + muscle strengthening exercise (RA), results and quality assessment

Outcome (outcome measure)	Study (date) [study type]	Standardised result, SMD (95% CI) unless otherwise stated	Natural result	AMSTAR2 quality	Rand. Seq.	Alloc. Conc.	Blind. Part.	Blind. Asses.
Pain	Durcan (2014) [RCT][89]	Exercise vs control at 12 weeks<br>SMD -0.78 (-1.24, -0.32)	Pain, BL / 12 weeks, mean (SD)<br>Exercise: 29 (21.5) / 21 (18)<br>Control: 41.4 (25.5) / 39.8 (29.3)		L	H/UC	H/UC	H/UC
	Flint-Wagner (2009) [RCT][93]	Exercise vs control, change BL-16 weeks<br>SMD -0.75 (-1.63, 0.13)	Pain VAS, change BL-16 weeks, mean (SD)<br>Exercise: -14.8 (19.2)<br>Control: -0.13 (20.1), p=0.07		H/UC	H/UC	H/UC	H/UC
	Neuberger (2007) [RCT][95]	Group exercise vs control<br>SMD -0.09 (-0.42, 0.24)<br>Home exercise vs control<br>SMD -0.05 (-0.37, 0.27)	McGill Pain, BL / 12 weeks, mean (SD)<br>Group exercise: 4.7 (2.1) / 4.1 (2.2)<br>Home exercise: 3.9 (1.9) / 4.2 (1.9)<br>Control: 4.1 (2.3) / 4.3 (2.3)		L	H/UC	H/UC	L
	Hakkinen (2004) [RCT][99]	Exercise vs control at 2 years<br>SMD -0.57 (-1.07, -0.06)	Pain VAS, BL / 2 years / 5 years, mean (SD)<br>Exercise: 41.7 (19.5) / 13.7 (16.2) / 22.0 (19.9)<br>Control: 41.3 (27.1) / 24.9 (22.8) / 25.9 (24.2)		H/UC	H/UC	H/UC	H/UC
	Hakkinen (1999) [RCT][103]	Exercise vs control, change BL-12 months<br>SMD -0.11 (-0.59, 0.38)	Pain VAS, change BL – 12 months, mean (SD †)<br>Exercise: -20.4 (28.6)<br>Control: -17.2 (31.9)		H/UC	H/UC	H/UC	H/UC
	Lyngberg (1994) [RCT][104]	Exercise vs control at 3 months<br>SMD -0.11 (-0.91, 0.69)	Joint pain, BL / 3 months, mean (SD §)<br>Exercise: 12.3 (8.3) / 15.0 (8.0)<br>Control: 11.5 (8.0) / 16 (10.4)		H/UC	H/UC	H/UC	L
	Ekdahl (1990) [RCT][105]		Pain VAS, change BL-6 weeks, mean<br>Dynamic: -0.5<br>Static: -0.2		H/UC	H/UC	H/UC	L
	Bespoke meta-analysis including[89;93;95;103;104]	Exercise vs control<br>SMD -0.25 (-0.52, 0.01), I^2 44.8%						
	Lofgren (2018) [single arm int.][110]		Pain, BL / 1 year / 2 years, mean (SD ‡)<br>11.7 (18.7) / 12.0 (17.1) / 8.7 (15.6)					
	Nordgren (2015) [single arm int.][111]		Pain VAS, change BL-1 years, mean (SD ⱡ)<br>-3.54 (23.6)					
	Strasser (2011) [single arm int.][113]		Pain VAS, BL / 6 months, mean (SD)<br>33.33 (21.60) / 25.86 (19.78)					
	Neuberger (1997) [single arm int.][116]		Pain, BL / 12 weeks, mean<br>5.09 / 4.50					

† SD calculated from 95% CI, ⱡ SD calculated from standard error, ‡ mean (SD) estimated from median (interquartile range) using a published formula[87], § mean (SD) estimated from median (range) using a published formula[87]

Alloc. Conc. = allocation concealment, AMSTAR2 = Assessing the Methodological Quality of Systematic Reviews, BL = baseline, Blind. Asses. = Blinded assessors, Blind. Part. = blinded participants, CI = confidence intervalH/UC = high / unclear risk of bias, L = low risk of bias, RA = rheumatoid arthritis, Rand. Seq. = random sequence generation, RCT = randomised controlled trial, SD = standard deviation, SMD = standardised mean difference, VAS = visual analogue scale

Table –Aerobic + muscle strengthening exercise (RA), results and quality assessment

Outcome (outcome measure)	Study (date) [study type]	Standardised result, SMD (95% CI) unless otherwise stated	Natural result	AMSTAR2 quality	Rand. Seq.	Alloc. Conc.	Blind. Part.	Blind. Asses.
Function	Lange (2019) [RCT][88]	Exercise vs control at 20 weeks<br>SMD -0.24 (-0.70, 0.22)	HAQ, change BL-20 weeks, mean (SD)<br>Exercise: -0.063 (0.16)<br>Control: -0.0097 (0.27)		L	L	H/UC	L
	Durcan (2014) [RCT][89]	Exercise vs control at 12 weeks<br>SMD -0.50 (-0.95, -0.04)	HAQ, BL / 12 weeks, mean (SD)<br>Exercise: 0.8 (0.4) / 0.5 (0.5)<br>Control: 0.9 (0.4) / 0.8 (0.7)		L	H/UC	H/UC	H/UC
	Breedland (2011) [RCT][90]	Exercise vs control at 9 weeks<br>SMD -0.10 (-0.77, 0.58)	AIMS2 physical, BL / 9 weeks, mean (SD)<br>Exercise: 1.95 (1.22) / 1.27 (1.05)<br>Control: 1.51 (1.14) / 1.37 (1.05)		L	L	H/UC	L
	Hurkmans (2010) [RCT][91]	Exercise vs control, change BL-2 years<br>SMD -0.03 (-0.40, 0.35)	HAQ, change BL-2 years, mean (SD †)<br>Individualised exercise: -0.04 (0.40)<br>Exercise guidance: -0.03 (0.37)		L	L	H/UC	L
	Flint-Wagner (2009) [RCT][93]	Exercise vs control, change BL-16 weeks<br>SMD -0.75 (-1.63, 0.13)	HAQ, change BL-16 weeks, means (SD)<br>Exercise: -0.4 (0.4)<br>Control: -0.1 (0.4) p=0.17		H/UC	H/UC	H/UC	H/UC
	Bulthuis (2007) [RCT][94]	Exercise vs control at 3 weeks<br>SMD -0.28 (-0.68, 0.13)	HAQ, BL / 3 weeks / 52 weeks, mean (SD †)<br>Exercise: 1.47 (0.6) / 1.21 (0.8) / 0.77(0.6)<br>Control: 1.47 (0.6) / 1.41 (0.6) / 0.87 (0.5)		L	L	H/UC	H/UC
	van den Berg (2006) [RCT][92]	Exercise vs control, change BL-12 months<br>SMD -0.15 (-0.46, 0.17)	HAQ, change Bl-12 months, mean (SD †)<br>Individualised exercise: -0.09 (0.35)<br>Exercise guidance: -0.04 (0.34)		L	L	H/UC	L
	Hakkinen (2004) [RCT][99]	Exercise vs control at 2 years<br>SMD -0.21 (-0.71, 0.29)	HAQ, BL / 2 years / 5 years, mean (SD)<br>Exercise: 0.60 (0.53) / 0.13 (0.21) / 0.30 (0.42)<br>Control: 0.77 (0.55) / 0.35 (0.45) / 0.40 (0.51)		H/UC	H/UC	H/UC	H/UC
	de Jong (2003) [RCT][101]	Exercise vs control, change BL-24 months<br>SMD -0.20 (-0.43, 0.03)	HAQ, change BL-24 months, mean (SD)<br>Exercise: 0.00 (0.4)<br>Control: 0.07 (0.3)		L	L	H/UC	L
	Westby (2000) [RCT][102]	Exercise vs control at 1 year<br>SMD -0.46 (-1.19, 0.27)	HAQ, BL / 1 year, mean (SD)<br>Exercise: 1.2 (0.6) / 1.0 (0.6)<br>Control: 1.5 (0.7) / 1.3 (0.7)		H/UC	H/UC	H/UC	L
	Hakkinen (1999) [RCT][103]	Exercise vs control, change BL-12 months<br>SMD -0.10 (-0.59, 0.39)	HAQ, change BL – 12 months, mean (SD †)<br>Exercise: -3.2 (4.0)<br>Control: -2.8 (4.0)		H/UC	H/UC	H/UC	H/UC

† SD calculated from 95% CI,
Alloc. Conc. = allocation concealment, AMSTAR2 = Assessing the Methodological Quality of Systematic Reviews, BL = baseline, Blind. Asses. = Blinded assessors, Blind. Part. = blinded participants, CI = confidence interval, H/UC = high / unclear risk of bias, L = low risk of bias, RA = rheumatoid arthritis, RADAI = Rheumatoid Arthritis Disease Activity Index, Rand. Seq. = random sequence generation, RCT = randomised controlled trial, SD = standard deviation, SMD = standardised mean difference

Table –Aerobic + muscle strengthening exercise (RA), results and quality assessment

Outcome (outcome measure)	Study (date) [study type]	Standardised result, SMD (95% CI) unless otherwise stated	Natural result	AMSTAR2 quality	Rand. Seq.	Alloc. Conc.	Blind. Part.	Blind. Asses.
Function	Lyngberg (1994) [RCT][104]	Exercise vs control at 3 months<br>SMD -0.32 (-1.12, 0.49)	Fries Index, BL / 3 months, mean (SD §)<br>Exercise: 18.0 (7.3) / 15.8 (9.5)<br>Control: 16.8 (11.3) / 19.8 (15.0)		H/UC	H/UC	H/UC	L
	Nordemar (1981) [RCT][108]		Activities of daily living, N can / cannot at follow-up after 8 years<br>Activities that did not differ: wash hair, wash face, intimate hygiene, wash feet, toilet, socks on-off, shirt on-off, trousers on-off, do up buttons, rise from lying to standing, walk on level group, eat with knife and fork, cook, wash dishes, make the bed, use scissors, pick up object from floor, write letter<br>Activities that did differ:<br>walk upstairs and downstairs: 1) 22 / 1; p) 13 / 8 p=0.007<br>go shopping: 1) 17 / 3; p) 13 / 9, p=0.06<br>clean house: 1) 18 / 3; p) 9 / 13, p=0.003<br>use public transport: 1) 17 / 5; p) 11 / 12, p=0.04<br>take object from shelf: 1) 23 / 0; p) 16 / 6 p=0.009		H/UC	H/UC	H/UC	H/UC
	Bespoke meta-analysis including[88-90;92-94;101-104]	Exercise vs control<br>SMD -0.24 (-0.37, -0.11), I^2 0%						
	Stavropoulos-Kalinoglou (2013) [NRT][109]	Exercise vs control at 3 months<br>SMD -1.09 (-1.79, -0.38)	HAQ, BL / 3 months / 6 months, mean (SD)<br>Exercise: 1.4 (0.8) / 1.0 (0.6) / 0.9 (0.6)<br>Control: 1.3 (0.7) / 1.6 (0.5) / 1.5 (0.6)					
	Nordgren (2015) [single arm int.][111]		HAQ, change BL-1 years, mean (SD ‽)<br>-0.20 (0.30)					
	Di Gioia (2013) [single arm int.][112]		HAQ, BL / 9 months, mean (SD)<br>2.42 (0.43) / 2.19 (0.38)					
	Strasser (2011) [single arm int.][113]		HAQ, BL / 6 months, mean (SD)<br>1.23 (0.80) / 1.01 (0.67)					
	van der Giesen (2010) [single arm int.][114]		HAQ, change BL-12 months, mean (SD †)<br>-0.06 (0.75)					
	de Jong (2009) [single arm int.][115]		MACTAR, BL / 18 months, median (net IQR)<br>Exercise: 58 (12.2) / 59 (9.0)					

† SD calculated from 95% CI, ‽ SD calculated from standard error, § mean (SD) estimated from median (range) using a published formula[87]

Alloc. Conc. = allocation concealment, AMSTAR2 = Assessing the Methodological Quality of Systematic Reviews, BL = baseline, Blind. Asses. = Blinded assessors, Blind. Part. = blinded participants, CI = confidence interval, DAS = Disease Activity Score, H/UC = high / unclear risk of bias, IQR = interquartile range, L = low risk of bias, MACTAR = McMasters Toronto Patient Preference Disability Questionnaire, NRT = non-randomised trial, RA = rheumatoid arthritis, Rand. Seq. = random sequence generation, RCT = randomised controlled trial, SD = standard deviation, SMD = standardised mean difference

Table –Aerobic + muscle strengthening exercise (RA), results and quality assessment

Outcome (outcome measure)	Study (date) [study type]	Standardised result, SMD (95% CI) unless otherwise stated	Natural result	AMSTAR2 quality	Rand. Seq.	Alloc. Conc.	Blind. Part.	Blind. Asses.
Disease activity	van den Berg (2006) [RCT][92]	Exercise vs control, change BL-12 months<br>SMD 0.09 (-0.22, 0.40)	DAS28, change Bl-12 months, mean (SD †)<br>Individualised exercise: -0.4 (1.2)<br>Exercise guidance: -0.5 (1.1)		L	L	H/UC	L
	Hakkinen (2004) [RCT][99]	Exercise vs control at 2 years<br>SMD -0.42 (-0.92, 0.09)	DAS28, BL / 2 years / 5 years, mean (SD)<br>Exercise: 4.4 (1.1) / 2.2 (1.2) / 2.3 (1.0)<br>Control: 9 [sic] (1.1) / 2.7 (1.2) / 3.0 (1.2)		H/UC	H/UC	H/UC	H/UC
	de Jong (2003) [RCT][101]	Exercise vs control, change BL-12 months<br>SMD -0.17 (-0.40, 0.05)	DAS4, change BL-24 months, mean (SD)<br>Exercise: -0.9 (1.2)<br>Control: -0.7 (1.1)		L	L	H/UC	L
	Hakkinen (1999) [RCT][103]	Exercise vs control, change BL-12 months<br>SMD -0.05 (-0.53, 0.44)	DAS28, change BL – 12 months, mean (SD †)<br>Exercise: -2.2 (1.2)<br>Control: -2.0 (5.9)		H/UC	H/UC	H/UC	H/UC
	Bespoke meta-analysis including[92;101;103]	Exercise vs control<br>SMD -0.08 (-0.25, 0.09), I^2 0%						
	Stavropoulos-Kalinoglou (2013) [NRT][109]	Exercise vs control at 3 months<br>SMD -0.28 (-0.94, 0.37)	DAS28, BL / 3 months / 6 months, mean (SD)<br>Exercise: 3.5 (1.2) / 2.9 (0.8) / 2.7 (0.7)<br>Control: 3.1 (1.2) / 3.1 (0.6) / 3.2 (0.9)					
	Di Gioia (2013) [single arm int.][112]		DAS28, BL / 9 months, mean (SD)<br>5.98 (0.5) / 5.3 (0.69)					
	Strasser (2011) [single arm int.][113]		DAS28, BL / 6 months, mean (SD)<br>3.57 (1.10) / 3.12 (1.27)					
	van der Giesen (2010) [single arm int.][114]		RADAI, change BL-12 months, mean (SD †)<br>-0.4 (3.4)					
	de Jong (2009) [single arm int.][115]		DAS4, BL / 18 months, median (net IQR)<br>Exercise: 2.59 (2.3) / 2.77 (1.09)					

† SD calculated from 95% CI

Alloc.. Conc. = allocation concealment, AMSTAR2 = Assessing the Methodological Quality of Systematic Reviews, BL = baseline, Blind. Asses. = Blinded assessors, Blind. Part. = blinded participants, CI = confidence interval, DAS = Disease Activity Score, H/UC = high / unclear risk of bias, IQR = interquartile range, L = low risk of bias, NRT = non-randomised trial, RA = rheumatoid arthritis, RADAI = Rheumatoid Arthritis Disease Activity Index, Rand. Seq. = random sequence generation, RCT = randomised controlled trial, SD = standard deviation, SMD = standardised mean difference

Table –Aerobic + muscle strengthening exercise (RA), results and quality assessment

Outcome (outcome measure)	Study (date) [study type]	Standardised result, SMD (95% CI) unless otherwise stated	Natural result	AMSTAR2 quality	Rand. Seq.	Alloc. Conc.	Blind. Part.	Blind. Asses.
Tender joints	Neuberger (2007) [RCT][95]	Group exercise vs control<br>SMD -0.15 (-0.48, 0.18)<br>Home exercise vs control<br>SMD -0.46 (-0.78, -0.14)	Tender joint count (164), BL / 12 weeks, mean (SD)<br>Group exercise: 32.2 (29.1) / 31.0 (29.2)<br>Home exercise: 29.0 (23.1) / 23.7 (23.1)<br>Control: 37.1 (25.1) / 35.1 (26.7)		L	H/UC	H/UC	L
	Di Gioia (2013) [single arm int.][112]		Tender joint count, BL / 9 months, mean (SD)<br>18.63 (3.4) / 11.69 (3.47)					
Swollen joints	Westby (2000) [RCT][102]	Exercise vs control at 1 year<br>SMD -1.05 (-1.82, -0.28)	Active joint count, BL / 1 year, mean (SD)<br>Exercise: 17.5 (12.3) / 16.2 (12.3)<br>Control: 30.4 (12.5) / 31 (15.5)		H/UC	H/UC	H/UC	L
	Lyngberg (1988) [RCT][106]		Swollen joint count, BL / 16 weeks, mean<br>Exercise: 77 / 56<br>Control: 42 / 49		L	H/UC	H/UC	L
	Nordemar (1981) [RCT][107]	Exercise vs control, change BL-8 years<br>SMD -1.46 (-2.11, -0.80)	Lansbury's index, change BL-8 years, mean (SD)<br>Exercise: -35 (21)<br>Control: -9 (14)		H/UC	H/UC	H/UC	H/UC
	Bespoke meta-analysis including[102;107]	Exercise vs control<br>SMD -1.53 (-2.46, -0.61), I^2 68%						
	Di Gioia (2013) [single arm int.][112]		Swollen joint count, BL / 9 months, mean (SD)<br>13.13 (3.96) / 6.94 (2.9)					
Stiffness	Durcan (2014) [RCT][89]	Exercise vs control at 12 weeks<br>SMD -0.65 (-1.11, -0.20)	Stiffness, BL / 12 weeks, mean (SD)<br>Exercise: 32 (23) / 24 (24)<br>Control: 43.8 (23.7) / 42.4 (32.2)		L	H/UC	H/UC	H/UC
Morning stiffness	Hakkinen (2004) [RCT][99]	Exercise vs control at 2 years<br>SMD -0.62 (-1.13, -0.11)	Morning stiffness, BL / 2 years / 5 years, mean (SD)<br>Exercise: 72.4 (54.5) / 16.3 (21.3) / 32.7 (55.2)<br>Control: 81.5 (90.4) / 37.7 (43.8) / 34.9 (49.9)		H/UC	H/UC	H/UC	H/UC
QoL	Hurkmans (2010) [RCT][91]	Exercise vs control, change BL-2 years<br>SMD -0.33 (-0.70, 0.05)	RAQoL, change BL-2 years, mean (SD †)<br>Individualised exercise: -1.3 (5.0)<br>Exercise guidance: 0.2 (4.1)		L	L	H/UC	L
	van den Berg (2006) [RCT][92]	Exercise vs control, change BL-12 months<br>SMD -0.17 (-0.48, 0.14)	RAQOL, change Bl-12 months, mean (SD †)<br>Individualised exercise: -1.3 (4.6)<br>Exercise guidance: -0.6 (3.6)		L	L	H/UC	L
	Nordgren (2015) [single arm int.][111]		EQ5D, change BL-1 years, mean (SD Ᵽ)<br>5.29 (19.1)					

† SD calculated from 95% CI, Ᵽ SD calculated from standard error

Alloc. Conc. = allocation concealment, AMSTAR2 = Assessing the Methodological Quality of Systematic Reviews, BL = baseline, Blind. Asses. = Blinded assessors, Blind. Part. = blinded participants, CI = confidence interval, H/UC = high / unclear risk of bias, L = low risk of bias, QoL = quality of life, RA = rheumatoid arthritisRand. Seq. = random sequence generation, RAQOL = Rheumatoid Arthritis Quality of Life Index, RCT = randomised controlled trial, SD = standard deviation, SMD = standardised mean difference

Table –Aerobic + muscle strengthening exercise (RA), results and quality assessment

Outcome (outcome measure)	Study (date) [study type]	Standardised result, SMD (95% CI) unless otherwise stated	Natural result	AMSTAR2 quality	Rand. Seq.	Alloc. Conc.	Blind. Part.	Blind. Asses.
Patient global	Di Gioia (2013) [single arm int.][112]		Patient global, BL / 9 months, mean (SD)<br>29.44 (9.08) / 48.39 (6.3)					
	Strasser (2011) [single arm int.][113]		Patient global VAS, BL / 6 months, mean (SD)<br>36.33 (21.25) / 25.20 (21.44)					
	Neuberger (1997) [single arm int.][116]		Arthritis impact, BL / 12 weeks, mean<br>3.27 / 2.51					
Fatigue	Durcan (2014) [RCT][89]	Exercise vs control at 12 weeks<br>SMD -0.52 (-0.97, -0.06)	Fatigue severity scale, BL / 12 weeks, mean (SD)<br>Exercise: 29.5 (17.8) / 21.4 (18.8)<br>Control: 30.5 (15.4) / 30.6 (16.8)		L	H/UC	H/UC	H/UC
	Neuberger (2007) [RCT][95]	Group exercise vs control<br>SMD -0.02 (-0.35, 0.31)<br>Home exercise vs control<br>SMD -0.16 (-0.48, 0.16)	Global fatigue, BL / 12 weeks, mean (SD)<br>Group exercise: 24.9 (10.3) / 20.7 (11.6)<br>Home exercise: 20.1 (10.2) / 19.2 (10.6)<br>Control: 21.9 (9.8) / 20.9 (11.2)		L	H/UC	H/UC	L
	Nordemar (1981) [RCT][108]		Fatigue, yes / no after 8 years<br>Exercise: 12 / 10<br>Control: 16 / 7, p = non-significant		H/UC	H/UC	H/UC	H/UC
	Nordgren (2015) [single arm int.][111]		Fatigue VAS, change BL-1 years, mean (SD ‡)<br>-2.29 (23.3)					
	Neuberger (1997) [single arm int.][116]		Multidimensional assessment of fatigue, BL / 12 weeks, mean<br>*Distress*:<br>High: 3.25 / 2.63<br>Mod: 2.69 / 2.13<br>Low: 2.11 / 2.89<br>*Severity:*<br>High: 4.03 / 3.38<br>Mod: 3.78 / 2.69<br>Low: 3.78 / 4.22<br>*Timing:*<br>High: 2.06 / 1.81<br>Mod: 2.22 / 1.44<br>Low: 2.03 / 2.28<br>*Global:*<br>High: 19.9 / 17.1<br>Mod: 18.5 / 13.7<br>Low: 17.5 / 19.4					

‡ SD calculated from standard error

Alloc. Conc. = allocation concealment, AMSTAR2 = Assessing the Methodological Quality of Systematic Reviews, BL = baseline, Blind. Asses. = Blinded assessors, Blind. Part. = blinded participants, CI = confidence interval, H/UC = high / unclear risk of bias, L = low risk of bias, RA = rheumatoid arthritis, Rand. Seq. = random sequence generation, RCT = randomised controlled trial, SD = standard deviation, SMD = standardised mean difference, VAS = visual analogue scale

Table –Aerobic + muscle strengthening exercise (RA), results and quality assessment

Outcome (outcome measure)	Study (date) [study type]	Standardised result, SMD (95% CI) unless otherwise stated	Natural result	AMSTAR2 quality	Rand. Seq.	Alloc. Conc.	Blind. Part.	Blind. Asses.
Anxiety	van der Giesen (2010) [single arm int.][114]		HADS-A, change BL-12 months, mean (SD †)<br>0.3 (4.4)					
Depression	Breedland (2011) [RCT][90]	Exercise vs control at 9 weeks<br>SMD -0.12 (-0.79, 0.56)	AIMS2 psychological, BL / 9 weeks, mean (SD)<br>Exercise: 2.47 (1.78) / 2.12 (1.58)<br>Control: 2.21 (1.27) / 2.29 (1.31)		L	L	H/UC	L
	Neuberger (2007) [RCT][95]	Group exercise vs control<br>SMD 0.22 (-0.12, 0.55)<br>Home exercise vs control<br>SMD -0.14 (-0.46, 0.18)	CES-D, BL / 12 weeks, mean (SD)<br>Group exercise: 14.8 (8.1) / 13.7 (9.5)<br>Home exercise: 10.6 (7.7) / 10.5 (8.2)<br>Control: 12.9 (8.6) / 11.7 (9.0)		L	H/UC	H/UC	L
	Bespoke meta-analysis including[90;95]	Exercise vs control<br>SMD 0.01 (-0.24, 0.26)						
	van der Giesen (2010) [single arm int.][114]		HADS-D, change BL-12 months, mean (SD †)<br>-0.4 (3.7)					
Psychological distress	de Jong (2003) [RCT][101]	Exercise vs control, change BL-24 months<br>SMD -0.32 (-0.55, -0.09)	HADS total, change BL-24 months, mean (SD)<br>Exercise: -1.2 (4.1)<br>Control: 0.1 (4.0)		L	L	H/UC	L
Self-efficacy	Breedland (2011) [RCT][90]	Exercise vs control at 9 weeks<br>Pain + symptoms: SMD -0.10 (-0.78, 0.57)<br>Function: SMD 0.01 (-0.66, 0.69)	ASES, BL / 9 weeks, mean (SD)<br>*Pain + symptoms*<br>Exercise: 3.12 (0.95) / 3.54 (0.88)<br>Control: 3.34 (0.80) / 3.63 (0.85)<br>*Function*<br>Exercise: 4.03 (0.84) / 4.32 (0.74)<br>Control: 4.21 (0.73) / 4.31 (0.87)		L	L	H/UC	L

† SD calculated from 95% CI

AIMS2 = Arthritis Impact Measurement Scales, Alloc. Conc. = allocation concealment, AMSTAR2 = Assessing the Methodological Quality of Systematic Reviews, ASES = Arthritis Self-Efficacy Scale, BL = baseline, Blind. Asses. = Blinded assessors, Blind. Part. = blinded participants, CES-D = Centre for Epidemiologic Studies – Depression Scale, CI = confidence interval, H/UC = high / unclear risk of bias, HADS = Hospital Anxiety and Depression Scale, L = low risk of bias, RA = rheumatoid arthritis, Rand. Seq. = random sequence generation, RCT = randomised controlled trial, SD = standard deviation, SMD = standardised mean difference

Table –Aerobic + muscle strengthening exercise (RA), results and quality assessment

Outcome (outcome measure)	Study (date) [study type]	Standardised result, SMD (95% CI) unless otherwise stated	Natural result	AMSTAR2 quality	Rand. Seq.	Alloc. Conc.	Blind. Part.	Blind. Asses.
CRP	Neuberger (2007) [RCT][95]	Group exercise vs control<br>SMD 0.08 (-0.25, 0.41)<br>Home exercise vs control<br>SMD -0.09 (-0.41, 0.23)	CRP, BL / 12 weeks, mean (SD)<br>Group exercise: 1.3 (2.0) / 1.1 (1.4)<br>Home exercise: 1.3 (1.6) / 0.9 (1.0)<br>Control: 1.3 (1.8) / 1.0 (1.2)		L	H/UC	H/UC	L
	Stavropoulos-Kalinoglou (2013) [NRT][109]	Exercise vs control at 3 months<br>SMD -1.84 (-2.62, -1.05)	CRP, BL / 3 months / 6 months, mean (SD §)<br>Exercise: 5.5 (2.7) / 3.3 (0.3) / 4.8 (1.4)<br>Control: 4.2 (1.3) / 5.8 (1.9) / 8.0 (3.3)					
	Strasser (2011) [single arm int.][113]		CRP, BL / 6 months, mean (SD)<br>2.85 (6.38) / 1.32 (2.05)					
ESR	Neuberger (2007) [RCT][95]	Group exercise vs control<br>SMD 0.22 (-0.11, 0.55)<br>Home exercise vs control<br>SMD -0.22 (-0.54, 0.10)	ESR, BL / 12 weeks, mean (SD)<br>Group exercise: 32.5 (22.7) / 32.0 (24.2)<br>Home exercise: 23.7 (25.6) / 21.9 (21.9)<br>Control: 27.6 (24.1) / 26.8 (23.4)		L	H/UC	H/UC	L
	Hakkinen (2004) [RCT][99]	Exercise vs control at 2 years<br>SMD -0.42 (-0.93, 0.08)	ESR, BL / 2 years / 5 years, mean (SD)<br>Exercise: 24.4 (17.8) / 10.9 (9.8) / 9.9 (12.1)<br>Control: 24.8 (15.7) / 15.4 (11.5) / 13.8 (12.1)		H/UC	H/UC	H/UC	H/UC
	Westby (2000) [RCT][102]	Exercise vs control at 1 year<br>SMD -0.64 (-1.38, 0.10)	ESR, BL / 1 year, mean (SD)<br>Exercise: 15.8 (19.5) / 12.5 (12.2)<br>Control: 19.3 (11.1) / 21.8 (16.3)		H/UC	H/UC	H/UC	L
	Hakkinen (1999) [RCT][103]	Exercise vs control, change BL-12 months<br>SMD -0.35 (-0.84, 0.14)	ESR, change BL – 12 months, mean (SD †)<br>Exercise: -15.5 (15.9)<br>Control: -9 (20.5)		H/UC	H/UC	H/UC	H/UC
	Lyngberg (1994) [RCT][104]	Exercise vs control at 3 months<br>SMD 0.06 (-0.74, 0.86)	ESR, BL / 3 months, mean (SD §)<br>Exercise: 41.3 (29.1) / 26.5 (17.7)<br>Control: 22 (12.8) / 25.5 (13.5)		H/UC	H/UC	H/UC	L
	Nordemar (1981) [RCT][107]	Exercise vs control, change BL-8 years<br>SMD 0.06 (-0.52, 0.64)	ESR, change BL-8 years, mean (SD)<br>Exercise: 43 (21)<br>Control: 42 (10)		H/UC	H/UC	H/UC	H/UC
	Bespoke meta-analysis including[95;102-104;107]	Exercise vs control<br>SMD -0.10 (-0.35, 0.14), I^2 34.1%						

† SD calculated from 95% CI, § mean (SD) estimated from median (range) using a published formula[87]

Alloc. Conc. = allocation concealment, AMSTAR2 = Assessing the Methodological Quality of Systematic Reviews, BL = baseline, Blind. Asses. = Blinded assessors, Blind. Part. = blinded participants, CI = confidence interval, CRP = C-reactive protein, ESR = erythrocyte sedimentation rate, H/UC = high / unclear risk of bias, L = low risk of bias, NRT = non-randomised trial, RA = rheumatoid arthritis, Rand. Seq. = random sequence generation, RCT = randomised controlled trial, SD = standard deviation, SMD = standardised mean difference

Table –Aerobic + muscle strengthening exercise (RA), results and quality assessment

Outcome (outcome measure)	Study (date) [study type]	Standardised result, SMD (95% CI) unless otherwise stated	Natural result	AMSTAR2 quality	Rand. Seq.	Alloc. Conc.	Blind. Part.	Blind. Asses.
Radiological damage	Munneke (2005) [RCT][96]	Larsen score, change BL-2 years<br>SMD 0.06 (-0.18, 0.29)	Larsen score, change BL-2 years, mean (SD‡)<br>Exercise: 1.3 (9.7)<br>Control: 0.8 (8.6)<br>Shoulder damage, incidence (%) (relative risk (95%CI))<br>without BL damage: 1) 5, p) 5 (RR 1.0 (0.4, 2.2)<br>with BL damage: 1) 27; p) 10 (RR 2.7 (1.1, 7.0)<br>Elbow, incidence (%) (relative risk (95%CI))<br>without BL damage: 1) 6, p) 4 (RR 1.6 (0.7, 3.8))<br>with BL damage: 1) 20 p) 16 (RR 1.3 (0.6, 2.5))<br>Hip, incidence (%) (relative risk (95%CI))<br>without BL damage: 1) 2, p) 1 (RR 1.5 (0.3, 6.4))<br>with BL damage: 1) 20; p) 11 (RR 1.8 (0.6, 4.7))<br>Knee, incidence (%) (relative risk (95%CI))<br>without BL damage: 1) 11, p) 10 (RR 1.1 (0.6, 1.9)<br>with BL damage: 1) 24 p) 19 (RR 1.3 (0.7, 2.1))<br>Ankle, incidence (%) (relative risk (95%CI))<br>without BL damage: 1) 4, p) 4 (RR 1.1 (0.5, 2.6)<br>with BL damage: 1) 19; p) 19 (RR 1.0 (0.4, 2.3)<br>Subtaler, incidence (%) (relative risk (95%CI))<br>without BL damage: 1) 2; p) 1 (RR 1.2 (0.3, 4.6)<br>with BL damage:1) 40); p) 4 (RR 10 (1.3, 79 [sic]))		L	L	H/UC	L
	de Jong (2004) [RCT][98]		Larsen score, mean difference between exercise and control's change score from BL-2 years, mean (95% CI)<br>-2.1 (-4.2, 0.2)		L	L	H/UC	L
	Hakkinen (2004) [RCT][100]	Exercise vs control at 2 years<br>SMD -0.81 (-1.33, -0.29)	Larsen score, BL / 2 years / 5 years, mean (SD ‡)<br>Exercise: 0.3 (0.8) / 0.7 (1.6) / 1.0 (2.3)<br>Control: 1.0 (2.3) / 2.3 (2.3) / 2.0 (3.1)					
	de Jong (2003) [RCT][101]	Exercise vs control, change BL-24 months<br>0.00 (-0.23, 0.23)	Larsen score, change BL-24 months, mean (SD)<br>Exercise: 0.0 (1.0)<br>Control: 0.0 (1.0)		L	L	H/UC	L

‡ mean (SD) estimated from median (interquartile range) using a published formula[87]

Alloc. Conc. = allocation concealment, AMSTAR2 = Assessing the Methodological Quality of Systematic Reviews, BL = baseline, Blind. Asses. = Blinded assessors, Blind. Part. = blinded participants, CI = confidence interval, H/UC = high / unclear risk of bias, L = low risk of bias, RA = rheumatoid arthritis, Rand. Seq. = random sequence generation, RCT = randomised controlled trial, SD = standard deviation, SMD = standardised mean difference

Table –Aerobic + muscle strengthening exercise (RA), results and quality assessment

Outcome (outcome measure)	Study (date) [study type]	Standardised result, SMD (95% CI) unless otherwise stated	Natural result	AMSTAR2 quality	Rand. Seq.	Alloc. Conc.	Blind. Part.	Blind. Asses.
Radiological damage	Nordemar (1981) [RCT][107]	Exercise vs control at 8 years<br>SMD -0.52 (-1.11, 0.07)	Larsen score, BL / 8 years, mean (SD)<br>Exercise: 6.2 (5.9) / 10.2 (7.2)<br>Control: 6.7 (5.4) / 13.6 (5.8)		H/UC	H/UC	H/UC	H/UC
	Bespoke meta-analysis including[96;100;101;107]	Exercise vs control<br>SMD -0.23 (-0.57, 0.10), I^2 73.8%						
	de Jong (2009) [single arm int.][115]		Larsen score, BL / 18 months, median (net IQR)<br>Exercise: 3.0 (4.5) / 3.0 (4.0)					
Walk-test	Flint-Wagner (2009) [RCT][93]	Exercise vs control, change BL-16 weeks<br>SMD -1.39 (-2.34, -0.45)	50ft walk test, change BL-16 weeks, means (SD)<br>Exercise: -1.2 (1.6)<br>Control: 0.8 (1.0)		H/UC	H/UC	H/UC	H/UC
	Neuberger (2007) [RCT][95]	Group exercise vs control<br>SMD -0.21 (-0.54, 0.13)<br>Home exercise vs control<br>SMD -0.14 (-0.46, 0.18)	50ft walk test, BL / 12 weeks, mean (SD)<br>Group exercise: 10.0 (3.1) / 9.3 (2.8)<br>Home exercise: 9.6 (5.2) / 9.4 (4.4)<br>Control: 9.4 (2.8) / 10.0 (3.9)		L	H/UC	H/UC	L
	Ekdahl (1990) [RCT][105]		60m walk, change 0-6 weeks, mean<br>Dynamic: -3.7<br>Static: -0.5		H/UC	H/UC	H/UC	L
	Nordemar (1981) [RCT][107]	Exercise vs control at 8 years<br>SMD 0.37 (-0.21, 0.97)	850m walk test (mins), BL / 8 years, mean (SD)<br>Exercise: 8.4 (1.0) / 8.9 (1.8)<br>Control: 8.2 (3.5) / 8.0 (2.9)		H/UC	H/UC	H/UC	H/UC
	Neuberger (1997) [single arm int.][116]		50ft walk test, BL / 12 weeks, mean<br>10.41 / 9.44					

Alloc. Conc. = allocation concealment, AMSTAR2 = Assessing the Methodological Quality of Systematic Reviews, BL = baseline, Blind. Asses. = Blinded assessors, Blind. Part. = blinded participants, CI = confidence interval, H/UC = high / unclear risk of bias, IQR = interquartile range, L = low risk of bias, RA = rheumatoid arthritis, Rand. Seq. = random sequence generation, RCT = randomised controlled trial, SD = standard deviation, SMD = standardised mean difference

Table –Aerobic + muscle strengthening exercise (RA), results and quality assessment

Outcome (outcome measure)	Study (date) [study type]	Standardised result, SMD (95% CI) unless otherwise stated	Natural result	AMSTAR2 quality	Rand. Seq.	Alloc. Conc.	Blind. Part.	Blind. Asses.
Grip strength	Neuberger (2007) [RCT][95]	Group exercise vs control<br>Left: SMD 0.01 (-0.32, 0.34)<br>Right: SMD -0.02 (-0.35, 0.31)<br>Home exercise vs control<br>Left: SMD 0.11 (-0.21, 0.43)<br>Right: SMD -0.08 (-0.40, 0.24)	Grip strength, BL / 12 weeks, mean (SD)<br>*Left*<br>Group exercise: 117.4 (46.8) / 138.8 (54.6)<br>Home exercise: 134.7 (59.4) / 144.7 (63.8)<br>Control: 134.8 (56.6) / 138.1 (59.5)<br>*Right*<br>Group exercise: 121.4 (52.4) / 141.8 (56.6)<br>Home exercise: 130.9 (58.7) / 144.8 (64.9)<br>Control: 133.4 (58.7) / 143.0 (60.3)		L	H/UC	H/UC	L
	Hakkinen (2004) [RCT][100]	Exercise vs control at 2 years<br>SMD 0.55 (0.04, 1.05)	Grip strength, BL / 2 years / 5 years, mean (SD)<br>Exercise: 54.8 (30.5) / 72.3 (24.4) / 73.3 (25.7)<br>Control: 50.2 (22.0) / 59.0 (24.4) / 61.5 (25.4)					
	Lyngberg (1994) [RCT][104]	Exercise vs control at 3 months<br>Left: SMD -0.45 (-1.26, 0.36)<br>Right: SMD -0.43 (-1.24, 0.38)	Grip strength, BL / 3 months, mean (SD §)<br>*Left*<br>Exercise: 100.8 (45.0) / 93.5 (42.8)<br>Control: 129.5 (34.9) / 113.8 (47.4)<br>*Right*<br>Exercise: 93.3 (32.1) / 100.3 (41.9)<br>Control: 114.5 (31.2) / 119.3 (46.8)		H/UC	H/UC	H/UC	L
	Nordgren (2015) [single arm int.][111]		Grip strength, change BL-1 years, mean (SD ‽)<br>13.74 (49.2)					
	Neuberger (1997) [single arm int.][116]		Grip strength, BL / 12 weeks, mean<br>Left: 127 / 150<br>Right: 121 / 139					

‽ SD calculated from standard error, § mean (SD) estimated from median (range) using a published formula[87]

Alloc. Conc. = allocation concealment, AMSTAR2 = Assessing the Methodological Quality of Systematic Reviews, BL = baseline, Blind. Asses. = Blinded assessors, Blind. Part. = blinded participants, CI = confidence interval, H/UC = high / unclear risk of bias, L = low risk of bias, RA = rheumatoid arthritis, Rand. Seq. = random sequence generation, RCT = randomised controlled trial, SD = standard deviation, SMD = standardised mean difference

Table –Aerobic + muscle strengthening exercise (RA), results and quality assessment

Outcome (outcome measure)	Study (date) [study type]	Standardised result, SMD (95% CI) unless otherwise stated	Natural result	AMSTAR2 quality	Rand. Seq.	Alloc. Conc.	Blind. Part.	Blind. Asses.
Bone mineral density	de Jong (2004) [RCT][97]		<u>Hip BMD, change BL – 1 year, median (IQR)</u><br>Exercise: 0% (-2.0, 2.0)<br>Control: 1% (-3.7, 0.5)<br><u>Lumbar spine BMD, change BL – 1 year, median (IQR)</u><br>Exercise: 1.1% (-0.7, 2.3)<br>Control: 0.9% (-1.2, 3.2)		L	L	H/UC	L
	Hakkinen (2004) [RCT][100]	<u>Exercise vs control, change BL-2 years</u><br>Lumbar spine: 0.43 (-0.08, 0.93)<br>Femoral neck: 0.39 (-0.11, 0.90)	<u>Bone mineral density, change BL-2 years, mean (SD †)</u><br>*<u>Lumbar spine</u>*<br>Exercise: 0.01 (0.07)<br>Control: -0.02 (0.07)<br>*<u>Femoral neck</u>*<br>Exercise: 0.01 (0.04)<br>Control: -0.01 (0.06)		H/UC	H/UC	H/UC	H/UC
	Hakkinen (1999) [RCT][103]	<u>Exercise vs control, change BL-12 months</u><br>Lumbar spine: 0.33 (-0.16, 0.82)<br>Femoral neck: 0.31 (-0.18, 0.80)	<u>Bone mineral density, % change BL – 12 months, mean (SD)</u><br>*<u>Lumbar spine</u>*<br>Exercise: 0.19% (3.71)<br>Control: -1.14% (4.36)<br>*<u>Femoral neck</u>*<br>Exercise: 1.10% (3.71)<br>Control: -0.03% (3.58)		H/UC	H/UC	H/UC	H/UC

† SD calculated from 95% CI

Alloc. Conc. = allocation concealment, AMSTAR2 = Assessing the Methodological Quality of Systematic Reviews, BL = baseline, Blind. Asses. = Blinded assessors, Blind. Part. = blinded participants, BMD = bone mineral density, CI = confidence interval, H/UC = high / unclear risk of bias, IQR = interquartile range, L = low risk of bias, RA = rheumatoid arthritis, Rand. Seq. = random sequence generation, RCT = randomised controlled trial, SD = standard deviation, SMD = standardised mean difference

Table – Aerobic + muscle strengthening exercise (RA), SF36 results at final follow-up, mean (SD)

Author (date)	PCS	MCS	GH	PF	RP	RE	SF	BP	V	MH
Hurkmans (2010) [exercise][91]	5.2 (20.8) ‡	4.9 (18.8) ‡								
Hurkmans (2010) [control][91]	1.7 (18.2) ‡	2.7 (19.7) ‡								
Bulthuis (2007) [exercise][94]	28.1 †	50.0 (10.3)								
Bulthuis (2007) [control][94]	25.8 (5.0)	46.2 (13.2)								
van den Berg (2006) [individualised exercise][92]	4.9 (17.6) §	-0.2 (21.0) §								
van den Berg (2006) [exercise guidance][92]	4.0 (18.2) §	0.8 (16.7) §								

† cannot calculate SD as full 95% CI not reported,

§ change from BL-12 months, ‡ change from BL-24 months

BL = baseline, BP = bodily pain, FU = follow-up, GH = general health, IQR = interquartile range, MCS = mental component score, MH = mental health, PCS = physical component score, PF = physical function, RE = role emotional, RP = role physical, SD = standard deviation, SF = social functioning, V = vitality

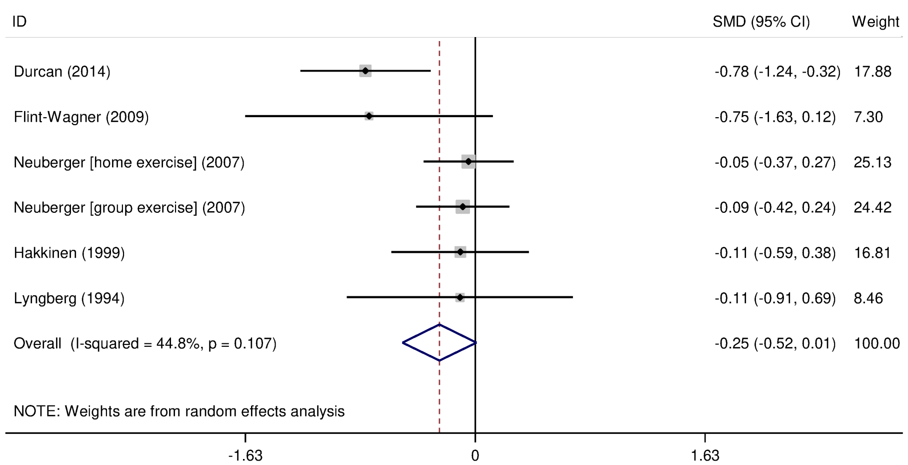

Figure – The effect of aerobic + muscle strengthening exercise on pain (RA)

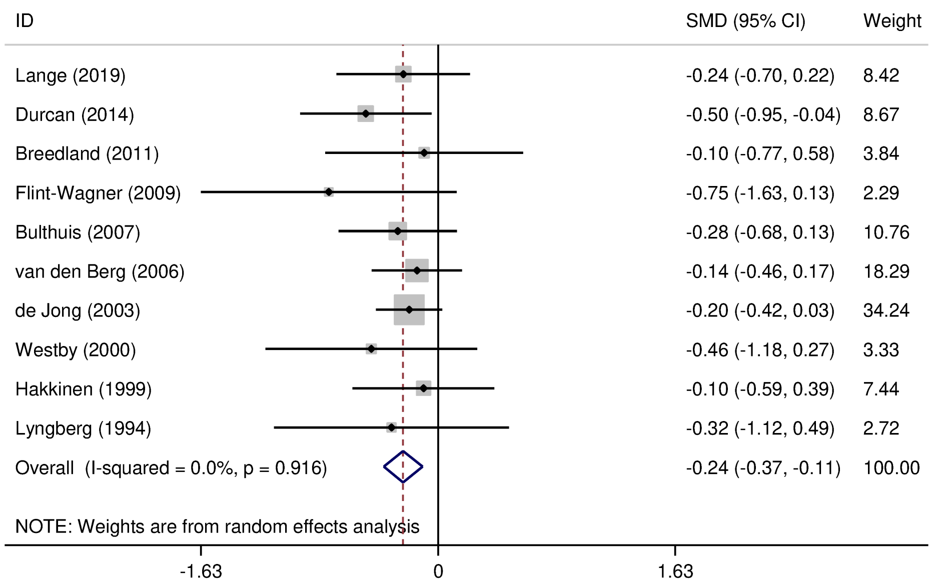

Figure – The effect of aerobic + muscle strengthening exercise on function (RA)

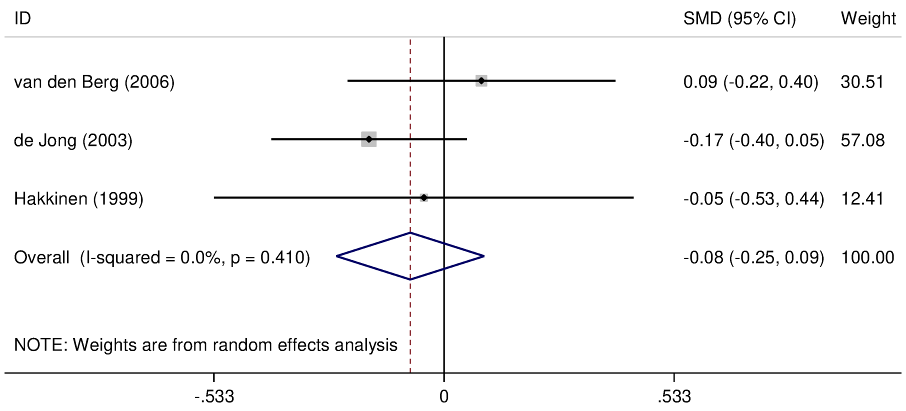

Figure – The effect of aerobic + muscle strengthening exercise on disease

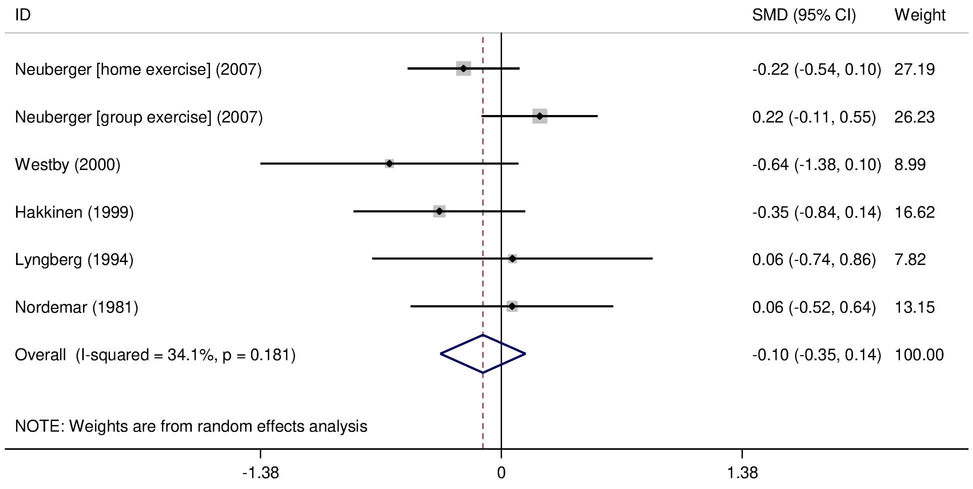

Figure – The effect of aerobic + muscle strengthening exercise on ESR (RA)

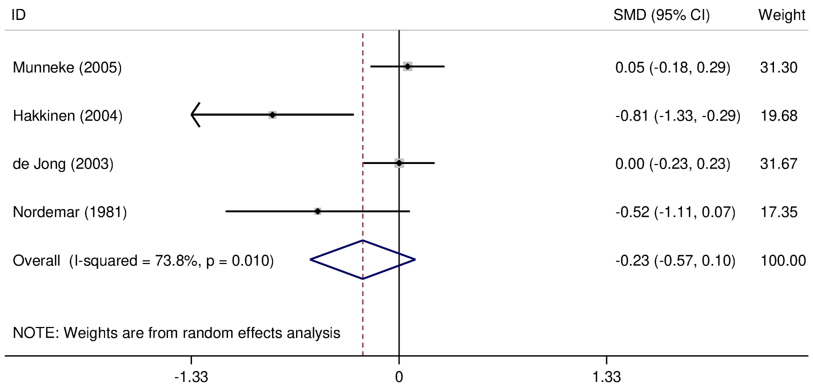

Figure – The effect of aerobic + muscle strengthening exercise on radiological damage (RA)

Supplementary table 34 – Description of reviews of studies of aquatic exercise in RA

Table – Aquatic exercises (RA), description of reviews

Authors (date)	Review type	Study type included	Exposure detail	Number of studies included	Funders
Siegel (2017)[69]	SR	Reviews, RCTs, observational	Aquatic exercises	2	**Not reported**
Al-Qubaeissy (2013)[117]	SR	RCTs	Aquatic exercises and hydrotherapy	6	**Not reported**

RA = rheumatoid arthritis, RCT = randomised controlled trial, SR = systematic review

Supplementary table 35 – Description of original studies of aquatic exercise in RA

Table – Aquatic exercises (RA), description of included studies

Author (date) [country]	Study design	Inclusion criteria	Exposure detail	N	Age years, mean (SD)	N (%) female	Funders
Siqueira (2017) [Brazil][118]	RCT	1987 ACR RA criteria, mild-moderate disease activity, functional class I-II, stable DMARDs for 3 months, capacity to perform exercise<br>Exclusions: circulatory problems, ulcers or skin lesions, regular physical activity or rehabilitation in past 3 months, use of orthoses / ambulatory device, prosthetic hip or keep, regular use of protein supplements or anabolic medication, orthopaedic surgery scheduled in next 6 months, intra-articular injection in past 3 months, cognitive/auditory/visual deficits, water phobia, hypersensitivity to pool cleaning products, active/recurrent infection, epilepsy, urinary or faecal incontinence, anaemia or liver kidney function out of range	Exercise classes divided into warm-up and then specific lower limb exercises – 3x per week<br>1) Aquatic<br>2) Land based<br>p) no physical activity	1) 33<br>2) 33<br>p) 34	1) 55 (6)<br>2) 54 (5.1)<br>p) 53.2 (7)	1) 33 (100)<br>2) 33 (100)<br>p) 34 (100)	**University** (Federal University of Sao Paulo)
Eversden (2007) [UK][119]	RCT	1987 ACR RA criteria, ≥18 years, functional class I-III, communicate in English, stable DMARDs for 6 weeks, stable NSAIDs for 2 weeks<br>Exclusions: surgery in past 3 months or planned, physiotherapy / hydrotherapy in past 3 months, chlorine sensitivity, infected open wound, poorly controlled epilepsy, hypertension, diabetes, incontinence of faeces, fear of water, pregnancy, patients with comorbidities that prevent safe hydrotherapy, carriers of methicillin resistant staphylococcus aureus in the upper respiratory tract; and those who weighed more than 102 kg	1) Exercises in a warm pool – exercises focused on joint mobility, muscle strength and functional activities<br>p) Similar exercises on land	1) 57<br>p) 58	1) 55.2 (13.3)<br>p) 56.1 (11.9)	1) 39 (68.4)<br>p) 42 (72.4)	**Charity** (University Hospital Birmingham NHS Foundation Trust Charities)
Bilberg (2005) [Sweden][120]	RCT	1987 ACR RA criteria, symptom duration 1-5 years, stable medication for 3 months, functional class I-III, aged 20-65 years<br>Exclusions: Other severe diseases or functional limitations that would make pool training impossible	1) 45 min long sessions of moderate intensity aerobic exercise in a warm pool<br>p) Continued daily activities	1) 20<br>p) 23	Median (range)<br>1) 49 (32-62)<br>p) 46 (21-65)	Not reported	**Charity** (Swedish Rheumatism Association, Rune and Ulla Amlov's Foundation)

ACR = American College of Rheumatology, DMARD = disease modifying anti-rheumatic drug, N = number, NHS = National Health Service, NSAID = non-steroidal anti-inflammatory drug, RA = rheumatoid arthritis, RCT = randomised controlled trial, SD = standard deviation, UK = United Kingdom

Table – Aquatic exercises (RA), description of included studies

Author (date) [country]	Study design	Inclusion criteria	Exposure detail	N	Age years, mean (SD)	N (%) female	Funders
Hansen (1993) [Denmark][78]	RCT	Definite or classical RA<br>Exclusions: Functional status III-IV, aged <20 or >60 years, disease other than RA with contraindicate / make physical training impossible, already training ≥3x per week	1) Self-training after instruction<br>2) As 1, plus training with physio once per week<br>3) As 1 plus weekly group training<br>4) As 3 plus training in hot pool<br>p) No training	1) 15<br>2) 15<br>3) 15<br>4) 15<br>p) 15	Median (IQR)<br>1) 55 (44, 58)<br>2) 52 (46, 58)<br>3) 51 (42, 56)<br>4) 54 (44, 56)<br>p) 51 (46, 57)	1) 12 (80.0)<br>2) 7 (46.7)<br>3) 9 (60.0)<br>4) 11 (73.3)<br>p) 10 (66.7)	**Charity** (Danish Arthritis Foundation, Danish Physiotherapists' Research Fund), **Government** (Danish Research Council, Fund for Medical Research in South Jutland)
Minor (1995) [USA][80]	NRT	Aged 21-64 years, women, expressed intent to exercise, no pre-existing medical condition, function class I-II	1) 12 weeks – low impact aerobic dance, walking, aquatics<br>p) Usual care	1) 20<br>p) 22	1) 46.0 (13.1)<br>p) 54.8 (8.4)	1) 20 (100)<br>p) 22 (100)	**Charity** (Arthritis Foundation), **Government (**NIDRR)

N = number, NIDRR = National Institute on Disability and Rehabilitation Research, NRT = non-randomised trial, RA = rheumatoid arthritis, RCT = randomised controlled trial, SD = standard deviation, USA = United States of America

Supplementary table 36 – Results from reviews and interventional studies of aquatic exercise in RA

Table – Aquatic exercises (RA), results and quality assessment

Outcome (outcome measure)	Study (date) [study type]	Standardised result, SMD (95% CI) unless otherwise stated	Natural result	AMSTAR2 quality	Rand. Seq.	Alloc. Conc.	Blind. Part.	Blind. Asses.
Pain	Al-Qubaeissy (2013) [SR][117]		1/3 studies reported improvements in pain	Moderate				
	Eversden (2007) [RCT][119]	Aquatic vs land exercise<br>SMD -0.27 (-0.64, 0.10) [in favour of aquatic exercises]	Pain VAS, BL / 6 weeks, mean (SD †)<br>Aquatic exercise: 28.0 (30.4) / 25.8 (22.8)<br>Land exercises: 30.5 (28.1) / 33.5 (32.7)		L	L	H/UC	L
	Hansen (1993) [RCT][78]	Aquatic exercise vs other exercises / control at 2 years<br>Aquatic vs group: SMD -1.21 (-2.00, -0.43)<br>Aquatic vs physio: SMD -0.73 (-1.47, 0.01)<br>Aquatic vs individual: SMD 0.49 (-0.24, 1.21)<br>Aquatic vs control: SMD -0.49 (-1.21, 0.24)	Pain VAS, BL / 2 years, mean (SD †)<br>Individual exercise: 1.6 (0.3) / 1.4 (0.3)<br>Physio: 1.8 (0.4) / 1.9 (0.3)<br>Group training: 1.9 (0.4) / 2.1 (0.3)<br>Group + pool: 1.9 (0.2) / 1.6 (0.5)<br>Control: 1.9 (0.2) / 1.8 (0.3)		H/UC	H/UC	H/UC	L
	Bespoke meta-analysis including[78;119]	Aquatic exercise vs land exercise<br>SMD -0.68 (-1.59, 0.24), I^2 78%<br>[included the aquatic vs group comparison from Hansen et al]						
Function	Siegel (2017) [SR][69]		1 review[121] reported an improvement in function, but not greater than control	Moderate				
	Al-Qubaeissy (2013) [SR][117]		0/3 studies reported improvements in function	Moderate				
	Siqueira (2017) [RCT][118]	Aquatic vs land at 16 weeks<br>SMD -0.78 (-1.29, -0.28)<br>Aquatic vs control at 16 weeks<br>SMD -0.72 (-1.22, -0.23)	HAQ, BL / 16 weeks, mean (SD)<br>Aquatic exercise: 0.7 (0.5) / 0.4 (0.4)<br>Land exercise: 0.7 (0.5) / 0.8 (0.6)<br>Control: 0.8 (0.5) / 1.3 (1.7)		L	L	H/UC	L
	Eversden (2007) [RCT][119]	Aquatic vs land exercise<br>SMD 0.17 (-0.20, 0.54) [in favour of land exercises]	HAQ, BL / 6 weeks, mean (SD †)<br>Aquatic exercise: 1.36 (1.00) / 1.47 (0.59)<br>Land exercises: 1.46 (0.85) / 1.35 (0.81)		L	L	H/UC	L
	Bilberg (2005) [RCT][120]	Exercise vs control at 3 months<br>SMD -0.18 (-0.78, 0.42)	HAQ, BL / 3 months, mean (SD)<br>Aquatic exercise: 0.9 (0.5) / 0.7 (0.5)<br>Control: 0.7 (0.5) / 0.8 (0.6)		L	H/UC	H/UC	L
	Bespoke meta-analysis including[118;119]	Aquatic exercise vs land exercise<br>SMD -0.29 (-1.23, 0.64), I^2 89%						
Disease activity	Siqueira (2017) [RCT][118]	Aquatic vs land at 16 weeks<br>SMD -0.45 (-0.94, 0.04)<br>Aquatic vs control at 16 weeks<br>SMD -1.16 (-1.68, -0.64)	DAS28, BL / 16 weeks, mean (SD)<br>Aquatic exercise: 3.8 (1.2) / 3.1 (1)<br>Land exercise: 3.6 (1.2) / 3.6 (1.2)<br>Control: 4.3 (0.9) / 4.2 (0.9)		L	L	H/UC	L

† Mean (SD) estimated from median (interquartile range) using published formula[87]

Alloc. Conc. = allocation concealment, AMSTAR2 = Assessing the Methodological Quality of Systematic Reviews, BL = baseline, Blind. Asses. = Blinded assessors, Blind. Part. = blinded participants, CI = confidence interval, DAS28 = Disease Activity Score 28, H/UC = high / unclear risk of bias, HAQ = Health Assessment Questionnaire, L = low risk of bias, RA = rheumatoid arthritis, Rand. Seq. = random sequence generation, RCT = randomised controlled trial, SD = standard deviation, SMD = standardised mean difference, SR = systematic review, VAS = visual analogue scale

Table – Aquatic exercises (RA), results and quality assessment

Outcome (outcome measure)	Study (date) [study type]	Standardised result, SMD (95% CI) unless otherwise stated	Natural result	AMSTAR2 quality	Rand. Seq.	Alloc. Conc.	Blind. Part.	Blind. Asses.
Swollen joints	Hansen (1993) [RCT][78]	Aquatic exercise vs other exercises / control at 2 years<br>Aquatic vs group: SMD -0.92 (-1.68, -0.17)<br>Aquatic vs physio: SMD -0.33 (-1.05, 0.39)<br>Aquatic vs individual: SMD -0.37 (-1.09, 0.35)<br>Aquatic vs control: SMD -1.16 (-1.94, -0.39)	Swollen joint count, BL / 2 years, mean (SD †)<br>Individual exercise: 3.5 (1.3) / 3.4 (1.8)<br>Physio: 3.9 (1.0) / 3.3 (1.6)<br>Group training: 2.8 (1.3) / 4.5 (2.2)<br>Group + pool: 3.3 (0.9) / 2.8 (1.4)<br>Control: 3.7 (1.7) / 5.9 (3.5)		H/UC	H/UC	H/UC	L
Morning stiffness	Hansen (1993) [RCT][78]	Aquatic exercise vs other exercises / control at 2 years<br>Aquatic vs group: SMD 0.87 (0.12, 1.62)<br>Aquatic vs physio: SMD -1.46 (-2.27, -0.65)<br>Aquatic vs individual: SMD 0.05 (-0.67, 0.77)<br>Aquatic vs control: SMD -1.57 (-2.40, -0.75)	Morning stiffness, BL / 2 years, mean (SD †)<br>Individual exercise: 39.3 (14.1) / 24.5 (10.9)<br>Physio: 33.5 (16.1) / 46.0 (18.4)<br>Group training: 32.5 (14.4) / 17.5 (8.6)<br>Group + pool: 58.8 (18.7) / 25.0 (8.6)<br>Control: 53.3 (19.3) / 53.8 (24.4)		H/UC	H/UC	H/UC	L
QoL	Siegel (2017) [SR][69]		2 reviews, one reported short-term benefits, the other reported improvements but not greater than control	Moderate				
	Al-Qubaeissy (2013) [SR][117]		0/3 studies reported improvements in QoL	Moderate				
	Eversden (2007) [RCT][119]	Aquatic vs land exercise<br>SMD 0.00 (-0.37, 0.37)	EQ5D, BL / 6 weeks, mean (SD †)<br>Aquatic exercise: 0.67 (0.21) / 0.69 (0.14)<br>Land exercises: 0.68 (0.13) / 0.69 (0.15)		L	L	H/UC	L
Acute phase-reactants	Al-Qubaeissy (2013) [SR][117]		0/2 studies reported improvements in CRP/ESR	Moderate				
ESR	Hansen (1993) [RCT][78]	Aquatic exercise vs other exercises / control at 2 years<br>Aquatic vs group: SMD -0.66 (-1.39, 0.08)<br>Aquatic vs physio: SMD -0.54 (-1.27, 0.19)<br>Aquatic vs individual: SMD -0.91 (-1.67, -0.16)<br>Aquatic vs control: SMD -1.40 (-2.21, -0.60)	ESR, BL / 2 years, mean (SD †)<br>Individual exercise: 32.3 (9.5) / 25.8 (11.2)<br>Physio: 25.5 (5.2) / 20.8 (6.0)<br>Group training: 22.0 (10.3) / 25.0 (14.9)<br>Group + pool: 21.3 (3.7) / 17.5 (6.3)<br>Control: 22.5 (5.7) / 30.5 (11.5)		H/UC	H/UC	H/UC	L

† Mean (SD) estimated from median (interquartile range) using published formula[87]

Alloc. Conc. = allocation concealment, AMSTAR2 = Assessing the Methodological Quality of Systematic Reviews, BL = baseline, Blind. Asses. = Blinded assessors, Blind. Part. = blinded participants, CI = confidence interval, ESR =erythrocyte sedimentation rate, H/UC = high / unclear risk of bias, L = low risk of bias, RA = rheumatoid arthritis, Rand. Seq. = random sequence generation, RCT = randomised controlled trial, SD = standard deviation, SMD = standardised mean difference, SR = systematic review

Table – Aquatic exercises (RA), results and quality assessment

Outcome (outcome measure)	Study (date) [study type]	Standardised result, SMD (95% CI) unless otherwise stated	Natural result	AMSTAR2 quality	Rand. Seq.	Alloc. Conc.	Blind. Part.	Blind. Asses.
Radiological damage	Hansen (1993) [RCT][78]	Aquatic exercise vs other exercises / control at 2 years<br>Aquatic vs group: SMD -0.10 (-0.82, 0.62)<br>Aquatic vs physio: SMD -0.38 (-1.10, 0.35)<br>Aquatic vs individual: SMD 0.08 (-0.64, 0.79)<br>Aquatic vs control: SMD -1.74 (-2.59, -0.89)	Larsen score, BL / 2 years, mean (SD †)<br>Individual exercise: 43.0 (19.0) / 50.0 (20.1)<br>Physio: 47.5 (9.2) / 57.8 (14.1)<br>Group training: 41.8 (17.0) / 53.3 (16.4)<br>Group + pool: 42.0 (19.5) / 51.5 (19.0)<br>Control: 70.8 (8.9) / 77.5 (9.2)		H/UC	H/UC	H/UC	L
Grip strength	Minor (1995) [NRT][80]	Exercise vs control at 12 months<br>0.28 (-0.33, 0.88)	Grip strength at 12 months, mean (SD)<br>Exercise: 110 (60)<br>Control: 95 (49)					
Walk-test	Eversden (2007) [RCT][119]	Aquatic vs land exercise<br>SMD 0.00 (-0.37, 0.37)	10m walk-time (s), BL / 6 weeks, mean (SD †)<br>Aquatic exercise: 10.5 (3.7) / 9.0 (2.7)<br>Land exercises: 10.7 (3.3) / 9.0 (3.3)		L	L	H/UC	L
Work	Minor (1995) [NRT][80]	Exercise vs control at 12 months<br>Hands: SMD 0.08 (-0.52, 0.69)<br>Lift: SMD 0.60 (-0.02, 1.22)<br>Legs: SMD 0.67 (0.05, 1.30)	Work Capacity Evaluation at 12 months, mean (SD)<br>*Hands*<br>Exercise: 1.8 (1.1)<br>Control: 1.7 (1.3)<br>*Lift*<br>Exercise: 2.6 (0.5)<br>Control: 2.3 (0.5)<br>*Legs*<br>Exercise: 2.9 (0.2)<br>Control: 2.5 (0.8)					

† Mean (SD) estimated from median (interquartile range) using published formula[87]

Alloc. Conc. = allocation concealment, AMSTAR2 = Assessing the Methodological Quality of Systematic Reviews, BL = baseline, Blind. Asses. = Blinded assessors, Blind. Part. = blinded participants, CI = confidence interval, H/UC = high / unclear risk of bias, L = low risk of bias, NRT = non-randomised trial, RA = rheumatoid arthritis, Rand. Seq. = random sequence generation, RCT = randomised controlled trial, SD = standard deviation, SMD = standardised mean difference, SR = systematic review

Table – Aquatic exercise (RA), SF36 results at final follow-up, mean (SD)

Author (date)	PCS	MCS	GH	PF	RP	RE	SF	BP	V	MH
Bilberg (2005) [exercise][120]	37.1 (10.5)	45.1 (11.5)	49.8 (19.3)	64.7 (20.0)	39.5 (37.6)	69.9 (36.1)	73.7 (22.4)	50.8 (23.4)	51.8 (22.6)	72.4 (15.9)
Bilberg (2005) [control][120]	38.3 (9.6)	46.2 (10.8)	59.3 (16.1)	64.9 (21.4)	48.9 (38.0)	69.6 (36.1)	71.2 (21.1)	50.9 (21.0)	49.1 (17.6)	72.7 (16.9)

BP = bodily pain, FU = follow-up, GH = general health, MCS = mental component score, MH = mental health, PCS = physical component score, PF = physical function, RE = role emotional, RP = role physical, SD = standard deviation, SF = social functioning, V = vitality

Supplementary table 37 – Description of original studies comparing high vs low intensity exercise in RA

Table – High vs low intensity exercises (RA), description of included studies

Author (date) [country]	Study design	Inclusion criteria	Exposure detail	N	Age years, mean (SD)	N (%) female	Funders
Lemmey (2012) [UK][122]	RCT	1987 ACR RA criteria, aged ≥18 years, functional class I-II, not cognitively impaired, stable drug therapy, steroid dose <10mg/day, Free of other cachectic diseases (e.g. cancer, HIV, infection), free of medical conditions contraindicating regular high intensity exercise, not taking drugs or nutritional supplements known to be anabolic, not undertaking regular, intense physical training, not pregnant	1) High intensity progressive muscle strengthening training<br>2) Home-based, low-intensity range of motion exercises	1) 9<br>2) 9	1) 55.7 (8.6)<br>2) 59.4 (10.8)	1) 8 (88.9)<br>p) 6 (66.7)	**Charity** (Arthritis Research Campaign)
Lemmey (2009) [UK][123]	RCT	See Lemmey 2012	1) High intensity progressive muscle strengthening training<br>2) Home-based, low-intensity range of motion exercises	1) 13<br>2) 15	1) 55.6 (8.3)<br>2) 60.6 (11.2)	1) 11 (84.6)<br>p) 12 (80.0)	**Charity** (Arthritis Research Campaign)
van den Ende (2000) [The Netherlands][124]	RCT	1987 ACR RA criteria, aged 20-80 years, ability to walk 50ft in doors, ≥6 swollen joints and at least two of the following: morning stiffness >45 mins, tender joint count >9, ESR >28mm/hr<br>Exclusions: presence of arthroplasty in the knee, inability to tolerate training due to serious cardiac or lung disease	1) Range of motion + isometric exercises + individually tailored regime consisting of strengthening exercises and cycling<br>2) Range of motion + isometric exercise only	1) 34<br>2) 30	1) 62 (13)<br>p) 58 (14)	1) 20 (58.8)<br>2) 20 (66.7)	**Charity (**ZONMw) [Netherlands Organization for Health Research and Development]
van den Ende (1996) [The Netherlands][125]	RCT	1987 ACR RA criteria, aged 20-70 years, stable medication for past 3 months, able to cycle on home trainer<br>Exclusions: High disease activity, inability to tolerate physical fitness training due to serious cardiac or lung disease, arthroplasty of weight bearing joint	1) High intensity – high paced exercise including cycling and strengthening exercises<br>2) Low intensity – range of motion and non-weight bearing isometric, muscle strengthening – performed seated, prone, standing<br>3) low intensity individual exercises (same as above)<br>4) written instructions to perform home exercises	1) 25<br>2) 25<br>3) 25<br>4) 25	1) 51.1 (9.5)<br>2) 47.7 (13.6)<br>3) 53.1 (12.1)<br>4) 56.1 (12.1)	1) 13 (52.0)<br>2) 16 (64.0)<br>3) 16 (64.0)<br>4) 18 (72.0)	**Charity** (Nationale Commissie Chronisch Zieken foundation), **Industry** (Zorg en Zekerheid)

ACR = American College of Rheumatology, DMARD = disease modifying anti-rheumatic drug, N = number, NSAID = non-steroidal anti-inflammatory drug, RA = rheumatoid arthritis, RCT = randomised controlled trial, SD = standard deviation, UK = United Kingdom

Supplementary table 38 – Results from reviews and interventional studies comparing high vs low intensity exercise in RA

Table – High vs low intensity, results and quality assessment

Outcome (outcome measure)	Study (date) [study type]	Standardised result, SMD (95% CI) unless otherwise stated	Natural result	AMSTAR2 quality	Rand. Seq.	Alloc. Conc.	Blind. Part.	Blind. Asses.
Pain	van den Ende (2000) [RCT][124]	High intensity vs low intensity, change Bl-24 weeks<br>SMD 0.03 (-0.46, 0.52)	Pain VAS, change BL-24 weeks, mean (SD †)<br>High intensity: -1.7 (2.7)<br>Low intensity: -1.8 (3.4)		H/UC	L	H/UC	L
	van den Ende (1996) [RCT][125]	High intensity vs low intensity, change Bl-12 weeks<br>SMD 0.00 (-0.55, 0.55)	Pain VAS, change BL-12 weeks, mean (95% CI)<br>High intensity: 0.2 (1.8)<br>Low intensity: 0.2 (1.4)<br>Low intensity – individual: 0 (1.7)<br>Low intensity – home: 0.9 (1.7)		H/UC	H/UC	H/UC	H/UC
	Bespoke meta-analysis including[124;125]	High vs low intensity exercise<br>SMD 0.02 (-0.35, 0.39), I^2 0%						
Function	Lemmey (2009) [RCT][123]	High intensity vs low intensity at 24 weeks<br>SMD 0.38 (-0.37, 1.13)	HAQ. BL / 24 weeks, mean (SD)<br>High intensity: 0.91 (0.68) / 0.82 (0.69)<br>Low intensity: 0.58 (0.62) / 0.58 (0.59)		L	H/UC	H/UC	H/UC
	van den Ende (1996) [RCT][125]	High intensity vs low intensity, change Bl-12 weeks<br>SMD 0.00 (-0.55, 0.55)	HAQ, change BL-12 weeks, mean (SD †)<br>High intensity: -0.05 (0.36)<br>Low intensity: -0.05 (0.40)<br>Low intensity – individual: -0.03 (0.26)<br>Low intensity – home: 0.16 (0.34)		H/UC	H/UC	H/UC	H/UC
	Bespoke meta-analysis including[123;125]	High vs low intensity exercise<br>SMD 0.13 (-0.31, 0.58), I^2 0%						
Disease activity	Lemmey (2009) [RCT][123]	High intensity vs low intensity at 24 weeks<br>SMD -0.42 (-1.17, 0.33)	DAS28. BL / 24 weeks, mean (SD)<br>High intensity: 3.29 (1.27) / 3.12 (1.34)<br>Low intensity: 3.28 (1.27) / 3.56 (0.71)		L	H/UC	H/UC	H/UC
	van den Ende (2000) [RCT][124]	High intensity vs low intensity, change Bl-24 weeks<br>SMD -0.36 (-0.85, 0.14)	DAS28, change BL-24 weeks, mean (SD †)<br>High intensity: -1.4 (1.9)<br>Low intensity: -0.7 (2.0)		H/UC	L	H/UC	L
	Bespoke meta-analysis including[123;124]	High vs low intensity exercise<br>SMD -0.38 (-0.79, 0.04), I^2 0%						
Tender joints	van den Ende (1996) [RCT][125]	High intensity vs low intensity, change Bl-12 weeks<br>SMD 0.00 (-0.55, 0.55)	Ritchie Index, change BL-12 weeks, mean (95% CI)<br>High intensity: -0.5 (4.3)<br>Low intensity: -0.5 (5.4)<br>Low intensity – individual: 0 (4.6)<br>Low intensity – home: 0.2 (3.8)		H/UC	H/UC	H/UC	H/UC

† SD calculated from 95% CI

Alloc. Conc. = allocation concealment, AMSTAR2 = Assessing the Methodological Quality of Systematic Reviews, BL = baseline, Blind. Asses. = Blinded assessors, Blind. Part. = blinded participants, CI = confidence interval, DAS28 = Disease Activity Score 28, HAQ = Health Assessment Questionnaire, H/UC = high / unclear risk of bias, L = low risk of bias, RA = rheumatoid arthritis, Rand. Seq. = random sequence generation, RCT = randomised controlled trial, SD = standard deviation, SMD = standardised mean difference, VAS = visual analogue scale

Table – High vs low intensity, results and quality assessment

Outcome (outcome measure)	Study (date) [study type]	Standardised result, SMD (95% CI) unless otherwise stated	Natural result	AMSTAR2 quality	Rand. Seq.	Alloc. Conc.	Blind. Part.	Blind. Asses.
Swollen joints	van den Ende (2000) [RCT][124]	High intensity vs low intensity, change Bl-24 weeks<br>SMD -0.35 (-0.84, 0.15)	Swollen joint count, change BL-24 weeks, mean (SD †)<br>High intensity: -6 (8.9)<br>Low intensity: -3 (8.4)		H/UC	L	H/UC	L
	van den Ende (1996) [RCT][125]		Swollen joint count, change BL-12 weeks, mean (95% CI)<br>High intensity: -1.7 (-2.8, -7.3 [sic])<br>Low intensity: 0.8 (0.0, 1.6)<br>Low intensity – individual: 0 (-1.1, 1.2)<br>Low intensity – home: 0.2 (-0.7, 1.2)		H/UC	H/UC	H/UC	H/UC
Patient global	van den Ende (1996) [RCT][125]	High intensity vs low intensity, change Bl-12 weeks<br>SMD 0.30 (-0.26, 0.86)	Patient global VAS, change BL-12 weeks, mean (95% CI)<br>High intensity: 0.1 (2.3)<br>Low intensity: -0.6 (2.4)<br>Low intensity – individual: -0.1 (1.9)<br>Low intensity – home: 0.3 (2.7)		H/UC	H/UC	H/UC	H/UC
ESR	van den Ende (2000) [RCT][124]	High intensity vs low intensity, change Bl-24 weeks<br>SMD -0.50 (-1.00, -0.001)	ESR, change BL-24 weeks, mean (SD †)<br>High intensity: -22 (35.7)<br>Low intensity: -4 (36.3)		H/UC	L	H/UC	L
	van den Ende (1996) [RCT][125]	High intensity vs low intensity, change Bl-12 weeks<br>SMD 0.22 (-0.33, 0.78)	ESR, change BL-12 weeks, mean (95% CI)<br>High intensity: 0 (15.3)<br>Low intensity: -3 (11.5)<br>Low intensity – individual: 3 (14.0)<br>Low intensity – home: -1 (15.3)		H/UC	H/UC	H/UC	H/UC
	Bespoke meta-analysis including[124;125]	High vs low intensity exercise<br>SMD -0.15 (-0.86, 0.56), I^2 72.1%						

† SD calculated from 95% CI

Alloc. Conc. = allocation concealment, AMSTAR2 = Assessing the Methodological Quality of Systematic Reviews, BL = baseline, Blind. Asses. = Blinded assessors, Blind. Part. = blinded participants, CI = confidence interval, ESR = erythrocyte sedimentation rate, H/UC = high / unclear risk of bias, L = low risk of bias, RA = rheumatoid arthritis, Rand. Seq. = random sequence generation, RCT = randomised controlled trial, SD = standard deviation, SMD = standardised mean difference

Table – High vs low intensity, results and quality assessment

Outcome (outcome measure)	Study (date) [study type]	Standardised result, SMD (95% CI) unless otherwise stated	Natural result	AMSTAR2 quality	Rand. Seq.	Alloc. Conc.	Blind. Part.	Blind. Asses.
Walk-test	Lemmey (2012) [RCT][122]	High intensity vs low intensity at 3 years<br>SMD -0.20 (-1.13, 0.73)	50ft walk, BL / 3 years, mean (SD)<br>High intensity: 9.68 (2.77) / 8.50 (1.77)<br>Low intensity: 8.80 (2.96) / 9.06 (3.51)		L	H/UC	H/UC	H/UC
	Lemmey (2009) [RCT][123]	High intensity vs low intensity at 24 weeks<br>SMD -0.65 (-1.41, 0.12)	50ft walk, BL / 24 weeks, mean (SD)<br>High intensity: 9.33 (2.40) / 7.77 (1.40)<br>Low intensity: 10.03 (3.78) / 9.89 (4.28)		L	H/UC	H/UC	H/UC
	van den Ende (1996) [RCT][125]	High intensity vs low intensity, change Bl-12 weeks<br>SMD -0.18 (-0.73, 0.38)	Walk-test, change BL-12 weeks, mean (SD †)<br>High intensity: -0.7 (1.5)<br>Low intensity: -0.4 (1.9)<br>Low intensity – individual: 0.0 (1.3)<br>Low intensity – home: 0.1 (1.5)		H/UC	H/UC	H/UC	H/UC
	Bespoke meta-analysis including[124;125]	High vs low intensity exercise<br>SMD -0.34 (-0.79, 0.11), I^2 0%						

† SD calculated from 95% CI

Alloc. Conc. = allocation concealment, AMSTAR2 = Assessing the Methodological Quality of Systematic Reviews, BL = baseline, Blind. Asses. = Blinded assessors, Blind. Part. = blinded participants, CI = confidence interval, H/UC = high / unclear risk of bias, L = low risk of bias, RA = rheumatoid arthritis, Rand. Seq. = random sequence generation, RCT = randomised controlled trial, SD = standard deviation, SMD = standardised mean difference

Supplementary table 39 – Description of reviews of studies comparing home vs supervised exercise in RA

Table –Home exercise (RA), description of reviews

Authors (date)	Review type	Study type included	Exposure detail	Number of studies included	Funders
Siegel (2017)[69]	SR	Reviews, RCTs, observational	Home exercise	2	**Not reported**
Hammond (2016)[126]	SR	RCTs	Home hand exercises	3	**Not reported**

RA = rheumatoid arthritis, RCT = randomised controlled trial, SR = systematic review

Supplementary table 40 – Description of original studies comparing home vs supervised exercise in RA

Table – Home vs supervised exercise, description of included studies

Author (date) [country]	Study design	Inclusion criteria	Exposure detail	N	Age years, mean (SD)	N (%) female	Funders
Zernicke (2016) [Germany][127]	RCT §	Met 1987 and 2010 criteria, patient global assessment <30, bDMARD treatment<br>Exclusions: epilepsy, flare of RA, previous use of Wii console.	1) Conventional home-based exercise group – strength, balance, joint mobility, relaxation<br>2) Home exercise using Nintendo Wii console – yoga, muscle strengthening, balance, aerobic	1) 15<br>2) 15	1) 59 (9)<br>2) 52 (8)	1) 10 (67)<br>2) 15 (100)	**None**
Seneca (2015) [Denmark][128]	RCT	2010 ACR/EULAR RA criteria, Early RA, aged >18 years, able to participate in intervention, live <20km from hospital, read Danish<br>Exclusions: DAS28-CRP >5.1, myocardial infarction within 6 months, angina pectoris, hypertension at ≥ 180/≥ 110 mmHg or treated hypertension at 160-179/100-109 mmHg, treatment with beta-blockers or presence of symptoms of severe or very severe chronic obstructive pulmonary disease.	1) Supervised exercise on bike as well as strength training<br>2) Self-administered exercises	1) 25<br>2) 26	Median (range)<br>1) 61 (27-79)<br>2) 54 (23-71)	1) 68%<br>2) 69%	**None**
Hsieh (2009) [Taiwan][129]	RCT	1987 ACR RA criteria, aged 20-65 years, symptom duration <6 months, well controlled condition<br>Exclusions: arthroplasty or major operation in knee or hip joint, presence of serious cardiac or pulmonary disease or any severe medical condition, severe arthritis or contracture of knee joint preventing bicycle exercise	1) 8 weeks, 3x per week – 10 mins stretching, 10 mins cycle/treadmill/other exercise machine, 30 min pool exercise, 10 min cool down<br>2) Home exercise programme similar to above	1) 15<br>2) 15	1) 54.1 (8.3)<br>2) 51.2 (12.0)	1) 15 (100)<br>2 15 (100)	**Government** (Taiwan National Science Council), **Hospital** (Shin Kong Wu Ho-Su Memorial Hospital), **University** (Taipei Medical University)
Neuberger (2007) [USA][95]	RCT	1987 ACR RA criteria, aged 40-70 years, communicate in English, ambulatory, no fibromyalgia or COPD, no beta-blockers or digitalis, not performing aerobic exercise 3x per week, having rheumatologist/physician approval to participate, meet criteria for aerobic fitness testing (no arrhythmias, recent myocardial infarction, acute infection, uncontrolled metabolic disease, known electrolyte abnormalities, or systolic BP 200 mm Hg or diastolic BP 115 mm Hg)	1) 12 weeks – low impact aerobic/ muscle strengthening exercise for 1hr 3x per week in a supervised group setting<br>2) Same as above but at home via videotape<br>p) No exercise control	1) 68<br>2) 79<br>p) 73	Median (range)<br>55.5 (40-70)	82.7% female	**Government** (National Institute of Nursing Research of the NIH)
Stenstrom (1994) [Sweden][130]	RCT	1987 ACR RA criteria, aged <70 years, functional class II<br>Exlcusions: 500m walk time >10 mins	1) Goal setting group<br>2) Pain attention group – avoiding pain by not increasing weight too much	1) 22<br>2) 20	median (range)<br>1) 53.5 (26-68)<br>p) 58 (43-69)	1) 68%<br>2) 70%	**Government** (Sormland County Council, Swedish Medical Reserarhc Council), **Professional body** (Swedish Association Against Rheumatism), **University** (Karolinska Intitute)

§ cross-over trial; ACR = American College of Rheumatology, bDMARD = biologic disease modifying anti-rheumatic drug, DAS28-CRP = Disease Activity Sore (28 – C-reactive protein), EULAR = European League Against Rheumatism, N = number, NIH = National Instiute of Health, RA = rheumatoid arthritis, RCT = randomised controlled trial, SD = standard deviation,

Supplementary table 41 – Results from reviews and interventional studies comparing home vs supervised exercise in RA

Table –Home exercise (RA), results and quality assessment

Outcome (outcome measure)	Study (date) [study type]	Standardised result, SMD (95% CI) unless otherwise stated	Natural result	AMSTAR2 quality	Rand. Seq.	Alloc. Conc.	Blind. Part.	Blind. Asses.
Pain	Siegel (2017) [SR][69]		Identified one previous systematic review[131] that reported home exercise was effective	Moderate				
	Hammond (2016) [SR][126]		Home exercises improved self-reported pain in the short-term	Moderate				
	Seneca (2015) [RCT][128]	Home vs supervised, change BL-12 weeks<br>SMD 0.81 (0.24, 1.38)	Pain VAS, change BL-12 weeks, mean (SD †)<br>Supervised exercise: -1.75 (2.29)<br>Home exercise: 0 (2.02)		H/UC	H/UC	H/UC	L
	Hsieh (2009) [RCT][129]	Home vs supervised exercise at 8 weeks<br>SMD -0.40 (-1.12, 0.33)	Pain (0-10), BL / 8 weeks, mean (SD)<br>Supervised exercise: 3.60 (1.88) / 2.70 (2.14)<br>Home exercise: 2.70 (2.14) / 1.79 (2.42)		L	L	H/UC	L
	Neuberger (2007) [RCT][95]	Home exercise vs group exercise at 12 weeks<br>SMD 0.05 (-0.28, 0.37)	McGill Pain, BL / 12 weeks, mean (SD)<br>Group exercise: 4.7 (2.1) / 4.1 (2.2)<br>Home exercise: 3.9 (1.9) / 4.2 (1.9)<br>Control: 4.1 (2.3) / 4.3 (2.3)		L	H/UC	H/UC	L
	Bespoke meta-analysis including[95;128;129]	Home exercise vs supervised exercise<br>SMD 0.17 (-0.43, 0.77), I^2 74.1% [in favour of supervised exercise]						
Function	Hammond (2016) [SR][126]		Home exercises improved hand function in the short-term	Moderate				
	Zernicke (2016) [RCT][127]		HAQ, BL / 12 weeks, mean<br>Home conventional exercise: 1.0 / 0.9<br>Wii console exercise: 0.7 / 0.7		H/UC	H/UC	H/UC	H/UC
	Seneca (2015) [RCT][128]	Home vs supervised, change BL-12 weeks<br>SMD -0.19 (-0.74, 0.36)	HAQ, change BL-12 weeks, mean (SD †)<br>Supervised exercise: -0.03 (0.29)<br>Home exercise: -0.08 (0.23)		H/UC	H/UC	H/UC	L
	Hsieh (2009) [RCT][129]	Home vs supervised exercise at 8 weeks<br>SMD -0.14 (-0.85, 0.58)	HAQ, BL / 8 weeks, mean (SD)<br>Supervised exercise: 0.44 (0.42) / 0.36 (0.31)<br>Home exercise: 0.41 (0.37) / 0.32 (0.27)		L	L	H/UC	L
	Stenstrom (1994) [RCT][130]	Goal setting vs pain attention<br>SMD -0.61 (-1.23, 0.01)	HAQ, change BL-24 weeks, mean (SD †)<br>Goal setting: -0.06 (0.32)<br>Pain attention: 0.22 (0.57)		H/UC	H/UC	H/UC	H/UC
	Bespoke meta-analysis including[128;129]	Home exercise vs supervised exercise<br>SMD -0.17 (-0.61, 0.26), I^2 0% [in favour of home exercise]						

† mean (SD) estimated from median (range) using published formula[87]

Alloc. Conc. = allocation concealment, AMSTAR2 = Assessing the Methodological Quality of Systematic Reviews, BL = baseline, Blind. Asses. = Blinded assessors, Blind. Part. = blinded participants, CI = confidence interval, H/UC = high / unclear risk of bias, HAQ = Health Assessment Questionnaire, L = low risk of bias, RA = rheumatoid arthritis, Rand. Seq. = random sequence generation, RCT = randomised controlled trial, SD = standard deviation, SMD = standardised mean difference, SR = systematic review, VAS = visual analogue scale

Table –Home exercise (RA), results and quality assessment

Outcome (outcome measure)	Study (date) [study type]	Standardised result, SMD (95% CI) unless otherwise stated	Natural result	AMSTAR2 quality	Rand. Seq.	Alloc. Conc.	Blind. Part.	Blind. Asses.
Disease activity	Zernicke (2016) [RCT][127]		Disease activity VAS, BL / 12 weeks, mean<br>Home conventional exercise: 16 / 20<br>Wii console exercise: 18 / 17		H/UC	H/UC	H/UC	H/UC
	Seneca (2015) [RCT][128]	Home vs supervised, change BL-12 weeks<br>SMD 0.89 (0.31, 1.47)	DAS28, change BL-12 weeks, mean (SD †)<br>Supervised exercise: -0.69 (0.85)<br>Home exercise: 0.07 (0.86)		H/UC	H/UC	H/UC	L
Tender joints	Hsieh (2009) [RCT][129]	Home vs supervised exercise at 8 weeks<br>SMD -0.24 (-0.96, 0.47)	Tender joint count, BL / 8 weeks, mean (SD)<br>Supervised exercise: 16.80 (15.77) / 16.73 (15.91)<br>Home exercise: 14.87 (9.75) / 13.53 (9.46)		L	L	H/UC	L
	Neuberger (2007) [RCT][95]	Home exercise vs group exercise at 12 weeks<br>SMD -0.28 (-0.61, 0.05)	Tender joint count (164), BL / 12 weeks, mean (SD)<br>Group exercise: 32.2 (29.1) / 31.0 (29.2)<br>Home exercise: 29.0 (23.1) / 23.7 (23.1)<br>Control: 37.1 (25.1) / 35.1 (26.7)		L	H/UC	H/UC	L
	Stenstrom (1994) [RCT][130]	Goal setting vs pain attention<br>SMD -1.06 (-1.71, -0.41)	Ritchie index, change BL-24 weeks, mean (SD †)<br>Goal setting: -12.5 (9.9)<br>Pain attention: -2.25 (9.4)		H/UC	H/UC	H/UC	H/UC
	Bespoke meta-analysis including[95;129]	Home exercise vs supervised exercise<br>SMD -0.27 (-0.57, 0.02), I^2 0% [in favour of home exercise]						
Swollen joints	Hsieh (2009) [RCT][129]	Home vs supervised exercise at 8 weeks<br>SMD -0.29 (-1.01, 0.43)	Swollen joint count, BL / 8 weeks, mean (SD)<br>Supervised exercise: 9.07 (10.40) / 8.40 (9.93)<br>Home exercise: 7.00 (4.42) / 6.13 (4.52)		L	L	H/UC	L
Stiffness	Siegel (2017) [SR][69]		Identified one previous systematic review[131] that reported home exercise was effective	Moderate				
Fatigue	Neuberger (2007) [RCT][95]	Home exercise vs group exercise at 12 weeks<br>SMD -0.14 (-0.46, 0.19)	Global fatigue, BL / 12 weeks, mean (SD)<br>Group exercise: 24.9 (10.3) / 20.7 (11.6)<br>Home exercise: 20.1 (10.2) / 19.2 (10.6)<br>Control: 21.9 (9.8) / 20.9 (11.2)		L	H/UC	H/UC	L
Patient global	Hsieh (2009) [RCT][129]	Home vs supervised exercise at 8 weeks<br>SMD -0.62 (-1.35, 0.12)	Patient global, BL / 8 weeks, mean (SD)<br>Supervised exercise: 4.01 (1.90) / 2.67 (2.06)<br>Home exercise: 2.44 (2.28) / 1.47 (1.82)		L	L	H/UC	L

† mean (SD) estimated from median (range) using published formula[87]

Alloc. Conc. = allocation concealment, AMSTAR2 = Assessing the Methodological Quality of Systematic Reviews, BL = baseline, Blind. Asses. = Blinded assessors, Blind. Part. = blinded participants, CI = confidence interval, DAS28 = disease activity score (28), H/UC = high / unclear risk of bias, L = low risk of bias, RA = rheumatoid arthritis, Rand. Seq. = random sequence generation, RCT = randomised controlled trial, SD = standard deviation, SMD = standardised mean differenceVAS = visual analogue scale

Table –Home exercise (RA), results and quality assessment

Outcome (outcome measure)	Study (date) [study type]	Standardised result, SMD (95% CI) unless otherwise stated	Natural result	AMSTAR2 quality	Rand. Seq.	Alloc. Conc.	Blind. Part.	Blind. Asses.
Anxiety	Hsieh (2009) [RCT][129]	Home vs supervised exercise at 8 weeks<br>SMD -0.13 (-0.85, 0.59)	AIMS anxiety, BL / 8 weeks, mean (SD)<br>Supervised exercise: 2.40 (1.72) / 2.07 (1.03)<br>Home exercise: 2.13 (1.30) / 1.93 (1.10)		L	L	H/UC	L
Depression	Hsieh (2009) [RCT][129]	Home vs supervised exercise at 8 weeks<br>SMD -0.12 (-0.83, 0.60)	AIMS depression, BL / 8 weeks, mean (SD)<br>Supervised exercise: 1.73 (1.03) / 1.73 (1.10)<br>Home exercise: 1.67 (1.05) / 1.60 (1.12)		L	L	H/UC	L
	Neuberger (2007) [RCT][95]	Home exercise vs group exercise at 12 weeks<br>SMD -0.36 (-0.69, -0.04)	CES-D, BL / 12 weeks, mean (SD)<br>Group exercise: 14.8 (8.1) / 13.7 (9.5)<br>Home exercise: 10.6 (7.7) / 10.5 (8.2)<br>Control: 12.9 (8.6) / 11.7 (9.0)		L	H/UC	H/UC	L
	Bespoke meta-analysis including[95;129]	Home exercise vs supervised exercise<br>SMD -0.32 (-0.62, -0.02), I^2 0% [in favour of home exercise]						
Self-efficacy	Siegel (2017) [SR][69]		Identified one previous systematic review[131] that reported home exercise was effective	Moderate				
	Hammond (2016) [SR][126]		Home exercises improved self-reported self-efficacy in the short-term	Moderate				
CRP	Hsieh (2009) [RCT][129]	Home vs supervised exercise at 8 weeks<br>SMD -0.09 (-0.80, 0.63)	CRP, BL / 8 weeks, mean (SD)<br>Supervised exercise: 1.66 (2.35) / 1.70 (2.71)<br>Home exercise: 1.55 (1.72) / 1.50 (1.80)		L	L	H/UC	L
	Neuberger (2007) [RCT][95]	Home exercise vs group exercise at 12 weeks<br>SMD -0.17 (-0.49, 0.16)	CRP, BL / 12 weeks, mean (SD)<br>Group exercise: 1.3 (2.0) / 1.1 (1.4)<br>Home exercise: 1.3 (1.6) / 0.9 (1.0)<br>Control: 1.3 (1.8) / 1.0 (1.2)		L	H/UC	H/UC	L
	Bespoke meta-analysis including[95;129]	Home exercise vs supervised exercise<br>SMD -0.15 (-0.45, 0.14), I^2 0% [in favour of home exercise]						

AIMS = Arthritis Impact Measurement Scales, Alloc. Conc. = allocation concealment, AMSTAR2 = Assessing the Methodological Quality of Systematic Reviews, BL = baseline, Blind. Asses. = Blinded assessors, Blind. Part. = blinded participants, CES-D = Centre for Epidemiologic Studies – Depression scale, CI = confidence interval, CRP = C-reactive protein, H/UC = high / unclear risk of bias, L = low risk of bias, RA = rheumatoid arthritis, Rand. Seq. = random sequence generation, RCT = randomised controlled trial, SD = standard deviation, SMD = standardised mean difference, SR = systematic review

Table – Home exercise (RA), results and quality assessment

Outcome (outcome measure)	Study (date) [study type]	Standardised result, SMD (95% CI) unless otherwise stated	Natural result	AMSTAR2 quality	Rand. Seq.	Alloc. Conc.	Blind. Part.	Blind. Asses.
ESR	Hsieh (2009) [RCT][129]	Home vs supervised exercise at 8 weeks<br>SMD -0.41 (-1.13, 0.32)	ESR, BL / 8 weeks, mean (SD)<br>Supervised exercise: 50.00 (31.20) / 53.20 (30.60)<br>Home exercise: 45.70 (31.40) / 40.70 (31.20)		L	L	H/UC	L
	Neuberger (2007) [RCT][95]	Home exercise vs group exercise at 12 weeks<br>SMD -0.44 (-0.77, -0.11)	ESR, BL / 12 weeks, mean (SD)<br>Group exercise: 32.5 (22.7) / 32.0 (24.2)<br>Home exercise: 23.7 (25.6) / 21.9 (21.9)<br>Control: 27.6 (24.1) / 26.8 (23.4)		L	H/UC	H/UC	L
	Bespoke meta-analysis including[95;129]	Home exercise vs supervised exercise<br>SMD -0.43 (-0.73, -0.13), I^2 0% [in favour of home exercise]						
Grip strength	Hammond (2016) [SR][126]		Home exercises improved grip strength in the short-term	Moderate				
	Hsieh (2009) [RCT][129]	Home vs supervised exercise at 8 weeks<br>SMD 0.37 (-0.35, 1.09)	Grip strength, BL / 8 weeks, mean (SD)<br>Supervised exercise: 10.46 (2.66) / 12.00 (3.70)<br>Home exercise: 12.27 (4.93) / 13.70 (5.37)		L	L	H/UC	L
	Neuberger (2007) [RCT][95]	Home exercise vs group exercise at 12 weeks<br>Left: SMD 0.10 (-0.23, 0.42)<br>Right: SMD 0.05 (-0.28, 0.37)	Grip strength, BL / 12 weeks, mean (SD)<br>*Left*<br>Group exercise: 117.4 (46.8) / 138.8 (54.6)<br>Home exercise: 134.7 (59.4) / 144.7 (63.8)<br>Control: 134.8 (56.6) / 138.1 (59.5)<br>*Right*<br>Group exercise: 121.4 (52.4) / 141.8 (56.6)<br>Home exercise: 130.9 (58.7) / 144.8 (64.9)<br>Control: 133.4 (58.7) / 143.0 (60.3)		L	H/UC	H/UC	L
	Bespoke meta-analysis including[95;129]	Home exercise vs supervised exercise<br>SMD 0.10 (-0.19, 0.40), I^2 0% [in favour of home exercise]						
Walk-test	Hsieh (2009) [RCT][129]	Home vs supervised exercise at 8 weeks<br>SMD -0.34 (-1.06, 0.39)	50ft walk test, BL / 8 weeks, mean (SD)<br>Supervised exercise: 12.47 (2.66) / 11.58 (2.17)<br>Home exercise: 11.87 (2.11) / 10.90 (1.86)		L	L	H/UC	L
	Neuberger (2007) [RCT][95]	Home exercise vs group exercise at 12 weeks<br>SMD 0.03 (-0.30, 0.35)	50ft walk test, BL / 12 weeks, mean (SD)<br>Group exercise: 10.0 (3.1) / 9.3 (2.8)<br>Home exercise: 9.6 (5.2) / 9.4 (4.4)<br>Control: 9.4 (2.8) / 10.0 (3.9)		L	H/UC	H/UC	L
	Bespoke meta-analysis including[95;129]	Home exercise vs supervised exercise<br>SMD -0.03 (-0.33, 0.26), I^2 0% [in favour of home exercise]						

Alloc. Conc. = allocation concealment, AMSTAR2 = Assessing the Methodological Quality of Systematic Reviews, BL = baseline, Blind. Asses. = Blinded assessors, Blind. Part. = blinded participants, CI = confidence interval, ESR = erythrocyte sedimentation rate, H/UC = high / unclear risk of bias, L = low risk of bias, RA = rheumatoid arthritis, Rand. Seq. = random sequence generation, RCT = randomised controlled trial, SD = standard deviation, SMD = standardised mean difference, SR = systematic review

Table – Home exercise (RA), SF36 results at final follow-up, mean (SD)

Author (date)	PCS	MCS	GH	PF	RP	RE	SF	BP	V	MH
Seneca (2015) [supervised][128]	1.5 (6.1) §	4.0 (6.4) §								
Seneca (2015) [home][128]	4.4 (6.6) §	-0.6 (10.5) §								

§ change from BL to 12 weeks

BL = baseline, BP = bodily pain, GH = general health, MCS = mental component score, MH = mental health, PCS = physical component score, PF = physical function, RE = role emotional, RP = role physical, SD = standard deviation, SF = social functioning, V = vitality

Supplementary table 42 – Description of reviews of studies of muscle strengthening exercise in RA

Table – Muscle strengthening exercise (RA), description of reviews

Authors (date)	Review type	Study type included	Exposure detail	Number of studies included	Funders
Williams (2018)[132]	MA	RCTs	Hand muscle strengthening exercises	7	**University** (University of Oxford, University of Warwick, University of Ottawa), **Government** (NIHR, NIHR CLAHRC)
Daien (2017)[133]	SR	RCTs	Hand muscle strengthening exercises and upper extremity exercise training	2	**Professional body** (EULAR)
Siegel (2017)[69]	SR	Reviews, RCTs, observational	Muscle strengthening exercise	2	**Not reported**
Bergstra (2014)[134]	SR	RCTs	Hand muscle strengthening exercises	8	**Not reported**

CLAHRC = Collaboration for Leadership in Applied Health Research & Care, EULAR = European League Against Rheumatism, MA = meta-analysis, NIHR = National Institute for Health Research, RA = rheumatoid arthritis, RCT = randomised controlled trial, SR = systematic review

Supplementary table 43 – Description of original studies of muscle strengthening exercise in RA

Table – Muscle strengthening exercises (RA), description of included studies

Author (date) [country]	Study design	Inclusion criteria	Exposure detail	N	Age years, mean (SD)	N (%) female	Funders
Lo (2017) [Hong Kong][135]	RCT	1987 ACR RA criteria, aged >18 years, symptom duration >1 year<br>Exclusions: Severe joint pain affecting mobility, currently receiving physiotherapy, recent joint injections, change in steroids in last 3 months, poor balance, pain preventing exercise	1) "Nerve mobilisation exercises" – range of motion and stretching exercises<br>p) Joint mobilization exercises not reaching full range of motion	1) 5<br>p) 4	1) 55.2 (11.4)<br>p) 59.8 (6.3)	1) 5 (100)<br>p) 3 (75.0)	**Not reported** - authors declared no conflict of interest
Williamson (2017) [UK][136]	RCT	Long-term follow-up. See Lamb et al 2015.	See Lamb et al 2015	1) 155<br>p) 173	1) 62.9 (11.0)<br>p) 64.3 (10.8)	1) 77.4%<br>p) 74.0%	**Government** (National Institute for Health Research)
Lourenzi (2017) [Brazil][137]	RCT	1987 ACR RA criteria, aged 18-65 years, stable medication for 3 months<br>Exclusions: difficulty understanding assessments, diagnosed with fibromyalgia, joint deformities that does not allow exercises, conditions that contraindicate exercise	1) Progressive muscle strengthening program for 12 weeks<br>p) Waitlist control	1) 27<br>p) 33	1) 52.6 (7.1)<br>p) 50.9 (8.6)	1) 25 (92.6)<br>p) 30 (90.9)	**Charity** (Sao Paulo Research Foundation)
Lamb (2015) [UK][138]	RCT	1987 ACR RA criteria, active pain and dysfunction in hand, stable DMARDs for 3 months<br>Exclusions: upper limb surgery or fracture in the previous 6 months, pregnancy, waiting upper limb surgery	1) Daily, home-based hand mobility and strength exercises + 6 face-to-face sessions with physio<br>p) Usual care	1) 246<br>p) 242	1) 61.3 (12)<br>p) 63.5 (11)	1) 188 (76.4)<br>p) 186 (76.9)	**Government** (National Institute for Health Research)
Manning (2014) [UK][139]	RCT	1987 ACR RA criteria, aged >18 years, symptom duration ≤5 years, no contraindications for upper extremity exercises<br>Exclusions: intramuscular or upper extremity intraarticular steroid injection in previous 4 weeks, upper extremity surgery/physiotherapy in last 6 months	1) Exercise circuit of 6 upper extremity exercises selected from set of 16 strengthening exercises. Also participated in group discussion about RA and exercise<br>p) Usual care	1) 52<br>p) 56	1) 53 (16)<br>p) 57 (15)	1) 44 (84.6)<br>p) 38 (67.9)	**Charity** (Physiotherapy Research Foundation)
Dogu (2013) [Turkey][140]	RCT	1987 ACR RA criteria, aged 40-70 years, functional class I-III<br>Exclusions: carpal tunnel and cubital syndrome, polyneuropathies, pregnant patients, patients having undergone hand surgery, active arthritis of hand joints	1) Isotonic muscle strengthening exercise<br>2) Isometric muscle strengthening exercise	1) 23<br>p) 24	1) 54.9 (9.3)<br>p) 50.4 (9.3)	1) 23 (100)<br>p) 24 (100)	**Not reported** - authors declared no conflict of interest

ACR = American College of Rheumatology, DMARDs = Disease Modifying Anti-Rheumatic Drugs, N = number, RA = rheumatoid arthritis, RCT = randomised controlled trial, SD = standard deviation, UK = United Kingdom

Table – Muscle strengthening exercises (RA), description of included studies

Author (date) [country]	Study design	Inclusion criteria	Exposure detail	N	Age years, mean (SD)	N (%) female	Funders
Lemmey (2012) [UK][122]	RCT	1987 ACR RA criteria, aged ≥18 years, functional class I-II, not cognitively impaired, stable drug therapy, steroid dose <10mg/day, Free of other cachectic diseases (e.g. cancer, HIV, infection), free of medical conditions contraindicating regular high intensity exercise, not taking drugs or nutritional supplements known to be anabolic, not undertaking regular, intense physical training, not pregnant	1) High intensity progressive muscle strengthening training<br>2) Home-based, low-intensity range of motion exercises	1) 9<br>p) 9	1) 55.7 (8.6)<br>2) 59.4 (10.8)	1) 8 (88.9)<br>p) 6 (66.7)	**Charity** (Arthritis Research Campaign)
Baillet (2009) [France][141]	RCT	1987 ACR RA criteria, treated with DMARDs, ≥45 years.<br>Exclusions: treatment with >10mg glucocorticoid per day, unstable DMARD regime, DAS28 variation >1.2 in past 3 months, aged <18 or >70 years, functional class III or IV, unable to follow educational programme	1) Training programme designed to improve muscle strength, flexibility, endurance and balance. Exercise of upper and lower limbs performed 5x per week at gym.<br>p) Conventional rehabilitation including lectures on disease management and hydrotherapy	1) 25<br>p) 23	1) 51.6 (8.3)<br>p) 56.3 (12.8)	1) 21 (84.0)<br>p) 18 (78.3)	**Not reported** - authors declared no conflict of interest
Lemmey (2009) [UK][123]	RCT	See Lemmey 2012	1) High intensity progressive muscle strengthening training<br>2) Home-based, low-intensity range of motion exercises	1) 13<br>p) 15	1) 55.6 (8.3)<br>2) 60.6 (11.2)	1) 11 (84.6)<br>p) 12 (80.0)	**Charity** (Arthritis Research Campaign)
Masiero (2007) [Italy][142]	RCT	1987 ACR RA criteria, treated with anti-tumour necrosis factor agent, aged 18-65 years, stable drug therapy for 6 months, no severe disability<br>Exclusions: participation in previous educational program, changes to drug treatment during trial, rehabilitation treatment / orthopaedic surgery during trial	1) Home based exercise program including upper and lower stretching and range of motion exercises as well as information on pathophysiology of RA, pain and stress mechanisms, importance of rest, principles of joint protection, assistive equipment<br>p) Usual care	1) 36<br>p) 34	1) 54.2 (9.8)<br>p) 52.2 (11.9)	1) 29 (80.6)<br>p) 28 (82.4)	**Not reported**
O'Brien (2006) [UK][143]	RCT	Aged >18 years, 1987 ACR RA criteria, stable DMARDs for 3 months<br>Exclusions: >7.5mg/day steroids or intramuscular injection within 1 month	1) Received joint protection leaflet + instructions on how to perform 8 strengthening and mobilizing hand exercises<br>2) Received joint protection leaflet + instructions for 8 stretching exercises<br>p) Non-exercise control	1) 21<br>2) 24<br>p) 22	1) 62.3 (10.0)<br>2) 57.3 (8.2)<br>p) 59.5 (12.9)	1) 15 (71.4)<br>2) 15 (62.5)<br>p) 16 (72.7)	**Industry** (Promedics, UK), **Professional body** (Birmingham Branch of the Chartered Society of Physiotherapy), **Charity** (Arthritis Research Campaign)
Buljina (2001) [Bosnia and Herzegovina][144]	RCT	Aged 20-70 years, 1987 ACR RA criteria, symptom duration ≥6 months, ≥3 swollen joints on both hands, ≥5 tender joints on both hands, decreased range of hand motion, ESR >25	1) Resistive hand exercises – 20-30 minutes, radon baths and wax bath treatments<br>p) Waitlist control	1) 50<br>p) 50	1) 47.9 (11.2)<br>p) 48.5 (10.7)	1) 38 (76.0)<br>p) 37 (74.0)	**Not reported**
Scholten (1999) [Austria][145]	RCT	Definite RA	1) Feasible therapeutic exercises preserving axis of joints and reinforcing weakened muscles<br>p) Waitlist control	1) 38<br>p) 30	48.3 (5.6)	54 (79.4)	**Government** (Mayor of Vienna grant)

ACR = American College of Rheumatology, DMARDs = Disease Modifying Anti-Rheumatic Drugs, ESR = erythrocyte sedimentation rate, HIV = Human Immunodeficieny Virus, N = number, RA = rheumatoid arthritis, RCT = randomised controlled trial, SD = standard deviation, UK = United Kingdom, USA = United States of America

Table – Muscle strengthening exercises (RA), description of included studies

Author (date) [country]	Study design	Inclusion criteria	Exposure detail	N	Age years, mean (SD)	N (%) female	Funders
Bostrom (1998) [Sweden][146]	RCT	1987 ACR RA criteria, aged >20 years, pain in shoulder/arm region and/or functional limitations in shoulder region	1) Dynamic shoulder arm resistive exercises<br>2) Static shoulder arm resistive exercises	1) 17<br>p) 20	Mean (range)<br>1) 59 (42-74)<br>p) 56 (24-74)	1) 17 (100)<br>p) 20 (100)	**University** (Karolinska), **Professional body** (Swedish Association against Rheumatism), **Government** (Swedish Medical Research Council, Swedish Foundation for Health Care Sciences and Allergy Research)
Komatireddy (1997) [USA][147]	RCT	Classical or definite RA, mild to moderate disease activity, ACR functional class II-III<br>Exclusions: symptomatic chest pain, abnormal electrocardiograms and stress tests, symptomatic dyspnea or prior pulmonary function abnormalities	1) Moderate intensity exercise using weights, dumbbells, elastic bands, 2x per week for four weeks and then increased to 3x per week<br>p) No exercise	1) 24<br>p) 25	1) 60.5 (11)<br>p) 57.7 (9.8)	1) 17 (70.8)<br>p) 20 (80.0)	**University** (University of Missouri-Columbia)
Hoenig (1993) [USA][148]	RCT	Definite or classical RA, stable medication for 6 weeks, functional class II-III	1) Range of motion hand exercises<br>2) Muscle strengthening hand exercises<br>3) Range of motion + muscle strengthening hand exercises<br>p) Control – encourage to maintain active lifestyle	1) 11<br>2) 9<br>3) 10<br>p) 11	Not reported	Not reported	**Charity** (Bassett Research Foundation), **Industry** (Fred Sammons, Inc.)
Dellhag (1992) [Sweden][149]	RCT	Resident of Gothenburg, aged <70 years, symptom duration 6-10 years, functional class I-II, hand problems, seropositive<br>Exclusions: other diagnoses	1) Eight different hand exercises using soft exercise ball<br>p) Non-exercise control	1) 11<br>p) 13	Women: 51.8<br>Men: 56.3	Not reported	**Professional body** (Swedish Rheumatism Association)
Marcora (2005) [UK][150]	NRT	1987 ACR RA criteria, functional class I-II, aged ≥18 years, no cognitive impairment, stable drug therapy for past 3 months, free of other cachectic diseases and any condition preventing safe participation in the study, no participation in another regular or intense exercise program, not pregnant	1) Progressive muscle strengthening training for 12 weeks<br>p) Usual care	1) 10<br>p) 10	1) 53 (13)<br>p) 54 (10)	1) 6 (60.0)<br>p) 6 (60.0)	**Government** (Wales Office of Research and Development for Health and Social Care)
Goksel Karatepe (2011) [Turkey][151]	Single arm int.	1987 ACR RA criteria, stable disease for 3 months, low-moderate disease activity, functional class I-II, no significant cardiovascular disease, no recent joint replacement surgery, no planned lower extremity surgery, did not participate in regular exercise before program	4 weeks home based exercise – strengthening and range of motion, twice daily	28	52.9 (8.6)	25 (89.3)	**Not reported**

ACR = American College of Rheumatology, N = number, NRT = Non-randomised trial, RA = rheumatoid arthritis, RCT = randomised controlled trial, SD = standard deviation, UK = United Kingdom, USA = United States of America

Supplementary table 44 – Results from reviews and interventional studies of muscle strengthening exercise in RA

Table – Muscle strengthening exercise (RA), results and quality assessment

Outcome (outcome measure)	Study (date) [study type]	Standardised result, SMD (95% CI) unless otherwise stated	Natural result	AMSTAR2 quality	Rand. Seq.	Alloc. Conc.	Blind. Part.	Blind. Asses.
Pain	Williams (2018) [MA][132]		Pain, Mean difference (95% CI)<br>-3.70 (-8.10, 0.70)	High				
	Siegel (2017) [SR][69]		1 MA[152] reported no improvement in pain (MD -4.13 [-10.97, 2.71])	Moderate				
	Bergstra (2014) [SR][134]		No significant increase in pain	Low				
	Lo (2017) [RCT][135]	Exercise vs control (effect size reversed – negative scores in favour of exercise)<br>SMD -0.77 (-2.15, 0.61)	RA pain scale (high scores are better), BL / 4 weeks, mean (SD) ‡<br>Exercise: 93.8 (13.2) / 110.2 (16.2)<br>Control: 109 (19.9) / 96.8 (18.9)		H/UC	H/UC	H/UC	H/UC
	Lourenzi (2017) [RCT][137]	Exercise vs control at 12 months<br>SMD -0.27 (-0.78, 0.24)	Pain VAS, 6 months / 12 months, mean (SD)<br>Exercise: 4.3 (1.6) / 3.4 (1.9)<br>Control: 3.9 (1.8) / 3.9 (1.8)		L	L	H/UC	L
	Lamb (2015) [RCT][138]	Hand Exercise vs control, mean change BL-12 weeks<br>SMD -0.11 (-0.28, 0.07)	MHQ Pain, change from BL – 12 months, mean (SD †)<br>Hand exercise: -8.26 (20.5)<br>Control: -6.01 (21.6)		L	L	H/UC	L
	Manning (2014) [RCT][139]	Exercise vs control, change from BL-12 months<br>SMD -0.41 (-0.80, -0.03)	Pain, change BL-12 weeks, mean (SD †)<br>Exercise: -13.8 (36.4)<br>Control: 1.7 (38.4)		L	L	H/UC	L
	Dogu (2013) [RCT][140]	Isotonic vs isometric exercise, change from BL-6 weeks<br>SMD 0.09 (-0.48, 0.66)	Pain VAS, Change BL-6 weeks, mean (SD)<br>Isotonic: -1.26 (2.68)<br>Isometric: -1.04 (2.13)		L	H/UC	H/UC	L
	Masiero (2007) [RCT][142]		Pain VAS, BL / 8 months, mean (SD)<br>Exercise: 46 (21.6) / 36.9 (26.3)<br>Control: 39 (26.9) / 4.2 (16.4) [sic]		H/UC	H/UC	H/UC	L
	Buljina (2001) [RCT][144]	Hand exercise vs control at 4 weeks<br>SMD -2.21 (-2.71, -1.71)	Pain VAS, BL / 4 weeks, mean (SD)<br>Hand exercises: 66.4 (17.0) / 32.4 (14.8)<br>Control: 67.6 (17.5) / 70.0 (19.0)		L	H/UC	H/UC	H/UC
	Bostrom (1998) [RCT][146]	Dynamic vs static exercise at 10 weeks<br>SMD -0.21 (-0.86, 0.44)	Shoulder pain at rest, BL / 10 weeks, mean (SD §)<br>Dynamic: 1.3 (1.9) / 0.5 (1.2)<br>Static: 0.9 (1.6) / 0.8 (1.6)		H/UC	H/UC	H/UC	L
	Komatireddy (1997) [RCT][147]	Exercise vs control at 12 weeks<br>SMD -0.21 (-0.77, 0.35)	Pain, BL / 12 weeks, mean (SD)<br>Exercise: 3.5 (1.6) / 4.2 (2.6)<br>Control: 4.0 (2.2) / 4.7 (2.2)		H/UC	H/UC	H/UC	L
	Dellhag (1992) [RCT][149]		Hand pain when moving, BL / 4 weeks, mean<br>Exercise: 28.8 / 17.0<br>Control: 27.7 / 33.1		H/UC	H/UC	H/UC	H/UC
	Bespoke meta-analysis including [135;137;138;144;147]	Exercise vs control<br>SMD -0.68 (-1.55, 0.19), I^2 94%<br>Removing outlier[144]: SMD -0.06 (-0.22, 0.10), I^2 0%<br>Non-hand exercises: SMD -0.28 (-0.64, 0.09), I^2 0%						

§ Mean (SD) estimated from median (interquartile range) using published formula[87], † SD calculated from 95% confidence interval,
Alloc. Conc. = allocation concealment, AMSTAR2 = Assessing the Methodological Quality of Systematic Reviews, BL = baseline, Blind. Asses. = Blinded assessors, Blind. Part. = blinded participants, CI = confidence interval, H/UC = high / unclear risk of bias, L = low risk of bias, RA = rheumatoid arthritis, Rand. Seq. = random sequence generation, RCT = randomised controlled trial, SD = standard deviation, SMD = standardised mean difference

Table – Muscle strengthening exercise (RA), results and quality assessment

Outcome (outcome measure)	Study (date) [study type]	Standardised result, SMD (95% CI) unless otherwise stated	Natural result	AMSTAR2 quality	Rand. Seq.	Alloc. Conc.	Blind. Part.	Blind. Asses.
Function	Williams (2018) [MA][132]	Exercise vs control, short term<br>SMD 0.79 (0.42, 1.17)		High				
	Siegel (2017) [SR][69]		1 MA[152] reported improvements in function (MD -0.22 [-0.35, -0.10])	Moderate				
	Williamson (2017) [RCT][136]	Exercise vs control at 26 months<br>SMD 0.09 (-0.13, 0.30)	SF12 (physical), change from BL – 26 months, mean (SD †)<br>Hand exercise: 0.19 (8.6)<br>Control: -0.51 (7.7)		L	L	H/UC	L
	Lourenzi (2017) [RCT][137]	Exercise vs control at 12 months<br>SMD -0.37 (-0.89, 0.14)	HAQ, 6 months / 12 months, mean (SD)<br>Exercise: 0.88 (0.54) / 0.69 (0.49)<br>Control: 0.90 (0.48) / 0.87 (0.48)		L	L	H/UC	L
	Lamb (2015) [RCT][138]	Hand Exercise vs control, mean change BL-12 weeks<br>SMD 0.15 (-0.03, 0.33)	SF12 (physical), change from BL – 12 months, mean (SD †)<br>Hand exercise: 1.19 (7.64)<br>Control: 0.03 (7.90)		L	L	H/UC	L
	Dogu (2013) [RCT][140]	Isotonic vs isometric exercise, change from BL-6 weeks<br>SMD -0.06 (-0.64, 0.51)	Duroz Hand Function Index, Change BL-6 weeks, mean (SD)<br>Isotonic: 2.83 (3.71)<br>Isometric: 3.06 (3.60)		L	H/UC	H/UC	L
	Baillet (2009) [RCT][141]	Exercise vs control at 12 months<br>SMD 0.00 (-0.57, 0.57)	HAQ, BL / 1 month, mean (SD)<br>Exercise: 0.9 (0.6) / 0.7 (0.6)<br>Control: 0.7 (0.5) / 0.7 (0.6)		L	L	H/UC	L
	Lemmey (2009) [RCT][123]	Exercise vs control at 24 weeks<br>SMD 0.38 (-0.37, 1.13)	HAQ. BL / 24 weeks, mean (SD)<br>Exercise: 0.91 (0.68) / 0.82 (0.69)<br>Control: 0.58 (0.62) / 0.58 (0.59)		L	H/UC	H/UC	H/UC
	Masiero (2007) [RCT][142]	Exercise vs control at 8 months<br>SMD -0.63 (-1.11, -0.15)	HAQ, BL / 8 months, mean (SD)<br>Exercise: 1.20 (0.56) / 0.93 (0.44)<br>Control: 1.17 (0.57) / 1.24 (0.54)		H/UC	H/UC	H/UC	L
	O'Brien (2006) [RCT][143]	Muscle strengthening hand exercise vs control, change from BL-6 months<br>SMD 1.13 (0.49, 1.78)<br>Stretching hand exercise vs control, change from BL-6 months<br>SMD 0.09 (-0.49, 0.67)	AIMS upper limb function, change BL-6 months, mean (SD)<br>Muscle strengthening hand exercise: 1.00 (1.07)<br>Stretching hand exercise: -0.18 (1.54)<br>Control: -0.30 (1.22)		L	L	H/UC	L

† SD calculated from 95% confidence interval, ‡ Mean (SD) calculated by reviewers from data in the paper

Alloc. Conc. = allocation concealment, AMSTAR2 = Assessing the Methodological Quality of Systematic Reviews, BL = baseline, Blind. Asses. = Blinded assessors, Blind. Part. = blinded participants, CI = confidence interval, H/UC = high / unclear risk of bias, HAQ = Health Assessment Questionnaire, L = low risk of bias, RA = rheumatoid arthritis, Rand. Seq. = random sequence generation, RCT = randomised controlled trial, SD = standard deviation, SMD = standardised mean difference

Table – Muscle strengthening exercise (RA), results and quality assessment

Outcome (outcome measure)	Study (date) [study type]	Standardised result, SMD (95% CI) unless otherwise stated	Natural result	AMSTAR2 quality	Rand. Seq.	Alloc. Conc.	Blind. Part.	Blind. Asses.
Function	Scholten (1999) [RCT][145]	Exercise vs control at 1 year<br>SMD -0.78 (-1.27, -0.28)	HAQ (score 1-5), BL / 1 year, mean (SD)<br>Exercise: 2.6 (0.78) / 2.2 (0.32)<br>Control: 2.9 (0.62) / 2.6 (0.69)		H/UC	H/UC	H/UC	H/UC
	Bostrom (1998) [RCT][146]	Dynamic vs static exercise at 10 weeks<br>SMD 0.25 (-0.40, 0.90)	HAQ, BL / 10 weeks, mean (SD §)<br>Dynamic: 1.21 (0.36) / 1.11 (0.45)<br>Static: 1.19 (0.49) / 0.97 (0.64)		H/UC	H/UC	H/UC	L
	Komatireddy (1997) [RCT][147]	Exercise vs control at 12 weeks<br>SMD 0.58 (0.01, 1.15)	HAQ, BL / 12 weeks, mean (SD)<br>Exercise: 13.0 (7.5) / 8.9 (6.6)<br>Control: 5.8 (5.8) / 5.0 (6.8)		H/UC	H/UC	H/UC	L
	Bespoke meta-analysis including [123;137;138;141-143;145;147]	Exercise vs control<br>SMD -0.27 (-0.60, 0.05), I^2 73.8%<br>Non-hand exercises: SMD -0.17 (-0.60, 0.26), I^2 72.9%						
	Goksel Karatepe (2011) [single arm int.][151]		HAQ, BL / 4 weeks, mean (SD)<br>1.6 (0.8) / 1.2 (0.7)					
	Marcora (2005) [NRT][150]	Exercise vs control at 12 weeks<br>SMD 0.00 (-0.88, 0.88)	MHAQ, BL / 12 weeks, mean (SD)<br>Exercise: 1.3 (0.3) / 1.3 (0.2)<br>Control: 1.5 (0.6) / 1.3 (0.4)					

§ Mean (SD) estimated from median (interquartile range) using published formula[87],
Alloc. Conc. = allocation concealment, AMSTAR2 = Assessing the Methodological Quality of Systematic Reviews, BL = baseline, Blind. Asses. = Blinded assessors, Blind. Part. = blinded participants, CI = confidence interval, H/UC = high / unclear risk of bias, HAQ = Health Assessment Questionnaire, L = low risk of bias, NRT = non-randomised trial, RA = rheumatoid arthritis, Rand. Seq. = random sequence generation, RCT = randomised controlled trial, SD = standard deviation, SMD = standardised mean difference

Table – Muscle strengthening exercise (RA), results and quality assessment

Outcome (outcome measure)	Study (date) [study type]	Standardised result, SMD (95% CI) unless otherwise stated	Natural result	AMSTAR2 quality	Rand. Seq.	Alloc. Conc.	Blind. Part.	Blind. Asses.
Disease activity	Bergstra (2014) [SR][134]		2/3 studies found no association between hand exercise and disease activity	Low				
	Lourenzi (2017) [RCT][137]	Exercise vs control at 12 months<br>SMD -0.11 (-0.62, 0.40)	DAS28, 6 months / 12 months, mean (SD)<br>Exercise: 3.93 (1.21) / 3.67 (1.17)<br>Control: 3.94 (1.06) / 3.79 (0.99)		L	L	H/UC	L
	Manning (2014) [RCT][139]	Exercise vs control, change from BL-12 months<br>SMD -0.33 (-0.71, 0.05)	DAS28, change BL-12 weeks, mean (SD †)<br>Exercise: -0.8 (2.2)<br>Control: -0.1 (2.1)		L	L	H/UC	L
	Dogu (2013) [RCT][140]	Isotonic vs isometric exercise, change from BL-6 weeks<br>SMD -0.08 (-0.66, 0.49)	DAS28, Change BL-6 weeks, mean (SD)<br>Isotonic: -0.70 (1.08)<br>Isometric: -0.78 (0.80)		L	H/UC	H/UC	L
	Baillet (2009) [RCT][141]	Exercise vs control at 12 months<br>SMD -0.11 (-0.67, 0.46)	DAS28, BL / 1 month, mean (SD)<br>Exercise: 4.9 (1.4) / 3.8 (2.1)<br>Control: 4.0 (1.7) / 4.0 (1.6)		L	L	H/UC	L
	Lemmey (2009) [RCT][123]	Exercise vs control at 24 weeks<br>SMD -0.42 (-1.17, 0.33)	DAS28. BL / 24 weeks, mean (SD)<br>Exercise: 3.29 (1.27) / 3.12 (1.34)<br>Control: 3.28 (1.27) / 3.56 (0.71)		L	H/UC	H/UC	H/UC
	Bespoke meta-analysis including[123;137;139;141]	Exercise vs control<br>SMD -0.24 (-0.49, 0.01), I^2 0%						
	Marcora (2005) [NRT][150]	Exercise vs control at 12 weeks<br>SMD 0.21 (-1.09, 0.67)	RADAI, BL / 12 weeks, mean (SD)<br>Exercise: 2.5 (1.1) / 2.0 (1.4)<br>Control: 2.8 (1.7) / 2.3 (1.5)					
	Goksel Karatepe (2011) [single arm int.][151]		DAS28, BL / 4 weeks, mean (SD)<br>4.4 (1.2) / 4.0 (1.2)					

† SD calculated from 95% confidence interval

Alloc. Conc. = allocation concealment, AMSTAR2 = Assessing the Methodological Quality of Systematic Reviews, BL = baseline, Blind. Asses. = Blinded assessors, Blind. Part. = blinded participants, CI = confidence interval, DAS28 = Disease Activity Score 28, H/UC = high / unclear risk of bias, L = low risk of bias, NRT = non-randomised trial, RA = rheumatoid arthritis, RADAI = Rheumatoid Arthritis Disease Activity Index, Rand. Seq. = random sequence generation, RCT = randomised controlled trial, SD = standard deviation, SMD = standardised mean difference

Table – Muscle strengthening exercise (RA), results and quality assessment

Outcome (outcome measure)	Study (date) [study type]	Standardised result, SMD (95% CI) unless otherwise stated	Natural result	AMSTAR2 quality	Rand. Seq.	Alloc. Conc.	Blind. Part.	Blind. Asses.
Joint count	Siegel (2017) [SR][69]		1 MA[152] reported improvements in joint count (MD -5.36 [-9.00, -1.72])	Moderate				
Tender joints	Lamb (2015) [RCT][138]	Hand Exercise vs control, mean change BL-12 weeks<br>SMD 0.03 (-0.14, 0.21)	Tender joint count, change from BL – 12 months, mean (SD †)<br>Hand Exercise: -0.96 (5.8)<br>Control: -1.15 (5.7)		L	L	H/UC	L
	Masiero (2007) [RCT][142]		Ritchie index, BL / 8 months, mean<br>Exercise: 18.8 / 17.9<br>Control: 21.4 / 22.1		H/UC	H/UC	H/UC	L
	O'Brien (2006) [RCT][143]	Muscle strengthening hand exercise vs control, change from BL-6 months<br>SMD 0.00 (-0.60, 0.60)<br>Stretching hand exercise vs control, change from BL-6 months<br>SMD -0.38 (-0.96, 0.21)	Tender joint count, BL / 6 months, mean (SD)<br>Muscle strengthening hand exercise: 5.1 (5.8) / 2.5 (3.7)<br>Stretching hand exercise: 3.4 (5.8) / 1.2 (2.1)<br>Control: 2.0 (2.5) / 2.5 (4.5)		L	L	H/UC	L
	Buljina (2001) [RCT][144]	Hand exercise vs control at 4 weeks<br>SMD -1.47 (-1.91, -1.03)	Hand Articular Index, BL / 4 weeks, mean (SD)<br>Hand exercises: 8.8 (3.5) / 4.2 (2.5)<br>Control: 8.4 (3.3) / 8.5 (3.3)		L	H/UC	H/UC	H/UC
	Bostrom (1998) [RCT][146]	Dynamic vs static exercise at 10 weeks<br>SMD -0.40 (-1.05, 0.25)	Ritchie Index, BL / 10 weeks, mean (SD §)<br>Dynamic: 6.2 (5.3) / 6.8 (5.3)<br>Static: 8.9 (5.7) / 8.6 (3.7)		H/UC	H/UC	H/UC	L
	Bespoke meta-analysis including [138;143;144]	Exercise vs control<br>SMD -0.48 (-1.46, 0.51), I^2 94.8%						

§ Mean (SD) estimated from median (interquartile range) using published formula[87]

† SD calculated from 95% confidence interval

Alloc. Conc. = allocation concealment, AMSTAR2 = Assessing the Methodological Quality of Systematic Reviews, BL = baseline, Blind. Asses. = Blinded assessors, Blind. Part. = blinded participants, CI = confidence interval, H/UC = high / unclear risk of bias, L = low risk of bias, RA = rheumatoid arthritis, Rand. Seq. = random sequence generation, RCT = randomised controlled trial, SD = standard deviation, SMD = standardised mean difference

Table – Muscle strengthening exercise (RA), results and quality assessment

Outcome (outcome measure)	Study (date) [study type]	Standardised result, SMD (95% CI) unless otherwise stated	Natural result	AMSTAR2 quality	Rand. Seq.	Alloc. Conc.	Blind. Part.	Blind. Asses.
Swollen joints	Lamb (2015) [RCT][138]	Hand Exercise vs control, mean change BL-12 weeks<br>SMD -0.02 (-0.20, 0.16)	Swollen joint count, change from BL – 12 months, mean (SD †)<br>Hand exercise: -1.13 (4.5)<br>Control: -1.02 (5.4)		L	L	H/UC	L
	O'Brien (2006) [RCT][143]	Muscle strengthening hand exercise vs control, change from BL-6 months<br>SMD -0.26 (-0.86, 0.35)<br>Stretching hand exercise vs control, change from BL-6 months<br>SMD -0.51 (-1.10, 0.07)	Swollen joint count, BL / 6 months, mean (SD)<br>Muscle strengthening hand exercise: 3.5 (3.0) / 2.3 (3.5)<br>Stretching hand exercise: 3.0 (3.6) / 1.4 (1.8)<br>Control: 3.1 (4.6) / 3.3 (5.6)		L	L	H/UC	L
	Buljina (2001) [RCT][144]	Hand exercise vs control at 4 weeks<br>SMD -0.49 (-0.88, -0.09)	PIP joint size, BL / 4 weeks, mean (SD)<br>Hand exercises: 21.2 (3.5) / 19.6 (3.3)<br>Control: 20.6 (3.4) / 21.2 (3.3)		L	H/UC	H/UC	H/UC
	Bostrom (1998) [RCT][146]	Dynamic vs static exercise at 10 weeks<br>SMD 0.07 (-0.58, 0.71)	Swollen joint count, BL / 10 weeks, mean (SD §)<br>Dynamic: 9.5 (7.7) / 7.3 (4.9)<br>Static: 11.7 (8.4) / 7.0 (4.4)		H/UC	H/UC	H/UC	L
	Komatireddy (1997) [RCT][147]	Exercise vs control at 12 weeks<br>SMD -0.73 (-1.30, -0.15)	Swollen joint count, BL / 12 weeks, mean (SD)<br>Exercise: 7.9 (6.3) / 6.8 (8.6)<br>Control: 11.6 (8.8) / 15.8 (15.2)		H/UC	H/UC	H/UC	L
	Bespoke meta-analysis including [138;143;144;147]	Exercise vs control<br>SMD -0.20 (-0.46, 0.07), I^2 45.5%						
Fatigue	Manning (2014) [RCT][139]	Exercise vs control, change from BL-12 weeks<br>SMD -0.23 (-0.61, 0.15)	Fatigue VAS, change BL-12 weeks, mean (SD †)<br>Exercise: -7.9 (38.4)<br>Control: 1.2 (39.5)		L	L	H/UC	L
	Komatireddy (1997) [RCT][147]	Exercise vs control at 12 weeks<br>SMD -0.19 (-0.75, 0.38)	Fatigue, BL / 12 weeks, mean (SD)<br>Exercise: 3.7 (1.8) / 4.5 (2.4)<br>Control: 4.6 (2.5) / 4.9 (1.9)		H/UC	H/UC	H/UC	L
	Bespoke meta-analysis including[139;147]	Exercise vs control<br>SMD -0.22 (-0.53, 0.10), I^2 0%						
	Marcora (2005) [NRT][150]	Exercise vs control at 12 weeks<br>SMD -0.47 (-1.36, 0.42)	Fatigue VAS, BL / 12 weeks, mean (SD)<br>Exercise: 4.4 (1.8) / 3.1 (2.1)<br>Control: 4.9 (3.2) / 4.4 (3.3)					

§ Mean (SD) estimated from median (interquartile range) using published formula[87]
† SD calculated from 95% confidence interval
Alloc. Conc. = allocation concealment, AMSTAR2 = Assessing the Methodological Quality of Systematic Reviews, BL = baseline, Blind. Asses. = Blinded assessors, Blind. Part. = blinded participants, CI = confidence interval, H/UC = high / unclear risk of bias, L = low risk of bias, NRT = non-randomised trial, PIP = proximal interphalangeal joint, RA = rheumatoid arthritis, Rand. Seq. = random sequence generation, , RCT = randomised controlled trial, SD = standard deviation, SMD = standardised mean difference, VAS = Visual Analogue Scale

Table – Muscle strengthening exercise (RA), results and quality assessment

Outcome (outcome measure)	Study (date) [study type]	Standardised result, SMD (95% CI) unless otherwise stated	Natural result	AMSTAR2 quality	Rand. Seq.	Alloc. Conc.	Blind. Part.	Blind. Asses.
Patient global	O'Brien (2006) [RCT][143]	Muscle strengthening hand exercise vs control, change from BL-6 months<br>SMD -0.02 (-0.62, 0.58)<br>Stretching hand exercise vs control, change from BL-6 months<br>SMD -0.35 (-0.93, 0.24)	Patient global assessment, BL / 6 months, mean (SD)<br>Muscle strengthening hand exercise: 4.71 (2.12) / 4.25 (2.41)<br>Stretching hand exercise: 3.95 (2.62) / 3.41 (2.52)<br>Control: 3.37 (1.63) / 4.31 (2.71)		L	L	H/UC	L
QoL	Williamson (2017) [RCT][136]	Exercise vs control at 26 months<br>SMD 0.00 (-0.22, 0.22)	EQ5D, change from BL – 26 months, mean (SD †)<br>Hand exercise: -0.01 (0.25)<br>Control: -0.01 (0.23)		L	L	H/UC	L
	Lamb (2015) [RCT][138]	Hand Exercise vs control, mean change BL-12 weeks<br>SMD 0.04 (-0.14, 0.22)	EQ5D, change from BL – 12 months, mean (SD †)<br>Hand exercise: 0.03 (0.24)<br>Control: 0.02 (0.28)		L	L	H/UC	L
	Manning (2014) [RCT][139]	Exercise vs control, change from BL-12 weeks<br>SMD -0.09 (-0.47, 0.29)	RAQOL, change BL-12 weeks, mean (SD †)<br>Exercise: -1.4 (7.0)<br>Control: -0.8 (6.7)		L	L	H/UC	L
	Dogu (2013) [RCT][140]	Isotonic vs isometric exercise, change from BL-6 weeks<br>SMD -0.27 (-0.85, 0.31)	RAQOL, Change BL-6 weeks, mean (SD)<br>Isotonic: -4.09 (5.14)<br>Isometric: -6.04 (8.76)		L	H/UC	H/UC	L
	Baillet (2009) [RCT][141]	Exercise vs control at 12 months<br>SMD -0.30 (-0.87, 0.27)	AIMS2-SF, BL / 1 month, mean (SD)<br>Exercise: 21.2 (5.6) / 18.0 (5.7)<br>Control: 19.6 (4.9) / 19.9 (7.1)		L	L	H/UC	L
	Bespoke meta-analysis including[138;139;141]	Exercise vs control<br>SMD -0.01 (-0.16, 0.15), I^2 0%						
	Goksel Karatepe (2011) [single arm int.][151]		RAQOL, BL / 4 weeks, mean (SD)<br>20.8 (7.9) 18.0 (8.5)					

† SD calculated from 95% confidence interval

AIMS2-SF = Arthritis Impact Score 2 – Short Form, Alloc. Conc. = allocation concealment, AMSTAR2 = Assessing the Methodological Quality of Systematic Reviews, BL = baseline, Blind. Asses. = Blinded assessors, Blind. Part. = blinded participants, CI = confidence interval, H/UC = high / unclear risk of bias, L = low risk of bias, QoL = Quality of life, RA = rheumatoid arthritis, Rand. Seq. = random sequence generation, RAQOL = Rheumatoid Arthritis Quality of Life Index, RCT = randomised controlled trial, SD = standard deviation, SMD = standardised mean difference

Table – Muscle strengthening exercise (RA), results and quality assessment

Outcome (outcome measure)	Study (date) [study type]	Standardised result, SMD (95% CI) unless otherwise stated	Natural result	AMSTAR2 quality	Rand. Seq.	Alloc. Conc.	Blind. Part.	Blind. Asses.
Depression	Williamson (2017) [RCT][136]	Exercise vs control at 26 months<br>SMD 0.01 (-0.21, 0.22)	SF12 (mental), change from BL – 26 months, mean (SD †)<br>Hand exercise: 0.27 (9.6)<br>Control: 0.21 (9.7)		L	L	H/UC	L
	Lamb (2015) [RCT][138]	Hand Exercise vs control, mean change BL-12 weeks<br>SMD 0.16 (-0.02, 0.34)	SF12 (mental), change from BL – 12 months, mean (SD †)<br>Hand exercise: 2.19 (11.5)<br>Control: 0.41 (10.3)		L	L	H/UC	L
	Scholten (1999) [RCT][145]	Exercise vs control at 1 year<br>SMD -0.54 (-1.03, -0.05)	Beck Depression Inventory, BL / 1 year, mean (SD)<br>Exercise: 12.1 (6.2) / 9.6 (2.3)<br>Control: 12.0 (6.4) / 12.1 (6.5)		H/UC	H/UC	H/UC	H/UC
	Komatireddy (1997) [RCT][147]	Exercise vs control at 12 weeks<br>SMD -0.25 (-0.81, 0.31)	AIMS, BL / 12 weeks, mean (SD)<br>Exercise: 2.1 (1.3) / 1.6 (0.8)<br>Control: 2.0 (1.4) / 1.9 (1.5)		H/UC	H/UC	H/UC	L
	Bespoke meta-analysis including [138;145;147]	Exercise vs control<br>SMD -0.21 (-0.38, -0.05), I^2 1.7%						
Anxiety	Komatireddy (1997) [RCT][147]	Exercise vs control at 12 weeks<br>SMD -0.06 (-0.62, 0.50)	AIMS, BL / 12 weeks, mean (SD)<br>Exercise: 4.0 (1.9) / 3.2 (1.4)<br>Control: 3.3 (1.3) / 3.3 (2.0)		H/UC	H/UC	H/UC	L
Self-efficacy	Williamson (2017) [RCT][136]	Exercise vs control at 26 months<br>SMD 0.19 (-0.02, 0.41)	ASES, change from BL – 26 months, mean (SD †)<br>Hand exercise: 2.96 (18.6)<br>Control: 0.22 (17.2)		L	L	H/UC	L
	Lamb (2015) [RCT][138]	Hand Exercise vs control, mean change BL-12 weeks<br>SMD 0.19 (0.02, 0.37)	ASES, change from BL – 12 months, mean (SD †)<br>Hand exercise: 5.19 (21.9)<br>Control: 1.11 (20.2)		L	L	H/UC	L
	Manning (2014) [RCT][139]	Exercise vs control, change from BL-12 weeks<br>Pain SMD 0.36 (-0.02, 0.74)<br>Function SMD 0.31 (-0.07, 0.69)<br>Symptoms: SMD 0.32 (-0.06, 0.70)	ASES, change BL-12 weeks, mean (SD †)<br>*Pain*<br>Exercise: 4.8 (29.2)<br>Control: -5.7 (28.6)<br>*Function*<br>Exercise: 2.6 (23.9)<br>Control: -4.7 (23.5)<br>*Symptoms*<br>Exercise: 4.6 (28.3)<br>Control: -4.7 (29.4)		L	L	H/UC	L

† SD calculated from 95% confidence interval

Alloc. Conc. = allocation concealment, AMSTAR2 = Assessing the Methodological Quality of Systematic Reviews, ASES = Arthritis Self-Efficacy Scale, BL = baseline, Blind. Asses. = Blinded assessors, Blind. Part. = blinded participants, CI = confidence interval, H/UC = high / unclear risk of bias, L = low risk of bias, RA = rheumatoid arthritis, Rand. Seq. = random sequence generation, RCT = randomised controlled trial, RoM = range of motion, SD = standard deviation, SMD = standardised mean difference

Table – Muscle strengthening exercise (RA), results and quality assessment

Outcome (outcome measure)	Study (date) [study type]	Standardised result, SMD (95% CI) unless otherwise stated	Natural result	AMSTAR2 quality	Rand. Seq.	Alloc. Conc.	Blind. Part.	Blind. Asses.
Morning stiffness	Manning (2014) [RCT][139]	Exercise vs control, change from BL-12 weeks<br>SMD -0.24 (-0.62, 0.14)	Morning stiffness (mins), change BL-12 weeks, mean (SD †)<br>Exercise: -115.9 (492.5)<br>Control: 4.1 (510.9)		L	L	H/UC	L
	Komatireddy (1997) [RCT][147]	Exercise vs control at 12 weeks<br>SMD -0.17 (-0.73, 0.39)	Morning stiffness (hours), BL / 12 weeks, mean (SD)<br>Exercise: 1.0 (0.8) / 0.9 (0.9)<br>Control: 1.1 (1.4) / 1.1 (1.4)		H/UC	H/UC	H/UC	L
	Bespoke meta-analysis including[139;147]	Exercise vs control<br>SMD -0.22 (-0.53, 0.10), I^2 0%						
CRP	Lo (2017) [RCT][135]	Exercise vs control at 4 weeks<br>SMD -0.42 (-1.75, 0.92)	CRP, BL / 4 weeks, mean (SD) ‡<br>Exercise: 5.9 (4.9) / 2.7 (2.7)<br>Control: 3.5 (1.6) / 5.1 (8.2)		H/UC	H/UC	H/UC	H/UC
	Lamb (2015) [RCT][138]	Hand Exercise vs control, mean change BL-12 weeks<br>SMD -0.02 (-0.19, 0.16)	CRP [log], change from BL – 12 months, mean (SD †)<br>Hand Exercise: -0.14 (1.2)<br>Control: -0.12 (1.3)		L	L	H/UC	L
	Bespoke meta-analysis including [135;138]	Exercise vs control<br>SMD -0.02 (-0.20, 0.15), I^2 0%						
ESR	Siegel (2017) [SR][69]		1 MA[152] reported improvement in ESR (MD -5.17 [-8.77, -1.58])	Moderate				
	Lo (2017) [RCT][135]	Exercise vs control at 4 weeks<br>SMD -0.46 (-1.80, 0.88)	CRP, BL / 4 weeks, mean (SD) ‡<br>Exercise: 21 (14) / 16.8 (11.8)<br>Control: 19 (11.5) / 23.5 (17.6)		H/UC	H/UC	H/UC	H/UC
	Lamb (2015) [RCT][138]	Hand Exercise vs control, mean change BL-12 weeks<br>SMD 0.06 (-0.12, 0.24)	ESR [log], change from BL – 12 months, mean (SD †)<br>Hand Exercise: -0.04 (1.1)<br>Control: -0.10 (1.0)		L	L	H/UC	L
	Buljina (2001) [RCT][144]	Hand exercise vs control at 4 weeks<br>SMD -0.49 (-0.89, -0.10)	ESR, BL / 4 weeks, mean (SD)<br>Hand exercises: 39.6 (15.0) / 34.2 (12.2)<br>Control: 40.3 (14.8) / 41.0 (15.2)		L	H/UC	H/UC	H/UC
	Bespoke meta-analysis including [135;138;144]	Exercise vs control<br>SMD -0.21 (-0.67, 0.25), I^2 69.3%						
	Marcora (2005) [NRT][150]	Exercise vs control at 12 weeks<br>SMD -0.29 (-1.18, 0.59)	ESR, BL / 12 weeks, mean (SD)<br>Exercise: 18.8 (16.6) / 16.7 (8.9)<br>Control: 22.5 (17.6) / 20.9 (18.1)					

† SD calculated from 95% confidence interval, ‡ Mean (SD) calculated by reviewers from data in the paper

Alloc. Conc. = allocation concealment, AMSTAR2 = Assessing the Methodological Quality of Systematic Reviews, BL = baseline, Blind. Asses. = Blinded assessors, Blind. Part. = blinded participants, CI = confidence interval, CRP = C-reactive protein, ESR = erythrocyte sedimentation rate, H/UC = high / unclear risk of bias, L = low risk of bias, MA = meta-analysis, NRT = non-randomised trial, RA = rheumatoid arthritis, Rand. Seq. = random sequence generation, RCT = randomised controlled trial, SD = standard deviation, SMD = standardised mean difference

Table – Muscle strengthening exercise (RA), results and quality assessment

Outcome (outcome measure)	Study (date) [study type]	Standardised result, SMD (95% CI) unless otherwise stated	Natural result	AMSTAR2 quality	Rand. Seq.	Alloc. Conc.	Blind. Part.	Blind. Asses.
Grip strength	Williams (2018) [MA][132]	Exercise vs control<br>SMD 0.46 (0.13, 0.80)		High				
	Daien (2017) [SR][133]		Hand grip<br>2 studies reported benefits of exercise interventions on muscle strength	Low				
	Bergstra (2014) [SR][134]		6/7 studies reported improvements in grip strength	Low				
	Lourenzi (2017) [RCT][137]	Exercise vs control at 12 months<br>Right SMD 0.35 (-0.16, 0.86)<br>Left SMD 0.15 (-0.36, 0.66)	Grip strength, 6 months / 12 months, mean (SD)<br>*Right hand*<br>Exercises: 17.39 (10.46) / 18.13 (8.58)<br>Control: 15.23 (10.05) / 14.69 (10.82)<br>*Left hand*<br>Exercises: 16.13 (9.42) / 16.59 (8.15)<br>Control: 15.33 (7.81) / 15.39 (7.52)		L	L	H/UC	L
	Lamb (2015) [RCT][138]	Hand Exercise vs control, mean change BL-12 weeks<br>SMD 0.13 (-0.04, 0.31)	Grip strength, change from BL – 12 months, mean (SD †)<br>Hand exercise: 15.77 (45.3)<br>Control: 9.57 (46.9)		L	L	H/UC	L
	Manning (2014) [RCT][139]	Exercise vs control, change from BL-12 weeks<br>SMD 0.14 (-0.24, 0.52)	Grip strength, change BL-12 weeks, mean (SD †)<br>Exercise: 16.8 (91.8)<br>Control: 3.7 (92.8)		L	L	H/UC	L
	Dogu (2013) [RCT][140]	Isotonic vs isometric exercise, change from BL-6 weeks<br>SMD -0.42 (-0.99, 0.16)	Dominant grip strength, Change BL-6 weeks, mean (SD)<br>Isotonic: 0.56 (2.62)<br>Isometric: 2.04 (4.28)		L	H/UC	H/UC	L
	O'Brien (2006) [RCT][143]	Muscle strengthening hand exercise vs control, change from BL-6 months<br>SMD 0.37 (-0.24, 0.97)<br>Stretching hand exercise vs control, change from BL-6 months<br>SMD 0.17 (-0.41, 0.75)	Grip strength, change BL-6 months, mean (SD)<br>Muscle strengthening hand exercise: 9.70 (11.50)<br>Stretching hand exercise: 6.70 (17.35)<br>Control: 3.40 (21.32)		L	L	H/UC	L

† SD calculated from 95% confidence interval

Alloc. Conc. = allocation concealment, AMSTAR2 = Assessing the Methodological Quality of Systematic Reviews, BL = baseline, Blind. Asses. = Blinded assessors, Blind. Part. = blinded participants, CI = confidence interval, H/UC = high / unclear risk of bias, L = low risk of bias, RA = rheumatoid arthritis, Rand. Seq. = random sequence generation, RCT = randomised controlled trial, SD = standard deviation, SMD = standardised mean difference

Table – Muscle strengthening exercise (RA), results and quality assessment

Outcome (outcome measure)	Study (date) [study type]	Standardised result, SMD (95% CI) unless otherwise stated	Natural result	AMSTAR2 quality	Rand. Seq.	Alloc. Conc.	Blind. Part.	Blind. Asses.
Grip strength	Buljina (2001) [RCT][144]	Hand exercise vs control at 4 weeks<br>Right hand: SMD 0.49 (0.09, 0.89)<br>Left hand: SMD 0.50 (0.10, 0.90)	Grip strength, BL / 4 weeks, mean (SD)<br>*Right hand*<br>Hand exercises: 15.0 (8.7) / 19.8 (9.4)<br>Control: 15.6 (8.7) / 15.3 (8.9)<br>*Left hand*<br>Hand exercises: 14.4 (8.2) / 18.5 (8.8)<br>Control: 14.3 (7.9) / 14.3 (8.0)		L	H/UC	H/UC	H/UC
	Komatireddy (1997) [RCT][147]	Exercise vs control at 12 weeks<br>SMD -0.12 (-0.68, 0.44)	Grip strength, BL / 12 weeks, mean (SD)<br>Exercise: 35.9 (20.0) / 44.2 (22.0)<br>Control: 43.7 (26.7) / 46.7 (20.8)		H/UC	H/UC	H/UC	L
	Hoenig (1993) [RCT][148]	Hand muscle strengthening exercise vs control at 3 months<br>Right: SMD 0.02 (-0.86, 0.90)<br>Left: SMD 0.11 (-0.77, 0.99)<br>Hand RoM + muscle strengthening vs control at 3 months<br>Right SMD 0.26 (-0.60, 1.12)<br>Left SMD 0.23 (-0.63, 1.09)	Grip strength, BL / 3 months, mean (SD)<br>*Right hand*<br>RoM: 93.4 (58.0) / 85.5 (43.8)<br>Muscle strengthening: 69.3 (43.3) / 82.1 (43.0)<br>RoM + muscle strengthening: 84.2 (61.5) / 97.6 (68.4)<br>Control: 68.2 (36.7) / 81.1 (60.1)<br>*Left hand*<br>RoM: 70.4 (44.0) / 84.0 (47.5)<br>muscle strengthening: 62.0 (32.8) / 87.4 (44.6)<br>RoM + muscle strengthening: 83.2 (62.1) / 96.8 (71.6)<br>Control: 83.0 (64.9) / 81.1 (64.7)		L	H/UC	H/UC	L
	Dellhag (1992) [RCT][149]		Grip strength, BL / 4 weeks, mean<br>Exercise: 90.7 / 109.7<br>Control: 82.6 / 85.4		H/UC	H/UC	H/UC	H/UC
	Bespoke meta-analysis including[137-139;143;144;148]	Exercise vs control<br>SMD 0.18 (0.05, 0.32)						
	Marcora (2005) [NRT][150]	Exercise vs control at 12 weeks<br>SMD 0.16 (-0.72, 1.04)	Grip strength, BL / 12 weeks, mean (SD)<br>Exercise: 187 (108) / 224 (115)<br>Control: 223 (133) / 204 (135)					

Alloc. Conc. = allocation concealment, AMSTAR2 = Assessing the Methodological Quality of Systematic Reviews, BL = baseline, Blind. Asses. = Blinded assessors, Blind. Part. = blinded participants, CI = confidence interval, H/UC = high / unclear risk of bias, L = low risk of bias, NRT = non-randomised trial, RA = rheumatoid arthritis, Rand. Seq. = random sequence generation, RCT = randomised controlled trial, RoM = range of motion, SD = standard deviation, SMD = standardised mean difference

Table – Muscle strengthening exercise (RA), results and quality assessment

Outcome (outcome measure)	Study (date) [study type]	Standardised result, SMD (95% CI) unless otherwise stated	Natural result	AMSTAR2 quality	Rand. Seq.	Alloc. Conc.	Blind. Part.	Blind. Asses.
Walk-test	Lemmey (2012) [RCT][122]	Exercise vs control at 3 years<br>SMD -0.20 (-1.13, 0.73)	50ft walk, BL / 3 years, mean (SD)<br>Exercise: 9.68 (2.77) / 8.50 (1.77)<br>Control: 8.80 (2.96) / 9.06 (3.51)		L	H/UC	H/UC	H/UC
	Lemmey (2009) [RCT][123]	Exercise vs control at 24 weeks<br>SMD -0.65 (-1.41, 0.12)	50ft walk, BL / 24 weeks, mean (SD)<br>Exercise: 9.33 (2.40) / 7.77 (1.40)<br>Control: 10.03 (3.78) / 9.89 (4.28)		L	H/UC	H/UC	H/UC
	Komatireddy (1997) [RCT][147]	Exercise vs control at 12 weeks<br>SMD 0.02 (-0.54, 0.58)	50m walk time, BL / 12 weeks, mean (SD)<br>Exercise: 12.4 (3.2) / 12.4 (3.2)<br>Control: 12.0 (4.0) / 12.3 (8.3)		H/UC	H/UC	H/UC	L

Alloc. Conc. = allocation concealment, AMSTAR2 = Assessing the Methodological Quality of Systematic Reviews, BL = baseline, Blind. Asses. = Blinded assessors, Blind. Part. = blinded participants, CI = confidence interval, H/UC = high / unclear risk of bias, L = low risk of bias, RA = rheumatoid arthritis, Rand. Seq. = random sequence generation, RCT = randomised controlled trial, RoM = range of motion, SD = standard deviation, SMD = standardised mean difference

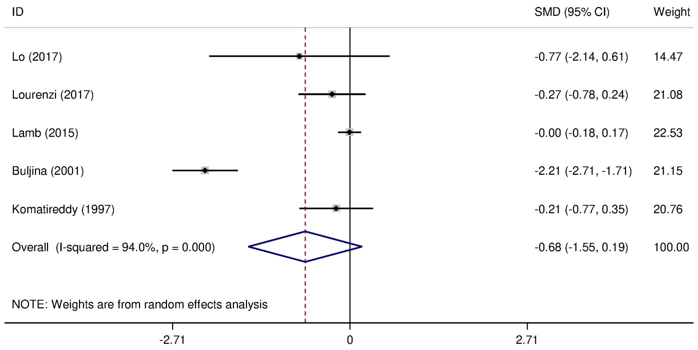

Figure – The effect of muscle strengthening exercise on pain (RA)

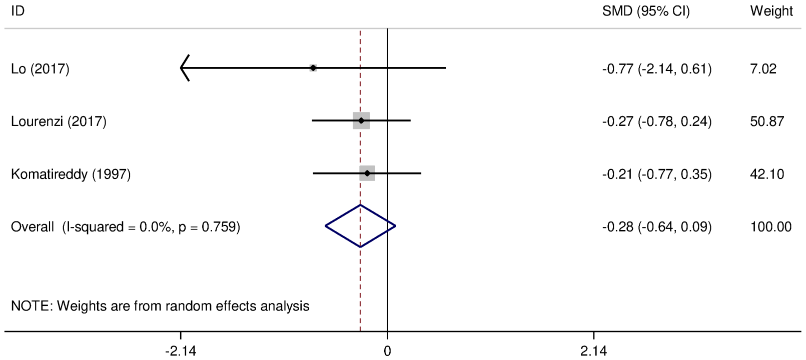

Figure – The effect of muscle strengthening exercise on pain (RA), only including non-hand muscle strengthening exercises

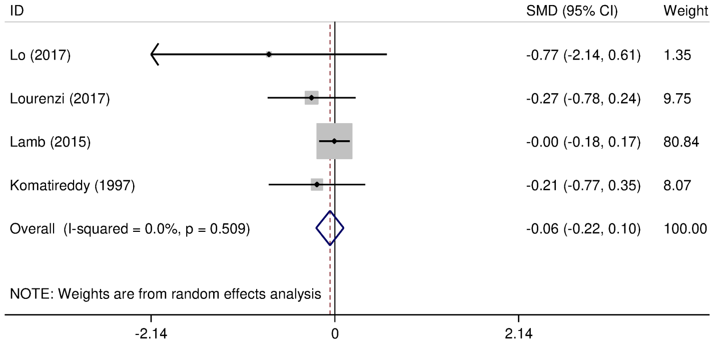

Figure – The effect of muscle strengthening exercise on pain (RA), excluding 1 outlier

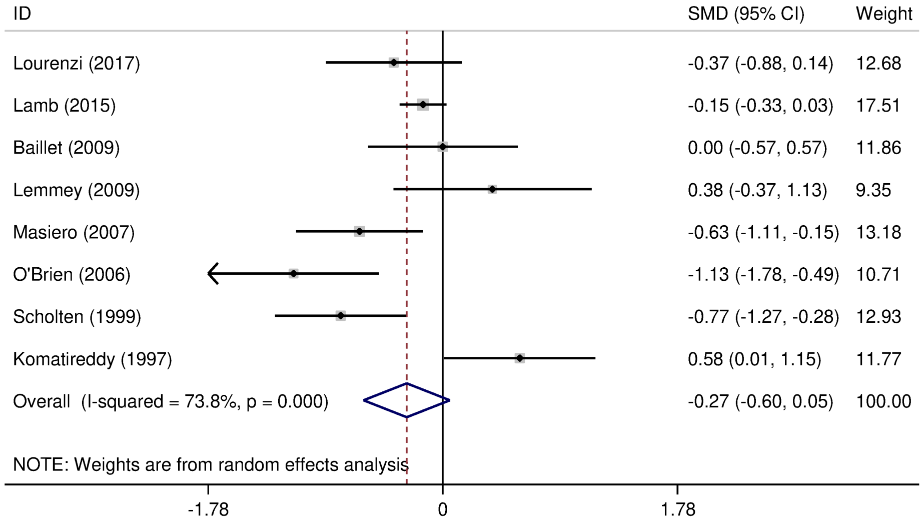

Figure – The effect of muscle strengthening exercise on function (RA)

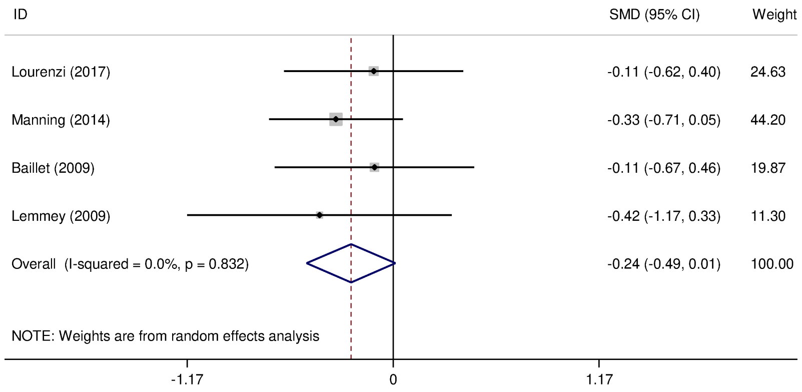

Figure – The effect of muscle strengthening exercise on disease activity (RA)

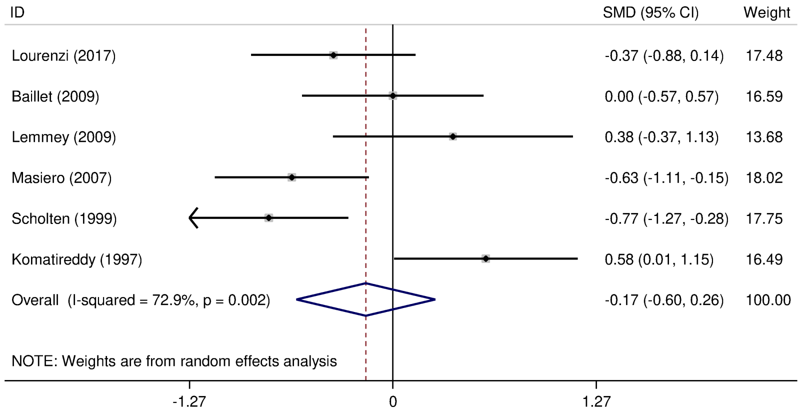

Figure – The effect of muscle strengthening exercise on function (RA), non-hand exercises only

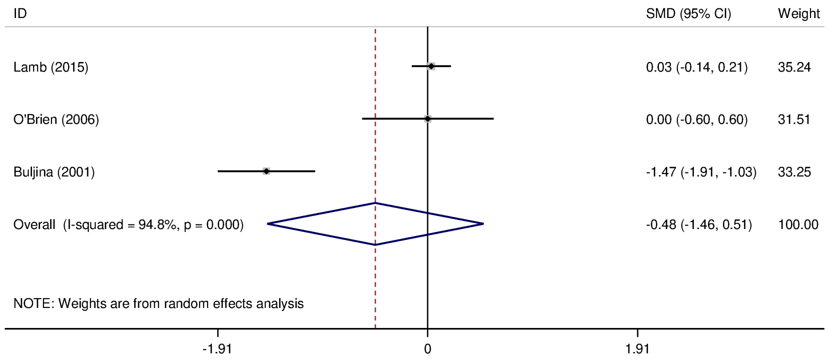

Figure – The effect of muscle strengthening exercise on tender joint count (RA)

BMJ Publishing Group Limited (BMJ) disclaims all liability and responsibility arising from any reliance placed on this supplemental material which has been supplied by the author(s)

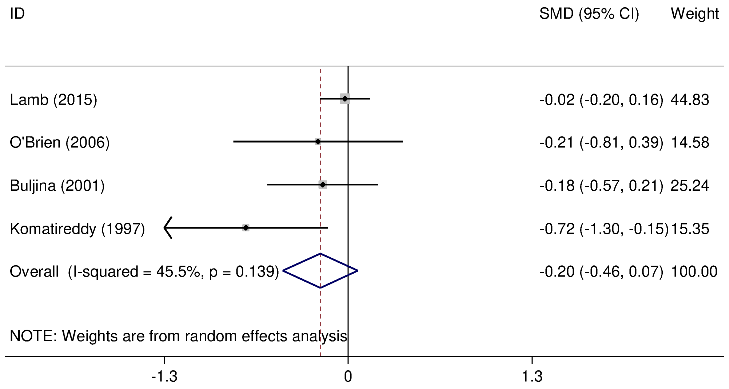

Figure – The effect of muscle strengthening exercise on swollen joint count (RA)

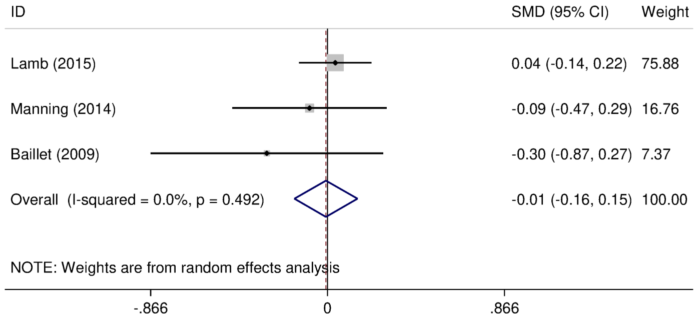

Figure – The effect of muscle strengthening exercise on quality of life (RA)

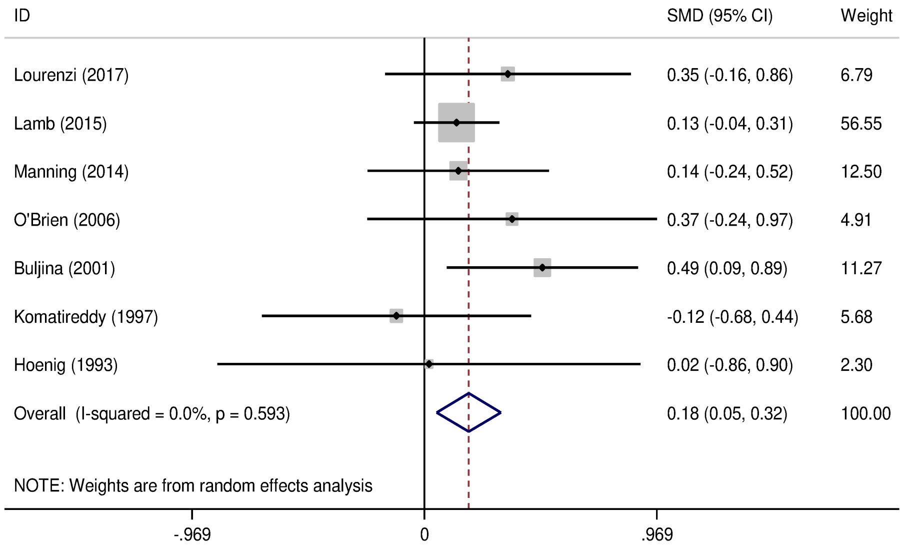

Figure – The effect of muscle strengthening exercise on grip strength (RA)

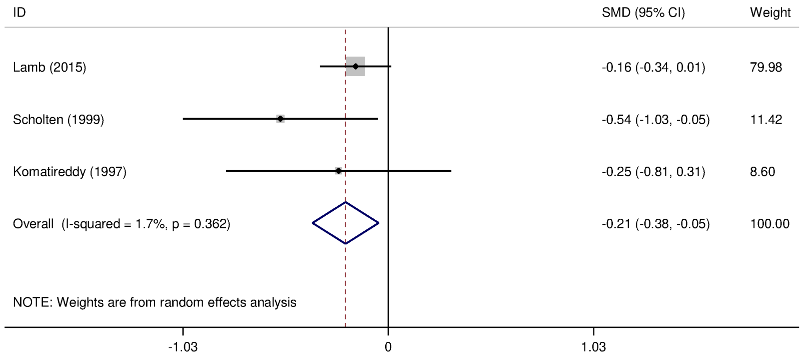

Figure – The effect of muscle strengthening exercise on depression (RA)

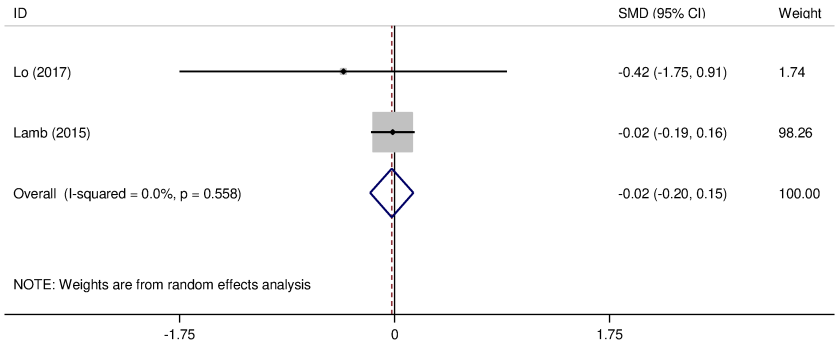

Figure – The effect of muscle strengthening exercise on CRP (RA)

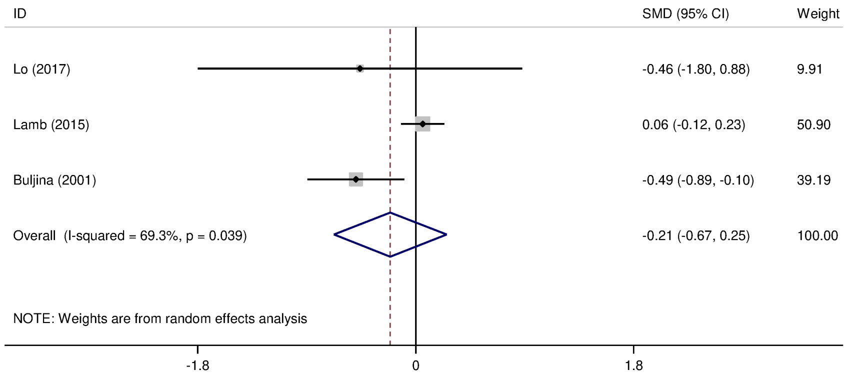

Figure – The effect of muscle strengthening exercise on ESR (RA)

Supplementary table 45 – Description of reviews of studies of Tai Chi in RA

Table – Tai-Chi, description of reviews

Authors (date)	Review type	Study type included	Exposure detail	Number of studies included	Funders
Siegel (2017)[69]	SR	Reviews, RCTs, observational	Aerobic exercise	1	**Not reported**

RA = rheumatoid arthritis, RCT = randomised controlled trial, SR = systematic review

Supplementary table 46 – Description of original studies of Tai Chi in RA

Table – Tai-Chi, description of included studies

Author (date) [country]	Study design	Inclusion criteria	Exposure detail	N	Age years, mean (SD)	N (%) female	Funders
Wang (2008) [USA][153]	RCT	RA functional class I-II, aged ≥18 years<br>Exclusions: prior experience with Tai-Chi or similar complementary therapy, cardiovascular disease or other severe disease, mini-mental state <24, pregnant or breastfeeding, non-English speaking, participated in another trial in last 30 days	1) 2x 60min sessions of Tai-Chi – 10 mins warm up, 30 mins Tai-Chi, 10 mins breathing, 10 mins relaxation. Also instructed to do 20 mins of Tai-Chi at home per day<br>p) Attention control – 2x 60 min sessions per week learning about RA (e.g. disease aspects, diet, therapies, mental health)	1) 10<br>p) 10	1) 48 (10)<br>p) 51 (17)	1) 80%<br>p) 70%	**Not reported**
Shin (2015) [South Korea][154]	NRT	1987 ACR RA criteria, aged >50 years, sedentary lifestyle, stable DMARDs/steroids ≥3 months<br>Exclusions: inability to bear weight on lower extremities, recent or ongoing disease flare, unstable heart condition, serious comorbidity such as terminal malignancy	1) "Twelve Movement Tai-Chi for arthritis" – small to large degrees of motion, knee flexion, straight and extended head and trunk, combined rotation of head, trunk and extremities, symmetrical diagonal arm and lef movements<br>p) Advice about regular, appropriate exercise	1) 29<br>p) 14	1) 64.0 (5.4)<br>p) 62.7 (5.9)	1) 29 (100)<br>p) 14 (100)	**University** (Hanyang University)
Lee (2012) [UK][155]	Single arm int. §	Rheumatologist diagnosis of RA, no need for walking aids indoors, 50ft walk test without shortness of breath, no pain on external ear, not currently participating in exercise program more than twice a week	Tai-Chi	21	60 (13.4)	7 (100)	**Charity** (National Research Foundation of Korea)
Uhlig (2010) [Norway][156]	Single arm int.	1987 ACR RA criteria, aged 18-70 years, stable medical treatment, no earlier experience of Tai-Chi<br>Exclusions: lack of ability to bear weight on lower extremities, recent or ongoing disease flare, unstable heart condition, participation in other physical exercise more than twice per week	"Twelve Movement Tai-Chi for arthritis" – group exercise 2x per week for 60 mins each	15	Median (range)<br>57 (33, 70)	11 (73.3)	**Hospital** (Diakonhjemmet Hospital)

§ Study also included a Tai-Chi + auricular acupressure arm – the study reports results of these arm combined

ACR = American College of Rheumatology, DMARDs = Disease modifying anti-rheumatic drugs, N = number, NRT = non-randomised trial, RA = rheumatoid arthritis, RCT = randomised controlled trial, SD = standard deviation,

Supplementary table 47 – Results from reviews and interventional studies of Tai Chi in RA

Table – Tai-Chi, results and quality assessment

Outcome (outcome measure)	Study (date) [study type]	Standardised result, SMD (95% CI) unless otherwise stated	Natural result	AMSTAR2 quality	Rand. Seq.	Alloc. Conc.	Blind. Part.	Blind. Asses.
Pain	Wang (2008) [RCT][153]	Tai-Chi vs control<br>SMD -0.32 (-1.20, 0.57)	Pain VAS, BL / 12 weeks, mean (SD)<br>Exercise: 3.2 (2.2) / 2.3 (2.0)<br>Control: 1.4 (1.3) / 3.0 (2.4)		L	L	H/UC	H/UC
	Lee (2012) [Single arm int.][155]		Pain VAS, BL / 12 weeks, median<br>5.0 / 4.0; p=0.19					
	Uhlig (2010) [Single arm int.][156]		Muscle pain, BL / 12 weeks, median (range)<br>30 (1, 60) / 23 (2, 61); p=0.88					
Function	Siegel (2017) [SR][69]		One SR[157] of 4 studies concluded that Tai-Chi did not produce clinically important or significant change in function	Moderate				
	Wang (2008) [RCT][153]	Tai-Chi vs control<br>SMD -0.25 (-1.13, 0.63)	HAQ, BL / 12 weeks, mean (SD)<br>Exercise: 0.9 (0.7) / 0.4 (0.4)<br>Control: 0.4 (0.3) / 0.5 (0.4)		L	L	H/UC	H/UC
	Shin (2015) [NRT][139]	Tai-Chi vs control<br>SMD -0.49 (-1.14, 0.16)	HAQ, change Bl-3 months, mean (SD)<br>Exercise: -0.13 (0.29)<br>Control: 0.00 (0.20); p=0.274					
	Uhlig (2010) [Single arm int.][156]		HAQ, BL / 12 weeks, median (range)<br>0.5 (0.0, 1.5) / 0.5 (0.0, 1.5); p=0.34					
Disease activity	Siegel (2017) [SR][69]		One SR[157] of 4 studies concluded that Tai-Chi did not produce clinically important or significant change in disease activity	Moderate				
	Shin (2015) [NRT][154]	Tai-Chi vs control<br>SMD -0.36 (-1.01, 0.28)	DAS28-ESR, change Bl-3 months, mean (SD)<br>Exercise: -0.4 (1.1)<br>Control: -0.0 (1.1)					
	Uhlig (2010) [Single arm int.][156]		DAS28, BL / 12 weeks, median (range)<br>4.7 (2.2, 6.7) / 4.7 (0.8, 6.5); p=0.24					

Alloc. Conc. = allocation concealment, AMSTAR2 = Assessing the Methodological Quality of Systematic Reviews, Blind. Asses. = Blinded assessors, Blind. Part. = blinded participants, CI = confidence interval, DAS28 = Disease Activity Score, ESR = erythrocyte sedimentation rate, H/UC = high / unclear risk of bias, HAQ = Health Assessment Questionnaire, L = low risk of bias, NRT = non-randomised trial, RA = rheumatoid arthritis, Rand. Seq. = random sequence generation, RCT = randomised controlled trial, SD = standard deviation, SMD = standardised mean difference, SR = systematic review, VAS = visual analogue scale

Table – Tai-Chi, results and quality assessment

Outcome (outcome measure)	Study (date) [study type]	Standardised result, SMD (95% CI) unless otherwise stated	Natural result	AMSTAR2 quality	Rand. Seq.	Alloc. Conc.	Blind. Part.	Blind. Asses.
Tender joints	Siegel (2017) [SR][69]		One SR[157] of 4 studies concluded that Tai-Chi did not produce clinically important or significant change in tender joint count	Moderate				
	Wang (2008) [RCT][153]	Tai-Chi vs control<br>SMD 0.06 (-0.82, 0.93)	Tender joint count, BL / 12 weeks, mean (SD)<br>Exercise: 17.0 (10.2) / 11.7 (8.1)<br>Control: 10.7 (8.6) / 11.1 (12.3)		L	L	H/UC	H/UC
	Shin (2015) [NRT][154]	Tai-Chi vs control<br>SMD -0.64 (-1.29, 0.02)	Tender joint count (69), change Bl-3 months, mean (SD)<br>Exercise: -2.5 (4.5)<br>Control: 0.1 (3.0); p=0.107					
	Lee (2012) [Single arm int.][155]		Tender joint count, BL / 12 weeks, median<br>11.0 / 5.0; p=0.002					
	Uhlig (2010) [Single arm int.][156]		Tender joint count, BL / 12 weeks, median (range)<br>8 (1, 29) / 9 (0, 20); p=0.40					
Swollen joints	Siegel (2017) [SR][69]		One SR[157] of 4 studies concluded that Tai-Chi did not produce clinically important or significant change in swollen joint count	Moderate				
	Wang (2008) [RCT][153]	Tai-Chi vs control<br>SMD 0.38 (-0.51, 1.27)	Swollen joint count, BL / 12 weeks, mean (SD)<br>Exercise: 13.5 (10.6) / 12.3 (10.6)<br>Control: 6.7 (8.0) / 8.4 (9.9)		L	L	H/UC	H/UC
	Shin (2015) [NRT][154]	Tai-Chi vs control<br>SMD -0.21 (-0.85, 0.43)	Swollen joint count (69), change Bl-3 months, mean (SD)<br>Exercise: -0.6 (3.3)<br>Control: 0.0 (1.7); p=0.834					
	Lee (2012) [Single arm int.][155]		Swollen joint count, BL / 12 weeks, median<br>9.0 / 5.0; p=0.002					
	Uhlig (2010) [Single arm int.][156]		Swollen joint count, BL / 12 weeks, median (range)<br>11 (2, 20) / 5 (0, 14); p=0.02					

Alloc. Conc. = allocation concealment, AMSTAR2 = Assessing the Methodological Quality of Systematic Reviews, BL = baseline, Blind. Asses. = Blinded assessors, Blind. Part. = blinded participants, CI = confidence interval, H/UC = high / unclear risk of bias, L = low risk of bias, NRT = non-randomised trial, QoL = quality of life, RA = rheumatoid arthritis, Rand. Seq. = random sequence generation, RCT = randomised controlled trial, SD = standard deviation, SMD = standardised mean difference, SR = systematic review

Table – Tai-Chi, results and quality assessment

Outcome (outcome measure)	Study (date) [study type]	Standardised result, SMD (95% CI) unless otherwise stated	Natural result	AMSTAR2 quality	Rand. Seq.	Alloc. Conc.	Blind. Part.	Blind. Asses.
Fatigue	Wang (2008) [RCT][153]	Tai-Chi vs control<br>SMD -0.10 (-0.97, 0.78)	Fatigue VAS, BL / 12 weeks, mean (SD)<br>Exercise: 4.5 (2.5) / 2.9 (2.3)<br>Control: 3.6 (3.0) / 3.1 (1.9)		L	L	H/UC	H/UC
	Lee (2012) [Single arm int.][155]		Fatigue VAS, BL / 12 weeks, median<br>5.0 / 3.0; p=0.004					
	Uhlig (2010) [Single arm int.][156]		Fatigue, BL / 12 weeks, median (range)<br>27 (5, 89) / 25 (6, 74); p=0.70					
QoL	Wang (2008) [RCT][153]	Tai-Chi vs control<br>SMD 0.30 (-0.59, 1.18)	EQ5D, BL / 12 weeks, mean (SD)<br>Exercise: 72.4 (19.2) / 78.8 (13.8)<br>Control: 84.0 (7.9) / 74.1 (17.7)		L	L	H/UC	H/UC
Patient global	Siegel (2017) [SR][69]		One SR[157] of 4 studies concluded that Tai-Chi did not produce clinically important or significant change in patient global assessment	Moderate				
	Wang (2008) [RCT][153]	Tai-Chi vs control<br>SMD -0.30 (-1.18, 0.59)	Patient global VAS, BL / 12 weeks, mean (SD)<br>Exercise: 2.9 (2.4) / 2.4 (2.6)<br>Control: 2.4 (1.9) / 3.2 (2.8)		L	L	H/UC	H/UC
	Uhlig (2010) [Single arm int.][156]		Patient global, BL / 12 weeks, median (range)<br>31 (1, 51) / 43 (6, 61); p=0.39					
Depression	Wang (2008) [RCT][153]	Tai-Chi vs control<br>SMD -0.47 (-1.36, 0.42)	CES-D, BL / 12 weeks, mean (SD)<br>Exercise: 16.6 (3.5) / 14.3 (1.9)<br>Control: 13.0 (3.6) / 15.8 (4.1)		L	L	H/UC	H/UC
	Lee (2012) [Single arm int.][155]		AIMS2 (affect), BL / 12 weeks, median<br>2.7 / 2.1; p=0.001					
Self-efficacy	Lee (2012) [Single arm int.][155]		ASES, BL / 12 weeks, median<br>function: 6.4 / 7.2; p=0.106<br>other symptoms: 6.3 / 6.8; p=0.019<br>pain: 5.8 / 7.2 ; p=0.02					
	Uhlig (2010) [Single arm int.][156]		Muscle pain, BL / 12 weeks, median (range)<br>pain: not measured / 62 (18, 86)<br>function: 89 (54, 100) / 90 (61, 100); p=0.22<br>symptoms: 75 (35, 90) / 78 (57, 97); p=0.13					

AIMS2 = Arthritis Impact Measurement Scales, Alloc. Conc. = allocation concealment, AMSTAR2 = Assessing the Methodological Quality of Systematic Reviews, ASES = Arthritis Self-efficacy scale, BL = baseline, Blind. Asses. = Blinded assessors, Blind. Part. = blinded participants, CI = confidence interval, H/UC = high / unclear risk of bias, L = low risk of bias, QoL = quality of life, RA = rheumatoid arthritis, Rand. Seq. = random sequence generation, RCT = randomised controlled trial, SD = standard deviation, SMD = standardised mean difference, SR = systematic review

Table – Tai-Chi, results and quality assessment

Outcome (outcome measure)	Study (date) [study type]	Standardised result, SMD (95% CI) unless otherwise stated	Natural result	AMSTAR2 quality	Rand. Seq.	Alloc. Conc.	Blind. Part.	Blind. Asses.
CRP	Wang (2008) [RCT][153]	Tai-Chi vs control<br>SMD 0.71 (-0.20, 1.62)	CRP, BL / 12 weeks, mean (SD)<br>Exercise: 1.4 (0.8) / 1.3 (1.3)<br>Control: 0.5 (0.4) / 0.6 (0.5)		L	L	H/UC	H/UC
	Shin (2015) [NRT][154]	Tai-Chi vs control<br>SMD 0.14 (-0.50, 0.77)	CRP, change Bl-3 months, mean (SD)<br>Exercise: 0.2 (0.8)<br>Control: 0.1 (0.6); p=0.399					
ESR	Wang (2008) [RCT][153]	Tai-Chi vs control<br>SMD 0.20 (-0.68, 1.08)	ESR, BL / 12 weeks, mean (SD)<br>Exercise: 35.3 (28.4) / 35.1 (27.6)<br>Control: 26.8 (14.1) / 30.1 (21.3)		L	L	H/UC	H/UC
	Shin (2015) [NRT][154]	Tai-Chi vs control<br>SMD 0.19 (-0.45, 0.83)	ESR, change Bl-3 months, mean (SD)<br>Exercise: 2.0 (18.0)<br>Control: -1.1 (12.4); p=0.569					
Grip strength	Wang (2008) [RCT][153]	Tai-Chi vs control<br>SMD -0.51 (-1.40. 0.39)	Grip strength, BL / 12 weeks, mean (SD)<br>Exercise: 16.7 (9.3) / 17.7 (9.9)<br>Control: 23.3 (9.3) / 22.5 (9.0)		L	L	H/UC	H/UC
	Lee (2012) [Single arm int.][155]		Grip strength, BL / 12 weeks, median<br>Right hand: 20.0 / 40.0; p=0.001<br>Left hand: 28.0 / 34.0; p=0.001					
Walk-test	Wang (2008) [RCT][153]	Tai-Chi vs control<br>SMD -0.08 (-0.96, 0.80)	50ft walk, BL / 12 weeks, mean (SD)<br>Exercise: 10.9 (3.2) / 9.6 (3.1)<br>Control: 11.3 (2.5) / 9.8 (1.6)		L	L	H/UC	H/UC
	Lee (2012) [Single arm int.][155]		50ft Walk-test, BL / 12 weeks, median<br>16.2 / 13.5; p=0.001					

Alloc. Conc. = allocation concealment, AMSTAR2 = Assessing the Methodological Quality of Systematic Reviews, BL = baseline, Blind. Asses. = Blinded assessors, Blind. Part. = blinded participants, CI = confidence interval, CRP = C-reactive protein, ESR = erythrocyte sedimentation rate, H/UC = high / unclear risk of bias, L = low risk of bias, NRT = non-randomised trial, RA = rheumatoid arthritis, Rand. Seq. = random sequence generation, RCT = randomised controlled trial, SD = standard deviation, SMD = standardised mean difference

Table – Tai-Chi (RA), SF36 results at final follow-up, mean (SD)

Author (date)	PCS	MCS	GH	PF	RP	RE	SF	BP	V	MH
Wang (2008) [Tai-Chi][153]	42.8 (8.2)	56.9 (5.4)	68.3 (18.3)	75.0 (16.3)	67.5 (39.2)	100.0 (0.0)	85.0 (16.5)	63.4 (16.5)	64.0 (15.4)	82.0 (14.9)
Wang (2008) [Control][153]	43.2 (9.5)	54.2 (9.2)	69.0 (17.0)	76.3 (17.5)	55.0 (38.7)	86.7 (23.3)	82.5 (17.9)	66.1 (20.1)	62.0 (16.5)	79.6 (16.2)
Uhlig (2010) [Tai-Chi][156] §			47 (35, 97)	75 (25, 95)	25 (0, 100)	67 (0, 100)	62 (12, 100)	42 (22, 100)	50 (25, 70)	76 (56, 92)

§ Results presented as median (range)

BL = baseline, BP = bodily pain, FU = follow-up, GH = general health, IQR = interquartile range, MCS = mental component score, MH = mental health, PCS = physical component score, PF = physical function, RE = role emotional, RP = role physical, SD = standard deviation, SF = social functioning, V = vitality

Supplementary table 48 – Description of reviews of studies of yoga in RA

Table – Yoga (RA), description of reviews

Authors (date)	Review type	Study type included	Exposure detail	Number of studies included	Funders
Wang (2018)[64]	MA	RCTs	Yoga	13	**Government** (National Natural Science Foundation of China)
Cramer (2013)[66]	SR	RCTs	Yoga	2	**Charity** (the Rut- and Klaus-Bahlsen-Foundation)

MA = meta-analysis, RA = rheumatoid arthritis, RCT = randomised controlled trial, SR = systematic review

Supplementary table 49 – Description of original studies of yoga in RA

Table –Yoga (RA), description of included studies

Author (date) [country]	Study design	Inclusion criteria	Exposure detail	N	Age years, mean (SD)	N (%) female	Funders
Ward (2018) [New Zealand][158]	RCT	2010 ACR/EULAR RA criteria, ≥18 years, pain in previous month ≥3 out of 10, average self-reported sleep disturbance over previous month greater than 30 min per night, ability to self-mobilize up and down from chair<br>Exclusion: current regular yoga practice (>1 per week, major surgery within past 6 months, planned surgery in following 6 months, intra-articular steroid injections within previous 4 weeks, serious co-morbidities, inability to commit to 13 week study	1) 8 week program of group and home yoga – group practice consisted of once-weekly 75 min yoga classes; home practice based on 20-min guided relaxation<br>p) Usual care	1) 13<br>p) 13	1) 50 (12)<br>p) 59 (8)	1) 13 (100)<br>p) 12 (92)	**Charity** (Arthritis New Zealand)
Evans (2013) [USA][159]	RCT	1987 ACR RA criteria, symptom duration >6 months, aged 16-35 years, using DMARDs or low dose steroids, communicate in English<br>Exclusions: pregnant, recently experienced injury, history of drug / alcohol abuse, taking experimental medication in last 6 months	1) Iyengar yoga – 2 classes per week for 6 weeks<br>p) Waitlist control	1) 11<br>p) 15	1) 29.9 (2.9)<br>p) 27.1 (4.2)	1) 11 (100)<br>p) 15 (100)	**Government** (NIAMS, NCCAM)
Singh (2011) [India][160]	RCT	Exclusion: not interested in yoga	1) Half-hour per day, 6 days per week for 7 weeks<br>p) Waitlist control	1) 40<br>p) 40	1) 35.1 (7.3)<br>p) 34.7 (7.3)	56 (70)	**Not reported**
Badsha (2009) [UAE][161]	NRT	1987 ACR RA criteria, aged >18 years, no physical disability	1) 12 sessions of yoga, 1 hour each<br>p) Waitlist control	1) 26<br>p) 21	1) 44.0 (10.0)<br>p) 46.2 (10.7)	Not reported	**Charity** (Emirates Arthritis Foundation), **Industry** (Abbott Pharmaceuticals)
Bosch (2009) [USA][162]	NRT	1987 ACR RA criteria, Post-menopause, aged 45-75 years, active RA, interested in doing yoga, functional class I-III, stable DMARDs for 4 weeks<br>Exclusions: any other major inflammatory disease, diabetes, systemic disease (e.g. COPD, congestive heart failure, stroke, chronic liver failure, renal disease), joint replacement in last 2 months, smokers, drug/alcohol abuse history, narcotic analgesic use (except for limited used <3 times a day of hydrocodone, codeine or propoxyphese	1) Hartha yoga, 3x per week – 75 mins<br>p) Patients who couldn't currently do yoga due to time constraints used as controls	1) 9<br>p) 7	1) 56.3 (7.6)<br>p) 66.7 (5.8)	1) 9 (100)<br>p) 7 (100)	**Not reported**

ACR = American College of Rheumatology, COPD = chronic obstructive pulmonary disease, DMARDs = disease modifying anti-rheumatic drugs, EULAR = European League Against Rheumatism, N = number, NCCAM = National Center for Complementary and Alternative Medicine, NIAMS = National Institute of Arthritis and Musculoskeletal and Skin Diseases, NRT = non-randomised trial, RA = rheumatoid arthritis, RCT = randomised controlled trial, SD = standard deviation, UAE = United Arab Emirates, USA = United States of America

Supplementary table 50 – Results from reviews and interventional studies of yoga in RA

Table – Yoga (RA), results and quality assessment

Outcome (outcome measure)	Study (date) [study type]	Standardised result, SMD (95% CI) unless otherwise stated	Natural result	AMSTAR2 quality	Rand. Seq.	Alloc. Conc.	Blind. Part.	Blind. Asses.
Pain	Wang (2018) [MA][64]	Yoga vs control<br>SMD -0.98 (-1.18, -0.78) †		Moderate				
	Cramer (2013) [SR][66]		Two RCTs, both at high risk of bias. The evidence for the effect of yoga on pain was graded as very low.	Moderate				
	Ward (2018) [RCT][158]	Yoga vs control<br>SMD 0.00 (-0.77, 0.77)	Pain VAS, BL / 9 weeks, mean (SD)<br>Yoga: 34 (18) / 33 (21)<br>Control: 31 (28) / 33 (32)		L	L	H/UC	L
	Evans (2013) [RCT][159]	Yoga vs control<br>SMD -0.12 (-0.90, 0.66)	Pain disability index, BL / 6 weeks, mean (SD)<br>Yoga: 26.5 (19.3) / 13.5 (14.5)<br>Control: 18.7 (18.7) / 15.4 (17.3)		H/UC	H/UC	H/UC	L
	Singh (2011) [RCT][160]	Yoga vs control<br>SMD -2.65 (-3.25, -2.04)	Pain, BL / 7 weeks, mean (SD)<br>Yoga: 1.90 (0.84) / 0.20 (0.516)<br>Control: 2.03 (0.7) / 1.92 (0.76)		H/UC	H/UC	H/UC	H/UC
	Bespoke meta-analysis including[158-160]	Yoga vs control<br>SMD -0.94 (-2.75, 0.88), I^2 94.9%<br>Excluding outlier[160]: SMD -0.06 (-0.61, 0.49), I^2 0%						
Function	Wang (2018) [MA][64]	Yoga vs control<br>SMD -0.55 (-0.83, -0.26)		Moderate				
	Ward (2018) [RCT][158]	Yoga vs control<br>SMD -0.81 (-1.61, -0.01)	HAQ, BL / 9 weeks, mean (SD)<br>Yoga: 0.51 (0.61) / 0.35 (0.35)<br>Control: 0.68 (0.63) / 0.83 (0.76)		L	L	H/UC	L
	Evans (2013) [RCT][159]	Yoga vs control<br>SMD 0.29 (-0.50, 1.07)	HAQ, BL / 6 weeks, mean (SD)<br>Yoga: 1.2 (0.8) / 0.9 (0.7)<br>Control: 0.9 (0.8) / 0.7 (0.7)		H/UC	H/UC	H/UC	L
	Bespoke meta-analysis including[158;159]	Yoga vs control<br>SMD -0.26 (-1.33, 0.82), I^2 72.8%						
	Badsha (2009) [NRT][161]		HAQ, BL / 8 weeks, mean<br>Yoga: 0.8 / 0.49<br>Control: 0.78 / 0.75					
	Bosch (2009) [NRT][162]		HAQ, BL / 10 weeks, mean (SD ‡)<br>Yoga: 1.10 (0.54) / 0.72 (0.54)<br>Control: 0.65 (0.48) / 0.77 (0.48)					

† Meta-analysis included RA and osteoarthritis patients, ‡ SD calculated from standard error

Alloc. Conc. = allocation concealment, AMSTAR2 = Assessing the Methodological Quality of Systematic Reviews, BL = baseline, Blind. Asses. = Blinded assessors, Blind. Part. = blinded participants, CI = confidence interval, H/UC = high / unclear risk of bias, HAQ = Health Assessment Questionnaire, L = low risk of bias, MA = meta-analysis, NRT = non-randomised trial, RA = rheumatoid arthritis, Rand. Seq. = random sequence generation, RCT = randomised controlled trial, SD = standard deviation, SMD = standardised mean difference, SR = systematic review, VAS = visual analogue scale

Table – Yoga (RA), results and quality assessment

Outcome (outcome measure)	Study (date) [study type]	Standardised result, SMD (95% CI) unless otherwise stated	Natural result	AMSTAR2 quality	Rand. Seq.	Alloc. Conc.	Blind. Part.	Blind. Asses.
Disease activity	Ward (2018) [RCT][158]	Yoga vs control<br>SMD 0.26 (-0.52, 1.03)	CDAI, BL / 9 weeks, mean (SD)<br>Yoga: 14.2 (6.2) / 11.5 (7.3)<br>Control: 14.5 (8.0) / 9.6 (7.6)		L	L	H/UC	L
	Evans (2013) [RCT][159]	Yoga vs control<br>SMD 0.00 (-0.78, 0.78)	DAS28, BL / 6 weeks, mean (SD)<br>Yoga: 4.6 (1.7) / 3.3 (1.2)<br>Control: 4.4 (2.1) / 3.3 (1.6)		H/UC	H/UC	H/UC	L
	Bespoke meta-analysis including[158;159]	Yoga vs control<br>SMD 0.13 (-0.42, 0.68), I^2 0%						
	Badsha (2009) [NRT][161]		DAS28, BL / 8 weeks, mean<br>Yoga: 3.9 / 3.3<br>Control: 3.8 / 3.9					
Tender joints	Badsha (2009) [NRT][161]		Tender joint count, BL / 8 weeks, mean<br>Yoga: 3.5 / 2.11<br>Control: 5 / 5.3					
Swollen joints	Singh (2011) [RCT][160]	Yoga vs control<br>SMD -1.65 (-2.15, -1.14)	Swollen joint count, BL / 7 weeks, mean (SD)<br>Yoga: 3.38 (1.31) / 0.43 (0.64)<br>Control: 3.25 (1.24) / 2.38 (1.55)		H/UC	H/UC	H/UC	H/UC
	Badsha (2009) [NRT][161]		Swollen joint count, BL / 8 weeks, mean<br>Yoga: 3.2 / 1<br>Control: 3.9 / 3.8					
Morning stiffness	Singh (2011) [RCT][160]	Yoga vs control<br>SMD -2.11 (-2.66, -1.56)	Morning stiffness (mins), BL / 7 weeks, mean (SD)<br>Yoga: 76.25 (20.56) / 13.15 (17.18)<br>Control: 81.68 (21.65) / 55.88 (22.90)		H/UC	H/UC	H/UC	H/UC
Fatigue	Ward (2018) [RCT][158]	Yoga vs control<br>SMD 0.29 (-0.48, 1.06)	BRAF-MDQ, BL / 9 weeks, mean (SD §)<br>Yoga: 5.7 (3.3) / 4.3 (4.2)<br>Control: 5.0 (5.0) / 3.3 (2.5)		L	L	H/UC	L
	Evans (2013) [RCT][159]	Yoga vs control<br>SMD 1.05 (0.22, 1.89)	FACIT-F, BL / 6 weeks, mean (SD)<br>Yoga: 32.3 (11.1) / 40.2 (6.4)<br>Control: 29.1 (15.0) / 29.7 (11.9)<br>[Higher scores indicate less fatigue]		H/UC	H/UC	H/UC	L
	Bespoke meta-analysis including[158;159]	Yoga vs control<br>SMD -0.37 (-1.69, 0.94), I^2 81.3%						
	Badsha (2009) [NRT][161]		Fatigue VAS, BL / 8 weeks, mean<br>Yoga: 34 / 26<br>Control: 32 / 44					

§ Mean (SD) estimated from median (IQR) using published formula[87]

Alloc. Conc. = allocation concealment, AMSTAR2 = Assessing the Methodological Quality of Systematic Reviews, BL = baseline, Blind. Asses. = Blinded assessors, Blind. Part. = blinded participants, BRAF-MDQ = Bristol Rheumatoid Arthritis Fatigue - Multidimensional Questionnaire, CDAI = Clinical Disease Activity Index, CI = confidence interval, DAS28 = Disease Activity Score 28, FACIT-F = Functional Assessment Chronic Illness Therapy – Fatigue, H/UC = high / unclear risk of bias, L = low risk of bias, RA = rheumatoid arthritis, Rand. Seq. = random sequence generation, RCT = randomised controlled trial, SD = standard deviation, SMD = standardised mean difference, VAS = visual analogue scale

Table – Yoga (RA), results and quality assessment

Outcome (outcome measure)	Study (date) [study type]	Standardised result, SMD (95% CI) unless otherwise stated	Natural result	AMSTAR2 quality	Rand. Seq.	Alloc. Conc.	Blind. Part.	Blind. Asses.
Quality of life	Ward (2018) [RCT][158]	Yoga vs control<br>SMD 0.14 (-0.63, 0.91)	EQ5D, BL / 9 weeks, mean (SD)<br>Yoga: 0.77 (0.17) / 0.76 (0.14)<br>Control: 0.77 (0.24) / 0.73 (0.26)		L	L	H/UC	L
Patient global	Wang (2018) [MA][64]	Yoga vs control, SF36 general health<br>SMD 0.80 (0.59, 1.01) †		Moderate				
	Evans (2013) [RCT][159]	Yoga vs control<br>SMD -0.67 (-1.47, 0.13)	Brief Symptom Inventory – global severity, BL / 6 weeks, mean (SD)<br>Yoga: 9.0 (5.2) / 5.3 (2.9)<br>Control: 7.5 (6.7) / 9.5 (7.8)		H/UC	H/UC	H/UC	L
	Badsha (2009) [NRT][161]		Patient global assessment, BL / 8 weeks, mean<br>Yoga: 32 / 25<br>Control: 26 / 40					
Mental-health	Wang (2018) [MA][64]	Yoga vs control, SF36 mental health<br>SMD 0.49 (0.15, 0.82)		Moderate				
Anxiety	Ward (2018) [RCT][158]	Yoga vs control<br>SMD -0.06 (-0.83, 0.71)	HADS - anxiety, BL / 9 weeks, mean (SD)<br>Yoga: 6.5 (2.8) / 4.7 (3.8)<br>Control: 4.9 (3.0) / 4.9 (2.7)		L	L	H/UC	L
	Evans (2013) [RCT][159]	Yoga vs control<br>SMD -0.30 (-1.08, 0.49)	Brief Symptom Inventory - anxiety, BL / 6 weeks, mean (SD)<br>Yoga: 3.0 (1.9) / 2.2 (1.6)<br>Control: 2.2 (2.3) / 2.9 (2.8)		H/UC	H/UC	H/UC	L
	Bespoke meta-analysis including[158;159]	Yoga vs control<br>SMD -0.18 (-0.72, -0.37), I^2 0%						
Depression	Ward (2018) [RCT][158]	Yoga vs control<br>SMD -0.04 (-0.81, 0.73)	HADS - depression, BL / 9 weeks, mean (SD)<br>Yoga: 3.4 (2.3) / 3.0 (1.9)<br>Control: 2.9 (2.6) / 3.1 (2.7)		L	L	H/UC	L
	Evans (2013) [RCT][159]	Yoga vs control<br>SMD -0.85 (-1.66, -0.04)	Brief Symptom Inventory - depression, BL / 6 weeks, mean (SD)<br>Yoga: 1.8 (1.8) / 1.0 (1.1)<br>Control: 2.1 (2.8) / 3.1 (3.1)		H/UC	H/UC	H/UC	L
	Bespoke meta-analysis including[158;159]	Yoga vs control<br>SMD -0.43 (-1.22, 0.36), I^2 49.8%						

Alloc. Conc. = allocation concealment, AMSTAR2 = Assessing the Methodological Quality of Systematic Reviews, BL = baseline, Blind. Asses. = Blinded assessors, Blind. Part. = blinded participants, CI = confidence interval, H/UC = high / unclear risk of bias, HADS = Hospital Anxiety and Depression Scale, L = low risk of bias, MA = meta-analysis, NRT = non-randomised trial, RA = rheumatoid arthritis, Rand. Seq. = random sequence generation, RCT = randomised controlled trial, SD = standard deviation, SMD = standardised mean difference, SR = systematic review

Table – Yoga (RA), results and quality assessment

Outcome (outcome measure)	Study (date) [study type]	Standardised result, SMD (95% CI) unless otherwise stated	Natural result	AMSTAR2 quality	Rand. Seq.	Alloc. Conc.	Blind. Part.	Blind. Asses.
Self-efficacy	Evans (2013) [RCT][159]	Yoga vs control<br>Function: SMD -0.08 (-0.86, 0.70)<br>Pain: SMD 0.50 (-0.29, 1.29)	ASES, BL / 6 weeks, mean (SD)<br>*Function*<br>Yoga: 6.20 (3.0) / 7.97 (2.22)<br>Control: 7.90 (2.25) / 8.16 (2.36)<br>*Pain*<br>Yoga: 12.96 (4.84) / 15.68 (2.90)<br>Control: 13.31 (4.07) / 13.84 (4.11)		H/UC	H/UC	H/UC	L
CRP	Singh (2011) [RCT][160]	Yoga vs control<br>SMD -0.03 (-0.47, 0.41)	CRP, BL / 7 weeks, mean (SD)<br>Yoga: 7.09 (0.95) / 6.43 (1.65)<br>Control: 6.59 (1.17) / 6.47 (1.04)		H/UC	H/UC	H/UC	H/UC
ESR	Badsha (2009) [NRT][161]		ESR, BL / 8 weeks, mean<br>Yoga: 31 / 27<br>Control: 24.9 / 25.7					

Alloc. Conc. = allocation concealment, AMSTAR2 = Assessing the Methodological Quality of Systematic Reviews, ASES = Arthritis Self-efficacy Scale, BL = baseline, Blind. Asses. = Blinded assessors, Blind. Part. = blinded participants, CI = confidence interval, CRP = C-reactive protein, ESR = erythrocyte sedimentation rate, H/UC = high / unclear risk of bias, L = low risk of bias, NRT = non-randomised trial, RA = rheumatoid arthritis, Rand. Seq. = random sequence generation, RCT = randomised controlled trial, SD = standard deviation, SMD = standardised mean difference, SR = systematic review

Supplementary table 51 – Description of reviews of studies of aerobic exercise in SLE

Table – Aerobic exercises (SLE), description of reviews

Authors (date)	Review type	Study type included	Exposure detail	Number of studies included	Funders
O'Dwyer (2017)[163]	MA	RCTs, quasi-RCTs	Aerobic exercise	7	**Not reported**
Wu (2017)[164]	MA	RCTs	Aerobic exercise	3	**Not reported**
Andrades (2017)[165]	SR	NRT	Aerobic exercise	1	**No funding**
del Pino-Sedeno (2016)[166]	SR	RCTs	Aerobic exercise	4	**Government** (Institute of Health)
Yuen (2014)[167]	SR	RCTs	Aerobic exercise	7	**Not reported – authors declared no conflicts of interest**

MA = meta-analysis, NRT = non-randomised trial, RCT = randomised controlled trial, SLE = systemic lupus erythematosus, SR = systematic review

Supplementary table 52 – Description of original studies of aerobic exercise in SLE

Table – Aerobic exercises (SLE), description of included studies

Author (date) [country]	Study design	Inclusion criteria	Exposure detail	N	Age years, mean (SD)	N (%) female	Funders
Abrahao (2016) [Brazil][168]	RCT	ACR SLE criteria, aged ≥18 years<br>Exclusions: absolute of relative contraindications to physical exercise according to the American College of Sports Medicine guidelines, not available for two consecutive sessions, participation in regular physical activity in past 6 months	1) Cardiovascular training – walking and exercise bike<br>2) Muscle strengthening training – free weights and elastic band exercises<br>p) Control	1) 21<br>2) 21<br>p) 21	1) 46.1 (14.1)<br>2) 43.8 (14.6)<br>p) 39.1 (14.4)	61 (96.8)	**Not reported**
Bostrom (2016) [Sweden][169]	RCT	1982 ACR criteria, haemoglobin value ≥100 g/L, s-creatinine ≤300 µmol/l, diastolic blood pressure ≤100 mm Hg at rest, stable prednisolone dose ≤15 mg/day, the ability to follow instructions and to perform a maximal bicycle ergometer exercise test<br>Exclusions: cerebrovascular disease, arthroplastic complication / surgery or pregnancy	1) First three months = education, supervised aerobic exercise, individual coaching, loan and use of heart monitor. Months four-nine = individual coaching, heart rate monitor, physical activity diary. Months nine-12 = heart rate monitor and diary<br>p) Asked not to change physical activity lifestyle during study.	1) 12<br>p) 13	1) 52 (10)<br>p) 53 (9)	1) 12 (100)<br>p) 13 (100)	**Charity** (Swedish Rheumatism Association, the Vardal Foundation), **University** (Karolinska Institutet)
Tench (2003) [UK][170]	RCT	1997 ACR criteria, aged 18-55 years, stable medication for ≥2 months<br>Exclusions: active severe myositis, nephritis, neurological involvement or cardiac or pulmonary disease, pregnant, exercise >1 week	1) Asked to exercise at home for ≥3x per week – main exercise was walking but patients were encouraged to take up other forms of exercise (e.g. cycling, swimming). Also seen every 2 weeks for supervised exercise session<br>p1) Relaxation group – listened to relaxing audiotape 3x per week in darkened, warm, quiet room<br>p2) No intervention – asked to continue normal daily activity pattern	1) 33<br>p1) 29<br>p2) 32	39 (7.8 †)	1) 33 (100)<br>p1) 29 (100)<br>p2) 32 (100)	**Charity** (Arthritis Research Campaign), **Hospital** (St Bartholomew's Hospital), **Professional body** (British Medical Association)
Robb-Nicholson (1989) [USA][171]	RCT	Exclusions: Serum creatinine ≥265 µmol/l, haematocrit ≤30%, previous myocardial infarction, previous cerebrovascular accident, severe cognitive impairment, resting diastolic blood pressure ≥100 mmHg, severe arthritis of ≥3 weight-bearing joints, using beta-blockers	1) Exercise at home for 30 mins per day to attain 60-80% of max heart rate<br>p) Non aerobic stretching for 30 mins 3x per week	1) 10<br>p) 10	1) 40.9 (9.8)<br>p) 38.3 (10.9)	1) 10 (100)<br>p) 10 (100)	**Government** (NIH, NHLBI), **Charity** (Lupus Foundation of America), **Industry** (J. R. Reynolds Corporation Grant)
Soriano-Maldonado (2018) [Spain][172]	NRT	1997 SLE criteria, treatment stability for ≥6 months, not performing exercise<br>Exclusions: biological treatment in previous 6 months or need of prednisone dose >10 mg/day, background cardiovascular disease in previous year, contraindications for exercise, other rheumatic conditions, pregnancy, active acute or chronic infections, neoplasms, acute renal failure, cardiac or pulmonary involvement, BMI >35, not being able to read, understand or sign consent	1) 75 min session 2x per week – aerobic exercise on a treadmill<br>p) Usual care – verbal information about healthy lifestyle	1) 26<br>p) 32	1) 43.0 (15.1)<br>p) 44.8 (13.1)	1) 26 (100)<br>p) 32 (100)	**Charity** (Fundación para la Investigación Biosanitaria de Andalucía Oriental), **Professional body** (Ilustre Colegio Oficial de Médicos de Granada)

† SD calculated for standard error in paper; ACR = American College of Rheumatology, N = number, NHLBI = National Heart, Lung, and Blood Institute, NIH = National Institutes of Health, NRT = non-randomised trial, RCT = randomised controlled trial, SD = standard deviation, SLE = systemic lupus erythematosus, UK = United Kingdom, USA = United States of America

Table – Aerobic exercises (SLE), description of included studies

Author (date) [country]	Study design	Inclusion criteria	Exposure detail	N	Age years, mean (SD)	N (%) female	Funders
dos Reis-Neto (2013) [Brazil][173]	NRT	1997 SLE criteria, aged 18-45 years<br>Exclusions: haemoglobin <10 mg/dl, neuropsychiatric, pulmonary, articular or vascular damage that would not allow the practice of exercise; coronary disease; heart failure (functional class 5II); pulmonary hypertension; uncontrolled hypertension; creatinine ≥1.4 mg/dl; BMI ≥35 kg/m2 ; diabetes mellitus; uncontrolled hypothyroidism; smoking in the last 12 months; pregnancy; menopause; use of statins or regular practice of exercise in the past 3 months and overlap with other autoimmune rheumatic diseases, except anti-phospholipid syndrome.	1) Exercise protocol, 3x per week for 60 mins (10 min warm up, 40 min walking, 10 min cool down<br>p) Non-exercise control	1) 18<br>p) 20	1) 35.3 (6.8)<br>p) 30.8 (7.2)	1) 18 (100)<br>p) 20 (100)	**Government** (Sao Paulo State Research Foundation)
Carvalho (2005) [Brazil][174]	NRT	1982 ACR criteria, aged 18-55 years<br>Exclusions: Haemoglobin values <10gm/dl, neurological disease or cardiovascular accident sequels, psychosis, depression, under psychiatric care, respiratory disease (pulmonary hypertension, pulmonary fibrosis, bronchitis, asthma, emphysema), heart insufficiency, functional class III, history of myocardial infarction/ischemic heart disease, diastolic blood pressure >100mmhg, active nephritis with creatinine levels >3.0 mg/dl, SLEDAI >8, thyroid dysfunction, diabetes, hip/knee prosthesis or aseptic bone necrosis, deep venous thrombosis in lower limbs, severe arthritis in ≥3 weight bearing joints, pregnancy, regular physical activity ≥3x per week, concomitant rheumatic disease	1) Supervised training program 3x per week – 10 minutes warm-up, 40 minutes walking, 10 minutes cool down<br>p) Did not participate in training	1) 41<br>p) 19	1) 36.2 (10.8)<br>p) 35.2 (9.1)	1) 41 (100)<br>p) 19 (100)	**Government** (Sao Paulo Research Foundation, Coordenação de Aperfeicoamento de Pessoal de Nível Superior)

ACR = American College of Rheumatology, N = number, NRT = non-randomised trial, SD = standard deviation, SLE = systemic lupus erythematosus

Supplementary table 53 – Results from reviews and interventional studies of aerobic exercise in SLE

Table – Aerobic exercise (SLE), results and quality assessment

Outcome (outcome measure)	Study (date) [study type]	Standardised result, SMD (95% CI) unless otherwise stated	Natural result	AMSTAR2 quality	Rand. Seq.	Alloc. Conc.	Blind. Part.	Blind. Asses.
Pain	Carvalho (2005) [NRT][174]	Exercise vs control at 12 weeks<br>SMD -0.45 (-1.00, 0.11)	Pain VAS, BL / 12 weeks, mean (SD)<br>Exercise: 2.02 (2.73) / 1.70 (2.69)<br>Control: 2.47 (2.71) / 3.01 (3.44)					
Function	Carvalho (2005) [NRT][174]	Exercise vs control at 12 weeks<br>SMD -0.49 (-1.04, 0.06)	HAQ, BL / 12 weeks, mean (SD)<br>Exercise: 0.14 (0.21) / 0.06 (0.19)<br>Control: 0.23 (0.27) / 0.38 (1.14)					
Disease activity	O'Dwyer (2017) [MA][163]		Disease activity<br>MD = 0.01 (-0.54, 0.56)	Moderate				
	Abrahao (2016) [RCT][168]	Aerobic exercise vs control<br>SMD 0.57 (-0.04, 1.19)	SLEDAI, BL / 12 weeks, mean (SD)<br>Aerobic: 1.8 (0.6) / 1.6 (0.9)<br>Muscle strengthening: 1.4 (0.6) / 1.3 (0.5)<br>Control: 2.3 (1.7) / 1.2 (0.4)		L	L	H/UC	L
	Bostrom (2016) [RCT][169]	Exercise vs control at 12 months<br>SMD 0.44 (-0.35, 1.24)	SLEDAI, BL / 12 months, mean (SD †)<br>Exercise: 3.0 (6.7) / 4.0 (3.4)<br>Control: 1.7 (2.5) / 2.3 (4.2)		L	L	H/UC	L
	Tench (2003) [RCT][170]	Exercise vs control at 12 weeks<br>Exercise vs relaxation: SMD 0.00 (-0.50, 0.50)<br>Exercise vs control: SMD -0.22 (-0.71, 0.27)	SLAM, BL / 12 weeks, mean (SD †)<br>Exercise: 5.3 (3.9) / 5.0 (3.9)<br>Relaxation: 5.7 (3.9) / 5.0 (3.1)<br>Control: 5.7 (3.1) / 5.7 (2.3)		L	H/UC	H/UC	H/UC
	Bespoke meta-analysis including[168-170]	Exercise vs control<br>SMD 0.22 (-0.32, 0.76), I^2 55.7%<br>[Exercise vs control comparison used for Tench[170]]						
	dos Reis-Neto (2013) [NRT][173]	Exercise vs control at 16 weeks<br>SMD -0.17 (-0.81, 0.47)	SLEDAI, BL / 16 weeks, mean (SD)<br>Exercise: 2.0 (2.1) / 2.4 (2.3)<br>Control: 2.4 (2.3) / 3.1 (5.3)					

† mean (SD) estimated from median (interquartile range) using published formula[87]

Alloc. Conc. = allocation concealment, AMSTAR2 = Assessing the Methodological Quality of Systematic Reviews, BL = baseline, Blind. Asses. = Blinded assessors, Blind. Part. = blinded participants, CI = confidence interval, H/UC = high / unclear risk of bias, HAQ = Health Assessment Questionnaire, L = low risk of bias, NRT = non-randomised trial, Rand. Seq. = random sequence generation, RCT = randomised controlled trial, SD = standard deviation, SLAM = systemic lupus activity measure, SLE = systemic lupus erythematosus, SLEDAI = Systemic Lupus Erythematosus Disease Activity Index, SMD = standardised mean difference, VAS = visual analogue scale

Table – Aerobic exercise (SLE), results and quality assessment

Outcome (outcome measure)	Study (date) [study type]	Standardised result, SMD (95% CI) unless otherwise stated	Natural result	AMSTAR2 quality	Rand. Seq.	Alloc. Conc.	Blind. Part.	Blind. Asses.
Fatigue	O'Dwyer (2017) [MA][163]		Fatigue<br>MD -0.61 (-1.19, -0.02))	Moderate				
	Wu (2017) [MA][164]		Fatigue Severity Scale, mean difference<br>MD -0.52 (-0.92, -0.13)	Moderate				
	del Pino-Sedeno (2016) [SR][166]		4/4 studies reported significant improvements in fatigue compared to non-exercise controls	Moderate				
	Yuen (2014) [SR][167]		4/7 studies reported reductions in fatigue following exercise	Moderate				
	Tench (2003) [RCT][170]	Exercise vs control at 12 weeks<br>*Chalder Fatigue Scale*<br>Exercise vs relaxation: SMD -0.33 (-0.83, 0.17)<br>Exercise vs control: SMD -0.68 (-1.18, -0.18)<br>*Fatigue VAS*<br>Exercise vs relaxation: SMD 0.06 (-0.44, 0.55)<br>Exercise vs control: SMD -0.53 (-1.03, -0.04)<br>*Fatigue Severity scale*<br>Exercise vs relaxation: SMD -0.35 (-0.85, 0.16)<br>Exercise vs control: SMD -0.35 (-0.84, 0.14)	Chalder fatigue scale, BL / 12 weeks, mean (SD ‡)<br>Exercise: 22 (7.5) / 15 (8.6)<br>Relaxation: 24 (8.6) / 18 (9.7)<br>Control: 24 (9.6) / 21 (9.1)<br>Fatigue VAS, BL / 12 weeks, mean (SD ‡)<br>Exercise: 300 (57) / 239 (86)<br>Relaxation: 290 (59) / 234 (97)<br>Control: 286 (68) / 283 (79)<br>Fatigue Severity Scale, BL / 12 weeks, mean (SD ‡)<br>Exercise: 5.4 (1.1) / 4.8 (1.7)<br>Relaxation: 5.4 (1.1) / 5.3 (1.1)<br>Control: 5.5 (1.1) / 5.4 (1.7)		L	H/UC	H/UC	H/UC
	Robb-Nicholson (1989) [RCT][171]	Exercise vs control, change BL-8 weeks<br>Stamina: SMD 1.63 (0.60, 2.65)<br>Comparative energy: SMD 1.00 (0.06, 1.93)<br>Sufficient energy: SMD 1.44 (0.44, 2.43)<br>Tiredness: SMD 0.86 (-0.06, 1.78)	Fatigue VAS, change BL-8 weeks, mean (SD)<br>*Q1 – Stamina*<br>Exercise: 1.9 (1.7)<br>Control: -1.3 (2.2)<br>*Q2 – Comparative energy*<br>Exercise: 1.1 (1.9)<br>Control: -1.0 (2.3)<br>*Q3 – Sufficient energy*<br>Exercise: 1.7 (2.2)<br>Control: -0.9 (1.3)<br>*Q4 – Tiredness*<br>Exercise: 1.7 (2.5)<br>Control: -0.2 (1.9)		H/UC	H/UC	H/UC	L
	Bespoke meta-analysis including[170;171]	Exercise vs control<br>SMD -0.72 (-1.16, -0.28), I^2 0%<br>[Exercise vs control comparison used for Tench[170]; Tiredness VAS used for Robb-Nicholson[171]]						

‡ SD calculated for standard error in paper

Alloc. Conc. = allocation concealment, AMSTAR2 = Assessing the Methodological Quality of Systematic Reviews, BL = baseline, Blind. Asses. = Blinded assessors, Blind. Part. = blinded participants, CI = confidence interval, H/UC = high / unclear risk of bias, L = low risk of bias, Rand. Seq. = random sequence generation, RCT = randomised controlled trial, SD = standard deviation, SLE = systemic lupus erythematosus, SMD = standardised mean difference

Table – Aerobic exercise (SLE), results and quality assessment

Outcome (outcome measure)	Study (date) [study type]	Standardised result, SMD (95% CI) unless otherwise stated	Natural result	AMSTAR2 quality	Rand. Seq.	Alloc. Conc.	Blind. Part.	Blind. Asses.
Fatigue	Carvalho (2005) [NRT][174]	Exercise vs control at 12 weeks<br>SMD -0.44 (-0.99, 0.11)	Fatigue scale (0-7), BL / 12 weeks, mean (SD)<br>Exercise: 3.57 (1.47) / 2.68 (1.33)<br>Control: 3.28 (1.33) / 3.29 (1.47)					
Quality of life	O'Dwyer (2017) [MA][163]		2/4 studies reported that QoL improved – the 2 studies that did not report improvements were RCTs, the two that did were NRTs	Moderate				
Anxiety	Tench (2003) [RCT][170]	Exercise vs control at 12 weeks<br>Exercise vs relaxation: SMD -0.22 (-0.72, 0.28)<br>Exercise vs control: SMD -0.18 (-0.66, 0.31)	HADS - anxiety, BL / 12 weeks, mean (SD ‡)<br>Exercise: 9.0 (4.6) / 7.4 (4.6)<br>Relaxation: 9.9 (4.8) / 8.5 (5.4)<br>Control: 8.8 (4.0) / 8.2 (4.5)		L	H/UC	H/UC	H/UC
Depression	O'Dwyer (2017) [MA][163]	Exercise vs control<br>SMD -0.40 (-0.71, -0.09)		Moderate				
	Abrahao (2016) [RCT][168]	Aerobic exercise vs control<br>SMD 0.00 (-0.61, 0.61)	BDI, BL / 12 weeks, mean (SD)<br>Aerobic: 20.6 (5.3) / 20.1 (7.1)<br>Muscle strengthening: 19.4 (5.0) / 17.3 (4.4)<br>Control: 19.1 (5.6) / 20.1 (5.9)		L	L	H/UC	L
	Tench (2003) [RCT][170]	Exercise vs control at 12 weeks<br>Exercise vs relaxation: SMD -0.52 (-1.03, -0.02)<br>Exercise vs control: SMD -0.30 (-0.79, 0.19)	HADS - depression, BL / 12 weeks, mean (SD ‡)<br>Exercise: 5.8 (4.0) / 4.6 (4.0)<br>Relaxation: 7.9 (4.3) / 6.9 (4.8)<br>Control: 6.4 (3.4) / 5.7 (3.4)		L	H/UC	H/UC	H/UC
	Bespoke meta-analysis including[168;170]	Exercise vs control<br>SMD -0.18 (-0.56, 0.20), I^2 0%<br>[Exercise vs control comparison used for Tench[170]]						
	Carvalho (2005) [NRT][174]	Exercise vs control at 12 weeks<br>SMD -0.70 (-1.26, -0.14)	BDI, BL / 12 weeks, mean (SD)<br>Exercise: 8.37 (12.79) / 2.90 (3.00)<br>Control: 5.79 (6.44) / 6.63 (8.50)					
CRP	Soriano-Maldonado (2018) [NRT][172]		CRP, change BL-12 weeks, median (SE)<br>Exercise : 0.17 (0.59)<br>Control: -0.24 (0.55)<br>Mean difference in change (95% CI)<br>0.411 (-1.25, 2.07)					
Cardiovascular risk factors	Andrades (2017) [SR][165]		Concluded that the effect of physical activity on cardiovascular risk factors has been poorly studied	Moderate				

‡ SD calculated for standard error in paper

Alloc. Conc. = allocation concealment, AMSTAR2 = Assessing the Methodological Quality of Systematic Reviews, BDI = Beck Depression Inventory, BL = baseline, Blind. Asses. = Blinded assessors, Blind. Part. = blinded participants, CI = confidence interval, CRP = C-reactive protein, H/UC = high / unclear risk of bias, HADS = Hospital Anxiety and Depression Scale, L = low risk of bias, NRT = non-randomised trial, Rand. Seq. = random sequence generation, RCT = randomised controlled trial, SD = standard deviation, SE = standard error, SLE = systemic lupus erythematosus, SLEDAI = Systemic Lupus Erythematosus Disease Activity Index, SMD = standardised mean difference

Table – Aerobic exercise (SLE), SF36 results at final follow-up, mean (SD)

Author (date)	PCS	MCS	GH	PF	RP	RE	SF	BP	V	MH
Abrahao (2016) [Exercise][168]			49.3 (22.2)	55.5 (28.4)	49.8 (34.8)	51.6 (25.9)	48.3 (22.1)	41.7 (28.4)	63.5 (21.2)	44.3 (19.4)
Abrahao (2016) [Control][168]			39.2 (23.2)	41.4 (15.7)	29.7 (17.4)	22.9 (11.0)	35.2 (27.9)	24.6 (14.4)	26.6 (14.3)	33.7 (18.9)
Bostrom (2016) [Exercise][169] †			34.8 (18.4)	75.0 (21.0)	20.8 (41.9)	55.6 (83.9)	64.6 (21.0)	38 (9.2)	34.2 (33.5)	64.0 (13.4)
Bostrom (2016) [Control][169] †			51.0 (37.0)	66.7 (20.8)	54.2 (83.1)	66.7 (69.2)	75.0 (36.4)	64.3 (19.1)	50.8 (27.0)	76.7 (15.0)
Tench (2003) [Exercise][170]				69 (29)	50 (77) †				46 (29)	
Tench (2003) [Relaxation][170]				57 (32)	50 (78) †				41 (27)	
Tench (2003) [Control][170]				60 (28)	29 (49) †				35 (28)	
Carvalho (2005) [Exercise][174]			73.17 (18.97)	91.10 (11.37)	85.24 (27.32) §	79.66 (31.58)	88.56 (15.28)	74.32 (20.59)	76.22 (14.61)	77.85 (16.45)
Carvalho (2005) [Control][174]			62.37 (26.08)	86.84 (11.21)	60.53 (43.55) §	80.74 (30.08)	81.74 (19.58)	67.89 (21.98)	66.05 (20.04)	72.63 (19.6)

† mean (SD) estimated from median (interquartile range) using published formula[87]

§ labelled "physical fitness" in paper

BP = bodily pain, FU = follow-up, GH = general health, MCS = mental component score, MH = mental health, PCS = physical component score, PF = physical function, RE = role emotional, RP = role physical, SD = standard deviation, SF = social functioning, SLE = systemic lupus erythematosus, V = vitality

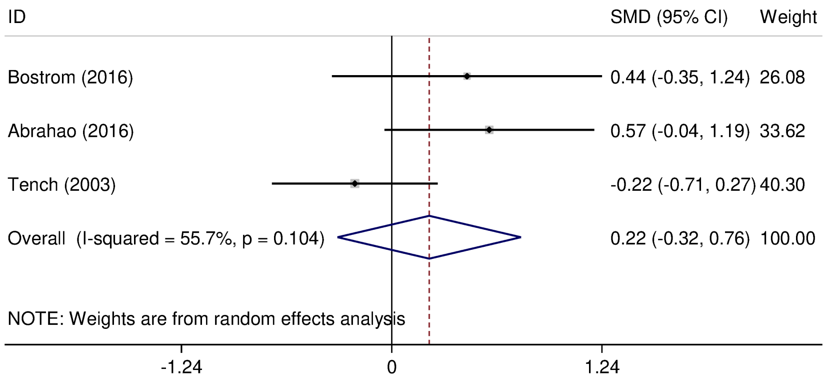

Figure – The effect of aerobic exercise on disease activity (SLE)

Supplementary table 54 – Description of reviews of studies of aerobic + muscle strengthening exercise in SLE

Table – Aerobic + muscle strengthening exercises (SLE), description of reviews

Authors (date)	Review type	Study type included	Exposure detail	Number of studies included	Funders
O'Dwyer (2017)[163]	MA	RCTs, quasi-RCTs	Aerobic vs muscle strengthening exercise	3	**Not reported**
del Pino-Sedeno (2016)[166]	SR	RCTs	Aerobic + muscle strengthening exercise	1	**Government** (Institute of Health)

MA = meta-analysis, RCT = randomised controlled trial, SLE = systemic lupus erythematosus, SR = systematic review

Supplementary table 55 – Description of original studies of aerobic + muscle strengthening exercise in SLE

Table – Aerobic + muscle strengthening exercises (SLE), description of included studies

Author (date) [country]	Study design	Inclusion criteria	Exposure detail	N	Age years, mean (SD)	N (%) female	Funders
Abrahao (2016) [Brazil][168]	RCT	ACR SLE criteria, aged ≥18 years<br>Exclusions: absolute of relative contraindications to physical exercise according to the American College of Sports Medicine guidelines, not available for two consecutive sessions, participation in regular physical activity in past 6 months	1) Cardiovascular training – walking and exercise bike<br>2) Muscle strengthening training – free weights and elastic band exercises<br>p) Control	1) 21<br>2) 21<br>p) 21	1) 46.1 (14.1)<br>2) 43.8 (14.6)<br>p) 39.1 (14.4)	61 (96.8)	**Not reported**
Bogdanovic (2015) [Serbia][175]	RCT	1982 ACR SLE criteria, SLEDAI ≤5, no immunosuppressive therapy	1) Aerobic training on exercise bike<br>2) Isotonic muscle strengthening exercises	1) 30<br>2) 30	1) 38.8 (12.6)<br>2) 47.9 (11.5)	1) 30 (100)<br>2) 30 (100)	**Government** (Ministry of Science)
Miossi (2012) [Brazil][176]	RCT	Physically inactive for ≥6 months, women, ACR SLE criteria, aged 20-40 years, SLEDAI ≤4<br>Exclusions: cardiovascular dysfunction, rhythm and conduction disorders, musculoskeletal disturbances, kidney and pulmonary involvement, peripheral neuropathy, use of tobacco, treatment with lipid lowering drugs, fibromyalgia, use of chronotropic or anti-hypertensive drugs	1) Supervised exercise training program – 35-40 mins of muscle strengthening training, 30 mins treadmill, 5 mins stretching<br>p) Usual care	1) 14<br>p) 10	1) 31.4 (5.9)<br>p) 31.0 (4.8)	1) 14 (100)<br>p) 10 (100)	**Industry** (Bank of America Merrill Lynch), **Government** (São Paulo Research Foundation, Conselho Nacional de Desenvolvimento Científico e Tecnológico), **Charity** (Federico Foundation)
Ramsey-Goldman (2000) [USA][177]	RCT	ACR SLE criteria<br>Exclusions: significant functional impairment due to heart disease, neurological diseases, chronic pulmonary disease, cognitive impairment that prevented following exercise directions, or conditions preventing exercise (e.g. avascular necrosis), symptomatic anaemia (haemoglobin <8g/dl), advanced renal insufficiency (creatinine >4mg/dl or creatinine clearance <10 ml/min), or thrombocytopenia (platelet count <50000/mm^3	1) Aerobic – patients instructed to exercise to 70-80% max heart rate.<br>2) Muscle strengthening – muscle strengthening and stretching	1) 5<br>2) 5	Mean (range)<br>1) 33.9 (24.2-49.9)<br>2) 43.2 (19.1-64.2)	1) 5 (100)<br>2) 5 (100)	**University** (Northwestern University), **Government** (NIH), **Charity** (Lupus Foundation of America, Arthritis Foundation)

ACR = American College of Rheumatology, N = number, NIH = National Institutes of Health, RCT = randomised controlled trial, SD = standard deviation, SLE = systemic lupus erythematosus, SLEDAI = systemic lupus erythematosus disease activity index, USA = United States of America

Table – Aerobic + muscle strengthening exercises (SLE), description of included studies

Author (date) [country]	Study design	Inclusion criteria	Exposure detail	N	Age years, mean (SD)	N (%) female	Funders
Timoteo (2018) [Brazil][178]	NRT	ACR SLE criteria	1) 4 months, 3x per week, individual with physiotherapist, muscle strengthening exercise with barbell and plates, bike training added in after 1 month<br>p) Non-exercise control	1) 5<br>p) 9	median (IQR)<br>1) 38.0 (30.0, 41.5<br>p) 45.0 (31.5, 52.0)	1) 5 (100)<br>p) 9 (100)	**Government** (Financiadora de Estudos e Projetos, Fundacao de Amparo a Pesquisa do Estado de Minas Gerais, Conselho Nacional de Desenvolvimento Cientifico e Tecnologico), **Charity** (Fundacao de Ensino e Pesquisa de Uberaba)

ACR = American College of Rheumatology, N = number, NRT = non-randomised trial, SD = standard deviation, SLE = systemic lupus erythematosus

Supplementary table 56 – Results from reviews and interventional studies of aerobic + muscle strengthening exercise in SLE

Table – Aerobic + muscle strengthening exercises (SLE), results and quality assessment

Outcome (outcome measure)	Study (date) [study type]	Standardised result, SMD (95% CI) unless otherwise stated	Natural result	AMSTAR2 quality	Rand. Seq.	Alloc. Conc.	Blind. Part.	Blind. Asses.
Disease activity	O'Dwyer (2017) [MA][163]		Compared aerobic vs muscle strengthening – no difference in disease activity scores	Moderate				
	Abrahao (2016) [RCT][168]	Aerobic exercise vs muscle strengthening exercise at 12 weeks<br>SMD 0.41 (-0.20, 1.02)	SLEDAI, BL / 12 weeks, mean (SD)<br>Aerobic: 1.8 (0.6) / 1.6 (0.9)<br>Muscle strengthening: 1.4 (0.6) / 1.3 (0.5)<br>Control: 2.3 (1.7) / 1.2 (0.4)		L	L	H/UC	L
	Miossi (2012) [RCT][176]	Exercise vs control at 12 weeks<br>SMD -0.42 (-1.24, 0.40)	SLEDAI, BL / 12 weeks, mean (SD)<br>Exercise: 0.9 (1.5) / 0.8 (1.2)<br>Control: 1.0 (1.3) / 1.3 (1.2)		H/UC	H/UC	H/UC	H/UC
	Ramsey-Goldman (2000) [RCT][177]	Aerobic exercise vs muscle strengthening exercise, change BL-7 months<br>SMD 0.91 (-0.41, 2.22)	SLAM, change BL – 7 months, mean (SD †)<br>Aerobic exercise: 2.80 (2.17)<br>Muscle strengthening exercise: 0.40 (3.05)		H/UC	H/UC	H/UC	H/UC
	Bespoke meta-analysis including[168;177]	Aerobic vs muscle strengthening exercise<br>SMD 0.63 (0.08, 1.19), I^2 0% [in favour of muscle strengthening exercise]						
Fatigue	O'Dwyer (2017) [MA][163]		Compared aerobic vs muscle strengthening – vitality scores on the SF36 higher in aerobic groups	Moderate				
	del Pino-Sedeno (2016) [SR][166]		One study reported significant improvement in fatigue compared to controls	Moderate				
	Bogdanovic (2015) [RCT][175]	Aerobic exercise vs muscle strengthening exercise at 6 weeks<br>SMD 0.00 (-0.51, 0.51)	Fatigue Severity Scale, BL / 6 weeks, mean (SD)<br>Aerobic: 53.6 (6.3) / 29.2 (7.9)<br>Muscle strengthening: 53.6 (6.3) / 29.2 (7.9) [sic]		H/UC	H/UC	H/UC	H/UC
	Ramsey-Goldman (2000) [RCT][177]	Aerobic exercise vs muscle strengthening exercise, change BL-7 months<br>SMD -0.05 (-1.29, 1.19)	Fatigue Severity Scale, change BL – 7 months, mean (SD †)<br>Aerobic exercise: -0.71 (0.60)<br>Muscle strengthening exercise: -0.68 (0.62)		H/UC	H/UC	H/UC	H/UC
	Bespoke meta-analysis including[175;177]	Aerobic vs muscle strengthening exercise<br>SMD -0.01 (-0.48, 0.46), I^2 0%						

† SD calculated from 95% CI

Alloc. Conc. = allocation concealment, AMSTAR2 = Assessing the Methodological Quality of Systematic Reviews, BL = baseline, Blind. Asses. = Blinded assessors, Blind. Part. = blinded participants, CI = confidence interval, H/UC = high / unclear risk of bias, L = low risk of bias, Rand. Seq. = random sequence generation, RCT = randomised controlled trial, SD = standard deviation, SLE = systemic lupus erythematosus, SLEDAI = systemic lupus erythematosus disease activity index, SMD = standardised mean difference

Table – Aerobic + muscle strengthening exercises (SLE), results and quality assessment

Outcome (outcome measure)	Study (date) [study type]	Standardised result, SMD (95% CI) unless otherwise stated	Natural result	AMSTAR2 quality	Rand. Seq.	Alloc. Conc.	Blind. Part.	Blind. Asses.
Depression	O'Dwyer (2017) [MA][163]		Compared aerobic vs muscle strengthening – no difference in depression scores	Moderate				
	Abrahao (2016) [RCT][168]	Aerobic exercise vs muscle strengthening exercise at 12 weeks<br>SMD 0.47 (-0.14, 1.09)	BDI, BL / 12 weeks, mean (SD)<br>Aerobic: 20.6 (5.3) / 20.1 (7.1)<br>Muscle strengthening: 19.4 (5.0) / 17.3 (4.4)<br>Control: 19.1 (5.6) / 20.1 (5.9)		L	L	H/UC	L
	Bogdanovic (2015) [RCT][175]		BDI at 6 weeks, N(%)<br>Aerobic:<br>Normal = 4 (13.3)<br>Mild = 18 (60.0)<br>Borderline = 5 (16.7)<br>Moderate = 3 (10.0)<br>Severe = 0 (0.0)<br>Muscle strengthening:<br>Normal = 2 (6.7)<br>Mild = 19 (63.3)<br>Borderline = 6 (23.3)<br>Moderate = 3 (6.6)<br>Severe = 0 (0.0)		H/UC	H/UC	H/UC	H/UC

Alloc. Conc. = allocation concealment, AMSTAR2 = Assessing the Methodological Quality of Systematic Reviews, BDI = Beck Depression Inventory, BL = baseline, Blind. Asses. = Blinded assessors, Blind. Part. = blinded participants, CI = confidence interval, H/UC = high / unclear risk of bias, L = low risk of bias, Rand. Seq. = random sequence generation, RCT = randomised controlled trial, SD = standard deviation, SLE = systemic lupus erythematosus, SMD = standardised mean difference

Table – Aerobic + muscle strengthening exercise (SLE), SF36 results at final follow-up, mean (SD)

Author (date)	PCS	MCS	GH	PF	RP	RE	SF	BP	V	MH
Abrahao (2016) [Aerobic][168]			49.3 (22.2)	55.5 (28.4)	49.8 (34.8)	51.6 (25.9)	48.3 (22.1)	41.7 (28.4)	63.5 (21.2)	44.3 (19.4)
Abrahao (2016) [muscle strengthening][168]			35.1 (12.9)	44.2 (14.3)	27.7 (15.1)	37.3 (28.7)	38.6 (26.6)	31.7 (15.0)	26.6 (17.4)	39.5 (17.3)
Ramsey-Goldman (2000) [Aerobic][177]	7.00 (13.5) §									
Ramsey-Goldman (2000) [Muscle strengthening][177]	2.50 (29.2) §									
Timoteo (2018) [Exercise][178]			65 (33) §	73 (41) §	83 (50) §	72 (84) §	60 (82) §	81 (41) §	58 (43) §	65 (52) §
Timoteo (2018) [Control][178]			62 (38) §	71 (38) §	50 (66) §	56 (87) §	60 (27) §	45 (46) §	39 (12) §	50 (32) §

§ Change from BL-7 months

BP = bodily pain, FU = follow-up, GH = general health, MCS = mental component score, MH = mental health, PCS = physical component score, PF = physical function, RE = role emotional, RP = role physical, SD = standard deviation, SF = social functioning, SLE = systemic lupus erythematosus, V = vitality

Supplementary table 57 – Description of original studies of muscle strengthening exercise in SLE

Table – Aerobic + muscle strengthening exercises (SLE), description of included studies

Author (date) [country]	Study design	Inclusion criteria	Exposure detail	N	Age years, mean (SD)	N (%) female	Funders
Abrahao (2016) [Brazil][168]	RCT	ACR SLE criteria, aged ≥18 years<br>Exclusions: absolute of relative contraindications to physical exercise according to the American College of Sports Medicine guidelines, not available for two consecutive sessions, participation in regular physical activity in past 6 months	1) Cardiovascular training – walking and exercise bike<br>2) Muscle strengthening training – free weights and elastic band exercises<br>p) Control	1) 21<br>2) 21<br>p) 21	1) 46.1 (14.1)<br>2) 43.8 (14.6)<br>p) 39.1 (14.4)	61 (96.8)	**Not reported**

ACR = American College of Rheumatology, N = number, RCT = randomised controlled trial, SD = standard deviation, SLE = systemic lupus erythematosus

Supplementary table 58 – Results from reviews and interventional studies of muscle strengthening exercise in SLE

Table – Muscle strengthening exercises (SLE), results and quality assessment

Outcome (outcome measure)	Study (date) [study type]	Standardised result, SMD (95% CI) unless otherwise stated	Natural result	AMSTAR2 quality	Rand. Seq.	Alloc. Conc.	Blind. Part.	Blind. Asses.
Disease activity	Abrahao (2016) [RCT][168]	Muscle strengthening exercise vs control at 12 weeks<br>SMD 0.22 (-0.39, 0.83)	SLEDAI, BL / 12 weeks, mean (SD)<br>Aerobic: 1.8 (0.6) / 1.6 (0.9)<br>Muscle strengthening: 1.4 (0.6) / 1.3 (0.5)<br>Control: 2.3 (1.7) / 1.2 (0.4)		L	L	H/UC	L
Depression	Abrahao (2016) [RCT][168]	Muscle strengthening exercise vs control at 12 weeks<br>SMD -0.54 (-1.15, 0.08)	BDI, BL / 12 weeks, mean (SD)<br>Aerobic: 20.6 (5.3) / 20.1 (7.1)<br>Mstrengthening: 19.4 (5.0) / 17.3 (4.4)<br>Control: 19.1 (5.6) / 20.1 (5.9)		L	L	H/UC	L

Alloc. Conc. = allocation concealment, AMSTAR2 = Assessing the Methodological Quality of Systematic Reviews, BDI = Beck Depression Inventory, BL = baseline, Blind. Asses. = Blinded assessors, Blind. Part. = blinded participants, CI = confidence interval, H/UC = high / unclear risk of bias, L = low risk of bias, Rand. Seq. = random sequence generation, RCT = randomised controlled trial, SD = standard deviation, SLE = systemic lupus erythematosus, SLEDAI = systemic lupus erythematosus disease activity index, SMD = standardised mean difference

Table – Muscle strengthening exercise (SLE), SF36 results at final follow-up, mean (SD)

Author (date)	PCS	MCS	GH	PF	RP	RE	SF	BP	V	MH
Abrahao (2016) [Muscle strengthening][168]			35.1 (12.9)	44.2 (14.3)	27.7 (15.1)	37.3 (28.7)	38.6 (26.6)	31.7 (15.0)	26.6 (17.4)	39.5 (17.3)
Abrahao (2016) [Control][168]			39.2 (23.2)	41.4 (15.7)	29.7 (17.4)	22.9 (11.0)	35.2 (27.9)	24.6 (14.4)	26.6 (14.3)	33.7 (18.9)

BP = bodily pain, FU = follow-up, GH = general health, MCS = mental component score, MH = mental health, PCS = physical component score, PF = physical function, RE = role emotional, RP = role physical, SD = standard deviation, SF = social functioning, SLE = systemic lupus erythematosus, V = vitality

Supplementary table 59 – Description of reviews of studies of aerobic exercise in axSpA

Table – Aerobic exercise (axSpA), description of reviews

Authors (date)	Review type	Study type included	Exposure detail	Number of studies included	Funders
Regel (2017)[179]	SR	RCTs	Aerobic exercises including walking and rehabilitation	2	**Professional bodies** (European League Against Rheumatism, Assessment of Spondyloarthritis international Society)
O'Dwyer (2014)[180]	SR	RCTs	Unsupervised exercise interventions vs Supervised	4	**Not reported –** Authors declared no conflicts of interest

axSpA = axial spondyloarthritis, RCT = randomised controlled trial, SR = systematic review

Supplementary table 60 – Description of original studies of aerobic exercise in axSpA

Table –Aerobic exercise (axSpA), description of included studies

Author (date) [country]	Study design	Inclusion criteria	Exposure detail	N	Age years, mean (SD)	N (%) female	Funders
Karahan (2016) [Turkey][181]	RCT	Modified New York criteria, aged 18-65 years, lack of regular exercise during previous 6 months, ability to understand questionnaires<br>Exclusions: cardiopulmonary dysfunction, central or peripheral neurological disease, issues hindering standing, psychiatric disorder, visual disorder, hearing disorder	1) Exergram – Microsoft Xbox 360 Kinect game console – 30 mins per day, 5x per week<br>p) No exercise program	1) 28<br>p) 29	1) 36.1 (12.4)<br>p) 36.6 (11.3)	1) 6 (21.4)<br>p) 7 (24.1)	**Not reported –** Authors declared no conflicts of interest
Jennings (2015) [Brazil][182]	RCT	Modified New York criteria, aged 18-60 years, functional class I-II, stable DMARDs for 3 months, stable steroids of 4 weeks<br>Exclusions: Uncontrolled hypertension, history of heart failure / coronary revascularization, history of syncope or exercise induced arrhythmias, decompensated Type 1 diabetes, severe psychiatric diseases, fibromyalgia, other medical conditions more incapacitating that AS, history of regular physical activity in last 6 months, arthroplasty in last year, other condition preventing walking	1) Aerobic exercise (walking) with stretching, 80 mins 3x per week<br>p) Stretching exercises only	1) 35<br>p) 35	1) 42.9 (9.9)<br>p) 40.2 (9.3)	1) 9 (25.7)<br>p) 12 (34.3)	**Charity** (São Paulo Research Foundation)
Niedermann (2013) [Switzerland][183]	RCT	Modified New York Criteria, aged >18 years, communicate in German<br>Exclusions: Severe heart disease, inability to use exercise bike	1) Cardiovascular training and flexibility<br>p) Attention control – Monthly 2.5 hour discussion groups on coping strategies and mindfulness	1) 53<br>p) 53	1) 50.1 (11.9)<br>p) 47.6 (12.4)	1) 19 (35.8)<br>p) 19 (35.8)	**Hospital** (University Hospital Zurich), **Charity** (Schweizerische Vereinigung Morbus Bechterew, Böhni Foundation for Research in Rheumatology, Zurich Rheumatology Foundation, Physiotherapie Wissenschaften Foundation), **Professional body** (Swiss Physiotherapy Association)

axSpA = axial spondyloarthritis, int. = intervention, N = number, RCT = randomised controlled trial, SD = standard deviation

Table –Aerobic exercise (axSpA), description of included studies

Author (date) [country]	Study design	Inclusion criteria	Exposure detail	N	Age years, mean (SD)	N (%) female	Funders
Sweeney (2002) [UK][185]	RCT	Aged 16-65 years	1) Delivered a home exercise regime video, booklet and wall chart<br>p) Usual care	1) 100<br>p) 100	1) 47 (10.2)<br>p) 47 (9.6)	1) 30 (30%) F<br>p) 32 (32%) F	**Industry** (Bupa), **Charity** (National Ankylosing Spondylitis Society, John Coates Charitable Trust, and Col. W.W. Pilkington Trust)
Ajeganova (2016) [Sweden][184] §	Single arm int.	Aged 18-80 years, clear clinical need for rehab, outpatient physiotherapy not sufficient<br>Exclusions: severe handicap that made evaluation assessments impossible, difficulties in answering questionnaires in Swedish	Intervention took place in Marbella and Tenerife. Training was performed individually and in groups – at least 3 scheduled activities per day with a minimum of 45 mins each, 5 days per week. These consisted dynamic and static exercises on land and in a temperature controlled pool	37	56.5 (1..3)	45.9%	**Charity** (The Swedish Rheumatism Association and King Gustav V 80 year's Foundation)
Brophy (2013) [UK][186]	Pros. cohort	AS patients part of the MRC / NISCHR patient research cohort	International Physical activity questionnaire	329	55 (14)	21%	**Government** (MRC, NISCHR)
Ward (2002) [USA][187]	Pros. cohort	Modified New York criteria, aged ≥18 years, communicate in English, completed ≥3 functional disability questionnaires<br>Exclusions: inflammatory bowel disease	Self-reported number of days per week with exercise – computed exercise minutes per week	212	47.8 (13.6)	63 (29.7)	**Charity** (Bartman Fondation)
Uhrin (2000) [USA][188]	Pros. cohort	Modified New York criteria, aged ≥18 years, communicate in English<br>Exclusions: history of inflammatory bowel disease	Self-reported number of days per week with exercise	220	47.5 (13.7)	70 (31.8)	**Charity** (Bartman Fondation)

§ Ajeganova et al[184] included patients with many different rheumatic diseases, including juvenile idiopathic arthritis, and analysed the data together. The only outcome that was only measured in one of the rheumatic diseases included in this review was the BASFI and therefore this study is in the AS section of the report.
axSpA = axial spondyloarthritis, MRC = Medical Research Council, N = number, NISCHR = National Institute for Social Care and Health Research, Pros. = prospective, RCT = randomised controlled trial, SD = standard deviation, UK = United Kingdom, USA = United States of America

Supplementary table 61 – Results from reviews and interventional studies of aerobic exercise in axSpA

Table – Aerobic exercise (axSpA), results and quality assessment

Outcome (outcome measure)	Study (date) [study type]	Standardised result, SMD (95% CI) unless otherwise stated	Natural result	AMSTAR2 quality	Rand. Seq.	Alloc. Conc.	Blind. Part.	Blind. Asses.
Pain	Karahan (2016) [RCT][181]	Exercise vs control at 8 weeks<br>SMD -0.67 (-1.21, -0.14)	Pain VAS, BL / 8 weeks, mean (SD)<br>Exercise: 4.9 (2.0) / 3.6 (1.7)<br>Control: 5.1 (2.2) / 5.0 (2.4)		H/UC	L	H/UC	H/UC
	Niedermann (2013) [RCT][183]	Exercise vs control at 3 months<br>SMD -0.07 (-0.45, 0.31)	BAS-G – pain, BL / 3 months, mean (SD †)<br>Exercise: 3.2 (2.0) / 3.25 (2.1)<br>Control: 3.5 (2.5) / 3.39 (2.0)		L	L	H/UC	L
	Bespoke meta-analysis including[181;183]	Exercise vs control<br>SMD -0.34 (-0.93, 0.25), I^2 69.2%						
Function	Regel (2017) [SR][179]		One study reporting no difference	Moderate				
	Karahan (2016) [RCT][181]	Exercise vs control at 8 weeks<br>SMD -0.66 (-1.19, -0.13)	BASFI, BL / 8 weeks, mean (SD)<br>Exercise: 3.7 (1.5) / 2.9 (1.3)<br>Control: 3.9 (1.6) / 3.9 (1.7)		H/UC	L	H/UC	H/UC
	Jennings (2015) [RCT][182]	Exercise vs control at 12 months<br>SMD 0.01 (-0.46, 0.48)<br>HAQ-S: SMD -0.14 (-0.61, 0.33)	BASFI, BL / 12 months / 24 months, mean (SD)<br>Exercise: 4.28 (2.78) / 3.37 (2.49) / 3.47 (2.48)<br>Control: 4.27 (2.32) / 3.34 (2.07) / 3.73 (2.19)<br>HAQ-S, BL / 12 weeks / 24 weeks, mean (SD)<br>Exercise: 1.04 (0.59) / 0.84 (0.52) / 0.92 (0.57)<br>Control: 1.01 (0.55) / 0.92 (0.62) / 0.97 (0.59)		L	L	H/UC	L
	Niedermann (2013) [RCT][183]	Exercise vs control at 3 months<br>SMD 0.09 (-0.29, 0.47)	BASFI, BL / 3 months, mean (SD †)<br>Exercise: 2.4 (1.9) / 2.53 (1.5)<br>Control: 2.4 (2.1) / 2.40 (1.5)		L	L	H/UC	L
	Sweeney (2002) [RCT][185]	Exercise vs control at 6 months<br>SMD -0.15 (-0.43, 0.13)	BASFI, BL / 6 months, mean (SD)<br>Exercise: 3.5 (2.4) / 3.06 (2.35)<br>Control: 3.6 (2.4) / 3.43 (2.61)		H/UC	H/UC	H/UC	H/UC
	Bespoke meta-analysis including[181-183;185]	Exercise vs control<br>SMD -0.14 (-0.41, 0.13), I^2 44.4%						
	Ajeganova (2016) [Single arm int.][184] §		BASFI, BL / 4 weeks / 1 year, mean (SD)<br>4.14 (2.57) / 2.34 (1.83) / 3.70 (2.27)					

† SD calculated from standard error in paper

Alloc. Conc. = allocation concealment, AMSTAR2 = Assessing the Methodological Quality of Systematic Reviews, axSpA = axial spondyloarthritis, BAS-G = Bath Ankylosing Spondylitis Global Score, BASFI = Bath Ankylosing Spondylitis Functional Index, BL = baseline, Blind. Asses. = Blinded assessors, Blind. Part. = blinded participants, CI = confidence interval, H/UC = high / unclear risk of bias, HAQ-S = Health Assessment Questionnaire for the Spondyloarthropaties, L = low risk of bias, Rand. Seq. = random sequence generation, RCT = randomised controlled trial, SD = standard deviation, SMD = standardised mean difference, VAS = visual analogue scale

Table – Aerobic exercise (axSpA), results and quality assessment

Outcome (outcome measure)	Study (date) [study type]	Standardised result, SMD (95% CI) unless otherwise stated	Natural result	AMSTAR2 quality	Rand. Seq.	Alloc. Conc.	Blind. Part.	Blind. Asses.
Disease activity	Regel (2017) [SR][179]		One study reporting small effects in both groups	Moderate				
	O'Dwyer (2014) [SR][180]		0/2 studies reported improvements in disease activity in favour of aerobic exercise	Moderate				
	Karahan (2016) [RCT][181]	Exercise vs control at 8 weeks<br>SMD -0.51 (-1.04, 0.02)	BASDAI, BL / 8 weeks, mean (SD)<br>Exercise: 4.1 (1.8) / 3.2 (1.3)<br>Control: 4.2 (2.1) / 4.1 (2.1)		H/UC	L	H/UC	H/UC
	Jennings (2015) [RCT][182]	Exercise vs control at 12 months<br>BASDAI: SMD -0.02 (-0.49, 0.45)<br>ASDAS-CRP: SMD -0.02 (-0.49, 0.45)	BASDAI, BL / 12 months / 24 months, mean (SD)<br>Exercise: 3.46 (2.39) / 2.75 (2.12) / 2.87 (1.97)<br>Control: 3.62 (2.06) / 2.79 (1.99) / 3.27 (2.07)<br>ASDAS-CRP, BL / 12 months / 24 months, mean (SD)<br>Exercise: 2.44 (1.07) / 1.98 (0.93) / 2.10 (0.92)<br>Control: 2.24 (0.91) / 2.00 (0.94) / 2.24 (0.89)		L	L	H/UC	L
	Niedermann (2013) [RCT][183]	Exercise vs control at 3 months<br>BASDAI: SMD -0.19 (-0.57, 0.20)<br>ASDAS: 0.09 (-0.29, 0.47)	BASDAI, BL / 3 months, mean (SD †)<br>Exercise: 3.3 (1.9) / 3.07 (1.5)<br>Control: 3.6 (2.1) / 3.35 (1.5)<br>ASDAS, BL / 3 months, mean (SD †)<br>Exercise: 2.2 (0.8) / 2.26 (1.09)<br>Control: 2.3 (1.0) / 2.16 (1.09)		L	L	H/UC	L
	Sweeney (2002) [RCT][185]	Exercise vs control at 6 months<br>SMD 0.08 (-0.20, 0.35)	BASDAI, BL / 6 months, mean (SD)<br>Exercise: 3.9 (2.4) / 3.65 (2.00)<br>Control: 3.8 (2.3) / 3.49 (2.16)		H/UC	H/UC	H/UC	H/UC
	Bespoke meta-analysis including[181-183;185]	Exercise vs control<br>SMD -0.10 (-0.34, 0.13), I^2 28.2%						

† SD calculated from standard error in paper

Alloc. Conc. = allocation concealment, AMSTAR2 = Assessing the Methodological Quality of Systematic Reviews, axSpA = axial spondyloarthritis, ASDAS-CRP = Ankylosing Spondylitis Disease Activity Score (C-reactive protein), BASDAI = Bath Ankylosing Spondylitis Disease Activity Index, BL = baseline, Blind. Asses. = Blinded assessors, Blind. Part. = blinded participants, CI = confidence interval, H/UC = high / unclear risk of bias, L = low risk of bias, Rand. Seq. = random sequence generation, RCT = randomised controlled trial, SD = standard deviation, SMD = standardised mean difference

Table – Aerobic exercise (axSpA), results and quality assessment

Outcome (outcome measure)	Study (date) [study type]	Standardised result, SMD (95% CI) unless otherwise stated	Natural result	AMSTAR2 quality	Rand. Seq.	Alloc. Conc.	Blind. Part.	Blind. Asses.
Spinal mobility	Regel (2017) [SR][179]		One study reporting small effect sizes in both arms	Moderate				
	O'Dwyer (2014) [SR][180]		0/2 studies reported improvements in spinal mobility in favour of aerobic exercise	Moderate				
	Jennings (2015) [RCT][182]	Exercise vs control at 12 months<br>SMD 0.14 (-0.33, 0.61)	BASMI, BL / 12 months / 24 months, mean (SD)<br>Exercise: 5.15 (1.95) / 4.93 (1.94) / 4.95 (2.03)<br>Control: 4.79 (2.22) / 4.65 (2.14) / 4.61 (2.24)		L	L	H/UC	L
	Niedermann (2013) [RCT][183]	Exercise vs control at 3 months<br>SMD -0.22 (-0.60, 0.17)	BASMI, BL / 3 months, mean (SD †)<br>Exercise: 2.9 (2.1) / 2.64 (1.8)<br>Control: 2.8 (1.9) / 3.02 (1.7)		L	L	H/UC	L
	Bespoke meta-analysis including[182;183]	Exercise vs control<br>SMD -0.07 (-0.41, 0.28), I^2 24.1%						
Patient global	Sweeney (2002) [RCT][185]	Exercise vs control at 6 months<br>SMD -0.00 (-0.28, 0.27)	BAS-G, BL / 6 months, mean (SD)<br>Exercise: 4.0 (2.6) / 3.60 (2.61)<br>Control: 3.7 (2.6) / 3.61 (2.81)		H/UC	H/UC	H/UC	H/UC
QoL	O'Dwyer (2014) [SR][180]		0/2 studies reported improvements in QoL in favour of aerobic exercise	Moderate				
	Karahan (2016) [RCT][181]	Exercise vs control at 8 weeks<br>SMD -0.64 (-1.17, -0.11)	ASQOL, BL / 8 weeks, mean (SD)<br>Exercise: 9.5 (6.1) / 6.8 (4.3)<br>Control: 10.2 (6.0) / 10.3 (6.4)		H/UC	L	H/UC	H/UC
	Niedermann (2013) [RCT][183]	Exercise vs control at 3 months<br>SMD 0.06 (-0.32, 0.44)	EQ5D, BL / 3 months, mean (SD †)<br>Exercise: 64.5 (22.0) / 64.24 (22.2)<br>Control: 65.9 (21.2) / 63.01 (21.3)		L	L	H/UC	L
	Bespoke meta-analysis including[181;183]	Exercise vs control<br>SMD -0.32 (-0.89, 0.25), I^2 67.2%						

† SD calculated from standard error in paper

Alloc. Conc. = allocation concealment, AMSTAR2 = Assessing the Methodological Quality of Systematic Reviews, ASQOL = Ankylosing Spondylitis Quality of Life, axSpA = axial spondyloarthritis, BAS-G = Bath Ankylosing Spondylitis Global Score, BASMI = Bath Ankylosing Spondylitis Metrology Index, BL = baseline, Blind. Asses. = Blinded assessors, Blind. Part. = blinded participants, CI = confidence interval, H/UC = high / unclear risk of bias, L = low risk of bias, Rand. Seq. = random sequence generation, RCT = randomised controlled trial, SD = standard deviation, SMD = standardised mean difference

Table – Aerobic exercise (axSpA), results and quality assessment

Outcome (outcome measure)	Study (date) [study type]	Standardised result, SMD (95% CI) unless otherwise stated	Natural result	AMSTAR2 quality	Rand. Seq.	Alloc. Conc.	Blind. Part.	Blind. Asses.
Fatigue	Niedermann (2013) [RCT][183]	Exercise vs control at 3 months<br>SMD -0.24 (-0.63, 0.14)	BASDAI - fatigue, BL / 3 months, mean (SD †)<br>Exercise: 4.4 (2.4) / 3.73 (2.3)<br>Control: 5.0 (2.7) / 4.29 (2.3)		L	L	H/UC	L
Anxiety	Niedermann (2013) [RCT][183]	Exercise vs control at 3 months<br>SMD -0.12 (-0.51, 0.26)	HADS-Anxiety, BL / 3 months, mean (SD †)<br>Exercise: 6.9 (5.3) / 6.27 (2.5)<br>Control: 6.7 (4.5) / 6.58 (2.5)		L	L	H/UC	L
Depression	Niedermann (2013) [RCT][183]	Exercise vs control at 3 months<br>SMD 0.28 (-0.11, 0.66)	HADS-Depression, BL / 3 months, mean (SD †)<br>Exercise: 5.2 (4.4) / 5.10 (2.3)<br>Control: 5.0 (4.5) / 4.48 (2.2)		L	L	H/UC	L
Self-efficacy	Sweeney (2002) [RCT][185]	Exercise vs control at 6 months<br>SMD 0.48 (0.20, 0.77)	Stanford self-efficacy - pain, BL / 6 months, mean (SD)<br>Exercise: 6.49 (1.8) / 6.80 (1.21)<br>Control: 6.06 (2.1) / 6.24 (1.1)		H/UC	H/UC	H/UC	H/UC
CRP	Jennings (2015) [RCT][182]	Exercise vs control at 12 months<br>SMD -0.07 (-0.54, 0.40)	CRP, BL / 12 months / 24 months, mean (SD)<br>Exercise: 10.49 (11.90) / 7.14 (8.21) / 6.53 (7.33)<br>Control: 6.01 (7.33) / 4.95 (4.86 / 7.84 (11.59)		L	L	H/UC	L
	Niedermann (2013) [RCT][183]	Exercise vs control at 3 months<br>SMD 0.17 (-0.21, 0.55)	CRP, BL / 3 months, mean (SD †)<br>Exercise: 7.5 (9.8) / 6.27 (7.9)<br>Control: 6.4 (8.7) / 4.95 (7.8)		L	L	H/UC	L
	Bespoke meta-analysis including[182;183]	Exercise vs control<br>SMD 0.07 (-0.22, 0.37), I^2 0.0%						
ESR	Jennings (2015) [RCT][182]	Exercise vs control at 12 months<br>SMD 0.27 (-0.20, 0.74)	ESR, BL / 12 months / 24 months, mean (SD)<br>Exercise: 18.5 (12.5) / 17.5 (12.8) / 20.5 (15.2)<br>Control: 17.1 (13.6) / 14.1 (12.5) / 14.7 (9.7)		L	L	H/UC	L
Walk-test	Jennings (2015) [RCT][182]	Exercise vs control at 12 months<br>SMD 0.85 (0.36, 1.34)	6MWT, BL / 12 months / 24 months, mean (SD)<br>Exercise: 443.14 (51.50) / 479.97 (54.56) / 473.53 (54.68)<br>Control: 423.81 (64.17) / 434.48 (52.56) / 432.14 (45.87)		L	L	H/UC	L

† SD calculated from standard error in paper

6MWT = six minute walk test, Alloc. Conc. = allocation concealment, AMSTAR2 = Assessing the Methodological Quality of Systematic Reviews, axSpA = axial spondyloarthritis, BL = baseline, Blind. Asses. = Blinded assessors, Blind. Part. = blinded participants, CI = confidence interval, CRP = C-reactive protein, ESR = erythrocyte Sedimentation Rate, H/UC = high / unclear risk of bias, HADS = Hospital Anxiety and Depression Scales, L = low risk of bias, Rand. Seq. = random sequence generation, RCT = randomised controlled trial, SD = standard deviation, SMD = standardised mean difference

Table – Aerobic + muscle strengthening exercise (axSpA), SF36 results at final follow-up, mean (SD)

Author (date)	PCS	MCS	GH	PF	RP	RE	SF	BP	V	MH
Jennings (2015) [exercise][182]			56.6 (23.1)	66.3 (19.8)	57.9 (42.8)	58.0 (46.0)	74.1 (24.4)	65.0 (22.6)	62.7 (24.1)	71.1 (21.6)
Jennings (2015) [control][182]			46.9 (22.9)	62.4 (20.8)	47.9 (40.8)	54.1 (40.5)	66.6 (26.8)	60.3 (22.5)	59.6 (21.7)	66.3 (22.0)

axSpA = axial spondyloarthritis, BL = baseline, BP = bodily pain, FU = follow-up, GH = general health, IQR = interquartile range, MCS = mental component score, MH = mental health, PCS = physical component score, PF = physical function, RE = role emotional, RP = role physical, SD = standard deviation, SF = social functioning, V = vitality

Supplementary table 62 – Results from observational studies of aerobic exercise in axSpA

Table – Aerobic exercise (axSpA), results and quality assessment of observational studies

Outcome (outcome measure)	Study (date) [study type]	Standardised result, SMD (95% CI) unless otherwise stated	Natural result	Study Pop.	Attr.	Prog. Meas.	Outc. Meas.	Conf.	Stats.
Pain	Uhrin (2000) [Pros. cohort][188]		Pain, <30 exercise mins per week = ref, adjusted mean<br>31-90 mins per week: 0.13<br>91-200 mins per week: 0.008<br>> 200 mins per week: -0.035	L	L	M	L	L	L
Function	Brophy (2013) [Pros. cohort][186]		BASFI, improvement in function compared to low physical activity, regression coefficient (95% CI)<br>*Low disease activity*<br>Medium physical activity: -8.9 (-17.9, 0.02)<br>High physical activity: -14.0 (-23.3, -4.8)<br>*Moderate disease activity*<br>Medium physical activity: -15.4 (-24.4, -6.5)<br>High physical activity: -21.3 (-30.2, 12.5)<br>*High disease activity*<br>Medium physical activity: -8.0 (-16.0, -0.08)<br>High physical activity: -19.9 (-27.9, -11.8)<br>[Analyses controlled for age]	L	M	M	L	M	L
	Ward (2002) [Pros. cohort][187]		Progression in HAQ over time - regression coefficient (95% CI) - univariable<br>per 10 min recreational exercise per week: 0.0000 (-0.003, 0.004)<br>per 1 day back exercise per week: -0.0022 (-0.0034, -0.001)	L	M	M	L	H	L
	Uhrin (2000) [Pros. cohort][188]		HAQ , <30 exercise mins per week = ref, adjusted mean<br>31-90 mins per week: -0.01<br>91-200 mins per week: -0.006<br>> 200 mins per week: -0.32	L	L	M	L	L	L
Stiffness	Uhrin (2000) [Pros. cohort][188]		Stiffness, <30 exercise mins per week = ref, adjusted mean<br>31-90 mins per week: 0.41<br>91-200 mins per week: -0.51<br>> 200 mins per week: -1.51	L	L	M	L	L	L

Attr. = attrition, axSpA = axial spondyloarthritis, , BASFI = Bath Ankylosing Spondylitis Functional Index, CI = confidence interval, Conf. = confounding, HAQ = Health Assessment Questionnaire, L = low risk of bias, M = moderate risk of bias, Outc. Meas = outcome measurement, Prog. Meas. = prognostic factor measurement, Pros = prospective, Rand. Seq. = random sequence generation, Stats. = statistical analysis, Study Pop. = study population

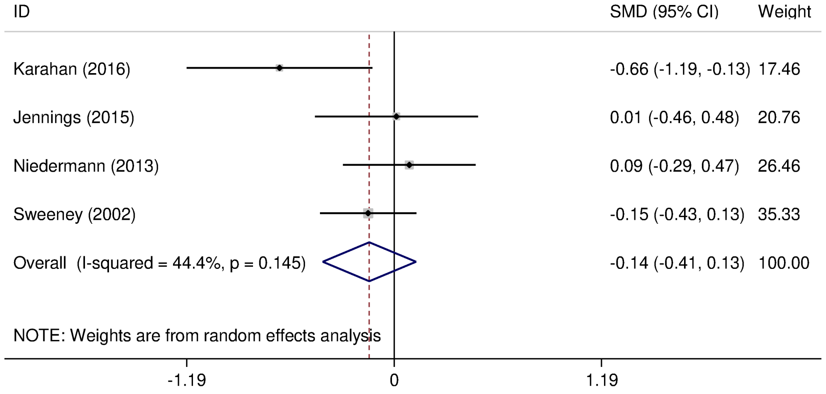

Figure – The effect of aerobic exercise on function (axSpA)

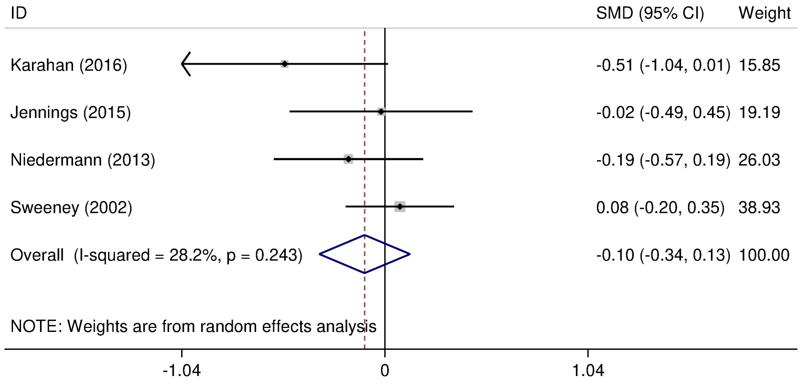

Figure – The effect of aerobic exercise on disease activity (axSpA)

Supplementary table 63 – Description of reviews of studies of aerobic + muscle strengthening exercise in axSpA

Table – Aerobic + muscle strengthening exercises (axSpA), description of reviews

Authors (date)	Review type	Study type included	Exposure detail	Number of studies included	Funders
Pecourneau (2018)[189]	MA	RCTs	Aerobic exercises of various types (including aquatic, Pilates, home-based – all included together)	8	**Industry** (Abbott)
Chang (2016)[190]	MA	RCTs	Specific exercise and physical therapies involving stretching, muscular strengthening and respiratory training vs standard exercise	8	**Not reported –** Authors declared no conflicts of interest
Millner (2016)[191]	MA	RCTs	A range of interventions included: Pilates, physiotherapy, group and home exercises, aerobic and flexibility	11	**Government** (Commonwealth Department of Health, Australia), **Industry** (Abbive)
Liang (2015)[192]	MA	RCTs	Home-based exercise programs including muscle relaxation, exercises for spine, range of motion, stretching, muscle strengthening and respiratory exercises	6	**Not reported –** Authors declared no conflicts of interest
Liang (2015)[193]	MA	RCTs	Exercise regimes + TNFi treatment	5	**Not reported –** Authors declared no conflicts of interest
Martins (2014)[194]	MA	RCTs	Exercise regimes vs normal care	3	**Not reported**
Regel (2017)[179]	SR	RCTs	Aerobic exercises including walking and rehabilitation	1	**Professional bodies** (European League Against Rheumatism, Assessment of Spondyloarthritis international Society)
Sharan (2017)[195]	SR	RCTs, reviews	Studies of aerobic and strengthening included	30	**Not reported –** Authors declared no conflicts of interest
O'Dwyer (2014)[180]	SR	RCTs	Therapeutic exercise interventions vs controls	7	**Not reported –** Authors declared no conflicts of interest

axSpA = axial spondyloarthritis, MA = meta-analysis, RCT = randomised controlled trial, SR = systematic review, TNFi = tumour necrosis factor inhibitors

BMJ Publishing Group Limited (BMJ) disclaims all liability and responsibility arising from any reliance placed on this supplemental material which has been supplied by the author(s)

Supplementary table 64 – Description of original studies of aerobic + muscle strengthening exercise in axSpA

Table – Aerobic + muscle strengthening exercises (axSpA), description of included studies

Author (date) [country]	Study design	Inclusion criteria	Exposure detail	N	Age years, mean (SD)	N (%) female	Funders
Sveaas (2019) [Norway][196]	RCT	ASAS axSpA criteria, aged 18-70 years, no change in TNFi in past three months, BASDAI ≥3.5 Not performed regular cardiorespiratory or strength exercises in past year<br>Exclusions: CV disease, other comorbidity involving reducing exercise capacity, inability to participate in supervised sessions, pregnancy	1) High intensity cardiorespiratory and strengthening exercises<br>p) Usual care	1) 50<br>p) 50	Mean (range)<br>1) 45.1 (23-68)<br>p) 47.2 (24-69)	1) 25 (50.0)<br>p) 28 (56.0)	**Charity** (Norwegian Foundation for Postgraduate Physiotherapist), **Professional body** (Norwegian Rheumatology Association)
Sveaas (2018) [Norway][197]	RCT	ASAS axSpA criteria, aged 18-70 years, no change in TNFi in past three months, BASDAI ≥3.5 Not performed regular cardiorespiratory or strength exercises in past year<br>Exclusions: pregnancy and established cardiovascular disease	1) 2x per week – high intensity interval training on treadmill and then 20 mins strength exercises. 1x per week – aerobic exercise session for 40 mins<br>p) Usual care	1) 10<br>p) 14	1) 46.6 (13.6)<br>p) 49.9 (11.1)	1) 8 (80.0)<br>p) 4 (28.6)	**Not reported**
Aydin (2016) [Turkey][198]	RCT	New York criteria, aged 20-65 years old, not practising regular exercise during previous 6 months, able to understand questionnaires, no co-existing systemic disease, no TNFi therapy, heart functional class I-III	1) Home based exercise – callisthenic exercises (consecutive and repetitive exercises aimed at training large muscle groups through aerobic and step routines)<br>2) Hospital based exercises – same as above	1) 19<br>2) 18	1) 33.5 (7.7)<br>2) 35.8 (8.1)	1) 8 (42.1)<br>2) 9 (50.0)	**Not reported –** authors declared no conflict of interest
Rosu (2014) [Romania][199]	RCT	Modified New York Criteria, axial disease subset<br>Exclusions: Peripheral or mixed AS, those with total ankyloses of the spine, patients with ESR >30mm/hr or CRP >2x upper limit of normal	1) Pilates, McKenzie and Heckscher training – 20 mins Pilates, 20 mins of Heckscher (aerobic exercise aiming to correct head posture, 10 mins McKenzie (aerobic exercise for lower back)<br>2) Step aerobic exercises for 20 mins + 10 min warm up and cool down	1) 48<br>2) 48	1) 25.3 (3.8)<br>2) 25.0 (3.8)	1) 9 (18.8)<br>2) 8 (16.7)	**Not reported –** Authors declared no conflicts of interest
Sveaas (2014) [Norway][200]	RCT	ASAS axSpA criteria, aged 18-70 years, no change in TNFi in past three months, BASDAI ≥3.5 Not performed regular cardiorespiratory or strength exercises in past year<br>Exclusions: pregnancy and established cardiovascular disease, inability to perform weekly exercise session in Oslo	1) 2x per week – high intensity interval training on treadmill and then 20 mins strength exercises. 1x per week – aerobic exercise session for 40 mins<br>p) Usual care	1) 10<br>p) 14	1) 46.6 (13.6)<br>p) 49.9 (11.1)	1) 8 (80.0)<br>p) 4 (28.6)	**Charity** (Norwegian Foundation for Postgraduate Physiotherapist)

axSpA = axial spondyloarthritis, ASAS = Assessment of Spondyloarthritis, BASDAI = Bath Ankylosing Spondylitis Disease Activity Index, N = number, Pros. = prospective, RCT = randomised controlled trial, SD = standard deviation, TNFi = Tumour Necrosis Factor Inhibitors, UK = United Kingdom

Table – Aerobic + muscle strengthening exercises (axSpA), description of included studies

Author (date) [country]	Study design	Inclusion criteria	Exposure detail	N	Age years, mean (SD)	N (%) female	Funders
Kjeken (2013) [Norway][201]	RCT	Modified New York criteria, aged 18-65 years, BASDAI≥40mm, communicate in Norwegian<br>Exclusions: Coronary heart disease, pregnancy, impaired function due to other medical problems, surgery or rehabilitations in last 6 months, cognitive or mental impairment, started biologic drug therapy	1) Physiotherapist designed weekly exercises programme in gym, pool and outdoors – involved muscle strength and fitness<br>p) Waitlist control	1) 46<br>p) 49	1) 49.4 (10.3)<br>p) 48.6 (9.4)	1) 10 (21.7)<br>p) 23 (46.9)	**Government** (Health South-East, Norway)
Analay (2003) [Turkey][202]	RCT	Amor criteria, aged 18-55 years, able to participate in exercise<br>Exclusions: systemic organ involvement, severe deformities or limited hip and knee joint motion preventing cycling, treated by physiotherapist in last 3 months or practising regular exercise and those received DMARDs	1) Intensive exercise regime given by physiotherapist. Program included stretching, mobilization, aerobic exercises on exercise bike, strengthening exercises for lower and upper extremities and back<br>2) Practice same exercises at home	1) 23<br>2) 22	1) 37.6 (11.3)<br>2) 34.3 (7.9)	1) 3 (13.0)<br>2) 4 (18.2)	**Not reported**
Hidding (1994) [The Netherlands][203]	RCT	Modified New York criteria<br>Exclusions: unable to engage in physical activity, total hip replacement, pregnancy, resting diastolic blood pressure >100mmhg, history of ischemic event, angina pectoris, heart failure, severe lung disease, diabetes, renal failure, chronic liver disease, malignancy, recent major surgery, mental retardation, serious emotional disorders	The intervention group from Hidding et al (1993)[204] were randomised to:<br>1) Group therapy once a week, 1 hour physical training, 1 hour sports, 1 hour hydrotherapy<br>p) Home exercises	1) 30<br>p) 34	1) 42.3 (9.5)<br>p) 44.3 (11.1)	1) 23%<br>p) 29%	**Government** (Health Insurance Executive Board)
Hidding (1993) [The Netherlands][204]	RCT	Aged <75 years, live <25km from assessment centre, no physical exercise therapy in last year, modified New York criteria, ≥1 of the following in past 3 months: pain, stiffness, functional limitations<br>Exclusions: Unable to engage in physical activity, total hip replacement, pregnancy, resting diastolic blood pressure >100mmhg, history of ischemic event, angina pectoris, heart failure, severe lung disease, diabetes, renal failure, chronic liver disease, malignancy, recent major surgery, mental retardation, serious emotional disorders	All patients received supervised individualised physical therapy<br>1) Received additional group physical therapy once a week<br>p) Waitlist control	1) 68<br>p) 76	1) 43.7 (10.4)<br>p) 41.5 (10.3)	1) 28%<br>p) 17%	**Government** (Health Insurance Executive Board)

axSpA = axial spondyloarthritis, ASAS = Assessment of Spondyloarthritis, BASDAI = Bath Ankylosing Spondylitis Disease Activity Index, DMARDs = disease modifying anti-rheumatic drugs, N = number, Pros. = prospective, RCT = randomised controlled trial, SD = standard deviation

Table – Aerobic + muscle strengthening exercises (axSpA), description of included studies

Author (date) [country]	Study design	Inclusion criteria	Exposure detail	N	Age years, mean (SD)	N (%) female	Funders
Kraag (1990) [Canada][205]	RCT	New York criteria, English language, stable steroids for past 3 months, DMARDs stable for 6 months, no surgery in next 4 months, not pregnant and using contraception<br>Exclusions: 10% loss of flexion in either hip joint, receiving contravening treatment	1) Home physiotherapy – therapeutic exercise aimed at increasing strength and endurance + education about posture<br>p) Waitlist control	1) 26<br>p) 27	1) 18-35: 13<br>≥36: 13<br>p) 18-35: 14<br>≥ 36: 13	1) 6 (23.1)<br>p) 5 (18.5)	**Government** (Health and Welfare Canada)
Levitova (2016) [Czech republic][206]	NRT	Modified New York criteria, stable treatment for 6 months, no steroids during study	1) Outpatient group physiotherapy 2x per week + home exercises – spinal exercises, posture correction, muscle stretching, Pilates<br>p) Non-exercise control matched for age and sex	1) 22<br>p) 14	1) 36.9 (1.2)<br>p) 36.7 (2.8)	1) 18.2%<br>p) 21.4%	**Government** (Ministry of Health Czech Republic)
Aytekin (2012) [Turkey][207]	NRT	Modified New York criteria<br>Exclusions: presences of prosthesis, hypertension, cardiovascular disease, chronic obstructive pulmonary disease, posteroanterior chest X-ray abnormalities	Home-based exercise regime including range of motion exercises, stretching, strengthening, posture and respiratory exercises – demonstrated by physiotherapist<br>1) Those who performed exercises ≥5x per week<br>p) Those who performed exercises <5x per week	1) 34<br>p) 32	1) 34.4 (9.5)<br>p) 35.8 (6.7)	1) 9 (26.5)<br>p) 5 (15.6)	**Not reported –** authors declared no conflict of interest
Viitanen (2001) [Finland][208]	NRT	ACR SPA criteria	3 weeks of intensive physiotherapy and exercise – swimming, group gym.<br>1) Control	1) 25<br>p) 18	1) 48 (9)<br>p) 18	Not reported	**Not reported**
Lubrano (2007) [Italy][209]	Single arm int.	Modified New York criteria<br>Exclusions: Complete ankylosing of the spine, previous admission for inpatients physiotherapy within 12 months, previous use of TNFi, use of DMARDs other than sulfasalazine or methotrexate within past 4 weeks, usage of >10mg prednisolone daily, variation of dosage of NSAIDs or prednisolone within 2 weeks of enrolment	Sessions supervised by physiotherapist – (i) warm-up followed by 30 mins strengthening, (ii) stretching exercises, (iii) endurance exercises, (iv) respiratory exercises	52	45.7 (10.0)	13 (25.0)	**Not reported –** authors declared no conflict of interest
Band (1997) [UK][210]	Single arm int.	Patients whose deterioration in clinical status is such that the patient would benefit from intensive inpatient treatment	Aims of intensive program: improve mobility, cardiorespiratory fitness, postural awareness, muscle strength, reduce pain and stiffness, increase understanding of disease, provide benefit of group setting	236	46.0 (10.9)	46 (19.5)	**Charity** (Arthritis and Rheumatism Council, National Ankylosing Spondylitis Society, Coates Trust, Pilkington Trust)
Viitanen (1995) [Finland][211]	Single arm int.	Modified New York criteria<br>Exclusions: history of psoriasis, chronic intestinal disorder (e.g. Crohn's disease or colitis ulcerosa), reactive arthritis of juvenile onset	Individual intensive program, pool exercise and group exercise – gym jogging, walking, heat and cold treatments, electrotherapy, massage	141	Men: 44.9 (8.9)<br>Women: 44.8 (9.7)	39 (27.7)	**Charity** (Sakari Sohlberg Foundation)

axSpA = axial spondyloarthritis, DMARDs = disease modifying anti-rheumatic drugs, N = number, NRT = non-randomised trial, Pros. = prospective, RCT = randomised controlled trial, SD = standard deviation, SPA = spondyloarthritis, UK = United Kingdom

Table – Aerobic + muscle strengthening exercises (axSpA), description of included studies

Author (date) [country]	Study design	Inclusion criteria	Exposure detail	N	Age years, mean (SD)	N (%) female	Funders
Kraag (1994) [Canada][212]	Single arm int.	New York criteria, English language, stable steroids for past 3 months, DMARDs stable for 6 months, no surgery in next 4 months, not pregnant and using contraception<br>Exclusions: 10% loss of flexion in either hip joint, receiving contravening treatment	Same intervention as Kraag (1990)[205] – original intervention group were followed up and provided support as needed, control group were given intervention	46	not reported	not reported	**Not reported**
Viitanen (1992) [Finland][213]	Single arm int.	New York criteria<br>Exclusions: Another disease, active peripheral arthritis	Inpatient physiotherapy course	505	43.0 (9.6)	143 (28.3)	**Charity** (Paivikki and Sakari Sohlberg Foundation)
Escalas (2016) [France][214]	Pros. cohort	Inflammatory back pain, aged >18 & <50 years, symptom duration >3 months & <3 years, met Calin or Berlin criteria<br>Exclusions: Definite diagnosis of non-spondyloarthritis back pain, conditions that might interfere with validity of informed consent and prevent compliance (e.g. alcoholism, history of psychiatric disorders), TNFi use	Self-reported number of physiotherapy sessions. Early physio defined as ≥8 sessions in first 6 months.	689	33.3 (8.6)	371 (53.8)	**Industry** (Pfizer)

axSpA = axial spondyloarthritis, DMARDs = disease modifying anti-rheumatic drugs, N = number, Pros. = prospective, SD = standard deviation, TNFi = Tumour Necrosis Factor Inhibitors

Supplementary table 65 – Results from reviews and interventional studies of aerobic + muscle strengthening exercise in axSpA

Table – Aerobic + muscle strengthening exercises (axSpA), results and quality assessment

Outcome (outcome measure)	Study (date) [study type]	Standardised result, SMD (95% CI) unless otherwise stated	Natural result	AMSTAR2 quality	Rand. Seq.	Alloc. Conc.	Blind. Part.	Blind. Asses.
Pain	Millner (2016) [MA][191]	Exercise vs control<br>SMD -0.42 (-0.74, -0.09)		Low				
	Liang (2015) [MA][192]	Exercise vs control<br>SMD -0.22 (-0.49, 0.06)		Moderate				
	Sharan (2017) [SR][195]		Relatively few studies reported improvements in pain	Critically low §				
	O'Dwyer (2014) [SR][180]		Two studies reported significantly lower pain compared to controls	Moderate				
	Rosu (2014) [RCT][199]	Pilates + extra exercises vs Pilates only at 48 weeks<br>SMD -0.90 (-1.32, -0.48)	Pain VAS, BL / 48 weeks, mean (SD)<br>Pilates + extra exercises: 36.46 (10.42) / 13.54 (7.85)<br>Pilates only: 34.79 (12.03) / 21.04 (8.81)		H/UC	H/UC	H/UC	H/UC
	Analay (2003) [RCT][202]	Group vs home exercise<br>SMD 0.07 (-0.52, 0.66)	Rest pain, BL / 6 weeks / 3 months, mean (SD)<br>Group exercise: 3.82 (3.4) / 3.3 (2.3) / 3.43 (2.5)<br>Home exercise: 3.13 (2.6) / 3.09 (3.6) / 3.18 (3.1)		H/UC	L	H/UC	L
	Hidding (1994) [RCT][203]		Pain VAS, change BL-9 months, mean<br>Exercise: -0.1<br>Control: -0.4		H/UC	H/UC	H/UC	L
	Hidding (1993) [RCT][204]		Pain VAS, change BL-9 months, mean<br>Exercise: 0.7<br>Control: 0.2<br>Mean difference in change: -0.43 (95% CI -1.24, 0.38)		H/UC	H/UC	H/UC	L
	Kraag (1990) [RCT][205]	Exercise vs control, change BL-4 months<br>SMD 0.39 (-1.50, 0.94)	Pain, Change BL-4 months, mean (SD)<br>Exercise: 5.2 (26.3)<br>Control: -5.2 (26.7)		H/UC	H/UC	H/UC	H/UC
	Aytekin (2012) [NRT][207]	Exercise ≥5x per week vs exercise <5x per week at 3 months<br>SMD 0.10 (-0.38, 0.58)	Pain VAS, BL / 3 months<br>Exercise ≥5x per week: 5.1 (2.1) / 4.1 (2.0)<br>Exercise <5x per week: 3.9 (2.3) / 3.9 (2.0)					
	Lubrano (2007) [Single arm int.][209]		Pain VAS, BL / 12 weeks, mean (SD)<br>76.6 (5.0) / 66.3 (6.3)					
	Kraag (1994) [Single arm int.][212]		Pain, 4 months / 8 months<br>Original exercise group: 27.9 (21.6) / 25.1 (21.1)<br>Original control group: 42.8 (25.8) / 42.2 (32.2)					

Alloc. Conc. = allocation concealment, AMSTAR2 = Assessing the Methodological Quality of Systematic Reviews, axSpA = axial spondyloarthritis, BL = baseline, Blind. Asses. = Blinded assessors, Blind. Part. = blinded participants, CI = confidence interval, H/UC = high / unclear risk of bias, L = low risk of bias, MD = mean difference, NRT = non-randomised trial, Rand. Seq. = random sequence generation, RCT = randomised controlled trial, SD = standard deviation, SMD = standardised mean difference, VAS = visual analogue scale

Table – Aerobic + muscle strengthening exercises (axSpA), results and quality assessment

Outcome (outcome measure)	Study (date) [study type]	Standardised result, SMD (95% CI) unless otherwise stated	Natural result	AMSTAR2 quality	Rand. Seq.	Alloc. Conc.	Blind. Part.	Blind. Asses.
Function	Pecourneau (2018) [MA][189]	Exercise vs control [BASFI]<br>All: SMD -0.72 (-1.03, -0.40)<br>Patients taking TNFi: SMD -0.81 (-1.25, -0.38)		Moderate				
	Chang (2016) [MA][190]	Specific exercise regimes vs standard exercise<br>SMD -0.39 (-0.58, -0.18)		Low				
	Millner (2016) [MA][191]	Exercise vs control<br>SMD -0.51 (-0.81, -0.21)		Low				
	Liang (2015) [MA][192]		BASFI, mean difference<br>MD -0.39 (-0.57, -0.20)	Moderate				
	Liang (2015) [MA][193]		BASFI, mean difference<br>MD -0.31 (-0.76, 0.15)	Moderate				
	Martins (2014) [MA][194]	Exercise vs normal physical activity<br>SMD -0.44 (-0.79, -0.09)		Moderate				
	Regel (2017) [SR][179]		One study[200] reported a moderate difference between the groups	Moderate				
	O'Dwyer (2014) [SR][180]		3 out of 7 studies favoured exercise over control for improving function	Moderate				
	Sveaas (2019) [RCT][196]	Exercise vs control at 3 months<br>SMD -0.81 (-1.22, -0.40)	BASFI, BL / 3 months, mean (SD)<br>Exercise: 2.9 (1.8) / 1.8 (1.4)<br>Control: 3.6 (2.1) / 3.2 (2.0)		L	L	H/UC	L
	Aydin (2016) [RCT][198]	Home vs hospital based exercise at 8 weeks<br>SMD 0.48 (-0.18, 1.13)	BASFI, BL / 8 weeks, mean (SD)<br>Home based exercise: 3.64 (2.87) / 3.78 (2.67)<br>Hospital based exercise: 3.16 (2.43) / 2.63 (2.07)		L	H/UC	H/UC	H/UC
	Rosu (2014) [RCT][199]	Pilates + extra exercises vs Pilates only at 48 weeks<br>SMD -0.93 (-1.35, -0.51)	BASFI. BL / 48 weeks, mean (SD)<br>Pilates + extra exercises: 3.56 (1.83) / 1.50 (1.11)<br>Pilates only: 3.42 (1.94) / 2.76 (1.56)		H/UC	H/UC	H/UC	H/UC
	Sveaas (2014) [RCT][200]	Exercise vs control at 3 months<br>SMD -1.11 (-1.99, -0.24)	BASFI, BL / 3 months, mean (SD)<br>Exercise: 2.6 (2.2) / 1.5 (1.5)<br>Control: 3.1 (1.6) / 3.1 (1.4)		L	L	H/UC	L

Alloc. Conc. = allocation concealment, AMSTAR2 = Assessing the Methodological Quality of Systematic Reviews, axSpA = axial spondyloarthritis, BASFI = Bath Ankylosing Spondylitis Functional Index, BL = baseline, Blind. Asses. = Blinded assessors, Blind. Part. = blinded participants, CI = confidence interval, H/UC = high / unclear risk of bias, HAQ-S = Health Assessment Questionnaire for the Spondyloarthropaties, L = low risk of bias, MA = meta-analysis, MD = mean difference, Rand. Seq. = random sequence generation, RCT = randomised controlled trial, SD = standard deviation, SMD = standardised mean difference

Table – Aerobic + muscle strengthening exercises (axSpA), results and quality assessment

Outcome (outcome measure)	Study (date) [study type]	Standardised result, SMD (95% CI) unless otherwise stated	Natural result	AMSTAR2 quality	Rand. Seq.	Alloc. Conc.	Blind. Part.	Blind. Asses.
Function	Kjeken (2013) [RCT][201]	Exercise vs control at 12 months<br>SMD -0.03 (-0.44, 0.37)	BASFI, BL / 12 months, mean (SD †)<br>Exercise: 38.6 (17.5) / 38.0 (19.0)<br>Control: 42.4 (20.0) / 38.6 (18.2)		L	L	H/UC	L
	Analay (2003) [RCT][202]	Group vs home exercise<br>SMD -0.39 (-0.98, 0.20)	BASFI, BL / 6 weeks / 3 months, mean (SD)<br>Group exercise: 26.34 (20.10) / 20.0 (16.76) / 22.0 (17.15)<br>Home exercise: 27.59 (17.82) / 27.31 (20.42) / 26.13 (17.20)		H/UC	L	H/UC	L
	Hidding (1994) [RCT][203]		HAQ-S, change BL-9 months, mean<br>Exercise: 0.01<br>Control: -0.08		H/UC	H/UC	H/UC	L
	Hidding (1993) [RCT][204]		HAQ-S, change BL-9 months, mean<br>Exercise: -0.02<br>Control: 0.03<br>Mean difference in change: 0.05 (95% CI 0.0, 0.11)		H/UC	H/UC	H/UC	L
	Kraag (1990) [RCT][205]	Exercise vs control, change BL-4 months<br>SMD 1.68 (1.05, 2.31)	Toronto Activities of Daily Living, Change BL-4 months, mean (SD)<br>Exercise: 3.92 (2.94)<br>Control: -0.19 (1.86)		H/UC	H/UC	H/UC	H/UC
	Bespoke meta-analysis including[196;200;201;205]	Exercise vs control<br>SMD -0.87 (-1.58, -0.16), I^2 85.7%						

† Mean (SD) estimated from median (range) using published formula[87]

Alloc. Conc. = allocation concealment, AMSTAR2 = Assessing the Methodological Quality of Systematic Reviews, axSpA = axial spondyloarthritis, BASFI = Bath Ankylosing Spondylitis Functional Index, BL = baseline, Blind. Asses. = Blinded assessors, Blind. Part. = blinded participants, CI = confidence interval, H/UC = high / unclear risk of bias, HAQ-S = Health Assessment Questionnaire for the Spondyloarthropaties, L = low risk of bias, NRT = non-randomised trial, Rand. Seq. = random sequence generation, RCT = randomised controlled trial, SD = standard deviation, SMD = standardised mean difference

Table – Aerobic + muscle strengthening exercises (axSpA), results and quality assessment

Outcome (outcome measure)	Study (date) [study type]	Standardised result, SMD (95% CI) unless otherwise stated	Natural result	AMSTAR2 quality	Rand. Seq.	Alloc. Conc.	Blind. Part.	Blind. Asses.
Function	Levitova (2016) [NRT][206]		BASFI, Change BL-6 months, mean (SD)<br>Exercise: 0.92 (0.17) / 0.93 (0.18)<br>Control: not reported					
	Aytekin (2012) [NRT][207]	Exercise ≥5x per week vs exercise <5x per week at 3 months<br>SMD -0.43 (-0.92, 0.06)	BASFI, BL / 3 months<br>Exercise ≥5x per week: 2.54 (2.26) / 2.05 (2.14)<br>Exercise <5x per week: 2.90 (2.30) / 2.99 (2.26)					
	Viitanen (2001) [NRT][208]		BASFI, change BL – 3 weeks, mean (SD ‡)<br>Exercise: -0.5 (1.5)<br>Control: 0.5 (1.2)<br>Dougados function index, change BL – 3 weeks, mean (SD ‡)<br>Exercise: -0.4 (4.6)<br>Control: 0.0 (1.5)<br>HAQ-S, change BL – 3 weeks, mean (SD ‡)<br>Exercise: -0.17 (0.92)<br>Control: -0.06 (0.22)					
	Lubrano (2007) [Single arm int.][209]		BASFI, BL / 12 weeks, mean (SD)<br>67.1 (7.9) / 57.9 (7.2)					
	Kraag (1994) [Single arm int.][212]		Toronto Activities of Daily Living, 4 months / 8 months<br>Original exercise group: 19.9 (3.9) / 2.3 (4.2)<br>Original control group: 16.6 (2.8) / 0.8 (2.4)					
	Band (1997) [Single arm int.][210]		BASFI, mean change BL-2 weeks<br>-1.27					

‡ SD calculated from 95% CI in paper

Alloc. Conc. = allocation concealment, AMSTAR2 = Assessing the Methodological Quality of Systematic Reviews, axSpA = axial spondyloarthritis, BASFI = Bath Ankylosing Spondylitis Functional Index, BL = baseline, Blind. Asses. = Blinded assessors, Blind. Part. = blinded participants, CI = confidence interval, H/UC = high / unclear risk of bias, HAQ-S = Health Assessment Questionnaire for the Spondyloarthropaties, L = low risk of bias, NRT = non-randomised trial, Rand. Seq. = random sequence generation, SD = standard deviation, SMD = standardised mean difference

Table – Aerobic + muscle strengthening exercises (axSpA), results and quality assessment

Outcome (outcome measure)	Study (date) [study type]	Standardised result, SMD (95% CI) unless otherwise stated	Natural result	AMSTAR2 quality	Rand. Seq.	Alloc. Conc.	Blind. Part.	Blind. Asses.
Disease activity	Pecourneau (2018) [MA][189]	Exercise vs control [BASDAI]<br>All: SMD -0.90 (-1.52, -0.27)<br>Patients taking TNFi: SMD -1.37 (-1.90, -0.84)		Moderate				
	Chang (2016) [MA][190]	Specific exercise regimes vs standard exercise<br>SMD -0.41 (-0.86, 0.05)		Low				
	Millner (2016) [MA][191]	Exercise vs control<br>SMD -0.47 (-0.84, -0.09)		Low				
	Liang (2015) [MA][192]		BASDAI, mean difference<br>MD -0.50 (-0.99, -0.02)	Moderate				
	Liang (2015) [MA][193]		BASDAI, mean difference<br>MD -0.58 (-1.10, -0.06)	Moderate				
	Martins (2014) [MA][194]	Exercise vs normal physical activity<br>SMD -0.58 (-0.94, -0.22)		Moderate				
	Regel (2017) [SR][179]		One study[200] reported a large difference between the groups	Moderate				
	O'Dwyer (2014) [SR][180]		2 out of 5 studies favoured exercise over control for improving disease activity measured using BASDAI	Moderate				
	Sveaas (2019) [RCT][196]	Exercise vs control at 3 months<br>ASDAS: SMD -1.00 (-1.42, -0.58)<br>BASDAI: SMD -0.97 (-1.38, -0.55)	ASDAS, BL / 3 months, mean (SD)<br>Exercise: 2.6 (0.8) / 1.9 (0.7)<br>Control: 2.7 (0.6) / 2.6 (0.7)<br>BASDAI, BL / 3 months, mean (SD)<br>Exercise: 4.9 (1.6) / 3.3 (1.6)<br>Control: 5.3 (1.5) / 4.8 (1.5)		L	L	H/UC	L
	Aydin (2016) [RCT][198]	Home vs hospital based exercise at 8 weeks<br>SMD 0.52 (-0.14, 1.17)	BASDAI, BL / 8 weeks, mean (SD)<br>Home based exercise: 5.02 (2.43) / 4.66 (2.02)<br>Hospital based exercise: 4.15 (1.79) / 3.66 (1.84)		L	H/UC	H/UC	H/UC

Alloc. Conc. = allocation concealment, AMSTAR2 = Assessing the Methodological Quality of Systematic Reviews, axSpA = axial spondyloarthritis, ASDAS = Ankylosing Spondylitis Disease Activity Score, BASDAI = Bath Ankylosing Spondylitis Disease Activity Index, BL = baseline, Blind. Asses. = Blinded assessors, Blind. Part. = blinded participants, CI = confidence interval, H/UC = high / unclear risk of bias, L = low risk of bias, MD = mean difference, Rand. Seq. = random sequence generation, RCT = randomised controlled trial, SD = standard deviation, SMD = standardised mean difference

Table – Aerobic + muscle strengthening exercises (axSpA), results and quality assessment

Outcome (outcome measure)	Study (date) [study type]	Standardised result, SMD (95% CI) unless otherwise stated	Natural result	AMSTAR2 quality	Rand. Seq.	Alloc. Conc.	Blind. Part.	Blind. Asses.
Disease activity	Rosu (2014) [RCT][199]	Pilates + extra exercises vs Pilates only at 48 weeks<br>SMD -1.55 (-2.01, -1.09)	BASDAI. BL / 48 weeks, mean (SD)<br>Pilates + extra exercises: 5.41 (1.95) / 2.10 (0.82)<br>Pilates only: 5.29 (1.96) / 4.13 (1.66)		H/UC	H/UC	H/UC	H/UC
	Sveaas (2014) [RCT][200]	Exercise vs control at 3 months<br>ASDAS: SMD -0.95 (-1.81, -0.09)<br>BASDAI: SMD -0.45 (-1.27, 0.37)	ASDAS, BL / 3 months, mean (SD)<br>Exercise: 2.3 (0.6) / 1.8 (0.9)<br>Control: 2.7 (0.8) / 2.6 (0.8)<br>BASDAI, BL / 3 months, mean (SD)<br>Exercise: 5.3 (1.4) / 3.3 (2.0)<br>Control: 5.3 (1.3) / 4.2 (2.0)		L	L	H/UC	L
	Kjeken (2013) [RCT][201]	Exercise vs control at 12 months<br>SMD -0.21 (-0.62, 0.19)	BASDAI, BL / 12 months, mean (SD †)<br>Exercise: 57.8 (10.6) / 49.6 (22.8)<br>Control: 56.9 (12.5) / 54.5 (22.9)		L	L	H/UC	L
	Bespoke meta-analysis including[196;200;201]	Exercise vs control<br>SMD -0.56 (-1.09, -0.02), I^2 69.6%						
	Levitova (2016) [NRT][206]	Exercise vs control, change BL-6 months<br>ASDAS SMD -0.16 (-0.83, 0.51)<br>BASDAI SMD -0.34 (-1.02, 0.34)	ASDAS, Change BL-6 months, mean (SD)<br>Exercise: -0.24 (0.68)<br>Control: -0.13 (0.72)<br>BASDAI, Change BL-6 months, mean (SD)<br>Exercise: -0.32 (1.34)<br>Control: 0.12 (1.22)					
	Aytekin (2012) [NRT][207]	Exercise ≥5x per week vs exercise <5x per week at 3 months<br>SMD -0.14 (-0.63, 0.34)	BASDAI, BL / 3 months<br>Exercise ≥5x per week: 4.44 (2.07) / 3.77 (1.98)<br>Exercise <5x per week: 3.98 (2.19) / 4.07 (2.21)					
	Viitanen (2001) [NRT][208]		BASDAI, change BL – 3 weeks, mean (SD ‡)<br>Exercise: 4 (17.9)<br>Control: 5 (11.9)					
	Lubrano (2007) [Single arm int.][209]		BASDAI, BL / 12 weeks, mean (SD)<br>65.9 (5.3) / 56.4 (5.9)					
	Band (1997) [Single arm int.][210]		BASDAI, mean change BL-2 weeks<br>-0.84					

† Mean (SD) estimated from median (range) using published formula[87]

‡ SD calculated from 95% CI in paper

Alloc. Conc. = allocation concealment, AMSTAR2 = Assessing the Methodological Quality of Systematic Reviews, axSpA = axial spondyloarthritis, ASDAS = Ankylosing Spondylitis Disease Activity Score, BASDAI = Bath Ankylosing Spondylitis Disease Activity Index, BL = baseline, Blind. Asses. = Blinded assessors, Blind. Part. = blinded participants, CI = confidence interval, H/UC = high / unclear risk of bias, L = low risk of bias, MD = mean difference, NRT = non-randomised trial, Rand. Seq. = random sequence generation, RCT = randomised controlled trial, SD = standard deviation, SMD = standardised mean difference

Table – Aerobic + muscle strengthening exercises (axSpA), results and quality assessment

Outcome (outcome measure)	Study (date) [study type]	Standardised result, SMD (95% CI) unless otherwise stated	Natural result	AMSTAR2 quality	Rand. Seq.	Alloc. Conc.	Blind. Part.	Blind. Asses.
Spinal mobility	Chang (2016) [MA][190]	Specific exercise regimes vs standard exercise<br>SMD -0.48 (-1.61, 0.64)		Low				
	Liang (2015) [MA][193]		BASMI, mean difference<br>MD -0.99 (-1.61, -0.38)	Moderate				
	Martins (2014) [MA][194]		Exercise vs normal physical activity<br>MD -0.51 (-0.95, -0.08)	Moderate				
	Regel (2017) [SR][179]		One study[200] reported a no difference between the groups	Moderate				
	O'Dwyer (2014) [SR][180]		BASMI scores were lower after rehabilitation programmes but not Pilates	Moderate				
	Sveaas (2019) [RCT][196]	Exercise vs control at 3 months<br>SMD 0.00 (-0.39, 0.39)<br>[Significant difference after adjusting for centre] and baseline values]	BASMI, BL / 3 months, mean (SD)<br>Exercise: 2.9 (1.3) / 2.5 (1.2)<br>Control: 2.6 (1.3) / 2.5 (1.4)		L	L	H/UC	L
	Aydin (2016) [RCT][198]	Home vs hospital based exercise at 8 weeks<br>SMD 0.57 (-0.09, 1.23)	BASMI, BL / 8 weeks, mean (SD)<br>Home based exercise: 2.42 (1.50) / 2.52 (1.34)<br>Hospital based exercise: 2.38 (1.19) / 1.83 (1.04)		L	H/UC	H/UC	H/UC
	Rosu (2014) [RCT][199]	Pilates + extra exercises vs Pilates only at 48 weeks<br>SMD -2.73 (-3.29, -2.17)	BASMI. BL / 48 weeks, mean (SD)<br>Pilates + extra exercises: 3.73 (0.45) / 1.19 (0.84)<br>Pilates only: 3.3 (0.45) / 3.02 (0.44)		H/UC	H/UC	H/UC	H/UC
	Sveaas (2014) [RCT][200]	Exercise vs control at 3 months<br>SMD -0.52 (-1.35, 0.30)	BASMI, BL / 3 months, mean (SD)<br>Exercise: 2.3 (1.5) / 2.0 (1.6)<br>Control: 3.0 (1.8) / 2.9 (1.8)		L	L	H/UC	L
	Bespoke meta-analysis including[196;200]	Exercise vs control<br>SMD -0.13 (-0.57, 0.31), I^2 20.4%						
	Levitova (2016) [NRT][206]		BASMI, Change BL-6 months, mean (SD)<br>Exercise: 1.43 (0.24) / 0.82 (0.23)					
	Band (1997) [Single arm int.][210]		BASMI, mean change BL-2 weeks<br>-0.97					

Alloc. Conc. = allocation concealment, AMSTAR2 = Assessing the Methodological Quality of Systematic Reviews, axSpA = axial spondyloarthritis, BASMI = Bath Ankylosing Spondylitis Metrology Index, BL = baseline, Blind. Asses. = Blinded assessors, Blind. Part. = blinded participants, CI = confidence interval, H/UC = high / unclear risk of bias, L = low risk of bias, MD = mean difference, NRT = non-randomised trial, Rand. Seq. = random sequence generation, RCT = randomised controlled trial, SD = standard deviation, SMD = standardised mean difference

Table – Aerobic + muscle strengthening exercises (axSpA), results and quality assessment

Outcome (outcome measure)	Study (date) [study type]	Standardised result, SMD (95% CI) unless otherwise stated	Natural result	AMSTAR2 quality	Rand. Seq.	Alloc. Conc.	Blind. Part.	Blind. Asses.
Stiffness	O'Dwyer (2014) [SR][180]		Two studies reported significantly lower stiffness compared to controls	Moderate				
	Analay (2003) [RCT][202]	Group vs home exercise<br>SMD -0.33 (-0.92, 0.26)	Morning stiffness, BL / 6 weeks / 3 months, mean (SD)<br>Group exercise: 38.65 (60.32) / 20.87 (32.34) / 24.04 (36.24)<br>Home exercise: 36.59 (51.44) / 37 (62.01) / 35.54 (36.77)		H/UC	L	H/UC	L
	Hidding (1994) [RCT][203]		Stiffness VAS, change BL-9 months, mean<br>Exercise: -0.6<br>Control: -0.2		H/UC	H/UC	H/UC	L
	Hidding (1993) [RCT][204]		Stiffness VAS, change BL-9 months, mean<br>Exercise: 0.2<br>Control: -0.1<br>Mean difference in change: -0.31 (95% CI -0.92, 0.29)		H/UC	H/UC	H/UC	L
	Aytekin (2012) [NRT][207]	Exercise ≥5x per week vs exercise <5x per week at 3 months<br>SMD 0.01 (-0.47, 0.49)	Morning stiffness, BL / 3 months<br>Exercise ≥5x per week: 55.15 (70.94) / 39.11 (65.86)<br>Exercise <5x per week: 32.66 (32.40) / 38.59 (34.43)					
	Viitanen (2001) [NRT][208]		Stiffness, change BL – 3 weeks, mean (SD ‡)<br>Exercise: 0.2 (21.7)<br>Control: 6 (19.5)					
Joint activity	Hidding (1994) [RCT][203]		Articular index, change BL-9 months, mean<br>Exercise: 0.1<br>Control: -1.6		H/UC	H/UC	H/UC	L
	Hidding (1993) [RCT][204]		Articular index, change BL-9 months, mean<br>Exercise: -0.3<br>Control: 0.3<br>Mean difference in change: 0.60 (95% CI -0.52, 1.72)		H/UC	H/UC	H/UC	L

‡ SD calculated from 95% CI in paper

Alloc. Conc. = allocation concealment, AMSTAR2 = Assessing the Methodological Quality of Systematic Reviews, axSpA = axial spondyloarthritis, BL = baseline, Blind. Asses. = Blinded assessors, Blind. Part. = blinded participants, CI = confidence interval, H/UC = high / unclear risk of bias, L = low risk of bias, NRT = non-randomised trial, Rand. Seq. = random sequence generation, RCT = randomised controlled trial, SD = standard deviation, SMD = standardised mean difference

Table – Aerobic + muscle strengthening exercises (axSpA), results and quality assessment

Outcome (outcome measure)	Study (date) [study type]	Standardised result, SMD (95% CI) unless otherwise stated	Natural result	AMSTAR2 quality	Rand. Seq.	Alloc. Conc.	Blind. Part.	Blind. Asses.
Patient global	Sveaas (2019) [RCT][196]	Exercise vs control at 3 months<br>SMD -0.91 (-1.32, -0.50)	Patient global, BL / 3 months, mean (SD)<br>Exercise: 4.7 (2.0) / 2.9 (2.3)<br>Control: 5.3 (2.0) / 4.9 (2.1)		L	L	H/UC	L
	Aydin (2016) [RCT][198]	Home vs hospital based exercise at 8 weeks<br>SMD 0.18 (-0.46, 0.83)	BAS-G, BL / 8 weeks, mean (SD)<br>Home based exercise: 5.10 (2.11) / 4.80 (1.67)<br>Hospital based exercise: 4.58 (1.88) / 4.45 (2.14)		L	H/UC	H/UC	H/UC
	Kjeken (2013) [RCT][201]	Exercise vs control at 12 months<br>SMD -0.38 (-0.79, 0.03)	BAS-G, BL / 12 months, mean (SD †)<br>Exercise: 56.2 (17.8) / 41.7 (23.5)<br>Control: 57.5 (18.8) / 50.5 (22.7)		L	L	H/UC	L
	Viitanen (2001) [NRT][208]		BAS-G, change BL – 3 weeks, mean (SD ‡)<br>Exercise: 1 (20.4)<br>Control: 4 (21.6)					
	Lubrano (2007) [Single arm int.][209]		Patients global VAS, BL / 12 weeks, mean (SD)<br>72.1 (6.8) / 59.7 (6.6)					
	Band (1997) [Single arm int.][210]		BAS-G, mean change BL-2 weeks<br>-1.38					
QoL	O'Dwyer (2014) [SR][180]		QoL scores were lower after rehabilitation programmes but not Pilates	Moderate				
	Aydin (2016) [RCT][198]	Home vs hospital based exercise at 8 weeks<br>SMD 0.58 (-0.08, 1.23)	ASQOL, BL / 8 weeks, mean (SD)<br>Home based exercise: 9.63 (5.41) / 9.00 (5.06)<br>Hospital based exercise: 7.11 (4.33) / 6.22 (4.59)		L	H/UC	H/UC	H/UC
	Aytekin (2012) [NRT][207]	Exercise ≥5x per week vs exercise <5x per week at 3 months<br>SMD -0.50 (-0.99, -0.01)	ASQOL, BL / 3 months<br>Exercise ≥5x per week: 9.56 (5.21) / 7.29 (4.6)<br>Exercise <5x per week: 9.34 (5.97) / 9.96 (6.1)					
Fatigue	Sharan (2017) [SR][195]		Relatively few studies reported improvements in pain	Critically low §				
	Sveaas (2019) [RCT][196]	Exercise vs control at 3 months<br>SMD -0.80 (-1.21, -0.39)	BASDAI question 1, BL / 3 months, mean (SD)<br>Exercise: 5.8 (1.8) / 3.8 (2.1)<br>Control: 6.1 (1.9) / 5.4 (1.9)		L	L	H/UC	L
	Sveaas (2018) [Norway][197]	Exercise vs control at 3 months<br>SMD -0.86 (-1.71, -0.01)	BASDAI question 1, BL / 3 months, mean (SD)<br>Exercise: 6.8 (1.5) / 3.7 (2.2)<br>Control: 6.3 (2.1) / 5.8 (2.6)		L	L	H/UC	H/UC
	Bespoke meta-analysis including[196;197]	Exercise vs control<br>SMD -0.81 (-1.18, -0.44), I^2 0%						

† Mean (SD) estimated from median (range) using published formula[87]; ‡ SD calculated from 95% CI in paper;

Alloc. Conc. = allocation concealment, AMSTAR2 = Assessing the Methodological Quality of Systematic Reviews, ASQOL = Ankylosing Spondylitis Quality of Life, axSpA = axial spondyloarthritis, BASDAI = Bath Ankylosing Spondylitis Disease Activity Index, BAS-G = Bath Ankylosing Spondylitis Global Score, BL = baseline, Blind. Asses. = Blinded assessors, Blind. Part. = blinded participants, CI = confidence interval, H/UC = high / unclear risk of bias, L = low risk of bias, MD = mean difference, NRT = non-randomised trial, QoL = quality of life, Rand. Seq. = random sequence generation, RCT = randomised controlled trial, SD = standard deviation, SMD = standardised mean difference, VAS = visual analogue scale

Table – Aerobic + muscle strengthening exercises (axSpA), results and quality assessment

Outcome (outcome measure)	Study (date) [study type]	Standardised result, SMD (95% CI) unless otherwise stated	Natural result	AMSTAR2 quality	Rand. Seq.	Alloc. Conc.	Blind. Part.	Blind. Asses.
Emotional distress	Sveaas (2018) [Norway][197]	Exercise vs control at 3 months<br>SMD -1.05 (-1.92, -0.18)	Emotional distress (GHQ), BL / 3 months, mean (SD)<br>Exercise: 20.0 (3.4) / 13.9 (6.1)<br>Control: 19.1 (3.9) / 19.1 (4.0)		L	L	H/UC	H/UC
Anxiety	Aydin (2016) [RCT][198]	Home vs hospital based exercise at 8 weeks<br>SMD 0.55 (-0.11, 1.21)	HADS - anxiety, BL / 8 weeks, mean (SD)<br>Home based exercise: 8.84 (4.08) / 8.63 (4.23)<br>Hospital based exercise: 8.22 (4.90) / 6.50 (3.45)		L	H/UC	H/UC	H/UC
Depression	Liang (2015) [MA][192]		Depression, mean difference<br>MD -2.31 (-3.33, -1.30)	Moderate				
	Sharan (2017) [SR][195]		Exercise has been reported to reduce depression	Critically low §				
	Aydin (2016) [RCT][198]	Home vs hospital based exercise at 8 weeks<br>SMD 0.65 (-0.01, 1.32)	HADS - depression, BL / 8 weeks, mean (SD)<br>Home based exercise: 9.21 (4.57) / 9.47 (5.61)<br>Hospital based exercise: 7.66 (4.25) / 6.38 (3.58)		L	H/UC	H/UC	H/UC
	Analay (2003) [RCT][202]	Group vs home exercise<br>SMD -0.38 (-0.97, 0.21)	BDI, BL / 6 weeks / 3 months, mean (SD)<br>Group exercise: 5.52 (4.56) / 3.95 (3.21) / 5.13 (6.34)<br>Home exercise: 6.31 (4.72) / 5.90 (6.62) / 6.77 (6.41)		H/UC	L	H/UC	L
Self-efficacy	Liang (2015) [MA][192]	Exercise vs control<br>SMD 0.07 (-0.25, 0.38)		Moderate				

Alloc. Conc. = allocation concealment, AMSTAR2 = Assessing the Methodological Quality of Systematic Reviews, axSpA = axial spondyloarthritis, BDI = Beck Depression Inventory, BL = baseline, Blind. Asses. = Blinded assessors, Blind. Part. = blinded participants, CI = confidence interval, GHQ = General Health Questionnaire, H/UC = high / unclear risk of bias, HADS = Hospital Anxiety and Depression Scale, L = low risk of bias, MD = mean difference, Rand. Seq. = random sequence generation, RCT = randomised controlled trial, SD = standard deviation, SMD = standardised mean difference

Table – Aerobic + muscle strengthening exercises (axSpA), results and quality assessment

Outcome (outcome measure)	Study (date) [study type]	Standardised result, SMD (95% CI) unless otherwise stated	Natural result	AMSTAR2 quality	Rand. Seq.	Alloc. Conc.	Blind. Part.	Blind. Asses.
CRP	Sveaas (2019) [RCT][196]	Exercise vs control at 3 months<br>SMD -0.29 (-0.68, 0.11)	CRP, BL / 3 months, mean (SD †)<br>Exercise: 8.3 (6.0) / 4.0 (2.7)<br>Control: 8.5 (6.2) / 4.8 (2.9)		L	L	H/UC	L
	Aydin (2016) [RCT][198]	Home vs hospital based exercise at 8 weeks<br>SMD -0.48 (-1.14, 0.17)	CRP, BL / 8 weeks, mean (SD)<br>Home based exercise: 0.44 (0.54) / 0.32 (0.30)<br>Hospital based exercise: 1.25 (2.80) / 0.90 (1.69)		L	H/UC	H/UC	H/UC
	Sveaas (2014) [RCT][200]	Exercise vs control at 3 months<br>SMD -0.34 (-1.16, 0.48)	CRP, BL / 3 months, mean (SD †)<br>Exercise: 3.0 (2.6) / 3.8 (3.6)<br>Control: 7.0 (6.4) / 5.0 (3.5)		L	L	H/UC	L
	Bespoke meta-analysis including[196;200]	Exercise vs control<br>SMD -0.30 (-0.65, 0.06), I^2 20.4%						
	Levitova (2016) [NRT][206]	Exercise vs control, change BL-6 months<br>SMD 0.21 (-0.46, 0.89)	CRP, Change BL-6 months, mean (SD)<br>Exercise: -1.52 (6.02)<br>Control: -2.76 (5.49)					
ESR	Sveaas (2019) [RCT][196]	Exercise vs control at 3 months<br>SMD 0.98 (0.56, 1.39)	ESR, BL / 3 months, mean (SD †)<br>Exercise: 21.3 (14.5) / 24.0 (17.4)<br>Control: 11.5 (6.2) / 11.3 (6.0)		L	L	H/UC	L
	Aydin (2016) [RCT][198]	Home vs hospital based exercise at 8 weeks<br>SMD -0.69 (-1.35, -0.02)	ESR, BL / 8 weeks, mean (SD)<br>Home based exercise: 21.52 (12.98) / 17.47 (8.59)<br>Hospital based exercise: 30.38 (21.74) / 26.38 (16.42)		L	H/UC	H/UC	H/UC
	Sveaas (2014) [RCT][200]	Exercise vs control at 3 months<br>SMD -0.27 (-1.09, 0.54)	ESR, BL / 3 months, mean (SD †)<br>Exercise: 15.8 (12.6) / 12.3 (6.8)<br>Control: 9.3 (6.7) / 15.3 (13.2)		L	L	H/UC	L

† Mean (SD) estimated from median (range) using published formula[87]

Alloc. Conc. = allocation concealment, AMSTAR2 = Assessing the Methodological Quality of Systematic Reviews, axSpA = axial spondyloarthritis, BL = baseline, Blind. Asses. = Blinded assessors, Blind. Part. = blinded participants, CI = confidence interval, CRP = C-reactive protein, ESR = erythrocyte sedimentation rate, H/UC = high / unclear risk of bias, L = low risk of bias, MD = mean difference, Rand. Seq. = random sequence generation, RCT = randomised controlled trial, SD = standard deviation, SMD = standardised mean difference

Table – Aerobic + muscle strengthening exercises (axSpA), results and quality assessment

Outcome (outcome measure)	Study (date) [study type]	Standardised result, SMD (95% CI) unless otherwise stated	Natural result	AMSTAR2 quality	Rand. Seq.	Alloc. Conc.	Blind. Part.	Blind. Asses.
Spinal flexion	Millner (2016) [MA][191]	Exercise vs control<br>SMD 0.35 (0.02, 0.67)		Low				
	Rosu (2014) [RCT][199]	Pilates + extra exercises vs Pilates only at 48 weeks<br>SMD 1.65 (1.18, 2.11)	Schober's test. BL / 48 weeks, mean (SD)<br>Pilates + extra exercises: 2.71 (0.76) / 4.56 (0.56)<br>Pilates only: 2.83 (0.77) / 3.48 (0.74)		H/UC	H/UC	H/UC	H/UC
	Kraag (1990) [RCT][205]	Exercise vs control, change BL-4 months<br>SMD 0.04 (-0.50, 0.57)	Schober, Change BL-4 months, mean (SD)<br>Exercise: -2.4 (6.7)<br>Control: -2.7 (9.9)		H/UC	H/UC	H/UC	H/UC
	Lubrano (2007) [Single arm int.][209]		Schober, BL / 12 weeks, mean (SD)<br>1.9 (0.6) / 2.2 (0.6)					
	Viitanen (1995) [Single arm int.][211]		Schober, BL mean(SD) / 3 weeks (post int.) mean (SD) / 12 months change from BL (95% CI)<br>3.3 (1.6) / 3.6 (1.6) / -0.1 (-0.2, 0.1)					
	Kraag (1994) [Single arm int.][212]		Schober, 4 months / 8 months<br>Original exercise group: 13.4 (1.6) / 13.7 (1.5)<br>Original control group: 13.1 (1.5) / 13.2 (1.3)					
	Viitanen (1992) [Finland][213]		Schober, BL / after intervention, mean (SD)<br>Men: 2.86 (1.54) / 3.26 (1.57)<br>Women: 3.20 (1.38) / 3.53 (1.35)					

Alloc. Conc. = allocation concealment, AMSTAR2 = Assessing the Methodological Quality of Systematic Reviews, axSpA = axial spondyloarthritis, BL = baseline, Blind. Asses. = Blinded assessors, Blind. Part. = blinded participants, CI = confidence interval, H/UC = high / unclear risk of bias, L = low risk of bias, MD = mean difference, NRT = non-randomised trial, Rand. Seq. = random sequence generation, RCT = randomised controlled trial, SD = standard deviation, SMD = standardised mean difference

Supplementary table 66 – Results from observational studies of aerobic + muscle strengthening exercise in axSpA

Table – Aerobic + muscle strengthening exercise (axSpA), results and quality assessment – observational studies

Outcome (outcome measure)	Study (date) [study type]	Standardised result, SMD (95% CI) unless otherwise stated	Natural result	Study Pop.	Attr.	Prog. Meas.	Outc. Meas.	Conf.	Stats.
Function	Escalas (2016) [Pros. Obs][214]		20% improvement in BASFI over follow-up, RR (95% CI), reference = no physio<br>Early physio = 1.15 (0.91, 1.45) [fully adjusted]<br>50% improvement in BASFI over follow-up, RR (95% CI), reference = no physio<br>Early physio = 1.20 (0.87, 1.66) [fully adjusted]	L	L	M	L	L	L

Attr. = attrition, axSpA = axial spondyloarthritis, BASFI = Bath Ankylosing Spondylitis Functional Index, CI = confidence interval, Conf. = confounding, L = low risk of bias, M = moderate risk of bias, Outc. Meas = outcome measurement, Prog. Meas. = prognostic factor measurement, Pros. Obs. = prospective observational, Stats. = statistical analysis, Study Pop. = study population

Supplementary table 67 – Description of reviews of studies of aquatic exercise in axSpA

Table – Aquatic exercises (axSpA), description of reviews

Authors (date)	Review type	Study type included	Exposure detail	Number of studies included	Funders
Regel (2017)[179]	SR	RCTs	Aerobic exercises including walking and rehabilitation	1	**Professional bodies** (European League Against Rheumatism, Assessment of Spondyloarthritis international Society)
Sharan (2017)[195]	SR	RCTs, reviews	Studies of aerobic and strengthening included	30	**Not reported –** Authors declared no conflicts of interest
Zao (2017)[215]	SR	5	Aquatic exercises	5	**Not reported** – Authors declared no conflicts of interest

axSpA = axial spondyloarthritis, RCT = randomised controlled trial, SR = systematic review

Supplementary table 68 – Description of original studies of aquatic exercise in axSpA

Table – Aquatic exercises (axSpA), description of included studies

Author (date) [country]	Study design	Inclusion criteria	Exposure detail	N	Age years, mean (SD)	N (%) female	Funders
Dundar (2014) [Turkey][216]	RCT	New York Criteria<br>Exclusions: prosthesis, hypertension, cardiovascular disease, chronic obstructive pulmonary disease, exercising regular for past 3 months	1) Aquatic exercise – 5x per week. Started with poolside range of motion exercises then 40 mins aquatic exercise in pool and the 5 min cool down<br>2) Home based land exercises	1) 35<br>2) 34	1) 42.3 (11.3)<br>2) 43.1 (11.7)	1) 5 (14.3)<br>2) 6 (17.6)	**Not reported** – Authors declared no conflict of interest
Karapolat (2009) [Turkey][217]	RCT	Modified New York criteria, aged 18=75 years, knew how to swim, able to understand program<br>Exclusions: Inability / unwillingness to participate, systemic organic involvement, active peripheral joint involvement, severe comorbidity affecting lung, heart, liver kidneys, receiving DMARDs other than sulfasalazine or methotrexate, previous use of TNFi, regular exercise during past 6 months	1) Swimming + conventional exercise<br>2) Walking + conventional exercise<br>3) Conventional exercise only<br>"conventional exercises" not defined	1) 13<br>2) 12<br>3) 12	1) 50.2 (12.4)<br>2) 46.9 (13.4)<br>3) 48.4 (9.5)	1) 3 (23.1)<br>2) 4 (33.3)<br>3) 3 (25.0)	**No funding**

axSpA = axial spondyloarthritisN = number, RCT = randomised controlled trial, SD = standard deviation, TNFi = tumour necrosis factor inhibitors

Supplementary table 69 – Results from reviews and interventional studies of aquatic exercise in axSpA

Table – Aquatic exercises (axSpA), results and quality assessment

Outcome (outcome measure)	Study (date) [study type]	Standardised result, SMD (95% CI) unless otherwise stated	Natural result	AMSTAR2 quality	Rand. Seq.	Alloc. Conc.	Blind. Part.	Blind. Asses.
Pain	Regel (2017) [SR][179]		1 study[216] reported a moderate difference between the groups	Moderate				
	Zao (2017) [SR][215]		1 study reported improvements	Low				
	Dundar (2014) [RCT][216]	Aquatic vs land at 4 weeks<br>SMD -0.29 (-0.77, 0.18)	Pain VAS, BL / 4 weeks / 12 weeks, mean (SD)<br>Aquatic exercise: 5.1 (2.6) / 2.6 (2.5) / 2.5 (2.6)<br>Land base exercise: 4.9 (2.8) / 3.3 (2.3) / 3.4 (2.5)		H/UC	H/UC	H/UC	L
	Karapolat (2009) [RCT][217]	Aquatic vs control at 6 weeks<br>vs walking: SMD 0.19 (-0.60, 0.98)<br>vs conventional exercise only: SMD 0.13 (-0.66, 0.91)	Nottingham Health Profile - pain, BL / 6 weeks, mean (SD)<br>Aquatic + conventional exercise: 27.89 (32.74) / 25.00 (28.41)<br>Walking+ conventional exercise: 25.00 (25.62) / 19.79 (26.89)<br>Conventional exercise: 25.75 (25.28) / 21.04 (34.32)		H/UC	H/UC	H/UC	H/UC
	Bespoke meta-analysis including[216;217]	Aquatic exercise vs control<br>SMD -0.18 (-0.59, 0.23) I^2 0%						
Function	Regel (2017) [SR][179]		1 study[216] reported a no difference between the groups	Moderate				
	Sharan (2017) [SR][195]		Group therapies in the water had beneficial effects on function	Critically low §				
	Zao (2017) [SR][215]		2/2 studies reported improvements	Low				
	Dundar (2014) [RCT][216]	Aquatic vs land at 4 weeks<br>SMD 0.00 (-0.47, 0.47)	BASFI, BL / 4 weeks / 12 weeks, mean (SD)<br>Aquatic exercise: 3.5 (2.9) / 2.5 (2.2) / 2.6 (2.4)<br>Land base exercise: 3.6 (2.8) / 2.5 (2.2) / 2.6 (2.3)		H/UC	H/UC	H/UC	L
	Karapolat (2009) [RCT][217]	Aquatic vs control at 6 weeks<br>vs walking: SMD -0.15 (-0.94, 0.63)<br>vs conventional exercise only: SMD -0.57 (-1.37, 0.23)	BASFI, BL / 6 weeks, mean (SD)<br>Aquatic + conventional exercise: 2.34 (1.70) / 1.97 (1.24)<br>Walking+ conventional exercise: 2.25 (1.81) / 2.25 (2.30)<br>Conventional exercise: 2.70 (2.52) / 3.13 (2.65)		H/UC	H/UC	H/UC	H/UC
	Bespoke meta-analysis including[216;217]	Aquatic exercise vs control<br>SMD -0.19 (-0.71, 0.34) I^2 30.3%						

Alloc. Conc. = allocation concealment, AMSTAR2 = Assessing the Methodological Quality of Systematic Reviews, axSpA = axial spondyloarthritis, BASFI = Bath Ankylosing Spondylitis Functional Index, BL = baseline, Blind. Asses. = Blinded assessors, Blind. Part. = blinded participants, CI = confidence interval, H/UC = high / unclear risk of bias, L = low risk of bias, Rand. Seq. = random sequence generation, RCT = randomised controlled trial, SD = standard deviation, SMD = standardised mean difference, VAS = visual analogue scale

Table – Aquatic exercises (axSpA), results and quality assessment

Outcome (outcome measure)	Study (date) [study type]	Standardised result, SMD (95% CI) unless otherwise stated	Natural result	AMSTAR2 quality	Rand. Seq.	Alloc. Conc.	Blind. Part.	Blind. Asses.
Disease activity	Regel (2017) [SR][179]		1 study[216] reported a small difference between the groups	Moderate				
	Zao (2017) [SR][215]		2/3 studies reported improvements	Low				
	Dundar (2014) [RCT][216]	Aquatic vs land at 4 weeks<br>SMD -0.11 (-0.58, 0.36)	BASDAI, BL / 4 weeks / 12 weeks, mean (SD)<br>Aquatic exercise: 3.9 (1.9) / 2.6 (1.5) / 2.7 (1.7)<br>Land base exercise: 4.0 (2.3) / 2.8 (2.1) / 2.8 (2.5)		H/UC	H/UC	H/UC	L
	Karapolat (2009) [RCT][217]	Aquatic vs control at 6 weeks<br>vs walking: SMD -0.41 (-1.20, 0.39)<br>vs conventional exercise only: SMD -0.08 (-0.86, 0.71)	BASDAI, BL / 6 weeks, mean (SD)<br>Aquatic + conventional exercise: 2.73 (1.93) / 1.90 (1.61)<br>Walking+ conventional exercise: 2.49 (1.68) / 2.68 (2.19)<br>Conventional exercise: 2.65 (2.13) / 2.03 (1.86)		H/UC	H/UC	H/UC	H/UC
	Bespoke meta-analysis including[216;217]	Aquatic exercise vs control<br>SMD -0.10 (-0.51, 0.30) I^2 30.3%						
Stiffness	Zao (2017) [SR][215]		3/3 studies reported improvements	Low				
Spinal mobility	Regel (2017) [SR][179]		1 study[216] reported a small difference between the groups	Moderate				
	Dundar (2014) [RCT][216]	Aquatic vs land at 4 weeks<br>SMD 0.04 (-0.43, 0.51)	BASMI, BL / 4 weeks / 12 weeks, mean (SD)<br>Aquatic exercise: 5.3 (2.7) / 4.0 (2.4) / 4.1 (2.6)<br>Land base exercise: 5.2 (3.1) / 3.9 (2.8) / 4.0 (2.7)		H/UC	H/UC	H/UC	L
	Karapolat (2009) [RCT][217]	Aquatic vs control at 6 weeks<br>vs walking: SMD 0.14 (-0.65, 0.93)<br>vs conventional exercise only: SMD 0.33 (-0.46, 1.12)	BASMI, BL / 6 weeks, mean (SD)<br>Aquatic + conventional exercise: 5.15 (2.27) / 4.54 (2.07)<br>Walking+ conventional exercise: 4.54 (2.58) / 4.18 (2.99)<br>Conventional exercise: 3.83 (3.75) / 3.75 (2.67)		H/UC	H/UC	H/UC	H/UC
	Bespoke meta-analysis including[216;217]	Aquatic exercise vs control<br>SMD 0.12 (-0.29, 0.52) I^2 0%						

Alloc. Conc. = allocation concealment, AMSTAR2 = Assessing the Methodological Quality of Systematic Reviews, axSpA = axial spondyloarthritis, BASDAI = Bath Ankylosing Spondylitis Disease Activity Index, BASMI = Bath Ankylosing Spondylitis Metrology Index, BL = baseline, Blind. Asses. = Blinded assessors, Blind. Part. = blinded participants, CI = confidence interval, H/UC = high / unclear risk of bias, L = low risk of bias, Rand. Seq. = random sequence generation, RCT = randomised controlled trial, SD = standard deviation, SMD = standardised mean difference

Table – Aquatic exercises (axSpA), results and quality assessment

Outcome (outcome measure)	Study (date) [study type]	Standardised result, SMD (95% CI) unless otherwise stated	Natural result	AMSTAR2 quality	Rand. Seq.	Alloc. Conc.	Blind. Part.	Blind. Asses.
Quality of life	Zao (2017) [SR][215]		1 study reported improvements	Low				
Depression	Karapolat (2009) [RCT][217]	Aquatic vs control at 6 weeks<br>vs walking: SMD -0.62 (-1.42, 0.19)<br>vs conventional exercise only: SMD 0.06 (-0.73, 0.85)	BDI, BL / 6 weeks, mean (SD)<br>Aquatic + conventional exercise: 6.85 (6.52) / 5.47 (4.77)<br>Walking+ conventional exercise: 8.50 (5.36) / 9.70 (8.59)<br>Conventional exercise: 6.17 (10.02) / 5.00 (10.22)		H/UC	H/UC	H/UC	H/UC
Spinal flexion	Dundar (2014) [RCT][216]	Aquatic vs land at 4 weeks<br>SMD -0.04 (-0.52, 0.43)	Schober's test, BL / 4 weeks / 12 weeks, mean (SD)<br>Aquatic exercise: 2.9 (1.9) / 3.7 (2.1) / 3.7 (2.4)<br>Land base exercise: 3.1 (2.1) / 3.8 (2.4) / 3.7 (2.2)		H/UC	H/UC	H/UC	L

Alloc. Conc. = allocation concealment, AMSTAR2 = Assessing the Methodological Quality of Systematic Reviews, axSpA = axial spondyloarthritis, BDI = Beck Depression Inventory, BL = baseline, Blind. Asses. = Blinded assessors, Blind. Part. = blinded participants, CI = confidence interval, H/UC = high / unclear risk of bias, L = low risk of bias, Rand. Seq. = random sequence generation, RCT = randomised controlled trial, SD = standard deviation, SMD = standardised mean difference

Table – Aquatic exercises (axSpA), SF36 results at final follow-up, mean (SD)

Author (date)	PCS	MCS	GH	PF	RP	RE	SF	BP	V	MH
Dundar (2014) [Aquatic][216]			77.4 (21.3)	80.8 (26.8)	77.7 (29.3)	80.4 (31.1)	89.5 (32.1)	78.6 (29.9)	74.8 (25.3)	89.4 (23.7)
Dundar (2014) [Control][216]			65.2 (25.6)	76.4 (24.9)	71.2 (26.3)	67.5 (27.3)	74.2 (28.3)	66.5 (21.3)	65.7 (26.1)	82.4 (23.8)

axSpA = axial spondyloarthritis, BP = bodily pain, FU = follow-up, GH = general health, MCS = mental component score, MH = mental health, PCS = physical component score, PF = physical function, RE = role emotional, RP = role physical, SD = standard deviation, SF = social functioning, V = vitality

Supplementary table 70 – Description of reviews of studies of home-based exercise in axSpA

Table – Home-based exercises (axSpA), description of reviews

Authors (date)	Review type	Study type included	Exposure detail	Number of studies included	Funders
Liang (2015)[192]	MA	RCTs	Home-based exercise programs including muscle relaxation, exercises for spine, range of motion, stretching, muscle strengthening and respiratory exercises	6	**Not reported –** Authors declared no conflicts of interest
O'Dwyer (2014)[180]	SR	RCTs	Unsupervised exercise interventions vs Supervised	4	**Not reported –** Authors declared no conflicts of interest

axSpA = axial spondyloarthritis, MA = meta-analysis, RCT = randomised controlled trial, SR = systematic review

Supplementary table 71 – Description of original studies of home-based exercise in axSpA

Table – Home based interventions (axSpA), description of included studies

Author (date) [country]	Study design	Inclusion criteria	Exposure detail	N	Age years, mean (SD)	N (%) female	Funders
Aydin (2016) [Turkey][198]	RCT	New York criteria, aged 20-65 years old, not practising regular exercise during previous 6 months, able to understand questionnaires, no co-existing systemic disease, no TNFi therapy, heart functional class I-III	1) Home based exercise – callisthenic exercises (consecutive and repetitive exercises aimed at training large muscle groups through aerobic and step routines)<br>2) Hospital based exercises – same as above	1) 19<br>2) 18	1) 33.5 (7.7)<br>2) 35.8 (8.1)	1) 8 (42.1)<br>2) 9 (50.0)	**Not reported –** authors declared no conflict of interest
Karahan (2016) [Turkey][181]	RCT	Modified New York criteria, aged 18-65 years, lack of regular exercise during previous 6 months, ability to understand questionnaires<br>Exclusions: cardiopulmonary dysfunction, central or peripheral neurological disease, issues hindering standing, psychiatric disorder, visual disorder, hearing disorder	1) Exergram – Microsoft Xbox 360 Kinect game console – 30 mins per day, 5x per week<br>p) No exercise program	1) 28<br>p) 29	1) 36.1 (12.4)<br>p) 36.6 (11.3)	1) 6 (21.4)<br>p) 7 (24.1)	**Not reported –** Authors declared no conflicts of interest
Hsieh (2014) [Taiwan][218]	RCT	Modified New York criteria, aged 20-65 years, well controlled disease, symptom duration >6 months<br>Exclusions: presence of serious medical conditions or acute febrile disorders, history of arthroplasties or major operations in the knee or hip joint, severe arthritis or contracture of knee or hip joints which preclude exercise	1) Range of motion and strengthening exercises at home<br>2) Range of motion only at home	1) 9<br>2) 10	1) 36.2 (11.7)<br>2) 42.1 (8.8)	1) 3 (33.3)<br>2) 3 (30.0)	**Government** (Taiwan National Science Council)
Rodriguez-Lozano (2013) [Spain][219]	RCT	Modified New York criteria, aged 18-70 years<br>Exclusions: severe AS with significant loss of motion and ankyloses precluding physical exercise, patients with other spondyloarthritis or concomitant diseases in which exercise could be contra-indicated	1) Education and range of motion / stretching exercises<br>p) Usual care	1) 381<br>p) 375	1) 45 (12)<br>p) 46 (11)	1) 29%<br>p) 27%	**Professional body** (Spanish Society of Rheumatology)
Lim (2005) [South Korea][220]	RCT	Outpatient without complications, sedentary, understands questionnaires, no changes in medications, functional class II	1) Home based exercise program – muscle relaxation, flexibility, muscular strength, breathing and posture.<br>p) Waitlist control	1) 25<br>p) 25	1) 38.8 (9.3)<br>p) 28.1 (7.5)	1) 6 (24.0)<br>p) 5 (20.0)	**Not reported**

AS = ankylosing spondylitis, axSpA = axial spondyloarthritis, N = number, RCT = randomised controlled trial, SD = standard deviation

Table – Home based interventions (axSpA), description of included studies

Author (date) [country]	Study design	Inclusion criteria	Exposure detail	N	Age years, mean (SD)	N (%) female	Funders
Analay (2003) [Turkey][202]	RCT	Amor criteria, aged 18-55 years, able to participate in exercise<br>Exclusions: systemic organ involvement, severe deformities or limited hip and knee joint motion preventing cycling, treated by physiotherapist in last 3 months or practising regular exercise and those received DMARDs	1) Intensive exercise regime given by physiotherapist. Program included stretching, mobilization, aerobic exercises on exercise bike, strengthening exercises for lower and upper extremities and back<br>2) Practice same exercises at home	1) 23<br>2) 22	1) 37.6 (11.3)<br>2) 34.3 (7.9)	1) 3 (13.0)<br>2) 4 (18.2)	**Not reported**
Sweeney (2002) [UK][185]	RCT	Aged 16-65 years	1) Delivered a home exercise regime video, booklet and wall chart<br>p) Usual care	1) 100<br>p) 100	1) 47 (10.2)<br>p) 47 (9.6)	1) 30 (30%) F<br>p) 32 (32%) F	**Industry** (Bupa), **Charity** (National Ankylosing Spondylitis Society, John Coates Charitable Trust, and Col. W.W. Pilkington Trust)
Kraag (1990) [Canada][205]	RCT	New York criteria, English language, stable steroids for past 3 months, DMARDs stable for 6 months, no surgery in next 4 months, not pregnant and using contraception<br>Exclusions: 10% loss of flexion in either hip joint, receiving contravening treatment	1) Home physiotherapy – therapeutic exercise aimed at increasing strength and endurance + education about posture<br>p) Waitlist control	1) 26<br>p) 27	1) 18-35: 13<br>≥36: 13<br>p) 18-35: 14<br>≥ 36: 13	1) 6 (23.1)<br>p) 5 (18.5)	**Government** (Health and Welfare Canada)
Yigit (2013) [Turkey][221]	NRT	Aged 18-65 years, Modified New York Criteria, Received TNFi for ≥3 months, understand context of program<br>Exclusions: Severe comorbidities affecting heart, lung, liver or kidneys, mental retardation, presence of severe arthritis or prosthetic device and exercising regularly in past 6 months	1) Home based exercise program – 5x per week, muscle relaxation, flexibility, range of motion and strengthening exercises as well as exercises to improve posture<br>p) Group who did not do the exercise program	1) 20<br>p) 20	1) 40.3 (8.1)<br>p) 36.5 (7.2)	1) 5 (25.0)<br>p) 3 (15.0)	**Not reported –** authors declared no conflict of interest
Aytekin (2012) [Turkey][207]	NRT	Modified New York criteria<br>Exclusions: presences of prosthesis, hypertension, cardiovascular disease, chronic obstructive pulmonary disease, posteroanterior chest X-ray abnormalities	Home-based exercise regime including range of motion exercises, stretching, strengthening, posture and respiratory exercises – demonstrated by physiotherapist<br>1) Those who performed exercises ≥5x per week<br>p) Those who performed exercises <5x per week	1) 34<br>p) 32	1) 34.4 (9.5)<br>p) 35.8 (6.7)	1) 9 (26.5)<br>p) 5 (15.6)	**Not reported –** authors declared no conflict of interest

axSpA = axial spondyloarthritis, N = number, NRT = non-randomised trial, RCT = randomised controlled trial, SD = standard deviation, UK = United Kingdom

Table – Home based interventions (axSpA), description of included studies

Author (date) [country]	Study design	Inclusion criteria	Exposure detail	N	Age years, mean (SD)	N (%) female	Funders
Durmus (2009) [Turkey][222]	NRT	New York criteria, not been practicing regular exercise during previous 6 months, able to understand content of questionnaire / experimental schedules, no co-existent systemic diseases, not been given TNFi, functional class I-III	1) Home based exercise program – 7 days per week, 20 exercises for muscle relaxation, strength stronger breathing and straighter posture<br>p) Non-exercise control	1) 25<br>p) 18	1) 37.3 (7.3)<br>p) 42.3 (8.2)	1) 4 (16.0)<br>p) 4 (22.2)	**Not reported –** authors declared no conflict of interest
Durmus (2009) [Turkey][223]	NRT	New York criteria, sedentary<br>Exclusions: Medical condition that impaired function more than AS, those with co-existant cardiac or respiratory diseases, severe arthritis, taking TNFi	1) "Global Posture Re-education" - Muscle chains are shortened and then stretched and strengthened. Includes dynamic axial exercise, static posture exercise, stretching.<br>2) Conventional exercises – motion and flexibility<br>p) Non-exercise control	1) 19<br>2) 19<br>p) 13	1) 38.1 (11.1)<br>2) 35.9 (7.3)<br>p) 43.5 (7.3)	1) 5 (26.3)<br>2) 2 (10.5)<br>p) 1 (7.7)	**Not reported**
Karapolat (2008) [Turkey][224]	NRT	Modified New York criteria, aged 18-75 years, clinically stable<br>Exclusions: Inability or unwillingness to participate in physiotherapy, systemic organic involvement, severe comorbidity of heart, lung, liver or kidneys, practising regular exercises in past 6 months	1) Group exercise program – respiratory exercises, stretching, mobilization, strengthening exercises<br>2) Those who couldn't participate in the group sessions performed the same exercises at home	1) 22<br>2) 16	1) 47.5 (11.8)<br>2) 46.6 (14.8)	1) 32%<br>2) 31%	**Not reported**
Kraag (1994) [Canada][212]	Single arm int.	New York criteria, English language, stable steroids for past 3 months, DMARDs stable for 6 months, no surgery in next 4 months, not pregnant and using contraception<br>Exclusions: 10% loss of flexion in either hip joint, receiving contravening treatment	Same intervention as Kraag (1990)[205] – original intervention group were followed up and provided support as needed, control group were given intervention	46	not reported	not reported	

axSpA = axial spondyloarthritis, N = number, NRT = non-randomised trial, RCT = randomised controlled trial, SD = standard deviation, TNFi = Tumour Necrosis Factor Inhibitor

Supplementary table 72 – Results from reviews and interventional studies of home-based exercise in axSpA

Table – Home based exercises (axSpA), results and quality assessment

Outcome (outcome measure)	Study (date) [study type]	Standardised result, SMD (95% CI) unless otherwise stated	Natural result	AMSTAR2 quality	Rand. Seq.	Alloc. Conc.	Blind. Part.	Blind. Asses.
Pain	Liang (2015) [MA][192]	Exercise vs control<br>SMD -0.22 (-0.49, 0.06)		Moderate				
	Karahan (2016) [RCT][181]	Exercise vs control at 8 weeks<br>SMD -0.67 (-1.21, -0.14)	Pain VAS, BL / 8 weeks, mean (SD)<br>Exercise: 4.9 (2.0) / 3.6 (1.7)<br>Control: 5.1 (2.2) / 5.0 (2.4)		H/UC	L	H/UC	H/UC
	Rodriguez-Lozano (2013) [RCT][219]	Exercise vs control, change BL-24 weeks<br>SMD -0.14 (-0.28, 0.01)	Pain VAS (0-10), change BL-24 weeks, mean (SD‡)<br>Exercise: -0.76 (2.29)<br>Control: -0.44 (2.37)		L	L	H/UC	H/UC
	Lim (2005) [RCT][220]		Pain, % change BL-8 weeks<br>Exercise: -33%<br>Control: 28%		H/UC	H/UC	H/UC	L
	Analay (2003) [RCT][202]	Group vs home exercise<br>SMD 0.07 (-0.52, 0.66)	Rest pain, BL / 6 weeks / 3 months, mean (SD)<br>Group exercise: 3.82 (3.4) / 3.3 (2.3) / 3.43 (2.5)<br>Home exercise: 3.13 (2.6) / 3.09 (3.6) / 3.18 (3.1)		H/UC	L	H/UC	L
	Kraag (1990) [RCT][205]	Exercise vs control, change BL-4 months<br>SMD 0.39 (-1.50, 0.94)	Pain, Change BL-4 months, mean (SD)<br>Exercise: 5.2 (26.3)<br>Control: -5.2 (26.7)		H/UC	H/UC	H/UC	H/UC
	Bespoke meta-analysis including[181;205;219]	Exercise vs control<br>SMD -0.68 (-1.41, 0.05), I^2 88.9%						
	Aytekin (2012) [NRT][207]	Exercise ≥5x per week vs exercise <5x per week at 3 months<br>SMD 0.10 (-0.38, 0.58)	Pain VAS, BL / 3 months<br>Exercise ≥5x per week: 5.1 (2.1) / 4.1 (2.0)<br>Exercise <5x per week: 3.9 (2.3) / 3.9 (2.0)					
	Durmus (2009) [NRT][223]	Muscle strengthening exercise vs conventional exercises<br>SMD -0.29 (-0.93, 0.35)<br>Muscle strengthening exercise vs control<br>SMD -0.78 (-1.51, -0.05)	Pain VAS, BL / 12 weeks<br>Muscle strengthening exercise: 4.78 (2.63) / 1.42 (1.80)<br>Conventional exercise: 4.84 (2.81) / 1.94 (1.80)<br>Control: 3.46 (2.40) / 3.00 (2.34)					
	Karapolat (2008) [NRT][224]	Home exercise vs group exercise at 6 weeks<br>SMD 0.77 (0.11, 1.44)	NHP - pain, BL / 6 weeks, mean (SD)<br>Home exercise: 45.81 (32.00) / 39.50 (29.85)<br>Group exercise: 35.75 (32.11) / 19.55 (22.39)					
	Kraag (1994) [Single arm int.][212]		Pain, 4 months / 8 months<br>Original exercise group: 27.9 (21.6) / 25.1 (21.1)<br>Original control group: 42.8 (25.8) / 42.2 (32.2)					

Alloc. Conc. = allocation concealment, AMSTAR2 = Assessing the Methodological Quality of Systematic Reviews, axSpA = axial spondyloarthritis, BL = baseline, Blind. Asses. = Blinded assessors, Blind. Part. = blinded participants, CI = confidence interval, H/UC = high / unclear risk of bias, L = low risk of bias, MD = mean difference, NHP = Nottingham Health Profile, Rand. Seq. = random sequence generation, RCT = randomised controlled trial, SD = standard deviation, SMD = standardised mean difference, VAS = visual analogue scale

Table – Home based exercises (axSpA), results and quality assessment

Outcome (outcome measure)	Study (date) [study type]	Standardised result, SMD (95% CI) unless otherwise stated	Natural result	AMSTAR2 quality	Rand. Seq.	Alloc. Conc.	Blind. Part.	Blind. Asses.
Function	Liang (2015) [MA][192]		BASFI, mean difference<br>MD -0.39 (-0.57, -0.20)	Moderate				
	O'Dwyer (2014)[180]		1/4 studies reported significant difference in favour of group therapy for function	Moderate				
	Aydin (2016) [RCT][198]	Home vs hospital based exercise at 8 weeks<br>SMD 0.48 (-0.18, 1.13)	BASFI, BL / 8 weeks, mean (SD)<br>Home based exercise: 3.64 (2.87) / 3.78 (2.67)<br>Hospital based exercise: 3.16 (2.43) / 2.63 (2.07)		L	H/UC	H/UC	H/UC
	Karahan (2016) [RCT][181]	Exercise vs control at 8 weeks<br>SMD -0.66 (-1.19, -0.13)	BASFI, BL / 8 weeks, mean (SD)<br>Exercise: 3.7 (1.5) / 2.9 (1.3)<br>Control: 3.9 (1.6) / 3.9 (1.7)		H/UC	L	H/UC	H/UC
	Hsieh (2014) [RCT][218]	Strength + range of motion vs range of motion only at 3 months<br>SMD -0.58 (-1.50, 0.34)	BASFI, BL / 3 months, mean (SD)<br>Strength + range of motion: 3.7 (3.3) / 1.9 (2.3)<br>Range of motion only: 3.5 (2.9) / 3.5 (3.1)		L	L	H/UC	L
	Rodriguez-Lozano (2013) [RCT][219]	Exercise vs control, change BL-24 weeks<br>SMD -0.21 (-0.35, -0.07)	BASFI, change BL-24 weeks, mean (SD‡)<br>Exercise: -0.54 (1.39)<br>Control: -0.21 (1.74)		L	L	H/UC	H/UC
	Lim (2005) [RCT][220]		Functional capacity, % change BL-8 weeks<br>Exercise: 46%<br>Control: unchanged		H/UC	H/UC	H/UC	L
	Analay (2003) [RCT][202]	Group vs home exercise<br>SMD -0.39 (-0.98, 0.20)	BASFI, BL / 6 weeks / 3 months, mean (SD)<br>Group exercise: 26.34 (20.10) / 20.0 (16.76) / 22.0 (17.15)<br>Home exercise: 27.59 (17.82) / 27.31 (20.42) / 26.13 (17.20)		H/UC	L	H/UC	L
	Sweeney (2002) [RCT][185]	Exercise vs control at 6 months<br>SMD -0.15 (-0.43, 0.13)	BASFI, BL / 6 months, mean (SD)<br>Exercise: 3.5 (2.4) / 3.06 (2.35)<br>Control: 3.6 (2.4) / 3.43 (2.61)		H/UC	H/UC	H/UC	H/UC
	Kraag (1990) [RCT][205]	Exercise vs control, change BL-4 months<br>SMD 1.68 (1.05, 2.31)	Toronto Activities of Daily Living, Change BL-4 months, mean (SD)<br>Exercise: 3.92 (2.94)<br>Control: -0.19 (1.86)		H/UC	H/UC	H/UC	H/UC
	Bespoke meta-analysis:<br>Exercise vs control[181;185;205;219]<br>Home vs group exercise [198;202]	Exercise vs control<br>SMD -0.58 (-1.03, -0.12), I^2 86.8%<br>Home exercise vs group<br>SMD 0.43 (-0.01, 0.87), I^2 0% in favour of groups						

‡ SD calculated from 95% CI in paper; Alloc. Conc. = allocation concealment, AMSTAR2 = Assessing the Methodological Quality of Systematic Reviews, axSpA = axial spondyloarthritis, BASFI = Bath Ankylosing Spondylitis Functional Index, BL = baseline, Blind. Asses. = Blinded assessors, Blind. Part. = blinded participants, CI = confidence interval, H/UC = high / unclear risk of bias, L = low risk of bias, MD = mean difference Rand. Seq. = random sequence generation, RCT = randomised controlled trial, SD = standard deviation, SMD = standardised mean difference

Table – Home based exercises (axSpA), results and quality assessment

Outcome (outcome measure)	Study (date) [study type]	Standardised result, SMD (95% CI) unless otherwise stated	Natural result	AMSTAR2 quality	Rand. Seq.	Alloc. Conc.	Blind. Part.	Blind. Asses.
Function	Yigit (2013) [NRT][221]	Muscle strengthening exercise vs control<br>SMD -0.77 (-1.41, -0.12)	BASFI, BL / 10 weeks<br>Muscle strengthening exercise: 3.22 (2.96) / 2.27 (2.10)<br>Control: 3.86 (2.36) / 4.00 (2.41)					
	Aytekin (2012) [NRT][207]	Exercise ≥5x per week vs exercise <5x per week at 3 months<br>SMD -0.43 (-0.92, 0.06)	BASFI, BL / 3 months<br>Exercise ≥5x per week: 2.54 (2.26) / 2.05 (2.14)<br>Exercise <5x per week: 2.90 (2.30) / 2.99 (2.26)					
	Durmus (2009) [NRT][222]	Muscle strengthening exercise vs control<br>SMD -0.89 (-1.53, -0.25)	BASFI, BL / 12 weeks, mean (SD)<br>Muscle strengthening exercise: 2.22 (1.53) / 1.25 (1.07)<br>Control: 2.55 (1.40) / 2.30 (1.32)					
	Durmus (2009) [NRT][223]	Muscle strengthening exercise vs conventional exercises<br>SMD -0.38 (-1.02, 0.26)<br>Muscle strengthening exercise vs control<br>SMD -0.82 (-1.56, -0.09)	BASFI, BL / 12 weeks<br>Muscle strengthening exercise: 2.65 (2.39) / 1.28 (1.47)<br>Conventional exercise: 3.18 (2.05) / 1.83 (1.41)<br>Control: 3.32 (2.59) / 2.97 (2.71)					
	Karapolat (2008) [NRT][224]	Home exercise vs group exercise at 6 weeks<br>SMD -0.15 (-0.80, 0.49)	BASFI, BL / 6 weeks, mean (SD)<br>Home exercise: 1.76 (1.67) / 1.76 (1.96)<br>Group exercise: 2.62 (2.15) / 2.05 (1.84)					
	Kraag (1994) [Single arm int.][212]		Toronto Activities of Daily Living, 4 months / 8 months<br>Original exercise group: 19.9 (3.9) / 2.3 (4.2)<br>Original control group: 16.6 (2.8) / 0.8 (2.4)					

Alloc. Conc. = allocation concealment, AMSTAR2 = Assessing the Methodological Quality of Systematic Reviews, axSpA = axial spondyloarthritis, BASFI = Bath Ankylosing Spondylitis Functional Index, Blind. Asses. = Blinded assessors, BL = baseline, Blind. Part. = blinded participants, CI = confidence interval, H/UC = high / unclear risk of bias, L = low risk of bias, NRT = non-randomised trial, Rand. Seq. = random sequence generation, RCT = randomised controlled trial, SD = standard deviation, SMD = standardised mean difference

Table – Home based exercises (axSpA), results and quality assessment

Outcome (outcome measure)	Study (date) [study type]	Standardised result, SMD (95% CI) unless otherwise stated	Natural result	AMSTAR2 quality	Rand. Seq.	Alloc. Conc.	Blind. Part.	Blind. Asses.
Disease activity	Liang (2015) [MA][192]		<u>BASDAI, mean difference</u><br>MD -0.50 (-0.99, -0.02)	Moderate				
	O'Dwyer (2014)[180]		1/2 studies reported significant difference in favour of group therapy for disease activity	Moderate				
	Aydin (2016) [RCT][198]	<u>Home vs hospital based exercise at 8 weeks</u><br>SMD 0.52 (-0.14, 1.17)	<u>BASDAI, BL / 8 weeks, mean (SD)</u><br>Home based exercise: 5.02 (2.43) / 4.66 (2.02)<br>Hospital based exercise: 4.15 (1.79) / 3.66 (1.84)		L	H/UC	H/UC	H/UC
	Karahan (2016) [RCT][181]	<u>Exercise vs control at 8 weeks</u><br>SMD -0.51 (-1.04, 0.02)	<u>BASDAI, BL / 8 weeks, mean (SD)</u><br>Exercise: 4.1 (1.8) / 3.2 (1.3)<br>Control: 4.2 (2.1) / 4.1 (2.1)		H/UC	L	H/UC	H/UC
	Hsieh (2014) [RCT][218]	<u>Strength + range of motion vs range of motion only at 3 months</u><br>SMD -0.32 (-1.23, 0.59)	<u>BASDAI, BL / 3 months, mean (SD)</u><br>Strength + range of motion: 4.2 (1.9) / 3.7 (1.8)<br>Range of motion only: 4.5 (2.1) / 4.5 (3.0)		L	L	H/UC	L
	Rodriguez-Lozano (2013) [RCT][219]	<u>Exercise vs control, change BL-24 weeks</u><br>SMD -0.16 (-0.30, -0.02)	<u>BASDAI, change BL-24 weeks, mean (SD‡)</u><br>Exercise: -0.65 (1.74)<br>Control: -0.37 (1.78)		L	L	H/UC	H/UC
	Sweeney (2002) [RCT][185]	<u>Exercise vs control at 6 months</u><br>SMD 0.08 (-0.20, 0.35)	<u>BASDAI, BL / 6 months, mean (SD)</u><br>Exercise: 3.9 (2.4) / 3.65 (2.00)<br>Control: 3.8 (2.3) / 3.49 (2.16)		H/UC	H/UC	H/UC	H/UC
	Bespoke meta-analysis including[181;185;219]	<u>Exercise vs control</u><br>SMD -0.13 (-0.37, 0.10), I^2 53.7%						
	Yigit (2013) [NRT][221]	<u>Muscle strengthening exercise vs control</u><br>SMD -0.55 (-1.19, 0.08)	<u>BASDAI, BL / 10 weeks</u><br>Muscle strengthening exercise: 3.85 (2.45) / 2.61 (1.83)<br>Control: 3.81 (2.38) / 3.77 (2.33)					
	Aytekin (2012) [NRT][207]	<u>Exercise ≥5x per week vs exercise <5x per week at 3 months</u><br>SMD -0.14 (-0.63, 0.34)	<u>BASDAI, BL / 3 months</u><br>Exercise ≥5x per week: 4.44 (2.07) / 3.77 (1.98)<br>Exercise <5x per week: 3.98 (2.19) / 4.07 (2.21)					
	Durmus (2009) [NRT][222]	<u>Muscle strengthening exercise vs control</u><br>SMD -1.12 (-1.77, -0.47)	<u>BASDAI, BL / 12 weeks, mean (SD)</u><br>Muscle strengthening exercise: 2.52 (1.25) / 1.35 (0.78)<br>Control: 2.63 (1.07) / 2.34 (1.01)					
	Durmus (2009) [NRT][223]	<u>Muscle strengthening exercise vs conventional exercises</u><br>SMD -0.20 (-0.84, 0.44)<br><u>Muscle strengthening exercise vs control</u><br>SMD -0.66 (-1.38, 0.07)	<u>BASDAI, BL / 12 weeks</u><br>Muscle strengthening exercise: 2.73 (1.31) / 1.33 (0.88)<br>Conventional exercise: 2.96 (1.21) / 1.50 (0.83)<br>Control: 2.50 (1.31) / 2.08 (1.44)					
	Karapolat (2008) [NRT][224]	<u>Home exercise vs group exercise at 6 weeks</u><br>SMD -0.27 (0.92, 0.38)	<u>BASDAI, BL / 6 weeks, mean (SD)</u><br>Home exercise: 3.03 (2.25) / 1.99 (1.50)<br>Group exercise: 3.30 (2.46) / 2.41 (1.62)					

Alloc. Conc. = allocation concealment, AMSTAR2 = Assessing the Methodological Quality of Systematic Reviews, axSpA = axial spondyloarthritis, BASDAI = Bath Ankylosing Spondylitis Disease Activity Index, BL = baseline, Blind. Asses. = Blinded assessors, Blind. Part. = blinded participants, CI = confidence interval, H/UC = high / unclear risk of bias, L = low risk of bias, MD = mean difference, Rand. Seq. = random sequence generation, RCT = randomised controlled trial, SD = standard deviation, SMD = standardised mean difference,

Table – Home based exercises (axSpA), results and quality assessment

Outcome (outcome measure)	Study (date) [study type]	Standardised result, SMD (95% CI) unless otherwise stated	Natural result	AMSTAR2 quality	Rand. Seq.	Alloc. Conc.	Blind. Part.	Blind. Asses.
Stiffness	Analay (2003) [RCT][202]	Group vs home exercise<br>SMD -0.33 (-0.92, 0.26)	Morning stiffness, BL / 6 weeks / 3 months, mean (SD)<br>Group exercise: 38.65 (60.32) / 20.87 (32.34) / 24.04 (36.24)<br>Home exercise: 36.59 (51.44) / 37 (62.01) / 35.54 (36.77)		H/UC	L	H/UC	L
	Aytekin (2012) [NRT][207]	Exercise ≥5x per week vs exercise <5x per week at 3 months<br>SMD 0.01 (-0.47, 0.49)	Morning stiffness, BL / 3 months<br>Exercise ≥5x per week: 55.15 (70.94) / 39.11 (65.86)<br>Exercise <5x per week: 32.66 (32.40) / 38.59 (34.43)					
Spinal mobility	Aydin (2016) [RCT][198]	Home vs hospital based exercise at 8 weeks<br>SMD 0.57 (-0.09, 1.23)	BASMI, BL / 8 weeks, mean (SD)<br>Home based exercise: 2.42 (1.50) / 2.52 (1.34)<br>Hospital based exercise: 2.38 (1.19) / 1.83 (1.04)		L	H/UC	H/UC	H/UC
	Yigit (2013) [NRT][221]	Muscle strengthening exercise vs control<br>SMD -0.60 (-1.24, 0.03)	BASMI, BL / 10 weeks<br>Muscle strengthening exercise: 5.05 (2.74) / 4.15 (2.62)<br>Control: 5.55 (2.50) / 5.70 (2.52)					
	Karapolat (2008) [NRT][224]	Home exercise vs group exercise at 6 weeks<br>SMD -0.53 (-1.19, 0.12)	BASMI, BL / 6 weeks, mean (SD)<br>Home exercise: 3.06 (2.35) / 2.94 (2.35)<br>Group exercise: 4.77 (2.29) / 4.18 (2.30)					

Alloc. Conc. = allocation concealment, AMSTAR2 = Assessing the Methodological Quality of Systematic Reviews, axSpA = axial spondyloarthritis, BASMI = Bath Ankylosing Spondylitis Metrology Index, BL = baseline, Blind. Asses. = Blinded assessors, Blind. Part. = blinded participants, CI = confidence interval, H/UC = high / unclear risk of bias, L = low risk of bias, Rand. Seq. = random sequence generation, RCT = randomised controlled trial, SD = standard deviation, SMD = standardised mean difference

Table – Home based exercises (axSpA), results and quality assessment

Outcome (outcome measure)	Study (date) [study type]	Standardised result, SMD (95% CI) unless otherwise stated	Natural result	AMSTAR2 quality	Rand. Seq.	Alloc. Conc.	Blind. Part.	Blind. Asses.
Patient global	Aydin (2016) [RCT][198]	Home vs hospital based exercise at 8 weeks<br>SMD 0.18 (-0.46, 0.83)	BAS-G, BL / 8 weeks, mean (SD)<br>Home based exercise: 5.10 (2.11) / 4.80 (1.67)<br>Hospital based exercise: 4.58 (1.88) / 4.45 (2.14)		L	H/UC	H/UC	H/UC
	Hsieh (2014) [RCT][218]	Strength + range of motion vs range of motion only at 3 months<br>SMD -0.17 (-1.08, 0.73)	BAS-G, BL / 3 months, mean (SD)<br>Strength + range of motion: 5.6 (2.7) / 3.6 (2.0)<br>Range of motion only: 5.0 (2.8) / 4.1 (3.5)		L	L	H/UC	L
	Rodriguez-Lozano (2013) [RCT][219]	Exercise vs control, change BL-24 weeks<br>SMD -0.18 (-0.32, -0.03)	Patients global VAS (0-10), change BL-24 weeks, mean (SD‡)<br>Exercise: -0.75 (2.24)<br>Control: -0.36 (2.22)		L	L	H/UC	H/UC
	Sweeney (2002) [RCT][185]	Exercise vs control at 6 months<br>SMD -0.00 (-0.28, 0.27)	BAS-G, BL / 6 months, mean (SD)<br>Exercise: 4.0 (2.6) / 3.60 (2.61)<br>Control: 3.7 (2.6) / 3.61 (2.81)		H/UC	H/UC	H/UC	H/UC
	Bespoke meta-analysis including[185;219]	Exercise vs control<br>SMD -0.13 (-0.28, 0.01), I^2 13.6%						
Quality of life	Aydin (2016) [RCT][198]	Home vs hospital based exercise at 8 weeks<br>SMD 0.58 (-0.08, 1.23)	ASQOL, BL / 8 weeks, mean (SD)<br>Home based exercise: 9.63 (5.41) / 9.00 (5.06)<br>Hospital based exercise: 7.11 (4.33) / 6.22 (4.59)		L	H/UC	H/UC	H/UC
	Karahan (2016) [RCT][181]	Exercise vs control at 8 weeks<br>SMD -0.64 (-1.17, -0.11)	ASQOL, BL / 8 weeks, mean (SD)<br>Exercise: 9.5 (6.1) / 6.8 (4.3)<br>Control: 10.2 (6.0) / 10.3 (6.4)		H/UC	L	H/UC	H/UC
	Rodriguez-Lozano (2013) [RCT][219]	Exercise vs control, change BL-24 weeks<br>SMD -0.25 (-0.39, -0.11)	ASQOL, change BL-24 weeks, mean (SD‡)<br>Exercise: -0.98 (3.04)<br>Control: -0.23 (3.01)		L	L	H/UC	H/UC
	Bespoke meta-analysis including[181;219]	Exercise vs control<br>SMD -0.36 (-0.70, -0.01), I^2 48.4%						
	Aytekin (2012) [NRT][207]	Exercise ≥5x per week vs exercise <5x per week at 3 months<br>SMD -0.50 (-0.99, -0.01)	ASQOL, BL / 3 months<br>Exercise ≥5x per week: 9.56 (5.21) / 7.29 (4.6)<br>Exercise <5x per week: 9.34 (5.97) / 9.96 (6.1)					

‡ SD calculated from 95% CI in paper

Alloc. Conc. = allocation concealment, AMSTAR2 = Assessing the Methodological Quality of Systematic Reviews, BL = baseline, Blind. Asses. = Blinded assessors, Blind. Part. = blinded participants, CI = confidence interval, H/UC = high / unclear risk of bias, L = low risk of bias, Rand. Seq. = random sequence generation, RCT = randomised controlled trial, SD = standard deviation, SMD = standardised mean difference

Table – Home based exercises (axSpA), results and quality assessment

Outcome (outcome measure)	Study (date) [study type]	Standardised result, SMD (95% CI) unless otherwise stated	Natural result	AMSTAR2 quality	Rand. Seq.	Alloc. Conc.	Blind. Part.	Blind. Asses.
Fatigue	Yigit (2013) [NRT][221]	Muscle strengthening exercise vs control<br>SMD -0.68 (-1.32, -0.04)	MAF, BL / 10 weeks<br>Muscle strengthening exercise: 21.48 (12.62) / 15.95 (11.52)<br>Control: 24.85 (13.60) / 24.42 (13.38)					
	Durmus (2009) [NRT][222]	Muscle strengthening exercise vs control<br>SMD -0.45 (-1.06, 0.16)	MAF, BL / 12 weeks, mean (SD)<br>Muscle strengthening exercise: 2.07 (0.77) / 1.30 (1.08)<br>Control: 2.02 (0.76) / 1.73 (0.74)		H/UC	H/UC	H/UC	H/UC
	Karapolat (2008) [NRT][224]	Home exercise vs group exercise at 6 weeks<br>SMD 0.21 (-0.44, 0.85)	NHP - fatigue, BL / 6 weeks, mean (SD)<br>Home exercise: 41.41 (33.20) / 32.10 (26.14)<br>Group exercise: 36.05 (28.75) / 26.64 (26.20)					

Alloc. Conc. = allocation concealment, AMSTAR2 = Assessing the Methodological Quality of Systematic Reviews, axSpA = axial spondyloarthritis, BL = baseline, Blind. Asses. = Blinded assessors, Blind. Part. = blinded participants, CI = confidence interval, H/UC = high / unclear risk of bias, L = low risk of bias, MAF = Multidimensional Assessment of Fatigue, NRT = non-randomised trial, MD = mean difference, NHP = Nottingham Health Profile, Rand. Seq. = random sequence generation, RCT = randomised controlled trial, SD = standard deviation, SMD = standardised mean difference

Table – Home based exercises (axSpA), results and quality assessment

Outcome (outcome measure)	Study (date) [study type]	Standardised result, SMD (95% CI) unless otherwise stated	Natural result	AMSTAR2 quality	Rand. Seq.	Alloc. Conc.	Blind. Part.	Blind. Asses.
Anxiety	Aydin (2016) [RCT][198]	Home vs hospital based exercise at 8 weeks<br>SMD 0.55 (-0.11, 1.21)	HADS - anxiety, BL / 8 weeks, mean (SD)<br>Home based exercise: 8.84 (4.08) / 8.63 (4.23)<br>Hospital based exercise: 8.22 (4.90) / 6.50 (3.45)		L	H/UC	H/UC	H/UC
Depression	Liang (2015) [MA][192]		Depression, mean difference<br>MD -2.31 (-3.33, -1.30)	Moderate				
	Aydin (2016) [RCT][198]	Home vs hospital based exercise at 8 weeks<br>SMD 0.65 (-0.01, 1.32)	HADS - depression, BL / 8 weeks, mean (SD)<br>Home based exercise: 9.21 (4.57) / 9.47 (5.61)<br>Hospital based exercise: 7.66 (4.25) / 6.38 (3.58)		L	H/UC	H/UC	H/UC
	Lim (2005) [RCT][220]		Depression, % change BL-8 weeks<br>Exercise: -31%<br>Control: 19%		H/UC	H/UC	H/UC	L
	Analay (2003) [RCT][202]	Group vs home exercise<br>SMD -0.38 (-0.97, 0.21)	BDI, BL / 6 weeks / 3 months, mean (SD)<br>Group exercise: 5.52 (4.56) / 3.95 (3.21) / 5.13 (6.34)<br>Home exercise: 6.31 (4.72) / 5.90 (6.62) / 6.77 (6.41)		H/UC	L	H/UC	L
	Bespoke meta-analysis: Home vs group exercise [198;202]	Home vs group exercise<br>SMD 0.43 (-0.01, 0.87), I^2 0% in favour of group interventions						
	Yigit (2013) [NRT][221]	Muscle strengthening exercise vs control<br>SMD -0.59 (-1.22, 0.05)	BDI, BL / 10 weeks<br>Muscle strengthening exercise: 8.30 (7.27) / 5.75 (6.03)<br>Control: 11.15 (10.45) / 10.7 (10.33)					
	Durmus (2009) [NRT][222]	Muscle strengthening exercise vs control<br>SMD -1.71 (-2.42, -1.00)	BDI, BL / 12 weeks, mean (SD)<br>Muscle strengthening exercise: 9.24 (3.17) / 3.16 (2.07)<br>Control: 9.88 (3.35) / 7.05 (2.53)					
	Karapolat (2008) [NRT][224]	Home exercise vs group exercise at 6 weeks<br>SMD 0.11 (-0.53, 0.76)	BDI, BL / 6 weeks, mean (SD)<br>Home exercise: 8.5 (5.3) / 8.5 (6.0)<br>Group exercise: 8.0 (5.8) / 7.8 (6.4)					

Alloc. Conc. = allocation concealment, AMSTAR2 = Assessing the Methodological Quality of Systematic Reviews, axSpA = axial spondyloarthritis, BDI = Beck Depression Inventory, BL = baseline, Blind. Asses. = Blinded assessors, Blind. Part. = blinded participants, CI = confidence interval, H/UC = high / unclear risk of bias, L = low risk of bias, Rand. Seq. = random sequence generation, RCT = randomised controlled trial, SD = standard deviation, SMD = standardised mean difference

Table – Home based exercises (axSpA), results and quality assessment

Outcome (outcome measure)	Study (date) [study type]	Standardised result, SMD (95% CI) unless otherwise stated	Natural result	AMSTAR2 quality	Rand. Seq.	Alloc. Conc.	Blind. Part.	Blind. Asses.
Self-efficacy	Liang (2015) [MA][192]	Exercise vs control<br>SMD 0.07 (-0.25, 0.38)		Moderate				
	Sweeney (2002) [RCT][185]	Exercise vs control at 6 months<br>SMD 0.48 (0.20, 0.77)	Stanford self-efficacy - pain, BL / 6 months, mean (SD)<br>Exercise: 6.49 (1.8) / 6.80 (1.21)<br>Control: 6.06 (2.1) / 6.24 (1.1)		H/UC	H/UC	H/UC	H/UC
CRP	Aydin (2016) [RCT][198]	Home vs hospital based exercise at 8 weeks<br>SMD -0.48 (-1.14, 0.17)	CRP, BL / 8 weeks, mean (SD)<br>Home based exercise: 0.44 (0.54) / 0.32 (0.30)<br>Hospital based exercise: 1.25 (2.80) / 0.90 (1.69)		L	H/UC	H/UC	H/UC
	Hsieh (2014) [RCT][218]	Strength + range of motion vs range of motion only at 3 months<br>SMD -0.14 (-1.04, 0.77)	CRP, BL / 3 months, mean (SD)<br>Strength + range of motion: 1.27 (1.10) / 0.79 (0.56)<br>Range of motion only: 1.07 (1.24) / 0.9 (0.99)		L	L	H/UC	L
ESR	Aydin (2016) [RCT][198]	Home vs hospital based exercise, change BL-4 weeks<br>SMD -0.69 (-1.35, -0.02)	ESR, BL / 8 weeks, mean (SD)<br>Home based exercise: 21.52 (12.98) / 17.47 (8.59)<br>Hospital based exercise: 30.38 (21.74) / 26.38 (16.42)		L	H/UC	H/UC	H/UC
	Hsieh (2014) [RCT][218]	Strength + range of motion vs range of motion only at 3 months<br>SMD -0.01 (-0.91, 0.89)	ESR, BL / 3 months, mean (SD)<br>Strength + range of motion: 36.8 (28.6) / 24.8 (12.0)<br>Range of motion only: 24.7 (23.1) / 25.0 (28.3)		L	L	H/UC	L
Walk test	Durmus (2009) [NRT][223]	Muscle strengthening exercise vs conventional exercises<br>SMD -0.06 (-0.69, 0.58)<br>Muscle strengthening exercise vs control<br>SMD 0.88 (0.14, 1.62)	6 minute walk distance, BL / 12 weeks<br>Muscle strengthening exercise: 555.8 (91.0) / 620.4 (87.6)<br>Conventional exercise: 548.8 (82.4) / 625.0 (74.9)<br>Control: 537.4 (90.4) / 539.2 (99.9)					
Grip strength	Hsieh (2014) [RCT][218]	Strength + range of motion vs range of motion only at 3 months<br>SMD -0.06 (-0.96, 0.84)	Grip strength, BL / 3 months, mean (SD)<br>Strength + range of motion: 28.6 (11.0) / 30.5 (12.0)<br>Range of motion only: 29.5 (10.7) / 31.1 (9.2)		L	L	H/UC	L
Spinal flexion	Kraag (1990) [RCT][205]	Exercise vs control, change BL-4 months<br>SMD 0.04 (-0.50, 0.57)	Schober, Change BL-4 months, mean (SD)<br>Exercise: -2.4 (6.7)<br>Control: -2.7 (9.9)		H/UC	H/UC	H/UC	H/UC
	Kraag (1994) [Single arm int.][212]		Pain, 4 months / 8 months<br>Original exercise group: 13.4 (1.6) / 13.7 (1.5)<br>Original control group: 13.1 (1.5) / 13.2 (1.3)					

Alloc. Conc. = allocation concealment, AMSTAR2 = Assessing the Methodological Quality of Systematic Reviews, axSpA = axial spondyloarthritis, BL = baseline, Blind. Asses. = Blinded assessors, Blind. Part. = blinded participants, CI = confidence interval, CRP = C-reactive protein, ESR = erythrocyte sedimentation rate, H/UC = high / unclear risk of bias, L = low risk of bias, Rand. Seq. = random sequence generation, RCT = randomised controlled trial, SD = standard deviation, SMD = standardised mean difference

Table – Home exercises (axSpA), SF36 results at final follow-up, mean (SD)

Author (date)	PCS	MCS	GH	PF	RP	RE	SF	BP	V	MH
Yigit (2013) [Exercise][221]			71.45 (15.93)	73.50 (18.93)	85.00 (32.85)	84.99 (27.53)	90.00 (22.43)	73.35 (17.21)	70.00 (16.86)	83.00 (12.03)
Yigit (2013) [Control][221]			52.80 (23.36)	59.50 (25.95)	61.15 (39.30)	68.81 (30.41)	65.58 (31.71)	54.30 (27.49)	54.25 (21.11)	68.2 (19.05)
Durmus (2009) [Exercise][222]			0.72 (0.13)	0.86 (0.14)	0.77 (0.26)	0.80 (0.21)	0.81 (0.20)	0.78 (0.12)	0.80 (0.08)	0.80 (0.10)
Durmus (2009) [Control][222]			0.66 (0.19)	0.68 (0.20)	0.67 (0.22)	0.68 (0.62)	0.72 (0.19)	0.69 (0.18)	0.68 (0.21)	0.68 (0.18)

axSpA = axial spondyloarthritis, BP = bodily pain, GH = general health, MCS = mental component score, MH = mental health, PCS = physical component score, PF = physical function, RE = role emotional, RP = role physical, SD = standard deviation, SF = social functioning, V = vitality

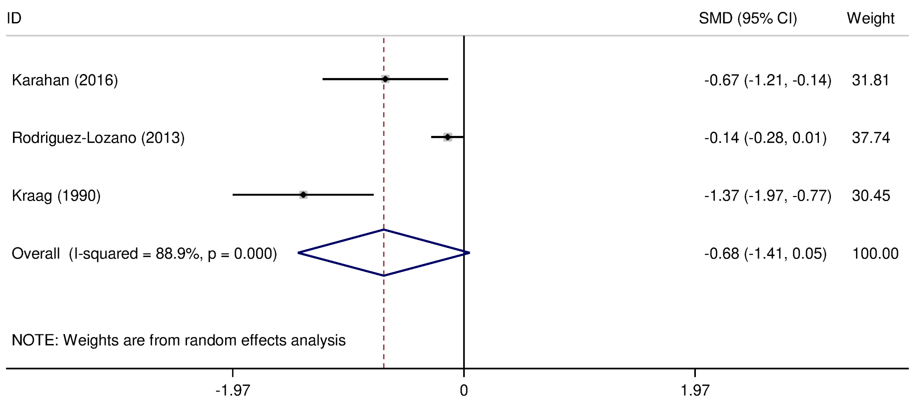

Figure – Comparing home exercise and controls in terms of pain (axSpA)

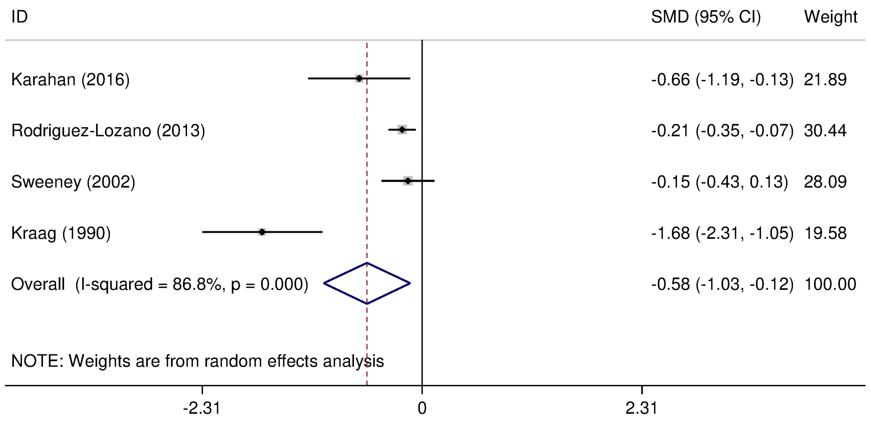

Figure – Comparing home exercise and controls in terms of function (axSpA)

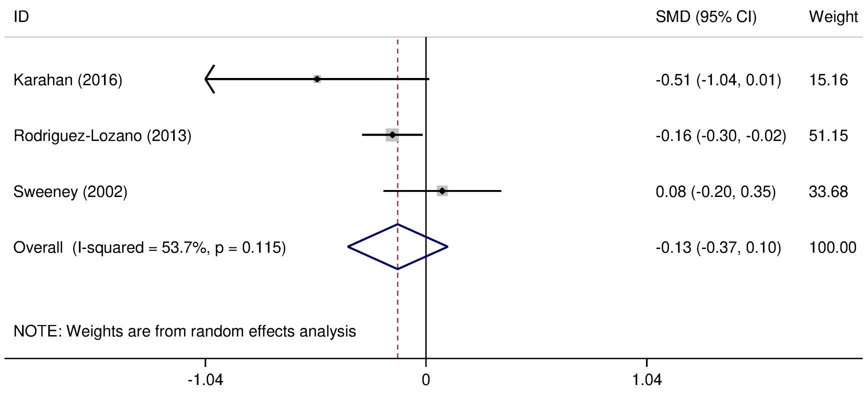

Figure – Comparing home exercise and controls in terms of disease activity (axSpA)

Supplementary table 73 – Description of original studies of muscle strengthening exercise in axSpA

Table – Muscle strengthening exercises (axSpA), description of included studies

Author (date) [country]	Study design	Inclusion criteria	Exposure detail	N	Age years, mean (SD)	N (%) female	Funders
de Souza (2017) [Brazil][225]	RCT	Modified New York criteria, aged 18-60 years, established diagnosis of AS, functional class I-II, stable DMARD dose for 3 months, stable NSAIDs / steroids for 4 months<br>Exclusions: hypertension, history of coronary artery disease, syncope or arrhythmia due to exercise, diabetes, severe psychiatric disorders, fibromyalgia, more disabling condition than AS, history of regular exercise ≥30mins for ≥2x per week in past 3 months, conditions preventing patients from exercising	1) Swiss ball training in groups with a physiotherapist<br>p) Usual care	1) 30<br>p) 30	1) 45 (9.8)<br>p) 43.8 (10.2)	1) 7 (23.3)<br>p) 9 (30.0)	**Government** (São Paulo Research Foundation)
Kasapoglu Aksoy (2017) [Turkey][226]	RCT	Modified New York criteria, No biologic treatment, necessary social and cognitive competence to be able to adjust to program<br>Exclusions: systemic disorder (cardiac, liver, kidney, blood disease), difficulty in co-operation	1) 5 day education program alongside stretching exercises<br>p) Routine care	1) 20<br>p) 21	1) 38.0 (9.8)<br>p) 37.5 (11.1)	1) 5 (25.0)<br>p) 4 (19.0)	**Not reported –** authors declared no conflict of interest
Rosu (2015) [Romania][227]	RCT	Modified New York criteria, early stage AS, radiological sacrolitiitis grade ≥2 without spinal involvement, clinically stable disease, no history of significant cardiovascular or respiratory comorbidity	1) McKenzie group – promote posture education, back stretching, respiratory re-education, pelvic stabilisation<br>2) Classic kinetic program – stretching and strengthening exercises to maintain function range or motion in spine	1) 26<br>2) 24	1) 25.1 (4.0)<br>p) 23.0 (3.7)	1) 4 (15.4)<br>2) 3 (12.5)	**Not reported –** authors declared no conflict of interest
Hsieh (2014) [Taiwan][218]	RCT	Modified New York criteria, aged 20-65 years, well controlled disease, symptom duration >6 months<br>Exclusions: presence of serious medical conditions or acute febrile disorders, history of arthroplasties or major operations in the knee or hip joint, severe arthritis or contracture of knee or hip joints which preclude exercise	1) Range of motion and strengthening exercises at home<br>2) Range of motion only at home	1) 9<br>2) 10	1) 36.2 (11.7)<br>2) 42.1 (8.8)	1) 3 (33.3)<br>2) 3 (30.0)	**Government** (Taiwan National Science Council)
Masiero (2014) [Italy][228]	RCT	12 month follow-up of Masiero et al (2011)[229]	12 month follow-up of Masiero et al (2011)[229]	1) 21<br>p1) 22<br>p1) 21	1) 49.1 (11.8)<br>p1) 43.9 (8.1)<br>p2) 46.2 (10.3)	1) 20%<br>p1) 5%<br>p2) 19.1%	**No funding**

axSpA = axial spondyloarthritis, DMARD = disease modifying anti-rheumatic drug, N = number, NSAIDs = non-steroidal anti-inflammatory drugs, RCT = randomised controlled trial, SD = standard deviation inhibitors,

Table – Muscle strengthening exercises (axSpA), description of included studies

Author (date) [country]	Study design	Inclusion criteria	Exposure detail	N	Age years, mean (SD)	N (%) female	Funders
Altan (2012) [Turkey][230]	RCT	AS modified New York Criteria<br>Exclusions: active peripheral arthritis, total spinal ankyloses, ESR over 50mm/h or CRP >10x normal value, treatment regime that has changed in past two months	1) Pilates, 1 hour 3x per week<br>p) Usual care	1) 29<br>p) 24	1) 46.5 (11.2)<br>p) 43.6 (10.1)	25 (47.2)	**Not reported –** Authors declared no conflicts of interest
Masiero (2011) [Italy][229]	RCT	New York criteria, treated with TNFi, did not require continuous NSAIDs, stable clinical picture (no change in BASDAI of more than 1/10 units in previous 3 months), aged 18-65 years, did not have severe disability presented no other osteoarticular disease.<br>Exclusions: Complete ankyloses of the spine, participation in rehabilitation treatment in previous 6 months, variations in standard biological therapy regimens	1) Educational behavioural program with exercise – Exercises included flexibility and strengthening exercises<br>p1) Educational program only<br>p2) No exercise control	1) 20<br>p1) 20<br>p2) 22	Median (IQR)<br>1) 47.5 (37.2, 61.5)<br>p1) 44.0 (38.2, 52.5)<br>p2) 47.5 (40.7, 52.5)	1) 5 (25.0)<br>p1) 4 (20.0)<br>p2) 4 (18.2)	**Not reported**
Fernandez-de-las-Penas (2006) [Spain][231]	RCT	New York criteria<br>Exclusions: medical condition that impaired function more than AS, osteoporosis, or a history of fractures secondary to osteoporosis	1) Strengthening and flexibility exercises using the Global Posture Re-education Method<br>2) Conventional exercise - Stretching and strengthening spine	1) 20<br>2) 20	1) 45 (9)<br>2) 46 (8)	1) 5 (25.0)<br>2) 4 (20.0)	**Not reported**
Fernandez-de-las-Penas (2005) [Spain][232]	RCT	New York criteria<br>Exclusions: medical condition that impaired function more than AS, osteoporosis, or a history of fractures secondary to osteoporosis	1) Strengthening and flexibility exercises using the Global Posture Re-education Method<br>2) Conventional exercise - Stretching and strengthening spine	1) 20<br>2) 20	1) 45 (9)<br>2) 46 (8)	1) 5 (25.0)<br>2) 4 (20.0)	**Not reported**
Lim (2005) [South Korea][220]	RCT	Outpatient without complications, sedentary, understands questionnaires, no changes in medications, functional class II	1) Home based exercise program – muscle relaxation, flexibility, muscular strength, breathing and posture.<br>p) Waitlist control	1) 25<br>p) 25	1) 38.8 (9.3)<br>p) 28.1 (7.5)	1) 6 (24.0)<br>p) 5 (20.0)	**Not reported**
Yigit (2013) [Turkey][221]	NRT	Aged 18-65 years, Modified New York Criteria, Received TNFi for ≥3 months, understand context of program<br>Exclusions: Severe comorbidities affecting heart, lung, liver or kidneys, mental retardation, presence of severe arthritis or prosthetic device and exercising regularly in past 6 months	1) Home based exercise program – 5x per week, muscle relaxation, flexibility, range of motion and strengthening exercises as well as exercises to improve posture<br>p) Group who did not do the exercise program	1) 20<br>p) 20	1) 40.3 (8.1)<br>p) 36.5 (7.2)	1) 5 (25.0)<br>p) 3 (15.0)	**Not reported –** authors declared no conflict of interest

axSpA = axial spondyloarthritis, BASDAI = Bath Ankylosing Spondylitis Disease Activity Index, ESR = erythrocyte sedimentation rate, N = number, NRT = non-randomised trial, NSAIDs = non-steroidal anti-inflammatory drugs, RCT = randomised controlled trial, SD = standard deviation, TNFi = tumour necrosis factor inhibitors,

Table – Muscle strengthening exercises (axSpA), description of included studies

Author (date) [country]	Study design	Inclusion criteria	Exposure detail	N	Age years, mean (SD)	N (%) female	Funders
Durmus (2009) [Turkey][222]	NRT	New York criteria, not been practicing regular exercise during previous 6 months, able to understand content of questionnaire / experimental schedules, no co-existent systemic diseases, not been given TNFi, functional class I-III	1) Home based exercise program – 7 days per week, 20 exercises for muscle relaxation, strength stronger breathing and straighter posture<br>p) Non-exercise control	1) 25<br>p) 18	1) 37.3 (7.3)<br>p) 42.3 (8.2)	1) 4 (16.0)<br>p) 4 (22.2)	**Not reported –** authors declared no conflict of interest
Durmus (2009) [Turkey][223]	NRT	New York criteria, sedentary<br>Exclusions: Medical condition that impaired function more than AS, those with co-existant cardiac or respiratory diseases, severe arthritis, taking TNFi	1) "Global Posture Re-education" - Muscle chains are shortened and then stretched and strengthened. Includes dynamic axial exercise, static posture exercise, stretching.<br>2) Conventional exercises – motion and flexibility<br>p) Non-exercise control	1) 19<br>2) 19<br>p) 13	1) 38.1 (11.1)<br>2) 35.9 (7.3)<br>p) 43.5 (7.3)	1) 5 (26.3)<br>2) 2 (10.5)<br>p) 1 (7.7)	**Not reported**
Gyurcsik (2012) [Hungary][233]	Single arm int.	Modified New York Criteria	Physical activity – 1.5 hours general posture re-educatoin, manual mobilization of the spine, pelvic-, upper-, and lower extremity exercises, stretching of the shortened muscles (mainly back, lumbar spine, hips, and shoulders) with joint prevention strategies, as well as functional exercises.	10	54.8 (14.9)	5 (50.0)	**Government** (Hungarian Medical Research Council), **University** (University of Debrecen)
Hulejova (2012) [Czech Republic][234]	Single arm int.	Modified New York Criteria	Physiotherapy – 45 mins 2x per week. Group based exercise consisting of stretching and muscle strengthening exercise and educated about home exercise	26	Median (IQR) 36 (22, 48)	8 (30.8)	MH CR & MSM [sic]
Ortancil (2009) [Turkey][235]	Single arm int.	Modified New York Criteria<br>Exclusions: cardiac and respiratory disease and significant pain in the hip, knee, ankle and feet	Breathing exercises and upper extremity exercises	22	42.4 (9.9)	5 (22.7)	**Not reported**

axSpA = axial spondyloarthritis, N = number, NRT = non-randomised trial, RCT = randomised controlled trial, SD = standard deviation, TNFi = tumour necrosis factor inhibitors

Supplementary table 74 – Results from reviews and interventional studies of muscle strengthening exercise in axSpA

Table – Muscle strengthening exercises (axSpA), results and quality assessment

Outcome (outcome measure)	Study (date) [study type]	Standardised result, SMD (95% CI) unless otherwise stated	Natural result	AMSTAR2 quality	Rand. Seq.	Alloc. Conc.	Blind. Part.	Blind. Asses.
Pain	Rosu (2015) [RCT][227]	McKenzie program vs classic kinetic exercises at 24 weeks<br>SMD -2.25 (-2.97, -1.54)	Pain VAS, BL / 24 weeks, mean (SD)<br>McKenzie: 32.31 (10.83) / 14.62 (5.82)<br>Classic Kinetic: 30.83 (10.60) / 28.33 (6.37)		H/UC	H/UC	H/UC	H/UC
	Masiero (2014) [RCT][228]	Exercise + education vs education at 6 months<br>Cervical pain: SMD -0.52 (-1.13, 0.09)<br>Lumbar pain: SMD -0.73 (-1.35, -0.10)<br>Exercise + education vs control at 6 months<br>Cervical pain: SMD -0.58 (-1.19, 0.04)<br>Lumbar pain: SMD -0.37 (-0.98, 0.25)	Pain VAS – cervical BL / 12 months, mean (SD)<br>Exercise + education: 33.3 (22.4) / 8.1 (8.9)<br>Education: 22.4 (26.0) / 12.5 (8.0)<br>Control: 33.7 (26.1) / 20.6 (22.7)<br>Pain VAS – lumbar BL / 12 months, mean (SD)<br>Exercise + education: 35.0 (25.0) / 11.6 (15.8)<br>Education: 29.5 (29.2) / 26.7 (33.3)<br>Control: 26.5 (24.1) / 18.4 (21.1)		L	L	H/UC	L
	Masiero (2011) [RCT][229]	Exercise + education vs education at 6 months<br>Cervical pain: SMD -0.45 (-1.08, 0.18)<br>Lumbar pain: SMD -0.47 (-1.10, 0.16)<br>Exercise + education vs control at 6 months<br>Cervical pain: SMD -1.53 (-2.22, -0.83)<br>Lumbar pain: SMD -1.38 (-2.06, -0.70)	Pain VAS – cervical BL / 6 months, mean (SD †)<br>Exercise + education: 30.4 (30.5) / 7.7 (10.4)<br>Education: 27.5 (28.7) / 16.0 (23.9)<br>Control: 26.4 (33.1) / 28.3 (15.8)<br>Pain VAS – lumbar BL / 6 months, mean (SD †)<br>Exercise + education: 33.2 (40.3) / 9.2 (16.0)<br>Education: 26.5 (31.9) / 19.6 (26.9)<br>Control: 23.2 (28.1) / 32.0 (17.0)		L	L	H/UC	L
	Lim (2005) [RCT][220]		Pain, % change BL-8 weeks<br>Exercise: -33%<br>Control: 28%		H/UC	H/UC	H/UC	L
	Bespoke meta-analysis including[227;229]	Exercise vs control<br>SMD -1.88 (-2.59, -1.17), I^2 51.2%						
	Durmus (2009) [NRT][223]	Muscle strengthening exercise vs conventional exercises<br>SMD -0.29 (-0.93, 0.35)<br>Muscle strengthening exercise vs control<br>SMD -0.78 (-1.51, -0.05)	Pain VAS, BL / 12 weeks<br>Muscle strengthening exercise: 4.78 (2.63) / 1.42 (1.80)<br>Conventional exercise: 4.84 (2.81) / 1.94 (1.80)<br>Control: 3.46 (2.40) / 3.00 (2.34)					
	Gyurcsik (2012) [Single arm int.][233]		Pain VAS, BL / 3 months, mean (SD)<br>41.1 (24.77) / 24 (21.16)					

† Mean (SD) estimated from median (interquartile range) using published formula[87]

Alloc. Conc. = allocation concealment, AMSTAR2 = Assessing the Methodological Quality of Systematic Reviews, axSpA = axial spondyloarthritis, BL = baseline, Blind. Asses. = Blinded assessors, Blind. Part. = blinded participants, CI = confidence interval, H/UC = high / unclear risk of bias, L = low risk of bias, Rand. Seq. = random sequence generation, RCT = randomised controlled trial, SD = standard deviation, SMD = standardised mean difference, VAS = visual analogue scale

Table – Muscle strengthening exercises (axSpA), results and quality assessment

Outcome (outcome measure)	Study (date) [study type]	Standardised result, SMD (95% CI) unless otherwise stated	Natural result	AMSTAR2 quality	Rand. Seq.	Alloc. Conc.	Blind. Part.	Blind. Asses.
Function	de Souza (2017) [RCT][225]	Exercise vs control at 16 weeks<br>BASFI - SMD -0.23 (-0.73, 0.28)<br>HAQ-S – SMD -0.26 (-0.76, 0.25)	BASFI, BL / 16 weeks, mean (SD)<br>Exercise: 4.62 (2.49) / 3.36 (2.16)<br>Control: 4.09 (2.40) / 3.90 (2.6)<br>HAQ-S, BL / 16 weeks, mean (SD)<br>Exercise: 0.79 (0.51) / 0.58 (0.44)<br>Control: 0.75 (0.53) / 0.70 (0.5)		L	L	H/UC	L
	Kasapoglu Aksoy (2017) [RCT][226]	Exercise vs control at 3 months<br>SMD -0.19 (-0.80, 0.43)	BASFI, BL / 3 months, mean (SD)<br>Exercise: 2.88 (1.98) / 1.76 (1.47)<br>Control: 2.17 (2.37) / 2.12 (2.26)		H/UC	H/UC	H/UC	H/UC
	Rosu (2015) [RCT][227]	McKenzie program vs classic kinetic exercises at 24 weeks<br>SMD -0.58 (-1.15, -0.01)	BASFI, BL / 24 weeks, mean (SD)<br>McKenzie: 3.44 (1.93) / 1.25 (0.90)<br>Classic Kinetic: 3.11 (1.78) / 2.04 (1.73)		H/UC	H/UC	H/UC	H/UC
	Hsieh (2014) [RCT][218]	Strength + range of motion vs range of motion only at 3 months<br>SMD -0.58 (-1.50, 0.34)	BASFI, BL / 3 months, mean (SD)<br>Strength + range of motion: 3.7 (3.3) / 1.9 (2.3)<br>Range of motion only: 3.5 (2.9) / 3.5 (3.1)		L	L	H/UC	L
	Masiero (2014) [RCT][228]	Exercise + education vs education at 6 months<br>SMD -0.10 (-0.70, 0.50)<br>Exercise + education vs control at 6 months<br>SMD -0.47 (-1.09, 0.14)	BASFI, BL / 12 months, mean (SD)<br>Exercise + education: 3.0 (1.5) / 2.2 (1.3)<br>Education: 2.7 (1.6) / 2.4 (2.4)<br>Control: 2.9 (1.7) / 3.0 (2.0)		L	L	H/UC	L
	Altan (2012) [RCT][230]	Exercise vs control at 12 weeks<br>BASFI: SMD -0.37 (-0.91, 0.18)	BASFI, BL / 12 weeks / 24 weeks, mean (SD)<br>Exercise: 2.4 (1.6) / 1.7 (1.6) / 1.7 (1.6)<br>Control: 2.2 (1.6) / 2.3 (1.7) / 2.3 (2.1)		L	H/UC	H/UC	L
	Masiero (2011) [RCT][229]	Exercise + education vs education at 6 months<br>SMD -0.08 (-0.70, 0.55)<br>Exercise + education vs control at 6 months<br>SMD -0.84 (-1.47, -0.21)	BASFI, BL / 6 months, mean (SD †)<br>Exercise + education: 3.1 (2.5) / 1.3 (1.0)<br>Education: 2.5 (1.8) / 1.4 (1.6)<br>Control: 3.1 (2.1) / 2.7 (2.1)		L	L	H/UC	L

Alloc. Conc. = allocation concealment, AMSTAR2 = Assessing the Methodological Quality of Systematic Reviews, axSpA = axial spondyloarthritis, BASFI = Bath Ankylosing Spondylitis Functional Index, BL = baseline, Blind. Asses. = Blinded assessors, Blind. Part. = blinded participants, CI = confidence interval, H/UC = high / unclear risk of bias, HAQ-S = Health Assessment Questionnaire for spondyloarthritis, L = low risk of bias, Rand. Seq. = random sequence generation, RCT = randomised controlled trial, SD = standard deviation, SMD = standardised mean difference

Table – Muscle strengthening exercises (axSpA), results and quality assessment

Outcome (outcome measure)	Study (date) [study type]	Standardised result, SMD (95% CI) unless otherwise stated	Natural result	AMSTAR2 quality	Rand. Seq.	Alloc. Conc.	Blind. Part.	Blind. Asses.
Function	Fernandez-de-las-Penas (2006) [RCT][231]	Exercise vs control at 12 months<br>SMD -0.07 (-0.69, 0.55)	BASFI, BL / 12 months, mean (SD)<br>Interventional exercise: 51.8 (20.8) / 46.7 (19.9)<br>Control exercise: 47 (19) / 48 (19.4)		L	H/UC	H/UC	L
	Fernandez-de-las-Penas (2005) [RCT][232]	Exercise vs control at 4 months<br>SMD -0.04 (-0.67, 0.58)	BASFI, BL / 4 months, mean (SD)<br>Interventional exercise: 51.8 (20.8) / 45.7 (20.6)<br>Control exercise: 47 (19) / 46.5 (21)		L	H/UC	H/UC	L
	Lim (2005) [RCT][220]		Functional capacity, % change BL-8 weeks<br>Exercise: 46%<br>Control: unchanged		H/UC	H/UC	H/UC	L
	Bespoke meta-analysis including[225-227;229;230;232]	Exercise vs control<br>SMD -0.36 (-0.60, -0.13), I^2 0%						
	Yigit (2013) [NRT][221]	Muscle strengthening exercise vs control<br>SMD -0.77 (-1.41, -0.12)	BASFI, BL / 10 weeks<br>Muscle strengthening exercise: 3.22 (2.96) / 2.27 (2.10)<br>Control: 3.86 (2.36) / 4.00 (2.41)					
	Durmus (2009) [NRT][222]	Muscle strengthening exercise vs control<br>SMD -0.89 (-1.53, -0.25)	BASFI, BL / 12 weeks, mean (SD)<br>Muscle strengthening exercise: 2.22 (1.53) / 1.25 (1.07)<br>Control: 2.55 (1.40) / 2.30 (1.32)					
	Durmus (2009) [NRT][223]	Muscle strengthening exercise vs conventional exercises<br>SMD -0.38 (-1.02, 0.26)<br>Muscle strengthening exercise vs control<br>SMD -0.82 (-1.56, -0.09)	BASFI, BL / 12 weeks<br>Muscle strengthening exercise: 2.65 (2.39) / 1.28 (1.47)<br>Conventional exercise: 3.18 (2.05) / 1.83 (1.41)<br>Control: 3.32 (2.59) / 2.97 (2.71)					
	Gyurcsik (2012) [Single arm int.][233]		BASFI, BL / 3 months, mean (SD)<br>4.33 (2.61) / 3.81 (2.71)					
	Hulejova (2012) [Single arm int.][234]		BASFI, BL / 3 months, mean (SD)<br>2.31 (1.92) / 1.37 (1.34)					
	Ortancil (2009) [Single arm int.][235]		BASFI, BL / 6 weeks, mean (SD)<br>2.9 (2.0) / 2.7 (2.0)					

Alloc. Conc. = allocation concealment, AMSTAR2 = Assessing the Methodological Quality of Systematic Reviews, axSpA = axial spondyloarthritis, BASFI = Bath Ankylosing Spondylitis Functional Index, BL = baseline, Blind. Asses. = Blinded assessors, Blind. Part. = blinded participants, CI = confidence interval, H/UC = high / unclear risk of bias, L = low risk of bias, NRT = non-randomised trial, Rand. Seq. = random sequence generation, RCT = randomised controlled trial, SD = standard deviation, SMD = standardised mean difference

Table – Muscle strengthening exercises (axSpA), results and quality assessment

Outcome (outcome measure)	Study (date) [study type]	Standardised result, SMD (95% CI) unless otherwise stated	Natural result	AMSTAR2 quality	Rand. Seq.	Alloc. Conc.	Blind. Part.	Blind. Asses.
Disease activity	de Souza (2017) [RCT][225]	Exercise vs control at 16 weeks<br>BASDAI - SMD -0.02 (-0.53, 0.49)<br>ASDAS-CRP – SMD 0.07 (-0.44, 0.58)<br>ASSDAS-ESR – SMD -0.42 (-0.93, 0.10)	BASDAI, BL / 16 weeks, mean (SD)<br>Exercise: 2.52 (1.65) / 2.08 (1.84)<br>Control: 2.34 (2.26) / 2.12 (2.4)<br>ASDAS-CRP, BL / 16 weeks, mean (SD)<br>Exercise: 2.20 (0.91) / 1.93 (0.84)<br>Control: 1.89 (1.00) / 1.86 (1.10)<br>ASDAS-ESR, BL / 16 weeks, mean (SD)<br>Exercise: 2.23 (0.87) / 1.73 (0.70)<br>Control: 2.11 (0.97) / 2.15 (1.25)		L	L	H/UC	L
	Kasapoglu Aksoy (2017) [RCT][226]	Exercise vs control at 3 months<br>SMD 0.00 (-0.61, 0.61)	BASDAI, BL / 3 months, mean (SD)<br>Exercise: 3.52 (1.55) / 2.74 (1.43)<br>Control: 2.93 (2.12) / 2.74 (1.69)		H/UC	H/UC	H/UC	H/UC
	Rosu (2015) [RCT][227]	McKenzie program vs classic kinetic exercises at 24 weeks<br>SMD -1.10 (-1.70, -0.50)	BASDAI, BL / 24 weeks, mean (SD)<br>McKenzie: 4.98 (1.83) / 1.91 (0.75)<br>Classic Kinetic: 4.98 (1.65) / 3.48 (1.91)		H/UC	H/UC	H/UC	H/UC
	Hsieh (2014) [RCT][218]	Strength + range of motion vs range of motion only at 3 months<br>SMD -0.32 (-1.23, 0.59)	BASDAI, BL / 3 months, mean (SD)<br>Strength + range of motion: 4.2 (1.9) / 3.7 (1.8)<br>Range of motion only: 4.5 (2.1) / 4.5 (3.0)		L	L	H/UC	L
	Masiero (2014) [RCT][228]	Exercise + education vs education at 6 months<br>SMD -0.34 (-0.94, 0.26)<br>Exercise + education vs control at 6 months<br>SMD -0.55 (-1.17, 0.06)	BASDAI, BL / 12 months, mean (SD)<br>Exercise + education: 3.8 (1.6) / 2.2 (1.3)<br>Education: 2.9 (1.2) / 2.8 (2.1)<br>Control: 3.1 (1.7) / 3.2 (2.2)		L	L	H/UC	L
	Altan (2012) [RCT][230]	Exercise vs control at 12 weeks<br>SMD -0.54 (-1.09, 0.02)	BASDAI, BL / 12 weeks / 24 weeks, mean (SD)<br>Exercise: 2.8 (1.7) / 2.1 (2 [sic]) / 2.4 (1.7)<br>Control: 2.6 (1.8) / 3.1 (1.7) / 3.1 (2 [sic])		L	H/UC	H/UC	L
	Masiero (2011) [RCT][229]	Exercise + education vs education at 6 months<br>SMD -0.33 (-0.96, 0.29)<br>Exercise + education vs control at 6 months<br>SMD -0.56 (-1.18, 0.06)	BASDAI, BL / 6 months, mean (SD †)<br>Exercise + education: error in paper / 2.0 (2.0)<br>Education: 3.8 (3.4) / 2.7 (2.2)<br>Control: 3.1 (2.1) / 3.3 (2.6)		L	L	H/UC	L

Alloc. Conc. = allocation concealment, AMSTAR2 = Assessing the Methodological Quality of Systematic Reviews, ASDAS = Ankylosing spondylitis disease activity index, axSpA = axial spondyloarthritis, BASDAI = Bath Ankylosing Spondylitis Disease Activity Index, BL = baseline, Blind. Asses. = Blinded assessors, Blind. Part. = blinded participants, CI = confidence interval, CRP = C-reactive protein, ESR = erythrocyte sedimentation rate, H/UC = high / unclear risk of bias, L = low risk of bias, Rand. Seq. = random sequence generation, RCT = randomised controlled trial, SD = standard deviation, SMD = standardised mean difference

Table – Muscle strengthening exercises (axSpA), results and quality assessment

Outcome (outcome measure)	Study (date) [study type]	Standardised result, SMD (95% CI) unless otherwise stated	Natural result	AMSTAR2 quality	Rand. Seq.	Alloc. Conc.	Blind. Part.	Blind. Asses.
Disease activity	Fernandez-de-las-Penas (2006) [RCT][231]	Exercise vs control at 12 months<br>SMD -0.12 (-0.74, 0.50)	BASDAI, BL / 12 months, mean (SD)<br>Interventional exercise: 27.6 (9.1) / 26.8 (11.3)<br>Control exercise: 28.5 (10) / 28 (8.9)		L	H/UC	H/UC	L
	Fernandez-de-las-Penas (2005) [RCT][232]	Exercise vs control at 4 months<br>SMD -0.02 (-0.64, 0.60)	BASDAI, BL / 4 months, mean (SD)<br>Interventional exercise: 27.6 (9.1) / 26 (11.3)<br>Control exercise: 28.5 (10) / 26.2 (8.6)		L	H/UC	H/UC	L
	Bespoke meta-analysis including[225-227;229;230;232]	Exercise vs control<br>-0.37 (-0.72, -0.02), I^2 54.1%						
	Yigit (2013) [NRT][221]	Muscle strengthening exercise vs control<br>SMD -0.55 (-1.19, 0.08)	BASDAI, BL / 10 weeks<br>Muscle strengthening exercise: 3.85 (2.45) / 2.61 (1.83)<br>Control: 3.81 (2.38) / 3.77 (2.33)					
	Durmus (2009) [NRT][222]	Muscle strengthening exercise vs control<br>SMD -1.12 (-1.77, -0.47)	BASDAI, BL / 12 weeks, mean (SD)<br>Muscle strengthening exercise: 2.52 (1.25) / 1.35 (0.78)<br>Control: 2.63 (1.07) / 2.34 (1.01)					
	Durmus (2009) [NRT][223]	Muscle strengthening exercise vs conventional exercises<br>SMD -0.20 (-0.84, 0.44)<br>Muscle strengthening exercise vs control<br>SMD -0.66 (-1.38, 0.07)	BASDAI, BL / 12 weeks<br>Muscle strengthening exercise: 2.73 (1.31) / 1.33 (0.88)<br>Conventional exercise: 2.96 (1.21) / 1.50 (0.83)<br>Control: 2.50 (1.31) / 2.08 (1.44)					
	Gyurcsik (2012) [Single arm int.][233]		BASDAI, BL / 3 months, mean (SD)<br>4.36 (2.62) / 3.94 (2.6)<br>Disease activity VAS, BL / 3 months, mean (SD)<br>38.7 (24.66) / 26.7 (23.61)					
	Hulejova (2012) [Single arm int.][234]		BASDAI, BL / 3 months, mean (SD)<br>2.98 (1.84) / 1.80 (1.43)					

Alloc. Conc. = allocation concealment, AMSTAR2 = Assessing the Methodological Quality of Systematic Reviews, axSpA = axial spondyloarthritis, BASDAI = Bath Ankylosing Spondylitis Disease Activity Index, BL = baseline, Blind. Asses. = Blinded assessors, Blind. Part. = blinded participants, CI = confidence interval, H/UC = high / unclear risk of bias, L = low risk of bias, NRT = non-randomised trial, Rand. Seq. = random sequence generation, RCT = randomised controlled trial, SD = standard deviation, SMD = standardised mean difference

Table – Muscle strengthening exercises (axSpA), results and quality assessment

Outcome (outcome measure)	Study (date) [study type]	Standardised result, SMD (95% CI) unless otherwise stated	Natural result	AMSTAR2 quality	Rand. Seq.	Alloc. Conc.	Blind. Part.	Blind. Asses.
Spinal mobility	de Souza (2017) [RCT][225]	Exercise vs control at 16 weeks<br>SMD -0.33 (-0.84, 0.18)	BASMI, BL / 16 weeks, mean (SD)<br>Exercise: 4.94 (2.09) / 4.69 (1.94)<br>Control: 5.19 (2.04) / 5.37 (2.2)		L	L	H/UC	L
	Kasapoglu Aksoy (2017) [RCT][226]	Exercise vs control at 3 months<br>SMD 0.13 (-0.49, 0.74)	BASMI, BL / 3 months, mean (SD)<br>Exercise: 0.62 (0.43) / 0.59 (0.41)<br>Control: 0.54 (0.35) / 0.54 (0.37)		H/UC	H/UC	H/UC	H/UC
	Rosu (2015) [RCT][227]	McKenzie program vs classic kinetic exercises at 24 weeks<br>SMD -3.52 (-4.42, -2.62)	BASMI, BL / 24 weeks, mean (SD)<br>McKenzie: 3.58 (0.50) / 0.77 (0.82)<br>Classic Kinetic: 3.58 (0.50) / 3.08 (0.41)		H/UC	H/UC	H/UC	H/UC
	Masiero (2014) [RCT][228]	Exercise + education vs education at 6 months<br>SMD 0.11 (-0.49, 0.71)<br>Exercise + education vs control at 6 months<br>SMD -0.20 (-0.81, 0.41)	BASMI, BL / 12 months, mean (SD)<br>Exercise + education: 4.7 (1.1) / 3.8 (1.4)<br>Education: 3.8 (1.1) / 3.6 (2.1)<br>Control: 4.0 (1.3) / 4.10 (1.6)		L	L	H/UC	L
	Masiero (2011) [RCT][229]	Exercise + education vs education at 6 months<br>SMD -0.17 (-0.79, 0.45)<br>Exercise + education vs control at 6 months<br>SMD -0.61 (-1.23, 0.01)	BASMI, BL / 6 months, mean (SD †)<br>Exercise + education: 4.6 (2.2) / 3.1 (1.1)<br>Education: 3.8 (2.0) / 3.4 (2.2)<br>Control: 4.0 (2.1) / 4.3 (2.5)		L	L	H/UC	L
	Altan (2012) [RCT][230]	Exercise vs control at 12 weeks<br>SMD -0.16 (-0.70, 0.38)	BASMI, BL / 12 weeks / 24 weeks, mean (SD)<br>Exercise: 8.8 (1.8) / 8.4 (1.9) / 8.4 (1.8)<br>Control: 8.9 (1.7) / 8.7 (1.8) / 9.1 (1.7)		L	H/UC	H/UC	L
	Bespoke meta-analysis including[225-227;229;230]	Exercise vs control<br>SMD -0.85 (-1.81, 0.12), I^2 91.9%<br>Removing outlier[227]: SMD -0.24 (-0.53, 0.04), I^2 0%						
	Yigit (2013) [NRT][221]	Muscle strengthening exercise vs control<br>SMD -0.60 (-1.24, 0.03)	BASMI, BL / 10 weeks<br>Muscle strengthening exercise: 5.05 (2.74) / 4.15 (2.62)<br>Control: 5.55 (2.50) / 5.70 (2.52)					

† Mean (SD) estimated from median (interquartile range) using published formula[87]

Alloc. Conc. = allocation concealment, AMSTAR2 = Assessing the Methodological Quality of Systematic Reviews, axSpA = axial spondyloarthritis, BASMI = Bath Ankylosing Spondylitis Metrology Index, BL = baseline, Blind. Asses. = Blinded assessors, Blind. Part. = blinded participants, CI = confidence interval, H/UC = high / unclear risk of bias, L = low risk of bias, Rand. Seq. = random sequence generation, RCT = randomised controlled trial, SD = standard deviation, SMD = standardised mean difference

Table – Muscle strengthening exercises (axSpA), results and quality assessment

Outcome (outcome measure)	Study (date) [study type]	Standardised result, SMD (95% CI) unless otherwise stated	Natural result	AMSTAR2 quality	Rand. Seq.	Alloc. Conc.	Blind. Part.	Blind. Asses.
Stiffness	Masiero (2014) [RCT][228]	Exercise + education vs education at 6 months<br>SMD -0.37 (-0.97, 0.24)<br>Exercise + education vs control at 6 months<br>SMD -0.43 (-1.04, 0.19)	Morning stiffness (0-10), BL / 12 months, mean (SD)<br>Exercise + education: 31.4 (17.8) / 18.9 (8.3)<br>Education: 27.6 (19.2) / 24.8 (21.0)<br>Control: 23.8 (19.8) / 26.5 (23.8)		L	L	H/UC	L
	Masiero (2011) [RCT][229]	Exercise + education vs education at 6 months<br>SMD -0.30 (-0.93, 0.32)<br>Exercise + education vs control at 6 months<br>SMD -0.31 (-0.92, 0.30)	Morning stiffness (0-10), BL / 6 months, mean (SD †)<br>Exercise + education: 2.6 (1.9) / 1.7 (2.2)<br>Education: 2.6 (2.7) / 2.4 (2.4)<br>Control: 2.5 (3.6) / 2.5 (2.9)		L	L	H/UC	L
Patient global	Kasapoglu Aksoy (2017) [RCT][226]	Exercise vs control at 3 months<br>SMD -0.21 (-0.82, 0.41)	BAS-G, BL / 3 months, mean (SD)<br>Exercise: 6.25 (1.68) / 4.70 (2.10)<br>Control: 5.19 (1.83) / 5.14 (2.12)		H/UC	H/UC	H/UC	H/UC
	Hsieh (2014) [RCT][218]	Strength + range of motion vs range of motion only at 3 months<br>SMD -0.17 (-1.08, 0.73)	BAS-G, BL / 3 months, mean (SD)<br>Strength + range of motion: 5.6 (2.7) / 3.6 (2.0)<br>Range of motion only: 5.0 (2.8) / 4.1 (3.5)		L	L	H/UC	L
Quality of life	Kasapoglu Aksoy (2017) [RCT][226]	Exercise vs control at 3 months<br>SMD 0.04 (-0.57, 0.66)	ASQOL, BL / 3 months, mean (SD)<br>Exercise: 8.3 (4.4) / 5.3 (3.3)<br>Control: 5.3 (5.5) / 5.1 (5.5)		H/UC	H/UC	H/UC	H/UC
	Altan (2012) [RCT][230]	Exercise vs control at 12 weeks<br>SMD 0.19 (-0.35, 0.73)	ASQOL, BL / 12 weeks / 24 weeks, mean (SD)<br>Exercise: 3.7 (4.6) / 4 (4.9) / 4 (4.8)<br>Control: 3.5 (3.3) / 3.2 (3.2) / 3 (3.4)		L	H/UC	H/UC	L
	Bespoke meta-analysis including[226;230]	Exercise vs control<br>SMD 0.13 (-0.28, 0.53), I^2 0%						

† Mean (SD) estimated from median (interquartile range) using published formula[87]

Alloc. Conc. = allocation concealment, AMSTAR2 = Assessing the Methodological Quality of Systematic Reviews, axSpA = axial spondyloarthritis, ASQOL = Ankylosing Spondylitis Quality of Life, BAS-G = Bath Ankylosing Spondylitis Global Score, BL = baseline, Blind. Asses. = Blinded assessors, Blind. Part. = blinded participants, CI = confidence interval, H/UC = high / unclear risk of bias, L = low risk of bias, Rand. Seq. = random sequence generation, RCT = randomised controlled trial, SD = standard deviation, SMD = standardised mean difference

Table – Muscle strengthening exercises (axSpA), results and quality assessment

Outcome (outcome measure)	Study (date) [study type]	Standardised result, SMD (95% CI) unless otherwise stated	Natural result	AMSTAR2 quality	Rand. Seq.	Alloc. Conc.	Blind. Part.	Blind. Asses.
Fatigue	Masiero (2014) [RCT][228]	Exercise + education vs education at 6 months<br>SMD -0.55 (-1.16, 0.06)<br>Exercise + education vs control at 6 months<br>SMD -0.68 (-1.30, -0.06)	Fatigue VAS, BL / 12 months, mean (SD)<br>Exercise + education: 4.6 (2.3) / 2.6 (1.7)<br>Education: 4.1 (2.4) / 3.4 (2.2)<br>Control: 3.0 (2.8) / 3.7 (2.5)		L	L	H/UC	L
	Masiero (2011) [RCT][229]	Exercise + education vs education at 6 months<br>SMD -0.55 (-1.18, 0.08)<br>Exercise + education vs control at 6 months<br>SMD -0.69 (-1.32, -0.07)	Fatigue VAS, BL / 6 months, mean (SD †)<br>Exercise + education: 4.2 (2.6) / 2.1 (2.2)<br>Education: 4.3 (3.4) / 3.4 (2.5)<br>Control: 3.1 (3.0) / 3.7 (2.4)		L	L	H/UC	L
	Yigit (2013) [NRT][221]	Muscle strengthening exercise vs control<br>SMD -0.68 (-1.32, -0.04)	MAF, BL / 10 weeks<br>Muscle strengthening exercise: 21.48 (12.62) / 15.95 (11.52)<br>Control: 24.85 (13.60) / 24.42 (13.38)					
	Durmus (2009) [NRT][222]	Muscle strengthening exercise vs control<br>SMD -0.45 (-1.06, 0.16)	MAF, BL / 12 weeks, mean (SD)<br>Muscle strengthening exercise: 2.07 (0.77) / 1.30 (1.08)<br>Control: 2.02 (0.76) / 1.73 (0.74)					
Depression	Lim (2005) [RCT][220]		Depression, % change BL-8 weeks<br>Exercise: -31%<br>Control: 19%		H/UC	H/UC	H/UC	L
	Yigit (2013) [NRT][221]	Muscle strengthening exercise vs control<br>SMD -0.59 (-1.22, 0.05)	BDI, BL / 10 weeks<br>Muscle strengthening exercise: 8.30 (7.27) / 5.75 (6.03)<br>Control: 11.15 (10.45) / 10.7 (10.33)					
	Durmus (2009) [NRT][222]	Muscle strengthening exercise vs control<br>SMD -1.71 (-2.42, -1.00)	BDI, BL / 12 weeks, mean (SD)<br>Muscle strengthening exercise: 9.24 (3.17) / 3.16 (2.07)<br>Control: 9.88 (3.35) / 7.05 (2.53)					

† Mean (SD) estimated from median (interquartile range) using published formula[87]

Alloc. Conc. = allocation concealment, AMSTAR2 = Assessing the Methodological Quality of Systematic Reviews, axSpA = axial spondyloarthritis, BDI = Beck Depression Inventory, BL = baseline, Blind. Asses. = Blinded assessors, Blind. Part. = blinded participants, CI = confidence interval, H/UC = high / unclear risk of bias, L = low risk of bias, MAF = Multidimensional Assessment of Fatigue, Rand. Seq. = random sequence generation, RCT = randomised controlled trial, SD = standard deviation, SMD = standardised mean difference

Table – Muscle strengthening exercises (axSpA), results and quality assessment

Outcome (outcome measure)	Study (date) [study type]	Standardised result, SMD (95% CI) unless otherwise stated	Natural result	AMSTAR2 quality	Rand. Seq.	Alloc. Conc.	Blind. Part.	Blind. Asses.
CRP	de Souza (2017) [RCT][225]	Exercise vs control at 16 weeks<br>SMD 0.45 (-0.07, 0.96)	CRP, BL / 16 weeks, mean (SD)<br>Exercise: 6.53 (6.00) / 9.27 (13.50)<br>Control: 4.70 (5.96) / 4.51 (6.75)		L	L	H/UC	L
	Kasapoglu Aksoy (2017) [RCT][226]	Exercise vs control at 3 months<br>SMD 0.10 (-0.51, 0.71)	CRP, BL / 3 months, mean (SD)<br>Exercise: 1.40 (1.47) / 1.22 (1.34)<br>Control: 1.30 (1.67) / 1.09 (1.28)		H/UC	H/UC	H/UC	H/UC
	Hsieh (2014) [RCT][218]	Strength + range of motion vs range of motion only at 3 months<br>SMD -0.14 (-1.04, 0.77)	CRP, BL / 3 months, mean (SD)<br>Strength + range of motion: 1.27 (1.10) / 0.79 (0.56)<br>Range of motion only: 1.07 (1.24) / 0.9 (0.99)		L	L	H/UC	L
	Bespoke meta-analysis including[225;226]	Exercise vs control<br>SMD 0.30 (-0.09, 0.70), I^2 0%						
	Hulejova (2012) [Single arm int.][234]		CRP, BL / 3 months, mean (SD)<br>8.14 (8.98) / 6.58 (7.93)					
ESR	de Souza (2017) [RCT][225]	Exercise vs control at 16 weeks<br>SMD -0.45 (-0.96, 0.06)	ESR, BL / 16 weeks, mean (SD)<br>Exercise: 18.10 (13.23) / 13.31 (9.01)<br>Control: 18.00 (12.27) / 18.71 (14.33)		L	L	H/UC	L
	Kasapoglu Aksoy (2017) [RCT][226]	Exercise vs control at 3 months<br>SMD -0.17 (-0.78, 0.45)	ESR, BL / 3 months, mean (SD)<br>Exercise: 14.60 (9.83) / 11.85 (7.59)<br>Control: 12.4 (11.40) / 13.42 (10.9)		H/UC	H/UC	H/UC	H/UC
	Hsieh (2014) [RCT][218]	Strength + range of motion vs range of motion only at 3 months<br>SMD -0.01 (-0.91, 0.89)	ESR, BL / 3 months, mean (SD)<br>Strength + range of motion: 36.8 (28.6) / 24.8 (12.0)<br>Range of motion only: 24.7 (23.1) / 25.0 (28.3)		L	L	H/UC	L
	Bespoke meta-analysis including[225;226]	Exercise vs control<br>SMD -0.33 (-0.73, 0.06), I^2 0%						
	Hulejova (2012) [Single arm int.][234]		ESR, BL / 3 months, mean (SD)<br>14.68 (11.9) / 13.2 (9.94)					

Alloc. Conc. = allocation concealment, AMSTAR2 = Assessing the Methodological Quality of Systematic Reviews, axSpA = axial spondyloarthritis, BL = baseline, Blind. Asses. = Blinded assessors, Blind. Part. = blinded participants, CI = confidence interval, CRP = C-reactive protein, ESR = erythrocyte sedimentation rate, H/UC = high / unclear risk of bias, L = low risk of bias, Rand. Seq. = random sequence generation, RCT = randomised controlled trial, SD = standard deviation, SMD = standardised mean difference

Table – Muscle strengthening exercises (axSpA), results and quality assessment

Outcome (outcome measure)	Study (date) [study type]	Standardised result, SMD (95% CI) unless otherwise stated	Natural result	AMSTAR2 quality	Rand. Seq.	Alloc. Conc.	Blind. Part.	Blind. Asses.
Spinal flexion	Rosu (2015) [RCT][227]	McKenzie program vs classic kinetic exercises at 24 weeks<br>SMD 1.44 (0.82, 2.07)	Schober, BL / 24 weeks, mean (SD)<br>McKenzie: 2.87 (0.77) / 4.63 (0.58)<br>Classic Kinetic: 2.96 (0.82) / 3.69 (0.72)		H/UC	H/UC	H/UC	H/UC
	Gyurcsik (2012) [Single arm int.][233]		Schober, BL / 3 months, mean (SD)<br>2.36 (1.85) / 2.84 (2.17)					
	Ortancil (2009) [Single arm int.][235]		Schober, BL / 6 weeks, mean (SD)<br>3.9 (1.6) / 4.0 (1.5)					
Grip strength	Hsieh (2014) [RCT][218]	Strength + range of motion vs range of motion only at 3 months<br>SMD -0.06 (-0.96, 0.84)	Grip strength, BL / 3 months, mean (SD)<br>Strength + range of motion: 28.6 (11.0) / 30.5 (12.0)<br>Range of motion only: 29.5 (10.7) / 31.1 (9.2)		L	L	H/UC	L
Walk-test	de Souza (2017) [RCT][225]	Exercise vs control at 16 weeks<br>SMD 0.76 (0.24, 1.29)	6 minute walk, BL / 16 weeks, mean (SD)<br>Exercise: 447.43 (54.99) / 464.43 (48.03)<br>Control: 435.43 (59.44) / 427.20 (49.6)		L	L	H/UC	L
	Durmus (2009) [NRT][223]	Muscle strengthening exercise vs conventional exercises<br>SMD -0.06 (-0.69, 0.58)<br>Muscle strengthening exercise vs control<br>SMD 0.88 (0.14, 1.62)	6 minute walk distance, BL / 12 weeks<br>Muscle strengthening exercise: 555.8 (91.0) / 620.4 (87.6)<br>Conventional exercise: 548.8 (82.4) / 625.0 (74.9)<br>Control: 537.4 (90.4) / 539.2 (99.9)					
	Ortancil (2009) [Single arm int.][235]		6 minute walk test, BL / 6 weeks, mean (SD)<br>574.2 (94.5) / 589.2 (87.1)					

Alloc. Conc. = allocation concealment, AMSTAR2 = Assessing the Methodological Quality of Systematic Reviews, axSpA = axial spondyloarthritis, BL = baseline, Blind. Asses. = Blinded assessors, Blind. Part. = blinded participants, CI = confidence interval, H/UC = high / unclear risk of bias, L = low risk of bias, Rand. Seq. = random sequence generation, RCT = randomised controlled trial, SD = standard deviation, SMD = standardised mean difference

Table – Muscle strengthening exercises (axSpA), SF36 results at final follow-up, mean (SD)

Author (date)	PCS	MCS	GH	PF	RP	RE	SF	BP	V	MH
de Souza (2017) [Exercise][225]			51.8 (21.5)	73 (18.3)	67.5 (42.1)	72.2 (43.9)	81.9 (21.7)	65.6 (19.4)	72.2 (17.8)	78.4 (18.4)
de Souza (2017) [Control][225]			47.3 (26.4)	68.2 (26)	71.7 (42.9)	84.4 (35.8)	78.7 (26.5)	65.6 (27.5)	3.7 (30.9) [sic]	76.7 (26.6)
Kasapoglu Aksoy (2017) [Exercise][226]			58.8 (19.4)	80.2 (16.5)	71.3 (35.6)	73.3 (41.3)	86.3 (18.9)	68.5 (20.4)	56.3 (17.7)	65.6 (18.7)
Kasapoglu Aksoy (2017) [Control][226]			51.4 (24.6)	73.3 (23.4)	66.7 (44.9)	69.8 (40.6)	77.9 (22.7)	57.1 (26.8)	47.1 (20.4)	55.4 (22.4)
Yigit (2013) [Exercise][221]			71.45 (15.93)	73.50 (18.93)	85.00 (32.85)	84.99 (27.53)	90.00 (22.43)	73.35 (17.21)	70.00 (16.86)	83.00 (12.03)
Yigit (2013) [Control][221]			52.80 (23.36)	59.50 (25.95)	61.15 (39.30)	68.81 (30.41)	65.58 (31.71)	54.30 (27.49)	54.25 (21.11)	68.2 (19.05)
Durmus (2009) [Exercise][222]			0.72 (0.13)	0.86 (0.14)	0.77 (0.26)	0.80 (0.21)	0.81 (0.20)	0.78 (0.12)	0.80 (0.08)	0.80 (0.10)
Durmus (2009) [Control][222]			0.66 (0.19)	0.68 (0.20)	0.67 (0.22)	0.68 (0.62)	0.72 (0.19)	0.69 (0.18)	0.68 (0.21)	0.68 (0.18)

axSpA = axial spondyloarthritis, BP = bodily pain, GH = general health, MCS = mental component score, MH = mental health, PCS = physical component score, PF = physical function, RE = role emotional, RP = role physical, SD = standard deviation, SF = social functioning, V = vitality

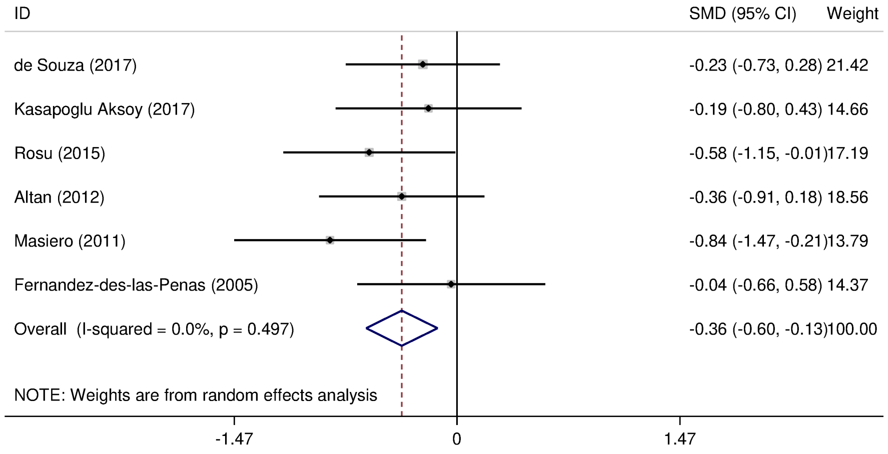

Figure – The effect of muscle strengthening exercise on function (axSpA)

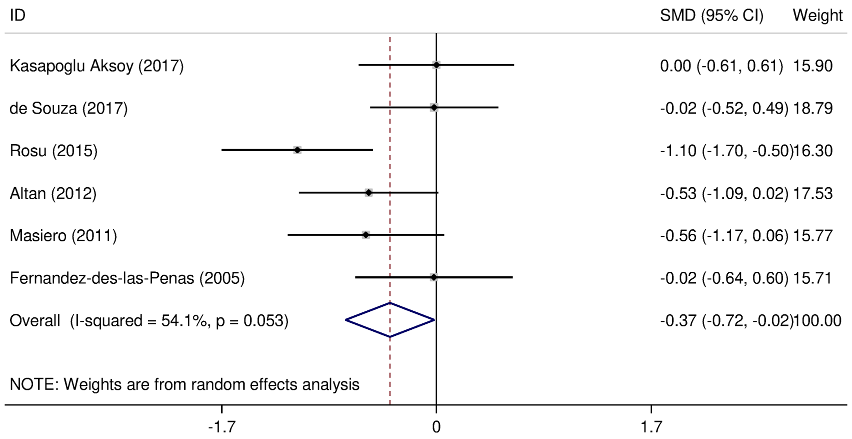

Figure – The effect of muscle strengthening exercise on disease activity

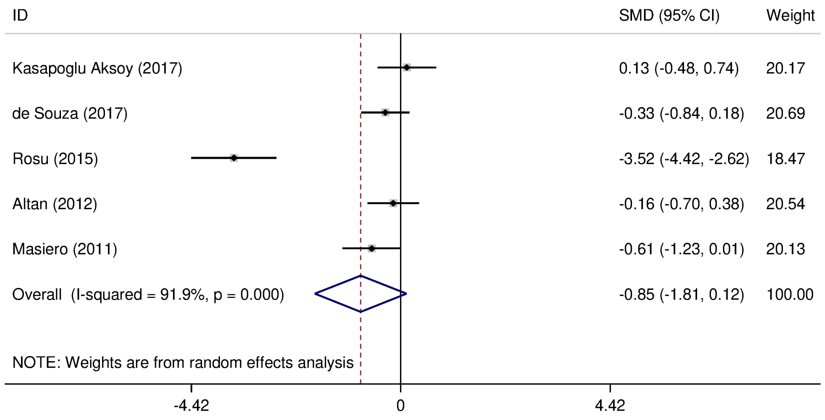

Figure – The effect of muscle strengthening exercise on spinal mobility

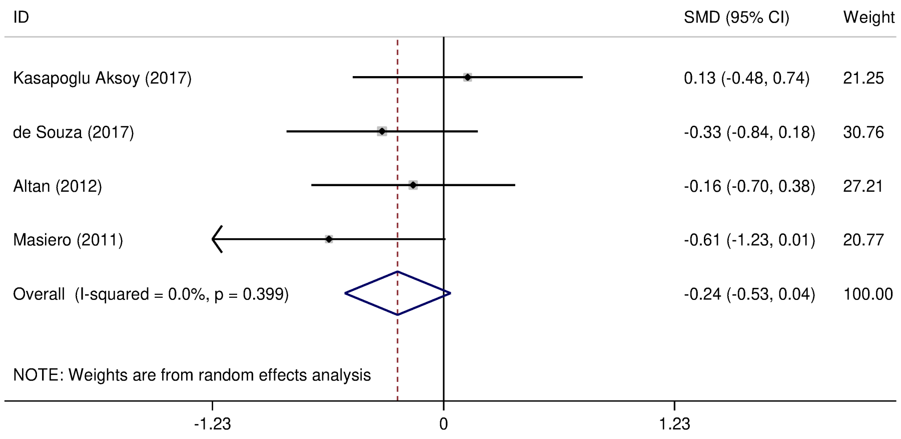

Figure – The effect of muscle strengthening exercise on spinal mobility – excluding one outlier (axSpA)

Supplementary table 75 – Description of original studies of muscle strengthening exercise in PsA

Table – Muscle strengthening exercise (PsA), description of included studies

Author (date) [country]	Study design	Inclusion criteria	Exposure detail	N	Age years, mean (SD)	N (%) female	Funders
Roger-Silva (2017) [Brazil][236]	RCT	CASPAR criteria, aged 18-65 years, use of DMARDs or TNFi with stable dose ≥3 months, stable doses of NSAIDs and steroids for ≥4 weeks<br>Exclusions: non-controlled cardiovascular disease, uncontrolled diabetes, severe psychiatric disease, fibromyalgia, history of regular exercise (≥30 mins 2x per week) in last 6 months, arthroplasty or hip/knee over last 12 months, other medical condition prohibiting exercise	1) Muscle strengthening exercises of upper and lower limbs and trunk<br>p) Waitlist control	1) 20<br>p) 21	1) 54.2 (8.2)<br>p) 50.8 (11.2)	1) 10 (50.0)<br>p) 11 (54.5)	**Not reported** – authors declared no conflicts of interest
Chimenti (2014) [Italy][237]	Single arm int.	Moderate disease activity, stable medication for 3 months, all receiving biologic and DMARD combination	Exercise program – stationary muscle contraction	23	50.8 (9.5)	12 (52.2)	**Industry** (Pfizer Italia)

CASPAR = Classification Criteria for Psoriatic Arthritis, DMARD = disease modifying anti-rheumatic drug, N = number, PsA = psoriatic arthritis, RCT = randomised controlled trial, SD = standard deviation, TNFi = tumour necrosis factor inhibitor

Supplementary table 76 – Results from reviews and interventional studies of muscle strengthening exercise in PsA

Table – Muscle strengthening exercise (PsA), results and quality assessment

Outcome (outcome measure)	Study (date) [study type]	Standardised result, SMD (95% CI) unless otherwise stated	Natural result	AMSTAR2 quality	Rand. Seq.	Alloc. Conc.	Blind. Part.	Blind. Asses.
Pain	Chimenti (2014) [Single arm int.][237]		Pain VAS, BL / 4 weeks / 12 weeks, mean SD<br>43.7 (23.1) / 34 (27.4) / 48.6 (24.8)					
Function	Roger-Silva (2017) [RCT][236]	Exercise vs control at 12 weeks<br>BASFI: SMD -0.50 (-1.12, 0.12)<br>HAQ-AS: SMD -0.65 (-1.28, -0.02)	BASFI, BL / 12 weeks, mean (SD)<br>Exercise: 4.2 (2.0) / 2.9 (2.2)<br>Control: 3.9 (2.4) / 4.0 (2.2)<br>HAQ-AS, BL / 12 weeks, mean (SD)<br>Exercise: 0.72 (0.45) / 0.45 (0.43)<br>Control: 0.69 (0.45) / 0.77 (0.55)		L	L	H/UC	L
	Chimenti (2014) [Single arm int.][237]		SPA-HAQ, BL / 4 weeks / 12 weeks, mean (SD)<br>0.58 (0.4) / 0.5 (0.42) / 0.56 (0.51)					
Disease activity	Roger-Silva (2017) [RCT][236]	Exercise vs control at 12 weeks<br>BASDAI: -0.66 (-1.29, -0.03)<br>DAS28: -0.42 (-1.04, 0.20)	BASDAI, BL / 12 weeks, mean (SD)<br>Exercise: 5.3 (2.4) / 3.3 (2.1)<br>Control: 4.5 (2.1) / 4.8 (2.4)<br>DAS28, BL / 12 weeks, mean (SD)<br>Exercise: 4.1 (1.3) / 3.1 (1.3)<br>Control: 3.9 (1.1) / 3.6 (1.1)		L	L	H/UC	L
Patient global	Chimenti (2014) [Single arm int.][237]		Global VAS, BL / 4 weeks / 12 weeks, mean (SD)<br>46.9 (18.7) / 38.25 (23.24) / 42.9 (27.01)					

Alloc. Conc. = allocation concealment, AMSTAR2 = Assessing the Methodological Quality of Systematic Reviews, BASDAI = Bath Ankylosing Spondylitis Disease Activity Index, BL = baseline, Blind. Asses. = Blinded assessors, Blind. Part. = blinded participants, CI = confidence interval, H/UC = high / unclear risk of bias, HAQ-AS = Health Assessment Questionnaire – Ankylosing spondylitis, L = low risk of bias, PsA = psoriatic arthritis, Rand. Seq. = random sequence generation, RCT = randomised controlled trial, SD = standard deviation, SMD = standardised mean difference, SPA-HAQ = Health Assessment Questionnaire for spondyloarthopathies, VAS = visual analogue scale

Table – Muscle strengthening exercises (PsA), SF36 results at final follow-up, median (IQR)

Author (date)	PCS	MCS	GH	PF	RP	RE	SF	BP	V	MH
Roger-Silva (2017) [Exercise][236]			63.6 (13.1)	77.2 (22.4)	71.3 (45.4)	83.3 (35.0)	79.5 (25.3)	72.5 (19.2)	70.9 (13.4)	71.1 (14.3)
Roger-Silva (2017) [Control][236]			53.0 (14.1)	71.2 (18.4)	58.8 (44.9)	81.0 (34.3)	72.0 (30.9)	53.4 (22.3)	61.4 (19.1)	66.8 (21.7)
Chimenti (2014) [Exercise][237]			6.21 (1.97)	5.77 (1.64)	24 (5.18)	4.53 (1.19)	5.54 (0.87)	6.36 (2.34)	36.3 (3.9)	12.7 (3.8)

BP = bodily pain, FU = follow-up, GH = general health, IQR = interquartile range, MCS = mental component score, MH = mental health, PCS = physical component score, PF = physical function, PsA = psoriatic arthritis, RE = role emotional, RP = role physical, SD = standard deviation, SF = social functioning, V = vitality

Supplementary table 77 – Description of original studies of aerobic exercise in SSc

Table – Aerobic exercise (SSc), description of included studies

Author (date) [country]	Study design	Inclusion criteria	Exposure detail	N	Age years, mean (SD)	N (%) female	Funders
Antonioli (2009) [Italy][238]	Single arm int.	Leroy criteria, aged 18-75 years, stable disease no change in medication for 3 months<br>Exclusions: inability to perform rehabilitation program due to skeletal-muscle impairment or other illness, presence of other diease interfering with performance of daily activities, pulmonary hypertension, psychiatric disorders, alcohol / drug abuse, pregnancy or planned pregnancy in next 6 months	1) 10x 30 min session – warm up, training of mother functions and respiratory exercises – lower extremity exercises included treadmill and free walking. Also hand stretching	1) 16	Median (IQR)<br>1) 66.5 (63.0, 70.5)	1) 16 (100)	**Not reported** – authors declared no conflict of interest

int. = intervention, N = number, SD = standard deviation, SSc = systemic sclerosis

Supplementary table 78 – Results from reviews and interventional studies of aerobic exercise in SSc

Table – Aerobic (SSc), results and quality assessment

Outcome (outcome measure)	Study (date) [study type]	Standardised result, SMD (95% CI) unless otherwise stated	Natural result	AMSTAR2 quality	Rand. Seq.	Alloc. Conc.	Blind. Part.	Blind. Asses.
Function	Antonioli (2009) [single arm int.][238]		HAQ, BL / 4 months, median (IQR)<br>0.63 (0.34, 0.75) / 0.44 (0.25, 0.75)					
Respiratory function	Antonioli (2009) [single arm int.][238]		SGRQ, BL / 4 months, median (IQR)<br>30.9 (17.3, 36.9) / 22.7 (12.5, 31.3)					

Alloc. Conc. = allocation concealment, AMSTAR2 = Assessing the Methodological Quality of Systematic Reviews, BL = baseline, Blind. Asses. = Blinded assessors, Blind. Part. = blinded participants, CI = confidence interval, H/UC = high / unclear risk of bias, HAQ = Health Assessment Questionnaire, IQR = interquartile range, Rand. Seq. = random sequence generation, SGRQ = Saint George's Respiratory Questionnaire, SSc = systemic sclerosis

Table – Aerobic exercises (SSc), SF36 results at final follow-up, median (IQR)

Author (date)	PCS	MCS	GH	PF	RP	RE	SF	BP	V	MH
Antonioli (2009) [exercise][238]	44.0<br>(41.5, 48)	50.4<br>(46, 54.3)	42.5<br>(33.8, 75)	75.0<br>(58.8, 81.3)	50.0<br>(25,75)	100<br>(0, 100)	87.5<br>(75, 100)	66.5<br>(41,74)	50.0<br>(43.8, 71.3)	66.0<br>(53.5, 78)

BP = bodily pain, FU = follow-up, GH = general health, IQR = interquartile range, MCS = mental component score, MH = mental health, PCS = physical component score, PF = physical function, RE = role emotional, RP = role physical, SD = standard deviation, SF = social functioning, SSC = systemic sclerosis, V = vitality

Supplementary table 79 – Description of reviews of studies of aerobic + muscle strengthening exercise in SSc

Table – Aerobic + muscle strengthening exercise (SSc), description of reviews

Authors (date)	Review type	Study type included	Exposure detail	Number of studies included	Funders
Moran (2014)[239]	SR	Single arm interventions	Exercise	1	**No funding**

RCT = randomised controlled trial, SR = systematic review, SSc = systemic sclerosis

Supplementary table 80 – Description of original studies of aerobic + muscle strengthening exercise in SSc

Table – Aerobic + muscle strengthening exercise (SSc), description of included studies

Author (date) [country]	Study design	Inclusion criteria	Exposure detail	N	Age years, mean (SD)	N (%) female	Funders
Rannou (2017) [France][240]	RCT	ACR SSc criteria or Leroy and Medsger criteria, aged ≥18 years, HAQ ≥0.5<br>Exclusions: disabling comorbidities, cognitive impairment, participation in clinical trial in last 3 months, inclusion in a standardized physical therapy program in previous 6 months	1) Individualised physical therapy program – objective to increase range of motion of impaired joints, increase muscle strength and aerobic capacity and decrease mouth microstomia, skin retractions and limitations in activities<br>p) Usual care	1) 110<br>p) 108	1) 52.7 (14.8)<br>p) 53.1 (14.4)	1) 95 (86.4)<br>p) 86 (79.6)	**Government** (Programme Hospitalier de Recherche Clinique)
Schouffoer (2011) [The Netherlands][241]	RCT	Leroy criteria, aged 18-75 years, able to cycle of exercise bike, stable anti-inflammatory medication over past 2 months, fluent in Dutch<br>Exclusions: engagement in another exercise program, concomitant disease interfering with performance of daily activities	1) Multidisciplinary care with standardised group sessions (general exercise, hand/mouth exercises, education) + individual treatment by rheumatologist and health professional. Patients also participated in weekly group exercise sessions near their home and performed exercise at home<br>p) Waitlist control	1) 28<br>p) 25	1) 53.9 (10.8)<br>p) 51.7 (10.8)	1) 19 (67.9)<br>p) 21 (84.0)	**Not reported**
Pinto (2011) [Brazil][242]	Single arm int.	ARA SSc criteria, women, physical inactive ≥6 months<br>Exclusions: moderate or severe pulmonary involvement, echocardiographic evidence of cardiac impairment, pulmonary artery systolic pressure ≥40 mm Hg, history of myositis, history of tobacco use, renal insufficiency, hypertension, anaemia, pathologic lung impairment	12 week combined muscle strengthening and aerobic training program	11	44 (13)	11 (100)	**Not reported** – authors declared no conflict of interest

ACR = American College of Rheumatology, ARA = American Rheumatism Association, HAQ = Health Assessment Questionnaire, int. = intervention, N = number, RCT = randomised controlled trial, SD = standard deviation, SSc = systemic sclerosis

Supplementary table 81 – Results from reviews and interventional studies of aerobic + muscle strengthening exercise in SSc

Table – Aerobic + muscle strengthening (SSc), results and quality assessment

Outcome (outcome measure)	Study (date) [study type]	Standardised result, SMD (95% CI) unless otherwise stated	Natural result	AMSTAR2 quality	Rand. Seq.	Alloc. Conc.	Blind. Part.	Blind. Asses.
Pain	Rannou (2017) [RCT][240]	Exercise vs control at 1 month<br>SMD -0.00 (-0.27, 0.27)	Pain VAS, BL / 1 month / 12 months, mean (SD)<br>Exercise: 37.57 (27.73) / 24.47 (22.88) / 33.80 (29.83)<br>Control: 41.04 (30.85) / 41.57 (28.40) / 33.81 (31.42)					
	Schouffoer (2011) [RCT][241]	Exercise vs control, change BL-24 weeks<br>SMD -0.12 (-0.66, 0.42)	Pain VAS, BL / change BL-24 weeks, mean (SD †)<br>Exercise: 27.0 (27.7) / -2.1 (24.7)<br>Control: 30.6 (25.5) / 1.2 (32.4)		L	H/UC	H/UC	L
Function	Moran (2014) [SR][239]		One study reported some improvement	Low				
	Rannou (2017) [RCT][240]	Exercise vs control at 1 month<br>HAQ: SMD -0.22 (-0.48, 0.05)<br>HAQ-S: SMD -0.30 (-0.57, 0.04)	HAQ, BL / 1 month / 12 months, mean (SD)<br>Exercise: 1.36 (0.64) / 1.13 (0.61) / 1.19 (0.74)<br>Control: 1.34 (0.67) / 1.27 (0.69) / 1.20 (0.74)<br>HAQ-S, BL / 1 month / 12 months, mean (SD)<br>Exercise: 1.18 (0.55) / 0.98 (0.51) / 1.09 (0.65)<br>Control: 1.23 (0.60) / 1.15 (0.61) / 1.08 (0.64)		L	L	H/UC	H/UC
	Schouffoer (2011) [RCT][241]	Exercise vs control, change BL-24 weeks<br>SMD -0.29 (-0.84, 0.25)	HAQ-S, BL / change BL-24 weeks, mean (SD †)<br>Exercise: 0.81 (0.66) / -0.26 (0.69)<br>Control: 0.73 (0.46) / -0.1 (0.31)		L	H/UC	H/UC	L
	Bespoke meta-analysis including[240;241]	Exercise vs control<br>SMD -0.30 (-0.54, -0.06), I^2 0%						
Hand function	Rannou (2017) [RCT][240]	Exercise vs control at 1 month<br>SMD -0.39 (-0.66, -0.12)	Cochin hand function, BL / 1 month / 12 months, mean (SD)<br>Exercise: 20.05 (15.59) / 14.82 (13.47) / 18.64 (16.78)<br>Control: 22.18 (18.19) / 21.20 (18.95) / 20.26 (18.69)		L	L	H/UC	H/UC
	Schouffoer (2011) [RCT][241]	Exercise vs control, change BL-24 weeks<br>SMD -0.54 (-1.09, 0.01)	HAMIS, BL / change BL-24 weeks, mean (SD †)<br>Exercise: 6.8 (5.4) / -1.6 (4.2)<br>Control: 5.7 (3.9) / 0.2 (2.0)		L	H/UC	H/UC	L
	Bespoke meta-analysis including[240;241]	Exercise vs control<br>SMD -0.42 (-0.66, -0.18), I^2 0%						
Raynaud's phenomenon	Moran (2014) [SR][239]		One study reported no improvement	Low				

† SD calculated from 95% CI in paper; Alloc. Conc. = allocation concealment, AMSTAR2 = Assessing the Methodological Quality of Systematic Reviews, BL = baseline, Blind. Asses. = Blinded assessors, Blind. Part. = blinded participants, CI = confidence interval, H/UC = high / unclear risk of bias, HAMIS = Hand mobility in scleroderma, HAQ-S = Health Assessment Questionnaire – scleroderma, L = low risk of bias, Rand. Seq. = random sequence generation, RCT = randomised controlled trial, SD = standard deviation, SMD = standardised mean difference, SSc = systemic sclerosis

Table – Aerobic + muscle strengthening (SSc), results and quality assessment

Outcome (outcome measure)	Study (date) [study type]	Standardised result, SMD (95% CI) unless otherwise stated	Natural result	AMSTAR2 quality	Rand. Seq.	Alloc. Conc.	Blind. Part.	Blind. Asses.
Skin score	Rannou (2017) [RCT][240]	Exercise vs control at 12 months<br>SMD -0.31 (-0.58, -0.04)	Rodnan skin score, BL / 12 months, mean (SD)<br>Exercise: 14.21 (8.77) / 10.98 (7.78)<br>Control: 16.67 (10.55) / 13.49 (8.38)		L	L	H/UC	H/UC
Digital ulcers	Moran (2014) [SR][239]		One study reported no improvement	Low				
Grip strength	Schouffoer (2011) [RCT][241]	Exercise vs control, change BL-24 weeks<br>SMD 0.57 (0.02, 1.12)	Grip strength, BL / change BL-24 weeks, mean (SD †)<br>Exercise: 26.2 (12.4) / 2.6 (4.5)<br>Control: 26.7 (10.3) / -0.4 (6.0)		L	H/UC	H/UC	L
	Pinto (2011) [single arm int.][242]		Grip strength, BL / 12 week, mean (SD)<br>20 (9) / 22 (11)					
Walk-test	Schouffoer (2011) [RCT][241]	Exercise vs control, change BL-24 weeks<br>SMD 0.33 (-0.21, 0.88)	6 min walk test, BL / change BL-24 weeks, mean (SD †)<br>Exercise: 499.9 (107.2) / 34.8 (72.8)<br>Control: 520.6 (94.2) / 12.0 (64.0)		L	H/UC	H/UC	L

† SD calculated from 95% CI in paper; Alloc. Conc. = allocation concealment, AMSTAR2 = Assessing the Methodological Quality of Systematic Reviews, BL = baseline, Blind. Asses. = Blinded assessors, Blind. Part. = blinded participants, CI = confidence interval, H/UC = high / unclear risk of bias, L = low risk of bias, Rand. Seq. = random sequence generation, RCT = randomised controlled trial, SD = standard deviation, SMD = standardised mean difference, SSc = systemic sclerosis

Table – Aerobic + muscle strengthening exercises (SSc), SF36 results at final follow-up, mean (SD)

Author (date)	PCS	MCS	GH	PF	RP	RE	SF	BP	V	MH
Rannou (2017) [Exercise][240]	36.33 (8.08)	44.87 (10.81)								
Rannou (2017) [Control][240]	35.98 (9.37)	41.74 (11.54)								
Schouffoer (2011) [Exercise][241]	-0.7 (8.2) §	1.9 (8.4) §								
Schouffoer (2011) [Control][241]	1.4 (9.6) §	1.6 (9.9) §								

§ Change from BL to 24 weeks

BP = bodily pain, GH = general health, IQR = interquartile range, MCS = mental component score, MH = mental health, PCS = physical component score, PF = physical function, RE = role emotional, RP = role physical, SD = standard deviation, SF = social functioning, V = vitality, SSc = systemic sclerosis

Supplementary table 82 – Description of original studies of aquatic exercise in SSc

Table – Aquatic exercise (SSc), description of included studies

Author (date) [country]	Study design	Inclusion criteria	Exposure detail	N	Age years, mean (SD)	N (%) female	Funders
Maddali Bongi (2009) [Italy][243]	Single arm int. §	SSc	1 hour session in pool – 10 mins warm up, 20 mins stretching and pulmonary rehabilitee, 20 min treatment of local and global pain by individualised exercises	10	58.0 (15.1)	6 (60.0)	**Professional body** (Italian Association for the study of Systemic Sclerosis and Fibrosis Diseases)

§ Was an RCT, but the authors did not report any outcome data for the controls
N = number, SD = standard deviation, SSc = systemic sclerosis

Supplementary table 83 – Results from reviews and interventional studies of aquatic exercise in SSc

Table – Aquatic exercise (SSc), results and quality assessment

Outcome (outcome measure)	Study (date) [study type]	Standardised result, SMD (95% CI) unless otherwise stated	Natural result	AMSTAR2 quality	Rand. Seq.	Alloc. Conc.	Blind. Part.	Blind. Asses.
Function	Maddali Bongi (2009) [Single arm int.][243]		HAQ, BL / 9 weeks / 18 weeks, mean (SD)<br>1.2 (1.2) / 0.9 (1.1) / 0.8 (1.2)					
Hand function	Maddali Bongi (2009) [Single arm int.][243]		HAMIS, BL / 9 weeks / 18 weeks, mean (SD)<br>10.2 (4.8) / 6.0 (3.7) / 6.4 (7.4)					

Alloc. Conc. = allocation concealment, AMSTAR2 = Assessing the Methodological Quality of Systematic Reviews, BL = baseline, Blind. Asses. = Blinded assessors, Blind. Part. = blinded participants, CI = confidence interval, H/UC = high / unclear risk of bias, HAMIS = Hand mobility in scleroderma, HAQ = Health Assessment Questionnaire, L = low risk of bias, Rand. Seq. = random sequence generation, RCT = randomised controlled trial, SD = standard deviation, SMD = standardised mean difference, SSc = systemic sclerosis

Table – Aquatic exercises (SSc), SF36 results at final follow-up, mean (SD)

Author (date)	PCS	MCS	GH	PF	RP	RE	SF	BP	V	MH
Maddali Bongi (2009) [Exercise][243]	44.9 (8.6)	44.6 (6.0								

BP = bodily pain, FU = follow-up, GH = general health, MCS = mental component score, MH = mental health, PCS = physical component score, PF = physical function, RE = role emotional, RP = role physical, SD = standard deviation, SF = social functioning, SSc = systemic sclerosis, V = vitality

Supplementary table 84 – Description of original studies of muscle strengthening exercise in SSc

Table – Muscle strengthening exercise (SSc), description of included studies

Author (date) [country]	Study design	Inclusion criteria	Exposure detail	N	Age years, mean (SD)	N (%) female	Funders
Stefanantoni (2016) [Italy][244]	RCT	ACR SSc criteria, communicated in Italian, hand involvement (skin thickening with or without joint synovitis, joint contractures, tendon friction rubs, digital ulcers), stable disease defined as the absence of synovitis and digital ulcers	1) Advice of hand exercises to maintain tissue elasticity<br>p) General guidelines for exercise	1) 15<br>p) 16	1) 61.4<br>p) 60.5	1) 15 (100)<br>p) 15 (93.8)	**Not reported –** Authors declare no conflict of interest
Horvath (2017) [Hungary][245]	NRT	Aged 18-75 years, joint contractures of the hands, ability to participate in physical therapy in another city 150km away from University<br>Exclusion: CRP >10, severe interstitial lung disease, hypertension, cardiac disease, active skin ulcers, urine incontinence, attending other physiotherapy	1) 30 mins isometric, isotonic and hand stretching exercises, ergotherapy, thermal baths, mud baths of the hands, whirlpool therapy<br>p) Had everything in intervention, but not hand therapy	1) 31<br>p) 22	1) 59.7 (14.5)<br>p) 62.1 (8.4)	1) 29 (93.5)<br>p) 20 (90.9)	**Government** (Hungarian Scientific Research Funds, European Union and the State of Hungary)
Mugii (2018) [Japan][246]	Single arm int.	ACR criteria	Home hand stretching program	43	Median (range)<br>51 (7 [sic]-73)	35 (81.4)	**Government** (Diseases from the Ministry of Health, Labor and Welfare of Japan)
Landim (2017) [Brazil][247]	Single arm int.	Aged ≥18 years, ACR/EULAR criteria, hand involvement, stable treatment for 3 months<br>Exclusions: Enrolled in any other rehabilitation program in previous 3 months, hand disability due to other pathology, could not perform exercises	home based, finger flexes and extensions, wrist flex and extension, forearm flexing and stretching, finger pinches	22	48.1 (11.7)	18 (85.7)	**Not reported**
Mugii (2006) [Japan][248]	Single arm int.	ACR criteria	Home hand stretching program	45	48.6 (17.3)	39 (86.7)	**Government** (Japanese Ministry of Health and Welfare)

ACR = American College of Rheumatology, CRP = C-reactive protein, EULAR = European League Against Rheumatism, N = number, NRT = non-randomised trial, RCT = randomised controlled trial, SD = standard deviation, SSc = systemic sclerosis

Supplementary table 85 – Results from reviews and interventional studies of muscle strengthening exercise in SSc

Table – Muscle strengthening exercise (SSc), results and quality assessment

Outcome (outcome measure)	Study (date) [study type]	Standardised result, SMD (95% CI) unless otherwise stated	Natural result	AMSTAR2 quality	Rand. Seq.	Alloc. Conc.	Blind. Part.	Blind. Asses.
Pain	Horvath (2017) [NRT][245]		Pain VAS, change BL-6 months, mean (SD †)<br>Exercise: -8.3 (21.4)<br>Control: 0.44 (32.1)					
	Landim (2017) [Single arm int.][247]		Pain VAS, BL / 8 weeks, mean (SD)<br>3.97 (2.92) / 2.21 (2.07)					
Function	Stefanantoni (2016) [RCT][244]	Exercise guidance vs control<br>SMD -0.55 (-1.27, 0.17)	HAQ, BL / 3 months, mean (SD §)<br>Exercise guidance: 1.28 (0.99) / 0.77 (0.74)<br>Control: 1.55 (0.81) / 1.20 (0.81)		H/UC	H/UC	H/UC	L
	Horvath (2017) [NRT][245]		HAQ, change BL-6 months, mean (SD †)<br>Exercise: -0.21 (0.47)<br>Control: 0.007 (0.89)					
	Mugii (2018) [Single arm int.][246]		HAQ, BL / 9 years, mean (SD)<br>"improved" ROM: 0.78 (0.47) / 0.67 (0.63)<br>"worsened" ROM: 0.50 (0.60) / 0.97 (0.62)					
	Landim (2017) [Single arm int.][247]		HAQ, BL / 8 weeks, mean (SD)<br>1.08 (0.88) / 0.67 (0.62)<br>HAQ-S, BL / 8 weeks, mean (SD)<br>0.95 (0.53) / 0.48 (0.39)					
	Mugii (2006) [Single arm int.][248]		HAQ, BL / 1 year, mean (SD)<br>0.48 (0.45) / 0.38 (0.47)					
Hand function	Stefanantoni (2016) [RCT][244]		Duroz, BL / 3 months, median (IQR)<br>Exercise guidance: 23 (10, 36.5) / 15 (10, 28)<br>Control: 27.5 (15.7, 49.2) / 21.5 (22.7, 45) [sic]		H/UC	H/UC	H/UC	L
	Horvath (2017) [NRT][245]		Cochin, change BL-6 months, mean (SD †)<br>Exercise: -2.0 (8.0)<br>Control: -0.5 (5.9)					
	Landim (2017) [Single arm int.][247]		Cochin, BL / 8 weeks, mean (SD)<br>19.24 (15.78) / 12.48 (12.04)					
Grip strength	Landim (2017) [Single arm int.][247]		Grip strength, BL / 8 weeks, mean (SD)<br>14.43 (6.87) / 19 (7.09)					

† SD calculated from 95% CI in paper
§ mean (SD) estimated from median (interquartile range) using publish formula)[87]
Alloc. Conc. = allocation concealment, AMSTAR2 = Assessing the Methodological Quality of Systematic Reviews, BL = baseline, Blind. Asses. = Blinded assessors, Blind. Part. = blinded participants, CI = confidence interval, H/UC = high / unclear risk of bias, IQR = interquartile range, L = low risk of bias, NRT = non-randomised trial, Rand. Seq. = random sequence generation, RCT = randomised controlled trial, SD = standard deviation, SMD = standardised mean difference, SSc = systemic sclerosis

Table – Muscle strengthening exercises (SSc), SF36 results at final follow-up, mean (SD)

Author (date)	PCS	MCS	GH	PF	RP	RE	SF	BP	V	MH
Stefanantoni (2016) [Exercise][244]	36.6 (10.63)	39.6 (7.9)								
Stefanantoni (2016) [Control][244]	40.6 (13.1)	40.6 (13.1)								
Landim (2017) [Exercise][247]			58.29 (16.15)	61.90 (45.84)	60.14 (20.06)	53.96 (47.70)	75.60 (17.44)	63.38 (19.37)	62 (20.86)	72.38 (19.75)

BP = bodily pain, FU = follow-up, GH = general health, MCS = mental component score, MH = mental health, PCS = physical component score, PF = physical function, RE = role emotional, RP = role physical, SD = standard deviation, SF = social functioning, SSc = systemic sclerosis, V = vitality

Supplementary table 86 – Description of original studies of aerobic exercise in gout

Table – Aerobic exercise (gout), description of included studies

Author (date) [country]	Study design	Inclusion criteria	Exposure detail	N	Age years, mean (SD)	N (%) female	Funders
Ma (2018) [China][249]	Case control	Primary gout – 2015 classification	Exercise was defined as doing regular sport ≥150 mins per week	5693	51.1 (14.2)	327 (5.7)	**Government** (Ministry of Science and Technology of China, National Science Foundation of China, Science and Technology Development Project of Shandong Province)

N = number, SD = standard deviation

Supplementary table 87 – Results from observational studies of aerobic exercise in gout

Table – Aerobic exercise (AS), results and quality assessment of observational studies

Outcome (outcome measure)	Study (date) [study type]	Standardised result, SMD (95% CI) unless otherwise stated	Natural result	Study Pop.	Attr.	Prog. Meas.	Outc. Meas.	Conf.	Stats.
Tophus	Ma (2018) [case control][249]		Tophus, OR (95% CI) [adjusted]<br>OR 0.67 (0.54, 0.83) – exercise associated with reduced odds of tophus	L	L	M	L	L	M

Attr. = attrition, CI = confidence interval, Conf. = confounding, JSW = joint space width, L = low risk of bias, M = moderate risk of bias, OR = odds ratio, Outc. Meas = outcome measurement, Prog. Meas. = prognostic factor measurement, Pros = prospective, Rand. Seq. = random sequence generation, SMD = Standardised mean difference, Stats. = statistical analysis, Study Pop. = study population

Supplementary table 88 – Description of original studies of yoga in gout

Table – Yoga (gout), description of included studies

Author (date) [country]	Study design	Inclusion criteria	Exposure detail	N	Age years, mean (SD)	N (%) female	Funders
Adithya Acharya (2013) [India][250]	RCT	Aged 20-65, symptom duration <10 years, gout symptoms, no tophi, not complete joint destruction<br>Exclusions: heamorthrosis [sic], Kochs arthritis, septic arthritis, chronic renal failure, severe systemic multiorgan syndrome, basti ayogya, siravyadha ayogya	1) Guduchi Siddha Yoga Basti<br>p) blood letting	1) 20<br>p) 20	21-30/31-40/41-50/51-60/61-70:<br>1) 5 / 9 / 3 / 0 / 3<br>p) 2 / 6 / 7 / 5 / 0	1) 6 (30.0)<br>p) 9 (45.0)	**No funding**

N = number, RCT = randomised controlled trial, SD = standard deviation

Supplementary table 89 – Results from reviews and interventional studies of yoga in gout

Table – Yoga (gout), results and quality assessment

Outcome (outcome measure)	Study (date) [study type]	Standardised result, SMD (95% CI) unless otherwise stated	Natural result	AMSTAR2 quality	Rand. Seq.	Alloc. Conc.	Blind. Part.	Blind. Asses.
Pain	Adithya Acharya (2013) [RCT][250]		Pricking pain, 0-3, BL / 24, N(%)<br>1) 0=0(0), 1=0(0), 2=13 (65), 3=7(35) / 0=6(30), 1=13 (65), 2=1(5), 3=0(0)<br>p) 0=0(0), 1=0(0), 2=14(70), 3=6(30) / 0=2(10), 1=17(85), 2=1(5), 3=0(0)		H/UC	H/UC	H/UC	L
Swelling	Adithya Acharya (2013) [RCT][250]		Swelling, 0-2, BL /24 weeks, N(%)<br>1) 0=0(0), 1=14(70), 2=6(30) / 0=5(25), 1=14(70), 2=1(5)<br>p) 0=0(0), 1=7(35), 2=13(65) / 0=0(0), 1=20(100), 2=0(0)		H/UC	H/UC	H/UC	L
Tenderness	Adithya Acharya (2013) [RCT][250]		Tenderness, 0-3, BL /24 weeks, N(%)<br>1) 0=0(0), 1=6(30), 2=14(70), 3=0(0) / 0=12(60), 1=8(40), 2=0(0), 3=0(0)<br>p) 0=0(0), 1=13(65), 2=7(35), 3=0(0) / 0=15(75), 1=5(25), 2=0(0), 3=0(0)		H/UC	H/UC	H/UC	L
Uric acid	Adithya Acharya (2013) [RCT][250]		Serum uric acid, BL /24 weeks, mean(SD)<br>1) 8.43 (1.12) / 6.63 (1.32)<br>p) 8.55 (1.53) / 7.41 (1.58)		H/UC	H/UC	H/UC	L

Alloc. Conc. = allocation concealment, AMSTAR2 = Assessing the Methodological Quality of Systematic Reviews, BL = baseline, Blind. Asses. = Blinded assessors, Blind. Part. = blinded participants, CI = confidence interval, H/UC = high / unclear risk of bias, L = low risk of bias, Rand. Seq. = random sequence generation, RCT = randomised controlled trial, SD = standard deviation

Supplementary table 90 – Description of studies of assessing weight and outcomes in OA

Table – Osteoarthritis, description of reviews

Authors (date)	Review type	Study type included	Type of OA	Exposure detail	Number of studies included	Funders
Pozzobon (2018)[18]	MA	Observational studies	Hip, knee	Obesity before arthroplasty	31	**No funding**
Corbett (2013)[15]	MA	RCTs	Knee	Weight loss interventions	5	**Government** (NIHR)
de Rooij (2016)[38]	SR	Prospective cohorts	Hip	Studies investigating the association between BMI and outcomes	15	**Professional body** (Royal Dutch Society for Physical Therapy)
de Rooij (2016)[22]	SR	Observational studies	Knee	Studies investigating the association between BMI and outcomes	58	**Professional body** (Royal Dutch Society for Physical Therapy)
Bastick (2015)[23]	SR	Observational studies	Knee	Studies investigating the association between BMI and radiographic progression	79	**Charity** (Dutch Arthritis Foundation)
Le Quintrec (2014)[24]	SR	RCTs	Hip, knee	Weight loss interventions	8	**Not reported –** Authors declared no conflict of interest
Fernandes (2013)[36]	SR	MA, SR, RCTs, observational studies	Hip, knee	Weight loss interventions	23	**Professional body** (EULAR)

BMI = body mass index, EULAR = European League Against Rheumatism, MA = meta-analysis, NIHR = National Institute for Health Research, OA = osteoarthritis, RCT = randomised controlled trial, SR = systematic review

Table – Osteoarthritis, description of included studies

Author (date) [country]	OA site	Study design	Inclusion criteria	Exposure / intervention detail	N	Age, mean (SD) years	N (%) female	Funders
Sadeghi (2019) [Iran][251]	Knee	RCT	ACR knee OA, KL grade I-II, OA considered through mechanical knee pain, joint crepitation and radiographic signs<br>Exclusions: Disease other than OA, knee / hip prosthesis, consumption of glucosamine/chondroitin through last 6 months, overuse of sedative drugs, KL grade III-IV, rheumatic disease history	1) Suggested to eat less starch, rice, spaghetti, fatty goods, solid and liquid oil<br>p) No suggestion diet alterations	1) 31<br>p) 31	1) 48 (8.1)<br>p) 44.5 (8.9)	1) 28 (90.3)<br>p) 28 (90.3)	**University** (Zanjan University of Medical Science)
O'Brien (2018) [Australia][252]	Knee	RCT	Primary complaint of pain due to knee OA last >3 months, aged ≥18 years, BMI 27-40, average pain intensity 3/4 on 10 point scale over past week, moderate interference in daily living, access to telephone<br>Exclusions: Known or suspected serious pathology as underlying cause of knee pain, previous obesity surgery, participating in commercial weight loss program, knee surgery in last 6 months or planned, unable to adapt to program due to living arrangements, medical conditions precluding safe participation in exercise, unable to speak English	1) Telephone intervention – brief advice and education about benefits of weight loss / physical activity for OA, then referred to generic weight loss service which supported people to make lifestyle improvements (diet, physical activity)<br>p) Usual care	1) 59<br>p) 60	1) 63.0 (11.1)<br>p) 60.2 (13.9)	1) 39 (66.1)<br>p) 35 (58.3)	**Industry (**Hunter Medical Research), **University** (University of Newcastle)
Allen (2017) [USA][253]	Hip, knee	Cluster RCT	OA hip or knee based on radiographic evidence in medical record or met ACR criteria plus joint symptoms, BMI ≥25 and not meeting physical activity recommendations<br>Exclusions: Other rheumatologic conditions, recent hip/knee surgery, waitlist for arthroplasty, recent hospitalization for cardiovascular or cerebrovascular events, severe neurological or psychiatric events, severe memory loss, terminal illness, nursing home residence, severe hearing or speech impairment, blindness, participation another OA intervention, current pregnancy, no primary care visits at Dukes in past 18 months, other health conditions that would prohibit sage participation	Telephone based intervention – goal setting and action planning, patients receivd educational materials, exercise video and audio CD. Practices randomised to intervention or control and then patients randomised to intervention creating four groups:<br>1) Patient intervention<br>2) Practice intervention<br>3) Patients + practice intervention<br>p) Usual care	1) 128<br>2) 140<br>3) 140<br>p) 129	1) 63.9 (9.3)<br>2) 62.6 (9.6)<br>3) 62.7 (9.3)<br>p) 63.9 (10.2)	1) 72.7%<br>2) 75.7%<br>3) 75.7%<br>p) 71.3%	**Government** (NIH, Department of Veterans Affairs Health Services Research and Development Service)

ACR = American College of Rheumatology, BMI = body mass index, KL = Kellgren-Lawrence, N = number, NIH = National Institutes of Health, OA = osteoarthritis, RCT = randomised controlled trial, SD = standard deviation, USA = United States of America

Table – Osteoarthritis, description of included studies

Author (date) [country]	OA site	Study design	Inclusion criteria	Exposure / intervention detail	N	Age, mean (SD) years	N (%) female	Funders
Allen (2016) [USA][254]	Hip, knee	Cluster RCT	Hip (radiographic) or knee (radiographic or ACR criteria) OA, joint symptoms present for most days in past month or patients used medication for these symptoms on most days, BMI ≥25<br>Exclusions: Other rheumatologic conditions, recent hip/knee surgery, waitlist for arthroplasty, recent hospitalization for cardiovascular or cerebrovascular events, severe neurological or psychiatric events, severe memory loss, terminal illness, nursing home residence, severe hearing or speech impairment, blindness, participation another OA intervention, current pregnancy, no primary care visits at Dukes in past 18 months	1) Patient intervention – telephone calls to reduced weight loss, Provider intervention – training on when to refer patients<br>p) Usual care	1) 151<br>p) 149	1) 60.4 (9.4)<br>p) 61.7 (9.0)	1) 13.2%<br>p) 5.4%	**Government** (Department of Veterans Affairs, Health Services Research and Development Service)
Christensen (2015) [Denmark][255]	Knee	RCT	Aged ≥50 years, confirmed knee OA, pain and on standing radiographs in at least 1 joint compartment [sic]<br>Exclusions: lack of motivation to lose weight, inability to speak Danish, planned anti-obesity surgery, total knee alloplasty [sic], receiving pharmacological treatment for obesity	All received 16 week intensive dietary therapy, then randomised to weight maintanence program<br>1) Dietary program – formula products<br>2) Exercise 3x per week<br>p) usual care	1) 64<br>2) 64<br>p) 64	1) 63.0 (6.5)<br>2) 62.9 (5.8)<br>p) 61.7 (6.8)	1) 52 (81.3)<br>2) 52 (81.3)<br>p) 51 (79.7)	**Charity** (Oak, Velux, Augustinus, A. P. Moller, Horslev, Bjarne Jensen and Ases and Ejnar Danielsens's, Foundations), **Industry** (Cambridge Weight Plan), **Professional bodies** (Danish rheumatism association)
Hunter (2015) [USA][256]	Knee	RCT	Aged ≥55 years, ambulatory, KL grade II-III, radiographic knee OA, pain on most days due to OA, BMI ≥27 & ≤41, sedentary lifestyle	1) Diet induced weight loss (800-1000kcal per day)<br>2) Exercise – 60 min sessions, 3x per week<br>3) Diet + exercise<br>[Additional outcomes of Messier 2013[26]	1) 152<br>2) 150<br>3) 152	1) 66 (6)<br>2) 66 (6)<br>3) 65 (6)	1) 108 (71.1)<br>2) 108 (72.0)<br>3) 109 (71.7)	**Government** (NIH), **Industry** (General Nutrition Centers, Inc.)
Saraboon (2015) [Thailand][257]	Knee	RCT	OA knee criteria, BMI 23.00-29.99, mild to moderate knee OA, no cognitive deficits, intention to complete study	1) Health education program, quadriceps muscle exercises, home visit program<br>p) OA knee booklet and video	1) 40<br>p) 40	1) 67.5 (7.3)<br>p) 67.3 (6.3)	1) 37 (92.5)<br>p) 37 (92.5)	**Not reported** – Authors declared no conflict of interest

BMI = body mass index, kcal = kilocalories, KL = Kellgren-Lawrence, N = number, OA = osteoarthritis, RCT = randomised controlled trial, SD = standard deviation, USA = United States of America

Table – Osteoarthritis, description of included studies

Author (date) [country]	OA site	Study design	Inclusion criteria	Exposure / intervention detail	N	Age, mean (SD) years	N (%) female	Funders
Beavers (2014) [USA][258]	Knee	RCT	Ambulatory, community dwelling persons aged 55 years or older with: KL II-III of knees, pain on most days due to knee OA, BMI 27-41, sedentary lifestyle	1) Diet induced weight loss (800-1000kcal per day)<br>2) Exercise – 60 min sessions, 3x per week<br>3) Diet + exercise<br>[Additional outcomes of Messier 2013[26]]	1) 88<br>2) 95<br>3) 101	1) 66.0 (6.0)<br>2) 65.8 (6.3)<br>3) 66.1 (6.4)	1) 61 (69.3)<br>2) 71 (74.7)<br>3) 77 (76.2)	**Government** (National Institute of Arthritis and Musculoskeletal and Skin Diseases), **University** (Wake Forest University)
Henriksen (2014) [Denmark][259]	Knee	RCT	Aged >50 years, clinical knee OA from radiograph and BMI >30<br>Exclusions: Lack of motivation to lose weight, inability to speak Danish, planned anti-obesity surgery, receiving pharmacological obesity treatment	Given intensive diet intervention inducing 10% weight loss – patients then enrolled in maintenance study<br>1) Continued diet intervention (1 formula product per day<br>2) Knee exercises<br>p) No attention control	1) 60<br>2) 63<br>p) 64	1) 64.6 (6.6)<br>2) 64.4 (5.8)<br>p) 63.1 (6.8)	Not reported	**Charity** (Oak, Velux, Augustinus, A. P. Moller, Horslev, Bjarne Jensen and Ases and Ejnar Danielsens's, Foundations), **Industry** (Cambridge Weight Plan), **Professional bodies** (Danish rheumatism association)
Messier (2013) [USA][26]	Knee	RCT	Ambulatory, community dwelling persons aged 55 years or older with: KL II-III of knees, pain on most days due to knee OA, BMI 27-41, sedentary lifestyle	1) Diet: Loosing 10% of BL weight with partial meal replacement (women = 1100kcal/day, men = 1200 kcal/day)<br>2) Exercise: 1 hour per session, 3 days per week<br>3) Diet + exercise	1) 152<br>2) 150<br>3) 152	1) 66 (6)<br>2) 66 (6)<br>3) 65 (6)	1) 108 (71.1)<br>2) 108 (72.0)<br>3) 109 (71.7)	**Government** (NIH)
Somers (2012) [USA][260]	Knee	RCT	Pain most day of the months, aged >18, BMI 25-42, ACR criteria for OA and erosions on radiographs, No other major weight bearing joint affected by OA, OA considered medical condition that contributed most to function limitation, able to read/speak English<br>Exclusions: medical condition that increased risk of significant adverse health event during physical activity, non-OA arthropathy/arthritis disorder, regular use of oral corticosteroids, participating in regular exercise / weight-loss program	1) Pain coping skills training<br>2) Behavioural weight loss – group sessions focussing on lifestyle, exercise, attitudes, relationships, nutrition, calorie goal setting. Also exercise program<br>3) Pain coping + behavioural weight loss<br>p) standard care	1) 60<br>2) 59<br>3) 62<br>p) 51	1) 58.1 (11.3)<br>2) 58.3 (11.0)<br>3) 57.5 (9.4)<br>p) 57.9 (10.1)	1) 40 (66.7)<br>2) 47 (79.7)<br>3) 57 (91.9)<br>p) 40 (78.4)	**Government** (NIH)

BMI = body mass index, kcal = kilocalories, KL = Kellgren-Lawrence, N = number, NIH = National Institutes of Health, OA = osteoarthritis, RCT = randomised controlled trialSD = standard deviation, USA = United States of America

Table – Osteoarthritis, description of included studies

Author (date) [country]	OA site	Study design	Inclusion criteria	Exposure / intervention detail	N	Age, mean (SD) years	N (%) female	Funders
Bliddal (2011) [Denmark][261]	Knee	RCT	Knee OA ACR criteria, aged >18 years, desire to lose weight<br>Exclusions: History of other rheumatic diseases, diabetes or other endocrine disease, substantial abnormalities in haematological, hepatic, renal or cardiac function<br>[long-term extension of Christensen 2005[262]]	1) Intensive weight loss diet using formula (810kcal/day)<br>p) Moderate conventional hypo-energetic, high protein diet (approx. 1200kcal/day)	1) 44<br>p) 45	1) 61.1 (11.1)<br>p) 64.1 (10.5)	1) 39 (88.6)<br>p) 40 (88.9)	**Charity** (Oak, Horselv and Bjarne Jensen foundations), **Hospital** (Frederiksberg hospital), **Professional body** (Danish Rheumatism Association)
Gudbergsen (2011) [Denmark][263]	Knee	RCT	Aged ≥18 years, diagnosis of primary knee OA, no other rheumatic diseases, no substantial haematological, hepatic, renal, cardiac or endocrine abnormalities, BMI ≥28, motivation for weight-loss, fluent in Danish	1) Low energy diet for 8 weeks, then hypoenergetic and high protein diet for 24 weeks<br>p) 2 hours nutrition advice session only	1) 15<br>p) 15	62 (6.8)	1) 15 (100)<br>p) 15 (100)	**Charity** (Oak, Velux, Augustinus, A. P. Moller, Horslev, Bjarne Jensen and Ases and Ejnar Danielsens's, Foundations), **Industry** (Cambridge Health and Weight Plan), **Professional bodies** (Danish rheumatism association)
Riecke (2010) [Denmark][264]	Knee	RCT	ACR OA knee criteria, BMI >30, OA radiologically verified<br>Exclusions: previous/planned total knee replacement or other surgery/injections in knee in last 3 months, anti-obesity pharmaceutical treatment, lack of motivation to lose weight, inability to speak Danish fluently, mental state impeding compliance	1) Low calorie diet (810 kcal per day)<br>2) Very low calorie diet (415 kcal per day)<br>Diets consisted of meal replacement powder	1) 96<br>2) 96	1) 63.3 (6.3)<br>2) 61.8 (6.4)	1) 77 (80.2%)<br>2) 78 (81.3%)	**Charity** (Oak, Velux, Augustinus, A. P. Moller, Horslev, Bjarne Jensen and Ases and Ejnar Danielsens's, Foundations), **Industry** (Cambridge Weight Plan), **Professional bodies** (Danish rheumatism association)
Ravaud (2009) [France][265]	Knee	Cluster RCT	aged 45-75 years, ACR knee OA criteria, knee pain 30-70mm on VAS needing treatment with NSAIDs, BMI ≥25 & <35, speaks French<br>Exclusions: Surgery in next 6 months, chronic disease, unable to walk without aid, participating in another nutritional program, electronic implantable device (pacemaker), participating in another clinical trial	1) 3 goal orientated visits to rheumatologist: (1) inform patients about disease and treatment, (2-3) exercise and weight loss<br>p) Usual care	1) 146<br>p) 181	1) 63.9 (8.1)<br>p) 64.6 (8.3)	1) 112 (76.7)<br>p) 132 (72.9)	**Industry** (Almirall SAS)

ACR = American College of Rheumatology, BMI = body mass index, kcal = kilocalories, KL = Kellgren-Lawrence, N = number, NSAIDs = non-steroidal anti-inflammatory drug, OA = osteoarthritis, RCT = randomised controlled trial, SD = standard deviation, VAS = visual analogue scale

Table – Osteoarthritis, description of included studies

Author (date) [country]	OA site	Study design	Inclusion criteria	Exposure / intervention detail	N	Age, mean (SD) years	N (%) female	Funders
Miller (2008) [USA][266]	Knee	RCT	BMI >30, aged ≥60 years, Knee pain and self-reported physician diagnosed OA, self-reported difficulty performing at least 1 of the following: lifting and carrying groceries, walking 1/4 mile, getting in and out of a chair, and going up and down stairs<br>Exclusions: Unable to complete intervention, unstable medical condition, condition where rapid weight loss was contraindicated	1) Aim to lose 10% BL body weight – meal replacements (women = 1100kcal/day, men = 1200 kcal/day), weekly education, behaviour modification, 60 mins 3x per week exercise<br>p) Attention control – met bimonthly for educational sessions	1) 31<br>p) 36	Mean (SE)<br>1) 69.8 (1.0)<br>p) 69.5 (1.0)	1) 64.9%<br>p) 56.7%	**Industry** (SlimFast Nutrition Institute), **University** (Wake Forest University), **Government** (NIH)
Miller (2006) [USA][267]	Knee	RCT	BMI ≥30, age ≥60 years, symptomatic knee OA, difficulty performing one of: lifting/carrying groceries, walking ¼ mile, getting in/out of chair, going up/down stairs<br>Exclusions: Unstable medical conditions where rapid weight loss contraindicated, unwilling to modify diet/physical activity, food allergies, living >50 miles from treatment centre, excessive alcohol consumption	1) Aim to lose 10% BL body weight – meal replacements (women = 1100kcal/day, men = 1200 kcal/day), weekly education, behaviour modification, 60 mins 3x per week exercise<br>p) Attention control – met bimonthly for educational sessions	1) 44<br>p) 43	Mean (SE)<br>1) 69.7 (0.9)<br>p) 69.3 (0.9)	1) 28 (63.6)<br>p) 26 (60.5)	**Industry** (SlimFast Nutrition Institute), **University** (Wake Forest University), **Government** (NIH)
Christensen (2005) [Denmark][262]	Knee	RCT	ACR OA criteria, KL grade II-III, BMI >28, motivated to lose weight, communicate in Danish<br>Exclusions: History / presence of other rheumatic disease, diabetes and other endocrine disorders, Substantial abnormalities in haematological, hepatic, renal or cardiac functions	1) Intensive weight loss diet using formula (810kcal/day)<br>p) Moderate conventional hypo-energetic, high protein diet (approx. 1200kcal/day)	1) 40<br>p) 40	1) 60.5 (11.6)<br>p) 64.6 (10.4)	1) 35 (87.5)<br>p) 36 (90.0)	**Charity** (Oak Foundation), **Professional body** (Danish Rheumatism Association), **Industry** (Dansk Droge)

ACR = American College of Rheumatology, BMI = body mass index, kcal = kilocalories, KL = Kellgren-Lawrence, N = number, OA = osteoarthritis, RCT = randomised controlled trial, SD = standard deviation, USA = United States of America

Table – Osteoarthritis, description of included studies

Author (date) [country]	OA site	Study design	Inclusion criteria	Exposure / intervention detail	N	Age, mean (SD) years	N (%) female	Funders
Messier (2004) [USA][25]	Knee	RCT	Aged ≥60 years, BMI ≥28, knee pain most days of the month, sedentary activity patent, KL grade I-III, willingness to undergo intervention, self-reported difficulty in one of the following activity: walking 1.4 mile, climbing stairs, bending, stooping, kneeling, shopping, house cleaning, getting in/out of bed, standing up from chair, lifting/carrying groceries, getting in/out of bath<br>Exclusions: Serious medical condition precluding safe participation, mini-mental state <24, inability to finish program, inability to walk without cane/device, participation in another study, alcohol consumption of 14 drinks per week, SR segment depression of ≥2 mm at an exercise level of 4 METS or less, hypotension, complex arrhythmia, frail	1) Exercise only (3x per week, aerobic and muscle strengthening)<br>2) Diet weight loss (reduced and maintain 5% weight loss – education only)<br>3) Diet + exercise<br>p) Usual care	1) 82<br>2) 80<br>3) 76<br>p) 78	Mean (SE)<br>1) 68 (0.7)<br>2) 69 (0.8)<br>3) 76 (0.8)<br>p) 69 (0.1)	1) 72%F<br>2) 74%<br>3) 74%<br>p) 68%	**Government** (NIH)
Rejeski (2002) [USA][268]	Knee	RCT	Aged ≥60 years, BMI ≥28, knee pain most days of the month, sedentary activity pattern, radiographic evidence of OA, willingness to participate, limitations in at least one of: walking ¼ mile, climbing stairs, bending, stooping, kneeling, shopping, housecleaning, getting out of bed, standing from chair, lifting/carrying groceries, getting in/out of bath<br>Exclusions: Serious medical condition precluding safe participation, mini-mental state <24, inability to finish program, inability to walk without cane/device, participation in another study, alcohol consumption of 14 drinks per week, frail	1) Diet – goal 5% weight loss<br>2) Exercise – 3x per week, aerobic and muscle strengthening. First four months supervised then at home<br>3) Diet + exercise<br>p) Healthy-lifestyle control	1) 73<br>2) 69<br>3) 68<br>p) 68	1) 68.1 (5.5)<br>2) 69.0 (6.6)<br>3) 68.5 (5.6)<br>p) 68.6 (6.39)	1) 74.07%<br>2) 73.75%<br>3) 73.33%<br>p) 66.67%	**Government** (NIH)

BMI = body mass index, KL = Kellgren-Lawrence, N = number, NIH = National Institutes of Health, OA = osteoarthritis, RCT = randomised controlled trial, SD = standard deviation, SE = standard error, USA = United States of America

Table – Osteoarthritis, description of included studies

Author (date) [country]	OA site	Study design	Inclusion criteria	Exposure / intervention detail	N	Age, mean (SD) years	N (%) female	Funders
Messier (2000) [USA][269]	Knee	RCT	Age ≥60 years, BMI ≥28, knee pain on most days of the month, self-reported difficulty in at least one of the following activities ascribed to knee pain: walking 1/4 mile, climbing stairs, bending, stooping, or kneeling, shopping, housecleaning, or other self-care activities; getting in and out of bed; standing up from a chair; lifting and carrying groceries; or getting in and out of the bathtub, radiographic evidence of tibiofemoral osteoarthritis as determined by a single observer and based on weight-bearing anteroposterior X-rays, willingness to undergo testing and intervention procedures.<br>Exclusions: had a serious medical condition that prevented safe participation in an exercise program, planned to leave the area or be admitted to a nursing home within the next 6 months, were unable to walk at least 420 ft in 6 minutes without a cane or other assistive device, were unable to walk on a treadmill without a cane or other assistive device, were participating in a regular exercise program more than one time per week for 20 minutes per session, were participating in another research study, were unable to participate in most of the facility-based intervention, would not be able to complete the protocol, in the opinion of the clinical staff, because of frailty, illness, or other reasons.	1) Exercise (3x per week, aerobic + muscle strengthening<br>2) Exercise + diet (aim to lose 6.8kg over 6 months – nutrition classes)	1) 11<br>2) 13	1) 69 (5)<br>2) 67 (4)	1) 7 (63.6)<br>2) 10 (76.9)	**Government** (NIH)
Toda (2001) [Japan][270]	Knee	NRT	Aged 45-69 years, knee OA as chief complaint	1) NSAIDs only<br>2) NSAIDs + non-weight bearing exercise<br>3) NSAIDs + walking<br>4) NSAIDs + diet<br>5) NSAIDs + diet and strength exercises<br>6) NSAIDS + diet + walking	1) 52<br>2) 49<br>3) 35<br>4) 29<br>5) 37<br>6) 26	61.1 (9.7)	100%	**Not reported**

BMI = body mass index, N = number, NIH = National Institutes of Health, NRT = non-randomised trial, NSAIDs = non-steroidal anti-inflammatory drug, OA = osteoarthritis, RCT = randomised controlled trial, SD = standard deviation, USA = United States of America

Table – Osteoarthritis, description of included studies

Author (date) [country]	OA site	Study design	Inclusion criteria	Exposure / intervention detail	N	Age, mean (SD) years	N (%) female	Funders
Huang (2000) [Taiwan][271]	Knee	NRT	Knee OA, BMI >25 in men and >30 in women	1) Weight reduction and diet of <500kcal/day + exercise + acupuncture<br>2) Electrotherapy	126	Not reported	112 (88.8)	**Not reported**
Bartholdy (2019) [Denmark][272]	Knee	Single arm int.	Reanalysis of RCT data, ACR knee OA criteria, KL grade I-III, BMI ≥27, motivation for weight loss<br>Exclusions: planned knee surgery, previous / planned treatment for obesity, current medical or dietary treatment	Intensive dietary intervention – full meal replacement diet of 800-1000kcal per day. Also educational sessions	124	59 (10.3)	78 (62.9)	**Industry** (Novo Nordisk, Cambridge Weight Plan), **Professional body** (Danish Physical Therapy Association, Danish Rheumatism Association), **Charity** (Oak Foundation)
Aree-Ue (2017) [Thailand][273]	Knee	Single arm int.	ACR OA criteria, BMI 23-29.99, mild to moderate knee OA, no cognitive impairment<br>Exclusions: secondary knee OA, history of knee surgery, injections in either knee 3 months before study, serious medical conditions (e.g. myocardial infarction) progressive symptom or severe OA	1) Weight loss program – Health education, muscle exercises, home visits<br>p) Knee booklet and video	74	67.6 (6.9)	68 (91.9)	**University** (Mahidol University)
Atukorala (2016) [Australia][274]	Knee	Single arm int.	1986 ACR knee OA criteria, diagnosis supported by radiograph, BMI >28, knee OA symptoms that required referral to an orthopaedic surgeon for evaluation for a knee joint replacement procedure. For this analysis: ≥50 years	Osteoarthritis Healthy Weight For Life Program (OAHWFL) implements none surgical OA best practice treatment recommendations with a target of losing >5% weight – land and water based exercise, education, eating plan, satisfaction tracking, available support via telephone	1383	64 (8.7)	71%	**Government** (National Health and Medical Research Council)
Bartels (2014) [Denmark][275]	Knee	Single arm int.	Aged >50 years, BMI ≥30, ACR Knee OA criteria	Formula weight loss diet 415-810kcal/day for 8 weeks and then 8 weeks of 1200kcal/day	175	62.6 (6.3)	142 (81.1)	**Charity** (Oak, Velux, Augustinus, A. P. Moller, Horslev, Bjarne Jensen and Ases and Ejnar Danielsens's Foundations), **Industry** (Cambridge Weight Plan), **Professional bodies** (Danish rheumatism association)

ACR = American College of Rheumatology, BMI = body mass index, kcal = kilocalories, KL = Kellgren-Lawrence, N = number, NRT = non-randomised trial, OA = osteoarthritis, SD = standard deviation

Table – Osteoarthritis, description of included studies

Author (date) [country]	OA site	Study design	Inclusion criteria	Exposure / intervention detail	N	Age, mean (SD) years	N (%) female	Funders
Paans (2013) [The Netherlands] [276]	Hip	Single arm int.	Radiological evidence of hip OA, BMI >25 & <40<br>Exclusions: conditions preventing safe participation in exercise, problems with foot/ankle that could prevent exercise, rheumatoid arthritis, inability to walk without assistive device, participation in another study, low chance/inability to finish study, language problems/dementia impeding completing questionnaires, future hip replacement	Exercise and weight-loss intervention.<br>Exercise – individual land group sessions focused on improving aerobic capacity.<br>Weight loss – improve awareness of the importance of change, discuss problems encountered	30	56.9 (11.9)	17 (56.7)	**University** (University of Groningen)
Gudbergsen (2012) [Denmark][277]	Knee	Single arm int.	Aged >50 years, BMI ≥30, ACR OA criteria<br>Exclusions: lack of motivation for weight reduction, insufficient understanding, planned anti-obesity operation, former/planned knee replacement, pharmacological obesity treatment, medical disease preventing physical training, active joint disease besides OA, significant hip OA, toe/other deformity influencing gait analysis, use of morphine	16 week dietary program – nutritional education and diet of normal food plus meal replacements	192	62.5 (6.4)	155 (80.7%)	**Charity** (Oak, Velux, Augustinus, A. P. Moller, Horslev, Bjarne Jensen and Ases and Ejnar Danielsens's Foundations), **Industry** (Cambridge Weight Plan), **Professional bodies** (Danish rheumatism association)
Bihlet (2018) [11 countries][278]	Knee	Pros. Cohort	Analysis of placebo arm of 2 RCTs, aged 51-80 years, painful OA in target knee, KL grade II-III on target knee and joint space width ≥2	BMI	771	64.5 (6.5)	491 (63.7)	**Industry** (Novartis)
Han (2018) [USA][279]	Knee	Pros. Cohort	OA initiative, aged 45-79 years, symptomatic knee OA<br>Exclusions: rheumatoid or inflammatory arthritis, end-stage OA defined as severe joint space narrowing in both knees, and bilateral knee replacements	BMI – continuous	1013	61.2	557 (55.0)	**Not reported –** Authors declared no conflict of interest
Jacobs (2018) [USA][280]	Knee	Pros. Cohort	OA initiative	Patients categorised as:<br>1) Obese<br>2) Obese + depression<br>3) Neither	1) 285<br>2) 33<br>3) 282	1) 60.2 (8.4)<br>2) 59.0 (8.3)<br>3) 63.1 (9.1)	1) 176 (61.8)<br>2) 24 (72.7)<br>3) 149 (52.8)	**No funding**
Pelletier (2018) [USA][281]	Knee	Pros. Cohort	OA initiative – radiographic OA (KL grade ≥ I), received hyaluronic acid	BMI – continuous	364	66 (9)	239 (65.7)	**Industry** (Sanofi, Merck, Novartis, GlaxoSmithKline, Pfizer), **Government** (NIH)

BMI = body mass index, KL = Kellgren-Lawrence, N = number, OA = osteoarthritis, RCT = randomised controlled trial, SD = standard deviation, USA = United States of America

Table – Osteoarthritis, description of included studies

Author (date) [country]	OA site	Study design	Inclusion criteria	Exposure / intervention detail	N	Age, mean (SD) years	N (%) female	Funders
Eymard (2017) [France][282]	Knee	Pros. Cohort	Reanalysis of RCT – aged 40-85 years, ACR criteria for OA, failed to response to analgesics and NSAIDs, 3-8 points on 0-10 VAS, KL grade III-IV<br>Exclusions: OA flare with knee OA, tibial plateau or femoral condyle bone attrition, symptomatic hip OA or any other active inflammatory or microcrystal rheumatic disease, excessive varus or valgus knee misalignment (8˚), viscosupplementation in the target knee within the previous 9 months,<br>and systemic/IA corticosteroids use within the previous 3 months	BMI – continuous and categorised as obese or not (BMI >30 = obese)	166	Mean (95% CI) 65.2 (63.6, 66.8)	101 (60.8)	**Industry** (LABRHA)
Moyer (2017) [USA][283]	Knee	Pros. Cohort	OA initiative, 25-79 years, undergone sagittal double-echo steady-state acquisitions at baseline and at 2-year follow-up, OA diagnosis (KL grade ≥2), frequent knee symptoms, no knee replacement during FU,	BMI	558	Varus definite: 60 (8)<br>Varus minor: 63 (11)<br>Neutral: 61 (9)<br>Valgus minor: 64 (11)<br>Valgus definite: 69 (8)	Varus definite: 116 (91.3)<br>Varus minor: 103 (92.8)<br>Neutral: 235 (98.7)<br>Valgus minor: 41 (95.3)<br>Valgus definite: 38 (97.4)	**University** (University of Western Ontario), **Professional body** (Osteoarthritis Research Society)
Bastick (2016) [The Netherlands] [284]	Hip	Pros. Cohort	Cohort Hip and Cohort Knee (Check) study – pain and/or stiffness of the knee and/or hip, aged 45-65 years, consulted physician for symptoms <6 months ago<br>Exclusions: other pathological conditions that could explain symptoms, comorbidity that would not allow physical evaluation malignancy in last 5 years, inability to understand Dutch	BMI – continuous	545	55.7 (5.2)	81%	**Professional body** (Dutch Arthritis Association

ACR = American College of Rheumatology, BMI = body mass index, KL = Kellgren-Lawrence, N = number, NSAIDs = non-steroidal anti-inflammatory drug, OA = osteoarthritis, SD = standard deviation, USA = United States of America

Table – Osteoarthritis, description of included studies

Author (date) [country]	OA site	Study design	Inclusion criteria	Exposure / intervention detail	N	Age, mean (SD) years	N (%) female	Funders
de Rezende (2016) [Brazil][285]	Knee	Pros. Cohort	Aged >45 years, knee OA according to ACR criteria, started treatment in last 6 months, no other RMD, knee pain >30mm<br>Exclusions: Participating in another program with nutritional education, engaging in another clinical trial, undergoing surgery not related to knee OA	BMI – continuous	228	Not reported	152 (66.7)	**No funding**
Beavers (2015) [USA][286]	Knee	Pros. Cohort	Ambulatory, community dwelling persons aged 55 years or older with: KL II-III of knees, pain on most days due to knee OA, BMI 27-41, sedentary lifestyle	1) Diet induced weight loss (800-1000kcal per day)<br>2) Exercise – 60 min sessions, 3x per week<br>3) Diet + exercise<br>[Additional outcomes of Messier 2013[26]]	450	65.6 (6.2)	321 (71.3)	**Government** (National Institute of Arthritis and Musculoskeletal and Skin Diseases), **University** (Wake Forest University)
Chatterjee (2015) [USA][287]	Knee	Pros. Cohort	Bone marrow oedema lesions visible on MRI, complete outcome measures, minimum 6 months between injection and assessment, no additional surgery	BMI	22	54.3 (8.05)	9 (40.9)	**Not reported –** Authors declared no conflict of interest
Karsdal (2015) [Denmark][288]	Knee	Pros. Cohort	RCT reanalysis - ACR OA criteria,<br>KL grade II-III, functional class I-III, joint space width ≥2mm, significant pain (WOMAC pain ≥150mm)	BMI categories:<br>Quartiles	2206	64.4 (6.8)	1430 (64.8)	**Industry** (Nordic Bioscience, CCBR, Novartis, Merck)
Kobayashi (2015) [Japan][289]	Hip	Pros. Cohort	Hips with confirmed dysplastic change on x-ray	BMI – continuous	57	50.8 (11.3)	49 (86.0)	**Not reported –** Authors declared no conflict of interest
Magnusson (2015) [Norway][290]	Hand	Pros. Cohort	Oslo hand OA cohort, aged 50-70 years, hand OA diagnosis, no inflammatory disease	BMI – continuous	103	61.6 (5.6)	94 (91.3)	**Government** (South-Eastern Norway Regional Health Authority)
Gudbergsen (2013) [Denmark][291]	Knee	Pros. Cohort	Reanalysis of RCT, Aged >50 years, BMI ≥30, primary knee OA	16 week dietary program - nutritional education and a diet of normal food plus meal replacements. 8 weeks of intensive weight loss then 8 weeks of part formula part food diet	175	62.7 (6.3)	136 (77.7)	**Charity** (Oak, Velux, Augustinus, A. P. Moller, Horslev, Bjarne Jensen and Ases and Ejnar Danielsens's Foundations), **Industry** (Cambridge Weight Plan), **Professional bodies** (Danish rheumatism association)
Perrot (2013) [France][292]	Hip, knee	Pros. Cohort	Hip or knee OA (ACR criteria), pain in last 24 hours ≥3 out of 10	BMI categories: <18.5, 18.5-25, 25-30, ≥30	hip: 808<br>Knee: 1606	Hip: 68.0 (8.2)<br>Knee : 66.9 (9.0)	Hip: 50.7%<br>Knee: 49.3%	**Industry** (Sanofi France)

BMI = body mass index, kcal = kilocalories, KL = Kellgren-Lawrence, MRI = magnetic resonance imaging, N = number, OA = osteoarthritis, pros. = prospective, RCT = randomised controlled trial, SD = standard deviation, USA = United States of America, WOMAC = Western Ontario and McMaster Universities Osteoarthritis Index

Table – Osteoarthritis, description of included studies

Author (date) [country]	OA site	Study design	Inclusion criteria	Exposure / intervention detail	N	Age, mean (SD) years	N (%) female	Funders
Coffman (2012) [USA][293]	Hand, hip, knee	Pros. Cohort	Physician diagnosis of OA in hand, hip or knee radiographs, self-reported OA symptom on most days in at least 1 month of last year	Self-reported BMI	157	Not reported	81 (51.6)	**Not reported**
Miyazaki (2012) [Japan][294]	Knee	Pros. Cohort	Aged >60 years, knee pain during some daily activities, no knee replacement surgery during follow-up<br>Exclusions: symptomatic musculoskeletal disorders other than those affecting knee joints, history or major trauma/sports injury to knee, rheumatoid arthritis, gout/pseudogout, autoimmune disease, other major systemic disease	BMI – continuous	84	72.3 (3.1)	78 (92.9)	**Not reported**
Rabago (2012) [USA][295]	Knee	Pros. Cohort	Data from RCT but analysed as a cohort, ACR knee OA, existing radiograph within 5 years, tenderness of ≥1 anterior knee structures, moderate-severe knee pain	BMI – continuous	36	60 (8.7)	21 (58.3)	**Government** (NIH, National Center for Complementary and Alternative Medicine)
Sands (2012) [N. / S. America and Europe][296]	Hip, knee	Pros. Cohort	Reanalysis of RCT comparing daily vs celecoxib treatment only when flaring, aged 18-80 years, knee or hip OA criteria	1) Celecoxib daily – stratified into BMI<30 and BMI ≥30<br>2) Celecoxib when patient is flaring – stratified into BMI<30 and BMI ≥30	1) BMI <30: 209<br>1) BMI ≥30: 222<br>2) BMI <30: 205<br>2) BMI ≥30: 222	1) BMI <30: 59.2 (10.2)<br>1) BMI ≥30: 57.8 (9.8)<br>2) BMI <30: 58.9 (10.3)<br>2) BMI ≥30: 58.6 (9.0)	1) BMI <30: 145 (69.4)<br>1) BMI ≥30: 172 (77.5)<br>2) BMI <30: 149 (72.7)<br>2) BMI ≥30: 154 (69.4)	**Industry** (Pfizer)

ACR = American College of Rheumatology, BMI = body mass index, N = number, NIH = National Institutes of Health, OA = osteoarthritis, pros. = prospective, RCT = randomised controlled trial, SD = standard deviation, USA = United States of America

Table – Osteoarthritis, description of included studies

Author (date) [country]	OA site	Study design	Inclusion criteria	Exposure / intervention detail	N	Age, mean (SD) years	N (%) female	Funders
Bartlett (2011) [N. America and Europe][297]	Knee	Pros. Cohort	Placebo arm of KOSTAR trial, signal knee pain due to OA on most days during 1 month, aged >50 years, morning knee stiffness <30 mins, knee crepitus according to ACR knee OA criteria<br>Exclusions: Inflammatory arthritis, BMI >40, cancer in last 10 years, tetracycline use within 6 months, hyaluronan injections within 3 months, calcitonin or fluoride use within 6 months, prior use of bisphosphonates within 12 months or for 60 days ever	BMI – continuous	626	61.9 (8.9)	439 (70.1)	**Industry** (Procter & Gamble), **University** (Johns Hopkins Arthritis Center Discovery Fund)
Bingham (2011) [USA][298]	Hip, knee	Pros. Cohort	reanalysis of an RCT of etoricoxib and celecoxib – aged ≥40 years, symptom duration > 6 months, functional class I-III, required NSAIDs, prior NSAID users must have pain walking on flat surface <80mm on VAS, and after NSAID washout, flare scores defined by a minimum score of 40 mm with an increase of 15 mm from screening level, and investigator global assessment of disease status (IGADS) worsening of at least 1 point on a 5-point Likert scale.	BMI – continuous<br>Three treatment arms, each analysed separately<br>Etoricoxib (E)<br>Celecoxib (C)<br>Placebo (P)	E) 475<br>C) 488<br>P) 244	E) 62.0 (9.9)<br>C) 62.4 (9.4)<br>P) 61.9 (9.2)	E) 323 (68.0)<br>C) 321 (65.8)<br>P) 159 (65.2)	**Industry** (Merck)
Nishimura (2011) [Japan][299]	Knee	Pros. Cohort	Aged ≥65 years	BMI – continuous	92	Progression: 70.7 (4.7)<br>No progression: 71.6 (5.0)	Progression: 3 (13.6)<br>No progression: 53 (75.7)	**No funding**
Richette (2011) [France][300]	Knee	Pros. Cohort	Having obesity surgery, KL grade II-IV of knee, BMI ≥40 or ≥35 with ≥1 comorbidity (hypertension, diabetes, dyslipidaemia, obstructive sleep apnoea syndrome)	Gastric bypass surgery	44	44 (10.3)	36 (81.8)	**Hospital** (Assistance Publique-Hôpitaux de Paris), **Government** (European community), **Professional body** (Association Rhumatisme et Travail)

ACR = American College of Rheumatology, BMI = body mass index, KL = Kellgren-Lawrence, N = number, NSAIDs = non-steroidal anti-inflammatory drug, OA = osteoarthritis, pros. = prospective, SD = standard deviation, USA = United States of America,

Table – Osteoarthritis, description of included studies

Author (date) [country]	OA site	Study design	Inclusion criteria	Exposure / intervention detail	N	Age, mean (SD) years	N (%) female	Funders
Woollard (2011) [USA][301]	Knee	Pros. Cohort	Re-analysis of an RCT – inclusion for RCT: KL grade ≥2, ≥1 compartment of the tibiofemoral joint and diagnosis of OA<br>Exclusions: aged <40 years, history of myocardial infarction, cerebral vascular accident or other neurological disorder, lower extremity joint arthroplasty, inability to walk without device	BMI – continuous	13	63.5 (11.4)	10 (76.9)	**Government** (National Institutes of Arthritis and Musculoskeletal and Skin Diseases)
Yusuf (2011) [The Netherlands][302]	Multiple sites	Pros. Cohort	Caucasian siblings (aged 40-70 years) with symptomatic OA in hands or other joints (KL grade ≥1) in ≥1 knee at baseline<br>Exclusions: secondary OA, familial syndromes with clear Mendelian inheritance, shortened life-expectancy (<1 year)	BMI categories:<br>Normal: ≤25<br>Overweight: 25-30<br>Obese: >30	155	59.6 (7.4)	132 (85.2)	**Industry** (TI-Pharma, Pfizer), **Professional body** (Dutch Arthritis Association)
Shea (2010) [USA][303]	Knee	Pros. Cohort	Reanalysis of RCT, Obese and older men and women with knee OA<br>Exclusions: cardiovascular disease, hypertension, chronic obstructive pulmonary disorder, other comorbidities that could limit mobility and participation in regular exercise	Patients divide into weight loss (WL) or no weight loss (NWL). Original treatment groups were:<br>1) Dietary weight loss<br>2) Exercise<br>3) Diet + exercise	WL: 159<br>NWL: 159	WL: 68.2 (6.1)<br>NWL: 69.0 (6.3)"	WL: 72.3%<br>NWL: 71.2%	**University** (Wake Forest University)
Eckstein (2009) [USA][304]	Knee	Pros. Cohort	Osteoarthritis Initiative, frequent knee symptoms, radiographic OA in at least their knees<br>Exclusions: rheumatoid or inflammatory arthritis, bilateral end-stage knee OA, inability to walk without aids and MRI contraindications	BMI categories:<br>1) BMI <25<br>2) BMI 25-30<br>3) BMI 30-35<br>4) BMI≥35	156	60.9 (9.9)	79 (50.6)	**Industry** (Pfizer)
Le Graverand (2009) [USA][305]	Knee	Pros. Cohort	Aged ≥40 years, definite radiographic OA at baseline (KL grade II-III), BMI 30-55, Knee pain/aching/stiffness on most days during the past year and/or treatment for knee pain most days in past year	BMI – continuous	60	KL grade II: 55.5 (7.4)<br>KL grade III: 58.2 (8.3)	60 (100)	**Industry** (Pfizer)

BMI = body mass index, KL = Kellgren-Lawrence, N = number, OA = osteoarthritis, pros. = prospective, RCT = randomised controlled trial, SD = standard deviation, USA = United States of America

Table – Osteoarthritis, description of included studies

Author (date) [country]	OA site	Study design	Inclusion criteria	Exposure / intervention detail	N	Age, mean (SD) years	N (%) female	Funders
Botha-Scheepers (2008) [The Netherlands] [306]	Knee	Pros. Cohort	Genetics, Arthrosis and Progression (GARP) study. OA at multiple sites in the hands or hand, spine, knee or hip, aged 40-70 years<br>Exclusions: secondary OA, familial syndromes with a Mendelian inheritance pattern or a shortened life expectancy	BMI <30 vs BMI ≥30	88	Median (IQR): 59.6 (55.3 - 66.6)	67 (76.1)	**Industry** (Pfizer)
Davies-Tuck (2008) [Australia][307]	Knee	Pros. Cohort	Aged >40 years, fulfilling ACR clinical and radiographic criteria for OA, pain and osteophytes present<br>Exclusions: other forms of arthritis, contraindication to MRI, planned knee replacement	Weight and BMI	117	63.7 (10.2)	68 (58.1)	**Government** (National Health and Medical Research Council of Australia), **Professional body** (Royal Australian College of Physicians)
Pelletier (2007) [Canada][308]	Knee	Pros. Cohort	Reanalysis of RCT – radiographic knee OA, minimum joint space width between 2-4mm<br>Exclusions: chondrocalcinosis or an acute or chronic infection or if there OA of the knee was secondary to other conditions. History of past or present gastrointestinal ulcerations, receipt of an intra-articular corticoid injection in knee within 6 months of study, KL grade IV, functional class IV	BMI – continuous	107	62.4 (7.5)	64%	**Industry** (Procter and Gamble, ArthroVision)
Reijman (2007) [The Netherlands] [309]	Hip, knee	Pros. Cohort	Rotterdam study – aged ≥55 years, radiographic OA at baseline (KL I-III)	BMI categories:<br>≤25, >25-27.5, >27.5	Hip: 1676<br>Knee: 532	Hip: 66.1 (6.9)<br>Knee: 68.6 (7.0)	Hip: 52.1%<br>Knee: 68.4%	**Not reported –** Authors declared no conflict of interest
Raynauld (2006) [Canada][310]	Knee	Pros. Cohort	Reanalysis of RCT – aged 40-80 years, ACR knee OA criteria, radiographic evidence of OA, joint space width 2-4mm<br>Exclusions: chondrocalcinosis or an acute or chronic infection or if there OA of the knee was secondary to other conditions. History of past or present gastrointestinal ulcerations, receipt of an intra-articular corticoid injection in knee within 6 months of study, KL grade IV, functional class IV:	BMI – continuous	107	Slow cartilage loss: 60.9 (7.5)<br>Intermediate: 63.0 (7.7)<br>Fast: 66.0 (5.0)	Slow cartilage loss: 68%<br>Intermediate: 64%<br>Fast: 45%	**Industry** (Procter and Gamble)

ACR = American College of Rheumatology, BMI = body mass index, KL = Kellgren-Lawrence, N = number, OA = osteoarthritis, pros. = prospective, RCT = randomised controlled trial, SD = standard deviation, USA = United States of America

Table – Osteoarthritis, description of included studies

Author (date) [country]	OA site	Study design	Inclusion criteria	Exposure / intervention detail	N	Age, mean (SD) years	N (%) female	Funders
Wluka (2006) [Australia][311]	Knee	Pros. Cohort	Aged >40 years, mild symptomatic knee OA (i.e. ≥1 pain dimension on WOMAC above 20% and osteophytes)<br>Exclusions: other forms of arthritis, contraindication to MRI, inability to walk 50 feet without assistive device, hemiparesis of either lower limb, planned knee replacement	BMI – continuous	105	63.8 (10.6)	59 (56.2)	**Government** (National Health and Medical Research Council of Australia)
Sharma (2003) [USA][312]	Knee	Pros. Cohort	Presence of definite tibiofemoral osteophytes (KL II) confirmed by radiograph, rating of at least "a little difficulty" on ≥2 WOMAC function scales	BMI – 5 unit increases	236	68.6 (10.8)	172 (72.9)	**Government** (National Institute of Arthritis and Musculoskeletal and Skin Diseases, National Center for Research Resources)
Cicuttini (2002) [Australia][313]	Knee	Pros. Cohort	Aged ≥40 years, ARA criteria for knee OA, radiographic evidence of osteophytes / joint space narrowing<br>Exclusions: Any other form of arthritis, contraindications to MRI, total knee replacement planned	BMI – continuous	110	63.2 (10.2)	66 (60.0)	**Government (**National Health and Medical Research Council)
Wolfe (2002) [USA][314]	Knee, hip	Pros. Cohort	Knee or hip OA based on clinical criteria (ACR)	BMI tertiles	1507	63.4 (11.8)	77%	**Industry** (Roche), **Charity** (Rosaline Russell Research Fund)
Detora (2001) [27 countries][315]	Knee, hip	Pros. Cohort	Patients taking part in a 3 trials of rofecoxib	BMI tertiles	1501	mean (range)<br>Study 1: 61.9 (38-92)<br>Study 2: 61.0 (39-91)<br>Study 3: 63.4 (40-86)	Study 1: 306 (72)<br>Study 2: 385 (75)<br>Study 3: 452 (81)	**Industry** (Merck)
Cooper (2000) [UK][316]	Knee	Pros. Cohort	Aged ≥55 years, KL grade II-III	BMI categories:<br>Low (<22.7), Middle (22.7-25.4), High (>25.4)	354	70.2	72%	**Charity** (Arthritis Research Campaign)
Harris (1994) [UK][317]	Hand	Pros. Cohort	Hand or knee OA	BMI – continuous	169	60	122 (72.2)	**Not reported**

ACR = American College of Rheumatology, ARA = American Rheumatism Association, BMI = body mass index, KL = Kellgren-Lawrence, MRI = magnetic resonance imaging, N = number, OA = osteoarthritis, pros. = prospective, SD = standard deviation, USA = United States of America, WOMAC = Western Ontario and McMaster Universities Osteoarthritis Index

Table – Osteoarthritis, description of included studies

Author (date) [country]	OA site	Study design	Inclusion criteria	Exposure / intervention detail	N	Age, mean (SD) years	N (%) female	Funders
Ledingham (1993) [UK][318]	Hip	Pros. Cohort	Presence of radiographic change together with pain or clinical abnormalities.	BMI	136	median (range): 65 (29-85)	85 (62.5)	**Government** (Trent Regional Health Authority)
Schouten (1992) [The Netherlands][319]	Knee	Pros. Cohort	Doctor diagnosed local OA, KL grade ≥II	BMI – quartiles: <24.35, 24.35-25.96, 25.97-27.73, >27.73	142	57.2 (6.1)	84 (59.2)	**Government** (Dutch Ministry of Science and Education, Ministry of Welfare, Public Health and Culture)
Berkhout (1985) [The Netherlands][320]	Knee	Pros. Cohort	Radiographs compatible with the clinical diagnosis of RA, localised or generalised OA<br>Exclusions: No patient with a history of knee trauma,	Categorised as obese or not obese	72	63.5	54 (75)	**Not reported**
Ahn (2016) [South Korea][321]	Knee	Retro. Cohort	Had meniscus allographic transplantation, 6 months of knee pain despite treatment and surgery<br>Exclusions: Lack of post-operative MRI, loss to follow-up for a minimum of 3 years, simultaneous surgery on articular cartilage of anterior cruciate ligament	BMI – continuous	69	No progression: 37.9 (8.9)<br>Progression: 35.4 (8.1)	No progression: 10 (26.3%)<br>Progress: 7 (22.6%)	**Not reported –** Authors declared no conflict of interest

BMI = body mass index, KL = Kellgren-Lawrence, MRI = magnetic resonance imaging, N = number, OA = osteoarthritis, pros. = prospective, Retro. = retrospective, SD = standard deviation, UK = United Kingdom

Supplementary table 91 – Pain outcomes from weight-loss interventions in OA

Table – Pain (OA), results and quality assessment

Study (date) [study type]	Standardised result, SMD (95% CI) unless otherwise stated	Natural result	Supports intervention	AMSTAR2 quality	Rand. Seq.	Alloc. Conc.	Blind. Part.	Blind. Asses.
Corbett (2013) [MA][15]	Trials of better quality<br>SMD -0.08 (-0.55, 0.39) vs standard care<br>Trials of any quality<br>SMD -0.26 (-0.67, 0.15) vs standard care		×	Moderate				
Le Quintrec (2014) [SR][24]		Only patients in the diet + exercise groups have improvements in pain	✓	Critically low				
Fernandes (2013) [SR][36]		Weight loss combined with exercise improves pain and function. EULAR recommends weight loss for patients with OA	✓	Critically low §				
Sadeghi (2019) [RCT][251]	Diet intervention vs control at 3 months<br>SMD -0.17 (-0.67, 0.33)	WOMAC pain, BL / 3 months, mean (SD)<br>Diet intervention: 248 (90) / 213.50 (96.6)<br>Control: 234 (115) / 232.01 (117)	×		H/UC	H/UC	H/UC	H/UC
O'Brien (2018) [RCT][252]	Telephone intervention vs control at week 26<br>SMD 0.00 (-0.36, 0.36)	WOMAC pain, BL / week 26, mean (SD)<br>Telephone weight loss: 9.0 (3.8) / 9.5 (3.5)<br>Control: 9.8 (4.1) / 9.5 (4.1)	×		L	L	H/UC	L
Allen (2017) [RCT][253]	Patient intervention vs control, change BL-12 months<br>SMD -0.13 (-0.37, 0.12)<br>Provider intervention vs control, change BL-12 months<br>SMD 0.05 (-0.19, 0.29)<br>Patient + provider intervention vs control, change BL-12 months<br>SMD -0.10 (-0.34, 0.14)	WOMAC pain, change BL-12 months, mean (SD ‡)<br>Patient intervention: -1.5 (4.0)<br>Provider intervention: -0.8 (3.9)<br>Patient + provider interventions: -1.4 (4.2)<br>Usual care: -1.0 (3.8)	×		L	L	H/UC	L
Allen (2016) [RCT][254]		WOMAC pain, difference between intervention and control at 12 months (95% CI)<br>-0.5 (-1.2, 0.2)	×		L	H/UC	H/UC	L

‡ SD calculated from 95% CI

§ Fernandes (2013) is a recommendations paper and so there is little information on the systematic review that was conducted to support the recommendations

Alloc. Conc. = allocation concealment, AMSTAR2 = Assessing the Methodological Quality of Systematic Reviews, BL = baseline, Blind. Asses. = Blinded assessors, Blind. Part. = blinded participants, CI = confidence interval, H/UC = high / unclear risk of bias, L = low risk of bias, MA = meta-analysis, OA = osteoarthritis, Rand. Seq. = random sequence generation, SMD = Standardised mean difference, SR = systematic review, WOMAC = Western Ontario and McMaster Universities Osteoarthritis Index

Table – Pain (OA), results and quality assessment

Study (date) [study type]	Standardised result, SMD (95% CI) unless otherwise stated	Natural result	Supports intervention	AMSTAR2 quality	Rand. Seq.	Alloc. Conc.	Blind. Part.	Blind. Asses.
Christensen (2015) [RCT][255]	Diet vs control, change BL-68 weeks<br>SMD -0.03 (-0.38, 0.32)<br>Exercise vs control, change BL-68 weeks<br>SMD -0.01 (-0.35, 0.34)	Pain VAS, change BL-68 weeks, mean (SD ‡)<br>Diet: -6.1 (20.4)<br>Exercise: -5.6 (20.2)<br>Control: -5.5 (20.4)	✗		L	L	H/UC	L
Saraboon (2015) [RCT][257]	Health education vs control<br>Both knees: SMD -2.77 (-3.38, -2.15)<br>Left knee: SMD -1.11 (-1.58, -0.64)<br>Right knee: SMD -1.71 (-2.22, -1.19)	Knee pain at 6 weeks, mean (SD)<br>*Both knees*<br>Health education: 1.84 (1.61)<br>Control: 6.32 (1.63)<br>*Left knee*<br>Health education: 2.50 (2.17)<br>Control: 5.29 (2.81)<br>*Right knee*<br>Health education: 2.86 (2.11)<br>Control: 5.63 (0.91)	✓		H/UC	H/UC	H/UC	H/UC
Messier (2013) [RCT][26]	Diet + exercise vs diet at 18 months<br>SMD -0.31 (-0.54, -0.09)<br>Diet + exercise vs exercise at 18 months<br>SMD -0.21 (-0.44, 0.02)	WOMAC pain, BL / 18 months, mean (SD ‡)<br>Diet 6.6 (3.1) / 4.8 (3.5)<br>Exercise: 6.1 (3.1) / 4.4 (3.1)<br>Diet + exercise: 6.7 (3.5) / 3.7 (3.5)	✓		L	H/UC	H/UC	L
Somers (2012) [RCT][260]	Pain coping vs control at week 24<br>SMD -0.24 (-0.62, 0.13)<br>Weight loss vs control at week 24<br>SMD -0.18 (-0.56, 0.20)<br>Pain coping + weight loss vs control at week 24<br>SMD -0.80 (-1.18, -0.41)	WOMAC pain, BL / 24 weeks, mean (SD ‡)<br>Pain coping: 42.8 (22.1) / 34.5 (14.6)<br>Weight loss: 42.6 (19.2) / 35.5 (13.9)<br>Pain coping + weight loss: 47.7 (22.5) / 27.2 (13.1)<br>Control: 43.4 (22.0) / 38.0 (14.0)	✓		L	L	H/UC	L
Bliddal (2011) [RCT][261]	Very low calories vs low calories, change BL-52 weeks<br>SMD -0.49 (-0.91, -0.07)	WOMAC pain, change BL-52 weeks, mean (SD †)<br>Very low calories: -7.7 (14.6)<br>Low calories: -0.5 (14.8)	✓		L	L	H/UC	L
Riecke (2010) [RCT][264]	Low vs very low calories, change BL-16 weeks<br>SMD 0.06 (-0.22, 0.34)	WOMAC Pain, change BL-16 week, mean (SD †)<br>Low calorie: -10.5 (17.9) ;<br>Very low calorie: -11.6 (18.6) p=0.68	✗		L	L	H/UC	L

† SD calculated from standard error

‡ SD calculated from 95% CI

Alloc. Conc. = allocation concealment, AMSTAR2 = Assessing the Methodological Quality of Systematic Reviews, BL = baseline, Blind. Asses. = Blinded assessors, Blind. Part. = blinded participants, CI = confidence interval, H/UC = high / unclear risk of bias, L = low risk of bias, OA = osteoarthritis, Rand. Seq. = random sequence generation, RR = risk ratio, SMD = Standardised mean difference, VAS = visual analogue scale, WOMAC = Western Ontario and McMaster Universities Osteoarthritis Index

Table – Pain (OA), results and quality assessment

Study (date) [study type]	Standardised result, SMD (95% CI) unless otherwise stated	Natural result	Supports intervention	AMSTAR2 quality	Rand. Seq.	Alloc. Conc.	Blind. Part.	Blind. Asses.
Ravaud (2009) [RCT][265]	Weight-loss session vs control, change BL-4 months<br>SMD -0.19 (-0.41, 0.03)<br>Weight-loss session vs control, change BL-12 months<br>SMD -0.19 (-0.41, 0.03)<br>[significant after using propensity score adjustments]	Pain VAS, change bl-4 months / bl-12 mth, mean (SD)<br>Weight-loss sessions: -1.65 (2.32) / -1.35 (2.48)<br>Control: -1.18 (2.58) / -0.86 (2.59)	✓		L	L	H/UC	H/UC
Miller (2006) [RCT][267]	Weight loss vs control at 6 months<br>SMD -0.66 (-1.10, -0.23)	WOMAC pain, BL / 6 months, mean (SD †)<br>Weight loss: 6.5 (3.3) / 4.1 (2.7)<br>Control: 6.3 (3.3) / 6.1 (3.3)	✓		H/UC	H/UC	H/UC	H/UC
Christensen (2005) [RCT][262]	Very low calories vs low calories, change BL-8 weeks<br>SMD -0.16 (-0.60, 0.28)	WOMAC pain, change BL-8 weeks, mean (SD †)<br>Very low calories: -57.0 (106.9)<br>Low calories: -29.8 (111.9)	×		L	H/UC	H/UC	H/UC
Messier (2004) [RCT][25]	Diet vs control at 18 months<br>SMD -0.13 (-0.44, 0.18)<br>Exercise vs control at 18 months<br>SMD 0.05 (-0.26, 0.37)<br>Exercise + diet vs control at 18 months<br>SMD -0.24 (-0.55, 0.08)	WOMAC pain, bl / 18 months, mean (SD †)<br>Diet: 6.58 (3.6) / 5.51 (4.1)<br>Exercise: 6.64 (3.5) / 6.24 (4.2)<br>Exercise + diet: 7.27 (3.6) / 5.07 (4.1)<br>Control: 7.25 (3.4) / 6.02 (4.0)	×		L	L	H/UC	L
Rejeski (2002) [RCT][268]	Diet vs control at 18 months<br>SMD 0.32 (-0.01, 0.66)<br>Exercise vs control at 18 months<br>SMD 0.25 (-0.09, 0.59)<br>Diet + exercise vs control at 18 months<br>SMD 0.61 (0.26, 0.95)	SF-36 pain, BL / 18 months, mean (SD †)<br>Diet: 49.38 (19.0) / 56.71 (21.1)<br>Exercise: 51.30 (19.5) / 55.03 (19.4)<br>Diet + exercise: 50.25 (19.9) / 62.41 (20.6)<br>Control: 45.49 (18.2) / 50.09 (19.9)	✓		H/UC	H/UC	H/UC	L
Bespoke meta-analysis including[25;251-253;255;257;260;265;267;268]	Weight loss interventions vs control<br>SMD -0.31 (-0.49, -0.13), I^2 82.6%<br>Excluding 1 outlier[257]: SMD -0.20 (-0.30, -0.09), I^2 48.2%		✓					

† SD calculated from standard error

Alloc. Conc. = allocation concealment, AMSTAR2 = Assessing the Methodological Quality of Systematic Reviews, BL = baseline, Blind. Asses. = Blinded assessors, Blind. Part. = blinded participants, CI = confidence interval, H/UC = high / unclear risk of bias, L = low risk of bias, OA = osteoarthritis, Rand. Seq. = random sequence generation, SMD = Standardised mean difference, VAS = visual analogue scale, WOMAC = Western Ontario and McMaster Universities Osteoarthritis Index

Table – Pain (OA), results and quality assessment

Study (date) [study type]	Standardised result, SMD (95% CI) unless otherwise stated	Natural result	Supports intervention	AMSTAR2 quality	Rand. Seq.	Alloc. Conc.	Blind. Part.	Blind. Asses.
Huang (2000) [NRT][271]		Pain VAS, BL / 12 weeks, mean (SD †)<br>Diet therapy-II) 4.4 (1.3) / 2.6 (0.6)<br>Diet therapy-III) 6.9 (1.2) / 4.7 (1.2)<br>Diet therapy -IV) 8.5 (2.1) / 4.7 (0.7)<br>Electrotherapy-II) 4.5 (1.0) / 3.0 (0.6)<br>Electrotherapy-III) 6.7 (1.2) / 5.0 (1.6)<br>Electrotherapy-IV) 8.3 (1.8) / 5.6 (1.9)<br>[numerals after dash indicated KL grade]	✓					
Bartholdy (2019) [Single arm int][272]		KOOS pain, change bl-8 weeks, mean (95% CI)<br>Unadjusted: 12.8 (10.6, 15.0)<br>Adjusted: 13.0 (10.8, 15.3)	✓					
Aree-Ue (2017) [Single arm int.][273]		Pain VAS, Change BL – 12 months, mean (SE)<br>left knee = 4.80 (0.53)<br>right knee = 5.52 (0.61)	✓					
Atukorala (2016) [Single arm int.][274]		KOOS pain, mean change BL - 18 weeks (SD)<br>≤2.5% weight loss: 6.1 (13.0)<br>2.5-5% weight loss: 9.9 (16.8)<br>5-7.5% weight loss: 12.0 (17.1)<br>7.5-10% weight loss: 13.3 (15.1)<br>>=10% weight loss: 16.7 (16.1)	✓					
Bartels (2014) [Single arm int.][275]		KOOS pain, mean change BL – 16 weeks<br>Unadjusted: 10.7 (SD = 14.5)<br>Adjusted: 10.0 (95% CI 7.3, 12.7)	✓					
Paans (2013) [Single arm int.][276]		VAS Pain, BL / 8 months, mean (SE)<br>[recoded VAS so that 10 = best outcome]<br>3.7 (0.3) / 6.8 (0.4)	✓					
Gudbergsen (2012) [Single arm int.][277]		KOOS pain, median percentage improvement over 16 weeks<br>14%	✓					

† SD calculated from standard error

Alloc. Conc. = allocation concealment, AMSTAR2 = Assessing the Methodological Quality of Systematic Reviews, BL = baseline, Blind. Asses. = Blinded assessors, Blind. Part. = blinded participants, CI = confidence interval, H/UC = high / unclear risk of bias, KOOS = knee injury and osteoarthritis outcome score, OA = osteoarthritis, Rand. Seq. = random sequence generation, SD = standard deviation, SE = standard error, VAS = visual analogue scale

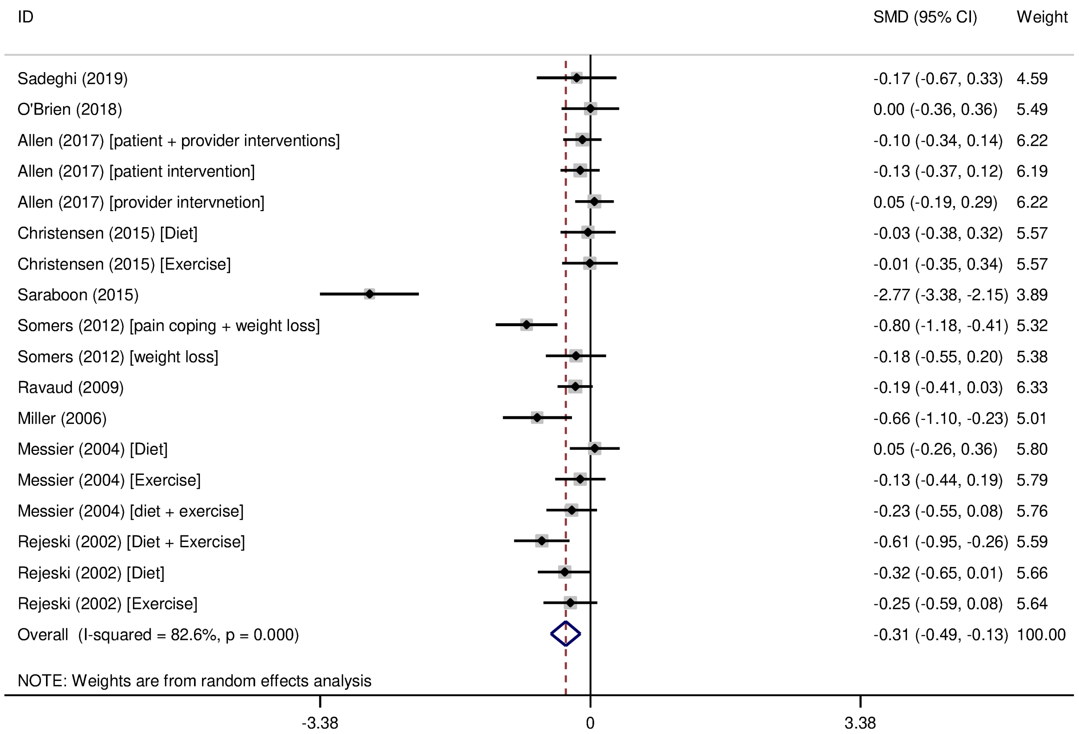

Figure – Weight loss interventions vs control - outcome = pain

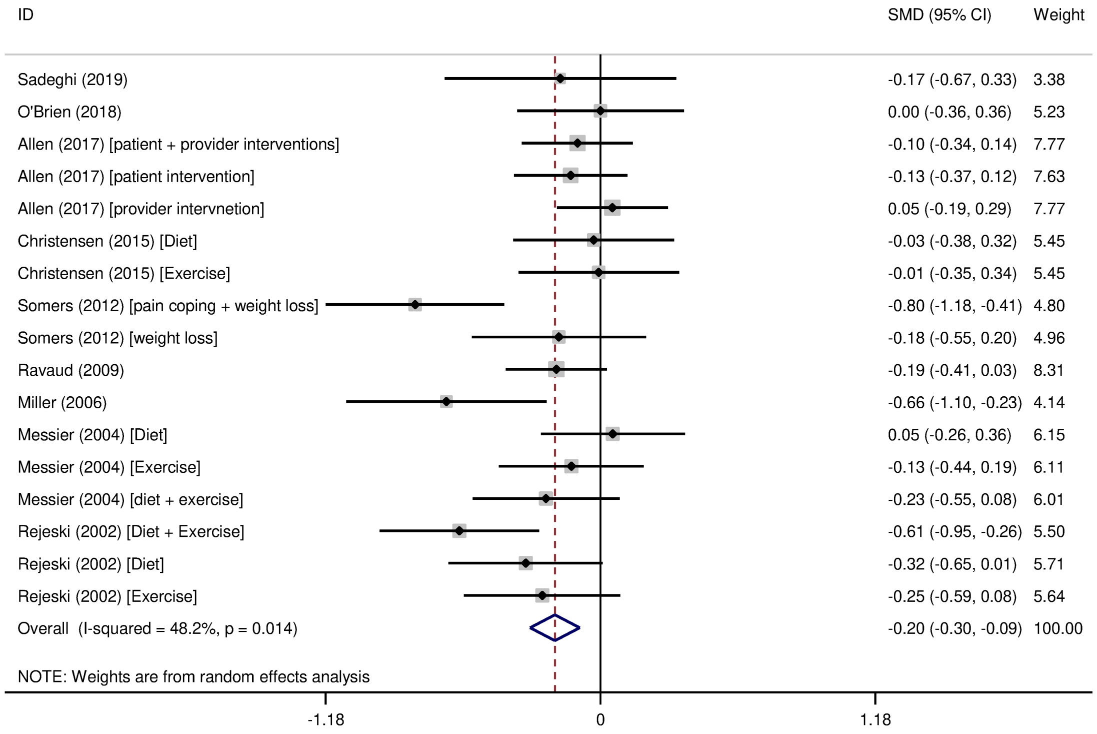

Figure – Weight loss interventions vs control - outcome = pain [excluding 1 outlier – Saraboon et al]

Supplementary table 92 – Pain outcomes from observational studies in OA

Table – Pain (OA), results and quality assessment of observational studies

Study (date) [study type]	Results	Weight associated with outcome	AMSTAR2	Study Pop.	Attr.	Prog. Meas.	Outc. Meas.	Conf.	Stats.
Pozzobon (2018) [MA][18]	Pain after arthroplasty, non-obese vs obese<br>Short-term: SMD -0.44 (-0.68, -0.20)<br>Long-term: SMD -0.36 (-0.47, -0.25)	✓	Low						
de Rooij (2016) [SR][38]	One study reported no association between BMI and pain	×	Moderate						
de Rooij (2016) [SR][22]	Four out of six studies reported that BMI predicts pain	✓	Moderate						
Bihlet (2018) [Prospective cohort][278]	Baseline BMI was not associated with change in pain	×		L	M	L	L	H	H
Jacobs (2018) [Prospective cohort][280]	WOMAC pain, BL / 2 years, mean (SD)<br>Obese: 2.4 (3.0) / 2.9 (3.2)<br>Obese + depression: 5.5 (4.6) / 7.1 (4.6)<br>Neither: 2.1 (2.9) / 2.7 (3.3)	×		L	L	L	L	L	M
Bastick (2016) [Prospective cohort][284]	Pain trajectories, mean (SD) BL BMI<br>Mild pain: 25 (4)<br>Moderate decrease: 26 (4)<br>Moderate progression: 27 (5)<br>Severe pain: 27 (5)<br>Pain trajectory, univariable RR (95%Ci) per unit increase in BMI<br>Mild pain: ref<br>Moderate decrease: 1.07 (1.00, 1.14)<br>Moderate progression: 1.10 (1.04, 1.16)<br>Severe pain: 1.12 (1.05, 1.19)	✓		L	M	L	L	M	M
de Rezende (2016) [Prospective cohort][285]	Correlation change BMI and change WOMAC Pain over one year<br>r=0.199, p=0.006<br>Correlation between change in BMI and WOMAC pain at 1 year<br>r=-0.193, p=0.007	✓		L	L	L	L	H	M
Kobayashi (2015) [Prospective cohort][289]	Pain progression, OR (95%CI)<br>BMI: 1.01 (0.88, 1.16) [unadj] / 0.10 (0.81, 1.22) [adj] [sic]	×		M	L	L	L	L	M
Magnusson (2015) [Prospective cohort][290]	AUSCAN pain, over follow-up, regression coefficient (95% CI)<br>BMI longitudinal: -0.02 (-0.37, 0.33) [adjusted]	×		L	M	L	L	L	M

Attr. = attrition, AUSCAN = Australian Canadian Osteoarthritis Hand Index, BL = baseline, BMI = body mass index, CI = confidence interval, Conf. = confounding, L = low risk of bias, M = moderate risk of bias, MA = meta-analysis, Outc. Meas = outcome measurement, Prog. Meas. = prognostic factor measurement, Pros = prospective, Rand. Seq. = random sequence generation, SD = standard deviation, SMD = Standardised mean difference, SR = systematic review, Stats. = statistical analysis, Study Pop. = study population, WOMAC = Western Ontario and McMaster Universities Osteoarthritis Index

Table – Pain (OA), results and quality assessment of observational studies

Study (date) [study type]	Results	Weight associated with outcome	Study Pop.	Attr.	Prog. Meas.	Outc. Meas.	Conf.	Stats.
Perrot (2013) [Prospective cohort][292]	lack of PASS pain, OR (95% CI) [adj]<br>hip<br><18.5: OR 1.89 (0.11, 33.33)<br>18.5-25: ref<br>25-30: 0.91 (0.62, 1.35)<br>≥30: 1.64 (1.02, 2.63)<br>knee<br>BMI not assessed for knee OA	✓	L	M	L	L	M	M
Coffman (2012) [Prospective cohort][293]	Pain, regression coefficient (SE) [adjusted]<br>Overweight vs normal: 10.36 (4.16) p=0.01<br>Obese vs normal: 17.04 (4.25) p<0.0001	✓	L	M	L	L	L	M
Rabago (2012) [Prospective cohort][295]	Repeated measurement model:<br>BMI ≤25 kg/m2 (p=0.04) associated with improvement in WOMAC score over 1 yr. (no effect size)	✓	M	L	H	L	L	M
Sands (2012) [Prospective cohort][296]	WOMAC pain, change BL-22 weeks, mean (SE) [unadjusted]<br>Celecoxib daily: BMI<30: 0.32 (0.23); BMI≥30: 0.40 (0.21)<br>Celecoxib flare: BMI<30: 1.05 (0.23); BMI≥30: 1.31 (0.21)	✗	L	L	M	L	H	M
Richette (2011) [Prospective cohort][300]	Pain VAS, BL / after surgery, mean (SD)<br>50 (26.6) / 24.5 (21) p<0.0001<br>WOMAC pain, BL / after surgery, mean (SD)<br>187.3 (124.4) / 94.1 (93.9) p<0.0001	✓	L	M	L	L	M	M

Attr. = attrition, BL = baseline, BMI = body mass index, CI = confidence interval, Conf. = confounding, L = low risk of bias, M = moderate risk of bias, OR = odds ratio, Outc. Meas = outcome measurement, PASS = patient acceptable symptom state, Prog. Meas. = prognostic factor measurement, Pros = prospective, Rand. Seq. = random sequence generation, SD = standard deviation, SE = standard error, Stats. = statistical analysis, Study Pop. = study population, VAS = visual analogue scale, WOMAC = Western Ontario and McMaster Universities Osteoarthritis Index

Supplementary table 93 – Function outcomes from weight-loss interventions in OA

Table – Function (OA), results and quality assessment

Study (date) [study type]	Standardised result, SMD (95% CI) unless otherwise stated	Natural result	Supports intervention	AMSTAR2 quality	Rand. Seq.	Alloc. Conc.	Blind. Part.	Blind. Asses.
Le Quintrec (2014) [SR][24]		Only patients in the diet + exercise groups have improvements in function	✓	Critically low				
Sadeghi (2019) [RCT][251]	Diet intervention vs control at 3 months<br>SMD -0.06 (-0.56, 0.44)	WOMAC Function, BL / 3 months, mean (SD)<br>Diet intervention: 752 (351) / 631.94 (361.2)<br>Control: 638 (402) / 655.35 (409.26)	×		H/UC	H/UC	H/UC	H/UC
O'Brien (2018) [RCT][252]	Telephone intervention vs control at week 26<br>SMD 0.26 (-0.10, 0.62)	WOMAC function, BL / week 26 mean (SD)<br>Telephone weight loss: 34.9 (12.6) / 36.5 (13.2)<br>Control: 34.5 (12.2) / 32.8 (15.1)	×		L	L	H/UC	L
Allen (2017) [RCT][253]	Patient intervention vs control, change BL-12 months<br>SMD -0.10 (-0.34, 0.15)<br>Provider intervention vs control, change BL-12 months<br>SMD 0.23 (-0.02, 0.47)<br>Patient + provider intervention vs control, change BL-12 months<br>SMD -0.02 (-0.26, 0.22)	WOMAC Function, change from BL to 12 months (SD ‡)<br>Patient intervention: -5.6 (10.7)<br>Provider intervention: -2.3 (10.3)<br>Patient + provider intervention: -4.8 (10.9)<br>Usual care: -4.6 (10.1)	×		L	L	H/UC	L
Allen (2016) [RCT][254]		WOMAC function, difference between intervention and control at 12 months (95% CI)<br>-3.3 (-5.7, -1.0)	✓		L	H/UC	H/UC	L
Christensen (2015) [RCT][255]	Diet vs control, change BL-68 weeks<br>SMD 0.07 (-0.28, 0.42)<br>Exercise vs control, change BL-68 weeks<br>SMD 0.06 (-0.28, 0.41)	VAS disability, change BL-68 weeks, mean (SD ‡)<br>Diet: -7.5 (21.8)<br>Exercise: -7.6 (22.0)<br>Control: -9.0 (22.0)	×		L	L	H/UC	L
Messier (2013) [RCT][26]	Diet + exercise vs diet at 18 months<br>SMD -0.29 (-0.51, -0.06)<br>Diet + exercise vs exercise at 18 months<br>SMD -0.30 (-0.53, -0.07)	WOMAC Function, BL / 18 months, mean (SD ‡)<br>Diet: 24.8 (10.4) / 17.7 (12.9)<br>Exercise: 23.1 (10.6) / 17.6 (11.2)<br>Diet + exercise: 24.6 (12.0) / 14.2 (11.6)	✓		L	H/UC	H/UC	L

‡ SD calculated from 95% CI

Alloc. Conc. = allocation concealment, AMSTAR2 = Assessing the Methodological Quality of Systematic Reviews, BL = baseline, Blind. Asses. = Blinded assessors, Blind. Part. = blinded participants, CI = confidence interval, H/UC = high / unclear risk of bias, L = low risk of bias, OA = osteoarthritis, Rand. Seq. = random sequence generation, RCT = randomised controlled trial, SD = standard deviation, SMD = Standardised mean difference, SR = systematic review, WOMAC = Western Ontario and McMaster Universities Osteoarthritis Index

Table – Function (OA), results and quality assessment

Study (date) [study type]	Standardised result, SMD (95% CI) unless otherwise stated	Natural result	Supports intervention	AMSTAR2 quality	Rand. Seq.	Alloc. Conc.	Blind. Part.	Blind. Asses.
Somers (2012) [RCT][260]	Pain coping vs control at week 24<br>SMD -0.17 (-0.55, 0.20)<br>Weight loss vs control at week 24<br>SMD -0.11 (-0.49, 0.26)<br>Pain coping + weight loss vs control at week 24<br>SMD -0.96 (-1.36, -0.57)	WOMAC Function, BL / 24 weeks, mean (SD ‡)<br>Pain coping: 46.2 (20.2) / 35.2 (13.4)<br>Weight loss: 44.3 (18.6) / 36.0 (13.1)<br>Pain coping + weight loss: 47.7 (21.9) / 25.1 (12.5)<br>Control: 46.1 (23.3) / 37.5 (13.3)	✓		L	L	H/UC	L
Bliddal (2011) [RCT][261]	Very low vs low calories, change BL-52 weeks<br>SMD -0.27 (-0.69, 0.15)	WOMAC Function, change BL-52 weeks, mean (SD †)<br>Very low calories: -7.5 (13.3)<br>Low calories: -3.9 (13.4)	×		L	L	H/UC	L
Gudbergsen (2011) [RCT][263]		WOMAC Function, change BL-6 months, mean difference between low calorie and control (95% CI)<br>-266 (-468.9, -63.1)	✓		L	L	H/UC	L
Riecke (2010) [RCT][264]	Low vs very low calories, change BL-16 weeks<br>SMD 0.08 (-0.20, 0.37)	WOMAC function, change BL-16 week, mean (SD †)<br>Low calories: -12.75 (18.9)<br>Very low calories: -14.44 (22.0)	×		L	L	H/UC	L
Ravaud (2009) [RCT][265]	Weight-loss session vs control, change BL-4 months<br>SMD -0.16 (-0.37, 0.06)<br>Weight-loss session vs control, change BL-12 months<br>SMD -0.26 (-0.48, -0.04)	WOMAC Function, change bl-4 months / bl-12 months, mean (SD)<br>Weight loss sessions: -5.74 (10.66) / -8.67 (12.05)<br>Control: -4.03 (11.35) / -5.44 (12.97)	✓		L	L	H/UC	H/UC
Miller (2006) [RCT][267]	Weight loss vs control at 6 months<br>SMD -0.74 (-1.18, -0.31)	WOMAC Function, BL / 6 months, mean (SD †)<br>Weight loss: 24.0 (9.9) / 15.2 (9.9)<br>Control: 26.7 (12.5) / 23.8 (13.1)	✓		H/UC	H/UC	H/UC	H/UC
Christensen (2005) [RCT][262]	Very low vs low calories, change BL-8 weeks<br>SMD -0.52 (-0.97, -0.07)	WOMAC Function, change BL-8 weeks, mean (SD †)<br>Very low calories: -252.5 (313.7)<br>Low calories: -85.6 (328.2)	✓		L	H/UC	H/UC	H/UC

† SD calculated from standard error

‡ SD calculated from 95% CI

Alloc. Conc. = allocation concealment, AMSTAR2 = Assessing the Methodological Quality of Systematic Reviews, BL = baseline, Blind. Asses. = Blinded assessors, Blind. Part. = blinded participants, CI = confidence interval, H/UC = high / unclear risk of bias, L = low risk of bias, OA = osteoarthritis, Rand. Seq. = random sequence generation, RCT = randomised controlled trial, SD = standard deviation, SMD = Standardised mean difference, WOMAC = Western Ontario and McMaster Universities Osteoarthritis Index

Table – Function (OA), results and quality assessment

Study (date) [study type]	Standardised result, SMD (95% CI) unless otherwise stated	Natural result	Supports intervention	AMSTAR2 quality	Rand. Seq.	Alloc. Conc.	Blind. Part.	Blind. Asses.
Messier (2004) [RCT][25]	<u>Diet vs control, change BL-16 months</u><br>SMD 0.06 (-0.25, 0.37)<br><u>Exercise vs control, change BL-16 months</u><br>SMD -0.03 (-0.34, 0.28)<br><u>Exercise + diet vs control, change BL-16 months</u><br>SMD 0.17 (-0.14, 0.49)	<u>WOMAC Function, Change BL-18 months mean (SD ‡)</u><br>Diet: 4.23 (13.7)<br>Exercise: 3.07 (9.6)<br>Exercise + diet: 5.73 (13.8)<br>Control: 3.40 (13.2)	×		L	L	H/UC	L
Bespoke meta-analysis including[25;251-253;255;260;265;267]	<u>Weight loss interventions vs control</u><br>SMD -0.08 (-0.23, 0.08), I^2 69.8%		×					
Toda (2001) [NRT][270]	<u>NSAIDs + non-weight bearing exercise vs NSAIDs only, change BL-8 weeks</u><br>SMD -0.67 (-1.07, -0.27)<br><u>NSAIDs + walking vs NSAIDs only, change BL-8 weeks</u><br>SMD -0.19 (-0.62, 0.24)<br><u>NSAIDs + diet vs NSAIDs only, change BL-8 weeks</u><br>SMD -0.58 (-1.04, -0.12)<br><u>NSAIDs + diet + strength exericses vs NSAIDs only, change BL-8 weeks</u><br>SMD -1.64 (-2.13, -1.15)<br><u>NSAIDs + diet + walking vs NSAIDs only, change BL-8 weeks</u><br>SMD -0.73 (-1.22, -0.25)	<u>Function (Lequesne index), mean (SD) change BL – 8 weeks</u><br>NSAIDs only: -0.39 (2.8)<br>NSAIDs + non-weight bearing exercises: -2.4 (3.2)<br>NSAIDs + walking: -1.1 (4.7)<br>NSAIDs + diet: -2.5 (4.8)<br>NSAIDs + diet + strength exercises: -6.3 (4.5)<br>NSAIDS + diet + walking: -2.6 (3.4)	✓					
Bartholdy (2019) [Single arm int.][272]		<u>KOOS Function, change bl-8 weeks, mean (95%CI)</u><br>Unadjusted: 14.5 (12.6, 16.4)<br>Adjusted: 14.6 (12.6, 16.5)	✓					
Atukorala (2016) [Single arm int.][274]		<u>KOOS Function mean change BL - 18 weeks (SD)</u><br>≤2.5% weight loss: 7.8 (13.3)<br>2.5-5% weight loss: 8.9 (14.7)<br>5-7.5% weight loss: 12.0 (16.7)<br>7.5-10% weight loss: 13.6 (15.5)<br>>=10% weight loss: 17.4 (16.3)	✓					

‡ SD calculated from 95% CI

Alloc. Conc. = allocation concealment, AMSTAR2 = Assessing the Methodological Quality of Systematic Reviews, BL = baseline, Blind. Asses. = Blinded assessors, Blind. Part. = blinded participants, CI = confidence interval, H/UC = high / unclear risk of bias, KOOS = knee injury and osteoarthritis outcome score, L = low risk of bias, NRT = non-randomised trial, OA = osteoarthritis, Rand. Seq. = random sequence generation, RCT = randomised controlled trial, SD = standard deviation, SMD = Standardised mean difference, WOMAC = Western Ontario and McMaster Universities Osteoarthritis Index

Table – Function (OA), results and quality assessment

Study (date) [study type]	Standardised result, SMD (95% CI) unless otherwise stated	Natural result	Supports intervention	AMSTAR2 quality	Rand. Seq.	Alloc. Conc.	Blind. Part.	Blind. Asses.
Bartels (2014) [Single arm int.][275]		KOOS Function, mean change from BL-16 weeks, unadjusted mean (SD) / adjusted mean (95% CI) 12.1 (14.1) / 12.5 (10.0, 15.1)	✓					
Paans (2013) [Single arm int.][276]		WOMAC Function, BL / 8 months, mean (SD †) [recoded WOMAC so that 100 = best outcome] 53.0 (15.9) / 70.3 (14.8)	✓					

‡ SD calculated from 95% CI

Alloc. Conc. = allocation concealment, AMSTAR2 = Assessing the Methodological Quality of Systematic Reviews, BL = baseline, Blind. Asses. = Blinded assessors, Blind. Part. = blinded participants, CI = confidence interval, H/UC = high / unclear risk of bias, KOOS = knee injury and osteoarthritis outcome score, L = low risk of bias, OA = osteoarthritis, Rand. Seq. = random sequence generation, RCT = randomised controlled trial, SD = standard deviation, SMD = Standardised mean difference, WOMAC = Western Ontario and McMaster Universities Osteoarthritis Index

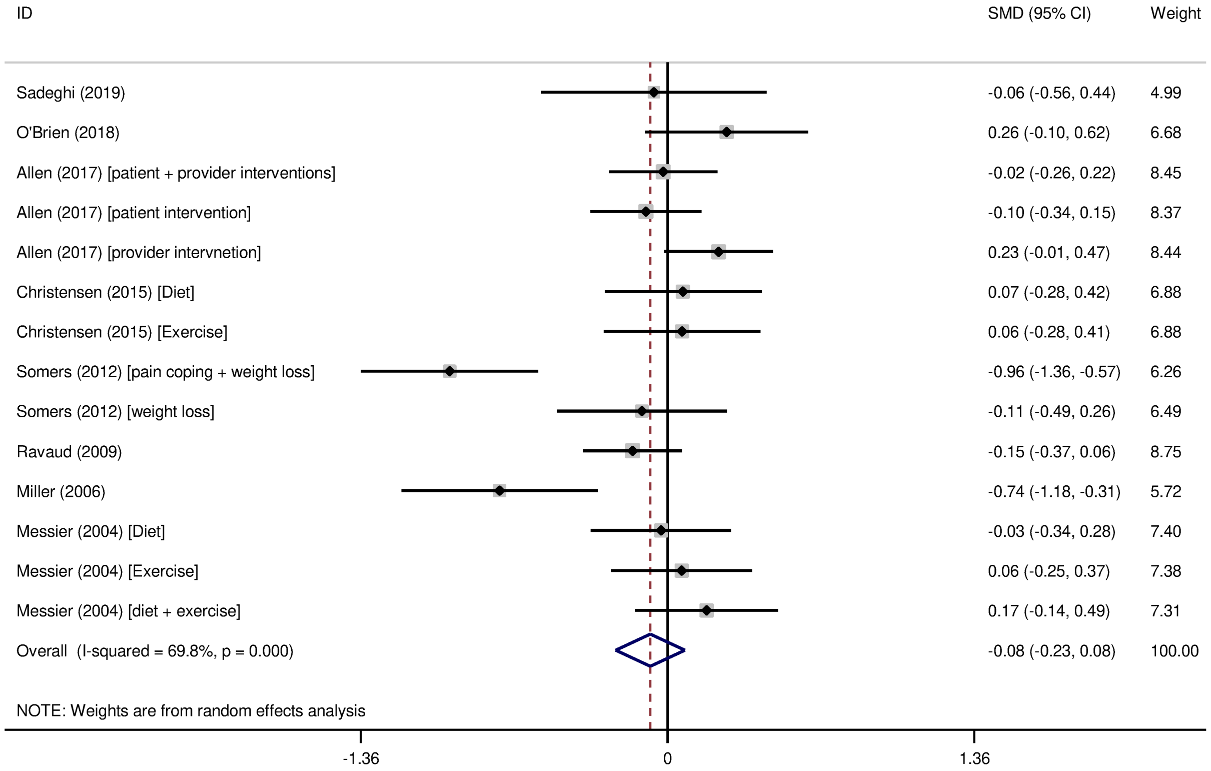

Figure – Weight loss interventions vs control - outcome = Function

Supplementary table 94 – Function outcomes from observational studies in OA

Table – Function (OA), results and quality assessment of observational studies

Study (date) [study type]	Results	Weight associated with outcome	AMSTAR2	Study Pop.	Attr.	Prog. Meas.	Outc. Meas.	Conf.	Stats.
Pozzobon (2018) [MA][18]	<u>Function after arthroplasty, non-obese vs obese</u><br>Short-term: SMD -0.16 (-0.42, 0.10)<br>Long-term: SMD -0.32 (-0.37, -0.28)	✓	Low						
de Rooij (2016) [SR][38]	One out of three studies reported BMI was associated with function in hip OA	×	Moderate						
de Rooij (2016) [SR][22]	Six out of 10 studies reported that BMI predicts pain	✓	Moderate						
Jacobs (2018) [Prospective cohort][280]	<u>WOMAC Function, BL / 2 years, mean (SD)</u><br>Obese: 9.1 (10.3) / 9.4 (10.4)<br>Obese and depressed: 20.7 (16.1) / 25.1 (14.4)<br>Not obese: 6.8 (9.2) / 7.9 (9.9)	×		L	L	L	L	L	M
Magnusson (2015) [Prospective cohort][290]	<u>AUSCAN function, over follow-up, regression coefficient (95% CI)</u><br>BMI longitudinal: -0.15 (-0.71, 0.41)	×		L	M	L	L	L	M
Sands (2012) [Prospective cohort][296]	<u>WOMAC function, change BL-22 weeks, mean (SE) [unadjusted]</u><br>Celecoxib daily: BMI<30: 0.89 (0.74); BMI >=30: 1.36 (0.71)<br>Celecoxib flare: BMI<30: 3.40 (0.74); BMI>=30: 3.45 (0.71)	×		L	L	M	L	H	M
Richette (2011) [Prospective cohort][300]	<u>WOMAC function, BL / after surgery, mean (SD)</u><br>643.9 (424.2) / 272.6 (289) p<0.0001	✓		L	M	L	L	M	M
Sharma (2003) [Prospective cohort][312]	<u>Poor physical function WOMAC score, OR (95%CI)</u><br>Unadjusted: OR 1.26 per 5 unit increase in BMI (1.01-1.57)<br>Adjusted: OR 1.14 per 5 unit increase in BMI (0.89-1.46)	×		L	L	L	L	L	M

Attr. = attrition, AUSCAN = Australian Canadian Osteoarthritis Hand Index, BL = baseline, BMI = body mass index CI = confidence interval, Conf. = confounding, L = low risk of bias, M = moderate risk of bias, MA = meta-analysis, Outc. Meas = outcome measurement, Prog. Meas. = prognostic factor measurement, Pros = prospective, Rand. Seq. = random sequence generation, SD = standard deviation, SE = standard error, SMD = Standardised mean difference, SR = systematic review, Stats. = statistical analysis, Study Pop. = study population, VAS = visual analogue scale, WOMAC = Western Ontario and McMaster Universities Osteoarthritis Index

Supplementary table 95 – Stiffness outcomes from weight-loss interventions in OA

Table – Stiffness (OA), results and quality assessment

Study (date) [study type]	Standardised result, SMD (95% CI) unless otherwise stated	Natural result	Supports intervention	AMSTAR2 quality	Rand. Seq.	Alloc. Conc.	Blind. Part.	Blind. Asses.
Sadeghi (2019) [RCT][251]	Diet intervention vs control at 3 months<br>SMD -0.24 (-0.74, 0.26)	WOMAC Stiffness, BL / 3 months, mean (SD)<br>Diet intervention: 87 (53) / 65.48 (50.1)<br>Control: 79 (64) / 79.03 (61.9)	×		H/UC	H/UC	H/UC	H/UC
O'Brien (2018) [RCT][252]	Telephone intervention vs control at week 26<br>SMD -0.10 (-0.46, 0.26)	WOMAC Stiffness, BL / week 26 mean (SD)<br>Telephone weight loss: 4.0 (2.1) / 4.0 (2.0)<br>Control: 4.3 (1.5) / 4.2 (1.9)	×		L	L	H/UC	L
Somers (2012) [RCT][260]	Pain coping vs control at week 24<br>SMD -0.10 (-0.48, 0.27)<br>Weight loss vs control at week 24<br>SMD -0.04 (-0.41, 0.34)<br>Pain coping + weight loss vs control at week 24<br>SMD -0.63 (-1.01, -0.25)	WOMAC Stiffness, BL / 24 weeks, mean (SD ‡)<br>Pain coping: 54.7 (25.3) / 44.5 (18.8)<br>Weight loss: 50.7 (23.7) / 45.7 (17.6)<br>Pain coping + weight loss: 61.5 (23.5) / 35.4 (16.9)<br>Control: 53.2 (26.8) / 46.4 (18.2)	✓		L	L	H/UC	L
Bliddal (2011) [RCT][261]	Very low vs low calories, change BL-52 weeks<br>SMD -0.13 (-0.54, 0.29)	WOMAC Stiffness, change bl-52 weeks, mean (SD †)<br>Very low calories: -6.2 (17.9)<br>Low calories -3.9 (18.1)	×		L	L	H/UC	L
Miller (2006) [RCT][267]	Weight loss vs control at 6 months<br>SMD -0.06 (-0.48, 0.36)	WOMAC Stiffness, BL / 6 months, mean (SD †)<br>Weight loss: 3.3 (1.3) / 3.0 (1.3)<br>Control: 3.6 (1.3) / 3.1 (2.0)	×		H/UC	H/UC	H/UC	H/UC
Christensen (2005) [RCT][262]	Very low vs low calories, change BL-8 weeks<br>SMD -0.27 (-0.71, 0.17)	WOMAC Stiffness, change BL-8 weeks, mean (SD †)<br>Very low calories: -22.6 (45.5)<br>Low calories: -10.2 (46.8)	×		L	H/UC	H/UC	H/UC
Bespoke meta-analysis including[251;252;260;267]	Weight loss interventions vs control<br>SMD -0.21 (-0.44, 0.01), I^2 36.7%		✓					
Paans (2013) [Single arm int.][276]		WOMAC Stiffness, BL / 8 months, mean (SD †)<br>[recoded WOMAC so that 100 = best outcome]<br>49.6 (3.6) / 66.4 (2.3)	✓					

† SD calculated from standard error
‡ SD calculated from 95% CI
Alloc. Conc. = allocation concealment, AMSTAR2 = Assessing the Methodological Quality of Systematic Reviews, BL = baseline, Blind. Asses. = Blinded assessors, Blind. Part. = blinded participants, CI = confidence interval, H/UC = high / unclear risk of bias, L = low risk of bias, OA = osteoarthritis, Rand. Seq. = random sequence generation, RCT = randomised controlled trial, SD = standard deviation, SMD = Standardised mean difference, WOMAC = Western Ontario and McMaster Universities Osteoarthritis Index

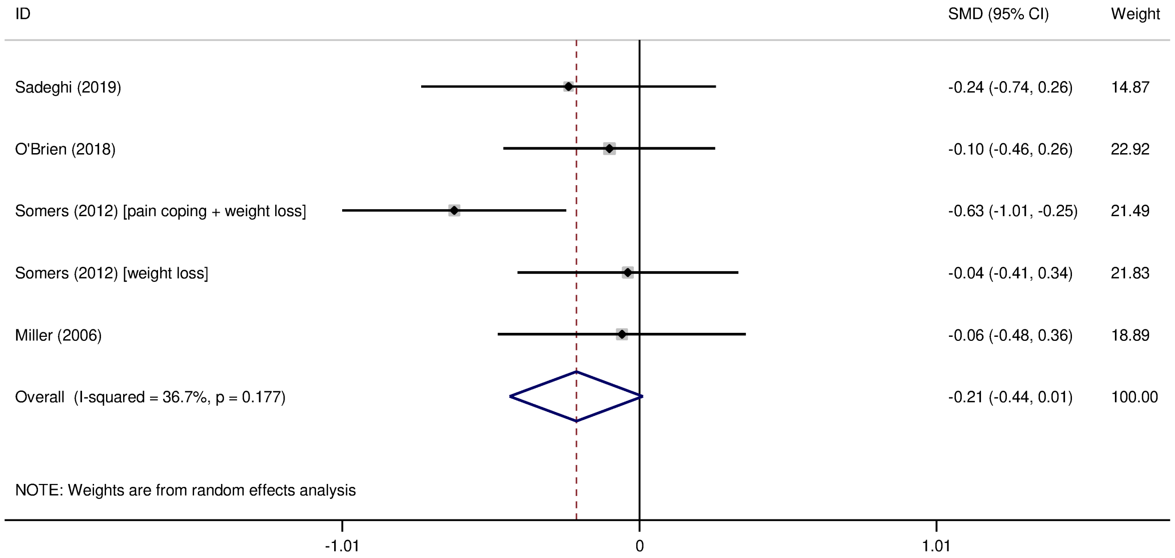

Figure – Weight loss interventions vs control - outcome = stiffness

Supplementary table 96 – Stiffness outcomes from observational studies in OA

Table – Stiffness (OA), results and quality assessment of observational studies

Study (date) [study type]	Results	Weight associated with outcome	Study Pop.	Attr.	Prog. Meas.	Outc. Meas.	Conf.	Stats.
Sands (2012) [Prospective cohort][296]	WOMAC Stiffness, change BL-22 weeks, mean (SE) [unadjusted]<br>Celecoxib daily: BMI<30: 0.10 (0.10); BMI>=30: 0.13 (0.10)<br>Celecoxib flare: BMI<30: 0.42 (0.10); BMI>=30: 0.38 (0.10)	×	L	L	L	L	H	M
Richette (2011) [Prospective cohort][300]	WOMAC Stiffness, BL / after surgery, mean (SD)<br>68.2 (53.8) / 36.4 (41.9) <0.0001	✓	L	M	L	L	M	M

Attr. = attrition, BL = baseline, BMI = body mass index, Conf. = confounding, L = low risk of bias, M = moderate risk of bias, Outc. Meas = outcome measurement, Prog. Meas. = prognostic factor measurement, Pros = prospective, Rand. Seq. = random sequence generation, SD = standard deviation, SE = standard error, SMD = Standardised mean difference, Stats. = statistical analysis, Study Pop. = study population, WOMAC = Western Ontario and McMaster Universities Osteoarthritis Index

Supplementary table 97 – QoL outcomes from weight-loss interventions in OA

Table – QoL (OA), results and quality assessment

Study (date) [study type]	Standardised result, SMD (95% CI) unless otherwise stated	Natural result	Supports intervention	AMSTAR2 quality	Rand. Seq.	Alloc. Conc.	Blind. Part.	Blind. Asses.
Christensen (2015) [RCT][255]	Diet vs control, change BL-68 weeks<br>SMD 0.18 (-0.16, 0.53)<br>Exercise vs control, change BL-68 weeks<br>SMD 0.03 (-0.32, 0.37)	KOOS QoL, change BL-68 weeks, mean (SD ‡)<br>Diet: 8.2 (15.1)<br>Exercise: 5.8 (15.1)<br>Control: 5.4 (15.3)	×		L	L	H/UC	L
Riecke (2010) [RCT][264]	Low vs very low calories, change BL-16 weeks<br>SMD 0.03 (-0.25, 0.32)	KOOS QoL, change BL-16 week, mean (SD †)<br>Low calories: 8.85 (15.7)<br>Very low calories: 8.31 (16.1)	×		L	L	H/UC	L
Bartholdy (2019) [Single arm int.][272]		KOOS QoL, change bl-8 weeks, mean (95% CI)<br>Unadjusted: 8.9 (6.5, 11.4)<br>Adjusted: 8.6 (6.0, 11.2)	✓					
Bartels (2014) [Single arm int.][275]		KOOS QOL, mean change from BL-16 weeks, unadjusted mean (SD) / adjusted mean (95% CI)<br>9.4 (16.4) / 8.1 (5.1, 11.1)	✓					

Alloc. Conc. = allocation concealment, AMSTAR2 = Assessing the Methodological Quality of Systematic Reviews, BL = baseline, Blind. Asses. = Blinded assessors, Blind. Part. = blinded participants, CI = confidence interval, H/UC = high / unclear risk of bias, KOOS = knee injury and osteoarthritis outcome score, L = low risk of bias, QoL = quality of life, OA = osteoarthritis, Rand. Seq. = random sequence generation, RCT = randomised controlled trial, SD = standard deviation, SMD = Standardised mean difference

Supplementary table 98 – QoL outcomes from observational studies in OA

Table – QoL (OA), results and quality assessment of observational studies

Study (date) [study type]	Results	Weight associated with outcome	Study Pop.	Attr.	Prog. Meas.	Outc. Meas.	Conf.	Stats.
Han (2018) [Prospective cohort][279]	QOL trajectory, RR (95% CI) [Adjusted]<br>moderate vs low QOL – RR 0.95 per unit BMI (0.91, 0.99)<br>high vs low QOL - RR 0.93 per unit BMI (0.88, 0.98)	✓	L	M	L	L	L	L
Jacobs (2018) [Prospective cohort][280]	KOOS QoL, BL / 2 years, mean (SD)<br>Obese: 64.1 (19.7) / 66.3 (21.8)<br>Obese and depressed: 48.7 (24.6) / 44.3 (21.6)<br>Not obese: 66.8 (20.7) / 68.7 (21.9)	×	L	L	L	L	L	M

Attr. = attrition, Bl = baseline, BMI = body mass index, CI = confidence interval, Conf. = confounding, KOOS = knee injury and osteoarthritis outcome score, L = low risk of bias, M = moderate risk of bias, Outc. Meas = outcome measurement, Prog. Meas. = prognostic factor measurement, Pros = prospective, QoL = quality of life, Rand. Seq. = random sequence generation, RR = relative risk, SD = standard deviation, SE = standard error, SMD = Standardised mean difference, Stats. = statistical analysis, Study Pop. = study population

Supplementary table 99 – Patient global outcomes from weight-loss interventions in OA

Table – Patient global (OA), results and quality assessment

Study (date) [study type]	Standardised result, SMD (95% CI) unless otherwise stated	Natural result	Supports intervention	AMSTAR2 quality	Rand. Seq.	Alloc. Conc.	Blind. Part.	Blind. Asses.
Christensen (2015) [RCT][255]	Diet vs control, change BL-68 weeks<br>SMD 0.05 (-0.30, 0.40)<br>Exercise vs control, change BL-68 weeks<br>SMD 0.07 (-0.27, 0.42)	Patient global VAS, change BL-68 weeks, mean (SD ‡)<br>Diet: -5.1 (20.4)<br>Exercise: -4.6 (20.4)<br>Control: -6.1 (20.4)	×		L	L	H/UC	L
Riecke (2010) [RCT][264]	Low vs very low calories, change BL-16 weeks<br>SMD -0.09 (-0.38, 0.19)	WOMAC Global, change BL-16 week, mean (SD †)<br>Low calories: -11.54 (20.5)<br>Very low calories: -9.64 (20.8)	×		L	L	H/UC	L
Ravaud (2009) [RCT][265]	Weight-loss session vs control, change BL-4 months<br>SMD -0.32 (-0.54, -0.10)<br>Weight-loss session vs control, change BL-12 months<br>SMD -0.35 (-0.57, -0.13)	Patient global VAS, change bl-4 months / bl-12 months, mean (SD)<br>Weight-loss sessions: -1.66 (2.26) / -1.40 (2.56)<br>Control: -0.90 (2.48) / -0.51 (2.59)	✓		L	L	H/UC	H/UC
Bespoke meta-analysis including[255;265]	Weight loss interventions vs control<br>SMD -0.09 (-0.37, 0.18), I^2 61.1%		×					

Alloc. Conc. = allocation concealment, AMSTAR2 = Assessing the Methodological Quality of Systematic Reviews, BL = baseline, Blind. Asses. = Blinded assessors, Blind. Part. = blinded participants, CI = confidence interval, H/UC = high / unclear risk of bias, L = low risk of bias, OA = osteoarthritis, Rand. Seq. = random sequence generation, RCT = randomised controlled trial, SMD = Standardised mean difference, VAS = visual analogue scale

Supplementary table 100 – Patient global outcomes from observational studies in OA

Table – Patient global (OA), results and quality assessment of observational studies

Study (date) [study type]	Results	Weight associated with outcome	Study Pop.	Attr.	Prog. Meas.	Outc. Meas.	Conf.	Stats.
Richette (2011) [Prospective cohort][300]	Patient global VAS, BL / after surgery, mean (SD)<br>51.6 (26.5) / 25.3 (20.9) p<0.0001	✓	L	M	L	L	M	M
Detora (2001) [Prospective cohort][315]	Patient global, difference from placebo (95% CI)<br>12.5mg rofecoxib:<br>≤28 BMI: -0.7 (-0.9, -0.5)<br>28-33 BMI: -0.9 (-1.1, -0.6)<br>≥33 BMI: -1.1 (-1.3, -0.8)<br>25mg<br>≤28 BMI: -1.0 (-1.3, -0.8)<br>28-33 BMI: -0.9 (-1.1, -0.6)<br>≥33 BMI: -1.1 (-1.3, -0.9)	×	L	M	M	L	M	M
Berkhout (1985) [Prospective cohort][320]	Global improvement score, obese / not obese, %<br>Localised OA: 56% / 54%<br>Generalised OA: 32% / 62%	✓	L	L	L	M	H	H

Attr. = attrition, BL = baseline, BMI = body mass index, CI = confidence interval, Conf. = confounding, L = low risk of bias, M = moderate risk of bias, Outc. Meas = outcome measurement, Prog. Meas. = prognostic factor measurement, Pros = prospective, Rand. Seq. = random sequence generation, SD = standard deviation, SE = standard error, Stats. = statistical analysis, Study Pop. = study population, VAS = visual analogue scale

Supplementary table 101 – Depression outcomes from weight-loss interventions in OA

Table – Depression (OA), results and quality assessment

Study (date) [study type]	Standardised result, SMD (95% CI) unless otherwise stated	Natural result	Supports intervention	AMSTAR2 quality	Rand. Seq.	Alloc. Conc.	Blind. Part.	Blind. Asses.
Allen (2016) [RCT][254]		Depression (PHQ8), difference between intervention and control at 12 months (95% CI)<br>-0.6 (-1.5, 0.3)	×		L	H/UC	H/UC	L

Alloc. Conc. = allocation concealment, AMSTAR2 = Assessing the Methodological Quality of Systematic Reviews, Blind. Asses. = Blinded assessors, Blind. Part. = blinded participants, CI = confidence interval, H/UC = high / unclear risk of bias, L = low risk of bias, OA = osteoarthritis, PHQ-8 = Patient Health Questionnaire depression scale, Rand. Seq. = random sequence generation, RCT = randomised controlled trial

Supplementary table 102 – Radiographic outcomes from observational studies in OA

Table – Radiographic progression (OA), results and quality assessment of observational studies

Study (date) [study type]	Results	Weight associated with outcome	AMSTAR2	Study Pop.	Attr.	Prog. Meas.	Outc. Meas.	Conf.	Stats.
Bastick (2015) [SR][23]	Conflicting evidence – 12/25 studies reported a significant association	×	Moderate						
Chatterjee (2015) [Prospective cohort][287]	BMI not associated with Tegner Lysholm knee score (no data)	×		L	M	L	L	H	H
Magnusson (2015) [Prospective cohort][290]	KL sum score (0-120) over follow-up, regression coefficient (95% CI)<br>BMI longitudinal: -0.27 (-0.83, 0.29) [adjusted]	×		L	M	L	L	L	M
Miyazaki (2012) [Prospective cohort][294]	Radiographic progression, OR (95%CI)<br>OR per unit BMI: 1.24 (1.04, 1.45) [adjusted]	✓		L	M	L	L	L	M
Nishimura (2011) [Prospective cohort][299]	Progression of knee OA, OR (95% CI)<br>OR per unit BMI 0.932 (0.779, 1.114) [adjusted]	×		L	L	L	L	L	M
Woollard (2011) [Prospective cohort][301]	Comparison progression versus non-progression:<br>median BMI = 30 vs 26 [no statistical test performed]	×		H	L	L	L	H	H
Yusuf (2011) [Prospective cohort][302]	OA progression, adjusted RR (95%CI):<br>Normal: ref<br>Overweight: 2.4 (1.3 to 3.6)<br>Obese: 2.9 (1.7 to 4.1)	✓		L	L	L	L	L	M
Reijman (2007) [Prospective cohort][309]	KL grade increase knee / hip, OR (95% CI) [adj]<br>≤25 BMI: ref<br>>25-27.5: 1 (0.5, 2.0) / 1.1 (0.8, 1.6)<br>>27.5: 2.1 (1.2, 3.7) / 1.3 (0.9, 1.8)	Knee: ✓<br>Hip: ×		L	L	L	L	M	M
Cooper (2000) [Prospective cohort][316]	Radiographic progression, 1+ grade / 2+ grade, OR (95% CI)<br>Low (BMI <22.7): ref<br>Middle (BMI 22.7-25.4): 2.3 (0.8, 6.4) / 1.8 (0.4, 8.2)<br>Highest (BMI >25.4): 2.6 (1.0, 6.8) / 1.3 (0.3, 5.0)	✓		L	M	L	L	L	L
Harris (1994) [Prospective cohort][317]	Radiographic knee progression, mean BMI<br>minor progression: mean BMI 25.8<br>severe progression: mean BMI 28.0	×		M	M	L	M	H	H
Ledingham (1993) [Prospective cohort][318]	BMI did not predict radiographic progression	×		M	L	L	L	M	H
Ahn (2016) [Retrospective cohort][321]	OA progression, OR (95% CI)<br>OR per unit BMI: 0.958 (0.760, 1.209) unvariable<br>[not included in multivariable analysis]	×		H	M	L	L	H	H

Attr. = attrition, BMI = body mass index, BL = baseline, CI = confidence interval, Conf. = confounding, KL = Kellgren-Laurence, L = low risk of bias, M = moderate risk of bias, OR = odds ratio, Outc. Meas = outcome measurement, Prog. Meas. = prognostic factor measurement, Pros = prospective, Rand. Seq. = random sequence generation, SD = standard deviation, SE = standard error, Stats. = statistical analysis, Study Pop. = study population, VAS = visual analogue scale, WOMAC = Western Ontario and McMaster Universities Osteoarthritis Index

Supplementary table 103 – Joint-space narrowing outcomes from weight-loss interventions in OA

Table – Joint space narrowing (OA), results and quality assessment

Study (date) [study type]	Standardised result, SMD (95% CI) unless otherwise stated	Natural result	Supports intervention	AMSTAR2 quality	Rand. Seq.	Alloc. Conc.	Blind. Part.	Blind. Asses.
Hunter (2015) [RCT][256]	Diet + exercise vs diet, change BL-18 months<br>SMD 0.01 (-0.22, 0.23)<br>Diet + exercise vs exercise, change BL-18 months<br>SMD -0.06 (-0.28, 0.17)	Joint space width, change BL-18 months, mean (SD ‡)<br>Diet: -0.28 (1.6)<br>Exercise: -0.18 (1.6)<br>Diet + exercise: -0.27 (1.6)	×		L	H/UC	H/UC	H/UC

‡ SD calculated from 95% CI

Alloc. Conc. = allocation concealment, AMSTAR2 = Assessing the Methodological Quality of Systematic Reviews, BL = baseline, Blind. Asses. = Blinded assessors, Blind. Part. = blinded participants, CI = confidence interval, H/UC = high / unclear risk of bias, L = low risk of bias, OA = osteoarthritis, Rand. Seq. = random sequence generation, RCT = randomised controlled trial, SD = standard deviation, SMD = Standardised mean difference

Supplementary table 104 – Joint-space narrowing outcomes from observational studies in OA

Table – Joint space narrowing (OA), results and quality assessment of observational studies

Study (date) [study type]	Results	Weight associated with outcome	Study Pop.	Attr.	Prog. Meas.	Outc. Meas.	Conf.	Stats.
Karsdal (2015) [Prospective cohort][288]	Joint space width, change baseline-2 years, Spearman's rho<br>BMI: rho -0.03 p=0.39	✗	L	L	L	M	L	M
Bartlett (2011) [Prospective cohort][297]	Mean BL BMI across the different trajectories of joint space width over two years, mean (SD)<br>high flat: 28.8<br>moderate flat: 29.3<br>moderate low flat: 29.1<br>low flat: 29.7<br>minimal decline: 31.4*<br>moderate decline: 31.1 *<br>greatest decline: 27.9<br>* sig higher than moderate flat	✓	L	M	L	L	M	M
Le Graverand (2009) [Prospective cohort][305]	Joint space width at 12 months, correlation coefficient, (95% CI)<br>BL BMI: -0.148 (-0.388, 0.109)	✗	L	L	L	L	H	H
Botha-Scheepers (2008) [Prospective cohort][306]	Joint space narrowing progression over 2 years, OR (95% CI) [adjusted]<br>BL BMI<30 : ref<br>BL BMI≥30 : 2.3 (0.9 - 5.8)	✓	L	L	L	L	M	M
Reijman (2007) [Prospective cohort][309]	≥1mm JSN knee / hip change, OR (95% CI) [adj]<br>≤25 BMI: ref<br>>25-27.5: 1.2 (0.6, 2.4) / 0.9 (0.6, 1.3)<br>>27.5: 1.4 (0.8, 2.6) / 0.9 (0.6, 1.3)<br>>1.5mm JSN knee / hip change, OR (95% CI) [adjusted]<br>≤25 BMI: ref<br>>25-27.5: 2.3 (0.7, 7.7) / 1.5 (0.6, 3.8)<br>>27.5: 3.2 (1.1, 9.7) / 1.5 (0.6, 3.7)	Knee: ✓<br>Hip: ✗	L	L	L	L	M	M
Wolfe (2002) [Prospective cohort][314]	Max radiographic narrowing, HR (95% CI) [adjusted]<br>BMI continuous: 1.03 (1.00 to 1.06)<br>2nd vs 1st tertile BMI: 1.21 (0.70, 2.08)<br>3rd vs 1st tertile BMI: 1.65 (1.00, 2.71)	✓	L	M	L	L	L	L

Attr. = attrition, BL = baseline, BMI = body mass index, CI = confidence interval, Conf. = confounding, HR = hazard ratio, L = low risk of bias, M = moderate risk of bias, OR = odds ratio, Outc. Meas = outcome measurement, Prog. Meas. = prognostic factor measurement, Pros = prospective, Rand. Seq. = random sequence generation, SD = standard deviation, Stats. = statistical analysis, Study Pop. = study population,

Supplementary table 105 – Bone mineral density outcomes from weight-loss interventions in OA

Table – Bone mineral density (OA), results and quality assessment

Study (date) [study type]	Standardised result, SMD (95% CI) unless otherwise stated	Natural result	Supports intervention	AMSTAR2 quality	Rand. Seq.	Alloc. Conc.	Blind. Part.	Blind. Asses.
Beavers (2014) [RCT][258]	<u>Diet + exercise vs diet, change BL-18 months</u><br>Hip: SMD 0.15 (-0.14, 0.44)<br>Neck: SMD 0.03 (-0.25, 0.32)<br>Spine: SMD -0.12 (-0.41, 0.16)<br><u>Diet + exercise vs exercise, change BL-18 months</u><br>Hip: SMD -0.76 (-1.05, -0.47)<br>Neck: SMD -0.43 (-0.71, -0.15)<br>Spine: SMD -0.17 (-0.45, 0.11)	<u>BMD hip, change bl-18 months, mean (SD †)</u><br>Diet: -24.0 (30.4)<br>Exercise: -2.1 (10.4)<br>Diet + exercise: -19.4 (30.3)<br><u>BMD neck, change bl-18 months, mean (SD †)</u><br>Diet: -15.3 (27.5)<br>Exercise: -2.6 (27.6)<br>Diet + exercise: -14.4 (27.4)<br><u>BMD spine, change bl-18 months, mean (SD †)</u><br>Diet: 3.5 (35.9)<br>Exercise: 5.2 (36.1)<br>Diet + exercise: -0.9 (35.9)	✓		L	H/UC	H/UC	L

† SD calculated from 95% CI

Alloc. Conc. = allocation concealment, AMSTAR2 = Assessing the Methodological Quality of Systematic Reviews, BL = baseline, BMD = bone mineral density, Blind. Asses. = Blinded assessors, Blind. Part. = blinded participants, CI = confidence interval, H/UC = high / unclear risk of bias, L = low risk of bias, OA = osteoarthritis, Rand. Seq. = random sequence generation, SD = standard deviation, SMD = Standardised mean difference

Supplementary table 106 – Cartilage loss outcomes from weight-loss interventions in OA

Table – Cartilage loss (OA), results and quality assessment

Study (date) [study type]	Standardised result, SMD (95% CI) unless otherwise stated	Natural result	Supports intervention	AMSTAR2 quality	Rand. Seq.	Alloc. Conc.	Blind. Part.	Blind. Asses.
Hunter (2015) [RCT][256]	Diet + exercise vs diet, change BL-18 months<br>SMD -0.04 (-0.26, 0.19)<br>Diet + exercise vs exercise, change BL-18 months<br>SMD -0.13 (-0.35, 0.10)	Cartilage volume , change bl-18 months, mean (SD †)<br>Diet -73.1 (479.6)<br>Exercise: -32.3 (451.9)<br>Diet + exercise: -89.9 (463.6)	×		L	H/UC	H/UC	H/UC
Henriksen (2014) [RCT][259]	Diet vs control, change BL-week 68<br>SMD -0.18 (-0.54, 0.17)<br>Exercise vs control, change BL-week 68<br>SMD 0.03 (-0.32, 0.37)	Medial tibiofemoral cartilage loss, change BL-week 68, mean (SD ‡)<br>Diet: -0.13 (0.38)<br>Exercise: -0.05 (0.38)<br>Control: -0.06 (0.39)	×		L	L	H/UC	L

† SD calculated from standard error

‡ SD calculated from 95% CI

Alloc. Conc. = allocation concealment, AMSTAR2 = Assessing the Methodological Quality of Systematic Reviews, BL = baseline, Blind. Asses. = Blinded assessors, Blind. Part. = blinded participants, CI = confidence interval, H/UC = high / unclear risk of bias, L = low risk of bias, OA = osteoarthritis, Rand. Seq. = random sequence generation, SD = standard deviation, SMD = Standardised mean difference

Supplementary table 107 – Cartilage loss outcomes from observational studies in OA

Table – Cartilage loss (OA), results and quality assessment of observational studies

Study (date) [study type]	Results	Weight associated with outcome	Study Pop.	Attr.	Prog. Meas.	Outc. Meas.	Conf.	Stats.
Moyer (2017) [Prospective cohort][283]	Cartilage loss, cMFTC / cLFTC, reg coef (95% CI) per unit BMI<br>neutral (+/- 0–2 degrees): -1.6 (-4.8, 1.6) / 1.3 (-1.7, 4.3)<br>minor malalignment (+/- 2-3.5 deg): -7.7 (-13.2, -2.2) / -2.2 (-6.6, 2.3)<br>definite malalignment (+/- ≥3.5 deg): -4.6 (-10.9, 1.7) / 1.7 (-3.1, 6.5)	×	L	M	L	L	L	M
Eckstein (2009) [Prospective cohort][304]	[1) BMI <25, 2) BMI 25-30, 3) BMI 30-35, 4) BMI ≥35]<br>Medial tibia, bl-1 year mean change %<br>1) -0.46, 2) -0.22, 3) -1.00, 4) -0.08<br>Weight-bearing medial femoral condyle, bl-1 year mean change %<br>1) 0.10, 2) -1.38, 3) -3.28, 4) -1.56<br>Medial femoro-tibial compartment, bl-1 year mean change %<br>1) -0.17, 2) -0.81, 3) -2.14, 4) -0.84<br>Lateral tibia, bl-1 year mean change %<br>1) -0.43, 2) -0.38, 3) -0.97, 4) -0.96<br>Weight-bearing lateral femoral condyle, bl-1 year mean change %<br>1) -0.02, 2) 0.17, 3) -0.09, 4) 0.48<br>Lateral femoro-tibial compartment, bl-1 year mean change %<br>1) -0.23, -0.12, -0.53, -0.25	×	L	L	L	L	M	M
Davies-Tuck (2008) [Prospective cohort][307]	Multivariable regression (95%CI):<br>Medial tibiofemoral cartilage defect:<br>BMI : 0.005 (-0.02 to 0.03)<br>Lateral tibiofemoral cartilage defects:<br>BMI : 0.004 (-0.02 to 0.03)	×	M	L	L	L	L	M
Pelletier (2007) [Prospective cohort][308]	medial central femur cartilage volume loss at 24 months, regression coefficient (SE)<br>BL BMI -0.19 (0.09) p=0.03 [adj]<br>medial central subregion cartilage volume loss at 24 months, regression coefficient (SE)<br>BL BMI -0.15 (0.09) p=0.09	✓	L	L	L	L	H	H

Attr. = attrition, BL = baseline, BMI = body mass index, CI = confidence interval, Conf. = confounding, cLFTC =central weight-bearing lateral femorotibial compartment, cMFTC = central weight-bearing medial femorotibial compartment L = low risk of bias, M = moderate risk of bias, Outc. Meas = outcome measurement, Prog. Meas. = prognostic factor measurement, Pros = prospective, Rand. Seq. = random sequence generation, SD = standard deviation, SE = standard error, SMD = Standardised mean difference, Stats. = statistical analysis, Study Pop. = study population

Table – Cartilage loss (OA), results and quality assessment of observational studies

Study (date) [study type]	Results	Weight associated with outcome	Study Pop.	Attr.	Prog. Meas.	Outc. Meas.	Conf.	Stats.
Raynauld (2006) [Prospective cohort][310]	Cartilage loss cluster, mean (SD) BL BMI<br>Slow cartilage loss: 29.6 (4.3)<br>Intermediate cartilage loss: 31.0 (4.3)<br>Fast cartilage loss: 32.6 (2.7) p=0.06	✓	M	L	L	L	H	H
Wluka (2006) [Prospective cohort][311]	Cartilage volume over 4.5 years, regression coefficient (95% CI)<br>Medial cartilage: BMI = 1.1 (-2.3 to 4.6) [multivariable]<br>lateral cartilage: BMI = 0.2 (-3.4, 3.9) [multivariable]	×	L	M	L	L	L	M
Cicuttini (2002) [Prospective cohort][313]	Cartilage lose (patella), regression coefficient (95% CI)<br>Unadjusted: -0.00645 per unit BMI (-0.002, 0.001)<br>Adjusted: -0.0019 per unit BMI (-0.004, 0.000)	✓	L	M	L	L	L	M
Schouten (1992) [Prospective cohort][319]	Cartilage loss, OR (95% CI) [adj]<br>BMI<24.35: ref<br>24.35-25.96: 1.77 (0.48, 6.50)<br>25.97-27.73: 5.28 (1.54, 18.1)<br>>27.73: 11.1 (3.28, 37.3)	✓	L	M	L	L	M	M

Attr. = attrition, BL = baseline, BMI = body mass index, CI = confidence interval, Conf. = confounding, L = low risk of bias, M = moderate risk of bias, OR = odds ratio, Outc. Meas = outcome measurement, Prog. Meas. = prognostic factor measurement, Pros = prospective, Rand. Seq. = random sequence generation, SD = standard deviation, Stats. = statistical analysis, Study Pop. = study population

Supplementary table 108 – Bone marrow lesion outcomes from weight-loss interventions in OA

Table – Bone marrow lesions (OA), results and quality assessment

Study (date) [study type]	Standardised result, SMD (95% CI) unless otherwise stated	Natural result	Supports intervention	AMSTAR2 quality	Rand. Seq.	Alloc. Conc.	Blind. Part.	Blind. Asses.
Henriksen (2014) [RCT][259]	Diet vs control, change BL-week 68<br>SMD 0.24 (-0.11, 0.60)<br>Exercise vs control, change BL-week 68<br>SMD 0.32 (-0.03, 0.67)	Medial tibiofemoral BML, change BL- week 68, mean (SD ‡)<br>Diet: -0.02 (0.49)<br>Exercise: 0.02 (0.51)<br>Control: -0.14 (0.50)	×		L	L	H/UC	L

‡ SD calculated from 95% CI

Alloc. Conc. = allocation concealment, AMSTAR2 = Assessing the Methodological Quality of Systematic Reviews, BL = baseline, Blind. Asses. = Blinded assessors, Blind. Part. = blinded participants, BML = bone marrow lesions, CI = confidence interval, H/UC = high / unclear risk of bias, L = low risk of bias, OA = osteoarthritis, Rand. Seq. = random sequence generation, SMD = Standardised mean difference

Supplementary table 109 – Bone marrow lesion outcomes from observational studies in OA

Table – Bone marrow lesions (OA), results and quality assessment of observational studies

Study (date) [study type]	Results	Weight associated with outcome	Study Pop.	Attr.	Prog. Meas.	Outc. Meas.	Conf.	Stats.
Gudbergsen (2013) [Prospective cohort][291]	Bone marrow lesion response, OR (95% CI)<br>weight loss >10% vs <10%: 1.95 (0.70, 5.45) [unadjusted]<br>weight loss >10% vs <10%: 1.86 (0.66, 5.26) [adjusted]	×	L	L	L	L	L	M

Attr. = attrition, CI = confidence interval, Conf. = confounding, L = low risk of bias, M = moderate risk of bias, OR = odds ratio, Outc. Meas = outcome measurement, Prog. Meas. = prognostic factor measurement, Pros = prospective, Rand. Seq. = random sequence generation, Stats. = statistical analysis, Study Pop. = study population

Supplementary table 110 – CRP outcomes from weight-loss interventions in OA

Table – CRP (OA), results and quality assessment

Study (date) [study type]	Standardised result, SMD (95% CI) unless otherwise stated	Natural result	Supports intervention	AMSTAR2 quality	Rand. Seq.	Alloc. Conc.	Blind. Part.	Blind. Asses.
Miller (2008) [RCT][266]	Diet vs control at 6 months<br>SMD 0.00 (-0.48, 0.48)	CRP, BL / 6 months, mean (SD †)<br>Diet 0.58 (0.50) / 0.62 (0.56)<br>Control: 0.72 (0.48) / 0.62 (0.90)	×		H/UC	H/UC	H/UC	H/UC
Bartels (2014) [Single arm int.][275]		CRP, mean change from BL-16 weeks, unadjusted mean (SD) / adjusted mean (95% CI)<br>-1.3 (5.7) / -1.3 (-2.1, -0.01)	✓					

† SD calculated from standard error

Alloc. Conc. = allocation concealment, AMSTAR2 = Assessing the Methodological Quality of Systematic Reviews, BL = baseline, Blind. Asses. = Blinded assessors, Blind. Part. = blinded participants, CI = confidence interval, CRP = C-reative protin, H/UC = high / unclear risk of bias, L = low risk of bias, OA = osteoarthritis, Rand. Seq. = random sequence generation, RCT = randomised controlled trial, SD = standard deviation, SMD = Standardised mean difference

Supplementary table 111 – CRP outcomes from observational studies in OA

Table – CRP (OA), results and quality assessment of observational studies

Study (date) [study type]	Results	Weight associated with outcome	Study Pop.	Attr.	Prog. Meas.	Outc. Meas.	Conf.	Stats.
Beavers (2015) [Prospective cohort][286]	CRP (logged, 18 mth change), regression coefficient (95% CI)<br>BMI: 0.15 (0.11, 0.19)<br>[Adjusted – including randomisation group]	✓	L	M	L	L	L	L

Attr. = attrition, BMI = body mass index, CI = confidence interval, Conf. = confounding, L = low risk of bias, M = moderate risk of bias, Outc. Meas = outcome measurement, Prog. Meas. = prognostic factor measurement, Pros = prospective, Rand. Seq. = random sequence generation, Stats. = statistical analysis, Study Pop. = study population

Supplementary table 112 – Response criteria outcomes from weight-loss interventions in OA

Table – Response criteria (OA), results and quality assessment

Study (date) [study type]	Standardised result, SMD (95% CI) unless otherwise stated	Natural result	Supports intervention	AMSTAR2 quality	Rand. Seq.	Alloc. Conc.	Blind. Part.	Blind. Asses.
Riecke (2010) [RCT][264]		<u>OMERACT-OASRI response, n(%)</u><br>Low calories: 63 (65.6%)<br>Very low calories 59 (61.5%); p=0.55	×		L	L	H/UC	L

Alloc. Conc. = allocation concealment, AMSTAR2 = Assessing the Methodological Quality of Systematic Reviews, Blind. Asses. = Blinded assessors, Blind. Part. = blinded participants, CI = confidence interval, H/UC = high / unclear risk of bias, L = low risk of bias, OA = osteoarthritis, OARSI = Osteoarthritis Research Soceity International, OMERACT = Outcome measures in Rheumatology, Rand. Seq. = random sequence generation, RCT = randomised controlled trial

Supplementary table 113 – Response criteria outcomes from observational studies in OA

Table – Response criteria (OA), results and quality assessment of observational studies

Study (date) [study type]	Results	Weight associated with outcome	Study Pop.	Attr.	Prog. Meas.	Outc. Meas.	Conf.	Stats.
Pelletier (2018) [Prospective cohort][281]	<u>WOMAC responders, mean (SD) BMI</u><br>Responders: 32.09 (6.04)<br>Non-responders: 30.06 (6.50) p=0.163	×	L	L	L	L	H	H
Eymard (2017) [Prospective cohort][282]	<u>OMERACT-OARSI response, OR (95% CI)</u><br>BMI: 0.89 (0.82, 0.95)<br>obesity (y/n): 0.23 (0.10, 0.51)	✓	L	M	L	L	L	M
Bingham (2011) [Prospective cohort][298]	<u>Responders, yes / no, mean (SD) BMI</u><br>Placebo: 31.7 (7.5) / 33.4 (7.3); p=0.164<br>Etoricoxib: 32.5 (7.0) / 33.3 (7.5); p=0.285<br>Celecoxib: 32.0 (7.3) / 32.3 (6.3) ; p=0.768	×	L	M	L	L	H	H

Attr. = attrition, BMI = body mass index, CI = confidence interval, Conf. = confounding, H = high risk of bias, L = low risk of bias, M = moderate risk of bias, OA = osteroarthritis, OARSI = Osteoarthritis Research Soceity International, OMERACT = Outcome measures in Rheumatology, OR = odds ratio, Outc. Meas = outcome measurement, Prog. Meas. = prognostic factor measurement, Pros = prospective, Rand. Seq. = random sequence generation, SD = standard deviation, Stats. = statistical analysis, Study Pop. = study population, WOMAC = Western Ontario and McMaster Universities Osteoarthritis Index

Supplementary table 114 – Walk-test outcomes from weight-loss interventions in OA

Table – Walk-test (OA), results and quality assessment

Study (date) [study type]	Standardised result, SMD (95% CI) unless otherwise stated	Natural result	Supports intervention	AMSTAR2 quality	Rand. Seq.	Alloc. Conc.	Blind. Part.	Blind. Asses.
Christensen (2015) [RCT][255]	Diet vs control, change BL-68 weeks<br>SMD 0.24 (-0.11, 0.59)<br>Exercise vs control, change BL-68 weeks<br>SMD 0.26 (-0.09, 0.61)	6MWT, change BL-68 weeks, mean (SD ‡)<br>Diet: 37.519 (60.3)<br>Exercise: 38.478 (60.3)<br>Control: 22.89 (60.2)	×		L	L	H/UC	L
Messier (2013) [RCT][26]	Diet + exercise vs diet at 18 months<br>SMD 0.37 (0.14, 0.60)<br>Diet + exercise vs exercise at 18 months<br>SMD 0.12 (-0.10, 0.35)	6MWT (m) , BL / 18 months, mean (SD ‡)<br>Diet: 475 (81.8) / 502 (84.9)<br>Exercise: 480 (90.6) / 525 (90.6)<br>Diet + exercise: 467 (88.1) / 537 (103.8)	✓		L	H/UC	H/UC	L
Miller (2006) [RCT][267]	Diet vs control at 6 months<br>SMD 0.48 (0.05, 0.90)	6MWT (m), BL / 6 months, mean (SD †)<br>Diet: 436.5 (86.2) / 510.0 (99.5)<br>Control: 447.8 (97.7) / 459.0 (114.1)	✓		H/UC	H/UC	H/UC	H/UC
Messier (2000) [RCT][269]	Exercise + diet vs exercise<br>SMD 0.78 (-0.06, 1.61)	6MWT (distance in feet), mean (SD †) at 6 months<br>Exercise: 1718 (136.0)<br>Exercise + diet: 1821 (129.8)	×		H/UC	H/UC	H/UC	L
Bespoke meta-analysis including[255;267]	Weight loss intervention vs control<br>SMD 0.31 (0.09, 0.52), I^2 0%		✓					
Paans (2013) [Single arm int.][276]		6MWT BL / 8 months, mean (SD †)<br>433.3 (73.9) / 481.4 (59.7)	✓					

† SD calculated from standard error

‡ SD calculated from 95% CI

6MWT = six minute walk test, Alloc. Conc. = allocation concealment, AMSTAR2 = Assessing the Methodological Quality of Systematic Reviews, BL = baseline, Blind. Asses. = Blinded assessors, Blind. Part. = blinded participants, CI = confidence interval, H/UC = high / unclear risk of bias, L = low risk of bias, m = metres, OA = osteoarthritis, Rand. Seq. = random sequence generation, RCT = randomised controlled trial, SD = standard deviation, SMD = Standardised mean difference

Supplementary table 115 – Mortality outcomes from observational studies in OA

Table – Death (OA), results and quality assessment of observational studies

Study (date) [study type]	Results	Weight associated with outcome	Study Pop.	Attr.	Prog. Meas.	Outc. Meas.	Conf.	Stats.
Shea (2010) [Prospective cohort][303]	<u>Death, Cox Regression analysis (mean weight loss-1.4 vs -4.8kg):</u><br>WL vs NWL : HR 0.5 (0.3, 0.9)<br>Adjusted HR : 0.5 (0.3, 1.0)	✓	L	L	L	L	M	M

Attr. = attrition, CI = confidence interval, Conf. = confounding, HR = hazard ratio, kg = kilograms, L = low risk of bias, M = moderate risk of bias, NWL = no weight loss, Outc. Meas = outcome measurement, Prog. Meas. = prognostic factor measurement, Pros = prospective, Rand. Seq. = random sequence generation, Stats. = statistical analysis, Study Pop. = study population, VAS = visual analogue scale, WL = weight loss

Supplementary table 116 – Description of studies of assessing weight and outcomes in RA

Table – Rheumatoid arthritis, description of reviews

Authors (date)	Review type	Study type included	Exposure detail	Number of studies included	Funders
Liu (2017)[322]	MA	Observational studies, RCTs	Studies investigating the association between obesity and outcomes in RA	16	**Government** (Canadian Institutes for Health Research)
Lupoli (2016)[323]	MA	Observational studies	Studies investigating the association between obesity and minimal disease activity in RA	17	**Government** (Italian Ministry of Health)
Baghdadi (2015)[324]	MA	Observational studies	Studies investigating the association between obesity and cardiovascular morbidity in RA	10	**No funding**

MA = meta-analysis, RA = rheumatoid arthritis, RCT = randomised controlled trial

Table – Rheumatoid arthritis, description of included studies

Author (date) [country]	Study design	Inclusion criteria	Exposure detail	N	Age, mean (SD) years	N (%) female	Funders
Baker (2019) [USA][325]	Pros. Cohort	National Data Bank for Rheumatic Diseases, aged >40 years.<br>Exclusions: BMI<14	BMI at age 30 and at enrolment:<br><18.5<br>18.5-25<br>25-30<br>>30	12268	59.9 (10.5)	80%	**Government** (Veterans Affairs)
Hirose (2019) [Japan][326]	Pros. Cohort	2010 ACR/EULAR RA criteria, aged >20 years	BMI <18	388	With MAC-PD:<br>71.4 (6.3)<br>without MAC-PD:<br>64.3 (13.2)	With MAC-PD: 78.6%<br>Without MAC-PAD: 80.8% F	**No funding**
Lechtenboehmer (2019) [Switzerland][327]	Pros. Cohort	Swiss Clinical Quality Management in Rheumatic Diseases (SCQM) registry – patients had two radiographs	BMI – continuous	Progression: 680<br>No progression: 519	Progression: 59.4 (9.8)<br>No progression: 60.5 (10.5)	Progression: 79%<br>No progression: 75%	**Industry** (Abbvie, Celgene, iQone, Lilly, MSD, Novartis, Pfizer, Roche, Samsung Bioepis, Sandoz, Sanofi, UCB)
England (2018) [USA][328]	Pros. Cohort	Veterans Affairs cohort – 1987 ACR RA criteria, aged >18 years, US veterans	BMI categories:<br>Underweight: <20<br>Normal: 20-25<br>Overweight: >25-30<br>Obese: >30	1600	63.4 (11.0)	8.8%	**Government** (Veterans Affairs, NIH)
Nikiphorou (2018) [UK][329]	Pros. Cohort	ERAN / ERAS studies	BMI categories:<br>Underweight: <18.5<br>Normal / overweight: 18.5-29.99<br>Obese: ≥30	2701	56 (14)	1812 (67)	**Charity** (Arthritis Research Campaign), **Industry** (BUPA), **Government** (NIHR)
Rydell (2018) [Sweden][330]	Pros. Cohort	1987 ACR RA criteria, symptom duration ≤12 months	BMI categories:<br>Normal: 18.5-24.99<br>Overweight: 25-39.99<br>Obese: ≥30	162	Median (IQR)<br>62 (52-70)	114 (70.4)	**University** (Lund), **Professional body** (Swedish Rheumatism Association), **Government** (Swedish Research Council), **Charity** (Foundation for Assistance to Disabled People in Skåne)

ACR = American College of Rheumatology, BMI = body mass index, EULAR = European League Against Rheumatism, IQR = interquartile range, MAC-PD = mycobacterium avium complex pulmonary disease, N = number, NIH = National Institutes of Health, NIHR = National Institute for Health Research, pros. = prospective, SD = standard deviation, UK = United Kingdom, United States of America

Table – Rheumatoid arthritis, description of included studies

Author (date) [country]	Study design	Inclusion criteria	Exposure detail	N	Age, mean (SD) years	N (%) female	Funders
Schulman (2018) [Canada][331]	Pros. Cohort	CATCH cohort - Fulfilment 1987 or 2010 criteria for RA, aged ≥18 years, early IA	BMI categories:<br>Healthy: 18.5-24.9<br>Overweight: 25-29.9<br>Obese: >30	Healthy: 315<br>Overweight: 343<br>Obese: 324	Healthy: 50 (17)<br>Overweight: 55 (15)<br>Obese: 54 (13)	Healthy: 260 (83%)<br>Overweight: 214 (63%)<br>Obese: 235 (73%)	**Industry** (Amgen and Pfizer Canada, Hoffmann-LaRoche, UCB Canada, Bristol-Myers Squibb Canada, AbbVie, Janssen Biotech,Medexus, Eli Lilly Canada, and Sanofi Canada)
Smolen (2018) [Multinational][332]	Pros. Cohort	Reanalysis of PRESERVE trial – Active RA with moderate disease activity (DAS28: 3.2-5.1)	BMI categories:<br><18.5<br>18.5-25<br>25-30<br>>30	834	48.4 (11.9)	694 (83.2)	**Industry** (Pfizer)
Sparks (2018) [USA][333]	Pros. Cohort	1987 ACR RA criteria	Weight change – before and after RA diagnosis<br>Stable: <10pounds;<br>Mild loss change weight <-10 to -20 pounds<br>Moderate loss <-20 to -30 pounds<br>Severe loss <-30 pounds.<br>For weight gain reverse	902	Severe Loss: 62.0 (8.9)<br>Moderate loss: 59.3 (12.9)<br>Mild loss: 58.1 (9.1)<br>stable: 55.8 (9.8)<br>Mild gain: 53.4 (9.4)<br>Moderate gain: 52.3 (8.3)<br>Severe gain: 50.0 (6.8)"	100%	**Charity** (Rheumatology Research Foundation), **Government** (NIH)

ACR = American College of Rheumatology, BMI = body mass index, DAS28 = Disease activity score 28, N = number, NIH = National Institutes of Health, pros. = prospective, SD = standard deviation, United States of America

Table – Rheumatoid arthritis, description of included studies

Author (date) [country]	Study design	Inclusion criteria	Exposure detail	N	Age, mean (SD) years	N (%) female	Funders
van der Heijde (2018) [Multinational] [334]	Pros. Cohort	Reanalysis of RA-BEGIN trial – aged ≥18 years, active RA, no / limited treatment with conventional DMARDs and no biologic DMARDs randomised to either methotrexate, baracitinib or methotrexate + baracitinib<br>Group A = sustained DAS28 ≤3.2<br>Group B = DAS28 >3.2	BMI – continuous	545	Group A:<br>MTX: 52 (14)<br>bari: 52 (13)<br>bari + MTX: 46 (14)<br>Group B:<br>MTX: 50 (13)<br>bari: 50 (13)<br>bari + MTX: 51 (13)"	Group A:<br>MTX: 31 (68.9)<br>bari: 49 (73.1)<br>bari + MTX: 71 (71.0)<br>Group B:<br>MTX: 117 (70.9)<br>bari: 72 (78.3)<br>bari + MTX: 85 (73.9)"	**RCT funded by Eli Lilly**
Bird (2017) [Australia][335]	Pros. Cohort	PREDICT study – symptom duration <12 months, aged >18 years	Weight in kilograms	1017	60.4 (14.7)	708 (69.6)	**Industry** (Roche)
D'Agostino (2017) [Multinational] [336]	Pros. Cohort	Reanalysis of ACQUIRE RCT (abatacept) – active RA, inadequate response to methotrexate	BMI categories:<br>Underweight/normal: <25<br>Overweight: 25-30<br>Obese: ≥30	1456	Underweight / normal: 57.5 (14.4)<br>Overweight: 51.3 (12.2)<br>Obese: 51.6 (11.2)	Underweight/ normal: 83.1%<br>Overweight: 78.9%<br>Obese: 85.7%	**Industry** (Bristol Myers Squibb)
George (2017) [USA][337]	Pros. Cohort	Reanalysis of RCT – those who had MRIs scores, aged ≥18 years, 1987 ACR RA	BMI – dichotomised as obese (≥30) or not	470	BMI <20:<br>44 (14)<br>20-25:<br>47 (12)<br>25-30:<br>51 (11)<br>≥30: 52 (11)	BMI <20:<br>43 (84%)<br>20-25:<br>136 (83%)<br>25-30:<br>126 (83%)<br>≥30: 87 (84%)	**Charity** (Rheumatology Research Foundation), **Government** (Veterans Affairs, NIHR)

ACR = American College of Rheumatology, BMI = body mass index, DAS28 = Disease activity score 28, DMARD = disease modifying anti-rheumatic drug, MRI = magnetic resonance imaging, N = number, NIHR = National Institute for Health Research, pros. = prospective, SD = standard deviation, United States of America

Table – Rheumatoid arthritis, description of included studies

Author (date) [country]	Study design	Inclusion criteria	Exposure detail	N	Age, mean (SD) years	N (%) female	Funders
Iannone (2017) [Multinational] [338]	Pros. Cohort	PANABA collaboration - 10 national registers: Czech Republic, Denmark, France, Italy, Norway, Portugal, Spain, Sweden, Switzerland and Canada<br>All taking abatcept	BMI categories:<br>Underweight/normal: <25<br>Overweight: 25-30<br>Obese: 30-35<br>Severely obese: ≥35	Under / normal weight: 1014<br>Overweight: 621<br>Obese: 267<br>severely obese: 113	Under / normal weight: 55.4 (14.1)<br>Overweight: 57.9 (12.6)<br>Obese: 58.4 (11.0)<br>Severely obese: 54.5 (10.8)	Under/normal weight: 877 (86.5)<br>Overweight: 442 (71.2)<br>Obese: 213 (79.8)<br>Severely obese: 96 (85.0)	**Industry** (Bristol Myers Squibb)
Joo (2017) [S. Korea][339]	Pros. Cohort	Hanyang Bae RA cohort, >19 years, 1987 RA, symptom duration <2 years	BMI – continuous	374	48.7 (12.0)	84.2%	**Government** (Ministry for Health & Welfare)
Levitsky (2017) [Sweden][340]	Pros. Cohort	SWEFOT RCT reanalysis (Methotrexate vs triple therapy)	BMI categories:<br>Normal: <25<br>Overweight: 25-29.9<br>Obese: >30	154	Median (IQR)<br>56 (44-63)	111 (72.1)	**SWEFOT trial funded by** (Swedish Rheumatism Association, Stockholm County, and Schering-Plough/Merck Sharp and Dohme)
Mariette (2017) [Canada][341]	Pros. Cohort	ACTION study – starting abatacept, biologic naïve, aged ≥18 years, 1987 ACR RA criteria	BMI categories:<br>Underweight / Normal: <25<br>Overweight: 25-29.9<br>Obese: >30	672	Mean (95% CI)<br>Under / Normal: 58.2 (56.5, 59.8)<br>Overweight: 61.8 (60.0, 63.4)<br>Obese: 60.3 (58.6, 62.0)	Under / normal: 74.2%<br>Overweight: 66.1%<br>Obese: 60.3%"	**Industry** (Bristol Myers Squibb)
Miwa (2017) [Japan][342]	Pros. Cohort	RA patients treated with non-TNFi biologics	BMI – continuous	97	Remission at FU:<br>59 (50, 68)<br>No remission:<br>70 (62, 74) §	Remission: 83%<br>No remission: 80%	**Industry** (Astellas Pharma, Mitsubishi Tanabe Pharma, AbbVie, Pfizer Japan, Chugai Pharmaceutical)

ACR = American College of Rheumatology, BMI = body mass index, FU = follow-upIQR = interquartile range, N = number, pros. = prospective, RCT = randomised controlled trial, SD = standard deviation, TNF = tumour necrosis factor inhibitor

Table – Rheumatoid arthritis, description of included studies

Author (date) [country]	Study design	Inclusion criteria	Exposure detail	N	Age, mean (SD) years	N (%) female	Funders
Ramirez (2017) [Spain][343]	Pros. Cohort	RA in clinical remission (DAS28<2.6) for >6 months	BMI – continuous	42	Median (IQR) 54.5 (45.0, 61.0)	32 (76.2)	**Government** (Spain and Ministeriode Economía y Competitividad)
Feldthusen (2016) [Sweden][344]	Pros. Cohort	ICD 10 code for RA, aged 20-65 years, symptom duration >3 years, stable medication >3 months<br>Exclusions: Other severe somatic or psychiatric diseases or not having the capacity to communicate effectively in Swedish	BMI – continuous	65	53.7 (9.9)	48 (73.8)	**University** (Gothenburg Centre for Person-Centred Care), **Government** (Swedish Research Council), **Hospital** (Sahlgrenska University Hospital)
Gardette (2016) [France][345]	Pros. Cohort	2010 ACR/EULAR RA criteria, active RA, all treated with abatacept	BMI – continuous	141	BMI <25: 54.0 (14.6)<br>BMI 25-30: 54.6 (13.2)<br>BMI >30: 53.5 (10.5)	BMI<25: 54 (84.3)<br>BMI 25-30: 32 (84.2)<br>BMI >30: 30 (77.0)	**Not reported** – authors declared no conflict of interest
Gardette (2016) [France][346]	Pros. Cohort	2010 ACR/EULAR RA criteria, active RA, all treated with tocilizumab	BMI – continuous	115	BMI<25 53.0 (12.5)<br>BMI 25-30: 52.9 (8.6)<br>BMI >30: 51.6 (12.7)	BMI <25: 45 (84.9)<br>BMI 25-30: 30 (81.1)<br>BMI >30: 22 (88.0)	**Not reported** – authors declared no conflict of interest
McWilliams (2016) [UK][347]	Pros. Cohort	BSRBR-RA study – first time biologic users or non-biologic cohort, 1987 ACR RA	BMI categories:<br><25<br>25-30<br>≥30	TNFi – 11995<br>Non-TNFi - 3632	TNFi – 56 (12)<br>Non-TNFi – 60 (12)	TNFi – 76%<br>Non-TNFi – 73%	**Industry** (Pfizer)
Tantayakom (2016) [Thailand][348]	Pros. Cohort	RA according to 2010 ACR/EULAR criteria<br>Exclusions: diagnosed with another rheumatic or autoimmune condition	BMI – continuous	267	59 (11.1)	236 (88.4)	**University** (Mahidol University)

ACR = American College of Rheumatology, BMI = body mass index, BSRBR = British Soceity for Rheumatology Biologics Register, DAS28 = Disease activity score 28, EULAR = European League Against Rheumatism, ICD = International Classification of Diseases, IQR = interquartile range, N = number, pros. = prospective, SD = standard deviation, TNF = tumour necrosis factor inhibitor

Table – Rheumatoid arthritis, description of included studies

Author (date) [country]	Study design	Inclusion criteria	Exposure detail	N	Age, mean (SD) years	N (%) female	Funders
Baker (2015) [USA][349]	Pros. Cohort	Veterans Affairs cohort – 1987 ACR RA, aged >18 years	BMI categories:<br><20<br>20-25<br>>25,30,<br>>30<br>Rate of change of BMI:<br><2 per year<br>2-3 per year<br>>3 per year	1674	63.5 (11.1)	9%	**Government** (Veterans affairs, NIH), **Charity** (Rheumatology Research Foundation)
Iannone (2015) [Italy][350]	Pros. Cohort	1987 ACR RA criteria, active disease about to start a biologic (adalimumab, certolizumab, etanercept, golimumab, infliximab, abatacept, tocilizumab, rituximab)	BMI categories:<br>Normal: <25<br>Overweight: 25-30<br>Obese: >30	292	Normal:<br>53.5 (20)<br>Overweight:<br>61 (14)<br>Obese: 60.5 (14)	Normal:<br>105 (89.7)<br>Overweight:<br>89 (81.6)<br>Obese:<br>55 (83.3)	**No funding**
Pers (2015) [France][351]	Pros. Cohort	Tocilizumab for RA, 2010 ACR/EULAR RA criteria	BMI categories:<br>Normal: <25<br>Overweight: 25-30<br>Obese: >30	222	Median (IQR)<br>56 (47, 66)	82.4%	**No reported**
Kim (2014) [S.Korea][352]	Pros. Cohort	starting anti-TNF, 1987 ACR RA criteria	BMI categories:<br>BMI <22	222	51.9 (12.6)	192 (86.5)	**Not reported** – authors declared no conflicts of interest
Ochi (2014) [Japan][353]	Pros. Cohort	IORRA study – RA diagnosis	BMI - continuous	9987	w/ facture:<br>61.5 (11.1)<br>no fracture:<br>55.7 (13.5)	w/ fracture:<br>93.2%<br>no fracture:<br>81.9%	**Industry** (34 pharmaceutical companies), **Government** (Japan Society for the Promotion of Science), **Charity** (Nakatomi Foundation, Orthopaedics and Traumatology Foundation), **Professional body** (Japan Osteoporosis Society)

ACR = American College of Rheumatology, BMI = body mass index, EULAR = European League Against Rheumatism, IORRA = Institute of Rheumatology Rheumatoid Arthritis, IQR = interquartile range, N = number, pros. = prospective, Retro. = retrospective, SD = standard deviation, TNF = tumour necrosis factor inhibitor, United States of America

Table – Rheumatoid arthritis, description of included studies

Author (date) [country]	Study design	Inclusion criteria	Exposure detail	N	Age, mean (SD) years	N (%) female	Funders
Sandberg (2014) [Sweden][354]	Pros. Cohort	EIRA study	BMI categories:<br>Normal <25<br>Overweight: 25-30<br>Obese: ≥30	495	<40 years: 17%<br>40-50: 15%<br>50-60: 27%<br>60-70: 41%	353 (71.3)	**Government** (Swedish Medical Research Council, Swedish Research Council for Health, Vinnova Working Life and Welfare), **Charity** (AFA foundation, King Gustaf V's 80-year foundation, the Swedish Rheumatic Foundation, Swedish Foundation for Strategic Research)
Ajeganova (2013) [Sweden][355]	Pros. Cohort	BARFOT study – 1987 ACR RA, symptom duration ≤12 months	BMI categories:<br>1) ≤20<br>2) >20 to <25<br>3) ≥25 to <30<br>4) ≥30	1596<br>1) 89<br>2) 775<br>3) 526<br>4) 206	55.6 (14.6)	67.8%	**Professional body** (Swedish Rheumatism Association), **Charity** (King Gustaf V's 80-Year Fund), **Government** (Stockholm County Council)
Gremese (2013) [Italy][356]	Pros. Cohort	GISEA study – active disease despite methotrexate treatment, 1987 ACR criteria, starting TNFi	BMI – continuous and categories:<br>BMI <20<br>BMI 20-30<br>BMI >30	641	52.1 (13.5)	521 (81.3)	**Industry** (CD-Pharma)
Kanecki (2013) [Poland][357]	Pros. Cohort	RA patients	BMI - continuous	51	62.5 (12.6)	42 (82.4)	**Not reported** – authors declared no conflict of interest
Dirven (2012) [The Netherlands][358]	Pros. Cohort	Diagnosis of RA	BMI - continuous	783	61 (13)	545 (32.2)	**Not reported**

ACR = American College of Rheumatology, BARFOT = Better Anti-Rheumatic Farmacotherapy, BMI = body mass index, GISEA = Gruppo Italiano di Studio sulle Early Arthritis, N = number, pros. = prospective, SD = standard deviation, TNF = tumour necrosis factor inhibitor

Table – Rheumatoid arthritis, description of included studies

Author (date) [country]	Study design	Inclusion criteria	Exposure detail	N	Age, mean (SD) years	N (%) female	Funders
Wevers-de Boer (2012) [The Netherlands][359]	Pros. Cohort	Reanalysis of IMPROVED RCT – 1987 ACR RA criteria, <2 years symptom duration, or undifferentiated arthritis according to rheumatologist, DAS28 ≥1.6 Exclusions: previous therapy with disease modifying antirheumatic drugs or corticosteroids, pregnancy or pregnancy wish during the study, malignancy within the last 5 years, bone marrow hypoplasia, elevated liver enzyme levels (aspartate transaminase (AST) and/or alanine transaminase (ALT)>3 times normal value), serum creatinine level >150 umol/l or estimated creatinine clearance of <75%, uncontrolled diabetes mellitus, uncontrolled hypertension, heart failure (New York Heart Association class III/IV), alcohol or drug abuse, serious infections in the previous 3 months or chronic infectious disease, opportunistic infections within previous 2 months, active or latent hepatitis B infection, documented HIV infection or AIDS, lymphoproliferative disease and multiple sclerosis, active TB or UA patients with latent TB	BMI - continuous	601	RA = 52 (13)<br>UA = 52 (16)	RA = 333 (70)<br>UA = 74 (61)	**Industry** (Abbott)
Wolfe (2012) [USA][360]	Pros. Cohort	RA according to rheumatologist – National Databank for Rheumatoid Diseases	BMI categories:<br>Underweight: <18.5<br>Normal: 18.5-24.9<br>Overweight: 25.0-29.9<br>Obese: ≥30	24535	58.9 (13.2)	78%	**Not reported**
de Rooy (2011) [The Netherlands][361]	Pros. Cohort	Leiden cohort – symptom duration <2 years, RA confirmed by physical exam	BMI – continuous	676	56.4 (15.7)	459 (67.9)	**Professional body** (Dutch Arthritis Association), **Government** (The Netherlands Organization for Health Research)

AIDS = acquired immune deficiency syndrome, BMI = body mass index, DAS28 = Disease Activity Score 28, N = number, pros. = prospective, SD = standard deviation, TB = tuberculosis, UA = undifferentiated arthritis, USA = United States of America

Table – Rheumatoid arthritis, description of included studies

Author (date) [country]	Study design	Inclusion criteria	Exposure detail	N	Age, mean (SD) years	N (%) female	Funders
Klaasen (2011) [The Netherlands] [362]	Pros. Cohort	1987 ACR RA criteria, infliximab treatment, DAS28 ≥3.2<br>Exclusions: intra-articular injection of steroids in previous month to baseline	BMI categories:<br><20<br>20-30<br>>30	89	BMI <20:<br>50 (15)<br>20-30:<br>57 (11)<br>>30:<br>53 (15)	BMI <20: 6 (75)<br>20-30: 47 (71)<br>>30: 13 (87)	**Government** (The Netherlands Organization for Health Research and Development, The Netherlands Organization for Scientific Research, European Union), **Professional body** (Dutch Arthritis Association)
Liao (2011) [USA][363]	Pros. Cohort	Brigham Rheumatoid Arthritis Sequential Study (BRASS) - ≥18 years, diagnosis of RA from rheumatologist	BMI – continuous	Erosion: 215<br>No erosion: 56	Erosion: 51.3 (13.2)<br>No erosion: 45.0 (14.6)	Erosion: 175 (81.4)<br>no erosion: 44 (78.6)	**Government** (NIH), **Professional body** (ACR), **Charity** (Katherine Swan Ginsburg Fund)
Tekaya (2011) [Tunisia][364]	Pros. Cohort	ACR RA criteria	BMI categories:<br>Obese (≥30)<br>Not obese (<30)	119	51.03 (12.59)	92 (77.3)	**Not reported**
Pye (2010) [UK][365]	Pros. Cohort	NOAR – early inflammatory arthritis	BMI – continuous	108	58.0 (13.2)	108 (100)	**Charity** (Arthritis Research UK)

ACR = American College of Rheumatology, BMI = body mass index, DAS28 = Disease activity score 28, N = number, NOAR = Norfolk Arthritis Register, NIH = National Institutes of Health, pros. = prospective, SD = standard deviation, UK = United Kingdom, United States of America

Table – Rheumatoid arthritis, description of included studies

Author (date) [country]	Study design	Inclusion criteria	Exposure detail	N	Age, mean (SD) years	N (%) female	Funders
Verstappen (2010) [The Netherlands] [366]	Pros. Cohort	CAMERA study – 1) intensive methotrexate treatment, 2) conventional methotrexate treatment– RA patients, methotrexate usage for ≥1 week<br>Exclusions: Creatinine clearance of <75 ml/min, serum aspartate aminotransferase or serum alanine aminotransferase >2x the upper limit of normal (ULN) and alcohol intake of >2 units a day	BMI – continuous	1) 149<br>2) 140	1) 54)14)<br>2) 52 (15)	1) 68.5%<br>2) 65.0%	**Not reported**
Furuya (2009) [Japan][367]	Pros. Cohort	IORRA study – 1987 ACR RA criteria	BMI – continuous	5106	Median (IQR)<br>≥1 fall:<br>64 (54, 72)<br>≥2 falls:<br>64 (53, 74.8)<br>no falls:<br>60 (51, 67)	4231 (82.9)	**Industry** (36 pharmaceutical companies), **Charity** (Japanese Osteoporosis Foundation)
Hashimoto (2009) [Japan][368]	Pros. Cohort	SAMURAI RCT reanalysis – 1987 ACR RA criteria, randomised to TCZ mono or conventional DMARDs	BMI – continuous	145	53.1 (12.5)	119 (82.1)	**Industry** (Chugai)
van der Helm-van Mil (2008) [The Netherlands] [369]	Pros. Cohort	Leiden cohort and BeST trial reanalysis – 1987 ACR RA criteria, treatment before 2002	BMI categories:<br><25<br>25-30<br>≥30	Leiden: 332<br>BeST: 257	Leiden:<br>BMI<25:<br>54.5 (17.1)<br>BMI 25-30:<br>58.3 (13.9)<br>BMI> 30:<br>55.4 (13.5)<br>BeST:<br>BMI<25:<br>53.1 (14.7)<br>BMI25-30:<br>55.9 (12.5)<br>BMI >30:<br>54.2 (10.4)	Leiden:<br>BMI<25:<br>116 (74%)<br>BMI 25-30:<br>88 (60%)<br>BMI> 30:<br>20 (67%)<br>BeST<br>BMI<25:<br>84 (76%)<br>BMI25-30:<br>57 (56%)<br>BMI >30:<br>31 (86%)	**Government** (Dutch College of Health Insurances), **Industry** (Schering-Plough, BV and Centocor, Inc)
Cohen (2006) [France][370]	Pros. Cohort	1987 ACR RA criteria	BMI – continuous	191	50.5 (14.7)	140 (73.3)	**Not reported**

ACR = American College of Rheumatology, BMI = body mass index, CAMERA = Computer-Assisted Management in Early Rheumatoid Arthritis DMARD = disease modifying anti-rheumatic drug, IORRA = Institute of Rheumatology Rheumatoid Arthritis, IQR = interquartile range, N = number, pros. = prospective, SD = standard deviation, TCZ = tocilizumab

Table – Rheumatoid arthritis, description of included studies

Author (date) [country]	Study design	Inclusion criteria	Exposure detail	N	Age, mean (SD) years	N (%) female	Funders
Escalante (2005) [USA][371]	Pros. Cohort	1987 ACR RA criteria, aged ≥18 years	BMI categorised:<br>Underweight: ≤20<br>Normal: 20-<25<br>Overweight: 25-<30<br>Obese: ≥30	779	Underweight: 63 (16)<br>Normal: 56 (14)<br>Overweight: 58 (12)<br>Obese: 52 (13)	Underweight: 27 (71)<br>Normal: 137 (70)<br>Overweight: 165 (62)<br>Obese: 222 (79)	**Charity** (Arthritis Foundation), **Government** (NIH)
Maradit-Kremers (2005) [USA][372]	Pros. Cohort	Mayo Clinic – 1987 ACR RA criteria, aged ≥18 years	BMI categories<br>Low BMI: <20<br>High BMI: >30	603	58.0 (15.2)	441 (73.1)	**Government** (NIH), **Charity** (Luso-American Founcation)
Skoldstam (2005) [Sweden][373]	Pros. Cohort	Pooled data from three RCTs, analysed as a cohort study – 1984 criteria for RA	Dichotomised as either weight reduction or no weight reduction	102	Diet: 54.4 (33-73)<br>Control: 57.0 (35-75)	84 (82.4)	**Not reported**
Maradit-Kremers (2004) [USA][374]	Pros. Cohort	Mayo Clinic – 1987 ACR RA criteria	BMI categories<br>Low BMI: <20<br>High BMI: >30	603	58.0 (15.2)	441 (73.1)	**Government** (NIH), **Charity** (Luso-American Founcation)
Hoekstra (2003) [The Netherlands] [375]	Pros. Cohort	RCT re-analysis of MTX + folic acid trial 1) folic acid + MTX, p) placebo + MTX<br>Exclusions: prior MTX use, a creatinine clearance <50 ml/min (Cockroft formula),28 liver disorders, leucopenia, thrombopenia, alcohol abuse, and treatment with folic or folinic acid	BMI – continuous	1) 274<br>p) 137	1) 55.4 (12.7)<br>p) 57.2 (12.7)	1) 189 (69.0)<br>p) 100 (73.0)	**Not reported**
Krishnan (2012) [USA][376]	Time-trend analysis	ARAMIS study – 1987 ACR RA criteria, aged ≥17 years	BMI – dichotomised as ≥30 or <30	4651	1983: 57.2 (13.0)<br>2006: 64.3 (12.2)	76%	**Industry** (Centocor Ortho-Biotech)

ACR = American College of Rheumatology, BMI = body mass index, N = number, NIH = National Institutes of Health, pros. = prospective, RCT = randomised controlled trial, SD = standard deviation, United States of America

Table – Rheumatoid arthritis, description of included studies

Author (date) [country]	Study design	Inclusion criteria	Exposure detail	N	Age, mean (SD) years	N (%) female	Funders
Kreps (2018) [USA][377]	Retro. Cohort	Brigham and Women's Hospital - medical review confirmed 2010 ACR/EULAR criteria	Weight loss ≥5 kilograms	174	60.4 (13.2)	147 (84.4)	**Charity** (Rheumatology Research Foundation), **Government** (National Institute of Arthritis and Musculoskeletal and Skin Diseases)
Mori (2017) [Japan][378]	Retro. Cohort	SARABA study – first beginning a biologic DMARD, 1987 or 2010 RA criteria	BMI <18.5	1596	60.9 (14.2)	1237 (77.5)	**Hospital** (National Hospital Organization)
Rashid (2016) [USA][379]	Retro. Cohort	Aged ≥18 years, RA diagnosis (ICD9), taking a bDMARD Exclusions: Crohn's disease, psoriasis, psoriatic arthritis, ulcerative colitis, ankylosing spondylitis, regional enteritis, or anal fistula	BMI categories:<br>Normal: 18.5-24.99<br>Overweight: ≥25 & <30<br>Obese: ≥30	2172	50 (12.6)	1762 (81.2)	**Industry** (Bristol-Myers Squibb)
Ottaviani (2015) [France][380]	Retro. Cohort	2010 ACR / EULAR RA criteria, active RA, receiving rituximab	BMI – continuous	114	Median (IQR)<br>BMI<25: 50.1 (45.1, 61.3)<br>BMI 25-30: 54.5 (47.2, 60.8)<br>BMI >30: 58.1 (48.4, 61.1)	BMI <25: 33 (87)<br>BMI 25-30: 31 (76)<br>BMI >30: 29 (83)	**Industry** (Schering Plough)
Ottaviani (2015) [France][381]	Retro. Cohort	2010 ACR / EULAR RA criteria, active RA, receiving infliximab	BMI – continuous	76	Median (IQR): 49.1 (42.3, 55.8)	63 (82.9)	**Not reported** – Authors declared no conflicts of interest
Sparks (2015) [USA][382]	Retro. Cohort	1987 criteria for RA and underwent bariatric surgery	Weight loss and percentage excess weight loss (percentage of baseline weight loss at each postsurgical time point, 6, 12 and last follow-up)"	53	47.9 (10.5)	50 (94.3)	**Charity** (Rheumatology Research Foundation), **Government** (NIH)

ACR = American College of Rheumatology, BMI = body mass index, DMARD = disease modifying anti-rheumatic drug, EULAR = European League Against Rheumatism, IQR = interquartile range, N = number, NIH = National Institutes of Health, Retro. = retrospective, SD = standard deviation, USA = United States of America

Table – Rheumatoid arthritis, description of included studies

Author (date) [country]	Study design	Inclusion criteria	Exposure detail	N	Age, mean (SD) years	N (%) female	Funders
Gonzalez (2008) [USA][383]	Retro. Cohort	Mayo Clinic – 1987 ACR RA criteria, inception cohort, aged ≥18 years	BMI categories:<br>Obese = ≥30 BMI<br>Low BMI = <20 BMI	603	58	73%	**Government** (NIH)
Kent (2004) [USA][384]	Retro. Cohort	Mayo Clinic	BMI – continuous	481	47 (14)	334 (69.4)	**Not reported**
Nadareishvili (2008) [USA][83]	Nested Case control	National Database for Rheumatoid Diseases - aged 25-110	BMI – continuous	1230	70.0 (9.6)	73.2	**Industry** (Centocor, Sanofi-Aventis, Bristol-Myers Squibb, Abbott, Amgen, Wyeth-Australia, Merck, Pfizer)

ACR = American College of Rheumatology, BMI = body mass index, N = number, NIH = National Institutes of Health, Retro. = retrospective, SD = standard deviation, United States of America

Supplementary table 117 – Pain outcomes from observational studies in RA

Table – Pain (RA), results and quality assessment of observational studies

Study (date) [study type]	Results	Weight associated with outcome	AMSTAR2	Study Pop.	Attr.	Prog. Meas.	Outc. Meas.	Conf.	Stats.
Liu (2017) [MA][322]	Pain<br>3/3 studies reported higher pain scores in obese patients vs non-obese	✓	Moderate						
McWilliams (2016) [Prospective cohort][347]	SF36 pain >median at 1 year, TNF / non-TNF OR (95% CI)<br>BMI <25: ref<br>25-30: 1.22 (1.10, 1.35) / 1.12 (0.92, 1.35) [unadj]<br>≥30: 1.48 (1.33, 1.66) / 1.60 (1.30, 1.98) [unadj]<br>BMI per group increase: 1.13 (1.05, 1.21) / 1.07 (0.93, 1.22) [adj]	✓		L	M	L	L	L	L
Sandberg (2014) [Prospective cohort][354]	Pain VAS remission, 3 mth/6mth, OR (95% CI) [adjusted]<br>normal: ref<br>overweight: 0.59 (0.37, 0.95) / 0.67 (0.42, 1.07)<br>obese: 0.53 (0.29, 0.97) / 0.59 (0.30, 1.16)<br>per unit BMI: 0.96 (0.92, 1.00) / 0.95 (0.90, 1.00)<br>Pain decrease over median, 3 mth/6mth, OR (95% CI) [adjusted]<br>normal: ref<br>overweight: 0.75 (0.45, 1.27) / 0.73 (0.42, 1.28)<br>obese: 0.43 (0.22, 0.85) / 0.73 (0.33, 1.62)<br>per unit BMI: 0.93 (0.88, 0.98) / 0.98 (0.92, 1.04)	✓		L	M	L	L	L	L
Ajeganova (2013) [Prospective cohort][355]	VAS pain, mean difference (95% CI)<br>BMI at BL: 0.014 (0.003, 0.025)<br>BMI≥30 vs 20-30 at BL: 0.05 (-0.02, 0.12)<br>BMI≥28 vs 20-28 at BL: 0.08 (0.03, 0.14)<br>Waist circumference: 0.02 (-0.02, 0.06)<br>Central obesity: 0.04 (-0.04, 0.12)	✓		L	M	M	L	L	L
Skoldstam (2005) [Prospective cohort][373]	OR between reduction and no reduction of body weight with outcome [unadjusted]<br>Dichotomised pain score (improvement or no improvement:<br>OR 2.10 (p=0.10)	×		L	L	L	L	H	H

AMSTAR2 = Assessing the Methodological Quality of Systematic Reviews, Attr. = attrition, BL = baseline, BMI = body mass index, CI = confidence interval, Conf. = confounding, L = low risk of bias, M = moderate risk of bias, MA = meta-analysis, OR = odds ratio, Outc. Meas = outcome measurement, Prog. Meas. = prognostic factor measurement, RA = rheumatoid arthritis, Rand. Seq. = random sequence generation, SD = standard deviation, Stats. = statistical analysis, Study Pop. = study population, TNF = tumour necrosis factor, VAS = visual analogue scale

Supplementary table 118 – Function outcomes from observational studies in RA

Table – Function (RA), results and quality assessment of observational studies

Study (date) [study type]	Results	Weight associated with outcome	AMSTAR2	Study Pop.	Attr.	Prog. Meas.	Outc. Meas.	Conf.	Stats.
Liu (2017) [MA][322]	HAQ<br>2/2 studies reported higher HAQ scores in obese patients vs non-obese	✓	Moderate						
Nikiphorou (2018) [Prospective cohort][329]	HAQ at 2 / 5 years, mean (95% CI) [adjusted]<br>BL normal/over: 0.83 (0.79, 0.87) / 0.99 (0.94, 1.04)<br>BL obese: 0.98 (0.89, 1.07) / 1.07 (0.97, 1.17)<br>BL underweight: 0.88 (0.57, 1.18) / 0.84 (0.51, 1.18)<br>p values obese vs normal: 0.003 / 0.165<br>p values underweight vs normal: 0.764 / 0.405	✓		L	L	L	L	L	L
George (2017) [Prospective cohort][337]	Low HAQ (≤0.5) at week 24, OR (95% CI) [adjusted]<br>obese vs not: 0.49 (0.28, 0.89)	✓		L	L	L	L	L	M
Miwa (2017) [Prospective cohort][342]	HAQ <0.5 at follow-up, median (IQR) BMI and BL<br>HAQ ≤0.5: 21 (20, 24)<br>HAQ >0.5: 21 (19, 25) p=0.830	×		M	M	L	L	H	H
Ajeganova (2013) [Prospective cohort][355]	HAQ, mean difference (95% CI)<br>BMI at BL: 0.021 per unit BMI (0.009 0.033)<br>BMI≥30 vs 20-30 at BL: 0.10 (0.03, 0.17)<br>BMI≥28 vs 20-28 at BL: 0.11 (0.05, 0.07)<br>Waist circumference: 0.09 (0.05, 0.11)<br>Central obesity: 0.09 (0.01, 0.18)	✓		L	M	M	L	L	L
Tekaya (2011) [Prospective cohort][364]	HAQ<br>obese: 1.49 (0.81)<br>non obese: 1.38 (0.84) p=0.51	×		L	L	L	L	H	H
Krishnan (2012) [Time-trend analysis][376]	HAQ slope over time, regression coef (95% CI)<br>BMI ≥30: -0.0210 (-0.024, -0.018)<br>BMI <30: -0.0160 (-0.017, -0.014)<br>i.e. in both subgroups, HAQ was getting lower over time	✓		L	L	L	L	L	M

AMSTAR2 = Assessing the Methodological Quality of Systematic Reviews, Attr. = attrition, BL = baseline, BMI = body mass index, CI = confidence interval, Conf. = confounding, HAQ = Health Assessment Questionnaire, IQR = interquartile range, L = low risk of bias, M = moderate risk of bias, MA = meta-analysis, Outc. Meas = outcome measurement, Prog. Meas. = prognostic factor measurement, Pros = prospective, RA = rheumatoid arthritis, Rand. Seq. = random sequence generation, SD = standard deviation, Stats. = statistical analysis, Study Pop. = study population

Supplementary table 119 – Disease activity outcomes from observational studies in RA

Table – Disease activity (RA), results and quality assessment of observational studies

Study (date) [study type]	Results	Weight associated with outcome	AMSTAR2	Study Pop.	Attr.	Prog. Meas.	Outc. Meas.	Conf.	Stats.
Liu (2017) [MA][322]	Remission<br>Obese vs non-obese: Meta-OR 0.53 (0.41, 0.69)<br>DAS28<br>6/8 studies reported higher DAS28 in obese patients compared to non-obese patients	✓	Moderate						
Lupoli (2016) [MA][323]	Minimal disease activity<br>Obese vs non-obese: OR 0.58 (0.40, 0.85)	✓	Low						
Nikiphorou (2018) [Prospective cohort][329]	DAS28, 2 years / 5 years, mean (95% CI) [adjusted]<br>BL normal/over: 3.53 (3.44, 3.62) / 3.81 (3.71, 3.90)<br>BL obese: 3.85 (3.68, 4.03) / 3.85 (3.64, 4.05)<br>BL underweight: 3.89 (3.29, 4.50) / 3.35 (2.68, 4.01)<br>p values obese vs normal: 0.001 / 0.727<br>p values underweight vs normal: 0.243 / 0.182<br>low disease activity, bl-2 year, OR (95% CI) [adjusted]<br>obese vs normal/over: 0.52 (0.41, 0.65)	✓		L	L	L	L	L	L
Schulman (2018) [Prospective cohort][331]	Sustained remission, adjusted HR (95%CI)<br>Healthy BMI: ref<br>Overweight: HR 0.75 (0.58-0.98)<br>Obese: HR 0.53 (0.39-0.71)	✓		L	H	L	L	L	M
Smolen (2018) [Prospective cohort][332]	DAS28 remission, OR (95% CI) [adjusted]<br><18.5=ref<br>18.5-25: 1.41 (0.57-3.51)<br>25-30: 1.25 (0.50-3.13)<br>>30: 0.79 (0.31-2.05)<br>BMI continuous: 0.96 (0.93, 0.99)	✓		L	L	L	L	L	M
Bird (2017) [Prospective cohort][335]	DAS28 remission, unadjusted OR (95% CI) / adjusted<br>Weight per kg: OR 0.99 (0.97, 1.00) / 0.98 (0.97, 1.00)	✓		M	H	L	L	M	M

AMSTAR2 = Assessing the Methodological Quality of Systematic Reviews, Attr. = attrition, BL = baseline, BMI = body mass index, CI = confidence interval, Conf. = confounding, DAS28 = Disease activity score (28), HR = hazard ratio, L = low risk of bias, M = moderate risk of bias, MA = meta-analysis, OR = odds ratio, Outc. Meas = outcome measurement, Prog. Meas. = prognostic factor measurement, RA = rheumatoid arthritis, Rand. Seq. = random sequence generation, Stats. = statistical analysis, Study Pop. = study population

Table – Disease activity (RA), results and quality assessment of observational studies

Study (date) [study type]	Results	Weight associated with outcome	AMSTAR2	Study Pop.	Attr.	Prog. Meas.	Outc. Meas.	Conf.	Stats.
D'Agostino (2017) [Prospective cohort][336] (abatacept)	DAS28 remission, % (95% CI) at 6 mths<br>BMI <25: 26.2 (22.2, 30.1)<br>BMI 25-30: 24.9 (20.9, 28.8)<br>BMI ≥30: 22.0 (17.9, 26.0)<br>SDAI // CDAI remission, % (95% CI) at 6 mths<br>BMI <25: 9.9 (7.2, 12.5) // 10.4 (7.6, 13.1)<br>BMI 25-30: 11.7 (8.8, 14.7) // 13.4 (10.3, 16.5)<br>BMI ≥30: 10.9 (7.8, 14.0) // 13.1 (9.8, 16.4)	✗		M	L	L	L	H	M
George (2017) [Prospective cohort][337]	DAS28 remission at week 24, OR (95% CI) [adjusted]<br>Obese vs not: OR 0.47 (0.24, 0.92)	✓		L	L	L	L	L	M
Iannone (2017) [Prospective cohort][338] (abatacept)	EULAR mod/good response, N(%) / difference from normal weight (95% CI) §<br>Normal: 39.8%<br>Overweight: 42.9% / 3.1% (-0.3, 0.1) [sic]<br>Obese: 40.0% / 0.2% (-0.08, 0.08) [sic]<br>Severely obese: 49.4% / 9.6% (-0.04, 0.23)	✗		L	H	L	L	L	M
Levitsky (2017) [Prospective cohort][340]	Non-remission at 24 months, OR (95% CI)<br>BL obesity: 5.4 (1.9, 15.2) [unadjusted] / 5.2 (1.8, 15.2) [adjusted]	✓		L	M	L	L	L	M
Mariette (2017) [Prospective cohort][341] (abatacept)	Moderate/good response, %<br>BMI <25: 80.7%<br>BMI 25-30: 86.1%<br>BMI ≥30: 77.0% p=0.178	✗		L	L	L	L	L	M
Ramirez (2017) [Prospective cohort][343]	Synovitis score, change BL-12 months, regression coefficient [adjusted]<br>"Higher" BMI 0.22 – no CI reported	✓		L	L	L	L	M	M
Gardette (2016) [Prospective cohort][345] (abatacept)	DAS28 decrease ≥1.2, median (IQR) BMI<br>DAS response: 25.0 (23.4-31.3)<br>no DAS response: 26.3 (22.9, 30.2) p=0.95<br>EULAR good response, median (IQR) BMI<br>good response: 26.4 (23.5, 30.9)<br>no good response: 26.0 (22.9, 30.6) p=0.96<br>EULAR remission, median (IQR) BMI<br>remission: 26.7 (21.7, 30.3)<br>no remission: 26.0 (23.0, 30.1) p=0.83	✗		L	L	L	L	H	H

§ Outcome actually LUNDEX – EULAR response adjusted for drug retention

AMSTAR2 = Assessing the Methodological Quality of Systematic Reviews, Attr. = attrition, BL = baseline, BMI = body mass index, CDAI = Clinical Disease Activity Index, CI = confidence interval, Conf. = confounding, DAS28 = Disease activity score (28), EULAR = European League Against Rheumatism, IQR = interquartile range, L = low risk of bias, M = moderate risk of bias, OR = odds ratio, Outc. Meas = outcome measurement, Prog. Meas. = prognostic factor measurement, RA = rheumatoid arthritis, Rand. Seq. = random sequence generation, SD = standard deviation, SDAI = Simplified Disease Activity Index, Stats. = statistical analysis, Study Pop. = study population

Table – Disease activity (RA), results and quality assessment of observational studies

Study (date) [study type]	Results	Weight associated with outcome	AMSTAR2	Study Pop.	Attr.	Prog. Meas.	Outc. Meas.	Conf.	Stats.
Gardette (2016) [Prospective cohort][346] (tocilizumab)	<u>DAS28 decrease ≥1.2, median (IQR) BMI</u><br>DAS response: 25.7 (22.1, 29.9)<br>No DAS response: 24.9 (22.0, 27.1) p=0.38<br><u>EULAR good response, median (IQR) BMI</u><br>Good response: 25.9 (22.8, 30.0)<br>No good response: 25.4 (22.0, 28.4) p=0.61<br><u>EULAR remission, median (IQR) BMI</u><br>Remission: 25.1 (22.5, 28.6)<br>No remission: 25.4 (22.0, 28.9) p=0.76	×		L	L	L	L	H	H
Iannone (2015) [Prospective cohort][350]	<u>DAS28 remission at 12 months, N(%)</u><br>Normal: 46%<br>Overweight: 55%<br>Obese: 12% p=0.004<br><u>EULAR good response at 12 months, N(%)</u><br>Normal: 75%<br>Overweight: 79%<br>Obese: 33% p=0.01	✓		L	L	L	L	L	L
Pers (2015) [Prospective cohort][351] (tocilizumab)	<u>EULAR response, OR (95% CI)</u><br><25 BMI: ref<br>25-30: OR 0.45 (0.16, 1.24)<br>>30: OR 1.19 (0.31, 4.48)<br>>25 vs <25: OR 0.64 (0.26, 1.60)<br><u>Remission, OR (95% CI)</u><br><25 BMI: ref<br>25-30: OR 0.41 (0.14, 1.16)<br>>30: 0.61 (0.21, 1.70)<br>> 25 vs <25: OR 0.50 (0.22, 1.14)<br><u>Low disease activity, OR (95% CI)</u><br><25 BMI: ref<br>25-30: OR 0.59 (0.23, 1.55)<br>>30: OR 1.41 (0.46, 4.36)<br>>25 vs <25: OR 0.84 (0.37, 1.91)	×		L	L	L	L	L	L

AMSTAR2 = Assessing the Methodological Quality of Systematic Reviews, Attr. = attrition, BMI = body mass index, CI = confidence interval, Conf. = confounding, DAS28 = Disease activity score (28), EULAR = European League Against Rheumatism, IQR = interquartile range, L = low risk of bias, M = moderate risk of bias, N = number, OR = odds ratio, Outc. Meas = outcome measurement, Prog. Meas. = prognostic factor measurement, RA = rheumatoid arthritis, Rand. Seq. = random sequence generation, SD = standard deviation, Stats. = statistical analysis, Study Pop. = study population

Table – Disease activity (RA), results and quality assessment of observational studies

Study (date) [study type]	Results	Weight associated with outcome	AMSTAR2	Study Pop.	Attr.	Prog. Meas.	Outc. Meas.	Conf.	Stats.
Sandberg (2014) [Prospective cohort][354]	DAS28 (decrease over median), 3 mth/6mth, OR (95% CI) [adjusted]<br>Normal: ref<br>Overweight: 0.71 (0.45, 1.13) / 0.54 (0.32, 0.90)<br>Obese: 0.37 (0.20, 0.68) / 0.44 (0.22, 0.90)<br>Per unit BMI: 0.93 (0.88, 0.97) / 0.94 (0.89, 0.99)<br>DAS28 (low disease activity), 3 mth/6mth, OR (95% CI) [adjusted]<br>Normal: ref<br>Overweight: 0.73 (0.47, 1.13) / 0.50 (0.30, 0.81)<br>Obese: 0.56 (0.32, 0.99) / 0.48 (0.25, 0.94)<br>Per unit BMI: 0.95 (0.91, 0.99) / 0.94 (0.89, 0.99)<br>EULAR good response, 3 mth/6mth, OR (95% CI) [adjusted]<br>Normal: ref<br>Overweight: 0.82 (0.54, 1.26) / 0.50 (0.31, 0.81)<br>Obese: 0.62 (0.36, 1.09) / 0.48 (0.25, 0.92)<br>Per unit BMI: 0.96 (0.92, 1.00) / 0.94 (0.89, 0.99)<br>EULAR remission, 3 mth/6mth, OR (95% CI) [adjusted]<br>Normal: ref<br>Overweight: 0.91 (0.58, 1.41) / 0.68 (0.42, 1.10)<br>Obese: 0.76 (0.43, 1.37) / 0.36 (0.18, 0.74)<br>Per unit BMI: 0.97 (0.93, 1.02) / 0.92 (0.87, 0.97)	✓		L	M	L	L	L	L
Ajeganova (2013) [Prospective cohort][355]	DAS28, mean difference (95% CI)<br>BMI at BL: 0.008 per unit BMI (0.002, 0.014)<br>BMI≥30 vs 20-30 at BL: 0.03 (0, 0.06)<br>BMI≥28 vs 20-30 at BL: 0.04 (0.02, 0.07)<br>Waist circumference: 0.01 (-0.01, 0.03)<br>Central bbesity: 0.04 (0.01, 0.08)	✓		L	M	M	L	L	L
Gremese (2013) [Prospective cohort][356]	DAS28 remission at 12 months, OR (95% CI)<br>BMI continuous: 0.892 (0.806, 0.987) [adjusted]<br>BMI <20 vs 20-30: 2.03 (1.91, 3.46)<br>BMI 20-30 vs >30: 2.43 (1.21, 4.88)	✓		L	H	L	L	L	M
Wevers-de Boer (2012) [Prospective cohort][359]	DAS28<1.6 multivariable, OR<br>BMI = 0.94 (0.90 to 0.98)	✓		L	L	L	L	M	M
Klaasen (2011) [Prospective cohort][362] (infliximab)	DAS28, change BL-16 weeks, mean diff (95% CI)<br>BMI continuous: -0.094 (-0.149, -0.038)<br>DAS28 response, change BL-16 weeks, %<br><20: 84%<br>20-30: 75%<br>>30: 50%	✓		M	L	L	L	M	H

AMSTAR2 = Assessing the Methodological Quality of Systematic Reviews, Attr. = attrition, BMI = body mass index, BL = baseline, BMI = body mass index, CI = confidence interval, Conf. = confounding, DAS28 = Disease activity score (28), EULAR = European League Against Rheumatism, L = low risk of bias, M = moderate risk of bias, OR = odds ratio, Outc. Meas = outcome measurement, Prog. Meas. = prognostic factor measurement, Pros = prospective, RA = rheumatoid arthritis, Rand. Seq. = random sequence generation, stats. = statistical analysis, Study Pop. = study population

Table – Disease activity (RA), results and quality assessment of observational studies

Study (date) [study type]	Results	Weight associated with outcome	AMSTAR2	Study Pop.	Attr.	Prog. Meas.	Outc. Meas.	Conf.	Stats.
Kreps (2018) [Retrospective cohort][377]	≥ 5 point improvement CDAI, OR (95% CI) [adj]<br>BMI ≥25 and did not lose 5kg: ref<br>BMI ≥25 & lost 5kg: 3.03 (1.18, 7.83)<br>BMI <25 and did not lose 5kg: 1.90 (0.88. 4.11)	✓		L	L	L	L	M	L
Ottaviani (2015) [Retrospective cohort][380] (rituximab)	DAS28 response, median BL BMI<br>Response: BMI 26.9 (24.1, 30.1)<br>No response: BMI 26.8 (23.2, 31.6) p=0.78 [unadj]<br>EULAR good response, median BL BMI<br>Response: BMI 27.7 (24.3, 30.7)<br>No response: BMI 26.7 (22.3, 31.5) p=0.57<br>EULAR remission, , median BL BMI<br>Remission: 26.9 (24.1, 30.8)<br>No remission: 26.8 (23.2, 31.5) p=0.94	×		L	L	L	L	M	M
Ottaviani (2015) [Retrospective cohort][381] (infliximab)	DAS response, OR (95% CI)<br>BMI 0.88 (0.79, 0.98) [adj]<br>EULAR good response, OR (95% CI)<br>BMI: 0.87 (0.76, 0.99) [adj]<br>EULAR remission, OR (95% CI)<br>BMI 0.88 (0.75, 1.04) [adj]	✓		L	L	L	L	M	M
Sparks (2015) [Retrospective cohort][382]	N (%) 6 months after bariatric surgery compared to baseline, § p<0.001:<br>Remission= 38 (72%) §<br>Low DAS =12 (23%) §<br>Moderate DAS = 2 (4%)<br>High DAS = 1 (2%)<br>N(%) 12 months after bariatric surgery compared to baseline, sign p<0.001:<br>Remission: 36 (68%)<br>Low DAS: 9 (17%)<br>Moderate DAS: 3 (6%)<br>High DAS: 0 (0%)	✓		L	L	L	L	M	M

AMSTAR2 = Assessing the Methodological Quality of Systematic Reviews, Attr. = attrition, BL = baseline, BMI = body mass index, CDAI = Clinical Disease Activity Index, CI = confidence interval, Conf. = confounding, DAS28 = Disease activity score (28), EULAR = European League Against Rheumatism, L = low risk of bias, M = moderate risk of bias, N = number, OR = odds ratio, Outc. Meas = outcome measurement, Prog. Meas. = prognostic factor measurement, RA = rheumatoid arthritis, Rand. Seq. = random sequence generation, Stats. = statistical analysis, Study Pop. = study population

Supplementary table 120 – Tender joint count outcomes from observational studies in RA

Table – Tender joint count (RA), results and quality assessment of observational studies

Study (date) [study type]	Results	Weight associated with outcome	AMSTAR2	Study Pop.	Attr.	Prog. Meas.	Outc. Meas.	Conf.	Stats.
Liu (2017) [MA][322]	Tender joint count<br>2/3 studies reported higher TJC in obese vs non-obese patients	✓	Moderate						
D'Agostino (2017) [Prospective cohort][336] (abatacept)	Tender joint count, change BL-6 mths, mean (SD)<br>BMI <25: -11.4 (0.3)<br>BMI 25-30: -11.4 (0.3)<br>BMI ≥30: -11.7 (0.4)	×		M	L	L	L	H	M
Klaasen (2011) [Prospective cohort][362] (infliximab)	Tender joint count, change BL-16 weeks, mean diff (95% CI)<br>BMI cont: -0.482 (-0.745, -0.218)	✓		M	L	L	L	M	H
Skoldstam (2005) [Prospective cohort][373]	OR between reduction and no reduction of body weight with outcome [unadjusted]<br>Dichotomised tender joint count: 1.77 (p=0.20)	×		L	L	L	L	H	H

AMSTAR2 = Assessing the Methodological Quality of Systematic Reviews, Attr. = attrition, BL = baseline, BMI = body mass index, CI = confidence interval, Conf. = confounding, L = low risk of bias, M = moderate risk of bias, MA = meta-analysis, OR = odds ratio, Outc. Meas = outcome measurement, Prog. Meas. = prognostic factor measurement, RA = rheumatoid arthritis, Rand. Seq. = random sequence generation, SD = standard deviation, SE = standard error, SMD = Standardised mean difference, Stats. = statistical analysis, Study Pop. = study population

Supplementary table 121 – Swollen joint count outcomes from observational studies in RA

Table – Swollen joint count (RA), results and quality assessment of observational studies

Study (date) [study type]	Results	Weight associated with outcome	AMSTAR2	Study Pop.	Attr.	Prog. Meas.	Outc. Meas.	Conf.	Stats.
Liu (2017) [MA][322]	Swollen joint count<br>0/5 studies reported high swollen joint count in obese vs non-obese patients	×	Moderate						
D'Agostino (2017) [Prospective cohort][336] (abatacept)	Swollen joint count, change BL-6 mths, mean (SD)<br>BMI <25: -10.0 (0.3)<br>BMI 25-30: -8.5 (0.3)<br>BMI ≥30: -10.0 (0.3)	×		M	L	L	L	H	M
Klaasen (2011) [Prospective cohort][362] (infliximab)	Swollen joint count, change BL-16 weeks, mean diff (95% CI)<br>BMI cont: -0.196 (-0.401, 0.009)	×		M	L	L	L	M	H

AMSTAR2 = Assessing the Methodological Quality of Systematic Reviews, Attr. = attrition, BL = baseline, BMI = body mass index, CI = confidence interval, Conf. = confounding, H = high, L = low risk of bias, M = moderate risk of bias, MA = meta-analysis, Outc. Meas = outcome measurement, Prog. Meas. = prognostic factor measurement, Pros = prospective, RA = rheumatoid arthritis, Rand. Seq. = random sequence generation, SD = standard deviation, Stats. = statistical analysis, Study Pop. = study population

Supplementary table 122 – Patient global assessment outcomes from observational studies in RA

Table – Patient global assessment (RA), results and quality assessment of observational studies

Study (date) [study type]	Results	Weight associated with outcome	AMSTAR2	Study Pop.	Attr.	Prog. Meas.	Outc. Meas.	Conf.	Stats.
Liu (2017) [MA][322]	Patient global<br>3/3 studies reported higher scores in obese vs non-obese patients	✓	Moderate						
D'Agostino (2017) [Prospective cohort][336] (abatacept)	Patient global (0-100), change BL-6 mths, mean (SD)<br>BMI <25: -35.7 (1.2)<br>BMI 25-30: -34.8 (1.2)<br>BMI ≥30: -32.3 (1.4)	×		M	L	L	L	H	M
George (2017) [Prospective cohort][337]	Patient global ≤1, OR (95% CI) [adjusted]<br>obese vs not: 0.47 (0.24, 0.92)	✓		L	L	L	L	L	M
Klaasen (2011) [Prospective cohort][362] (infliximab)	Patient global VAS, change BL-16 weeks, mean diff (95% CI)<br>BMI continuous: -1.080 (-2.107, -0.052)	✓		M	L	L	L	M	H

AMSTAR2 = Assessing the Methodological Quality of Systematic Reviews, Attr. = attrition, BL = baseline, BMI = body mass inde, CI = confidence interval, Conf. = confounding, L = low risk of bias, M = moderate risk of bias, MA = meta-analysis, OR = odds ratio, Outc. Meas = outcome measurement, Prog. Meas. = prognostic factor measurement, Pros = prospective, RA = rheumatoid arthritis, Rand. Seq. = random sequence generation, SD = standard deviation, Stats. = statistical analysis, Study Pop. = study population, VAS = visual analogue scale

Supplementary table 123 – Fatigue outcomes from observational studies in RA

Table – Fatigue (RA), results and quality assessment of observational studies

Study (date) [study type]	Results	Weight associated with outcome	Study Pop.	Attr.	Prog. Meas.	Outc. Meas.	Conf.	Stats.
Feldthusen (2016) [Prospective cohort][344]	BRAF-MDQ total, regression coefficient (95% CI) [adjusted]<br>BMI 0.412 (-0.00976, 0.834)<br>BRAF-MDQ physical, regression coefficient (95% CI) [adjusted]<br>0.179 (-0.00823, 0.367)<br>BRAF-MDQ living, regression coefficient (95% CI) [adjusted]<br>0.153 (0.00885, 0.298)<br>BRAF-MDQ emotion, regression coefficient (95% CI) [adjusted]<br>0.0734 (-0.0346, 0.181)	✓	L	H	L	L	L	M

Attr. = attrition, BL = Baseline, BMI = body mass index, BRAF-MDQ = Bristol Rheumatoid Arthritis Fatigue – Multidimensional Assessment Questionnaire, CI = confidence interval, Conf. = confounding, L = low risk of bias, M = moderate risk of bias, Outc. Meas = outcome measurement, Prog. Meas. = prognostic factor measurement, Pros = prospective, RA = rheumatoid arthritis, Rand. Seq. = random sequence generation, Stats. = statistical analysis, Study Pop. = study population

Supplementary table 124 – Mental health outcomes from observational studies in RA

Table – Mental health (RA), results and quality assessment of observational studies

Study (date) [study type]	Results	Weight associated with outcome	Study Pop.	Attr.	Prog. Meas.	Outc. Meas.	Conf.	Stats.
Kanecki (2013) [Prospective cohort][357]	SF36 mental health component, correlation coef, p<br>BMI: r -0.31 p<0.05	✓	L	L	L	L	H	H
Cohen (2006) [Prospective cohort][370]	AIMS affect scale, OR (95%CI)<br>BMI 4.31 (1.59, 11.7) [cut-points not specified]	✓	L	M	L	L	M	M

AIMS = Arthritis Impact Measurement Scales, Attr. = attrition, BMI = body mass index, CI = confidence interval, Conf. = confounding, L = low risk of bias, M = moderate risk of bias, OR = odds ratio, Outc. Meas = outcome measurement, Prog. Meas. = prognostic factor measurement, Pros = prospective, RA = rheumatoid arthritis, Rand. Seq. = random sequence generation, Stats. = statistical analysis, Study Pop. = study population

Supplementary table 125 – Radiographic outcomes from observational studies in RA

Table – Radiographic damage (RA), results and quality assessment of observational studies

Study (date) [study type]	Results	Weight associated with outcome	Study Pop.	Attr.	Prog. Meas.	Outc. Meas.	Conf.	Stats.
Lechtenboehmer (2019) [Prospective cohort][327]	OA progression, OR (95%) [adjusted]<br>BMI cont: 1.03 (1.00, 1.06)	✓	L	L	L	L	L	L
Rydell (2018) [Prospective cohort][330]	Rapid radiographic progression up to 5 years, OR (95% CI)<br>BMI per SD: 0.67 (0.44, 1.03) [adj]<br>normal BMI: ref<br>obese: 0.07 (0.01, 0.58)<br>obese or overweight: 0.27 (0.12, 0.63)<br>overweight: 0.36 (0.15, 0.84)	✓	L	M	L	L	L	M
van der Heijde (2018) [Prospective cohort][334]	Structural damage progression, OR (95% CI) [adjusted]<br>Baseline BMI: 0.94 (0.89, 0.99)	✓	L	L	L	L	L	M
Joo (2017) [Prospective cohort][339]	Joint damage, OR (95% CI)<br>BMI 0.91 (0.84, 0.99) [unadjusted]<br>BMI 0.88 (0.80, 0.97) [adjusted]	✓	L	M	L	L	L	M
Levitsky (2017) [Prospective cohort][340]	Radiographic progression bl-24 months, OR (95% CI)<br>obese: 0.46 (0.22, 0.99) [unadjusted] / 0.37 (0.13, 1.1) [adjusted]	✓	L	M	L	L	L	M
Ramirez (2017) [Prospective cohort][343]	erosion score, change BL-12 months, reg coef [adjusted]<br>"higher" BMI: 0.1 – no confidence interval reported	✓	L	L	L	L	M	M
de Rooy (2011) [Prospective cohort][361]	Rate of joint progression, exponentiated regression coefficient (95%CI) [adjusted]<br>BMI: 0.96 (0.94, 0.98)	✓	L	M	M	L	L	L
Liao (2011) [Prospective cohort][363]	Erosions at 2 years, mean (SD) BL BMI<br>erosion: 26.5 (5.3)<br>no erosion: 27.4 (5.9) p=0.34	×	L	L	L	L	H	H

Attr. = attrition, BL = basline, BMI = body mass index, CI = confidence interval, Conf. = confounding, L = low risk of bias, M = moderate risk of bias, OA = osteoarthritis, OR = odds ratio, Outc. Meas = outcome measurement, Prog. Meas. = prognostic factor measurement, Pros = prospective, RA = rheumatoid arthritis, Rand. Seq. = random sequence generation, SD = standard deviation, Stats. = statistical analysis, Study Pop. = study population

Table – Radiographic damage (RA), results and quality assessment of observational studies

Study (date) [study type]	Results	Weight associated with outcome	Study Pop.	Attr.	Prog. Meas.	Outc. Meas.	Conf.	Stats.
Tekaya (2011) [Prospective cohort][364]	Sharp score, mean (SD)<br>Obese: 64.97 (82.28)<br>Non obese: 113.64 (122.62) p=0.032	✓	L	M	M	L	L	L
Hashimoto (2009) [Prospective cohort][368]	Total sharp score, mean change per unit BMI (p value) [adjusted]<br>BMI: -0.92 (<0.01)<br>Bone erosion progression, mean change per unit BMI (p value) [adjusted]<br>BMI: -0.48 (0.02)	✓	L	L	L	L	L	M
van der Helm-van Mil (2008) [Prospective cohort][369]	Sharp score, regression coefficient (SE)<br>Leiden: BMI continuous = -0.65 (0.29), p=0.026<br>BesT: BMI continuous = --0.94 (0.29) p<0.001	✓	L	M	L	L	M	M

Attr. = attrition, BMI = bosy mass index, CI = confidence interval, Conf. = confounding, L = low risk of bias, M = moderate risk of bias, Outc. Meas = outcome measurement, Prog. Meas. = prognostic factor measurement, RA = rheumatoid arthritis, Rand. Seq. = random sequence generation, SD = standard deviation, SE = standard error, Stats. = statistical analysis, Study Pop. = study population

Supplementary table 126 – Bone mineral density outcomes from observational studies in RA

Table – Bone mineral density (RA), results and quality assessment of observational studies

Study (date) [study type]	Results	Weight associated with outcome	Study Pop.	Attr.	Prog. Meas.	Outc. Meas.	Conf.	Stats.
Pye (2010) [Prospective cohort][365]	Femoral neck BMD, regression coefficient (95% CI)<br>BMI: 0.008 (0.002, 0.014)<br>Lumbar spine BMD, regression coefficient (95% CI)<br>BMI: 0.008 (0.001, 0.016)	✓	L	L	L	L	M	M

Attr. = attrition, BMI = body mass index, BMD = bone mineral density, BMI = body mass index, CI = confidence interval, Conf. = confounding, L = low risk of bias, M = moderate risk of bias, Outc. Meas = outcome measurement, Prog. Meas. = prognostic factor measurement, Pros = prospective, RA = rheumatoid arthritis, Rand. Seq. = random sequence generation,, Stats. = statistical analysis, Study Pop. = study population

Supplementary table 127 – Joint space narrowing outcomes from observational studies in RA

Table – Joint space narrowing (RA), results and quality assessment of observational studies

Study (date) [study type]	Results	Weight associated with outcome	Study Pop.	Attr.	Prog. Meas.	Outc. Meas.	Conf.	Stats.
Hashimoto (2009) [Prospective cohort][368]	Joint space narrowing progression, mean change per unit BMI (p value)<br>-0.46 (<0.01)	✓	L	L	L	L	L	M

Attr. = attrition, BMI = body mass index, CI = confidence interval, Conf. = confounding, L = low risk of bias, M = moderate risk of bias, Outc. Meas = outcome measurement, Prog. Meas. = prognostic factor measurement, Pros = prospective, RA = rheumatoid arthritis, Rand. Seq. = random sequence generation, Stats. = statistical analysis, Study Pop. = study population,

Supplementary table 128 – CRP outcomes from observational studies in RA

Table – CRP (RA), results and quality assessment of observational studies

Study (date) [study type]	Results	Weight associated with outcome	AMSTAR2	Study Pop.	Attr.	Prog. Meas.	Outc. Meas.	Conf.	Stats.
Liu (2017) [MA][322]	CRP<br>2/4 studies reported higher CRP in obese compared to non-obese patients	✓	Moderate						
D'Agostino (2017) [Prospective cohort][336] (abatacept)	CRP, change BL-6 mths, mean (SD)<br>BMI <25: -2.0 (0.1)<br>25-30: -1.5 (0.1)<br>≥30 -0.9 (0.1) – statistically significant difference	✓		M	L	L	L	H	M
Tekaya (2011) [Prospective cohort][364]	CRP<br>Obese: 26.96 (31.07)<br>Non obese: 26.21 (32.04) p=0.91	×		L	M	M	L	L	L
George (2017) [Prospective cohort][337]	CRP ≤1 at week 24, OR (95% CI) [adjusted]<br>Obese vs not: 0.44 (0.23, 0.84)	✓		L	L	L	L	L	M
Skoldstam (2005) [Prospective cohort][373]	Univariate OR between reduction and no reduction of body weight with outcome:<br>Dichotomous CRP: 2.85 (p=0.03)	✓		L	L	L	L	H	H

AMSTAR2 = Assessing the Methodological Quality of Systematic Reviews, Attr. = attrition, BL = baseline, BMI = body mass index, CI = confidence interval, Conf. = confounding, CRP = C-reactive protein, L = low risk of bias, M = moderate risk of bias, MA = meta-analysis, OR = odds rtaio, Outc. Meas = outcome measurement, Prog. Meas. = prognostic factor measurement, Pros = prospective, RA = rheumatoid arthritis, Rand. Seq. = random sequence generation, SD = standard deviation, Stats. = statistical analysis, Study Pop. = study population,

Supplementary table 129 – ESR outcomes from observational studies in RA

Table – ESR (RA), results and quality assessment of observational studies

Study (date) [study type]	Results	Weight associated with outcome	AMSTAR2	Study Pop.	Attr.	Prog. Meas.	Outc. Meas.	Conf.	Stats.
Liu (2017) [MA][322]	<u>ESR</u><br>3/4 studies reported higher ESR in obese patients vs non-obese	✓	Moderate						
Skoldstam (2005) [Prospective cohort][373]	<u>Univariate OR between reduction and no reduction of body weight with outcome:</u><br>Dichotomous ESR: 1.64 (p=0.29)	×		L	L	L	L	H	H

AMSTAR2 = Assessing the Methodological Quality of Systematic Reviews, Attr. = attrition, CI = confidence interval, Conf. = confounding, ESR = erythrocyte sedimentation rate, H = high, L = low risk of bias, M = moderate risk of bias, MA = meta-analysis, OR = odds ratio, Outc. Meas = outcome measurement, Prog. Meas. = prognostic factor measurement, Pros = prospective, RA = rheumatoid arthritis, Rand. Seq. = random sequence generation, Stats. = statistical analysis, Study Pop. = study population

Supplementary table 130 – Comorbidity outcomes from observational studies in RA

Table – Comorbidity (RA), results and quality assessment of observational studies

Study (date) [study type]	Results	Weight associated with outcome	AMSTAR2	Study Pop.	Attr.	Prog. Meas.	Outc. Meas.	Conf.	Stats.
Baghdadi (2015) [MA][324]	<u>Cardiovascular morbidity</u><br>Meta-risk ratio: 1.16 (95% CI 1.03, 1.29)	✓	Moderate						
Hirose (2019) [Prospective cohort][326]	<u>MAC-PD, OR (95% CI) [adjusted]</u><br>BMI <18 vs BMI ≥18: 4.24 (1.30, 13.84)	✓		L	L	L	L	M	M
Tantayakom (2016) [Prospective cohort][348]	<u>Metabolic syndrome, OR (95%CI), [adjusted]</u><br>BMI: 1.20 (1.1-1.3)	✓		L	M	L	L	M	M
Kim (2014) [Prospective cohort][352]	<u>Predictors of tuberculosis occurrence, OR (95% CI)</u><br>RA: BMI <22: 1.08 (0.17, 6.87)	✗		L	L	L	L	L	M
Ochi (2014) [Prospective cohort][353]	<u>Distal radial fracture, HR (95% CI) [adjusted]</u><br>BMI continuous: 1.11 (1.03, 1.19)	✓		L	L	L	L	L	M
Dirven (2012) [Prospective cohort][358]	<u>Reporting influenza, OR (95% CI) [adjusted]</u><br>BMI: 1.06 (1.0, 1.1)	✓		L	M	L	L	L	M
Verstappen (2010) [Prospective cohort][366]	<u>OR (95%CI) MTX related adverse events</u><br>Unadjusted: OR 1.074 per unit BMI (0.96, 1.20)<br>Adjusted: OR 1.076 per unit BMI (0.95, 1.21)	✗		L	L	L	L	L	M
Furuya (2009) [Prospective cohort][367]	<u>≥1 fall, OR (95% CI) [adjusted]</u><br>BMI 1.05 (1.02, 1.08)<br><u>≥2 falls, OR (95% CI) [adjusted]</u><br>BMI 1.08 (1.02, 1.15)	✓		L	L	L	L	L	M
Mori (2017) [Retrospective cohort][378]	<u>Hospitalised infection, HR (95% CI) [adjusted]</u><br>BMI <18.5 vs BMI ≥18.5: 2.55 (1.57, 4.14)	✓		L	L	L	L	L	M
Gonzalez (2008) [Retrospective cohort][383]	<u>Combined cardiovascular outcome, HR (95% CI) [adjusted]</u><br>Time-varying high BMI ≥30 vs other BMI: 1.27 (0.93, 1.74)<br>Time-varying low BMI (<20) vs other BMI: 1.58 (1.19, 2.10)	✓		L	L	L	L	L	L
Kent (2004) [Retrospective cohort][384]	<u>Abnormal AST [unadjusted]</u><br>BMI - not significant	✗		M	L	L	L	M	M
Nadareishvili (2008) [Nested case control][83]	<u>First stroke, OR (95% CI)</u><br>BMI continuous: 1.00 (0.95, 1.05)	✗		L	M	L	L	L	M

AMSTAR2 = Assessing the Methodological Quality of Systematic Reviews, Attr. = attrition, AST = aspartate transaminase, BMI = body mas index, = confidence interval, Conf. = confounding, HR = hazard ratio, L = low risk of bias, M = moderate risk of bias, MAC-PD = mycobacterium avium complex pulmonary disease, MTX = methotrexate, OR = odds ratio, Outc. Meas = outcome measurement, Prog. Meas. = prognostic factor measurement, Pros = prospective, RA = rheumatoid arthritis, Rand. Seq. = random sequence generation, SD = standard deviation, Stats. = statistical analysis, Study Pop. = study population

Supplementary table 131 – Drug discontinuation outcomes from observational studies in RA

Table – Drug discontinuation (RA), results and quality assessment of observational studies

Study (date) [study type]	Results	Weight associated with outcome	Study Pop.	Attr.	Prog. Meas.	Outc. Meas.	Conf.	Stats.
Iannone (2017) [Prospective cohort][338] (abatacept)	Abatacept discontinuation, HR (95% CI) [adjusted]<br>Normal weight: ref<br>Overweight: 1.03 (0.89, 1.19)<br>obese: 1.08 (0.89, 1.30)<br>Severely obese: 0.93 (0.72, 1.19)	×	L	H	L	L	L	M
Mariette (2017) [Prospective cohort][341] (abatacept)	Abatacept discontinuation, HR (95% CI) [adjusted]<br>BMI <25: ref<br>BMI 25-30: 0.46 (0.22, 0.99)<br>BMI ≥30: 0.69 (0.34, 1.41)	×	L	L	L	L	L	M
McWilliams (2016) [Prospective cohort][347]	Discontinue TNF at 1 year, OR (95% CI)<br>BMI per category increase: 0.98 (0.93, 1.04	×	L	M	L	L	L	L
Rashid (2016) [Prospective cohort][379]	Switching bDMARD, OR (95% CI) [adjusted]<br>normal BMI: ref<br>overweight: 1.20 (0.81, 1.77)<br>obese: 1.51 (1.04, 2.19)	✓	L	L	L	L	L	M
Iannone (2015) [Prospective cohort][350]	Discontinuing first anti-TNF, HR (95% CI) [adjusted]<br>Normal weight: ref<br>Underweight: 1.22 (0.79, 1.88)<br>Obese: 1.63 (1.02, 2.62)<br>Discontinue second biologic, HR (95% CI) [adjusted]<br>Normal: ref<br>Underweight: 1.56 (0.57, 4.27)<br>Obese: 2.90 (1.0, 8.45)	✓	L	L	L	L	L	L
Verstappen (2010) [Prospective cohort][366]	Withdrawal of MTX due to AEs OR (95% CI)<br>Adjusted: OR 1.207 per unit BMI (1.02, 1.44)	✓	L	L	L	L	L	M
Hoekstra (2003) [Prospective cohort][375]	MTX withdrawal due to toxicity, OR (95% CI) [adjusted]<br>BMI: 1.07 (1.01, 1.14)	✓	L	L	L	L	L	M
Kent (2004) [Retrospective cohort][384]	Discontinuing MTX, mean BMI (SD)<br>Discontinued: BMI 32.1 (6.9)<br>Didn't discontinue: 28.5 (6.0) p<0.03	✓	M	L	L	L	M	M

AEs = adverse events, Attr. = attrition, BMI = body mass index, CI = confidence interval, Conf. = confounding, H = high, HR = hazard ratio, L = low risk of bias, M = moderate risk of bias, MTX = methotrexate, OR = odds ratio, Outc. Meas = outcome measurement, Prog. Meas. = prognostic factor measurement, Pros = prospective, RA = rheumatoid arthritis, Rand. Seq. = random sequence generation, SD = standard deviation, Stats. = statistical analysis, Study Pop. = study population, TNF = tumour necrosis factor

Supplementary table 132 – Mortality outcomes from observational studies in RA

Table – Death (RA), results and quality assessment of observational studies

Study (date) [study type]	Results	Weight associated with outcome	Study Pop.	Attr.	Prog. Meas.	Outc. Meas.	Conf.	Stats.
Baker (2019) [Prospective cohort][325]	<u>Mortality, BMI categories aged 30, HR (95% CI) [adjusted]</u><br>BMI <18.5: 1.01 (0.75, 1.35)<br>BMI 18.5-25: ref<br>BMI 25-30: 1.19 (1.04, 1.36)<br>BMI >30: 2.00 (1.65, 2.42)<br><u>Mortality, BMI categories at baseline, HR (95% CI) [adjusted]</u><br>BMI <18.5: 1.37 (1.00, 1.88)<br>BMI 18.5-25: ref<br>BMI 25-30: 0.99 (0.87, 1.12)<br>BMI >30: 1.34 (1.18, 1.53)	✓	L	L	L	L	L	M
England (2018) [Prospective cohort][328]	<u>Mortality: CVD / cancer / respiratory, time-varying BMI HR (95% CI) [adjusted]</u><br><20: 1.30 (0.34, 5.00) / 1.43 (0.65, 3.13) / 2.93 (1.28, 6.67)<br>20-25: ref<br>>25-30: 0.59 (0.38, 0.91) / 0.87 (0.40, 1.86) / 0.93 (0.57, 1.52)<br>>30: 0.73 (0.31, 1.73) / 0.71 (0.38, 1.33) / 0.50 (0.23, 1.11)<br><u>Mortality: CVD / cancer / respiratory, time-varying weight loss rate HR (95% CI) [adjusted]</u><br>none: ref<br><2: 1.01 (0.77, 1.33) / 0.82 (0.54, 1.25) / 0.85 (0.50, 1.43)<br>2-3: 1.29 (0.68, 2.45) / 1.25 (0.72, 2.17) / 0.92 (0.44, 1.96)<br>>3: 2.27 (1.61, 3.19) / 2.36 (1.11, 5.01) / 1.30 (0.75, 2.26)<br><u>Mortality: CVD / cancer / respiratory, time-varying percentage weight loss rate HR (95% CI) [adjusted]</u><br>none: ref<br><5%: 1.37 (1.04, 1.81) / 0.92 (0.47, 1.83) / 1.18 (0.75, 1.88)<br>5-10%: 1.39 (1.00, 1.94) / 1.30 (0.76, 2.23) / 1.86 (1.07, 3.25)<br>>10%: 2.31 (1.06, 5.01) / 1.90 (1.00, 3.62) / 2.19 (1.30, 3.70)	✓	M	M	L	L	L	M

Attr. = attrition, BMI = body mass index, CI = confidence interval, Conf. = confounding, CVD = cardiovascular disease, L = low risk of bias, HR = hazard ratio, M = moderate risk of bias, Outc. Meas = outcome measurement, Prog. Meas. = prognostic factor measurement, Pros = prospective, RA = rheumatoid arthritis, Rand. Seq. = random sequence generation, Stats. = statistical analysis, Study Pop. = study population

Table – Death (RA), results and quality assessment of observational studies

Study (date) [study type]	Results	Weight associated with outcome	Study Pop.	Attr.	Prog. Meas.	Outc. Meas.	Conf.	Stats.
Sparks (2018) [Prospective cohort][333]	<u>Multivariable HR (95%CI) death:</u><br>Severe loss: 2.78 (1.58, 4.89)<br>Moderate loss: 1.35 (0.76, 2.38)<br>Mild loss: 1.78 (1.25, 2.54)<br>stable=ref<br>Mild gain: 1.21 (0.88, 1.67)<br>Moderate gain: 1.05 (0.63, 1.75)<br>Severe gain: 1.45 (0.69, 3.07)	✓	L	L	L	L	L	M
Baker (2015) [Prospective cohort][349]	<u>Mortality, HR (95% CI), multivariable</u><br>model 1:<br>BMI <20: 3.12 (2.12, 4.57)<br>BMI 20-25: ref<br>BMI >25-30: 0.91 (0.67, 1.23)<br>BMI >30: 0.87 (0.61, 1.24)<br>>1 unit per year change vs <1: 1.99 (1.53, 2.59)<br>Model 2:<br>BMI <20: 2.31 (1.50, 3.57)<br>BMI 20-25: ref<br>BMI >25-30: 0.92 (0.66, 1.29)<br>BMI >30: 0.74 (0.49, 1.11)<br>>1 unit per year change vs <1: 1.81 (1.36, 2.41)<br><u>Mortality, rate of change, HR (95% CI)</u><br>no weight loss: ref<br>0-<2 per year: 1.12 (0.85, 1.49)<br>2-3 per year: 1.65 (1.09, 2.50)<br>>3 per year: 2.49 (1.73, 3.57)	✓	M	L	L	L	L	M
Wolfe (2012) [Prospective cohort][360]	<u>All-cause mortality for all ages (RR (95%CI) [adjusted]</u><br>Underweight = 1.9 (1.7, 2.3)<br>Normal = reference<br>Overweight = 0.8 (0.8, 0.9)<br>Obese = 0.8 (0.7, 0.8)<br><u>All-cause mortality, <50 / 50-70 / >70 years, RR (95% CI) [adjusted]</u><br>Underweight: 1.6 (0.7, 3.6) / 2.2 (1.7, 2.9) / 1.6 (1.3, 2.0)<br>Normal: ref<br>Overweight: 0.8 (0.5, 1.2) / 0.8 (0.8, 1.0) / 0.8 (0.7, 0.9)<br>Obese: 1.6 (1.1, 2.2) / 1.0 (0.9, 1.8) / 0.9 (0.7, 1.0)	✓	L	L	L	L	L	M

Model 1 includes age, female sex, white race, current body mass index (BMI) category, weight loss over the previous interval (versus no weight loss), use of methotrexate, prednisone, or tumor necrosis factor (TNF) inhibitor, presence of diabetes, cardiovascular disease, chronic kidney disease, or malignancy, and active smoking Model 2 includes the variables in model 1 but with the addition of the natural log–transformed C-reactive protein (CRP) level and the Multidimensional Health Assessment Questionnaire (MDHAQ)

Attr. = attrition, BMI = body mass index, CI = confidence interval, Conf. = confounding, HR = hazard ratio, L = low risk of bias, M = moderate risk of bias, Outc. Meas = outcome measurement, Prog. Meas. = prognostic factor measurement, Pros = prospective, RA = rheumatoid arthritis, Rand. Seq. = random sequence generation, RR = risk ratio, SD = standard deviation, Stats. = statistical analysis, Study Pop. = study population

Table – Death (RA), results and quality assessment of observational studies

Study (date) [study type]	Results	Weight associated with outcome	Study Pop.	Attr.	Prog. Meas.	Outc. Meas.	Conf.	Stats.
Escalante (2005) [Prospective cohort][371]	Mortality rate per 100 person years,(95% CI) [adjusted]<br><20: 6.8 (4.2, 11.1)<br>20 to <25: 3.3 (2.3, 4.9)<br>25 to <30: 2.1 (1.4, 3.0)<br>≥30: 1.4 (0.1, 2.2)<br>Mortality, HR (95% CI) [adjusted]<br>BMI HR 0.97 (0.94, 1.00)	✓	L	L	L	L	L	M
Maradit-Kremers (2005) [Prospective cohort][372]	Cardiovascular death, HR (95% CI) [adjusted]<br>BMI ≥30: 0.93 (0.60, 1.45)<br>BMI <20: 1.80 (1.27, 2.54)	✓	L	L	L	L	L	M
Maradit-Kremers (2004) [Prospective cohort][374]	Cardiovascular mortality, HR (95% CI) [adjusted]<br>Normal BMI BL - normal FU: ref<br>Low BMI BL - low/normal FU: 3.06 (1.99, 4.69)<br>Normal BMI BL - low BMI FU: 2.09 (1.50, 2.92)<br>Normal BMI BL - high BMI FU: 1.33 (0.87, 2.02)<br>High BMI BL - high / normal BMI FU: 0.95 (0.57, 1.61)	✓	L	L	L	L	L	L

Attr. = attrition, BL = baseline, BMI = body mass index, CI = confidence interval, Conf. = confounding, FU = follow-up, HR = hazard ratio, L = low risk of bias, M = moderate risk of bias, Outc. Meas = outcome measurement, Prog. Meas. = prognostic factor measurement, RA = rheumatoid arthritis, SD = standard deviation, Stats. = statistical analysis, Study Pop. = study population

Supplementary table 133 – Description of studies of assessing weight and outcomes in SLE

Table – Systemic Lupus Erythematosus, description of included studies

Author (date) [country]	Study design	Inclusion criteria	Exposure detail	N	Age, mean (SD) years	N (%) female	Funders
Figueiredo-Braga (2018) [Portugal][385]	Pros. Cohort	ACR SLE criteria	BMI – continuous	70	44.31 (9.9)	70 (100)	**Government** (Fulbright commission: Portugal)
Jacobs (2013) [The Netherlands][386]	Pros. Cohort	ACR SLE criteria	BMI – continuous	126	39.0 (12.2)	89.7%	**Not reported –** Authors declared no conflict of interest
Katz (2011) [USA][387]	Pros. Cohort	University of California at San Francisco (UCSF) Lupus Outcomes Study (LOS) - SLE diagnosis confirmed by medical record review<br>Exclusions: underweight <18.5 BMI	BMI categories:<br>obesity = 1) BMI ≥30 and 2) BMI 26.8 kg/m2 , a revised obesity criterion recently proposed for women with SLE based on data regarding body composition from DXA analyses of a subset of these women. This lower criterion was found to correspond better with the percentage of body fat associated with threshold of obesity than a BMI of 30 kg/m2	716	48.1 (12.6)	716 (100)	**Government** (NIH)
Chaiamnuay (2007) [USA][388]	Pros. Cohort	ACR SLE criteria, symptom duration ≤5 years	BMI – continuous	614	37.2 (12.9)	90%	**Government** (National Institute of Arthritis and Musculoskeletal and Skin Diseases, National Center for Research Resources), **University** (University of Alabama Birmingham), **Industry** (Rheuminations, Inc)
Chaiamnuay (2007) [USA][389]	Pros. Cohort	LUMINA study – ACR SLE criteria, symptom duration <5 years	BMI – continuous	488	34.9 (11.7)	90%	**Government** (National Institute of Arthritis and Musculoskeletal and Skin Diseases, National Center for Research Resources), **University** (University of Alabama Birmingham), **Industry** (Rheuminations, Inc)
Uaratanawong (2004) [Thailand][390]	Pros. Cohort	Pre-monpause, prednisolone treated SLE	BMI	106	31.7 (7.5)	106 (100)	**Not reported**

ACR = American College of Rheumatology, BMI = body mass index, N = number, NIH = National instates of Health, pros. = prospective, Retro. = retrospective, SD = standard deviation, SLE = systemic lupus erythematosus, USA = United States of America

Table – Systemic Lupus Erythematosus, description of included studies

Author (date) [country]	Study design	Inclusion criteria	Exposure detail	N	Age, mean (SD) years	N (%) female	Funders
Bruce (1998) [Canada][391]	Pros. Cohort	University of Toronto Lupus Database	BMI categories:<br>Obese<br>Non-obese	24	50 (12.2) at first event	18 (75.0)	**Charity** (Ontario Lupus Association, Arthritis Society Canada)
Petri (1992) [USA][392]	Pros. Cohort	John Hopkins Lupus Cohort, clinical diagnosis of SLE	BMI categories:<br>Obese= >27.8 in men and >27.3 in women	229	CAD+:<br>47.1 (11.3)<br>CAD-:<br>34.7 (11.2)	209 (91.3)	**Government** (NIH), **University (**John Hopkins),

BMI = body mass index, N = number, NIH = National instates of Health, pros. = prospective, SD = standard deviation, SLE = systemic lupus erythematosus, USA = United States of America

Supplementary table 134 – Function outcomes from observational studies in SLE

Table – Function (SLE), results and quality assessment of observational studies

Study (date) [study type]	Results	Weight associated with outcome	Study Pop.	Attr.	Prog. Meas.	Outc. Meas.	Conf.	Stats.
Katz (2011) [Prospective cohort][387]	VLA disability, mean (SD), regression coefficient (p value)<br>BMI ≥30: 1.03 (0.59)<br>BMI <30: 0.67 (0.62) p<0.0001<br>Regression coefficient : 0.10 (0.01) [adjusted]<br>BMI ≥26.8: 0.95 (0.63)<br>BMI <26.8: 0.65 (0.61) p<0.0001<br>Regression coefficient: 0.04 (0.32) [adjusted]	✓	L	M	L	L	L	M

Attr. = attrition, BMI = body mass index, CI = confidence interval, Conf. = confounding, L = low risk of bias, M = moderate risk of bias, Outc. Meas = outcome measurement, Prog. Meas. = prognostic factor measurement, Pros = prospective, SD = standard deviation, SLE = systemic lupus erythematosus, Stats. = statistical analysis, Study Pop. = study population, VLA = valued life activities.

Supplementary table 135 – Disease activity outcomes from observational studies in SLE

Table – Disease activity (SLE), results and quality assessment of observational studies

Study (date) [study type]	Results	Weight associated with outcome	Study Pop.	Attr.	Prog. Meas.	Outc. Meas.	Conf.	Stats.
Chaiamnuay (2007) [USA][388]	<u>SLAM-R, BL BMI predicting average over follow-up</u><br>Unadjusted: r 0.095, p=0.072<br>Adjusted: t 1.093, p=0.203	×	L	H	M	L	L	M

Attr. = attrition, BMI = body mass index, CI = confidence interval, Conf. = confounding, H = high, L = low risk of bias, M = moderate risk of bias, Outc. Meas = outcome measurement, Prog. Meas. = prognostic factor measurement, Pros = prospective, SD = standard deviation, SLAM-R = Systemic Lupus Activity Measure – revised), SLE = systemic lupus erythematosus, Stats. = statistical analysis, Study Pop. = study population

Supplementary table 136 – Fatigue outcomes from observational studies in SLE

Table – Fatigue (SLE), results and quality assessment of observational studies

Study (date) [study type]	Results	Weight associated with outcome	Study Pop.	Attr.	Prog. Meas.	Outc. Meas.	Conf.	Stats.
Chaiamnuay (2007) [USA][388]	<u>Fatigue severity scale, BL BMI predicting average over follow-up</u><br>Unadjusted: r 0.155 p=0.003<br>Adjusted: t 1.231 p=0.219	×	L	H	M	L	L	M

Attr. = attrition, BL = baseline, BMI = body mass index, CI = confidence interval, Conf. = confounding, H = high,L = low risk of bias, M = moderate risk of bias, Outc. Meas = outcome measurement, Prog. Meas. = prognostic factor measurement, Pros = prospective, SLE = systemic lupus erythematosus, Stats. = statistical analysis, Study Pop. = study population, USA = United States of America

Supplementary table 137 – Damage outcomes from observational studies in SLE

Table – Damage (SLE), results and quality assessment of observational studies

Study (date) [study type]	Results	Weight associated with outcome	Study Pop.	Attr.	Prog. Meas.	Outc. Meas.	Conf.	Stats.
Chaiamnuay (2007) [USA][388]	SLICC Damage Index, BL BMI predicting average over follow-up<br>Unadjusted: r 0.035, p=0.540<br>Adjusted: na	×	L	H	M	L	L	M

Attr. = attrition, BL = baseline, BMI = body mass index, CI = confidence interval, Conf. = confounding, H = high, L = low risk of bias, M = moderate risk of bias, Outc. Meas = outcome measurement, Prog. Meas. = prognostic factor measurement, Pros = prospective, SLE = systemic lupus erythematosus, SLICC = Systemic Lupus Erythematosus International Collaborating Clinics Group, Stats. = statistical analysis, Study Pop. = study population, USA = United States of America

Supplementary table 138 – Mental health outcomes from observational studies in SLE

Table – Mental health (SLE), results and quality assessment of observational studies

Study (date) [study type]	Results	Weight associated with outcome	Study Pop.	Attr.	Prog. Meas.	Outc. Meas.	Conf.	Stats.
Figueiredo-Braga (2018) [Prospective cohort][385]	HADS depression >8 after 1 month, OR (95% CI)<br>BMI 1.01 (1.00, 1.02)	✓	L	M	M	L	L	M

Attr. = attrition, BMI = body mass index, CI = confidence interval, Conf. = confounding, HADS = Hospital Anxiety and Depression Scale, L = low risk of bias, M = moderate risk of bias, OR = odss ratio, Outc. Meas = outcome measurement, Prog. Meas. = prognostic factor measurement, Pros = prospective, SLE = systemic lupus erythematosus, Stats. = statistical analysis, Study Pop. = study population

Supplementary table 139 – Bone mineral density outcomes from observational studies in SLE

Table – Bone mineral density (SLE), results and quality assessment of observational studies

Study (date) [study type]	Results	Weight associated with outcome	Study Pop.	Attr.	Prog. Meas.	Outc. Meas.	Conf.	Stats.
Jacobs (2013) [Prospective cohort][386]	<u>Hip BMD, regression coefficient (p value) [unadjusted]</u><br>BMI change: 0.075 (0.103)<br><u>lumbar spine</u><br>BMI non-significant	×	L	L	L	L	M	M
Uaratanawong (2004) [Prospective cohort][390]	BMI not associated with change in BMD [unadjusted]	×	L	L	L	L	H	H

Attr. = attrition, BMD = bone mineral density, BMI = body mass index, CI = confidence interval, Conf. = confounding, H = high, L = low risk of bias, M = moderate risk of bias, Outc. Meas = outcome measurement, Prog. Meas. = prognostic factor measurement, Pros = prospective, SD = standard deviation, SLE = systemic lupus erythematosus, Stats. = statistical analysis, Study Pop. = study population

Supplementary table 139 – Comorbidity outcomes from observational studies in SLE

Table – Comorbidity (SLE), results and quality assessment of observational studies

Study (date) [study type]	Results	Weight associated with outcome	Study Pop.	Attr.	Prog. Meas.	Outc. Meas.	Conf.	Stats.
Chaiamnuay (2007) [Prospective cohort][389]	<u>Hypertension, OR (95% CI)</u><br>BMI, per unit: OR 1.060 (1.009, 1.114)	✓	L	L	L	L	L	M
Bruce (1998) [Prospective cohort][391]	79% of the patients with a cardiovascular event were obese	✓	L	H	M	L	H	H
Petri (1992) [Prospective cohort][392]	<u>Developing CAD, OR (95% C)</u><br>obese: OR 2.1 (0.8, 5.6) [univariate]<br>obese: beta 1.23089, SE0.67838	✓	L	M	M	L	M	H

Attr. = attrition, BMI = body mass index, CAD = coronary artery disease, CI = confidence interval, Conf. = confounding, H= high, L = low risk of bias, M = moderate risk of bias, OR = odds ratio, Outc. Meas = outcome measurement, Prog. Meas. = prognostic factor measurement, Pros = prospective, Rand. Seq. = random sequence generation, SE = standard error, SLE = systemic lupus erythematosus, Stats. = statistical analysis, Study Pop. = study population

Supplementary table 139 – Work outcomes from observational studies in SLE

Table – Work (SLE), results and quality assessment of observational studies

Study (date) [study type]	Results	Weight associated with outcome	Study Pop.	Attr.	Prog. Meas.	Outc. Meas.	Conf.	Stats.
Katz (2011) [Prospective cohort][387]	Employed yes/no, OR (95% CI)<br>BMI ≥30 vs <30: 0.6 (0.3, 0.9) [adjusted]<br>BMI ≥26.8 vs <26.8: 0.5 (0.3, 0.8) [adjusted]	✓	L	M	L	L	L	M

Attr. = attrition, BMI = body mass index, CI = confidence interval, Conf. = confounding, L = low risk of bias, M = moderate risk of bias, OR = odds ratio, Outc. Meas = outcome measurement, Prog. Meas. = prognostic factor measurement, Pros = prospective, SLE = systemic lupus erythematosus, Stats. = statistical analysis, Study Pop. = study population

Supplementary table 140 – Description of studies of assessing weight and outcomes in axSpA

Table – Axial Spondyloarthritis (axSpA), description of included studies

Author (date) [country]	Study design	Inclusion criteria	Exposure detail	N	Age, mean (SD) years	N (%) female	Funders
Hernandez-Breijo (2019) [Spain / The Netherlands][393]	Pros. Cohort	axSpA according to ASAS and start INF or ADA	BMI categories:<br>BMI ≤25<br>BMI >25	180	47.0 (12.7)	73 (40.5)	**No funding**
Jeong (2018) [S. Korea][394]	Pros. Cohort	Modified New York criteria, aged >18 years, Baseline + Follow-up ≥2 years radiographs	BMI – continuous	151	25.3 (10.2)	17 (11.3)	**Not reported** – authors declared no conflict of interest
Pedersen (2018) [Denmark][395]	Pros. Cohort	BIOSPA - starting anti-TNF, European spondyloarthropathy Study Group (ESSG) classification criteria for spondyloarthritis, BASDAI >3 despite NSAID treatment, clinical indication for anti-TNF, fulfil radiographic section of modified New York criteria, have inflammation and/or structural lesions on MRI	BMI categories:<br>Normal weight: 18.5-24.9<br>Overweight-obese: ≥25	33	40.3 (10.9)	7 (21.2)	**No funding**
Maas (2017) [The Netherlands][396]	Pros. Cohort	Groningen Leeuwarden AS (GLAS) cohort - started anti-TNF treatment, available radiographs at baseline and after 6 years, ≥18 years, modified New York Criteria	BMI – continuous<br>BMI categories:<br>≥25<br><25	80	41.3 (10.5)	24 (30.0)	**Industry** (Pfizer)
Maas (2017) [The Netherlands][397]	Pros. Cohort	Groningen Leeuwarden AS (GLAS) cohort - radiographs available at baseline and 2 years, starting anti-TNF	BMI – continuous	292	42.8 (12.5)	87 (29.8)	**Industry** (Pfizer)
Micheroli (2017) [Switzerland][398]	Pros. Cohort	Swiss Clinical Quality Management cohort - ASAS AXSPA patients, starting anti-TNF exclusions: concurrent fibromyalgia, BMI <18.5	BMI categories:<br>Normal: 18.5 - <25<br>Overweight: 25-30<br>Obese: >30	624	39.4 (11.6)	37.8%	**Professional body** (Swiss Society of Rheumatology Swiss Balgrist Society), **Industry** (AbbVie, Bristol-Myers-Squibb, Janssen-Cilag, Merck Sharp & Dohme, Novartis, Pfizer, Roche, UCB), **Charity** (Arco Foundation)
Hwang (2017) [S. Korea][399]	Pros. Cohort	ASAS axial SpA, AS patients met modified New York criteria, all treated with either adalimumab or infliximab	BMI categories:<br>BMI ≥25	100	Adalimumab: 34.9 (9.6)<br>Infliximab: 34.8 (11.7)	Adalimumab: 8.3%<br>Infliximab: 17.9%	**Government** (Ministry of Health & Welfare, Republic of Korea)

ACR = American College of Rheumatology, ADA = adalimumab, AS = Ankylosing spondylitis, ASAS = Assessment of SpondyloArthritis international Society, axSpA = axial spondyloarthritis, BASDAI = Bath Ankylosing Spondylitis Disease Activity Index, BMI = body mass index, INF = infliximab, N = number, NSAID = non-steroidal anti-inflammatory drug, pros. = prospective, SD = standard deviation, TNFi = tumour necrosis factor inhibitor

Table – Axial Spondyloarthritis (axSpA), description of included studies

Author (date) [country]	Study design	Inclusion criteria	Exposure detail	N	Age, mean (SD) years	N (%) female	Funders
van Weely (2016) [The Netherlands][400]	Pros. Cohort	AS (modified New York criteria) patients, aged ≥18 years, TNFi-naïve and eligible for TNFi treatment, Dutch speaking	BMI – continuous	257	43.3 (11.5)	84 (32.7)	**Not reported**
Maas (2015) [The Netherlands][401]	Pros. Cohort	Groningen Leeuwarden AS (GLAS) cohort - aged >18 years, modified New York criteria, starting anti-TNF	BMI – continuous	176	42.3 (11.1)	55 (31.3)	**Industry** (Pfizer)
Gremese (2014) [Italy][402]	Pros. Cohort	ASAS criteria for Axial SpA, treated with anti-TNF, axial involvement, naïve to previous TNFi, active disease (BASDAI ≥4 despite 3 months of NSAIDs)	BMI categories:<br><25 = normal<br>25-30 = overweight<br>>30 = obese	170	39.5 (11.8)	52 (30.6)	**Charity** (ASRALES ONLUS Foundation)
Kim (2014) [S. Korea][352]	Pros. Cohort	Starting anti-TNF, modified New York criteria	BMI categories:<br>BMI <22	336	36.6 (12.3)	64 (19.0)	**Not reported –** Authors declared no conflict of interest
Ottaviani (2012) [France][403]	Pros. Cohort	European Spondyloarthropathy Study Group AS criteria, starting infliximab	BMI – continuous	155	Median (IQR): 43.1 (35.0, 51.8)	57 (36.8)	**Not reported** – authors declared no conflicts of interest
Ward (2002) [USA][187]	Pros. Cohort	New York criteria, aged >18 years, no inflammatory bowel disease<br>exclusion: inflammatory bowel disease	BMI – continuous	212	47.8 (13.6)	63 (29.7)	**Charity** (Bartman Foundation)

ACR = American College of Rheumatology, AS = Ankylosing spondylitis, ASAS = Assessment of SpondyloArthritis international Society, axSpA = axial spondyloarthritis, BASDAI = Bath Ankylosing Spondylitis Disease Activity Index, BMI = body mass index, DMARD = disease modifying anti-rheumatic drug, IQR = interquartile range, N = number, NSAID = non-steroidal anti-inflammatory drugs, pros. = prospective, SD = standard deviation, TNFi = tumour necrosis factor inhibitor, USA = United States of America

Supplementary table 141 – Pain outcomes from observational studies in axSpA

Table – Pain (axSpA), results and quality assessment of observational studies

Study (date) [study type]	Results	Weight associated with outcome	Study Pop.	Attr.	Prog. Meas.	Outc. Meas.	Conf.	Stats.
Ottaviani (2012) [Prospective cohort][403] (Infliximab)	Pain VAS50 at 6 months, OR (95% CI)<br>BMI: 0.87 (0.80, 0.93)	✓	L	L	L	L	M	M

Attr. = attrition, axSpA = axial spondyloarthritis, BMI = body mass index, CI = confidence interval, Conf. = confounding, L = low risk of bias, M = moderate risk of bias, OR = odds ratio, Outc. Meas = outcome measurement, Prog. Meas. = prognostic factor measurement, Pros = prospective, Rand. Seq. = random sequence generation, SD = standard deviation, Stats. = statistical analysis, Study Pop. = study population, VAS = visual analogue scale

Supplementary table 142 – Function outcomes from observational studies in axSpA

Table – Function (axSpA), results and quality assessment of observational studies

Study (date) [study type]	Results	Weight associated with outcome	Study Pop.	Attr.	Prog. Meas.	Outc. Meas.	Conf.	Stats.
van Weely (2016) [Prospective cohort][400]	Mixed model, multivariable model change (95%CI):<br>BASFI 0-6 months: BMI not included<br>BASFI 6-36 months: BMI = -0.029 (-0.078, 0.020),	×	L	L	L	L	M	M
Ward (2002) [Prospective cohort][187]	Change in slope HAQ score over time:<br>Univariate: BMI 0.0059 (-0.0003, 0.0122) p=0.07<br>Multivariable: BMI not included in final model	×	L	M	L	L	H	H

Attr. = attrition, axSpA = axial spondyloarthritis, BASFI = Bath Ankylosing Spondylitis Functional Index, BMI = body mass index, CI = confidence interval, Conf. = confounding, H = high, HAQ = Health Assessment Questionnaire, L = low risk of bias, M = moderate risk of bias, Outc. Meas = outcome measurement, Prog. Meas. = prognostic factor measurement, Pros = prospective, Stats. = statistical analysis, Study Pop. = study population

Supplementary table 143 – Disease activity outcomes from observational studies in axSpA

Table – Disease activity (axSpA), results and quality assessment of observational studies

Study (date) [study type]	Results	Weight associated with outcome	Study Pop.	Attr.	Prog. Meas.	Outc. Meas.	Conf.	Stats.
Hernandez-Breijo (2019) [Prospective cohort][393]	<u>Multivariable Logistic regression, OR (95%CI), ref= no csDMARDs: clinical response</u><br>BMI≤25 (n=60):<br>any csDMARD = 1.10 (0.33-3.58)<br>MTX/ +/- SSZ = 1.04 (0.25 - 4.25)<br>SSZ = 1.18 (0.25-5.63)<br>BMI>25 (n=81):<br>any csDMARD = 7.86 (2.39, 25.78)<br>MTX/ +/- SSZ = 9.82 (2.13-45.20)<br>SSZ = 6.86 (1.85-25.40)<br><u>remission</u><br>BMI≤25<br>any csDMARD = 0.76 (0.20, 2.86)<br>MTX +/- SSZ = 0.60 (0.11, 3.18)<br>SSZ = 0.99 (0.17, 5.64)<br>BMI>25<br>any csDMARD = 4.84 (1.09, 21.39)<br>MTX +/- SSZ = 5.56 (0.84, 36.52)<br>SSZ = 4.35 (0.77, 24.54)<br>[Concomitant DMARDs with TNF inhibitor improves chances of response and remission in overweight patients but not in normal weight patients]	✓	M	M	M	L	L	M
Micheroli (2017) [Prospective cohort][398]	<u>BASDAI, baseline / 1 year, mean(SD) [adjusted]</u><br>normal: 5.3 (2.0) / 2.9 (2.2)<br>overweight: 5.6 (1.9) / 3.2 (2.2)<br>obese: 6.1 (1.7) / 4.1 (2.4)	✓	L	M	L	L	L	M
Ottaviani (2012) [Prospective cohort][403] (infliximab)	<u>BASDAI50 at 6 months, OR (95% CI)</u><br>BMI: 0.87 (0.81, 0.94)	✓	L	L	L	L	M	M

Attr. = attrition, axSpA = axial spondyloarthritis, BASDAI = Bath Ankylosing Spondylitis Disease Activity Index, BMI = body mass index, CI = confidence interval, Conf. = confounding, csDAMRD = conventional synthetic disease modifying anti-rheumatic drug, L = low risk of bias, M = moderate risk of bias, MTX = methotrexate, OR = odds ratio, Outc. Meas = outcome measurement, Prog. Meas. = prognostic factor measurement, Pros = prospective, SD = standard deviation, SSZ = sulfasalazine, Stats. = statistical analysis, Study Pop. = study population

Supplementary table 144 – Fatigue outcomes from observational studies in axSpA

Table – Fatigue (axSpA), results and quality assessment of observational studies

Study (date) [study type]	Results	Weight associated with outcome	Study Pop.	Attr.	Prog. Meas.	Outc. Meas.	Conf.	Stats.
Micheroli (2017) [Prospective cohort][398]	BASDAI fatigue, 1 year, mean(SD)<br>Normal: 4.0 (2.7)<br>Overweight: 4.1 (2.6)<br>Obese: 5.0 (2.4)	✓	L	M	L	L	L	M

Attr. = attrition, axSpA = axial spondyloarthritis, BASDAI = Bath Ankylosing Spondylitis Disease Activity Index, CI = confidence interval, Conf. = confounding, L = low risk of bias, M = moderate risk of bias, Outc. Meas = outcome measurement, Prog. Meas. = prognostic factor measurement, Pros = prospective, SD = standard deviation, Stats. = statistical analysis, Study Pop. = study population

Supplementary table 145 – Radiographic progression outcomes from observational studies in axSpA

Table – Radiographic progression (axSpA), results and quality assessment of observational studies

Study (date) [study type]	Results	Weight associated with outcome	Study Pop.	Attr.	Prog. Meas.	Outc. Meas.	Conf.	Stats.
Jeong (2018) [Prospective cohort][394]	Spinal radiographic progression, Regression (p value) [adjusted]<br>BMI continuous: 0.045 (SE=0.021) (p=0.039)	✓	L	L	L	L	L	M
Pedersen (2018) [Prospective cohort][395]	Progression in spine mSASSS score, OR (95% CI)<br>BMI normal vs overweight: 0.57 (0.11, 3.04) [unadjusted]<br>Progression in spine New bone formation score, OR (95% CI)<br>BMI normal vs overweight: 2.86 (0.53, 15.47) [unadjusted]<br>Progression in sacrolitic joint, OR (95% CI)<br>BMI normal vs overweight: 0.28 (0.06, 1.41) [unadjusted]	×	L	M	L	L	M	M
Maas (2017) [Prospective cohort][396]	Spinal radiographic progression over time (GEE), regression coefficient (95% CI)<br>Baseline BMI continuous: 1.53 (0.41, 2.64)<br>Baseline ≥25 BMI vs <25: 12.62 (4.85, 20.40)	✓	L	L	L	L	L	L
Maas (2015) [Prospective cohort][401]	Spinal radiographic progression over time (GEE), regression coefficient (95% CI)<br>Longitudinal BMI: -0.02 (-0.09, 0.05)	×	L	M	L	L	L	M

Attr. = attrition, axSpA = axial spondyloarthritis, BL = baseline, BMI = body mass index, CI = confidence interval, Conf. = confounding, GEE = Generalised Estimating Equations, L = low risk of bias, M = moderate risk of bias, mSASSS, modified Stoke Ankylosing Spondylitis Spinal Score, OR = odds ratio, Outc. Meas = outcome measurement, Prog. Meas. = prognostic factor measurement, Pros = prospective, SE = standard error, Stats. = statistical analysis, Study Pop. = study population,

Supplementary table 146 – CRP outcomes from observational studies in axSpA

Table – CRP (axSpA), results and quality assessment of observational studies

Study (date) [study type]	Results	Weight associated with outcome	Study Pop.	Attr.	Prog. Meas.	Outc. Meas.	Conf.	Stats.
Ottaviani (2012) [Prospective cohort][403]	CRP50 at 6 months, OR (95% CI)<br>BMI: 0.93 (0.88, 0.99)	✓	L	L	L	L	M	M

Attr. = attrition, axSpA = axial spondyloarthritis, BMI = body mass index, CI = confidence interval, Conf. = confounding, CRP = C-reactive protein, L = low risk of bias, M = moderate risk of bias, OR = odds ratio, Outc. Meas = outcome measurement, Prog. Meas. = prognostic factor measurement, Pros = prospective, Stats. = statistical analysis, Study Pop. = study population

Supplementary table 147 – Response criteria outcomes from observational studies in axSpA

Table – Response criteria (axSpA), results and quality assessment of observational studies

Study (date) [study type]	Results	Weight associated with outcome	Study Pop.	Attr.	Prog. Meas.	Outc. Meas.	Conf.	Stats.
Micheroli (2017) [Prospective cohort][398]	ASAS40 at 1 year, OR (95% CI)<br>normal: ref<br>overweight: 0.66 (0.34, 1.30) [adjusted]<br>obese: 0.18 (0.05, 0.59) [adjusted]	✓	L	M	L	L	L	M
Gremese (2014) [Prospective cohort][402]	BASDAI50 Poor response at 12 months, OR (95% CI)<br>BMI <25: 0.41 (0.19, 0.86) vs BMI 25-30<br>BMI ≥30: 3.57 (1.15, 11.11) vs BMI 25-30	✓	L	L	L	L	L	M

ASAS40 = Assessment in SpondyloArthritis International Society 40% Response, Attr. = attrition, axSpA = axial spondyloarthritis, BASDAI = Bath Ankylosing Spondylitis Disease Activity Index, BMI = body mass index, CI = confidence interval, Conf. = confounding, L = low risk of bias, M = moderate risk of bias, OR = odds ratio, Outc. Meas = outcome measurement, Prog. Meas. = prognostic factor measurement, Pros = prospective, SD = standard deviation, Stats. = statistical analysis, Study Pop. = study population,

Supplementary table 148 – Comorbidity outcomes from observational studies in axSpA

Table – Comorbidity (axSpA), results and quality assessment of observational studies

Study (date) [study type]	Results	Weight associated with outcome	Study Pop.	Attr.	Prog. Meas.	Outc. Meas.	Conf.	Stats.
Maas (2017) [Prospective cohort][397]	Vertebral fracture at 2 years, mean (SD) BL BMI [unadjusted]<br>Present fracture: 27.7 (4.3)<br>Absent fracture: 26.2 (4.6) p=0.040	✓	L	M	L	L	H	H
Kim (2014) [Prospective cohort][352]	Predictors of tuberculosis occurrence, OR (95% CI) [adjusted]<br>BMI <22 vs BMI ≥22: OR 13.0 (1.51, 111.92)	✓	L	L	L	L	L	M

Attr. = attrition, axSpA = axial spondyloarthritis, BL = baseline, BMI = body mass index, CI = confidence interval, Conf. = confounding, H = high, L = low risk of bias, M = moderate risk of bias, OR = odds ratio, Outc. Meas = outcome measurement, Prog. Meas. = prognostic factor measurement, Pros = prospective, Rand. Seq. = random sequence generation, SD = standard deviation, Stats. = statistical analysis, Study Pop. = study population

Supplementary table 149 – Drug discontinuation outcomes from observational studies in axSpA

Table – Drug discontinuation (axSpA), results and quality assessment of observational studies

Study (date) [study type]	Results	Weight associated with outcome	Study Pop.	Attr.	Prog. Meas.	Outc. Meas.	Conf.	Stats.
Micheroli (2017) [Prospective cohort][398]	TNFi discontinuation, HR (95% CI) [adjusted]<br>normal: ref<br>overweight: 0.98 (0.79, 1.38)<br>obese: 1.01 (0.63, 1.65)	×	L	M	L	L	L	M
Hwang (2016) [Prospective cohort][399]	TNFi discontinuation, OR (95% CI)<br>BMI ≥25: OR 4.35 (1.01, 18.69)	✓	M	L	L	M	H	H

Attr. = attrition, axSpA = axial spondyloarthritis, BMI = body mass index, CI = confidence interval, Conf. = confounding, HR = hazard ratio, L = low risk of bias, M = moderate risk of bias, OR = odds ratio, Outc. Meas = outcome measurement, Prog. Meas. = prognostic factor measurement, Pros = prospective, Stats. = statistical analysis, Study Pop. = study population, TNFi = tumour necrosis factor inhibitor

Supplementary table 150 – Description of studies of assessing weight and outcomes in PsA

Table – Psoriatic arthritis, description of reviews

Authors (date)	Review type	Study type included	Exposure detail	Number of studies included	Funders
Lupoli (2016)[323]	MA	Observational studies	Studies investigating the association between obesity and minimal disease activity in PsA	7	**Government** (Italian Ministry of Health)

MA = meta-analysis, PsA = psoriatic arthritis

Table – Psoriatic arthritis, description of included studies

Author (date) [country]	Study design	Inclusion criteria	Exposure detail	N	Age, mean (SD) years	N (%) female	Funders
Di Minno (2014) [Italy][404]	RCT	CASPAR criteria PsA, referred to start anti-TNF<br>Exclusion: aged <18 years, previous treatment with TNFα blockers, current treatment with corticosteroids, history of arterial or venous thrombosis, malignancy, haematological/oncological diseases, autoimmune diseases other than PsA, unstable medical conditions, ongoing pregnancy.	overweight = BMI 25-30,<br>obese = BMI >30 and/or waist circumference >102cm for men and >88cm for women<br>1) Hypocaloric diet - designed to produce a caloric restriction of about 30% of total energy requirements, restriction of calorie intake to <1500 kcal/day, restriction of fat intake to 30–35%, avoidance of trans fats, high-fibre uptake<br>p) Free self-managed diet	1) 63<br>p) 63	1) 46.8 (10.4)<br>p) 43.5 (12.4)	1) 40 (63.5)<br>p) 40 (63.5)	**Not reported** – authors declared no conflict of interest
Klingberg (2019) [Sweden][405]	Single arm int.	CASPAR criteria, BMI ≥33, aged 25-75 years, consistent meds for 3 months<br>Exclusions: pregnancy, porphyria, epilepsy, type 1 diabetes, severe heart, kidney or catabolic disease, binge eating disorders, treatment with warfarin, lithionin or phenantoin, mental imbalance affecting participation, being subject to a heart infarction, stroke, major surgery or trauma during last the 3 months and being treated for cancer during the last 5 years	Calorie restriction 640 cal / day, four daily portions of powder dissolved in cold or hot water and consumed as shakes or soups for 12 weeks. After 12 weeks, food gradually reintroduced.	41	Median (IQR): 54 (48.5, 62)	26 (63.4)	**Government** (Swedish Government), **Professional body** (Gothenburg Society of Medicine, Swedish Rheumatology Association), **Charity** (Inger Bendix foundation, Rune and Ulla Amlövs foundation, Stiftelsen Psoriasisfonden), **Industry** (Roche)
Polachek (2017) [Canada][406]	Pros. Cohort	University of Toronto PsA cohort - fulfilled CASPAR criteria	BMI – continuous	803	50.8 (13.4)	43%	**Charity** (Krembil Foundation), **Industry** (Janssen Canada)
Hojgaard (2016) [Denmark, Iceland][407]	Pros. Cohort	DANBIO & ICEBIO registers<br>Exclusions: participation in clinical trials, erroneous baseline information, patients treated with biologics other than TNFi and those not followed from initiation of treatment or without any consecutive clinical registrations	BMI categories:<br>Normal: BMI <30<br>Obese: BMI ≥30	1943	Normal: 47.3 (12.5)<br>Obese: 49.4 (11.9)	Normal: 458 (53.1)<br>Obese: 236 (57.8)	**Professional body** (Danish Rheumatism Association, Danish Psoriasis Association), **Charity** (Robert and Kirsten Wehnert's fund, OAK Foundation), **Hospital** (Gentofte Hospital)

BMI = body mass index, CASPAR = Classification Criteria for Psoriatic Arthritis, IQR = interquartile range, N = number, PsA = psoriatic arthritis, RCT= randomised controlled trial, SD = standard deviation, TNFi = tumour necrosis factor inhibitor

Table – Psoriatic arthritis, description of included studies

Author (date) [country]	Study design	Inclusion criteria	Exposure detail	N	Age, mean (SD) years	N (%) female	Funders
Eder (2015) [Canada][408]	Pros. Cohort	Presence of psoriasis and inflammatory arthritis and exclusion of other types of arthritis	BMI categories:<br>Normal: BMI <25<br>Overweight: BMI 25-30<br>Obese: BMI >30	557	Normal: 50.7 (14.9)<br>Overweight: 52.3 (13.2)<br>Obese: 53.2 (10.1)	Normal: 99 (55.0)<br>Overweight: 62 (31.0)<br>Obese: 90 (45.7)	**Charity** (The Arthritis Society, Krembil Foundation), **Government** (Canadian Institutes of Health Research),
Mease (2015) [USA][409]	Pros. Cohort	Corrona Study - diagnosis of PsA with CASPAR criteria, aged ≥18 years, started biologic ≥2005, follow-up >=90 days after initiation	BMI – continuous	519	51.6 (13.0)	266 (51.3)	**Industry** (AbbVie, Amgen, AstraZeneca, Genentech, Horizon Pharma, Eli Lilly, Janssen Biotech, Novartis, Pfizer, Vertex, UCB)
Di Minno (2013) [Italy][410]	Pros. Cohort	Classification of Psoriatic Arthritis (study group) criteria, referred to start TNFi, aged ≥18 years<br>Exclusions: previous treatment with TNFi, malignancy, hematologic diseases, autoimmune diseases other than PsA, unstable medical condition, pregnancy	BMI categories:<br>Normal weight: BMI ≤30<br>Obese: BMI >30	270	Normal weight: 51.1 (13.0)<br>Obese: 52.3 (9.8)	Normal weight: 65 (48.1)<br>Obese: 81 (60.0)	**Not reported**
Iannone (2013) [Italy][411]	Pros. Cohort	CASPAR, DAS28≥3.2, anti-TNF therapy<br>Exclusions: axial or mutilans subset	BMI categories:<br>Normal: BMI <25<br>Overweight: BMI 25-30<br>Obese: BMI >30	135	Normal: 50.9 (12)<br>Overweight: 53.0 (11)<br>Obese: 56.0 (11)	Normal: 23 (53.4)<br>Overweight: 20 (42.6)<br>Obese: 24 (53.3)	**Not reported**
Haddad (2013) [Canada][412]	Case-control	CASPAR criteria	BMI – continuous	312	DISH: 62.9 (8.9)<br>No DISH: 49.3 (12.8)	DISH: 21 (26.9)<br>No DISH: 62 (26.5)	**Industry** (Janssen Canada), **Government** (Canadian Institutes of Health Research), **Charity** (The Arthritis Society, Krembil Foundation)

BMI = body mass index, CASPAR = Classification Criteria for Psoriatic Arthritis, DAS28 = Disease Activity Score 28, DISH = Diffuse Idiopathic Skeletal Hyperostosis, N = number, pros. = prospective, PsA = psoriatic arthritis, Retro. = retrospective, SD = standard deviation, TNFi = tumour necrosis factor inhibitor, UK = United Kingdom, United States of America

Supplementary table 151 – Pain outcomes from weight-loss interventions in PsA

Table – Pain (PsA), results and quality assessment

Study (date) [study type]	Standardised result, SMD (95% CI) unless otherwise stated	Natural result	Supports intervention	AMSTAR2 quality	Rand. Seq.	Alloc. Conc.	Blind. Part.	Blind. Asses.
Di Minno (2014) [RCT][404]		Pain VAS, change baseline-6 months, mean (SD)<br><5% weight loss: -1.97 (2.42)<br>≥5% weight loss: -4.00 (2.90), p=0.018 vs <5%<br>>10% weight loss: -5.06 (2.64), p<0.001 vs <5%	✓		L	H/UC	H/UC	L
Klingberg (2019) [Single arm int.][405]		VAS pain (mm), Baseline / 6 months, median (IQR)<br>30 (18.5, 62.5) / 20 (5, 51.5) p=0.004	✓					

Alloc. Conc. = allocation concealment, AMSTAR2 = Assessing the Methodological Quality of Systematic Reviews, Blind. Asses. = Blinded assessors, Blind. Part. = blinded participants, CI = confidence interval, H/UC = high / unclear risk of bias, IQR = interquartile range, L = low risk of bias, Rand. Seq. = random sequence generation, SD = standard deviation, SMD = Standardised mean difference, VAS = visual analogue scale

Supplementary table 152 – Pain outcomes from observational studies in PsA

Table – Pain (PsA), results and quality assessment of observational studies

Study (date) [study type]	Results	Weight associated with outcome	Study Pop.	Attr.	Prog. Meas.	Outc. Meas.	Conf.	Stats.
Eder (2015) [Prospective cohort][408]	Pain VAS <=5, OR (95% CI), adjusted<br>Overweight vs Normal: 0.56 (0.43, 0.73)<br>Obese vs Normal: 0.45 (0.34, 0.58)	✓	L	M	L	L	L	M

Attr. = attrition, CI = confidence interval, Conf. = confounding, L = low risk of bias, M = moderate risk of bias, OR = odds ratio, Outc. Meas = outcome measurement, Prog. Meas. = prognostic factor measurement, Pros = prospective, PsA = psoriatic arthritis, Stats. = statistical analysis, Study Pop. = study population, VAS = visual analogue scale

Supplementary table 153 – Function outcomes from weight-loss interventions in PsA

Table – Function (PsA), results and quality assessment

Study (date) [study type]	Standardised result, SMD (95% CI) unless otherwise stated	Natural result	Supports intervention	AMSTAR2 quality	Rand. Seq.	Alloc. Conc.	Blind. Part.	Blind. Asses.
Di Minno (2014) [RCT][404]		HAQ, change bl-6 mth, mean (SD)<br><5% weight loss: -0.53 (0.67)<br>≥5% weight loss: -1.29 (0.79) p=0.004	✓		L	H/UC	H/UC	L
Klingberg (2019) [Single arm int.][405]		HAQ, Baseline / 6 months, median (IQR)<br>0.70 (0.13, 1.00) / 0.43 (0, 0.69) p<0.001	✓					

Alloc. Conc. = allocation concealment, AMSTAR2 = Assessing the Methodological Quality of Systematic Reviews, BL = baseline, Blind. Asses. = Blinded assessors, Blind. Part. = blinded participants, CI = confidence interval, H/UC = high / unclear risk of bias, HAQ = Health Assessment Questionnaire, IQR = interquartile range, L = low risk of bias, PsA = psoriatic arthritis, Rand. Seq. = random sequence generation, SMD = Standardised mean difference, VAS = visual analogue scale

Supplementary table 154 – Function outcomes from observational studies in PsA

Table – Function (PsA), results and quality assessment of observational studies

Study (date) [study type]	Results	Weight associated with outcome	Study Pop.	Attr.	Prog. Meas.	Outc. Meas.	Conf.	Stats.
Eder (2015) [Prospective cohort][408]	HAQ <=0.5, OR (95% CI), adjusted<br>Overweight vs Normal weight: 0.84 (0.68, 1.03)<br>Obese vs Normal weight: 0.62 (0.51, 0.75)	✓	L	M	L	L	L	M
Iannone (2013) [Prospective cohort][411]	HAQ at follow-up, mean (SD)<br>Normal: 0.79 (0.9)<br>Overweight: 0.47 (0.8)<br>Obese: 0.81 (0.8) p=0.06	×	L	L	L	L	L	M

Attr. = attrition, CI = confidence interval, Conf. = confounding, HAQ = Health Assessment Questionnaire, L = low risk of bias, M = moderate risk of bias, OR = odds ratio, Outc. Meas = outcome measurement, Prog. Meas. = prognostic factor measurement, Pros = prospective, PsA = psoriatic arthritis, SD = standard deviation, Stats. = statistical analysis, Study Pop. = study population

Supplementary table 155 – Disease activity outcomes from weight-loss interventions in PsA

Table – Disease activity (PsA), results and quality assessment

Study (date) [study type]	Standardised result, SMD (95% CI) unless otherwise stated	Natural result	Supports intervention	AMSTAR2 quality	Rand. Seq.	Alloc. Conc.	Blind. Part.	Blind. Asses.
Di Minno (2014) [RCT][404]		MDA, N(%) and OR (95% CI)<br><5% weight loss: 23.1%<br>≥5% weight loss: 50% OR 4.20 (1.82, 9.66)<br>Weight loss intervention: 42.9%<br>Control: 34.9% HR 1.85 (1.019, 3.345)	✓		L	H/UC	H/UC	L
Klingberg (2019) [Single arm int.][405]		DAS28, baseline /6 months, median (IQR)<br>2.9 (2.1, 3.7) / 2.4 (1.7, 3.0) p<0.001<br>DAPSA, Baseline / 6 months, median (IQR)<br>15.3 (6.6, 29.1) / 11.0 (2.8, 17.6) p<0.001	✓					

Alloc. Conc. = allocation concealment, AMSTAR2 = Assessing the Methodological Quality of Systematic Reviews, Blind. Asses. = Blinded assessors, Blind. Part. = blinded participants, CI = confidence interval, DAPSA = Disease Activity in Psoriatic Arthritis, DAS28 = Disease Activity Score 28, H/UC = high / unclear risk of bias, IQR = interquartile range, L = low risk of bias, MDA = minimal disease activity, OR = odds ratio, Rand. Seq. = random sequence generation, SMD = Standardised mean difference

Supplementary table 156 – Disease activity outcomes from observational studies in PsA

Table – Disease activity (PsA), results and quality assessment of observational studies

Study (date) [study type]	Results	Weight associated with outcome	AMSTAR2	Study Pop.	Attr.	Prog. Meas.	Outc. Meas.	Conf.	Stats.
Lupoli (2016) [Meta-analysis][323]	MDA, obese vs normal<br>OR 0.369 (0.249, 0.546)	✓	Low						
Hojgaard (2016) [Prospective cohort][407] (TNFi)	DAS28 at 3 months, mean change (SD)<br>Obese: -1.49 (SD 1.48)<br>Non-obese: -1.47 (1.37) p=0.82<br>DAS28 at 6 months, mean change (SD)<br>Obese: -1.65 (1.49)<br>Non-obese: -1.59 (1.42) p=0.72<br>EULAR good response 6 months, OR (95% CI) [adjusted]<br>Obese vs non-obese: 0.75 (0.50, 1.15)<br>EULAR good or moderate 6 months, OR (95% CI) [adjusted]<br>Obese vs non-obese: 0.47 (0.30, 0.74)	✓		L	H	L	L	L	M
Eder (2015) [Prospective cohort][408]	MDA, OR (95% CI), unadjusted / adjusted<br>Overweight vs normal: 0.65 (0.50, 0.85) / 0.66 (0.50, 0.87)<br>Obese vs normal: 0.52 (0.40, 0.67) / 0.53 (0.41, 0.69)	✓		L	M	L	L	L	M
Mease (2015) [Prospective cohort][409]	Time to remission, HR (p value)<br>BMI continuous: 0.955 (p<0.001)	✓		L	M	L	L	L	M
Di Minno (2013) [Prospective cohort][410]	Not achieving MDA in first 12 months, HR (95% CI) [adjusted]<br>Obese vs normal: HR 4.90 (3.04, 7.87)<br>Not achieving MDA in second 12 months, HR (95% CI) [adjusted]<br>Obese vs normal: HR 2.04 (1.015, 3.61)	✓		L	L	L	L	L	M
Iannone (2013) [Prospective cohort][411] (TNFi)	DAS28 at follow-up, normal/overweight/obese, mean (SD)<br>3.1 (1.6) / 2.9 (1.6) / 3.2 (1.5) p=0.42<br>SDAI at follow-up, normal/overweight/obese, mean (SD)<br>14.2 (13) / 11.6 (12) / 13.0 (12) p=0.44<br>DAS28 remission, normal/overweight/obese, %<br>44 / 46 / 37<br>BMI: OR 0.96 (0.78, 1.17) [adjusted]<br>obesity (y/n): OR 1.17 (0.11, 11.8) [adjusted]<br>SDAI remission, normal/overweight/obese, %<br>21 / 38 / 21 p=0.07	×		L	L	L	L	L	M

Attr. = attrition, BMI = body mass index, CI = confidence interval, Conf. = confounding, DAS28 = disease activity score 28, EULAR = European League Against Rheumatism, H = high, HR = hazard ratio, L = low risk of bias, M = moderate risk of bias, MDA = minimal disease activity, OR = odds ratio, Outc. Meas = outcome measurement, Prog. Meas. = prognostic factor measurement, Pros = prospective, PsA = psoriatic arthritis, SD = standard deviation, SDAI = Simplified Disease Activity Index, Stats. = statistical analysis, Study Pop. = study population, TNFi = tumour necrosis factor inhibitor

Supplementary table 157 – Tender joint count outcomes from weight-loss interventions in PsA

Table – Tender joint count (PsA), results and quality assessment

Study (date) [study type]	Standardised result, SMD (95% CI) unless otherwise stated	Natural result	Supports intervention	AMSTAR2 quality	Rand. Seq.	Alloc. Conc.	Blind. Part.	Blind. Asses.
Klingberg (2019) [Single arm int.][405]		Tender joint count, Baseline /6 months, median (IQR)<br>4 (1-14) / 2 (0-6.5) p<0.001	✓					

Alloc. Conc. = allocation concealment, AMSTAR2 = Assessing the Methodological Quality of Systematic Reviews, Blind. Asses. = Blinded assessors, Blind. Part. = blinded participants, CI = confidence interval, H/UC = high / unclear risk of bias, IQR = interquartile range, L = low risk of bias, PsA = psoriatic arthritis, Rand. Seq. = random sequence generation, SMD = Standardised mean difference

Supplementary table 158 – Tender joint count outcomes from observational studies in PsA

Table – Tender joint count (PsA), results and quality assessment of observational studies

Study (date) [study type]	Results	Weight associated with outcome	Study Pop.	Attr.	Prog. Meas.	Outc. Meas.	Conf.	Stats.
Eder (2015) [Prospective cohort][408]	Tender joint count ≤1, OR (95% CI), adjusted<br>Overweight vs Normal: 0.88 (0.73, 1.06)<br>Obese vs Normal: 0.79 (0.66, 0.93)	✓	L	M	L	L	L	M

Attr. = attrition, CI = confidence interval, Conf. = confounding, L = low risk of bias, M = moderate risk of bias, OR = odds ratio, Outc. Meas = outcome measurement, Prog. Meas. = prognostic factor measurement, Pros = prospective, PsA = psoriatic arthritis, Stats. = statistical analysis, Study Pop. = study population

Supplementary table 159 – Swollen joint count outcomes from weight-loss interventions in PsA

Table – Swollen joint count (PsA), results and quality assessment

Study (date) [study type]	Standardised result, SMD (95% CI) unless otherwise stated	Natural result	Supports intervention	AMSTAR2 quality	Rand. Seq.	Alloc. Conc.	Blind. Part.	Blind. Asses.
Klingberg (2019) [Single arm int.][405]		Swollen joint count, Baseline / 6 months, median (IQR)<br>0 (0-1) / 0 (0-0.5) p=0.021	✓					

Alloc. Conc. = allocation concealment, AMSTAR2 = Assessing the Methodological Quality of Systematic Reviews, Blind. Asses. = Blinded assessors, Blind. Part. = blinded participants, CI = confidence interval, H/UC = high / unclear risk of bias, IQR = interquartile range, L = low risk of bias, PsA = psoriatic arthritis, SMD = Standardised mean difference

Supplementary table 160 – Swollen joint count outcomes from observational studies in PsA

Table – Swollen joint count (PsA), results and quality assessment of observational studies

Study (date) [study type]	Results	Weight associated with outcome	Study Pop.	Attr.	Prog. Meas.	Outc. Meas.	Conf.	Stats.
Eder (2015) [Prospective cohort][408]	Swollen joint count ≤1, OR (95% CI) [adjusted]<br>Overweight vs Normal: 1.11 (0.88, 1.40)<br>Obese vs Normal: 1.19 (0.95, 1.48)	×	L	M	L	L	L	M

Attr. = attrition, CI = confidence interval, Conf. = confounding, L = low risk of bias, M = moderate risk of bias, OR = odds ratio, Outc. Meas = outcome measurement, Prog. Meas. = prognostic factor measurement, Pros = prospective, PsA = psoriatic arthritis, Rand. Seq. = random sequence generation, Stats. = statistical analysis, Study Pop. = study population

Supplementary table 161 – Patient global assessment outcomes from weight-loss interventions in PsA

Table – Patient global (PsA), results and quality assessment

Study (date) [study type]	Standardised result, SMD (95% CI) unless otherwise stated	Natural result	Supports intervention	AMSTAR2 quality	Rand. Seq.	Alloc. Conc.	Blind. Part.	Blind. Asses.
Di Minno (2014) [RCT][404]		Patient global VAS, change baseline-6 months, mean (SD), p values vs <5% weight loss<br><5% weight loss: -2.56 (1.94)<br>≥5% weight loss: -4.68 (2.92), p<0.001<br>>10 % weight loss: -4.26 (2.02), p=0.008	✓		L	H/UC	H/UC	L
Klingberg (2019) [Single arm int.][405]		VAS global (mm), baseline / 6 months, median (IQR)<br>34 (19,61) / 12 (5,51) p=0.001	✓					

Alloc. Conc. = allocation concealment, AMSTAR2 = Assessing the Methodological Quality of Systematic Reviews, Blind. Asses. = Blinded assessors, Blind. Part. = blinded participants, CI = confidence interval, H/UC = high / unclear risk of bias, IQR = interquartile range, L = low risk of bias, Rand. Seq. = random sequence generation, SD = standard deviation, SMD = Standardised mean difference, VAS = visual analogue scale

Supplementary table 162 – Patient global assessment outcomes from observational studies in PsA

Table – Patient global (PsA), results and quality assessment of observational studies

Study (date) [study type]	Results	Weight associated with outcome	Study Pop.	Attr.	Prog. Meas.	Outc. Meas.	Conf.	Stats.
Eder (2015) [Prospective cohort][408]	Patient global assessment VAS <=20, OR (95% CI), [adjusted]<br>Overweight vs Normal: 0.44 (0.36, 0.55)<br>Obese vs Normal: 0.35 (0.29, 0.43)	✓	L	M	L	L	L	M

Attr. = attrition, CI = confidence interval, Conf. = confounding, L = low risk of bias, M = moderate risk of bias, OR = odds ratio, Outc. Meas = outcome measurement, Prog. Meas. = prognostic factor measurement, Pros = prospective, PsA = psoriatic arthritis, Rand. Seq. = random sequence generation, Stats. = statistical analysis, Study Pop. = study population, VAS = visual analogue scale

Supplementary table 163 – Quality of life outcomes from weight-loss interventions in PsA

Table – Quality of life (PsA), results and quality assessment

Study (date) [study type]	Standardised result, SMD (95% CI) unless otherwise stated	Natural result	Supports intervention	AMSTAR2 quality	Rand. Seq.	Alloc. Conc.	Blind. Part.	Blind. Asses.
Klingberg (2019) [Single arm int.][405]		Dermatology Quality of Life , Baseline /6 months, median (IQR)<br>1 (0, 4.5) / 1 (0, 4) p=0.453	×					

Alloc. Conc. = allocation concealment, AMSTAR2 = Assessing the Methodological Quality of Systematic Reviews, Blind. Asses. = Blinded assessors, Blind. Part. = blinded participants, CI = confidence interval, H/UC = high / unclear risk of bias, IQR = interquartile range, L = low risk of bias, PsA = psoriatic arthritis, Rand. Seq. = random sequence generation, SMD = Standardised mean difference

Supplementary table 164 – Fatigue outcomes from weight-loss interventions in PsA

Table – Fatigue (PsA), results and quality assessment

Study (date) [study type]	Standardised result, SMD (95% CI) unless otherwise stated	Natural result	Supports intervention	AMSTAR2 quality	Rand. Seq.	Alloc. Conc.	Blind. Part.	Blind. Asses.
Klingberg (2019) [Single arm int.][405]		VAS fatigue (mm) , Baseline /6 months, median (IQR)<br>56 (21.5, 67) / 25 (8, 44) p=0.001	✓					

Alloc. Conc. = allocation concealment, AMSTAR2 = Assessing the Methodological Quality of Systematic Reviews, Blind. Asses. = Blinded assessors, Blind. Part. = blinded participants, CI = confidence interval, H/UC = high / unclear risk of bias, IQR = interquartile range, L = low risk of bias, PsA = psoriatic arthritis, Rand. Seq. = random sequence generation, SMD = Standardised mean difference, VAS = visual analogue scale

Supplementary table 165 – CRP outcomes from weight-loss interventions in PsA

Table – CRP (PsA), results and quality assessment

Study (date) [study type]	Standardised result, SMD (95% CI) unless otherwise stated	Natural result	Supports intervention	AMSTAR2 quality	Rand. Seq.	Alloc. Conc.	Blind. Part.	Blind. Asses.
Di Minno (2014) [RCT][404]		CRP , change Baseline -6 months, mean (SD)<br><5% weight loss: -1.37 (7.48)<br>≥5% weight loss: -5.01 (9.5) p=0.023	✓		L	H/UC	H/UC	L
Klingberg (2019) [Single arm int.][405]		CRP, Baseline / 6 months, median (IQR)<br>4 (2, 8.5) / 2 (1,6.5) p=0.041	✓					

Alloc. Conc. = allocation concealment, AMSTAR2 = Assessing the Methodological Quality of Systematic Reviews, Blind. Asses. = Blinded assessors, Blind. Part. = blinded participants, CI = confidence interval, CRP = C-reactive Protein, H/UC = high / unclear risk of bias, IQR = interquartile range, L = low risk of bias, PsA = psoriatic arthritis, Rand. Seq. = random sequence generation, SD = standard deviation, SMD = Standardised mean difference

Supplementary table 166 – ESR outcomes from weight-loss interventions in PsA

Table – ESR (PsA), results and quality assessment

Study (date) [study type]	Standardised result, SMD (95% CI) unless otherwise stated	Natural result	Supports intervention	AMSTAR2 quality	Rand. Seq.	Alloc. Conc.	Blind. Part.	Blind. Asses.
Di Minno (2014) [RCT][404]		ESR, change bl-6 mth, mean (SD)<br>Weight loss intervention: -14.9 (18.0)<br>Control: -2.04 (15.5) p<0.001<br><5% weight loss: -3.09 (16.2)<br>≥5% weight loss: -12.25 (18.22), p=0.004<br>>10% weight loss: -14.45 (20.14), p<0.001	✓		L	H/UC	H/UC	L

Alloc. Conc. = allocation concealment, AMSTAR2 = Assessing the Methodological Quality of Systematic Reviews, BL = baseline, Blind. Asses. = Blinded assessors, Blind. Part. = blinded participants, CI = confidence interval, ESR = erythrocyte sedimentation rate, H/UC = high / unclear risk of bias, L = low risk of bias, PsA = psoriatic arthritis, Rand. Seq. = random sequence generation, SD = standard deviation, SMD = Standardised mean difference

Supplementary table 167 – Psorisasis outcomes from weight-loss interventions in PsA

Table – Psoriasis score (PsA), results and quality assessment

Study (date) [study type]	Standardised result, SMD (95% CI) unless otherwise stated	Natural result	Supports intervention	AMSTAR2 quality	Rand. Seq.	Alloc. Conc.	Blind. Part.	Blind. Asses.
Klingberg (2019) [Single arm int.][405]		Psoriasis body surface area %, Baseline / 6 months, median (IQR)<br>1.6 (0, 2.2) / 0.9 (0, 1.1) p=0.014	✓					

Alloc. Conc. = allocation concealment, AMSTAR2 = Assessing the Methodological Quality of Systematic Reviews, Blind. Asses. = Blinded assessors, Blind. Part. = blinded participants, CI = confidence interval, H/UC = high / unclear risk of bias, IQR = interquartile range, L = low risk of bias, PsA = psoriatic arthritis, SMD = Standardised mean difference

Supplementary table 168 – Psoriasis outcomes from observational studies in PsA

Table – Psoriasis score (PsA), results and quality assessment of observational studies

Study (date) [study type]	Results	Weight associated with outcome	Study Pop.	Attr.	Prog. Meas.	Outc. Meas.	Conf.	Stats.
Eder (2015) [Prospective cohort][408]	PASI ≤1 or BSA ≤1, OR (95% CI), adjusted<br>Overweight vs Normal weight: 0.43 (0.31, 0.61)<br>Obese vs Normal weight: 0.28 (0.21, 0.39)	✓	L	M	L	L	L	M

Attr. = attrition, CI = confidence interval, Conf. = confounding, L = low risk of bias, M = moderate risk of bias, OR = odds ratio, Outc. Meas = outcome measurement, PASI = Psoriasis Area Severity Index, Prog. Meas. = prognostic factor measurement, Pros = prospective, PsA = psoriatic arthritis, Stats. = statistical analysis, Study Pop. = study population

Supplementary table 169 – Comorbidity outcomes from observational studies in PsA

Table – Comorbidity (PsA), results and quality assessment of observational studies

Study (date) [study type]	Results	Weight associated with outcome	Study Pop.	Attr.	Prog. Meas.	Outc. Meas.	Conf.	Stats.
Polachek (2017) [Prospective cohort][406]	Enthesitis occurrence, HR (95% CI)<br>BMI: 1.04 (1.005, 1.07) [adjusted]	✓	L	L	L	L	L	M
Haddad (2013) [Case-control][412]	DISH, OR (95% CI)<br>BMI cont.: 1.18 (1.09, 1.28) [adjusted]	✓	L	L	L	L	L	M

Attr. = attrition, BMI = body mass index, CI = confidence interval, Conf. = confounding, DISH = Diffuse Idiopathic Skeletal Hyperostosis, HR = hazard ratio, L = low risk of bias, M = moderate risk of bias, OR = odds ratio, Outc. Meas = outcome measurement, Prog. Meas. = prognostic factor measurement, Pros = prospective, PsA = psoriatic arthritis, Rand. Seq. = random sequence generation, Stats. = statistical analysis, Study Pop. = study population

Supplementary table 170 – Drug discontinuation outcomes from observational studies in PsA

Table – Drug discontinuation (PsA), results and quality assessment of observational studies

Study (date) [study type]	Results	Weight associated with outcome	Study Pop.	Attr.	Prog. Meas.	Outc. Meas.	Conf.	Stats.
Mease (2015) [Prospective cohort][409]	TNF persistence, HR (p value)<br>BMI continuous: 1.011 (p=0.44) [adjusted]	×	L	M	L	L	L	M

Attr. = attrition, BMI = body mass index, CI = confidence interval, Conf. = confounding, HR = hazard ratio, L = low risk of bias, M = moderate risk of bias, Outc. Meas = outcome measurement, Prog. Meas. = prognostic factor measurement, Pros = prospective, PsA = psoriatic arthritis, Stats. = statistical analysis, Study Pop. = study population, TNFi = tumour necrosis factor inhibitor

Supplementary table 171 – Description of studies of assessing weight and outcomes in SSc

Table – Systemic sclerosis, description of included studies

Author (date) [country]	Study design	Inclusion criteria	Exposure detail	N	Age, mean (SD) years	N (%) female	Funders
Marini (2016) [Italy][413]	Pros. Cohort	SSc ACR/EULAR criteria with pulmonary artery hypertension (HAP)"	BMI quartiles	49	Died: 62 (13)<br>Alive: 63 (12)	Died: 15 (88.2)<br>Alive: 26 (81.3)	**Not reported** – authors declared no conflict of interest
Assassi (2009) [USA][414]	Pros. Cohort	GENIOS study – aged ≥18 years, ACR 1980 SSc criteria, disease duration <5 years, defined ethnicity with all 4 grandparents from the same ethnic group<br>Exclusions: SSc like illnesses associated with environmental, ingested or injected agents	BMI categories:<br>BMI 18.5-24.9<br>BMI 25-29.9<br>BMI >30<br>BMI <18.5	250	48.9 (13.2)	84%	**Government** (NIH), **University** (University of Texas), **Professional body** (ACR)

ACR = American College of Rheumatology, BMI = body mass index, EULAR = European League Against Rheumatism, N = number, NIH = National Institute of Health, pros. = prospective, SD = standard deviation, SSc = systemic sclerosis, USA = United States of America

Supplementary table 172 – Mortality outcomes from observational studies in SSc

Table – Death (SSc), results and quality assessment of observational studies

Study (date) [study type]	Results	Weight associated with outcome	Study Pop.	Attr.	Prog. Meas.	Outc. Meas.	Conf.	Stats.
Marini (2016) [Prospective cohort][413]	Mortality, HR (95% CI) [unadjusted]<br>BMI up to 24 months: HR 2.20 (1.19, 4.06)<br>BMI up to 72 months: HR 0.95 (0.886, 1.017)<br><br>Percentage survived over 24 months<br>Obese: 100%<br>Overweight: 72.7%<br>Normal weight: 58.4%<br>Underweight: 40.0%, p=0.031<br><br>Mortality, HR (95% CI) [adjusted]<br>BMI : 0.89 (0.79, 1.01) up to 24 months<br>BMI: 0.93 (0.82, 1.05) up to 72 months	✓	M	L	M	L	L	M
Assassi (2009) [Prospective cohort][414]	Mortality, HR (95% CI), adjusted for age<br>BMI 18.5-24.9: ref<br>BMI 25-29.9: 0.53 (0.26, 1.08)<br>BMI >30: 0.48 (0.2, 1.17)<br>BMI <18.5: 6.12 (2.26, 16.58)<br>Mortality, HR (95% CI), further adjusted<br>≥25 BMI: ref<br>18.5-24.9: 2.39 (1.21, 4.72)<br><18.5: 12.94 (4.32, 38.80)	✓	L	L	L	M	L	M

Attr. = attrition, BMI = body mass index, CI = confidence interval, Conf. = confounding, HR = hazard ratio, L = low risk of bias, M = moderate risk of bias, Outc. Meas = outcome measurement, Prog. Meas. = prognostic factor measurement, Pros = prospective, SD = standard deviation, SSc = systemic sclerosis, Stats. = statistical analysis, Study Pop. = study population

Supplementary table 173 – Description of studies of assessing weight and outcomes in gout

Table – Gout, description of reviews

Authors (date)	Review type	Study type included	Exposure detail	Number of studies included	Funders
Nielsen (2017)[415]	SR	RCTs, observational studies	Studies reporting on the effect of weight loss in overweight / obese gout patients	10	**Charity** (Oak Foundation, The will of Mrs Elise Fredriksen)

RCT = randomised controlled trial, SR = systematic review

Table – Gout, description of included studies

Author (date) [country]	Study design	Inclusion criteria	Exposure detail	N	Age, mean (SD) years	N (%) female	Funders
Dessein (2000) [S. Afrcia][416]	Single arm int.	Exclusions: biochemical evidence of diabetes, hypothyroidism or renal impairment, coronary artery disease, alcohol consumption in excess of 25 g a day, and current use of equal or less carbohydrate or saturated fat than recommended in the diet. Fewer than 2 gout attacks or could not accurately remember gout attacks	Calorie restriction – 1600 kcal per day	13	median (range): 50 (30-62)	0 (0)	**Industry** (Lancet Laboratories)
Nguyen (2017) [USA][417]	Pros. Cohort	Multiple Risk Factor Intervention Trial (MRFIT) - incident gout during the study, 35-57 years, men	BMI categories:<br>Obesity: BMI ≥39<br>Percentage change in BMI	408	Not reported	408 (100)	**Charity** (Arthritis Foundation, Rheumatology Research Foundation), **Government** (NIH)
Romero-Talamas (2014) [USA][418]	Pros. Cohort	Morbidly obese, active gout (at least one documented episode / treatment)	Bariatric surgery	Surgery: 99<br>Control: 56	Surgery: 52.1 (10.3)<br>Control: 63.3 (11.9)	Surgery: 74 (74.7)<br>Control: 34 (60.7)	**Not reported**
Su (2008) [Taiwan][419]	Pros. Cohort	Subjects attending medical centre, primary clinically defined gout	BMI – continuous	318	Renal function deterioration: 57.2 (13.0)<br>No renal function deterioration: 62.5 (15.0)	0 (0)	**Not reported**
Abhishek (2016) [UK][420]	Case-control	Cases = >2 acute gout attacks in previous 12 months. ACR gout crit.<br>Controls = ≤2 gout attacks in previous 12 months<br>Exclusions: taking urate lowering treatment	BMI tertiles:<br>T1 = <27.4<br>T2 = 27.4-30.8<br>T3 = >30.8	468	62.2 (11.3)	11.5%	**Industry** (AstraZeneca, Oxford Immunotech)
Alvarez-Nemegyei (2005) [Mexico][421]	Nested Case-control	Wallace criteria for gout<br>Exclusions: Secondary gout, no measured outcomes	BMI categories<br>BMI >30	90	54 (12)	2 (2.2)	**Not reported**

ACR = American College of Rheumatology, BMI = body mass index, N = number, NIH = National Institute of Health, pros. = prospective, SD = standard deviation, UK = United Kingdom, USA = United States of America

Supplementary table 174 – Function outcomes from weight-loss interventions in gout

Table – Function (Gout), results and quality assessment

Study (date) [study type]	Standardised result, SMD (95% CI) unless otherwise stated	Natural result	Supports intervention	AMSTAR2 quality	Rand. Seq.	Alloc. Conc.	Blind. Part.	Blind. Asses.
Nielsen (2017) [SR][415]		One study reported reductions in function over time at the same time as weight loss from bariatic sturgery. Population included patients with and without gout.	×	Moderate				

Alloc. Conc. = allocation concealment, AMSTAR2 = Assessing the Methodological Quality of Systematic Reviews, Blind. Asses. = Blinded assessors, Blind. Part. = blinded participants, CI = confidence interval, H/UC = high / unclear risk of bias, L = low risk of bias, Rand. Seq. = random sequence generation, SMD = Standardised mean difference, SR = systematic review

Supplementary table 175 – Function outcomes from observational studies in gout

Table – Function (Gout), results and quality assessment of observational studies

Study (date) [study type]	Results	Weight associated with outcome	Study Pop.	Attr.	Prog. Meas.	Outc. Meas.	Conf.	Stats.
Alvarez-Nemegyei (2005) [Prospective cohort][421]	Number (%) of disabled/not disability patients with obesity<br>Disabled: 33/42 (78%)<br>Not disabled: 34/48 (70%) p=0.27<br>Mean (SD) BMI, disabled/not disabled<br>disabled: 31 (4.6)<br>not disabled: 30 (5.3) p=0.36	×	L	M	M	L	H	H

Attr. = attrition, BMI = body mass index, CI = confidence interval, Conf. = confounding, L = low risk of bias, M = moderate risk of bias, Outc. Meas = outcome measurement, Prog. Meas. = prognostic factor measurement, Pros = prospective, Rand. Seq. = random sequence generation, SD = standard deviation, Stats. = statistical analysis, Study Pop. = study population

Supplementary table 176 – Uric acid outcomes from weight-loss interventions in gout

Table – Serum uric acid (Gout), results and quality assessment

Study (date) [study type]	Standardised result, SMD (95% CI) unless otherwise stated	Natural result	Supports intervention	AMSTAR2 quality	Rand. Seq.	Alloc. Conc.	Blind. Part.	Blind. Asses.
Nielsen (2017) [SR][415]		Low to moderate quality evidence for the benefit of weight loss for overweight patient with gout in terms of serum uric acid	✓	Moderate				
Dessein (2000) [Single arm int.][416]		Serum uric acid, Baseline / 16 weeks, median (SD)<br>0.57 (0.10) / 0.47 (0.09) p=0.001	✓					

Alloc. Conc. = allocation concealment, AMSTAR2 = Assessing the Methodological Quality of Systematic Reviews, Blind. Asses. = Blinded assessors, Blind. Part. = blinded participants, CI = confidence interval, H/UC = high / unclear risk of bias, L = low risk of bias, Rand. Seq. = random sequence generation, SD = standard deviation, SMD = Standardised mean difference, SR = systematic review

Supplementary table 177 – Uric acid outcomes from observational studies in gout

Table – Uric acid (Gout), results and quality assessment of observational studies

Study (date) [study type]	Results	Weight associated with outcome	Study Pop.	Attr.	Prog. Meas.	Outc. Meas.	Conf.	Stats.
Romero-Talamas (2014) [Prospective cohort][418]	Serum uric acid, baseline / 13 months, mean (SD)<br>Bariatric surgery: 9.1 (2.0) / 5.6 (2.5)<br>Control: 7.7 (2.0) / 7.0 (1.6)	✓	L	L	L	L	H	H

Attr. = attrition, CI = confidence interval, Conf. = confounding, L = low risk of bias, M = moderate risk of bias, Outc. Meas = outcome measurement, Prog. Meas. = prognostic factor measurement, Pros = prospective, PsA = psoriatic arthritis, Rand. Seq. = random sequence generation, SD = standard deviation, Stats. = statistical analysis, Study Pop. = study population

Supplementary table 178 – Gout attack outcomes from weight-loss interventions in gout

Table – Gout attacks (Gout), results and quality assessment

Study (date) [study type]	Standardised result, SMD (95% CI) unless otherwise stated	Natural result	Supports intervention	AMSTAR2 quality	Rand. Seq.	Alloc. Conc.	Blind. Part.	Blind. Asses.
Nielsen (2017) [SR][415]		Low to moderate quality evidence for the benefit of weight loss for overweight patient with gout in terms of gout attacks	✓	Moderate				
Dessein (2000) [Single arm int.][416]		Attacks per month, BL / 16 weeks, median (SD)<br>2.1 (0.8) / 0.6 (0.7), p=0.002	✓					

Alloc. Conc. = allocation concealment, AMSTAR2 = Assessing the Methodological Quality of Systematic Reviews, BL = baseline, Blind. Asses. = Blinded assessors, Blind. Part. = blinded participants, CI = confidence interval, H/UC = high / unclear risk of bias, L = low risk of bias, Rand. Seq. = random sequence generation, SD = standard deviation, SMD = Standardised mean difference, SR = systematic review

Supplementary table 179 – Gout attack outcomes from observational studies in gout

Table – Gout attacks (Gout), results and quality assessment of observational studies

Study (date) [study type]	Results	Weight associated with outcome	Study Pop.	Attr.	Prog. Meas.	Outc. Meas.	Conf.	Stats.
Nguyen (2017) [Prospective cohort][417]	Recurrent gout attacks, OR (95% CI)<br>BL BMI: 0.98 (0.53, 1.81)<br>decrease >5% BMI: 0.61 (0.32, 1.16)<br>decrease 3.6-5% BMI: 0.94 (0.43, 2.06)<br>no change (-3.5% to 3.5%): ref<br>increase 3.6%-5%: 1.43 (0.75, 2.72)<br>increase >5%: 1.60 (0.89, 2.89)<br>p for trend <0.01	✓	L	L	L	L	L	M
Romero-Talamas (2014) [Prospective cohort][418]	Gout attacks (%), number from surgery to 12 months<br>Bariatric surgery: 8.0%<br>Control: 11.1%	✓	L	L	L	L	H	H
Abhishek (2016) [Case-control][420]	Gout attacks, Tertile 1 = ref, OR (95%CI)<br>unadjusted<br>Tertile 2 = 1.42 (0.91, 2.23)<br>Tertile 3 = 1.72 (1.10, 2.70)<br>adjusted<br>Tertile 2 = 1.44 (0.90, 2.31)<br>Tertile 3 = 1.53 (0.95, 2.46)	×	L	L	L	H	L	L

Attr. = attrition, BL = baseline, BMI = body mass index, CI = confidence interval, Conf. = confounding, L = low risk of bias, M = moderate risk of bias, OR = odds ratio, Outc. Meas = outcome measurement, Prog. Meas. = prognostic factor measurement, SD = standard deviation, Stats. = statistical analysis, Study Pop. = study population

Supplementary table 180 – Comorbidity outcomes from observational studies in gout

Table – Comorbidity (Gout), results and quality assessment of observational studies

Study (date) [study type]	Results	Weight associated with outcome	Study Pop.	Attr.	Prog. Meas.	Outc. Meas.	Conf.	Stats.
Su (2008) [Prospective cohort][419]	Mean baseline BMI (SD)<br>Renal failure: 27.69 (4.53)<br>No renal failure: 26.16 (3.66) p=0.111<br>BMI not included in multivariable model, in neither logistic and Cox regression analysis<br>Multivariable logistic regression<br>Waist circumference: 1.058 (1.009, 1.110)	BMI: ✗<br>Waist circumference: ✓	L	L	L	L	M	M
Alvarez-Nemegyei (2005) [Prospective cohort][421]	Number (%) of renal failure/no failure patients with obesity<br>Renal failure: 18/25 (72%)<br>No renal failure: 43/55 (78%) p=0.54<br>Mean (SD) BMI, renal failure/no failure<br>Renal failure: 29 (3)<br>No renal failure: 30 (4) p=0.35	✗	L	M	M	L	H	H

Attr. = attrition, BMI = body mass index, CI = confidence interval, Conf. = confounding, L = low risk of bias, M = moderate risk of bias, Outc. Meas = outcome measurement, Prog. Meas. = prognostic factor measurement, Pros = prospective, SD = standard deviation, Stats. = statistical analysis, Study Pop. = study population

Reference List

(1) Mease PJ. Measures of psoriatic arthritis: Tender and Swollen Joint Assessment, Psoriasis Area and Severity Index (PASI), Nail Psoriasis Severity Index (NAPSI), Modified Nail Psoriasis Severity Index (mNAPSI), Mander/Newcastle Enthesitis Index (MEI), Leeds Enthesitis Index (LEI), Spondyloarthritis Research Consortium of Canada (SPARCC), Maastricht Ankylosing Spondylitis Enthesis Score (MASES), Leeds Dactylitis Index (LDI), Patient Global for Psoriatic Arthritis, Dermatology Life Quality Index (DLQI), Psoriatic Arthritis Quality of Life (PsAQOL), Functional Assessment of Chronic Illness Therapy-Fatigue (FACIT-F), Psoriatic Arthritis Response Criteria (PsARC), Psoriatic Arthritis Joint Activity Index (PsAJAI), Disease Activity in Psoriatic Arthritis (DAPSA), and Composite Psoriatic Disease Activity Index (CPDAI). *Arthritis Care Res (Hoboken)* 2011; 63 Suppl 11:S64-S85.

(2) Zochling J. Measures of symptoms and disease status in ankylosing spondylitis: Ankylosing Spondylitis Disease Activity Score (ASDAS), Ankylosing Spondylitis Quality of Life Scale (ASQoL), Bath Ankylosing Spondylitis Disease Activity Index (BASDAI), Bath Ankylosing Spondylitis Functional Index (BASFI), Bath Ankylosing Spondylitis Global Score (BAS-G), Bath Ankylosing Spondylitis Metrology Index (BASMI), Dougados Functional Index (DFI), and Health Assessment Questionnaire for the Spondylarthropathies (HAQ-S). *Arthritis Care Res (Hoboken)* 2011; 63 Suppl 11:S47-S58.

(3) Romero-Diaz J, Isenberg D, Ramsey-Goldman R. Measures of adult systemic lupus erythematosus: updated version of British Isles Lupus Assessment Group (BILAG 2004), European Consensus Lupus Activity Measurements (ECLAM), Systemic Lupus Activity Measure, Revised (SLAM-R), Systemic Lupus Activity Questionnaire for Population Studies (SLAQ), Systemic Lupus Erythematosus Disease Activity Index 2000 (SLEDAI-2K), and Systemic Lupus International Collaborating Clinics/American College of Rheumatology Damage Index (SDI). *Arthritis Care Res (Hoboken)* 2011; 63 Suppl 11:S37-S46.

(4) Khanna D, Lovell DJ, Giannini E, Clements PJ, Merkel PA, Seibold JR et al. Development of a provisional core set of response measures for clinical trials of systemic sclerosis. *Ann Rheum Dis* 2008; 67(5):703-709.

(5) Taylor WJ, Schumacher HR, Jr., Singh JA, Grainger R, Dalbeth N. Assessment of outcome in clinical trials of gout--a review of current measures. *Rheumatology (Oxford)* 2007; 46(12):1751-1756.

(6) Dalbeth N, McQueen FM, Singh JA, MacDonald PA, Edwards NL, Schumacher HR, Jr. et al. Tophus measurement as an outcome measure for clinical trials of chronic gout: progress and research priorities. *J Rheumatol* 2011; 38(7):1458-1461.

(7) Andrews JS, Trupin L, Schmajuk G, Barton J, Margaretten M, Yazdany J et al. Muscle strength, muscle mass, and physical disability in women with systemic lupus erythematosus. *Arthritis Care Res (Hoboken)* 2015; 67(1):120-127.

(8) Alvarez-Hernandez E, Pelaez-Ballestas I, Vazquez-Mellado J, Teran-Estrada L, Bernard-Medina AG, Espinoza J et al. Validation of the Health Assessment Questionnaire disability index in patients with gout. *Arthritis Rheum* 2008; 59(5):665-669.

(9) OARSI. Outcome Measures [https://www.oarsi.org/research/outcome-measures]. 2013. 14-5-2018.
Ref Type: Online Source

(10) Singh JA, Taylor WJ, Simon LS, Khanna PP, Stamp LK, McQueen FM et al. Patient-reported outcomes in chronic gout: a report from OMERACT 10. *J Rheumatol* 2011; 38(7):1452-1457.

(11) Whalley D, McKenna SP, de JZ, van der Heijde D. Quality of life in rheumatoid arthritis. *Br J Rheumatol* 1997; 36(8):884-888.

(12) Doward LC, Spoorenberg A, Cook SA, Whalley D, Helliwell PS, Kay LJ et al. Development of the ASQoL: a quality of life instrument specific to ankylosing spondylitis. *Ann Rheum Dis* 2003; 62(1):20-26.

(13) McKenna SP, Doward LC, Whalley D, Tennant A, Emery P, Veale DJ. Development of the PsAQoL: a quality of life instrument specific to psoriatic arthritis. *Ann Rheum Dis* 2004; 63(2):162-169.

(14) Juhl C, Christensen R, Roos EM, Zhang W, Lund H. Impact of exercise type and dose on pain and disability in knee osteoarthritis: a systematic review and meta-regression analysis of randomized controlled trials. *Arthritis Rheumatol* 2014; 66(3):622-636.

(15) Corbett MS, Rice SJ, Madurasinghe V, Slack R, Fayter DA, Harden M et al. Acupuncture and other physical treatments for the relief of pain due to osteoarthritis of the knee: network meta-analysis. *Osteoarthritis Cartilage* 2013; 21(9):1290-1298.

(16) Tanaka R, Ozawa J, Kito N, Moriyama H. Efficacy of strengthening or aerobic exercise on pain relief in people with knee osteoarthritis: a systematic review and meta-analysis of randomized controlled trials. *Clin Rehabil* 2013; 27(12):1059-1071.

(17) Uthman OA, van der Windt DA, Jordan JL, Dziedzic KS, Healey EL, Peat GM et al. Exercise for lower limb osteoarthritis: systematic review incorporating trial sequential analysis and network meta-analysis. *BMJ* 2013; 347:f5555.

(18) Pozzobon D, Ferreira PH, Blyth FM, Machado GC, Ferreira ML. Can obesity and physical activity predict outcomes of elective knee or hip surgery due to osteoarthritis? A meta-analysis of cohort studies. *BMJ Open* 2018; 8(2):e017689.

(19) Wijnen A, Bouma SE, Seeber GH, van der Woude LHV, Bulstra SK, Lazovic D et al. The therapeutic validity and effectiveness of physiotherapeutic exercise following total hip arthroplasty for osteoarthritis: A systematic review. *PLoS One* 2018; 13(3):e0194517.

(20) Alrushud AS, Rushton AB, Kanavaki AM, Greig CA. Effect of physical activity and dietary restriction interventions on weight loss and the musculoskeletal function of overweight and obese older adults with knee osteoarthritis: a systematic review and mixed method data synthesis. *BMJ Open* 2017; 7(6):e014537.

(21) Brosseau L, Taki J, Desjardins B, Thevenot O, Fransen M, Wells GA et al. The Ottawa panel clinical practice guidelines for the management of knee osteoarthritis. Part three: aerobic exercise programs. *Clin Rehabil* 2017; 31(5):612-624.

(22) de Rooij M., van der Leeden M, Heymans MW, Holla JF, Hakkinen A, Lems WF et al. Prognosis of Pain and Physical Functioning in Patients With Knee Osteoarthritis: A Systematic Review and Meta-Analysis. *Arthritis Care Res (Hoboken)* 2016; 68(4):481-492.

(23) Bastick AN, Belo JN, Runhaar J, Bierma-Zeinstra SM. What Are the Prognostic Factors for Radiographic Progression of Knee Osteoarthritis? A Meta-analysis. *Clin Orthop Relat Res* 2015; 473(9):2969-2989.

(24) Le Quintrec JL, Verlhac B, Cadet C, Breville P, Vetel JM, Gauvain JB et al. Physical exercise and weight loss for hip and knee osteoarthritis in very old patients: a systematic review of the literature. *Open Rheumatol J* 2014; 8:89-95.

(25) Messier SP, Loeser RF, Miller GD, Morgan TM, Rejeski WJ, Sevick MA et al. Exercise and dietary weight loss in overweight and obese older adults with knee osteoarthritis: the Arthritis, Diet, and Activity Promotion Trial. *Arthritis Rheum* 2004; 50(5):1501-1510.

(26) Messier SP, Mihalko SL, Legault C, Miller GD, Nicklas BJ, DeVita P et al. Effects of intensive diet and exercise on knee joint loads, inflammation, and clinical outcomes among overweight and obese adults with knee osteoarthritis: the IDEA randomized clinical trial. *JAMA* 2013; 310(12):1263-1273.

(27) Beumer L, Wong J, Warden SJ, Kemp JL, Foster P, Crossley KM. Effects of exercise and manual therapy on pain associated with hip osteoarthritis: a systematic review and meta-analysis. *Br J Sports Med* 2016; 50(8):458-463.

(28) Bartels EM, Juhl CB, Christensen R, Hagen KB, Danneskiold-Samsoe B, Dagfinrud H et al. Aquatic exercise for the treatment of knee and hip osteoarthritis. *Cochrane Database Syst Rev* 2016; 3:CD005523.

(29) Lu M, Su Y, Zhang Y, Zhang Z, Wang W, He Z et al. Effectiveness of aquatic exercise for treatment of knee osteoarthritis: Systematic review and meta-analysis. *Z Rheumatol* 2015; 74(6):543-552.

(30) Waller B, Ogonowska-Slodownik A, Vitor M, Lambeck J, Daly D, Kujala UM et al. Effect of therapeutic aquatic exercise on symptoms and function associated with lower limb osteoarthritis: systematic review with meta-analysis. *Phys Ther* 2014; 94(10):1383-1395.

(31) McAlindon TE, Bannuru RR, Sullivan MC, Arden NK, Berenbaum F, Bierma-Zeinstra SM et al. OARSI guidelines for the non-surgical management of knee osteoarthritis. *Osteoarthritis Cartilage* 2014; 22(3):363-388.

(32) Romeo A, Parazza S, Boschi M, Nava T, Vanti C. Manual therapy and therapeutic exercise in the treatment of osteoarthritis of the hip: a systematic review. *Reumatismo* 2013; 65(2):63-74.

(33) Bartels EM, Lund H, Hagen KB, Dagfinrud H, Christensen R, Danneskiold-Samsoe B. Aquatic exercise for the treatment of knee and hip osteoarthritis. *Cochrane Database Syst Rev* 2007;(4):CD005523.

(34) Gay C, Chabaud A, Guilley E, Coudeyre E. Educating patients about the benefits of physical activity and exercise for their hip and knee osteoarthritis. Systematic literature review. *Ann Phys Rehabil Med* 2016; 59(3):174-183.

(35) Nelson AE, Allen KD, Golightly YM, Goode AP, Jordan JM. A systematic review of recommendations and guidelines for the management of osteoarthritis: The chronic osteoarthritis management initiative of the U.S. bone and joint initiative. *Semin Arthritis Rheum* 2014; 43(6):701-712.

(36) Fernandes L, Hagen KB, Bijlsma JW, Andreassen O, Christensen P, Conaghan PG et al. EULAR recommendations for the non-pharmacological core management of hip and knee osteoarthritis. *Ann Rheum Dis* 2013; 72(7):1125-1135.

(37) Regnaux JP, Lefevre-Colau MM, Trinquart L, Nguyen C, Boutron I, Brosseau L et al. High-intensity versus low-intensity physical activity or exercise in people with hip or knee osteoarthritis. *Cochrane Database Syst Rev* 2015;(10):CD010203.

(38) de Rooij M., van der Leeden M, Heymans MW, Holla JF, Hakkinen A, Lems WF et al. Course and predictors of pain and physical functioning in patients with hip osteoarthritis: Systematic review and meta-analysis. *J Rehabil Med* 2016; 48(3):245-252.

(39) Anwer S, Alghadir A, Brismee JM. Effect of Home Exercise Program in Patients With Knee Osteoarthritis: A Systematic Review and Meta-analysis. *J Geriatr Phys Ther* 2016; 39(1):38-48.

(40) Moseng T, Dagfinrud H, Smedslund G, Osteras N. The importance of dose in land-based supervised exercise for people with hip osteoarthritis. A systematic review and meta-analysis. *Osteoarthritis Cartilage* 2017; 25(10):1563-1576.

(41) Fernandopulle S, Perry M, Manlapaz D, Jayakaran P. Effect of Land-Based Generic Physical Activity Interventions on Pain, Physical Function, and Physical Performance in Hip and Knee Osteoarthritis: A Systematic Review and Meta-Analysis. *Am J Phys Med Rehabil* 2017; 96(11):773-792.

(42) Henriksen M, Hansen JB, Klokker L, Bliddal H, Christensen R. Comparable effects of exercise and analgesics for pain secondary to knee osteoarthritis: a meta-analysis of trials included in Cochrane systematic reviews. *J Comp Eff Res* 2016; 5(4):417-431.

(43) Fransen M, McConnell S, Harmer AR, Van der Esch M, Simic M, Bennell KL. Exercise for osteoarthritis of the knee. *Cochrane Database Syst Rev* 2015; 1:CD004376.

(44) Aebischer B, Elsig S, Taeymans J. Effectiveness of physical and occupational therapy on pain, function and quality of life in patients with trapeziometacarpal osteoarthritis - A systematic review and meta-analysis. *Hand Ther* 2016; 21(1):5-15.

(45) Finney A, Healey E, Jordan JL, Ryan S, Dziedzic KS. Multidisciplinary approaches to managing osteoarthritis in multiple joint sites: a systematic review. *BMC Musculoskelet Disord* 2016; 17:266.

(46) Bartholdy C, Juhl C, Christensen R, Lund H, Zhang W, Henriksen M. The role of muscle strengthening in exercise therapy for knee osteoarthritis: A systematic review and meta-regression analysis of randomized trials. *Semin Arthritis Rheum* 2017; 47(1):9-21.

(47) Magni NE, McNair PJ, Rice DA. The effects of resistance training on muscle strength, joint pain, and hand function in individuals with hand osteoarthritis: a systematic review and meta-analysis. *Arthritis Res Ther* 2017; 19(1):131.

(48) Osteras N, Kjeken I, Smedslund G, Moe RH, Slatkowsky-Christensen B, Uhlig T et al. Exercise for hand osteoarthritis. *Cochrane Database Syst Rev* 2017; 1:CD010388.

(49) Coudeyre E, Jegu AG, Giustanini M, Marrel JP, Edouard P, Pereira B. Isokinetic muscle strengthening for knee osteoarthritis: A systematic review of randomized controlled trials with meta-analysis. *Ann Phys Rehabil Med* 2016; 59(3):207-215.

(50) Li Y, Su Y, Chen S, Zhang Y, Zhang Z, Liu C et al. The effects of resistance exercise in patients with knee osteoarthritis: a systematic review and meta-analysis. *Clin Rehabil* 2016; 30(10):947-959.

(51) Brosseau L, Taki J, Desjardins B, Thevenot O, Fransen M, Wells GA et al. The Ottawa panel clinical practice guidelines for the management of knee osteoarthritis. Part two: strengthening exercise programs. *Clin Rehabil* 2017; 31(5):596-611.

(52) Brosseau L, Wells GA, Pugh AG, Smith CA, Rahman P, Alvarez Gallardo IC et al. Ottawa Panel evidence-based clinical practice guidelines for therapeutic exercise in the management of hip osteoarthritis. *Clin Rehabil* 2016; 30(10):935-946.

(53) Briani RV, Ferreira AS, Pazzinatto MF, Pappas E, De Oliveira SD, Azevedo FM. What interventions can improve quality of life or psychosocial factors of individuals with knee osteoarthritis? A systematic review with meta-analysis of primary outcomes from randomised controlled trials. *Br J Sports Med* 2018; 52(16):1031-1038.

(54) Hurley M, Dickson K, Hallett R, Grant R, Hauari H, Walsh N et al. Exercise interventions and patient beliefs for people with hip, knee or hip and knee osteoarthritis: a mixed methods review. *Cochrane Database Syst Rev* 2018; 4:CD010842.

(55) Sampath KK, Mani R, Miyamori T, Tumilty S. The effects of manual therapy or exercise therapy or both in people with hip osteoarthritis: a systematic review and meta-analysis. *Clin Rehabil* 2016; 30(12):1141-1155.

(56) Bertozzi L, Valdes K, Vanti C, Negrini S, Pillastrini P, Villafane JH. Investigation of the effect of conservative interventions in thumb carpometacarpal osteoarthritis: systematic review and meta-analysis. *Disabil Rehabil* 2015; 37(22):2025-2043.

(57) Desveaux L, Beauchamp M, Goldstein R, Brooks D. Community-based exercise programs as a strategy to optimize function in chronic disease: a systematic review. *Med Care* 2014; 52(3):216-226.

(58) Ferreira GE, Robinson CC, Wiebusch M, Viero CC, da Rosa LH, Silva MF. The effect of exercise therapy on knee adduction moment in individuals with knee osteoarthritis: A systematic review. *Clin Biomech (Bristol , Avon)* 2015; 30(6):521-527.

(59) Fehring TK, Fehring K, Odum SM, Halsey D. Physical therapy mandates by Medicare administrative contractors: effective or wasteful? *J Arthroplasty* 2013; 28(9):1459-1462.

(60) Zhang Y, Huang L, Su Y, Zhan Z, Li Y, Lai X. The Effects of Traditional Chinese Exercise in Treating Knee Osteoarthritis: A Systematic Review and Meta-Analysis. *PLoS One* 2017; 12(1):e0170237.

(61) Chen YW, Hunt MA, Campbell KL, Peill K, Reid WD. The effect of Tai Chi on four chronic conditions-cancer, osteoarthritis, heart failure and chronic obstructive pulmonary disease: a systematic review and meta-analyses. *Br J Sports Med* 2016; 50(7):397-407.

(62) Yan JH, Gu WJ, Sun J, Zhang WX, Li BW, Pan L. Efficacy of Tai Chi on pain, stiffness and function in patients with osteoarthritis: a meta-analysis. *PLoS One* 2013; 8(4):e61672.

(63) Brosseau L, Taki J, Desjardins B, Thevenot O, Fransen M, Wells GA et al. The Ottawa panel clinical practice guidelines for the management of knee osteoarthritis. Part one: introduction, and mind-body exercise programs. *Clin Rehabil* 2017; 31(5):582-595.

(64) Wang Y, Lu S, Wang R, Jiang P, Rao F, Wang B et al. Integrative effect of yoga practice in patients with knee arthritis: A PRISMA-compliant meta-analysis. *Medicine (Baltimore)* 2018; 97(31):e11742.

(65) Kan L, Zhang J, Yang Y, Wang P. The Effects of Yoga on Pain, Mobility, and Quality of Life in Patients with Knee Osteoarthritis: A Systematic Review. *Evid Based Complement Alternat Med* 2016; 2016:6016532.

(66) Cramer H, Lauche R, Langhorst J, Dobos G. Yoga for rheumatic diseases: a systematic review. *Rheumatology (Oxford)* 2013; 52(11):2025-2030.

(67) Rongen-van Dartel SA, Repping-Wuts H, Flendrie M, Bleijenberg G, Metsios GS, van den Hout WB et al. Effect of Aerobic Exercise Training on Fatigue in Rheumatoid Arthritis: A Meta-Analysis. *Arthritis Care Res (Hoboken)* 2015; 67(8):1054-1062.

(68) Hernandez-Hernandez MV, Diaz-Gonzalez F. Role of physical activity in the management and assessment of rheumatoid arthritis patients. *Reumatol Clin* 2017; 13(4):214-220.

(69) Siegel P, Tencza M, Apodaca B, Poole JL. Effectiveness of Occupational Therapy Interventions for Adults With Rheumatoid Arthritis: A Systematic Review. *Am J Occup Ther* 2017; 71(1):7101180050p1-7101180050p11.

(70) Larkin L, Kennedy N. Correlates of physical activity in adults with rheumatoid arthritis: a systematic review. *J Phys Act Health* 2014; 11(6):1248-1261.

(71) Katz P, Margaretten M, Gregorich S, Trupin L. Physical Activity to Reduce Fatigue in Rheumatoid Arthritis: A Randomized Controlled Trial. *Arthritis Care Res (Hoboken)* 2018; 70(1):1-10.

(72) Baxter SV, Hale LA, Stebbings S, Gray AR, Smith CM, Treharne GJ. Walking is a Feasible Physical Activity for People with Rheumatoid Arthritis: A Feasibility Randomized Controlled Trial. *Musculoskeletal Care* 2016; 14(1):47-56.

(73) Feldthusen C, Dean E, Forsblad-d'Elia H, Mannerkorpi K. Effects of Person-Centered Physical Therapy on Fatigue-Related Variables in Persons With Rheumatoid Arthritis: A Randomized Controlled Trial. *Arch Phys Med Rehabil* 2016; 97(1):26-36.

(74) Sjoquist ES, Brodin N, Lampa J, Jensen I, Opava CH. Physical activity coaching of patients with rheumatoid arthritis in everyday practice: a long-term follow-up. *Musculoskeletal Care* 2011; 9(2):75-85.

(75) Brodin N, Eurenius E, Jensen I, Nisell R, Opava CH. Coaching patients with early rheumatoid arthritis to healthy physical activity: a multicenter, randomized, controlled study. *Arthritis Rheum* 2008; 59(3):325-331.

(76) Li LC, Davis AM, Lineker SC, Coyte PC, Bombardier C. Effectiveness of the primary therapist model for rheumatoid arthritis rehabilitation: a randomized controlled trial. *Arthritis Rheum* 2006; 55(1):42-52.

(77) Melikoglu MA, Karatay S, Senel K, Akcay F. Association between dynamic exercise therapy and IGF-1 and IGFBP-3 concentrations in the patients with rheumatoid arthritis. *Rheumatol Int* 2006; 26(4):309-313.

(78) Hansen TM, Hansen G, Langgaard AM, Rasmussen JO. Longterm physical training in rheumatoid arthritis. A randomized trial with different training programs and blinded observers. *Scand J Rheumatol* 1993; 22(3):107-112.

(79) Nordstrom DC, Konttinen YT, Solovieva S, Friman C, Santavirta S. In- and out-patient rehabilitation in rheumatoid arthritis. A controlled, open, longitudinal, cost-effectiveness study. *Scand J Rheumatol* 1996; 25(4):200-206.

(80) Minor MA, Hewett JE. Physical fitness and work capacity in women with rheumatoid arthritis. *Arthritis Care Res* 1995; 8(3):146-154.

(81) Noreau L, Martineau H, Roy L, Belzile M. Effects of a modified dance-based exercise on cardiorespiratory fitness, psychological state and health status of persons with rheumatoid arthritis. *Am J Phys Med Rehabil* 1995; 74(1):19-27.

(82) Ekblom B, Lovgren O, Alderin M, Fridstrom M, Satterstrom G. Effect of short-term physical training on patients with rheumatoid arthritis. a six-month follow-up study. *Scand J Rheumatol* 1975; 4(2):87-91.

(83) Nadareishvili Z, Michaud K, Hallenbeck JM, Wolfe F. Cardiovascular, rheumatologic, and pharmacologic predictors of stroke in patients with rheumatoid arthritis: a nested, case-control study. *Arthritis Rheum* 2008; 59(8):1090-1096.

(84) Wolfe F, Michaud K. The risk of myocardial infarction and pharmacologic and nonpharmacologic myocardial infarction predictors in rheumatoid arthritis: a cohort and nested case-control analysis. *Arthritis Rheum* 2008; 58(9):2612-2621.

(85) Stenstrom CH. Radiologically observed progression of joint destruction and its relationship with demographic factors, disease severity, and exercise frequency in patients with rheumatoid arthritis. *Phys Ther* 1994; 74(1):32-39.

(86) Baillet A, Zeboulon N, Gossec L, Combescure C, Bodin LA, Juvin R et al. Efficacy of cardiorespiratory aerobic exercise in rheumatoid arthritis: meta-analysis of randomized controlled trials. *Arthritis Care Res (Hoboken)* 2010; 62(7):984-992.

(87) Wan X, Wang W, Liu J, Tong T. Estimating the sample mean and standard deviation from the sample size, median, range and/or interquartile range. *BMC Med Res Methodol* 2014; 14:135.

(88) Lange E, Kucharski D, Svedlund S, Svensson K, Bertholds G, Gjertsson I et al. Effects of Aerobic and Resistance Exercise in Older Adults With Rheumatoid Arthritis: A Randomized Controlled Trial. *Arthritis Care Res (Hoboken)* 2019; 71(1):61-70.

(89) Durcan L, Wilson F, Cunnane G. The effect of exercise on sleep and fatigue in rheumatoid arthritis: a randomized controlled study. *J Rheumatol* 2014; 41(10):1966-1973.

(90) Breedland I, van Scheppingen C., Leijsma M, Verheij-Jansen NP, van Weert E. Effects of a group-based exercise and educational program on physical performance and disease self-management in rheumatoid arthritis: a randomized controlled study. *Phys Ther* 2011; 91(6):879-893.

(91) Hurkmans EJ, van den Berg MH, Ronday KH, Peeters AJ, le CS, Vlieland TP. Maintenance of physical activity after Internet-based physical activity interventions in patients with rheumatoid arthritis. *Rheumatology (Oxford)* 2010; 49(1):167-172.

(92) van den Berg MH, Ronday HK, Peeters AJ, le CS, van der Giesen FJ, Breedveld FC et al. Using internet technology to deliver a home-based physical activity intervention for patients with rheumatoid arthritis: A randomized controlled trial. *Arthritis Rheum* 2006; 55(6):935-945.

(93) Flint-Wagner HG, Lisse J, Lohman TG, Going SB, Guido T, Cussler E et al. Assessment of a sixteen-week training program on strength, pain, and function in rheumatoid arthritis patients. *J Clin Rheumatol* 2009; 15(4):165-171.

(94) Bulthuis Y, Drossaers-Bakker KW, Taal E, Rasker J, Oostveen J, van't Pad BP et al. Arthritis patients show long-term benefits from 3 weeks intensive exercise training directly following hospital discharge. *Rheumatology (Oxford)* 2007; 46(11):1712-1717.

(95) Neuberger GB, Aaronson LS, Gajewski B, Embretson SE, Cagle PE, Loudon JK et al. Predictors of exercise and effects of exercise on symptoms, function, aerobic fitness, and disease outcomes of rheumatoid arthritis. *Arthritis Rheum* 2007; 57(6):943-952.

(96) Munneke M, de JZ, Zwinderman AH, Ronday HK, van SD, Dijkmans BA et al. Effect of a high-intensity weight-bearing exercise program on radiologic damage progression of the large joints in subgroups of patients with rheumatoid arthritis. *Arthritis Rheum* 2005; 53(3):410-417.

(97) de Jong Z., Munneke M, Lems WF, Zwinderman AH, Kroon HM, Pauwels EK et al. Slowing of bone loss in patients with rheumatoid arthritis by long-term high-intensity exercise: results of a randomized, controlled trial. *Arthritis Rheum* 2004; 50(4):1066-1076.

(98) de Jong Z., Munneke M, Zwinderman AH, Kroon HM, Ronday KH, Lems WF et al. Long term high intensity exercise and damage of small joints in rheumatoid arthritis. *Ann Rheum Dis* 2004; 63(11):1399-1405.

(99) Hakkinen A, Sokka T, Hannonen P. A home-based two-year strength training period in early rheumatoid arthritis led to good long-term compliance: a five-year followup. *Arthritis Rheum* 2004; 51(1):56-62.

(100) Hakkinen A, Sokka T, Kautiainen H, Kotaniemi A, Hannonen P. Sustained maintenance of exercise induced muscle strength gains and normal bone mineral density in patients with early rheumatoid arthritis: a 5 year follow up. *Ann Rheum Dis* 2004; 63(8):910-916.

(101) de Jong Z., Munneke M, Zwinderman AH, Kroon HM, Jansen A, Ronday KH et al. Is a long-term high-intensity exercise program effective and safe in patients with rheumatoid arthritis? Results of a randomized controlled trial. *Arthritis Rheum* 2003; 48(9):2415-2424.

(102) Westby MD, Wade JP, Rangno KK, Berkowitz J. A randomized controlled trial to evaluate the effectiveness of an exercise program in women with rheumatoid arthritis taking low dose prednisone. *J Rheumatol* 2000; 27(7):1674-1680.

(103) Hakkinen A, Sokka T, Kotaniemi A, Kautiainen H, Jappinen I, Laitinen L et al. Dynamic strength training in patients with early rheumatoid arthritis increases muscle strength but not bone mineral density. *J Rheumatol* 1999; 26(6):1257-1263.

(104) Lyngberg KK, Harreby M, Bentzen H, Frost B, Danneskiold-Samsoe B. Elderly rheumatoid arthritis patients on steroid treatment tolerate physical training without an increase in disease activity. *Arch Phys Med Rehabil* 1994; 75(11):1189-1195.

(105) Ekdahl C, Andersson SI, Moritz U, Svensson B. Dynamic versus static training in patients with rheumatoid arthritis. *Scand J Rheumatol* 1990; 19(1):17-26.

(106) Lyngberg K, Danneskiold-Samsoe B, Halskov O. The effect of physical training on patients with rheumatoid arthritis: changes in disease activity, muscle strength and aerobic capacity. A clinically controlled minimized cross-over study. *Clin Exp Rheumatol* 1988; 6(3):253-260.

(107) Nordemar R, Ekblom B, Zachrisson L, Lundqvist K. Physical training in rheumatoid arthritis: a controlled long-term study. I. *Scand J Rheumatol* 1981; 10(1):17-23.

(108) Nordemar R. Physical training in rheumatoid arthritis: A controlled long-term study. II. Functional capacity and general attitudes. *Scand J Rheumatol* 1981; 10(1):25-30.

(109) Stavropoulos-Kalinoglou A, Metsios GS, Veldhuijzen van Zanten JJ, Nightingale P, Kitas GD, Koutedakis Y. Individualised aerobic and resistance exercise training improves cardiorespiratory fitness and reduces cardiovascular risk in patients with rheumatoid arthritis. *Ann Rheum Dis* 2013; 72(11):1819-1825.

(110) Lofgren M, Opava CH, Demmelmaier I, Friden C, Lundberg IE, Nordgren B et al. Long-term, health-enhancing physical activity is associated with reduction of pain but not pain sensitivity or improved exercise-induced hypoalgesia in persons with rheumatoid arthritis. *Arthritis Res Ther* 2018; 20(1):262.

(111) Nordgren B, Friden C, Demmelmaier I, Bergstrom G, Lundberg IE, Dufour AB et al. An outsourced health-enhancing physical activity programme for people with rheumatoid arthritis: exploration of adherence and response. *Rheumatology (Oxford)* 2015; 54(6):1065-1073.

(112) Di Gioia L., Zincarelli C, Di Minno MN, Rengo G, Peluso R, Spano A et al. Effectiveness of a rehabilitative programme in improving fatigue and function in rheumatoid arthritis patients treated with biologics: a pilot study. *Clin Exp Rheumatol* 2013; 31(2):285-288.

(113) Strasser B, Leeb G, Strehblow C, Schobersberger W, Haber P, Cauza E. The effects of strength and endurance training in patients with rheumatoid arthritis. *Clin Rheumatol* 2011; 30(5):623-632.

(114) van der Giesen FJ, van LW, Hopman-Rock M, de JZ, Munneke M, Hazes JM et al. Exploring the public health impact of an intensive exercise program for patients with rheumatoid arthritis: a dissemination and implementation study. *Arthritis Care Res (Hoboken)* 2010; 62(6):865-872.

(115) de Jong Z., Munneke M, Kroon HM, van SD, Dijkmans BA, Hazes JM et al. Long-term follow-up of a high-intensity exercise program in patients with rheumatoid arthritis. *Clin Rheumatol* 2009; 28(6):663-671.

(116) Neuberger GB, Press AN, Lindsley HB, Hinton R, Cagle PE, Carlson K et al. Effects of exercise on fatigue, aerobic fitness, and disease activity measures in persons with rheumatoid arthritis. *Res Nurs Health* 1997; 20(3):195-204.

(117) Al-Qubaeissy KY, Fatoye FA, Goodwin PC, Yohannes AM. The effectiveness of hydrotherapy in the management of rheumatoid arthritis: a systematic review. *Musculoskeletal Care* 2013; 11(1):3-18.

(118) Siqueira US, Orsini Valente LG, de Mello MT, Szejnfeld VL, Pinheiro MM. Effectiveness of Aquatic Exercises in Women With Rheumatoid Arthritis: A Randomized, Controlled, 16-Week Intervention-The HydRA Trial. *Am J Phys Med Rehabil* 2017; 96(3):167-175.

(119) Eversden L, Maggs F, Nightingale P, Jobanputra P. A pragmatic randomised controlled trial of hydrotherapy and land exercises on overall well being and quality of life in rheumatoid arthritis. *BMC Musculoskelet Disord* 2007; 8:23.

(120) Bilberg A, Ahlmen M, Mannerkorpi K. Moderately intensive exercise in a temperate pool for patients with rheumatoid arthritis: a randomized controlled study. *Rheumatology (Oxford)* 2005; 44(4):502-508.

(121) Oldfield V, Felson DT. Exercise therapy and orthotic devices in rheumatoid arthritis: evidence-based review. *Curr Opin Rheumatol* 2008; 20(3):353-359.

(122) Lemmey AB, Williams SL, Marcora SM, Jones J, Maddison PJ. Are the benefits of a high-intensity progressive resistance training program sustained in rheumatoid arthritis patients? A 3-year followup study. *Arthritis Care Res (Hoboken)* 2012; 64(1):71-75.

(123) Lemmey AB, Marcora SM, Chester K, Wilson S, Casanova F, Maddison PJ. Effects of high-intensity resistance training in patients with rheumatoid arthritis: a randomized controlled trial. *Arthritis Rheum* 2009; 61(12):1726-1734.

(124) van den Ende CH, Breedveld FC, le CS, Dijkmans BA, de Mug AW, Hazes JM. Effect of intensive exercise on patients with active rheumatoid arthritis: a randomised clinical trial. *Ann Rheum Dis* 2000; 59(8):615-621.

(125) van den Ende CH, Hazes JM, le CS, Mulder WJ, Belfor DG, Breedveld FC et al. Comparison of high and low intensity training in well controlled rheumatoid arthritis. Results of a randomised clinical trial. *Ann Rheum Dis* 1996; 55(11):798-805.

(126) Hammond A, Prior Y. The effectiveness of home hand exercise programmes in rheumatoid arthritis: a systematic review. *Br Med Bull* 2016; 119(1):49-62.

(127) Zernicke J, Kedor C, Muller A, Burmester GR, Reisshauer A, Feist E. A prospective pilot study to evaluate an animated home-based physical exercise program as a treatment option for patients with rheumatoid arthritis. *BMC Musculoskelet Disord* 2016; 17(1):351.

(128) Seneca T, Hauge EM, Maribo T. Comparable effect of partly supervised and self-administered exercise programme in early rheumatoid arthritis--a randomised, controlled trial. *Dan Med J* 2015; 62(8):A5127.

(129) Hsieh LF, Chen SC, Chuang CC, Chai HM, Chen WS, He YC. Supervised aerobic exercise is more effective than home aerobic exercise in female chinese patients with rheumatoid arthritis. *J Rehabil Med* 2009; 41(5):332-337.

(130) Stenstrom CH. Home exercise in rheumatoid arthritis functional class II: goal setting versus pain attention. *J Rheumatol* 1994; 21(4):627-634.

(131) Crowley L. The effectiveness of home exercise programmes for patients with rheumatoid arthritis: A review of the literature. *Physical Therapy Reviews* 2009; 14:149-159.

(132) Williams MA, Srikesavan C, Heine PJ, Bruce J, Brosseau L, Hoxey-Thomas N et al. Exercise for rheumatoid arthritis of the hand. *Cochrane Database Syst Rev* 2018; 7:CD003832.

(133) Daien CI, Hua C, Combe B, Landewe R. Non-pharmacological and pharmacological interventions in patients with early arthritis: a systematic literature review informing the 2016 update of EULAR recommendations for the management of early arthritis. *RMD Open* 2017; 3(1):e000404.

(134) Bergstra SA, Murgia A, Te Velde AF, Caljouw SR. A systematic review into the effectiveness of hand exercise therapy in the treatment of rheumatoid arthritis. *Clin Rheumatol* 2014; 33(11):1539-1548.

(135) Lo CN, Xia G, Leung BP. The effect of nerve mobilization exercise in patients with rheumatoid arthritis: a pilot study. *Reumatismo* 2017; 69(3):111-118.

(136) Williamson E, McConkey C, Heine P, Dosanjh S, Williams M, Lamb SE. Hand exercises for patients with rheumatoid arthritis: an extended follow-up of the SARAH randomised controlled trial. *BMJ Open* 2017; 7(4):e013121.

(137) Lourenzi FM, Jones A, Pereira DF, Santos JHCA, Furtado RNV, Natour J. Effectiveness of an overall progressive resistance strength program for improving the functional capacity of patients with rheumatoid arthritis: a randomized controlled trial. *Clin Rehabil* 2017; 31(11):1482-1491.

(138) Lamb SE, Williamson EM, Heine PJ, Adams J, Dosanjh S, Dritsaki M et al. Exercises to improve function of the rheumatoid hand (SARAH): a randomised controlled trial. *Lancet* 2015; 385(9966):421-429.

(139) Manning VL, Hurley MV, Scott DL, Coker B, Choy E, Bearne LM. Education, self-management, and upper extremity exercise training in people with rheumatoid arthritis: a randomized controlled trial. *Arthritis Care Res (Hoboken)* 2014; 66(2):217-227.

(140) Dogu B, Sirzai H, Yilmaz F, Polat B, Kuran B. Effects of isotonic and isometric hand exercises on pain, hand functions, dexterity and quality of life in women with rheumatoid arthritis. *Rheumatol Int* 2013; 33(10):2625-2630.

(141) Baillet A, Payraud E, Niderprim VA, Nissen MJ, Allenet B, Francois P et al. A dynamic exercise programme to improve patients' disability in rheumatoid arthritis: a prospective randomized controlled trial. *Rheumatology (Oxford)* 2009; 48(4):410-415.

(142) Masiero S, Boniolo A, Wassermann L, Machiedo H, Volante D, Punzi L. Effects of an educational-behavioral joint protection program on people with moderate to severe rheumatoid arthritis: a randomized controlled trial. *Clin Rheumatol* 2007; 26(12):2043-2050.

(143) O'Brien AV, Jones P, Mullis R, Mulherin D, Dziedzic K. Conservative hand therapy treatments in rheumatoid arthritis--a randomized controlled trial. *Rheumatology (Oxford)* 2006; 45(5):577-583.

(144) Buljina AI, Taljanovic MS, Avdic DM, Hunter TB. Physical and exercise therapy for treatment of the rheumatoid hand. *Arthritis Rheum* 2001; 45(4):392-397.

(145) Scholten C, Brodowicz T, Graninger W, Gardavsky I, Pils K, Pesau B et al. Persistent functional and social benefit 5 years after a multidisciplinary arthritis training program. *Arch Phys Med Rehabil* 1999; 80(10):1282-1287.

(146) Bostrom C, Harms-Ringdahl K, Karreskog H, Nordemar R. Effects of static and dynamic shoulder rotator exercises in women with rheumatoid arthritis: a randomised comparison of impairment, disability, handicap, and health. *Scand J Rheumatol* 1998; 27(4):281-290.

(147) Komatireddy GR, Leitch RW, Cella K, Browning G, Minor M. Efficacy of low load resistive muscle training in patients with rheumatoid arthritis functional class II and III. *J Rheumatol* 1997; 24(8):1531-1539.

(148) Hoenig H, Groff G, Pratt K, Goldberg E, Franck W. A randomized controlled trial of home exercise on the rheumatoid hand. *J Rheumatol* 1993; 20(5):785-789.

(149) Dellhag B, Wollersjo I, Bjelle A. Effect of active hand exercise and wax bath treatment in rheumatoid arthritis patients. *Arthritis Care Res* 1992; 5(2):87-92.

(150) Marcora SM, Lemmey AB, Maddison PJ. Can progressive resistance training reverse cachexia in patients with rheumatoid arthritis? Results of a pilot study. *J Rheumatol* 2005; 32(6):1031-1039.

(151) Goksel Karatepe A., Gunaydin R, Turkmen G, Kaya T. Effects of home-based exercise program on the functional status and the quality of life in patients with rheumatoid arthritis: 1-year follow-up study. *Rheumatol Int* 2011; 31(2):171-176.

(152) Baillet A, Vaillant M, Guinot M, Juvin R, Gaudin P. Efficacy of resistance exercises in rheumatoid arthritis: meta-analysis of randomized controlled trials. *Rheumatology (Oxford)* 2012; 51(3):519-527.

(153) Wang C. Tai Chi improves pain and functional status in adults with rheumatoid arthritis: results of a pilot single-blinded randomized controlled trial. *Med Sport Sci* 2008; 52:218-229.

(154) Shin JH, Lee Y, Kim SG, Choi BY, Lee HS, Bang SY. The beneficial effects of Tai Chi exercise on endothelial function and arterial stiffness in elderly women with rheumatoid arthritis. *Arthritis Res Ther* 2015; 17:380.

(155) Lee HY, Hale CA, Hemingway B, Woolridge MW. Tai Chi exercise and auricular acupressure for people with rheumatoid arthritis: an evaluation study. *J Clin Nurs* 2012; 21(19-20):2812-2822.

(156) Uhlig T, Fongen C, Steen E, Christie A, Odegard S. Exploring Tai Chi in rheumatoid arthritis: a quantitative and qualitative study. *BMC Musculoskelet Disord* 2010; 11:43.

(157) Han A, Robinson V, Judd M, Taixiang W, Wells G, Tugwell P. Tai chi for treating rheumatoid arthritis. *Cochrane Database Syst Rev* 2004;(3):CD004849.

(158) Ward L, Stebbings S, Athens J, Cherkin D, David BG. Yoga for the management of pain and sleep in rheumatoid arthritis: a pilot randomized controlled trial. *Musculoskeletal Care* 2018; 16(1):39-47.

(159) Evans S, Moieni M, Lung K, Tsao J, Sternlieb B, Taylor M et al. Impact of iyengar yoga on quality of life in young women with rheumatoid arthritis. *Clin J Pain* 2013; 29(11):988-997.

(160) Singh VK, Bhandari RB, Rana BB. Effect of yogic package on rheumatoid arthritis. *Indian J Physiol Pharmacol* 2011; 55(4):329-335.

(161) Badsha H, Chhabra V, Leibman C, Mofti A, Kong KO. The benefits of yoga for rheumatoid arthritis: results of a preliminary, structured 8-week program. *Rheumatol Int* 2009; 29(12):1417-1421.

(162) Bosch PR, Traustadottir T, Howard P, Matt KS. Functional and physiological effects of yoga in women with rheumatoid arthritis: a pilot study. *Altern Ther Health Med* 2009; 15(4):24-31.

(163) O'Dwyer T, Durcan L, Wilson F. Exercise and physical activity in systemic lupus erythematosus: A systematic review with meta-analyses. *Semin Arthritis Rheum* 2017; 47(2):204-215.

(164) Wu ML, Yu KH, Tsai JC. The Effectiveness of Exercise in Adults With Systemic Lupus Erythematosus: A Systematic Review and Meta-Analysis to Guide Evidence-Based Practice. *Worldviews Evid Based Nurs* 2017; 14(4):306-315.

(165) Andrades C, Fuego C, Manrique-Arija S, Fernandez-Nebro A. Management of cardiovascular risk in systemic lupus erythematosus: a systematic review. *Lupus* 2017; 26(13):1407-1419.

(166) del Pino-Sedeno T, Trujillo-Martin MM, Ruiz-Irastorza G, Cuellar-Pompa L, de Pascual-Medina AM, Serrano-Aguilar P. Effectiveness of Nonpharmacologic Interventions for Decreasing Fatigue in Adults With Systemic Lupus Erythematosus: A Systematic Review. *Arthritis Care Res (Hoboken)* 2016; 68(1):141-148.

(167) Yuen HK, Cunningham MA. Optimal management of fatigue in patients with systemic lupus erythematosus: a systematic review. *Ther Clin Risk Manag* 2014; 10:775-786.

(168) Abrahao MI, Gomiero AB, Peccin MS, Grande AJ, Trevisani VF. Cardiovascular training vs. resistance training for improving quality of life and physical function in patients with systemic lupus erythematosus: a randomized controlled trial. *Scand J Rheumatol* 2016; 45(3):197-201.

(169) Bostrom C, Elfving B, Dupre B, Opava CH, Lundberg IE, Jansson E. Effects of a one-year physical activity programme for women with systemic lupus erythematosus - a randomized controlled study. *Lupus* 2016; 25(6):602-616.

(170) Tench CM, McCarthy J, McCurdie I, White PD, D'Cruz DP. Fatigue in systemic lupus erythematosus: a randomized controlled trial of exercise. *Rheumatology (Oxford)* 2003; 42(9):1050-1054.

(171) Robb-Nicholson LC, Daltroy L, Eaton H, Gall V, Wright E, Hartley LH et al. Effects of aerobic conditioning in lupus fatigue: a pilot study. *Br J Rheumatol* 1989; 28(6):500-505.

(172) Soriano-Maldonado A, Morillas-de-Laguno P, Sabio JM, Gavilan-Carrera B, Rosales-Castillo A, Montalban-Mendez C et al. Effects of 12-week Aerobic Exercise on Arterial Stiffness, Inflammation, and Cardiorespiratory Fitness in Women with Systemic LUPUS Erythematosus: Non-Randomized Controlled Trial. *J Clin Med* 2018; 7(12).

(173) dos Reis-Neto ET, da Silva AE, Monteiro CM, de Camargo LM, Sato EI. Supervised physical exercise improves endothelial function in patients with systemic lupus erythematosus. *Rheumatology (Oxford)* 2013; 52(12):2187-2195.

(174) Carvalho MR, Sato EI, Tebexreni AS, Heidecher RT, Schenkman S, Neto TL. Effects of supervised cardiovascular training program on exercise tolerance, aerobic capacity, and quality of life in patients with systemic lupus erythematosus. *Arthritis Rheum* 2005; 53(6):838-844.

(175) Bogdanovic G, Stojanovich L, Djokovic A, Stanisavljevic N. Physical Activity Program Is Helpful for Improving Quality of Life in Patients with Systemic Lupus Erythematosus. *Tohoku J Exp Med* 2015; 237(3):193-199.

(176) Miossi R, Benatti FB, Luciade de Sa PA, Lima FR, Borba EF, Prado DM et al. Using exercise training to counterbalance chronotropic incompetence and delayed heart rate recovery in systemic lupus erythematosus: a randomized trial. *Arthritis Care Res (Hoboken)* 2012; 64(8):1159-1166.

(177) Ramsey-Goldman R, Schilling EM, Dunlop D, Langman C, Greenland P, Thomas RJ et al. A pilot study on the effects of exercise in patients with systemic lupus erythematosus. *Arthritis Care Res* 2000; 13(5):262-269.

(178) Timoteo RP, Silva AF, Micheli DC, Candido Murta EF, Freire M, Teodoro RB et al. Increased flexibility, pain reduction and unaltered levels of IL-10 and CD11b + lymphocytes in patients with systemic lupus erythematosus were associated with kinesiotherapy. *Lupus* 2018; 27(7):1159-1168.

(179) Regel A, Sepriano A, Baraliakos X, van der Heijde D, Braun J, Landewe R et al. Efficacy and safety of non-pharmacological and non-biological pharmacological treatment: a systematic literature review informing the 2016 update of the ASAS/EULAR recommendations for the management of axial spondyloarthritis. *RMD Open* 2017; 3(1):e000397.

(180) O'Dwyer T, O'Shea F, Wilson F. Exercise therapy for spondyloarthritis: a systematic review. *Rheumatol Int* 2014; 34(7):887-902.

(181) Karahan AY, Tok F, Yildirim P, Ordahan B, Turkoglu G, Sahin N. The Effectiveness of Exergames in Patients with Ankylosing Spondylitis: A Randomized Controlled Trial. *Adv Clin Exp Med* 2016; 25(5):931-936.

(182) Jennings F, Oliveira HA, de Souza MC, Cruz VG, Natour J. Effects of Aerobic Training in Patients with Ankylosing Spondylitis. *J Rheumatol* 2015; 42(12):2347-2353.

(183) Niedermann K, Sidelnikov E, Muggli C, Dagfinrud H, Hermann M, Tamborrini G et al. Effect of cardiovascular training on fitness and perceived disease activity in people with ankylosing spondylitis. *Arthritis Care Res (Hoboken)* 2013; 65(11):1844-1852.

(184) Ajeganova S, Wornert M, Hafstrom I. A four-week team-rehabilitation programme in a warm climate decreases disability and improves health and body function for up to one year: A prospective study in Swedish patients with inflammatory joint diseases. *J Rehabil Med* 2016; 48(8):711-718.

(185) Sweeney S, Taylor G, Calin A. The effect of a home based exercise intervention package on outcome in ankylosing spondylitis: a randomized controlled trial. *J Rheumatol* 2002; 29(4):763-766.

(186) Brophy S, Cooksey R, Davies H, Dennis MS, Zhou SM, Siebert S. The effect of physical activity and motivation on function in ankylosing spondylitis: a cohort study. *Semin Arthritis Rheum* 2013; 42(6):619-626.

(187) Ward MM. Predictors of the progression of functional disability in patients with ankylosing spondylitis. *J Rheumatol* 2002; 29(7):1420-1425.

(188) Uhrin Z, Kuzis S, Ward MM. Exercise and changes in health status in patients with ankylosing spondylitis. *Arch Intern Med* 2000; 160(19):2969-2975.

(189) Pecourneau V, Degboe Y, Barnetche T, Cantagrel A, Constantin A, Ruyssen-Witrand A. Effectiveness of Exercise Programs in Ankylosing Spondylitis: A Meta-Analysis of Randomized Controlled Trials. *Arch Phys Med Rehabil* 2018; 99(2):383-389.

(190) Chang W, Tsou Y, Lee C. Comparison between specific exercises and physical therapy for managing patients with ankylosing spondylitis: A meta-analysis of randomized controlled trials. *International Journal of Clinical and Experimental Medicine* 2016; 9(9):17028-17039.

(191) Millner JR, Barron JS, Beinke KM, Butterworth RH, Chasle BE, Dutton LJ et al. Exercise for ankylosing spondylitis: An evidence-based consensus statement. *Semin Arthritis Rheum* 2016; 45(4):411-427.

(192) Liang H, Zhang H, Ji H, Wang C. Effects of home-based exercise intervention on health-related quality of life for patients with ankylosing spondylitis: a meta-analysis. *Clin Rheumatol* 2015; 34(10):1737-1744.

(193) Liang H, Li WR, Zhang H, Tian X, Wei W, Wang CM. Concurrent Intervention With Exercises and Stabilized Tumor Necrosis Factor Inhibitor Therapy Reduced the Disease Activity in Patients With Ankylosing Spondylitis: A Meta-Analysis. *Medicine (Baltimore)* 2015; 94(50):e2254.

(194) Martins NA, Furtado GE, Campos MJ, Leitao JC, Filaire E, Ferreira JP. Exercise and ankylosing spondylitis with New York modified criteria: a systematic review of controlled trials with meta-analysis. *Acta Reumatol Port* 2014; 39(4):298-308.

(195) Sharan D, Rajkumar JS. Physiotherapy for Ankylosing Spondylitis: Systematic Review and a Proposed Rehabilitation Protocol. *Curr Rheumatol Rev* 2017; 13(2):121-125.

(196) Sveaas SH, Bilberg A, Berg IJ, Provan SA, Rollefstad S, Semb AG et al. High intensity exercise for 3 months reduces disease activity in axial spondyloarthritis (axSpA): a multicentre randomised trial of 100 patients. *Br J Sports Med* 2019.

(197) Sveaas SH, Berg IJ, Fongen C, Provan SA, Dagfinrud H. High-intensity cardiorespiratory and strength exercises reduced emotional distress and fatigue in patients with axial spondyloarthritis: a randomized controlled pilot study. *Scand J Rheumatol* 2018; 47(2):117-121.

(198) Aydin T, Taspinar O, Sariyildiz MA, Guneser M, Keskin Y, Canbaz N et al. Evaluation of the effectiveness of home based or hospital based calisthenic exercises in patients with ankylosing spondylitis. *J Back Musculoskelet Rehabil* 2016; 29(4):723-730.

(199) Rosu MO, Topa I, Chirieac R, Ancuta C. Effects of Pilates, McKenzie and Heckscher training on disease activity, spinal motility and pulmonary function in patients with ankylosing spondylitis: a randomized controlled trial. *Rheumatol Int* 2014; 34(3):367-372.

(200) Sveaas SH, Berg IJ, Provan SA, Semb AG, Hagen KB, Vollestad N et al. Efficacy of high intensity exercise on disease activity and cardiovascular risk in active axial spondyloarthritis: a randomized controlled pilot study. *PLoS One* 2014; 9(9):e108688.

(201) Kjeken I, Bo I, Ronningen A, Spada C, Mowinckel P, Hagen KB et al. A three-week multidisciplinary in-patient rehabilitation programme had positive long-term effects in patients with ankylosing spondylitis: randomized controlled trial. *J Rehabil Med* 2013; 45(3):260-267.

(202) Analay Y, Ozcan E, Karan A, Diracoglu D, Aydin R. The effectiveness of intensive group exercise on patients with ankylosing spondylitis. *Clin Rehabil* 2003; 17(6):631-636.

(203) Hidding A, van der Linden S, Gielen X, de WL, Dijkmans B, Moolenburgh D. Continuation of group physical therapy is necessary in ankylosing spondylitis: results of a randomized controlled trial. *Arthritis Care Res* 1994; 7(2):90-96.

(204) Hidding A, van der Linden S, Boers M, Gielen X, de WL, Kester A et al. Is group physical therapy superior to individualized therapy in ankylosing spondylitis? A randomized controlled trial. *Arthritis Care Res* 1993; 6(3):117-125.

(205) Kraag G, Stokes B, Groh J, Helewa A, Goldsmith C. The effects of comprehensive home physiotherapy and supervision on patients with ankylosing spondylitis--a randomized controlled trial. *J Rheumatol* 1990; 17(2):228-233.

(206) Levitova A, Hulejova H, Spiritovic M, Pavelka K, Senolt L, Husakova M. Clinical improvement and reduction in serum calprotectin levels after an intensive exercise programme for patients with ankylosing spondylitis and non-radiographic axial spondyloarthritis. *Arthritis Res Ther* 2016; 18(1):275.

(207) Aytekin E, Caglar NS, Ozgonenel L, Tutun S, Demiryontar DY, Demir SE. Home-based exercise therapy in patients with ankylosing spondylitis: effects on pain, mobility, disease activity, quality of life, and respiratory functions. *Clin Rheumatol* 2012; 31(1):91-97.

(208) Viitanen JV, Heikkila S. Functional changes in patients with spondylarthropathy. A controlled trial of the effects of short-term rehabilitation and 3-year follow-up. *Rheumatol Int* 2001; 20(5):211-214.

(209) Lubrano E, D'Angelo S, Parsons WJ, Corbi G, Ferrara N, Rengo F et al. Effectiveness of rehabilitation in active ankylosing spondylitis assessed by the ASAS response criteria. *Rheumatology (Oxford)* 2007; 46(11):1672-1675.

(210) Band DA, Jones SD, Kennedy LG, Garrett SL, Porter J, Gay L et al. Which patients with ankylosing spondylitis derive most benefit from an inpatient management program? *J Rheumatol* 1997; 24(12):2381-2384.

(211) Viitanen JV, Lehtinen K, Suni J, Kautiainen H. Fifteen months' follow-up of intensive inpatient physiotherapy and exercise in ankylosing spondylitis. *Clin Rheumatol* 1995; 14(4):413-419.

(212) Kraag G, Stokes B, Groh J, Helewa A, Goldsmith CH. The effects of comprehensive home physiotherapy and supervision on patients with ankylosing spondylitis--an 8-month followup. *J Rheumatol* 1994; 21(2):261-263.

(213) Viitanen JV, Suni J, Kautiainen H, Liimatainen M, Takala H. Effect of physiotherapy on spinal mobility in ankylosing spondylitis. *Scand J Rheumatol* 1992; 21(1):38-41.

(214) Escalas C, Dalichampt M, Dougados M, Poiraudeau S. Evaluation of physiotherapy in a prospective cohort of early axial spondyloarthritis. Data from the DESIR cohort. *Joint Bone Spine* 2016; 83(2):185-190.

(215) Zao A, Cantista P. The role of land and aquatic exercise in ankylosing spondylitis: a systematic review. *Rheumatol Int* 2017; 37(12):1979-1990.

(216) Dundar U, Solak O, Toktas H, Demirdal US, Subasi V, Kavuncu V et al. Effect of aquatic exercise on ankylosing spondylitis: a randomized controlled trial. *Rheumatol Int* 2014; 34(11):1505-1511.

(217) Karapolat H, Eyigor S, Zoghi M, Akkoc Y, Kirazli Y, Keser G. Are swimming or aerobic exercise better than conventional exercise in ankylosing spondylitis patients? A randomized controlled study. *Eur J Phys Rehabil Med* 2009; 45(4):449-457.

(218) Hsieh LF, Chuang CC, Tseng CS, Wei JC, Hsu WC, Lin YJ. Combined home exercise is more effective than range-of-motion home exercise in patients with ankylosing spondylitis: a randomized controlled trial. *Biomed Res Int* 2014; 2014:398190.

(219) Rodriguez-Lozano C, Juanola X, Cruz-Martinez J, Pena-Arrebola A, Mulero J, Gratacos J et al. Outcome of an education and home-based exercise programme for patients with ankylosing spondylitis: a nationwide randomized study. *Clin Exp Rheumatol* 2013; 31(5):739-748.

(220) Lim HJ, Moon YI, Lee MS. Effects of home-based daily exercise therapy on joint mobility, daily activity, pain, and depression in patients with ankylosing spondylitis. *Rheumatol Int* 2005; 25(3):225-229.

(221) Yigit S, Sahin Z, Demir SE, Aytac DH. Home-based exercise therapy in ankylosing spondylitis: short-term prospective study in patients receiving tumor necrosis factor alpha inhibitors. *Rheumatol Int* 2013; 33(1):71-77.

(222) Durmus D, Alayli G, Cil E, Canturk F. Effects of a home-based exercise program on quality of life, fatigue, and depression in patients with ankylosing spondylitis. *Rheumatol Int* 2009; 29(6):673-677.

(223) Durmus D, Alayli G, Uzun O, Tander B, Canturk F, Bek Y et al. Effects of two exercise interventions on pulmonary functions in the patients with ankylosing spondylitis. *Joint Bone Spine* 2009; 76(2):150-155.

(224) Karapolat H, Akkoc Y, Sari I, Eyigor S, Akar S, Kirazli Y et al. Comparison of group-based exercise versus home-based exercise in patients with ankylosing spondylitis: effects on Bath Ankylosing Spondylitis Indices, quality of life and depression. *Clin Rheumatol* 2008; 27(6):695-700.

(225) de Souza MC, Jennings F, Morimoto H, Natour J. Swiss ball exercises improve muscle strength and walking performance in ankylosing spondylitis: a randomized controlled trial. *Rev Bras Reumatol Engl Ed* 2017; 57(1):45-55.

(226) Kasapoglu Aksoy M., Birtane M, Tastekin N, Ekuklu G. The Effectiveness of Structured Group Education on Ankylosing Spondylitis Patients. *J Clin Rheumatol* 2017; 23(3):138-143.

(227) Rosu OM, Ancuta C. McKenzie training in patients with early stages of ankylosing spondylitis: results of a 24-week controlled study. *Eur J Phys Rehabil Med* 2015; 51(3):261-268.

(228) Masiero S, Poli P, Bonaldo L, Pigatto M, Ramonda R, Lubrano E et al. Supervised training and home-based rehabilitation in patients with stabilized ankylosing spondylitis on TNF inhibitor treatment: a controlled clinical trial with a 12-month follow-up. *Clin Rehabil* 2014; 28(6):562-572.

(229) Masiero S, Bonaldo L, Pigatto M, Lo NA, Ramonda R, Punzi L. Rehabilitation treatment in patients with ankylosing spondylitis stabilized with tumor necrosis factor inhibitor therapy: a randomized controlled trial. *J Rheumatol* 2011; 38(7):1335-1342.

(230) Altan L, Korkmaz N, Dizdar M, Yurtkuran M. Effect of Pilates training on people with ankylosing spondylitis. *Rheumatol Int* 2012; 32(7):2093-2099.

(231) Fernandez-de-Las-Penas C, Alonso-Blanco C, Alguacil-Diego IM, Miangolarra-Page JC. One-year follow-up of two exercise interventions for the management of patients with ankylosing spondylitis: a randomized controlled trial. *Am J Phys Med Rehabil* 2006; 85(7):559-567.

(232) Fernandez-de-Las-Penas C, Alonso-Blanco C, Morales-Cabezas M, Miangolarra-Page JC. Two exercise interventions for the management of patients with ankylosing spondylitis: a randomized controlled trial. *Am J Phys Med Rehabil* 2005; 84(6):407-419.

(233) Gyurcsik ZN, Andras A, Bodnar N, Szekanecz Z, Szanto S. Improvement in pain intensity, spine stiffness, and mobility during a controlled individualized physiotherapy program in ankylosing spondylitis. *Rheumatol Int* 2012; 32(12):3931-3936.

(234) Hulejova H, Levitova A, Kuklova M, Stochl J, Haluzik M, Pavelka K et al. No effect of physiotherapy on the serum levels of adipocytokines in patients with ankylosing spondylitis. *Clin Rheumatol* 2012; 31(1):67-71.

(235) Ortancil O, Sarikaya S, Sapmaz P, Basaran A, Ozdolap S. The effect(s) of a six-week home-based exercise program on the respiratory muscle and functional status in ankylosing spondylitis. *J Clin Rheumatol* 2009; 15(2):68-70.

(236) Roger-Silva D, Natour J, Moreira E, Jennings F. A resistance exercise program improves functional capacity of patients with psoriatic arthritis: a randomized controlled trial. *Clin Rheumatol* 2018; 37(2):389-395.

(237) Chimenti MS, Triggianese P, Conigliaro P, Santoro M, Lucchetti R, Perricone R. Self-reported adherence to a home-based exercise program among patients affected by psoriatic arthritis with minimal disease activity. *Drug Dev Res* 2014; 75 Suppl 1:S57-S59.

(238) Antonioli CM, Bua G, Frige A, Prandini K, Radici S, Scarsi M et al. An individualized rehabilitation program in patients with systemic sclerosis may improve quality of life and hand mobility. *Clin Rheumatol* 2009; 28(2):159-165.

(239) Moran ME. Scleroderma and evidence based non-pharmaceutical treatment modalities for digital ulcers: a systematic review. *J Wound Care* 2014; 23(10):510-516.

(240) Rannou F, Boutron I, Mouthon L, Sanchez K, Tiffreau V, Hachulla E et al. Personalized Physical Therapy Versus Usual Care for Patients With Systemic Sclerosis: A Randomized Controlled Trial. *Arthritis Care Res (Hoboken)* 2017; 69(7):1050-1059.

(241) Schouffoer AA, Ninaber MK, Beaart-van de Voorde LJ, van der Giesen FJ, de JZ, Stolk J et al. Randomized comparison of a multidisciplinary team care program with usual care in patients with systemic sclerosis. *Arthritis Care Res (Hoboken)* 2011; 63(6):909-917.

(242) Pinto AL, Oliveira NC, Gualano B, Christmann RB, Painelli VS, Artioli GG et al. Efficacy and safety of concurrent training in systemic sclerosis. *J Strength Cond Res* 2011; 25(5):1423-1428.

(243) Maddali Bongi S, Del Rosso A, alluccio F, ai G, igismondi F, assalacqua M et al. Efficacy of a tailored rehabilitation program for systemic sclerosis. *Clin Exp Rheumatol* 2009; 27(3 Suppl 54):44-50.

(244) Stefanantoni K, Sciarra I, Iannace N, Vasile M, Caucci M, Sili SA et al. Occupational therapy integrated with a self-administered stretching program on systemic sclerosis patients with hand involvement. *Clin Exp Rheumatol* 2016; 34 Suppl 100(5):157-161.

(245) Horvath J, Balint Z, Szep E, Deiszinger A, Minier T, Farkas N et al. Efficacy of intensive hand physical therapy in patients with systemic sclerosis. *Clin Exp Rheumatol* 2017; 35 Suppl 106(4):159-166.

(246) Mugii N, Matsushita T, Oohata S, Okita H, Yahata T, Someya F et al. Long-term follow-up of finger passive range of motion in Japanese systemic sclerosis patients treated with self-administered stretching. *Mod Rheumatol* 2019; 29(3):484-490.

(247) Landim SF, Bertolo MB, Marcatto de Abreu MF, Del Rio AP, Mazon CC, Marques-Neto JF et al. The evaluation of a home-based program for hands in patients with systemic sclerosis. *J Hand Ther* 2017.

(248) Mugii N, Hasegawa M, Matsushita T, Kondo M, Orito H, Yanaba K et al. The efficacy of self-administered stretching for finger joint motion in Japanese patients with systemic sclerosis. *J Rheumatol* 2006; 33(8):1586-1592.

(249) Ma L, Sun R, Jia Z, Zou Y, Xin Y, Cheng X et al. Clinical characteristics associated with subcutaneous tophi formation in Chinese gout patients: a retrospective study. *Clin Rheumatol* 2018; 37(5):1359-1365.

(250) Adithya Acharya K, Sharma A. Evaluation of the efficacy of siravyadha and guduchi siddha yoga basti in the management of vatarakta with special reference to gout. *International Journal of Reserach in Ayuveda and Pharmacy* 2019; 4(3):402-409.

(251) Sadeghi A, Rad ZA, Sajedi B, Heydari AH, Akbarieh S, Jafari B. Effect of weight losing on the clinical status improvement of patients with knee osteoarthritis. *Reumatol Clin* 2019; 15(2):73-76.

(252) O'Brien KM, Wiggers J, Williams A, Campbell E, Hodder RK, Wolfenden L et al. Telephone-based weight loss support for patients with knee osteoarthritis: a pragmatic randomised controlled trial. *Osteoarthritis Cartilage* 2018; 26(4):485-494.

(253) Allen KD, Oddone EZ, Coffman CJ, Jeffreys AS, Bosworth HB, Chatterjee R et al. Patient, Provider, and Combined Interventions for Managing Osteoarthritis in Primary Care: A Cluster Randomized Trial. *Ann Intern Med* 2017; 166(6):401-411.

(254) Allen KD, Yancy WS, Jr., Bosworth HB, Coffman CJ, Jeffreys AS, Datta SK et al. A Combined Patient and Provider Intervention for Management of Osteoarthritis in Veterans: A Randomized Clinical Trial. *Ann Intern Med* 2016; 164(2):73-83.

(255) Christensen R, Henriksen M, Leeds AR, Gudbergsen H, Christensen P, Sorensen TJ et al. Effect of weight maintenance on symptoms of knee osteoarthritis in obese patients: a twelve-month randomized controlled trial. *Arthritis Care Res (Hoboken)* 2015; 67(5):640-650.

(256) Hunter DJ, Beavers DP, Eckstein F, Guermazi A, Loeser RF, Nicklas BJ et al. The Intensive Diet and Exercise for Arthritis (IDEA) trial: 18-month radiographic and MRI outcomes. *Osteoarthritis Cartilage* 2015; 23(7):1090-1098.

(257) Saraboon Y, Aree-Ue S, Maruo SJ. The Effect of Multifactorial Intervention Programs on Health Behavior and Symptom Control Among Community-Dwelling Overweight Older Adults With Knee Osteoarthritis. *Orthop Nurs* 2015; 34(5):296-308.

(258) Beavers DP, Beavers KM, Loeser RF, Walton NR, Lyles MF, Nicklas BJ et al. The independent and combined effects of intensive weight loss and exercise training on bone mineral density in overweight and obese older adults with osteoarthritis. *Osteoarthritis Cartilage* 2014; 22(6):726-733.

(259) Henriksen M, Christensen R, Hunter DJ, Gudbergsen H, Boesen M, Lohmander LS et al. Structural changes in the knee during weight loss maintenance after a significant weight loss in obese patients with osteoarthritis: a report of secondary outcome analyses from a randomized controlled trial. *Osteoarthritis Cartilage* 2014; 22(5):639-646.

(260) Somers TJ, Blumenthal JA, Guilak F, Kraus VB, Schmitt DO, Babyak MA et al. Pain coping skills training and lifestyle behavioral weight management in patients with knee osteoarthritis: a randomized controlled study. *Pain* 2012; 153(6):1199-1209.

(261) Bliddal H, Leeds AR, Stigsgaard L, Astrup A, Christensen R. Weight loss as treatment for knee osteoarthritis symptoms in obese patients: 1-year results from a randomised controlled trial. *Ann Rheum Dis* 2011; 70(10):1798-1803.

(262) Christensen R, Astrup A, Bliddal H. Weight loss: the treatment of choice for knee osteoarthritis? A randomized trial. *Osteoarthritis Cartilage* 2005; 13(1):20-27.

(263) Gudbergsen H, Boesen M, Christensen R, Astrup A, Bliddal H. Radiographs and low field MRI (0.2T) as predictors of efficacy in a weight loss trial in obese women with knee osteoarthritis. *BMC Musculoskelet Disord* 2011; 12:56.

(264) Riecke BF, Christensen R, Christensen P, Leeds AR, Boesen M, Lohmander LS et al. Comparing two low-energy diets for the treatment of knee osteoarthritis symptoms in obese patients: a pragmatic randomized clinical trial. *Osteoarthritis Cartilage* 2010; 18(6):746-754.

(265) Ravaud P, Flipo RM, Boutron I, Roy C, Mahmoudi A, Giraudeau B et al. ARTIST (osteoarthritis intervention standardized) study of standardised consultation versus usual care for patients with osteoarthritis of the knee in primary care in France: pragmatic randomised controlled trial. *BMJ* 2009; 338:b421.

(266) Miller GD, Nicklas BJ, Loeser RF. Inflammatory biomarkers and physical function in older, obese adults with knee pain and self-reported osteoarthritis after intensive weight-loss therapy. *J Am Geriatr Soc* 2008; 56(4):644-651.

(267) Miller GD, Nicklas BJ, Davis C, Loeser RF, Lenchik L, Messier SP. Intensive weight loss program improves physical function in older obese adults with knee osteoarthritis. *Obesity (Silver Spring)* 2006; 14(7):1219-1230.

(268) Rejeski WJ, Focht BC, Messier SP, Morgan T, Pahor M, Penninx B. Obese, older adults with knee osteoarthritis: weight loss, exercise, and quality of life. *Health Psychol* 2002; 21(5):419-426.

(269) Messier SP, Loeser RF, Mitchell MN, Valle G, Morgan TP, Rejeski WJ et al. Exercise and weight loss in obese older adults with knee osteoarthritis: a preliminary study. *J Am Geriatr Soc* 2000; 48(9):1062-1072.

(270) Toda Y. The effect of energy restriction, walking, and exercise on lower extremity lean body mass in obese women with osteoarthritis of the knee. *J Orthop Sci* 2001; 6(2):148-154.

(271) Huang MH, Chen CH, Chen TW, Weng MC, Wang WT, Wang YL. The effects of weight reduction on the rehabilitation of patients with knee osteoarthritis and obesity. *Arthritis Care Res* 2000; 13(6):398-405.

(272) Bartholdy C, Christensen R, Kristensen LE, Gudbergsen H, Bliddal H, Overgaard A et al. Association between weight loss and spontaneous changes in physical inactivity in overweight/obese individuals with knee osteoarthritis: an 8-week prospective cohort study. *Arthritis Care Res (Hoboken)* 2019.

(273) Aree-Ue S, Saraboon Y, Belza B. Long-Term Adherence and Effectiveness of a Multicomponent Intervention for Community-Dwelling Overweight Thai Older Adults with Knee Osteoarthritis: 1-Year Follow Up. *J Gerontol Nurs* 2017; 43(4):40-48.

(274) Atukorala I, Makovey J, Lawler L, Messier SP, Bennell K, Hunter DJ. Is There a Dose-Response Relationship Between Weight Loss and Symptom Improvement in Persons With Knee Osteoarthritis? *Arthritis Care Res (Hoboken)* 2016; 68(8):1106-1114.

(275) Bartels EM, Christensen R, Christensen P, Henriksen M, Bennett A, Gudbergsen H et al. Effect of a 16 weeks weight loss program on osteoarthritis biomarkers in obese patients with knee osteoarthritis: a prospective cohort study. *Osteoarthritis Cartilage* 2014; 22(11):1817-1825.

(276) Paans N, van dA-S, I, Dilling RG, Bos M, van der Meer K, Bulstra SK et al. Effect of exercise and weight loss in people who have hip osteoarthritis and are overweight or obese: a prospective cohort study. *Phys Ther* 2013; 93(2):137-146.

(277) Gudbergsen H, Boesen M, Lohmander LS, Christensen R, Henriksen M, Bartels EM et al. Weight loss is effective for symptomatic relief in obese subjects with knee osteoarthritis independently of joint damage severity assessed by high-field MRI and radiography. *Osteoarthritis Cartilage* 2012; 20(6):495-502.

(278) Bihlet AR, Byrjalsen I, Bay-Jensen AC, Andersen JR, Christiansen C, Riis BJ et al. Identification of pain categories associated with change in pain in patients receiving placebo: data from two phase 3 randomized clinical trials in symptomatic knee osteoarthritis. *BMC Musculoskelet Disord* 2018; 19(1):17.

(279) Han A, Gellhorn AC. Trajectories of Quality of Life and Associated Risk Factors in Patients With Knee Osteoarthritis: Findings From the Osteoarthritis Initiative. *Am J Phys Med Rehabil* 2018; 97(9):620-627.

(280) Jacobs CA, Vranceanu AM, Thompson KL, Lattermann C. Rapid Progression of Knee Pain and Osteoarthritis Biomarkers Greatest for Patients with Combined Obesity and Depression: Data from the Osteoarthritis Initiative. *Cartilage* 2018;1947603518777577.

(281) Pelletier JP, Raynauld JP, Abram F, Dorais M, Delorme P, Martel-Pelletier J. Exploring determinants predicting response to intra-articular hyaluronic acid treatment in symptomatic knee osteoarthritis: 9-year follow-up data from the Osteoarthritis Initiative. *Arthritis Res Ther* 2018; 20(1):40.

(282) Eymard F, Chevalier X, Conrozier T. Obesity and radiological severity are associated with viscosupplementation failure in patients with knee osteoarthritis. *J Orthop Res* 2017; 35(10):2269-2274.

(283) Moyer R, Wirth W, Eckstein F. Longitudinal Changes in Magnetic Resonance Imaging-Based Measures of Femorotibial Cartilage Thickness as a Function of Alignment and Obesity: Data From the Osteoarthritis Initiative. *Arthritis Care Res (Hoboken)* 2017; 69(7):959-965.

(284) Bastick AN, Verkleij SP, Damen J, Wesseling J, Hilberdink WK, Bindels PJ et al. Defining hip pain trajectories in early symptomatic hip osteoarthritis--5 year results from a nationwide prospective cohort study (CHECK). *Osteoarthritis Cartilage* 2016; 24(5):768-775.

(285) de Rezende MU, Hissadomi MI, de Campos GC, Frucchi R, Pailo AF, Pasqualin T et al. One-Year Results of an Educational Program on Osteoarthritis: A Prospective Randomized Controlled Trial in Brazil. *Geriatr Orthop Surg Rehabil* 2016; 7(2):86-94.

(286) Beavers KM, Beavers DP, Newman JJ, Anderson AM, Loeser RF, Jr., Nicklas BJ et al. Effects of total and regional fat loss on plasma CRP and IL-6 in overweight and obese, older adults with knee osteoarthritis. *Osteoarthritis Cartilage* 2015; 23(2):249-256.

(287) Chatterjee D, McGee A, Strauss E, Youm T, Jazrawi L. Subchondral Calcium Phosphate is Ineffective for Bone Marrow Edema Lesions in Adults With Advanced Osteoarthritis. *Clin Orthop Relat Res* 2015; 473(7):2334-2342.

(288) Karsdal MA, Bihlet A, Byrjalsen I, Alexandersen P, Ladel C, Michaels M et al. OA phenotypes, rather than disease stage, drive structural progression--identification of structural progressors from 2 phase III randomized clinical studies with symptomatic knee OA. *Osteoarthritis Cartilage* 2015; 23(4):550-558.

(289) Kobayashi N, Inaba Y, Yukizawa Y, Ike H, Kubota S, Inoue T et al. Use of 18F-fluoride positron emission tomography as a predictor of the hip osteoarthritis progression. *Mod Rheumatol* 2015; 25(6):925-930.

(290) Magnusson K, Slatkowsky-Christensen B, van der Heijde D, Kvien TK, Hagen KB, Haugen IK. Body mass index and progressive hand osteoarthritis: data from the Oslo hand osteoarthritis cohort. *Scand J Rheumatol* 2015; 44(4):331-336.

(291) Gudbergsen H, Boesen M, Christensen R, Bartels EM, Henriksen M, Danneskiold-Samsoe B et al. Changes in bone marrow lesions in response to weight-loss in obese knee osteoarthritis patients: a prospective cohort study. *BMC Musculoskelet Disord* 2013; 14:106.

(292) Perrot S, Bertin P. "Feeling better" or "feeling well" in usual care of hip and knee osteoarthritis pain: determination of cutoff points for patient acceptable symptom state (PASS) and minimal clinically important improvement (MCII) at rest and on movement in a national multicenter cohort study of 2414 patients with painful osteoarthritis. *Pain* 2013; 154(2):248-256.

(293) Coffman C, Allen K, Woolson R. Mixed-effects regression modeling of real-time momentary pain assessments in osteoarthritis (OA) patients. *Health Service and Outcomes Research Methodology* 2012; 12(2-3):200-218.

(294) Miyazaki T, Uchida K, Sato M, Watanabe S, Yoshida A, Wada M et al. Knee laxity after staircase exercise predicts radiographic disease progression in medial compartment knee osteoarthritis. *Arthritis Rheum* 2012; 64(12):3908-3916.

(295) Rabago D, Zgierska A, Fortney L, Kijowski R, Mundt M, Ryan M et al. Hypertonic dextrose injections (prolotherapy) for knee osteoarthritis: results of a single-arm uncontrolled study with 1-year follow-up. *J Altern Complement Med* 2012; 18(4):408-414.

(296) Sands GH, Brown PB, Essex MN. The Efficacy of Continuous Versus Intermittent Celecoxib Treatment in Osteoarthritis Patients with Body Mass Index >/=30 and <30 kg/m(2.). *Open Rheumatol J* 2013; 7:32-37.

(297) Bartlett SJ, Ling SM, Mayo NE, Scott SC, Bingham CO, III. Identifying common trajectories of joint space narrowing over two years in knee osteoarthritis. *Arthritis Care Res (Hoboken)* 2011; 63(12):1722-1728.

(298) Bingham CO, III, Smugar SS, Wang H, Peloso PM, Gammaitoni A. Predictors of response to cyclo-oxygenase-2 inhibitors in osteoarthritis: pooled results from two identical trials comparing etoricoxib, celecoxib, and placebo. *Pain Med* 2011; 12(3):352-361.

(299) Nishimura A, Hasegawa M, Kato K, Yamada T, Uchida A, Sudo A. Risk factors for the incidence and progression of radiographic osteoarthritis of the knee among Japanese. *Int Orthop* 2011; 35(6):839-843.

(300) Richette P, Poitou C, Garnero P, Vicaut E, Bouillot JL, Lacorte JM et al. Benefits of massive weight loss on symptoms, systemic inflammation and cartilage turnover in obese patients with knee osteoarthritis. *Ann Rheum Dis* 2011; 70(1):139-144.

(301) Woollard JD, Gil AB, Sparto P, Kwoh CK, Piva SR, Farrokhi S et al. Change in knee cartilage volume in individuals completing a therapeutic exercise program for knee osteoarthritis. *J Orthop Sports Phys Ther* 2011; 41(10):708-722.

(302) Yusuf E, Bijsterbosch J, Slagboom PE, Rosendaal FR, Huizinga TW, Kloppenburg M. Body mass index and alignment and their interaction as risk factors for progression of knees with radiographic signs of osteoarthritis. *Osteoarthritis Cartilage* 2011; 19(9):1117-1122.

(303) Shea MK, Houston DK, Nicklas BJ, Messier SP, Davis CC, Miller ME et al. The effect of randomization to weight loss on total mortality in older overweight and obese adults: the ADAPT Study. *J Gerontol A Biol Sci Med Sci* 2010; 65(5):519-525.

(304) Eckstein F, Maschek S, Wirth W, Hudelmaier M, Hitzl W, Wyman B et al. One year change of knee cartilage morphology in the first release of participants from the Osteoarthritis Initiative progression subcohort: association with sex, body mass index, symptoms and radiographic osteoarthritis status. *Ann Rheum Dis* 2009; 68(5):674-679.

(305) Le Graverand MP, Brandt K, Mazzuca SA, Raunig D, Vignon E. Progressive increase in body mass index is not associated with a progressive increase in joint space narrowing in obese women with osteoarthritis of the knee. *Ann Rheum Dis* 2009; 68(11):1734-1738.

(306) Botha-Scheepers S, Dougados M, Ravaud P, Hellio Le Graverand MP, Watt I, Breedveld FC et al. Effect of medial tibial plateau alignment on serial radiographs on the capacity to predict progression of knee osteoarthritis. *Osteoarthritis Cartilage* 2008; 16(2):272-276.

(307) Davies-Tuck ML, Wluka AE, Wang Y, Teichtahl AJ, Jones G, Ding C et al. The natural history of cartilage defects in people with knee osteoarthritis. *Osteoarthritis Cartilage* 2008; 16(3):337-342.

(308) Pelletier JP, Raynauld JP, Berthiaume MJ, Abram F, Choquette D, Haraoui B et al. Risk factors associated with the loss of cartilage volume on weight-bearing areas in knee osteoarthritis patients assessed by quantitative magnetic resonance imaging: a longitudinal study. *Arthritis Res Ther* 2007; 9(4):R74.

(309) Reijman M, Pols HA, Bergink AP, Hazes JM, Belo JN, Lievense AM et al. Body mass index associated with onset and progression of osteoarthritis of the knee but not of the hip: the Rotterdam Study. *Ann Rheum Dis* 2007; 66(2):158-162.

(310) Raynauld JP, Martel-Pelletier J, Berthiaume MJ, Beaudoin G, Choquette D, Haraoui B et al. Long term evaluation of disease progression through the quantitative magnetic resonance imaging of symptomatic knee osteoarthritis patients: correlation with clinical symptoms and radiographic changes. *Arthritis Res Ther* 2006; 8(1):R21.

(311) Wluka AE, Forbes A, Wang Y, Hanna F, Jones G, Cicuttini FM. Knee cartilage loss in symptomatic knee osteoarthritis over 4.5 years. *Arthritis Res Ther* 2006; 8(4):R90.

(312) Sharma L, Cahue S, Song J, Hayes K, Pai YC, Dunlop D. Physical functioning over three years in knee osteoarthritis: role of psychosocial, local mechanical, and neuromuscular factors. *Arthritis Rheum* 2003; 48(12):3359-3370.

(313) Cicuttini F, Wluka A, Wang Y, Stuckey S. The determinants of change in patella cartilage volume in osteoarthritic knees. *J Rheumatol* 2002; 29(12):2615-2619.

(314) Wolfe F, Lane NE. The longterm outcome of osteoarthritis: rates and predictors of joint space narrowing in symptomatic patients with knee osteoarthritis. *J Rheumatol* 2002; 29(1):139-146.

(315) Detora LM, Krupa D, Bolognese J, Sperling RS, Ehrich EW. Rofecoxib shows consistent efficacy in osteoarthritis clinical trials, regardless of specific patient demographic and disease factors. *J Rheumatol* 2001; 28(11):2494-2503.

(316) Cooper C, Snow S, McAlindon TE, Kellingray S, Stuart B, Coggon D et al. Risk factors for the incidence and progression of radiographic knee osteoarthritis. *Arthritis Rheum* 2000; 43(5):995-1000.

(317) Harris PA, Hart DJ, Dacre JE, Huskisson EC, Spector TD. The progression of radiological hand osteoarthritis over ten years: a clinical follow-up study. *Osteoarthritis Cartilage* 1994; 2(4):247-252.

(318) Ledingham J, Dawson S, Preston B, Milligan G, Doherty M. Radiographic progression of hospital referred osteoarthritis of the hip. *Ann Rheum Dis* 1993; 52(4):263-267.

(319) Schouten JS, van den Ouweland FA, Valkenburg HA. A 12 year follow up study in the general population on prognostic factors of cartilage loss in osteoarthritis of the knee. *Ann Rheum Dis* 1992; 51(8):932-937.

(320) Berkhout B, Macfarlane JD, Cats A. Symptomatic osteoarthrosis of the knee: a follow-up study. *Br J Rheumatol* 1985; 24(1):40-45.

(321) Ahn JH, Kang HW, Yang TY, Lee JY. Risk Factors for Radiographic Progression of Osteoarthritis After Meniscus Allograft Transplantation. *Arthroscopy* 2016; 32(12):2539-2546.

(322) Liu Y, Hazlewood GS, Kaplan GG, Eksteen B, Barnabe C. Impact of Obesity on Remission and Disease Activity in Rheumatoid Arthritis: A Systematic Review and Meta-Analysis. *Arthritis Care Res (Hoboken)* 2017; 69(2):157-165.

(323) Lupoli R, Pizzicato P, Scalera A, Ambrosino P, Amato M, Peluso R et al. Impact of body weight on the achievement of minimal disease activity in patients with rheumatic diseases: a systematic review and meta-analysis. *Arthritis Res Ther* 2016; 18(1):297.

(324) Baghdadi LR, Woodman RJ, Shanahan EM, Mangoni AA. The impact of traditional cardiovascular risk factors on cardiovascular outcomes in patients with rheumatoid arthritis: a systematic review and meta-analysis. *PLoS One* 2015; 10(2):e0117952.

(325) Baker JF, Stokes A, Mikuls TR, George M, England BR, Sayles H et al. Current and early life weight and associations with mortality in rheumatoid arthritis. *Clin Exp Rheumatol* 2019.

(326) Hirose W, Harigai M, Uchiyama T, Itoh K, Ishizuka T, Matsumoto M et al. Low body mass index and lymphocytopenia associate with Mycobacterium avium complex pulmonary disease in patients with rheumatoid arthritis. *Mod Rheumatol* 2019; 29(1):105-112.

(327) Lechtenboehmer CA, Jaeger VK, Kyburz D, Walker UA, Hugle T. Brief Report: Influence of Disease Activity in Rheumatoid Arthritis on Radiographic Progression of Concomitant Interphalangeal Joint Osteoarthritis. *Arthritis Rheumatol* 2019; 71(1):43-49.

(328) England BR, Baker JF, Sayles H, Michaud K, Caplan L, Davis LA et al. Body Mass Index, Weight Loss, and Cause-Specific Mortality in Rheumatoid Arthritis. *Arthritis Care Res (Hoboken)* 2018; 70(1):11-18.

(329) Nikiphorou E, Norton S, Young A, Dixey J, Walsh D, Helliwell H et al. The association of obesity with disease activity, functional ability and quality of life in early rheumatoid arthritis: data from the Early Rheumatoid Arthritis Study/Early Rheumatoid Arthritis Network UK prospective cohorts. *Rheumatology (Oxford)* 2018.

(330) Rydell E, Forslind K, Nilsson JA, Jacobsson LTH, Turesson C. Smoking, body mass index, disease activity, and the risk of rapid radiographic progression in patients with early rheumatoid arthritis. *Arthritis Res Ther* 2018; 20(1):82.

(331) Schulman E, Bartlett SJ, Schieir O, Andersen KM, Boire G, Pope JE et al. Overweight, Obesity, and the Likelihood of Achieving Sustained Remission in Early Rheumatoid Arthritis: Results From a Multicenter Prospective Cohort Study. *Arthritis Care Res (Hoboken)* 2018; 70(8):1185-1191.

(332) Smolen JS, Szumski A, Koenig AS, Jones TV, Marshall L. Predictors of remission with etanercept-methotrexate induction therapy and loss of remission with etanercept maintenance, reduction, or withdrawal in moderately active rheumatoid arthritis: results of the PRESERVE trial. *Arthritis Res Ther* 2018; 20(1):8.

(333) Sparks JA, Chang SC, Nguyen US, Barbhaiya M, Tedeschi SK, Lu B et al. Weight Change During the Early Rheumatoid Arthritis Period and Risk of Subsequent Mortality in Women With Rheumatoid Arthritis and Matched Comparators. *Arthritis Rheumatol* 2018; 70(1):18-29.

(334) van der Heijde D, Durez P, Schett G, Naredo E, Ostergaard M, Meszaros G et al. Structural damage progression in patients with early rheumatoid arthritis treated with methotrexate, baricitinib, or baricitinib plus methotrexate based on clinical response in the phase 3 RA-BEGIN study. *Clin Rheumatol* 2018; 37(9):2381-2390.

(335) Bird P, Nicholls D, Barrett R, de JJ, Griffiths H, Roberts L et al. Longitudinal study of clinical prognostic factors in patients with early rheumatoid arthritis: the PREDICT study. *Int J Rheum Dis* 2017; 20(4):460-468.

(336) D'Agostino MA, Alten R, Mysler E, Le BM, Ye J, Murthy B et al. Body mass index and clinical response to intravenous or subcutaneous abatacept in patients with rheumatoid arthritis. *Clin Rheumatol* 2017; 36(12):2655-2665.

(337) George MD, Ostergaard M, Conaghan PG, Emery P, Baker DG, Baker JF. Obesity and rates of clinical remission and low MRI inflammation in rheumatoid arthritis. *Ann Rheum Dis* 2017; 76(10):1743-1746.

(338) Iannone F, Courvoisier DS, Gottenberg JE, Hernandez MV, Lie E, Canhao H et al. Body mass does not impact the clinical response to intravenous abatacept in patients with rheumatoid arthritis. Analysis from the "pan-European registry collaboration for abatacept (PANABA). *Clin Rheumatol* 2017; 36(4):773-779.

(339) Joo YB, Bang SY, Ryu JA, Lee S, Lee HS, Bae SC. Predictors of severe radiographic progression in patients with early rheumatoid arthritis: A Prospective observational cohort study. *Int J Rheum Dis* 2017; 20(10):1437-1446.

(340) Levitsky A, Brismar K, Hafstrom I, Hambardzumyan K, Lourdudoss C, van Vollenhoven RF et al. Obesity is a strong predictor of worse clinical outcomes and treatment responses in early rheumatoid arthritis: results from the SWEFOT trial. *RMD Open* 2017; 3(2):e000458.

(341) Mariette X, Alten R, Nusslein HG, Galeazzi M, Lorenz HM, Cantagrel A et al. The effect of body mass index on clinical response to abatacept as a first-line biologic for rheumatoid arthritis: 6-month results from the 2-year, observational, prospective ACTION study. *Joint Bone Spine* 2017; 84(5):571-576.

(342) Miwa Y, Saito M, Furuya H, Yanai R, Ikari Y, Hayashi T et al. Clinical Characteristics of Rheumatoid Arthritis Patients Achieving Functional Remission after Six Months of Non-tumor Necrosis Factor Biological Disease-Modifying Antirheumatic Drugs (DMARDs) Treatment. *Intern Med* 2017; 56(17):2271-2275.

(343) Ramirez J, Narvaez JA, Ruiz-Esquide V, Hernandez-Ganan J, Cuervo A, Inciarte-Mundo J et al. Clinical and sonographic biomarkers of structural damage progression in RA patients in clinical remission: A prospective study with 12 months follow-up. *Semin Arthritis Rheum* 2017; 47(3):303-309.

(344) Feldthusen C, Grimby-Ekman A, Forsblad-d'Elia H, Jacobsson L, Mannerkorpi K. Explanatory factors and predictors of fatigue in persons with rheumatoid arthritis: A longitudinal study. *J Rehabil Med* 2016; 48(5):469-476.

(345) Gardette A, Ottaviani S, Sellam J, Berenbaum F, Liote F, Fautrel B et al. Body mass index and response to abatacept in rheumatoid arthritis. *Eur J Clin Invest* 2016; 46(12):1048-1052.

(346) Gardette A, Ottaviani S, Sellam J, Berenbaum F, Liote F, Meyer A et al. Body mass index and response to tocilizumab in rheumatoid arthritis: a real life study. *Clin Rheumatol* 2016; 35(4):857-861.

(347) McWilliams DF, Walsh DA. Factors predicting pain and early discontinuation of tumour necrosis factor-alpha-inhibitors in people with rheumatoid arthritis: results from the British society for rheumatology biologics register. *BMC Musculoskelet Disord* 2016; 17:337.

(348) Tantayakom P, Koolvisoot A, Arromdee E, Chiowchanwisawakit P, Muangchan C, Katchamart W. Metabolic syndrome is associated with disease activity in patients with rheumatoid arthritis. *Joint Bone Spine* 2016; 83(5):563-567.

(349) Baker JF, Billig E, Michaud K, Ibrahim S, Caplan L, Cannon GW et al. Weight Loss, the Obesity Paradox, and the Risk of Death in Rheumatoid Arthritis. *Arthritis Rheumatol* 2015; 67(7):1711-1717.

(350) Iannone F, Fanizzi R, Notarnicola A, Scioscia C, Anelli MG, Lapadula G. Obesity reduces the drug survival of second line biological drugs following a first TNF-alpha inhibitor in rheumatoid arthritis patients. *Joint Bone Spine* 2015; 82(3):187-191.

(351) Pers YM, Godfrin-Valnet M, Lambert J, Fortunet C, Constant E, Mura T et al. Response to tocilizumab in rheumatoid arthritis is not influenced by the body mass index of the patient. *J Rheumatol* 2015; 42(4):580-584.

(352) Kim HW, Park JK, Yang JA, Yoon YI, Lee EY, Song YW et al. Comparison of tuberculosis incidence in ankylosing spondylitis and rheumatoid arthritis during tumor necrosis factor inhibitor treatment in an intermediate burden area. *Clin Rheumatol* 2014; 33(9):1307-1312.

(353) Ochi K, Go Y, Furuya T, Ikari K, Taniguchi A, Yamanaka H et al. Risk factors associated with the occurrence of distal radius fractures in Japanese patients with rheumatoid arthritis: a prospective observational cohort study. *Clin Rheumatol* 2014; 33(4):477-483.

(354) Sandberg ME, Bengtsson C, Kallberg H, Wesley A, Klareskog L, Alfredsson L et al. Overweight decreases the chance of achieving good response and low disease activity in early rheumatoid arthritis. *Ann Rheum Dis* 2014; 73(11):2029-2033.

(355) Ajeganova S, Andersson ML, Hafstrom I. Association of obesity with worse disease severity in rheumatoid arthritis as well as with comorbidities: a long-term followup from disease onset. *Arthritis Care Res (Hoboken)* 2013; 65(1):78-87.

(356) Gremese E, Carletto A, Padovan M, Atzeni F, Raffeiner B, Giardina AR et al. Obesity and reduction of the response rate to anti-tumor necrosis factor alpha in rheumatoid arthritis: an approach to a personalized medicine. *Arthritis Care Res (Hoboken)* 2013; 65(1):94-100.

(357) Kanecki K, Tyszko P, Wislowska M, Lyczkowska-Piotrowska J. Preliminary report on a study of health-related quality of life in patients with rheumatoid arthritis. *Rheumatol Int* 2013; 33(2):429-434.

(358) Dirven L, Huizinga TW, Allaart CF. Risk factors for reported influenza and influenza-like symptoms in patients with rheumatoid arthritis. *Scand J Rheumatol* 2012; 41(5):359-365.

(359) Wevers-de BK, Visser K, Heimans L, Ronday HK, Molenaar E, Groenendael JH et al. Remission induction therapy with methotrexate and prednisone in patients with early rheumatoid and undifferentiated arthritis (the IMPROVED study). *Ann Rheum Dis* 2012; 71(9):1472-1477.

(360) Wolfe F, Michaud K. Effect of body mass index on mortality and clinical status in rheumatoid arthritis. *Arthritis Care Res (Hoboken)* 2012; 64(10):1471-1479.

(361) de Rooy DP, van der Linden MP, Knevel R, Huizinga TW, van der Helm-van Mil AH. Predicting arthritis outcomes--what can be learned from the Leiden Early Arthritis Clinic? *Rheumatology (Oxford)* 2011; 50(1):93-100.

(362) Klaasen R, Wijbrandts CA, Gerlag DM, Tak PP. Body mass index and clinical response to infliximab in rheumatoid arthritis. *Arthritis Rheum* 2011; 63(2):359-364.

(363) Liao KP, Weinblatt ME, Cui J, Iannaccone C, Chibnik LB, Lu B et al. Clinical predictors of erosion-free status in rheumatoid arthritis: a prospective cohort study. *Rheumatology (Oxford)* 2011; 50(8):1473-1479.

(364) Tekaya R, Sahli H, Zribi S, Mahmoud I, Ben Hadj YC, Abdelmoula L et al. [Obesity has a protective effect on radiographic joint damage in rheumatoid arthritis]. *Tunis Med* 2011; 89(5):462-465.

(365) Pye SR, Marshall T, Gaffney K, Silman AJ, Symmons DP, O'Neill TW. Influence of arthritis and non-arthritis related factors on areal bone mineral density (BMDa) in women with longstanding inflammatory polyarthritis: a primary care based inception cohort. *BMC Musculoskelet Disord* 2010; 11:106.

(366) Verstappen SM, Bakker MF, Heurkens AH, van der Veen MJ, Kruize AA, Geurts MA et al. Adverse events and factors associated with toxicity in patients with early rheumatoid arthritis treated with methotrexate tight control therapy: the CAMERA study. *Ann Rheum Dis* 2010; 69(6):1044-1048.

(367) Furuya T, Yamagiwa K, Ikai T, Inoue E, Taniguchi A, Momohara S et al. Associated factors for falls and fear of falling in Japanese patients with rheumatoid arthritis. *Clin Rheumatol* 2009; 28(11):1325-1330.

(368) Hashimoto J, Garnero P, van der Heijde D, Miyasaka N, Yamamoto K, Kawai S et al. A combination of biochemical markers of cartilage and bone turnover, radiographic damage and body mass index to predict the progression of joint destruction in patients with rheumatoid arthritis treated with disease-modifying anti-rheumatic drugs. *Mod Rheumatol* 2009; 19(3):273-282.

(369) van der Helm-van Mil AH, van der Kooij SM, Allaart CF, Toes RE, Huizinga TW. A high body mass index has a protective effect on the amount of joint destruction in small joints in early rheumatoid arthritis. *Ann Rheum Dis* 2008; 67(6):769-774.

(370) Cohen JD, Dougados M, Goupille P, Cantagrel A, Meyer O, Sibilia J et al. Health assessment questionnaire score is the best predictor of 5-year quality of life in early rheumatoid arthritis. *J Rheumatol* 2006; 33(10):1936-1941.

(371) Escalante A, Haas RW, del R, I. Paradoxical effect of body mass index on survival in rheumatoid arthritis: role of comorbidity and systemic inflammation. *Arch Intern Med* 2005; 165(14):1624-1629.

(372) Maradit-Kremers H, Nicola PJ, Crowson CS, Ballman KV, Gabriel SE. Cardiovascular death in rheumatoid arthritis: a population-based study. *Arthritis Rheum* 2005; 52(3):722-732.

(373) Skoldstam L, Brudin L, Hagfors L, Johansson G. Weight reduction is not a major reason for improvement in rheumatoid arthritis from lacto-vegetarian, vegan or Mediterranean diets. *Nutr J* 2005; 4:15.

(374) Maradit-Kremers H, Nicola PJ, Crowson CS, Ballman KV, Gabriel SE. Prognostic importance of low body mass index in relation to cardiovascular mortality in rheumatoid arthritis. *Arthritis Rheum* 2004; 50(11):3450-3457.

(375) Hoekstra M, van Ede AE, Haagsma CJ, van de Laar MA, Huizinga TW, Kruijsen MW et al. Factors associated with toxicity, final dose, and efficacy of methotrexate in patients with rheumatoid arthritis. *Ann Rheum Dis* 2003; 62(5):423-426.

(376) Krishnan E, Lingala B, Bruce B, Fries JF. Disability in rheumatoid arthritis in the era of biological treatments. *Ann Rheum Dis* 2012; 71(2):213-218.

(377) Kreps DJ, Halperin F, Desai SP, Zhang ZZ, Losina E, Olson AT et al. Association of weight loss with improved disease activity in patients with rheumatoid arthritis: A retrospective analysis using electronic medical record data. *Int J Clin Rheumtol* 2018; 13(1):1-10.

(378) Mori S, Yoshitama T, Hidaka T, Sakai F, Hasegawa M, Hashiba Y et al. Comparative risk of hospitalized infection between biological agents in rheumatoid arthritis patients: A multicenter retrospective cohort study in Japan. *PLoS One* 2017; 12(6):e0179179.

(379) Rashid N, Lin AT, Aranda G, Jr., Lin KJ, Guerrero VN, Nadkarni A et al. Rates, factors, reasons, and economic impact associated with switching in rheumatoid arthritis patients newly initiated on biologic disease modifying anti-rheumatic drugs in an integrated healthcare system. *J Med Econ* 2016; 19(6):568-575.

(380) Ottaviani S, Gardette A, Roy C, Tubach F, Gill G, Palazzo E et al. Body Mass Index and response to rituximab in rheumatoid arthritis. *Joint Bone Spine* 2015; 82(6):432-436.

(381) Ottaviani S, Gardette A, Tubach F, Roy C, Palazzo E, Gill G et al. Body mass index and response to infliximab in rheumatoid arthritis. *Clin Exp Rheumatol* 2015; 33(4):478-483.

(382) Sparks JA, Halperin F, Karlson JC, Karlson EW, Bermas BL. Impact of Bariatric Surgery on Patients With Rheumatoid Arthritis. *Arthritis Care Res (Hoboken)* 2015; 67(12):1619-1626.

(383) Gonzalez A, Maradit KH, Crowson CS, Ballman KV, Roger VL, Jacobsen SJ et al. Do cardiovascular risk factors confer the same risk for cardiovascular outcomes in rheumatoid arthritis patients as in non-rheumatoid arthritis patients? *Ann Rheum Dis* 2008; 67(1):64-69.

(384) Kent PD, Luthra HS, Michet C, Jr. Risk factors for methotrexate-induced abnormal laboratory monitoring results in patients with rheumatoid arthritis. *J Rheumatol* 2004; 31(9):1727-1731.

(385) Figueiredo-Braga M, Cornaby C, Bernardes M, Figueiredo M, Mesquita CDS, Costa L et al. Correlation between physical markers and psychiatric health in a Portuguese systemic lupus erythematosus cohort: The role of suffering in chronic autoimmune disease. *PLoS One* 2018; 13(4):e0195579.

(386) Jacobs J, Korswagen LA, Schilder AM, van Tuyl LH, Dijkmans BA, Lems WF et al. Six-year follow-up study of bone mineral density in patients with systemic lupus erythematosus. *Osteoporos Int* 2013; 24(6):1827-1833.

(387) Katz P, Yazdany J, Julian L, Trupin L, Margaretten M, Yelin E et al. Impact of obesity on functioning among women with systemic lupus erythematosus. *Arthritis Care Res (Hoboken)* 2011; 63(10):1357-1364.

(388) Chaiamnuay S, Bertoli AM, Fernandez M, Apte M, Vila LM, Reveille JD et al. The impact of increased body mass index on systemic lupus erythematosus: data from LUMINA, a multiethnic cohort (LUMINA XLVI) [corrected]. *J Clin Rheumatol* 2007; 13(3):128-133.

(389) Chaiamnuay S, Bertoli AM, Roseman JM, McGwin G, Apte M, Duran S et al. African-American and Hispanic ethnicities, renal involvement and obesity predispose to hypertension in systemic lupus erythematosus: results from LUMINA, a multiethnic cohort (LUMINAXLV). *Ann Rheum Dis* 2007; 66(5):618-622.

(390) Uaratanawong S, Deesomchok U, Hiransuttikul N, Uaratanawong S. Four years follow-up of bone mineral density change in premenopausal women with systemic lupus erythematosus. *J Med Assoc Thai* 2004; 87(11):1374-1379.

(391) Bruce IN, Gladman DD, Urowitz MB. Detection and modification of risk factors for coronary artery disease in patients with systemic lupus erythematosus: a quality improvement study. *Clin Exp Rheumatol* 1998; 16(4):435-440.

(392) Petri M, Perez-Gutthann S, Spence D, Hochberg MC. Risk factors for coronary artery disease in patients with systemic lupus erythematosus. *Am J Med* 1992; 93(5):513-519.

(393) Hernandez-Breijo B, Plasencia-Rodriguez C, Navarro-Compan V, Martinez-Feito A, Jochems A, Kneepkens EL et al. Association between concomitant csDMARDs and clinical response to TNF inhibitors in overweight patients with axial spondyloarthritis. *Arthritis Res Ther* 2019; 21(1):66.

(394) Jeong H, Eun YH, Kim IY, Park EJ, Kim H, Lee J et al. Effect of tumor necrosis factor alpha inhibitors on spinal radiographic progression in patients with ankylosing spondylitis. *Int J Rheum Dis* 2018; 21(5):1098-1105.

(395) Pedersen SJ, Weber U, Said-Nahal R, Sorensen IJ, Loft AG, Kollerup G et al. Structural progression rate decreases over time on serial radiography and magnetic resonance imaging of sacroiliac joints and spine in a five-year follow-up study of patients with ankylosing spondylitis treated with tumour necrosis factor inhibitor. *Scand J Rheumatol* 2018;1-13.

(396) Maas F, Arends S, Wink FR, Bos R, Bootsma H, Brouwer E et al. Ankylosing spondylitis patients at risk of poor radiographic outcome show diminishing spinal radiographic progression during long-term treatment with TNF-alpha inhibitors. *PLoS One* 2017; 12(6):e0177231.

(397) Maas F, Spoorenberg A, van der Slik BPG, van d, V, Brouwer E, Bootsma H et al. Clinical Risk Factors for the Presence and Development of Vertebral Fractures in Patients With Ankylosing Spondylitis. *Arthritis Care Res (Hoboken)* 2017; 69(5):694-702.

(398) Micheroli R, Hebeisen M, Wildi LM, Exer P, Tamborrini G, Bernhard J et al. Impact of obesity on the response to tumor necrosis factor inhibitors in axial spondyloarthritis. *Arthritis Res Ther* 2017; 19(1):164.

(399) Hwang J, Kim HM, Jeong H, Lee J, Ahn JK, Koh EM et al. Higher body mass index and anti-drug antibodies predict the discontinuation of anti-TNF agents in Korean patients with axial spondyloarthritis. *Rev Bras Reumatol Engl Ed* 2017; 57(4):311-319.

(400) van Weely SF, Kneepkens EL, Nurmohamed MT, Dekker J, van der Horst-Bruinsma IE. Continuous Improvement of Physical Functioning in Ankylosing Spondylitis Patients by Tumor Necrosis Factor Inhibitors: Three-Year Followup and Predictors. *Arthritis Care Res (Hoboken)* 2016; 68(10):1522-1529.

(401) Maas F, Spoorenberg A, Brouwer E, Bos R, Efde M, Chaudhry RN et al. Spinal radiographic progression in patients with ankylosing spondylitis treated with TNF-alpha blocking therapy: a prospective longitudinal observational cohort study. *PLoS One* 2015; 10(4):e0122693.

(402) Gremese E, Bernardi S, Bonazza S, Nowik M, Peluso G, Massara A et al. Body weight, gender and response to TNF-alpha blockers in axial spondyloarthritis. *Rheumatology (Oxford)* 2014; 53(5):875-881.

(403) Ottaviani S, Allanore Y, Tubach F, Forien M, Gardette A, Pasquet B et al. Body mass index influences the response to infliximab in ankylosing spondylitis. *Arthritis Res Ther* 2012; 14(3):R115.

(404) Di Minno MN, Peluso R, Iervolino S, Russolillo A, Lupoli R, Scarpa R. Weight loss and achievement of minimal disease activity in patients with psoriatic arthritis starting treatment with tumour necrosis factor alpha blockers. *Ann Rheum Dis* 2014; 73(6):1157-1162.

(405) Klingberg E, Bilberg A, Bjorkman S, Hedberg M, Jacobsson L, Forsblad-d'Elia H et al. Weight loss improves disease activity in patients with psoriatic arthritis and obesity: an interventional study. *Arthritis Res Ther* 2019; 21(1):17.

(406) Polachek A, Li S, Chandran V, Gladman DD. Clinical Enthesitis in a Prospective Longitudinal Psoriatic Arthritis Cohort: Incidence, Prevalence, Characteristics, and Outcome. *Arthritis Care Res (Hoboken)* 2017; 69(11):1685-1691.

(407) Hojgaard P, Glintborg B, Kristensen LE, Gudbjornsson B, Love TJ, Dreyer L. The influence of obesity on response to tumour necrosis factor-alpha inhibitors in psoriatic arthritis: results from the DANBIO and ICEBIO registries. *Rheumatology (Oxford)* 2016; 55(12):2191-2199.

(408) Eder L, Thavaneswaran A, Chandran V, Cook RJ, Gladman DD. Obesity is associated with a lower probability of achieving sustained minimal disease activity state among patients with psoriatic arthritis. *Ann Rheum Dis* 2015; 74(5):813-817.

(409) Mease PJ, Collier DH, Saunders KC, Li G, Kremer JM, Greenberg JD. Comparative effectiveness of biologic monotherapy versus combination therapy for patients with psoriatic arthritis: results from the Corrona registry. *RMD Open* 2015; 1(1):e000181.

(410) Di Minno MN, Peluso R, Iervolino S, Lupoli R, Russolillo A, Scarpa R et al. Obesity and the prediction of minimal disease activity: a prospective study in psoriatic arthritis. *Arthritis Care Res (Hoboken)* 2013; 65(1):141-147.

(411) Iannone F, Fanizzi R, Scioscia C, Anelli MG, Lapadula G. Body mass does not affect the remission of psoriatic arthritis patients on anti-TNF-alpha therapy. *Scand J Rheumatol* 2013; 42(1):41-44.

(412) Haddad A, Thavaneswaran A, Toloza S, Chandran V, Gladman DD. Diffuse idiopathic skeletal hyperostosis in psoriatic arthritis. *J Rheumatol* 2013; 40(8):1367-1373.

(413) Marini C, Formichi B, Bauleo C, Michelassi C, Airo E, Rossi G et al. Survival protection by bodyweight in isolated scleroderma-related pulmonary artery hypertension. *Intern Emerg Med* 2016; 11(7):941-952.

(414) Assassi S, Del JD, Sutter K, McNearney TA, Reveille JD, Karnavas A et al. Clinical and genetic factors predictive of mortality in early systemic sclerosis. *Arthritis Rheum* 2009; 61(10):1403-1411.

(415) Nielsen SM, Bartels EM, Henriksen M, Waehrens EE, Gudbergsen H, Bliddal H et al. Weight loss for overweight and obese individuals with gout: a systematic review of longitudinal studies. *Ann Rheum Dis* 2017; 76(11):1870-1882.

(416) Dessein PH, Shipton EA, Stanwix AE, Joffe BI, Ramokgadi J. Beneficial effects of weight loss associated with moderate calorie/carbohydrate restriction, and increased proportional intake of protein and unsaturated fat on serum urate and lipoprotein levels in gout: a pilot study. *Ann Rheum Dis* 2000; 59(7):539-543.

(417) Nguyen UD, Zhang Y, Louie-Gao Q, Niu J, Felson DT, LaValley MP et al. Obesity Paradox in Recurrent Attacks of Gout in Observational Studies: Clarification and Remedy. *Arthritis Care Res (Hoboken)* 2017; 69(4):561-566.

(418) Romero-Talamas H, Daigle CR, Aminian A, Corcelles R, Brethauer SA, Schauer PR. The effect of bariatric surgery on gout: a comparative study. *Surg Obes Relat Dis* 2014; 10(6):1161-1165.

(419) Su BY, Lai HM, Chen CJ, Chen YC, Chiu CK, Lin KM et al. Ischemia heart disease and greater waist circumference are risk factors of renal function deterioration in male gout patients. *Clin Rheumatol* 2008; 27(5):581-586.

(420) Abhishek A, Valdes AM, Zhang W, Doherty M. Association of Serum Uric Acid and Disease Duration With Frequent Gout Attacks: A Case-Control Study. *Arthritis Care Res (Hoboken)* 2016; 68(10):1573-1577.

(421) Alvarez-Nemegyei J, Cen-Piste JC, Medina-Escobedo M, Villanueva-Jorge S. Factors associated with musculoskeletal disability and chronic renal failure in clinically diagnosed primary gout. *J Rheumatol* 2005; 32(10):1923-1927.
